# *Ank3* loss in adult forebrain excitatory neurons disrupts behavior, neuronal activity, membrane proteome, and myelination

**DOI:** 10.1073/pnas.2606900123

**Published:** 2026-08-25

**Authors:** Sehyoun Yoon, Marc Dos Santos, Natalia Khalatyan, Jeffrey N. Savas, Peter Penzes

**Affiliations:** ^a^Department of Neuroscience, Northwestern University Feinberg School of Medicine, Chicago, IL 60611; ^b^Department of Neurology, Northwestern University Feinberg School of Medicine, Chicago, IL 60611; ^c^Department of Psychiatry and Behavioral Sciences, Northwestern University Feinberg School of Medicine, Chicago, IL 60611; ^d^Department of Pharmacology, Northwestern University Feinberg School of Medicine, Chicago, IL 60611; ^e^Center for Autism and Neurodevelopment, Northwestern University Feinberg School of Medicine, Chicago, IL 60611

**Keywords:** *Ank3*, Mbp, neuronal activity, proteomics, neuropsychiatric disorders

## Abstract

*ANK3*, encoding the scaffolding protein ankyrin-G, is a major risk gene for bipolar disorder and schizophrenia, but its cellular and circuit-level mechanisms remain poorly defined. Here, we demonstrate that deletion of *Ank3* in forebrain excitatory neurons-either prenatally (*Ank3^–/^^−^:Emx1-Cre*) or in adolescence (*Ank3^–/^^–^:CaMKIIα-Cre*) leads to convergent behavioral phenotypes in adulthood, including hyperactivity, reduced anxiety-like behavior, and decreased depression-like responses. Calcium imaging in cultured neurons and acute brain slices revealed that ankyrin-G loss reduces both spontaneous and evoked neuronal activity. Quantitative proteomic profiling of membrane-enriched cortical fractions uncovered widespread remodeling of the synaptic proteome, including upregulation of the kinase Taok2 and unexpected downregulation of myelin basic protein (Mbp), a structural component of oligodendrocyte-derived myelin. Importantly, chronic lithium treatment, known to reverse behavioral abnormalities in *Ank3*-deficient mice, also restored Mbp expression. Together, our findings identify ankyrin-G as a molecular bridge between excitatory neuronal activity, synaptic structure, and myelin-associated protein expression, revealing a pathway by which *ANK3* variants may contribute to neuropsychiatric disease.

## Significance

This study demonstrates that deletion of *Ank3* in adult excitatory neurons is sufficient to induce behavioral abnormalities resembling bipolar disorder-like phenotypes. Ankyrin-G loss reduces neuronal activity and leads to a striking downregulation of myelin basic protein (Mbp). Notably, chronic lithium treatment restores Mbp levels, providing a molecular correlate of its therapeutic effects and suggesting a remyelination-based mechanism of action. Together, these findings uncover a neuron-to-glia pathway underlying *ANK3*-associated psychiatric risk and provide a systems-level framework for understanding the cellular basis of lithium responsiveness in neuropsychiatric disorders.

Genome-wide association studies (GWAS) have significantly advanced our understanding of the genetic foundations of neuropsychiatric disorders. By identifying genetic risk loci, GWAS have provided a crucial framework for investigating the molecular mechanisms that may underlie these conditions. Among these risk genes, *ANK3* has emerged as a prominent candidate, suggesting that alterations in this gene may predispose individuals to a spectrum of psychiatric illnesses. Ankyrin-G, the protein encoded by *ANK3*, is well known for its association with bipolar disorder (BD) and schizophrenia (SZ) (1–3), and plays a critical role in maintaining the structural integrity and functional organization of the nervous system (4, 5).

Robust evidence from animal models indicates that mutations or deletions in *Ank3* contribute to a variety of behavioral abnormalities. Studies involving targeted alterations of *Ank3* have reported behavioral phenotypes that recapitulate features of human neuropsychiatric conditions. For example, decreased anxiety-like behaviors have been observed in *Ank3* cerebellum-specific heterozygous mice and in mice with *Ank3* knockdown via RNA interference in the dentate gyrus, both of which spent more time in the open arms of the elevated plus maze or in the light compartment of the light/dark box. Interestingly, lithium treatment was able to rescue these anxiety-related abnormalities (6, 7).

Intriguingly, whole-body *Ank3* heterozygous mice exhibited the opposite phenotype, with elevated anxiety-like behaviors in both the light/dark box and elevated plus maze tests, as well as increased immobility time in the forced swim test (FST) (8). In another study, a conditional knockout (cKO) mouse model with *Ank3* deletion specifically in pyramidal neurons of the adult forebrain exhibited hyperactivity and reduced anxiety-like behavior (9).

At the neuronal level, alterations in *Ank3* expression or structure have been associated with abnormal patterns of neuronal activation. These abnormalities include disrupted firing patterns and altered excitatory/inhibitory balance, both of which are critical for proper brain function (10–12). Such disruptions can initiate cascading effects on neuronal connectivity and signaling, ultimately impacting behavior and cognitive processes.

Ankyrin-G, the protein product of *Ank3*, interacts with a broad array of proteins including ion channels, transporters, cell adhesion molecules, cytoskeletal scaffolds, motor proteins, and signaling enzymes. It is localized predominantly at the axon initial segment (AIS) and postsynaptic sites, where it organizes membrane microdomains essential for synaptic structure and function (5). Given its crucial scaffolding role, deletion of the *Ank3* gene is likely to induce widespread proteomic changes in the neuronal environment, potentially disrupting the composition and function of synaptic proteins. These proteomic alterations may directly affect neuronal signaling pathways and help explain the behavioral abnormalities observed in *Ank3* deletion models. The association between gene deletions, proteomic shifts, and behavioral changes underscores the complexity of the molecular pathways underlying neuropsychiatric disorders, and offers potential targets for therapeutic intervention.

In this study, we investigate the role of ankyrin-G in the brain and its impact on behavior using two brain-specific *Ank3* cKO mouse models. Like *Ank3^–/^^–^*:*CaMKIIα*-Cre mice, *Ank3^–/^^–^*:*Emx1*-Cre mice display similar behavioral abnormalities, namely, hyperactivity and reduced depressive-like behavior without impairments in social interaction. In primary cortical neurons with *Ank3* knockdown, spontaneous calcium-dependent fluorescence changes, an indirect indicator of neuronal activity, exhibit reduced peak magnitudes. Similarly, calcium imaging in cortical slices from *Ank3^–/^^–^*:*CaMKIIα*-Cre mice reveals a decreased average calcium amplitude in response to potassium chloride (KCl)-induced neuronal depolarization.

To explore the molecular basis of these findings, we conducted a multiplexed proteomic analysis of forebrain membrane fraction (P2) using 10-plex tandem mass tag (TMT) labeling in combination with liquid chromatography-triple-stage mass spectrometry (LC/MS). This analysis identified a significant reduction in Mbp in *Ank*3*^–/^^–^*:*CaMKIIα*-Cre mice. Notably, chronic lithium treatment restored synaptic Mbp expression to wild-type levels. These findings suggest that Mbp downregulation may contribute to impaired neuronal excitability and the observed behavioral phenotypes in *Ank3* cKO mice. Moreover, they highlight a potential mechanism by which lithium modulates neuropsychiatric outcomes.

## Materials and Methods

### Animals.

*Ank3-*floxed mice (The Jackson Laboratory; stock #029797) (*Ank3*^fl/fl^) were crossed with either *CaMKIIα*-Cre (stock #005359) or *Emx1*-Cre (stock #005628) driver lines to generate cKO models with forebrain specific deletion of ankyrin-G (*Ank3*^−/−^:*CaMKIIα*-Cre or *Ank3*^−/−^:*Emx1*-Cre). Colonies were maintained by mating homozygous cKO mice with *Ank3*^fl/fl^ mice. Littermates lacking Cre recombinase expression (*Ank3*^fl/fl^) were used as controls. To generate *Ank3*^fl/fl^;*GCaMP3*^fl/fl^ mice, *Ank3*^fl/fl^ mice were crossed with RCL-*GCaMP3* mice (The Jackson Laboratory; stock #014538). After the 3rd generation, homozygous *Ank3*^fl/fl^;*GCaMP3*^fl/fl^ were bred with the same genotype to maintain the line. These mice were then crossed with *Ank3*^−/−^:*CaMKIIα*-Cre mice to generate *Ank3*^−/−^;*GCaMP3*^+/−^;*CaMKIIα*-Cre mice. Control mice expressing *GCaMP3* without *Ank3* deletion were obtained by crossing *GCaMP3*^fl/fl^ mice with *CaMKIIα*-Cre mice, resulting in *GCaMP3*^+/−^;*CaMKIIα*-Cre mice. All mice were group-housed in same-sex, mixed-genotype cages under specific pathogen-free conditions. Housing was maintained in a temperature- and humidity-controlled environment with a 12-h light/dark cycle. Mice had ad libitum access to autoclaved, membrane-filtered tap water, and standard rodent chow. Only male mice were used in the experiments. Behavioral assays were conducted on 12- to 14-wk-old animals. Mice aged 12 wk were used for TMT-LC/MS, neuronal recordings, and calcium imaging studies. All experimental procedures were approved by and conducted in accordance with the guidelines of the Institutional Animal Care and Use Committee (IACUC) at Northwestern University.

### Behavioral Analysis.

All behavioral assays were conducted during the light phase under standardized conditions in a dedicated behavioral testing room. Behavioral tracking and analysis were performed using EthoVision XT software (Noldus Information Technology).

#### Open field test.

Locomotor activity was assessed in a square open field arena (56 × 56 cm). Mice were individually placed in the center of the arena, and spontaneous activity was recorded for 30 min using an overhead video camera. Total distance traveled was quantified as an index of basal locomotor activity. Activity within a predefined center zone (28 × 28 cm) was also analyzed. The total distance traveled in the center zone and the frequency of center-zone entries were quantified.

#### Elevated zero maze.

Anxiety-like behavior was evaluated using an elevated circular zero maze (outer diameter: 56 cm) divided into four equal quadrants, two enclosed and two open, with opposing walls positioned 180° apart. Mice were placed in the enclosed section and allowed to freely explore the maze for 10 min. Time spent in open vs. closed quadrants was recorded and analyzed. A preference for closed quadrants was interpreted as increased anxiety-like behavior.

#### Light/dark box test.

The light/dark box consisted of a rectangular apparatus (50 × 25 × 25 cm) partitioned into equal light and dark compartments, with the dark half covered by a black lid. Mice were placed in the illuminated side and allowed free exploration for 5 min. Transitions between compartments and time spent in each were recorded as measures of anxiety-related behavior.

#### Forced swim test.

To assess behavioral despair, mice were placed in a transparent cylindrical tank (20 cm height × 14 cm diameter) filled with water (16 cm depth, 25 ± 1 °C). Each session lasted 6 min, and behavior was recorded using a digital video system. Quantitative analysis of mobility was conducted for the last 4 min (2 to 6 min). Immobility was defined as movement resulting in <5% change in the detected body area, with parameters confirmed by manual scoring.

#### Three-chamber social interaction test.

Sociability was assessed using a custom-built rectangular arena (76.2 × 30.5 × 30.5 cm) divided into three equal chambers. Mesh cylinders (11.4 cm diameter) were placed at each end, allowing sensory (visual, olfactory, auditory) interaction without physical contact. The assay included a 5-min habituation phase (with empty mesh enclosures), a 5-min rest period in a neutral holding cage, and a 5-min test phase in which a novel stimulus mouse (age-matched wild-type, 12 wk) was placed in one of the mesh cylinders. The position of the stimulus mouse was counterbalanced across subjects. The arena was divided into social, nonsocial, and central zones. Time spent in each zone and duration in close proximity (within 5 cm) to the stimulus mouse were recorded to assess sociability. A novel stimulus mouse was used for each test subject to avoid repeated exposure.

### Membrane Proteomics and Data Analysis.

Male littermate mice consisting of five *Ank3*^−/−^:*CaMKIIα*-Cre and five *Ank3*^fl/fl^ controls from two independent cohorts (14 wk old) were used for proteomic profiling. Cortical tissues were rapidly dissected, homogenized in ice-cold sucrose buffer (20 mM HEPES, pH 7.4; 320 mM sucrose; 5 mM EDTA; supplemented with Roche Complete protease inhibitor cocktail), and subjected to differential centrifugation. Homogenates were first centrifuged at 3,000 × g for 20 min at 4 °C to remove nuclei and debris. The resulting supernatant was centrifuged at 16,000 × g for 30 min at 4 °C to separate the cytosolic fraction (S2) and membrane-enriched pellet (P2). The P2 fraction was resuspended in solubilization buffer containing 50 mM Tris-HCl (pH 7.5), 1% Triton X-100, 150 mM NaCl, 1 mM EDTA, 1 mM AEBSF, and supplemented with protease and phosphatase inhibitors. Samples were incubated on ice for 1 h to facilitate protein extraction. Detailed protocols for sample preparation and TMT-based quantitative proteomics have been previously described (13). Protein quantification was performed using Census software, and normalized intensity values were used to compute relative protein abundance. For each protein, intensity values from *Ank3*^−/−^:*CaMKIIα*-Cre samples were standardized to the mean of the five *Ank3*^fl/fl^ controls. Fold changes were calculated as the mean of the standardized values for the knockout group. Statistical significance was determined using a one-tailed Student’s *t* test. Gene Ontology (GO) enrichment analysis was conducted using SynGO, a synapse-specific, evidence-based knowledgebase. Proteins showing statistically significant differential expression were submitted to SynGO to identify enriched synaptic cellular components and biological processes. When overlapping terms were enriched, the broader or functionally relevant annotations were prioritized and reported with their corresponding *P*-values.

### Gene Set Analysis.

Proteins showing altered expression in the proteomic analysis were analyzed for enrichment in biological processes and cellular components using the SynGO database. To examine disease-associated gene enrichment, a curated list of 1,230 autism spectrum disorder (ASD) risk genes from the SFARI database was used. Additionally, gene sets associated with Alzheimer’s disease (AD; n = 362), BD (n = 652), and SZ (n = 1,512) were obtained from the Human Gene Mutation Database (HGMD) Professional (v2025.02, Qiagen) and analyzed based on the identified gene groups. Statistical analysis for each overlap was assessed using a hypergeometric test.

### Primary Cortical Neuron Culture and Transfection.

Primary cortical neurons were isolated from postnatal day 0 C57BL/6J mouse pups (The Jackson Laboratory) as previously described. Brains were rapidly dissected in ice-cold Leibovitz’s L-15 medium supplemented with penicillin/streptomycin. Cortical tissue was isolated and enzymatically dissociated using 0.25% trypsin-EDTA at 37 °C for 15 min, followed by gentle mechanical trituration in high-glucose Dulbecco’s Modified Eagle Medium containing 10% fetal bovine serum, 1.4 mM L-glutamine, and 6.0 g/L glucose. Dissociated neurons were seeded at a density of 320,000 cells per 18 × 18 mm glass coverslip or 640,000 cells per well in six-well plates, both precoated with poly-D-lysine (50 µg/mL; Sigma-Aldrich) and laminin (2 µg/mL; Sigma-Aldrich). Cultures were maintained at 37 °C in a humidified incubator with 5% CO_2_ in neurobasal medium supplemented with B27 (2%), GlutaMAX-I (2 mM), and penicillin/streptomycin (100 U/mL and 100 µg/mL, respectively). At 14 d in vitro, neurons were transfected using Lipofectamine 2000 (Invitrogen) with a total of 4 μg plasmid DNA per coverslip. Lipofectamine 2000 was diluted in DMEM supplemented with 10 mM HEPES, gently mixed with plasmid DNA, and incubated at 37 °C for 20 to 30 min to allow complex formation. The transfection mixture was then added to the neuronal cultures. After 3 to 4 h of incubation, cells were returned to antibiotic-containing feeding media composed of 50% conditioned medium and 50% fresh Neurobasal medium. Transfected neurons were incubated for an additional 72 h prior to downstream assays.

### Calcium Imaging Analysis.

Four *Ank3*^−/−^;*GCaMP3*^+/−^;*CaMKIIα*-Cre mice and four *GCaMP3*^+/−^;*CaMKIIα*-Cre control mice (5 mo old) were deeply anesthetized with 3.5% isoflurane in 100% O_2_ and transcardially perfused with ice-cold N-methyl-D-glucamine-based artificial cerebrospinal fluid (NMDG-aCSF) to enhance neuronal viability during acute slicing of aged tissue. Brains were rapidly extracted and coronally sectioned into 400 μm-thick slices using a vibratome (VT1000S, Leica) in ice-cold, oxygenated NMDG-aCSF (substituting NaCl with NMDG to reduce excitotoxic damage). Slices were incubated in warmed NMDG-aCSF at 33 °C for 11 min, then transferred to standard sodium-based aCSF and allowed to recover for 1 h at room temperature before imaging. Calcium imaging was performed using a two-photon laser-scanning microscope (excitation at 950 nm) at 3 frames per second. Slices were continuously superfused with oxygenated aCSF (95% O_2_/5% CO_2_) at a flow rate of 3 mL/min, maintained at 33 °C. Neuronal activity was elicited by bath application of 5 mM KCl, inducing calcium transients comparable to physiological sensory stimulation in layer 2/3 of the primary somatosensory cortex (S1). Regions of interest (ROIs) corresponding to neuronal somata were identified using a custom ImageJ macro, detecting cellular contours ranging from 40 to 500 μm^2^ and exhibiting fluorescence intensity changes with a Z-score ≥ 2.5, indicative of calcium transients. Fluorescence time-series data were exported to MATLAB for further analysis. Signals were normalized to a local baseline and smoothed using a wavelet filter. Relative fluorescence changes were expressed as ΔF/F_0_. Calcium events were identified using MATLAB’s findpeaks function, with a threshold of ≥20% above baseline. Cells lacking identifiable peaks were excluded from subsequent analyses.

### Calcium Imaging of Transfected Cortical Neurons.

Primary cortical neurons cultured on 18 × 18 mm glass coverslips were cotransfected with either an mCherry-tagged scrambled shRNA control plasmid (GeneCopoeia; CSHCTR001-1-mU6) or shRNA targeting ankyrin-G (shAnkG; GeneCopoeia; MSH0338708-33-mU6; GGTGATAAATGCACATGGTTC for ankyrin-G) (14), along with a plasmid encoding the genetically encoded calcium indicator GCaMP6f (Addgene plasmid #40755). For live-cell imaging, coverslips were transferred to a circular imaging chamber (Warner Instruments; RC-41LP) and maintained within a controlled CO_2_ incubation system (TOKAI HIT; UNIV-D35) during data acquisition. Time-lapse imaging was performed using a Nikon C2 confocal microscope equipped with an EMCCD camera. Fluorescence from both mCherry and GCaMP6f was captured at a single focal plane (field of view: 42.43 × 42.43 μm) at a resolution of 128 × 128 pixels. Images were acquired at 2 Hz (one frame every 0.5 s) for a total duration of 10 min. Fluorescence intensity values were normalized to the average intensity of the three frames preceding each activation event. Calcium transients were defined as fluorescence increases ≥threefold above baseline (ΔF/F_0_), and these events were used for downstream quantitative analyses.

### Immunofluorescence Histochemistry.

Eighteen-week-old mice were euthanized via intraperitoneal injection of EUTHASOL (Virbac; 710101) at a dosage of 5 µL/g body weight according to the guidelines of the IACUC at Northwestern University, followed by transcardial perfusion with filter-sterilized phosphate-buffered saline (PBS), and subsequently with 4% paraformaldehyde (PFA) in PBS. All procedures were conducted in accordance with protocols approved by the IACUC at Northwestern University and complied with the guidelines of the U.S. NIH. Brains were extracted and postfixed in 4% PFA for 4 h at 4 °C, followed by cryoprotection in 30% sucrose in PBS for 48 h at 4 °C. Coronal brain sections (40 μm thickness) were prepared using a cryostat (Leica) and processed as free-floating sections for immunofluorescent labeling. Sections were washed three times in PBS (10 min each), then incubated for 30 min at room temperature in PBS containing 0.3% Triton X-100 and 1% bovine serum albumin (BSA) to permeabilize tissue and block nonspecific binding. Samples were then incubated overnight at 4 °C with a mouse monoclonal anti-ankyrin-G primary antibody (NeuroMab; 1:200 dilution) or rabbit anti-Mbp (Thermofisher, PA5-78397, 1:200). Following three PBS washes, sections were incubated for 1 h at room temperature with Alexa Fluor 568-conjugated donkey anti-mouse IgG secondary antibody or Alexa Fluor 488-conjugated donkey anti-rabbit IgG secondary antibody (Invitrogen; 1:1,000 dilution). All antibody dilutions were prepared in PBS supplemented with 1% BSA and 0.3% Triton X-100. After two PBS washes, a Hoechst solution (5 µg/mL) was applied for nucleic acid staining. Finally, the sections were mounted in Fluorescent Mounting Medium.

### Confocal and Super Resolution Microscope.

Mbp immunofluorescence was imaged using a Nikon C2 confocal microscope equipped with a 20× objective lens. All images were acquired using identical imaging parameters, including laser power, high-voltage (HV) gain, and offset settings. For each section, 7 to 8 optical slices were collected at 1-µm intervals and projected into a single maximum-intensity image using Fiji (ImageJ), yielding a final field of view of 640.57 × 640.57 µm. Regions of interest (131.99 × 120.73 µm) corresponding to individual layers of the primary somatosensory cortex were manually defined. Mbp density was quantified in Fiji by applying a consistent threshold to all images, followed by measurement of the threshold-positive area using the Measure function. To evaluate myelin morphology, images were acquired using a Nikon W1-SoRa superresolution microscope equipped with a 60× objective lens. Z-stack images consisting of 81 optical sections were collected at 100-nm intervals, covering a total imaging depth of 8 µm. Raw images were processed using Richardson-Lucy deconvolution and reconstructed into three-dimensional images with Imaris software.

### Lithium Treatment.

Mice were administered lithium carbonate via a specialized diet over a 3-wk period. During the first week, animals received chow containing 0.2% lithium carbonate (Envigo), which was followed by 0.4% lithium carbonate chow for the subsequent 2 wk. Throughout the treatment period, mice were also provided with ad libitum access to a saline solution to mitigate potential lithium-induced dehydration (9, 15).

### Fractionation Protocol.

Subcellular fractionation was conducted as previously described (14), with minor modifications. Cortical tissue from 16-wk-old mice was homogenized in ice-cold sucrose buffer (20 mM HEPES, pH 7.4; 320 mM sucrose; 5 mM EDTA) supplemented with a protease inhibitor cocktail (Roche). Homogenates were centrifuged at 3,000 × g for 20 min at 4 °C to remove nuclei and cellular debris (yielding the S1 fraction). The supernatant was subsequently centrifuged at 16,000 × g for 30 min at 4 °C to separate the cytosolic fraction (S2) and pellet membrane-associated components (P2). The P2 pellet was resuspended in Triton extraction buffer (20 mM Tris-HCl, pH 7.4; 2% Triton X-100; 150 mM NaCl; 1 mM EGTA) containing protease inhibitors and solubilized on ice for 1 h. The mixture was centrifuged at 16,000 × g for 10 min at 4 °C, and the Triton-insoluble pellet was further extracted with SDS buffer (20 mM Tris, pH 7.4; 150 mM NaCl; 1 mM EGTA; 1% SDS; with protease inhibitors) by incubation at 37 °C for 20 min. This extract was centrifuged at 38,000 × g for 10 min at 4 °C. The resulting supernatant was collected as the membrane fraction (P3). Samples were mixed with 4× Laemmli sample buffer and then denatured (5 min at 95 °C). The proteins were separated with a 4 to 20% SDS-PAGE and western blotting with mouse anti-ankyrin-G (Neuromab, N106/20, 1:1,000), rabbit anti-Mbp (Thermofisher, PA5-78397, 1:2,000), rabbit anti-Cap2 (Proteintech, 15865-1-AP, 1:1,000), rabbit anti-Ryr2 (Proteintech, 19765-1-AP, 1:1,000), mouse anti-Pfn2 (Santa Cruz, sc-100955, 1:500), mouse anti-Bin1 (Millipore, 05-449, 1:1,000), rabbit anti-Git2 (Thermo, PA5-118830, 1:1,000), rabbit anti-Wasf1 (Thermo, PA5-78273, 1:1,000), rabbit anti-Wfs1 (Thermo, PA5-102465, 1:1,000), rabbit anti-Cnksr2 (Thermo, PA5-101221, 1:1,000), rabbit anti-Taok2 (Thermo, PA5-29249, 1:1,000), mouse anti-N+/K+ ATPase α1 (Novos, NB300-146, 1:1,000), rabbit anti-GluA1 (Millipore, AB1504, 1:1,000), rabbit anti-PSD95 (Cell Signaling, #2507, 1:1,000), rabbit anti-β-tubulin (Cell Signaling, #5568, 1:1,000) antibodies.

### Statistical Analysis.

All statistical tests were performed with GraphPad Prism 10. A two-sample comparison was performed using unpaired Student’s *t* test, and multiple comparisons were made using two-way ANOVA followed by a Bonferroni test. Differential protein abundance was analyzed using unpaired Welch’s *t* tests followed by Benjamini–Hochberg false discovery rate correction (Q = 0.05). Bar graphs are displayed as mean ± SEM. Statistical significance was indicated as follows: *P < 0.05, **P < 0.01, ***P < 0.001.

## Results

### Both Developmental and Adult *Ank3* Deletions in the Forebrain Lead to Hyperlocomotion, Reduced Anxiety, and Reduced Depression-Like Behaviors.

Previous studies have demonstrated that deletion of neurodevelopmental or neuropsychiatric risk genes can alter adult behavioral phenotypes in mice through Cre recombinase models, where gene expression in the forebrain varies depending on the developmental stage (13, 16). Notably, age at onset and the presence of psychotic symptoms in BD are clinically relevant and heritable subphenotypes (17). We therefore hypothesized that forebrain-specific deletion of the *Ank3* gene at distinct developmental stages would differentially influence the emergence of behavioral deficits in adulthood.

Behavioral abnormalities in adult mice with *Ank3* deletion in forebrain excitatory neurons (*Ank3^–/^^–^*:*CaMKIIα*-Cre) have been previously reported (9). However, no studies have examined whether deletion of *Ank3* during early development results in similar or distinct behavioral outcomes in adulthood (*SI Appendix*, Fig. S1). To address this, we generated a cKO line with prenatal deletion of *Ank3* (*Ank3^–/^^–^*:*Emx1*-Cre), in which Cre recombinase is robustly expressed in the forebrain from embryonic day 10.5 (18). In both *Ank3^–/^^–^*:*CaMKIIα*-Cre and *Ank3^–/^^–^*:*Emx1*-Cre mice, western blot analysis of cortical homogenates revealed significantly reduced ankyrin-G protein levels following Cre expression (*SI Appendix*, Fig. S2*A*).

To assess behavioral phenotypes resulting from *Ank3* deletion in cortical excitatory neurons and oligodendrocytes (*Ank3^–/^^–^*:*Emx1*-Cre, prenatal) and compare them with those previously reported in adult-onset excitatory neuron-specific deletions (*Ank3^–/^^–^*:*CaMKIIα*-Cre), we conducted a battery of behavioral tests. Adult (3-mo-old) mice from both lines were evaluated using the open field test, elevated zero maze, light/dark box, FST, and social interaction test. These assays were used to assess motor activity, anxiety-related behavior, depressive-like symptoms, and sociability.

**Fig. 1.**
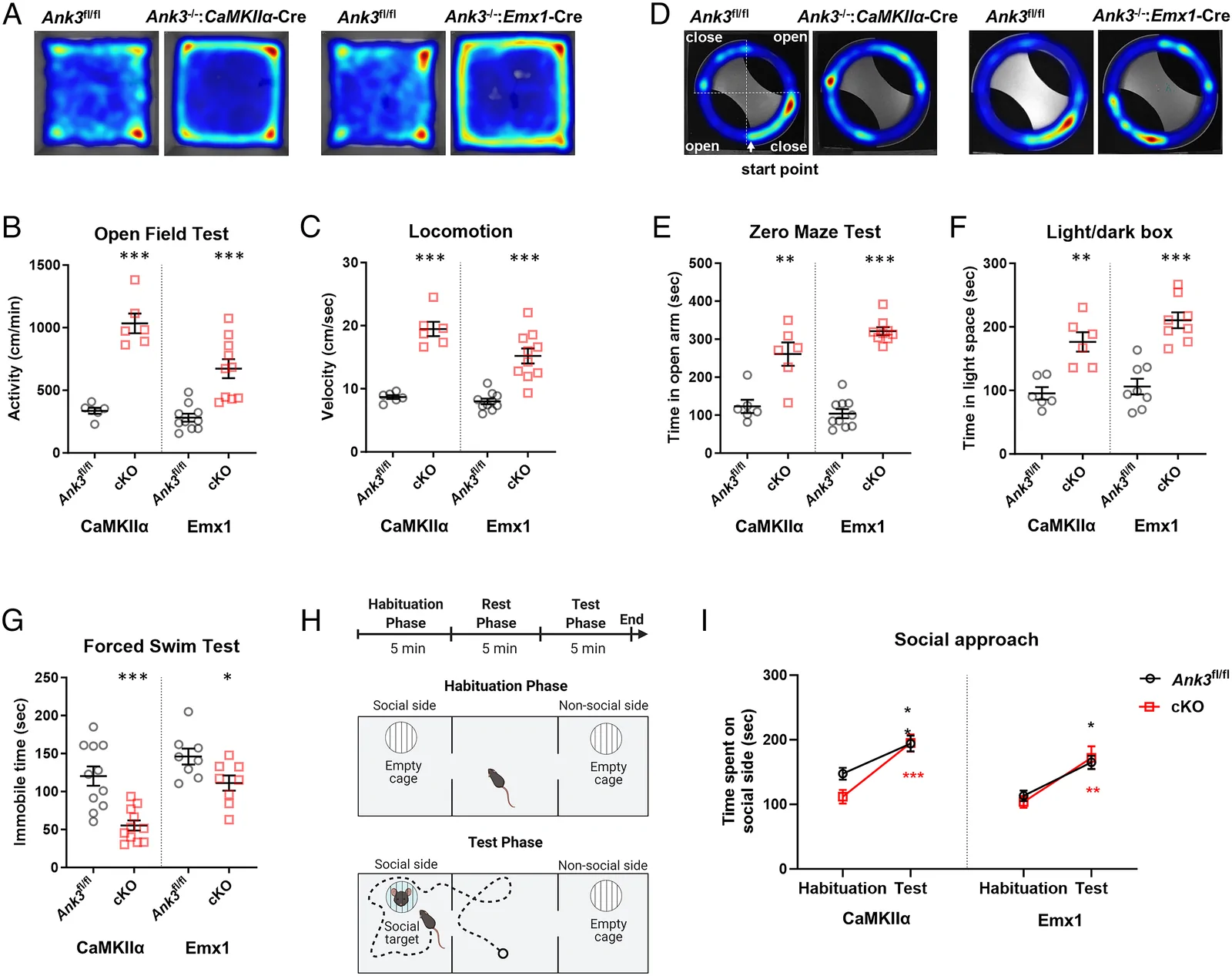
Deletion of ankyrin-G in the forebrain during development or adulthood leads to similar behavioral phenotypes. (*A*) Heatmaps showing locomotor activity during the open field test. (*B* and *C*) Quantification of distance traveled during a 30-min open field test. ***P < 0.001; *Ank3^–/^^–^*:*CaMKIIα-*Cre, *n* = 6 per *Ank3^fl/fl^* or cKO; *Ank3^–/^^–^*:*Emx1-*Cre, *n* = 10 per *Ank3^fl/fl^* or cKO. (*D*) Heatmaps showing activity during the elevated zero maze test. (*E*) Quantification of time spent in open arms over a 10-min trial. **P < 0.01, ***P < 0.001; *n* as in panel (*B*). (*F*) Time spent in the light chamber during the 5-min light/dark box test. **P < 0.01, ***P < 0.001; *Ank3^–/^^–^*:*CaMKIIα-*Cre, *n* = 6; *Ank3^–/^^–^*:*Emx1-*Cre, *n* = 8 per *Ank3^fl/fl^* or cKO. (*G*) Immobility time in the FST during the final 4 min. *P < 0.05, ***P < 0.001; *Ank3^–/^^–^*:*CaMKIIα-*Cre, *n* = 11; *Ank3^–/^^–^*:*Emx1-*Cre, *n* = 8 per *Ank3^fl/fl^* or cKO. (*H*) Schematic of the apparatus used for the three-chamber social interaction test. Created with BioRender.com. (*I*) Time spent in the social chamber during habituation and test phases. *P < 0.05, **P < 0.01, ***P < 0.001; *Ank3^–/^^–^*:*CaMKIIα-*Cre, *n* = 6; *Ank3^–/^^–^*:*Emx1-*Cre, *n* = 8 per *Ank3^fl/fl^* or cKO. Statistical analysis was performed using two-tailed unpaired *t* tests. All data are presented as mean ± SEM.

Consistent with prior findings, *Ank3^–/^^–^*:*CaMKIIα*-Cre mice displayed increased locomotor activity, reduced anxiety, and mania-like behavior (Fig. 1 *A*–*G* and *SI Appendix*, Fig. S2 *B* and *C*). Interestingly, *Ank3^–/^^–^*:*Emx1*-Cre mice exhibited similar behavioral abnormalities—hyperactivity and anxiolytic-like behavior—but showed a less pronounced mania-like phenotype in the FST. In contrast, both cKO models exhibited normal performance in the social interaction test (Fig. 1 *H* and *I* and *SI Appendix*, Fig. S2*D*).

These results suggest that postnatal deletion of *Ank3* in forebrain pyramidal neurons is sufficient to induce behavioral abnormalities such as hyperlocomotion, reduced anxiety, and mania-like traits—similar to those observed with early developmental deletion. This indicates that *Ank3*’s role in postnatal neuronal maintenance or plasticity may be more critical for adult behavioral regulation than its role during early embryonic development.

### *Ank3^–/^^–^*:*CaMKIIα*-Cre Mice Show Lower Neuronal Activity.

Reduced neuronal depolarization is known to influence cortical dynamics and contributes to phenomena such as spreading depression in neurological disorders (19). Deletion of the *Ank3* exon 1b isoform specifically in parvalbumin interneurons has previously been shown to elevate firing thresholds and reduce the dynamic range of action potentials, thereby disrupting the excitation/inhibition balance in neuronal circuits (11). In earlier work, we demonstrated that reduced *Ank3* expression not only decreases the number and size of postsynaptic spines but also diminishes neuronal activity in pyramidal neurons by impairing AMPA receptor trafficking and surface expression at the cell membrane (10).

**Fig. 2.**
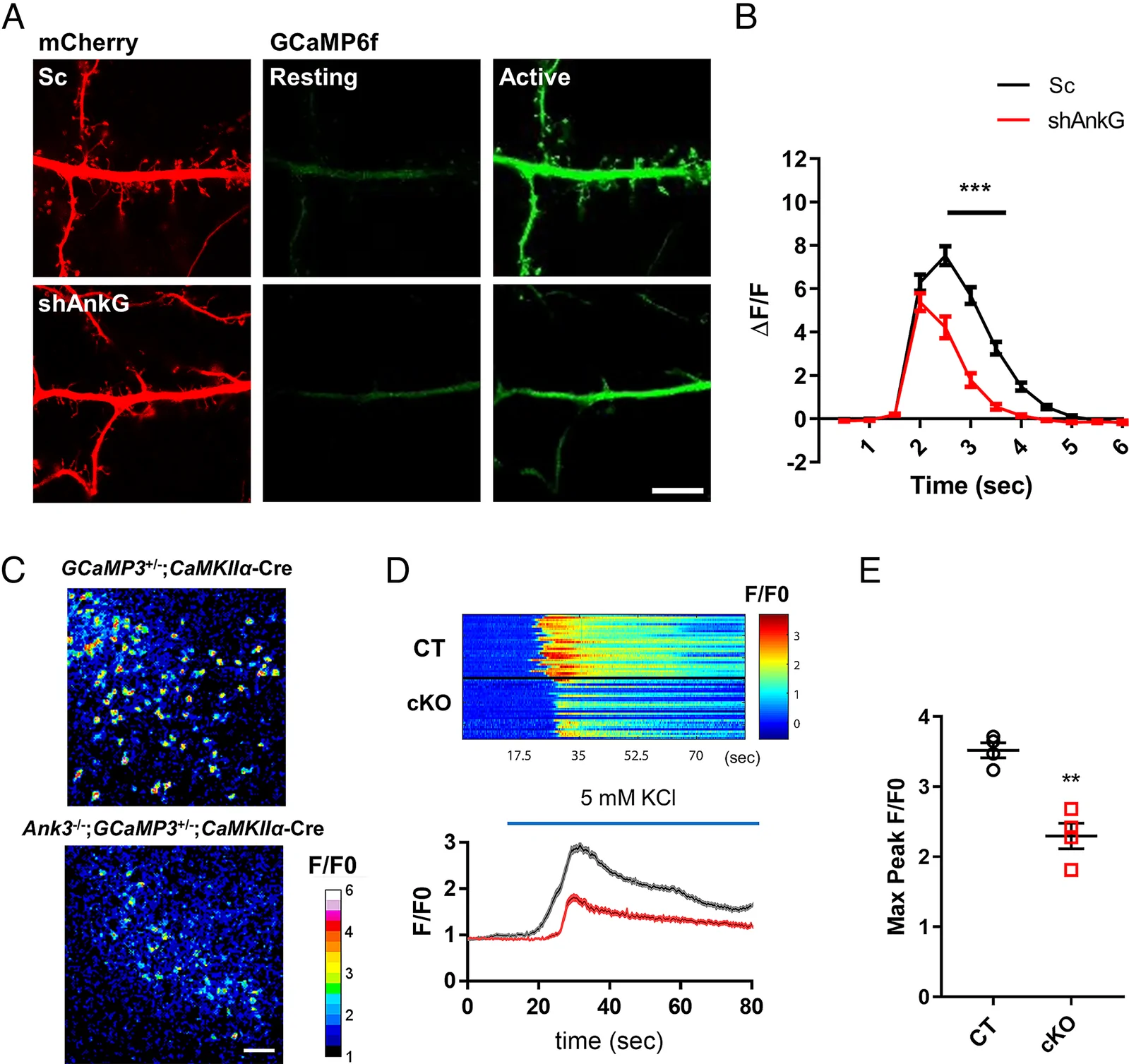
Knockdown or deletion of ankyrin-G alters cortical neuronal Ca^2+^ dynamics. (*A*) Representative confocal images of primary cultured cortical neurons transfected with GCaMP6f and either Sc or ankyrin-G knockdown (shAnkG) construct. (Scale bar, 10 μm.) (*B*) Normalized traces of spontaneous calcium transients showing average fluorescence peak amplitudes over a 10-min recording period. ***P < 0.001; *Sc*, *n* = 20 neurons; *shAnkG*, *n* = 10 neurons. Statistical analysis was performed using two-way ANOVA followed by the Bonferroni post hoc test. (*C*) Representative fluorescence images showing calcium response to high-potassium aCSF in acute cortical slices from *GCaMP3^+/^^–^*;*CaMKIIα-*Cre and *Ank3^–/^^–^*;*GCaMP3^+/^^–^*;*CaMKIIα-*Cre mice. (*D*) Heatmaps representing evoked calcium activity at the single-neuron level from one slice per genotype, with corresponding average time traces of KCl-evoked calcium transients. (*E*) Quantification of peak calcium response amplitudes per animal in *GCaMP3^+/^^–^*;*CaMKIIα-*Cre (n = 4) and *Ank3^–/^^–^*;*GCaMP3^+/^^–^*;*CaMKIIα-*Cre (n = 4) mice. **P < 0.01; two-tailed unpaired *t* test. All data are presented as mean ± SEM.

To investigate neuronal activity more broadly, we employed calcium imaging, an effective method for visualizing activity across large populations of neurons. To assess how *Ank3* knockdown (*SI Appendix*, Fig. S3) affects spontaneous neuronal firing, we expressed GCaMP6f, a genetically encoded calcium indicator, along with the fluorescent marker mCherry, in primary cultured cortical neurons. These cells were cotransfected with either scrambled control (Sc) or shAnkG (short hairpin RNA targeting ankyrin-G). As expected, shAnkG expression significantly reduced spontaneous cytosolic Ca^2+^ influx, indicating decreased basal neuronal activity (Fig. 2 *A* and *B*).

We next evaluated evoked neuronal activity in acute cortical slices by measuring KCl-induced depolarization responses. Calcium imaging was performed on 5-mo-old *Ank3^–/^^–^*;*GCaMP3*^+/^*^–^*;*CaMKIIα*-Cre mice and their *GCaMP3*^+/^*^–^*;*CaMKIIα*-Cre littermate controls. To confirm coexpression of GCaMP3 expression and ankyrin-G, we performed ankyrin-G immunostaining in the cortex and hippocampus of *GCaMP3*^+/^*^–^*;*CaMKIIα*-Cre mice (*SI Appendix*, Fig. S4). As anticipated, this staining pattern matched previous observations in *Ank3^–/^^–^*:*CaMKIIα*-Cre mice, showing ankyrin-G expressed in CaMKIIα-positive excitatory neurons (9, 14).

Imaging focused on layer 2/3 of the S1 cortex under a multiphoton laser-scanning microscope in low-magnesium aCSF (Fig. 2*C*). Following KCl stimulation, *Ank3^–/^^–^*;*GCaMP3*^+/^*^–^*;*CaMKIIα*-Cre mice exhibited a significant reduction in calcium event amplitude compared to controls (Fig. 2 *D* and *E*).

Although our findings may appear inconsistent with the increased cortical c-Fos expression previously reported in *Ank3^–/^^–^*:*CaMKIIα*-Cre mice (9), the two studies assessed distinct aspects of neuronal activity. Differences in the temporal scale of measurement, brain regions analyzed, cellular populations examined, and behavioral context may contribute to the distinct outcomes observed. Therefore, these findings should be interpreted as complementary rather than mutually exclusive.

Taken together, these findings demonstrate that layer 2/3 neurons in the S1 cortex of *Ank3^–/^^–^*;*GCaMP3*^+/^*^–^*;*CaMKIIα*-Cre mice are less excitable and exhibit blunted depolarization responses to KCl. This decrease in neuronal activity may underlie, or at least contribute to, the abnormal behavioral phenotypes observed in these mice.

### Absence of Ankyrin-G Alters the Membrane Proteome.

Alterations in protein expression represent a fundamental mechanism by which the brain adapts to genetic mutations that affect neuronal development and connectivity. The brain exhibits a high degree of plasticity, enabling it to respond to genetic perturbations through dynamic changes in its proteome. We therefore hypothesized that *Ank3* deletion would result in proteomic changes that may contribute to the altered neuronal activity and behavioral phenotypes observed in *Ank3*-deficient mice.

**Fig. 3.**
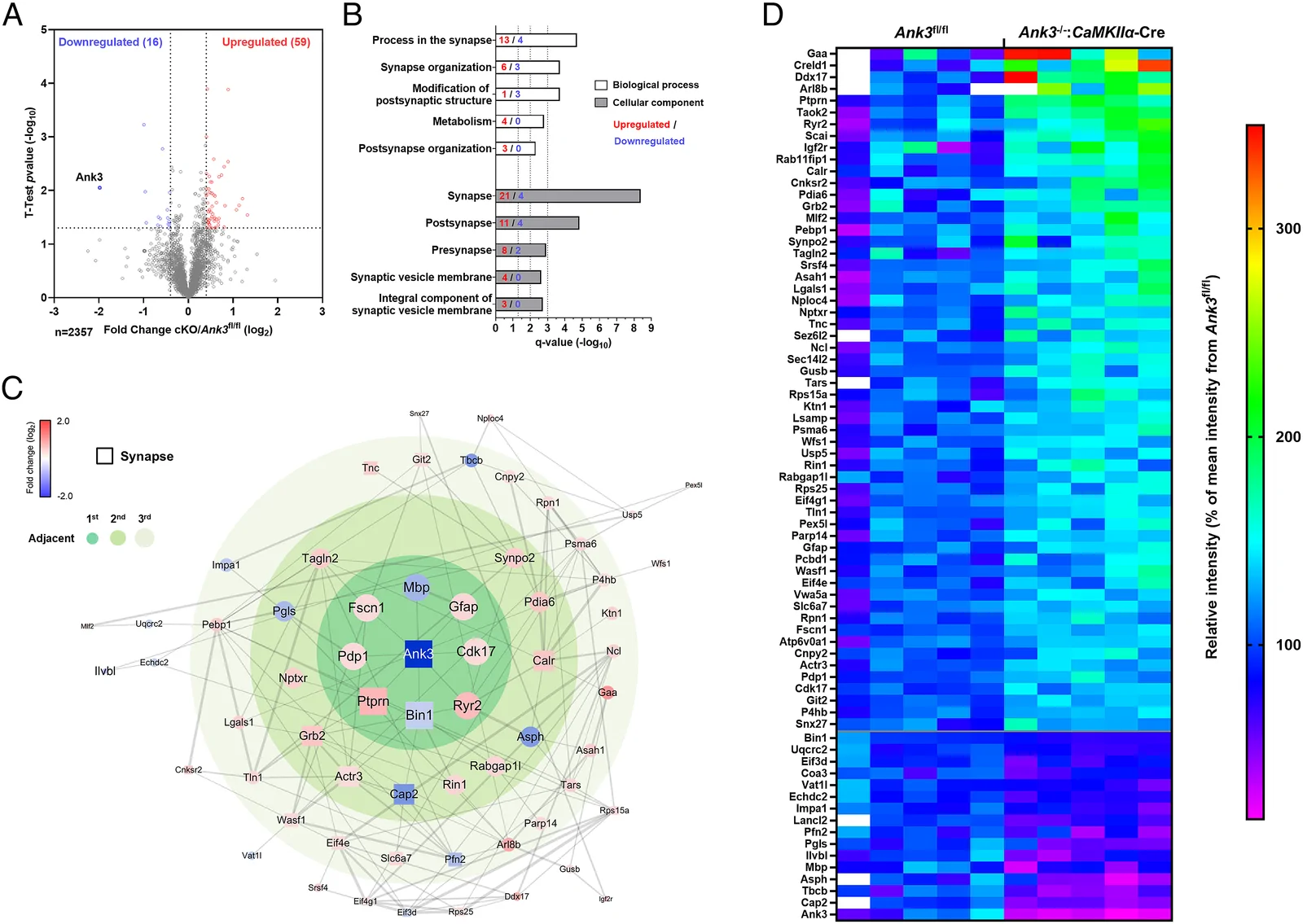
The cortical membrane proteome is remodeled in *Ank3^–/^^–^:CaMKIIα-Cre* mice. (*A*) Volcano plot showing the fold change (log_2_) vs. statistical significance (−log_10_ *P*-value) of protein expression levels in cortical membrane-enriched fractions from *Ank3^–/^^–^*:*CaMKIIα-*Cre vs. *Ank3^fl/fl^* mice. Significantly upregulated proteins (*P* < 0.05) are shown in red; significantly downregulated proteins (*P* < 0.05) in blue; nonsignificant proteins in gray. (*B*) GO enrichment analysis of significantly altered proteins with synaptic localization. Categories reflect biological process and cellular component annotations based on the SynGO database. (*C*) Protein–protein interaction network of significantly up- and downregulated proteins centered on ankyrin-G, generated using the STRING database and visualized in Cytoscape. The network is organized by interaction confidence levels defined by STRING score thresholds: first layer (>0.05), second layer (>0.00055), and third layer (>0.000094). (*D*) Heatmap showing the expression levels of selected significantly upregulated or downregulated proteins across samples.

To identify proteins that are upregulated or downregulated in response to *Ank3* deletion, we performed TMT labeling combined with LC/MS on crude membrane fractions isolated from the adult forebrain of *Ank3^–/^^–^*:*CaMKIIα*-Cre and *Ank3*^fl/fl^ mice. A total of 2,357 proteins were reliably quantified across both groups. Extensive proteomic remodeling was observed, with 75 proteins showing significant differential expression (*SI Appendix*, Table S1). Notably, the number of upregulated proteins (n = 59, red, *P* < 0.05) was more than threefold greater than the number of downregulated proteins (n = 16, light blue, *P* < 0.05; ≤0.5-fold change) (Fig. 3*A*).

Because in adult mice ankyrin-G is abundant in synaptic spines, to determine the functional relevance of these differentially expressed proteins, GO analysis was performed using the SynGO database (20). This analysis revealed that 25 of the 75 proteins were synaptically localized, with 15 localized to postsynaptic regions, 10 to presynaptic regions, and 4 proteins (Lsamp, Rps15a, Bin1, and Pfn2) found in both compartments (Fig. 3*B* and *SI Appendix*, Fig. S5*A*). Proteins related to synaptic vesicle membranes and metabolic processes—such as Atp6v0a1, Eif4e, Ptprn, Rps15a, Slc6a7, Usp5, and Wfs1—were predominantly upregulated. In contrast, proteins involved in structural remodeling of the postsynaptic compartment, such as Cap2 and Pfn2, were primarily downregulated.

To explore the broader interaction landscape of these altered proteins within ankyrin-G-related functional networks, a protein–protein interaction map was generated using the STRING database. This map included both predicted and experimentally validated interactions, stratified into three confidence layers: first layer (>0.05), second layer (>0.00055), and third layer (>0.000094) (Fig. 3*C* and *SI Appendix*, Fig. S5*B*).

Interestingly, proteins not traditionally associated with synaptic function were disproportionately enriched in the highest-confidence layer. In this top layer, several risk genes for neuropsychiatric disorders were differentially expressed: Ryr2 (SZ) and Gfap (autism spectrum disorder, ASD) were upregulated, whereas Mbp (SZ) and Bin1 (Alzheimer’s disease, AD) were downregulated. In the second layer, SZ risk genes Actr3 and Calr, and the BD risk gene Nptxr, were upregulated, while Cap2 (BD risk gene) was downregulated. The third layer showed upregulation of Asah1 (SZ), Tnc (BD), and Wasf1 (ASD).

**Fig. 4.**
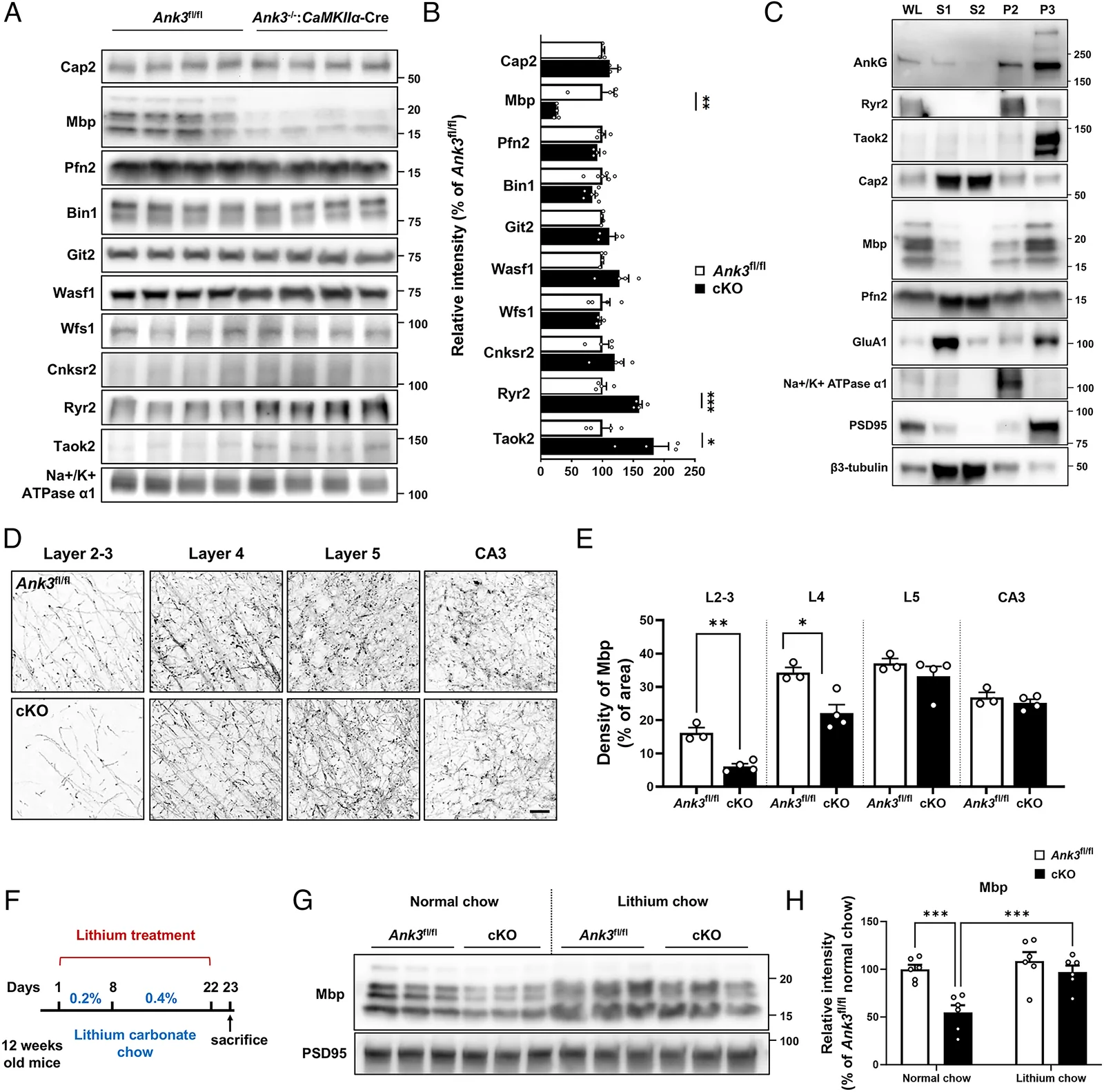
Reduced Mbp levels in the forebrain of *Ank3^–/^^–^*:*CaMKIIα-*Cre adult mice are restored by chronic lithium treatment. (*A*) Representative western blots of the P2 membrane-enriched fraction from the cortex of *Ank3^fl/fl^* and *Ank3^–/^^–^*:*CaMKIIα-*Cre mice. (*B*) Quantification of relative protein abundance in 12-wk-old mouse cortex (*n* = 4-6 per group). *P < 0.05; **P < 0.01; ***P < 0.001; two-tailed unpaired *t* test. (*C*) Western blot of subcellular fractions from the cortex of 16-wk-old mice, showing distribution of selected proteins across compartments. (*D*) Representative images of Mbp immunohistochemical staining in layers 2-3, 4, and 5 of the primary somatosensory cortex and the hippocampal CA3 region from 18-wk-old *Ank3^fl/fl^* and *Ank3^–/^^–^*:*CaMKIIα-*Cre mice. Images were acquired using a Nikon C2 confocal microscope and are shown as maximum-intensity projections. (Scale bar, 20 μm.) (*E*) Quantification of Mbp-positive area (%) in each brain region shown in (*D*). Data are presented as mean ± SEM (*Ank3^fl/fl^*, *n* = 3; *Ank3* cKO, *n* = 4). *P < 0.05, **P < 0.01; two-tailed unpaired Student’s *t* test. (*F*) Schematic of the chronic lithium treatment protocol. (*G*) Representative western blots of the P3 fraction, a postsynaptic density-enriched membrane fraction, from lithium-treated and untreated mice. (*H*) Quantification of Mbp levels in the P3 fraction from 15-wk-old *Ank3^fl/fl^* and *Ank3^–/^^–^*:*CaMKIIα-*Cre mice (*n* = 6 per group). ***P < 0.001; two-way ANOVA followed by the post hoc test. All data are presented as mean ± SEM.

To validate these findings, we conducted western blot analysis on the P2 membrane fraction from cortical tissues of *Ank3^–/^^–^*:*CaMKIIα*-Cre and *Ank3*^fl/fl^ mice. Consistent with the TMT-LC/MS results (Fig. 3*D*), we confirmed significant upregulation of Ryr2 and Taok2, as well as downregulation of Mbp (Fig. 4 *A* and *B*). However, despite showing reduced expression in the proteomic analysis, Cap2 did not exhibit significant changes by western blot. To investigate this discrepancy, we examined the subcellular distribution of Ryr2, Taok2, Cap2, Mbp, and Pfn2. Fractionation of forebrain tissue revealed that Cap2 and Pfn2 were enriched in the cytosolic fraction, while Taok2 was specifically localized to synaptic compartments. Mbp and Ryr2 were detected in both synaptic and membrane fractions (Fig. 4*C*), suggesting that some differences between LC/MS and western blot data may result from differences in subcellular localization. Functionally, Taok2 is involved in regulating actin cytoskeleton dynamics and synaptic protein positioning. Its activity influences dendritic spine morphology, synaptic strength, and postsynaptic protein clustering. Deletion or suppression of Taok2 has been shown to impair excitatory synaptic transmission (21). Ryr2 plays a pivotal role in calcium-induced calcium release, contributing to spine remodeling, synaptic plasticity, and the propagation of Ca^2+^ waves in the hippocampus (22, 23). Given these roles, the upregulation of Taok2 and Ryr2 in *Ank3^–/^^–^*:*CaMKIIα*-Cre mice likely reflects compensatory mechanisms triggered by loss of ankyrin-G. In contrast, Mbp is a structural component of the myelin sheath formed by oligodendrocytes and is essential for axonal insulation and rapid nerve impulse conduction. Its downregulation may have profound consequences, as myelin abnormalities have been strongly linked to disrupted neuronal connectivity and the pathophysiology of several neurodevelopmental and neuropsychiatric disorders.

To further investigate whether the reduction in Mbp protein levels was associated with regional alterations in myelin-related structures, we performed Mbp immunohistochemistry in the primary somatosensory cortex and hippocampal CA3 region of *Ank3^–/^^–^*:*CaMKIIα*-Cre mice. Quantitative analysis revealed significantly reduced Mbp density in layers 2/3 and 4 of the primary somatosensory cortex, whereas no significant differences were detected in layer 5 or the CA3 region (Fig. 4 *D* and *E*). To assess myelin morphology, we next performed super resolution imaging of Mbp-positive structures in layer 3 of the primary somatosensory cortex (*SI Appendix*, Fig. S6 *A* and *B*). Although myelin sheath diameter showed a trend toward an increase in *Ank3^–/^^–^*:*CaMKIIα*-Cre mice, this difference did not reach statistical significance (*SI Appendix*, Fig. S6 *C* and *D*). Together, these findings indicate that neuronal *Ank3* deficiency is associated with region-specific alterations in Mbp expression patterns without detectable changes in myelin sheath diameter.

### Chronic Lithium Treatment Restores Reduced Levels of Myelin Basic Protein.

A previous study using the *Ank3****^–/^^–^***:*CaMKIIα*-Cre mouse model-identical to the model employed in the present work demonstrated that lithium administration ameliorates BD-like behaviors (9). Beyond its psychiatric applications, lithium has also been implicated in white matter (WM) repair through the process of remyelination, which is associated with increased expression of Mbp (24–26). To investigate whether lithium affects Mbp expression in our model, we examined forebrain tissue from *Ank3****^–/^^–^***:*CaMKIIα*-Cre mice following a 3-wk lithium-enriched diet (Fig. 4*F*), using the same methods reported previously (9, 15). Contrary to our initial hypothesis, lithium treatment did not significantly alter Mbp expression in the P2 membrane fraction (*SI Appendix*, Fig. S7*A*). However, analysis of the P3 fraction from the same animals revealed a significant lithium-induced restoration of Mbp expression in *Ank3****^–/^^–^***:*CaMKIIα*-Cre mice (Fig. 4 *G* and *H*). To determine whether the effects of lithium extended to other proteins dysregulated in *Ank3****^–/^^–^***:*CaMKIIα*-Cre mice, we examined the expression of Ryr2 and Taok2. Ryr2 expression was elevated in *Ank3****^–/^^–^***:*CaMKIIα*-Cre mice relative to control littermates and remained elevated following lithium treatment (*SI Appendix*, Fig. S7 *C* and *D*). Similarly, lithium treatment did not significantly alter Taok2 expression in *Ank3****^–/^^–^***:*CaMKIIα*-Cre mice between lithium-treated and untreated groups (*SI Appendix*, Fig. S7 *E* and *F*). These results demonstrate that lithium treatment normalizes the previously downregulated Mbp levels, but in a compartment-specific manner within the P3 fraction, rather than the P2 membrane fraction. This compartmental specificity may reflect differences in the subcellular localization of Mbp and its functional role in myelin integrity. Taken together, these findings suggest that the behavioral improvements observed with chronic lithium administration may be mediated, at least in part, by its ability to restore myelination. This supports a broader role for lithium in modulating structural plasticity and white matter health in the context of *Ank3* deficiency.

## Discussion

Pathogenic variants in *ANK3* have been linked to neurodevelopmental conditions, including autism and intellectual disability (27–29), as well as neuropsychiatric disorders such as BD and SZ (30–32). Developmentally targeted approaches are critical to account for the dynamic expression patterns of ankyrin-G isoforms throughout brain maturation (5).

Prior studies have shown that the timing of gene deletion or restoration influences behavioral phenotypes. For example, deletion of *Shank3* during development results in synaptic deficits and autistic-like behaviors, including social impairment and repetitive grooming. Adult restoration partially rescues these phenotypes, improving social interaction and repetitive behaviors but not anxiety or motor coordination (33). Similarly, in a Rett syndrome model, neurons lacking *MeCP2* develop abnormally but do not degenerate, and adult reactivation of *MeCP2* reverses neurological and behavioral deficits (34). Developmental deletion of *BDNF* causes hyperactivity and hippocampal learning impairment, whereas adult deletion mainly affects learning (35). Overexpression of constitutively active GSK-3β induces hyperactivity and behavioral disinhibition postnatally, resembling mania, suggesting that dysregulation in this period disrupts behavior without early developmental abnormalities (36).

Surprisingly, our study shows that both prenatal and adult *Ank3* deletion in the forebrain result in similar behavioral phenotypes—hyperactivity and reduced anxiety—without affecting social interaction. These findings suggest that adult *Ank3* expression is necessary for behavioral regulation, and that distinct brain regions may contribute to neurodevelopmental aspects of disorders such as ASD.

A key finding of our study is that *Ank3* deletion in excitatory neurons significantly reduces Mbp levels. However, it remains unclear how loss of *Ank3*, which is expressed in adult forebrain excitatory neurons, leads to reduced expression of Mbp, a protein predominantly expressed in oligodendrocytes. Mbp encodes a major component of the myelin sheath that insulates axons and shows marked postnatal upregulation during active myelination in the mouse brain (37). In the cortex, Mbp expression begins in layer 6 at P5-P8 and spreads to all layers by P45-P60. In the corpus callosum, Mbp-positive oligodendrocytes are observed from P5 and extend broadly by P12, continuing to mature through P60. While direct mutations in Mbp are not strongly associated with disease, altered Mbp expression has been linked to BD, SZ, and ASD (38–40). The observed reduction in Mbp density suggests that *Ank3* deficiency may influence myelin-associated pathways in a region-specific manner. Interestingly, although ankyrin-G is broadly expressed across cortical layers, Mbp alterations were restricted to layers 2/3 and 4, suggesting a selective vulnerability whose underlying mechanisms remain unclear. However, because no significant changes in myelin sheath diameter were detected and the cellular source of the Mbp signal could not be determined, these findings should be interpreted as evidence of altered Mbp expression rather than definitive structural myelin abnormalities. Future studies using cell type–specific approaches will be required to determine the cellular source, layer specificity, and functional significance of the observed Mbp alterations.

The formation and maintenance of myelin rely on dynamic, bidirectional signaling between oligodendrocytes and axons (41, 42). Suppressing neuronal activity disrupts vesicular neurotransmitter release, impairing axo-glial signaling required for myelin initiation and stability, and resulting in reduced Mbp synthesis and myelin retraction (43). L1CAM and NrCAM, both L1 family cell adhesion molecules, modulate myelination by guiding OPC development and axon–glial interactions (44, 45). Ankyrin-G directly binds L1CAM and NrCAM through the conserved FIGQY motif, stabilizing them at the AIS and nodes of Ranvier (4, 5). This suggests a role for ankyrin-G in coordinating axonal architecture and myelin formation via axo-glial recognition.

Ankyrin-G interacts with ion channels, membrane transporters, adhesion molecules, and cytoskeletal components through its ankyrin-repeat domain (5). Loss of ankyrin-G disrupts these interactions and impairs neuronal function. In mice lacking the 480-kDa ankyrin-G isoform via exon 37 deletion, AIS formation is lost, and action potential kinetics are altered—specifically, slower upstroke and repolarization phases and reduced firing frequency (46). The 190-kDa isoform serves a postsynaptic scaffolding role, stabilizing AMPARs and supporting synaptic strength and plasticity. Its knockdown reduces AMPAR-mediated mEPSCs and GluA1 clustering and mobility (10). Consistent with these findings, we observed reduced spontaneous neuronal activity following ankyrin-G knockdown (GCaMP6f in vitro), and diminished KCl-induced depolarization in vivo (GCaMP3 imaging in layer 2/3 cortex). These results suggest that ankyrin-G deletion at excitatory synapses impairs neuronal excitability, potentially disrupting myelination.

Lithium, a widely used mood stabilizer for BD since its 1970 FDA approval, reduces suicide risk and treats manic and depressive episodes (47). WM abnormalities are common in psychiatric disorders, though findings on regional and directional change vary (24–26). Lithium has been shown to promote WM repair by enhancing remyelination under pathological conditions (48–50), often accompanied by increased Mbp expression (50, 51). In *Ank3^–/^^–^*:*CaMKIIα*-Cre mice, lithium reverses stress-induced mania-like behaviors (9). An additional finding of our study is that Mbp expression was restored by lithium treatment, whereas Ryr2 remained elevated and Taok2 expression was unaffected. These observations suggest that the molecular effects of lithium are selective rather than global. Although the mechanisms underlying this selectivity remain unclear, the differential responsiveness of these proteins indicates that not all molecular alterations associated with *Ank3* deficiency are equally sensitive to lithium treatment.

## Supplementary Material

Appendix 01 (PDF)

Dataset S01 (PDF)

## Data Availability

Study data are included in the article and/or supporting information.
