# Supplementary material for: Ank3 loss in adult forebrain excitatory neurons disrupts behavior, neuronal activity, membrane proteome, and myelination
Source: Proc Natl Acad Sci U S A. 2026 Aug 25;123(35):e2606900123. doi: 10.1073/pnas.2606900123 (PMC13535106; doi:10.1073/pnas.2606900123)
Supplement: Supplementary file 1 [file pnas.2606900123.sapp.pdf]

PNAS

Supporting Information for

Ank3 loss in adult forebrain excitatory neurons disrupts behavior, neuronal activity, membrane proteome, and myelination

Corresponding author: Peter Penzes
Email: p-penzes@northwestern.edu

This PDF file includes:

Figures S1 to S7
SI References

Other supporting material for this manuscript includes the following:

Dataset S1

Figures

Phenotypes	*Ank3* KO mice[1]	*Ank3* HT mice and shAnk3 in DG[2]		*Ank3* HT mice[3]	*Ank3* HT mice[4]	*Ank3* cHT mice[5]	*Ank3* cKO mice[6]	*Ank3* cKO mice[7]	*Ank3* cKO mice (In this paper)
Deletion target organ/cell types	Cerebellum-specific deletion	shAnk3 (exon 28) Dentate gyrus	Cerebellum-specific heterozygous	Cerebellum-specific heterozygous	Whole body	Neuronal and glial precursors (Nestin-Cre)	Pyramidal cell in the forebrain (CamkIIα-Cre)	Pyramidal cell in the forebrain (CamkIIα-Cre)	Forebrain-specific early deletion (Emx1-Cre)
Deleted isoforms	190, 270, 480 kDa	Reducing 190, 270,480 kDa	190, 270, 480 kDa	190, 270, 480 kDa	190, 270, 480 kDa	480 kDa	190, 270, 480 kDa	190, 270, 480 kDa	190, 270, 480 kDa
Survival	Premature death (4-6 months)	Normal	Normal	N/A	Normal	Normal	Normal	Normal	Normal
Locomotion	↓	Normal	Normal	N/A	Normal	Normal	↑ (Lithium rescue) (VPA rescue)	↑ (FSK no effect)	↑
Motor coordination	↓ (Ataxia from P16)	Normal	Normal	N/A	Normal	↓	Normal	Normal	Normal
Social interaction	N/A	N/A	N/A	N/A	N/A	Normal	N/A	Normal	Normal
Marble burying assay	N/A	N/A	N/A	N/A	N/A	Normal	N/A	N/A	N/A
Sucrose preference	N/A	N/A	↑ (Chronic stress Normal)	N/A	↓	N/A	N/A	N/A	N/A
Fear conditioning	N/A	Normal	Normal	N/A	N/A	N/A	N/A	N/A	N/A
Prepulse inhibition	N/A	Normal	Normal	N/A	N/A	Normal	Normal	N/A	N/A
Water maze	N/A	Normal	N/A	N/A	↓	Normal	N/A	N/A	N/A
Light/dark box	N/A	↓ (Lithium rescue)	↓ (Chronic stress ↑)	N/A	↑	N/A	↓	↓ (FSK rescue)	↓
Elevated plus maze	N/A	↓ (Lithium rescue)	↓ (Chronic stress ↑)	↓ (Lithium rescue)	↑	↑	↓ (VPA rescue) (Stress effect: dramatic fold change)	↓ (FSK rescue)	↓
Forced swim	N/A	Normal	Normal (Chronic stress ↑ immobility)	N/A	Immobility ↑	N/A	Less immobility (Stress effect: dramatic fold change)	Less immobility (FSK no effect)	Less immobility

Fig. S1. Behavioral phenotypes based on different *Ank3* deletion mice models. cKO: conditional knockout.

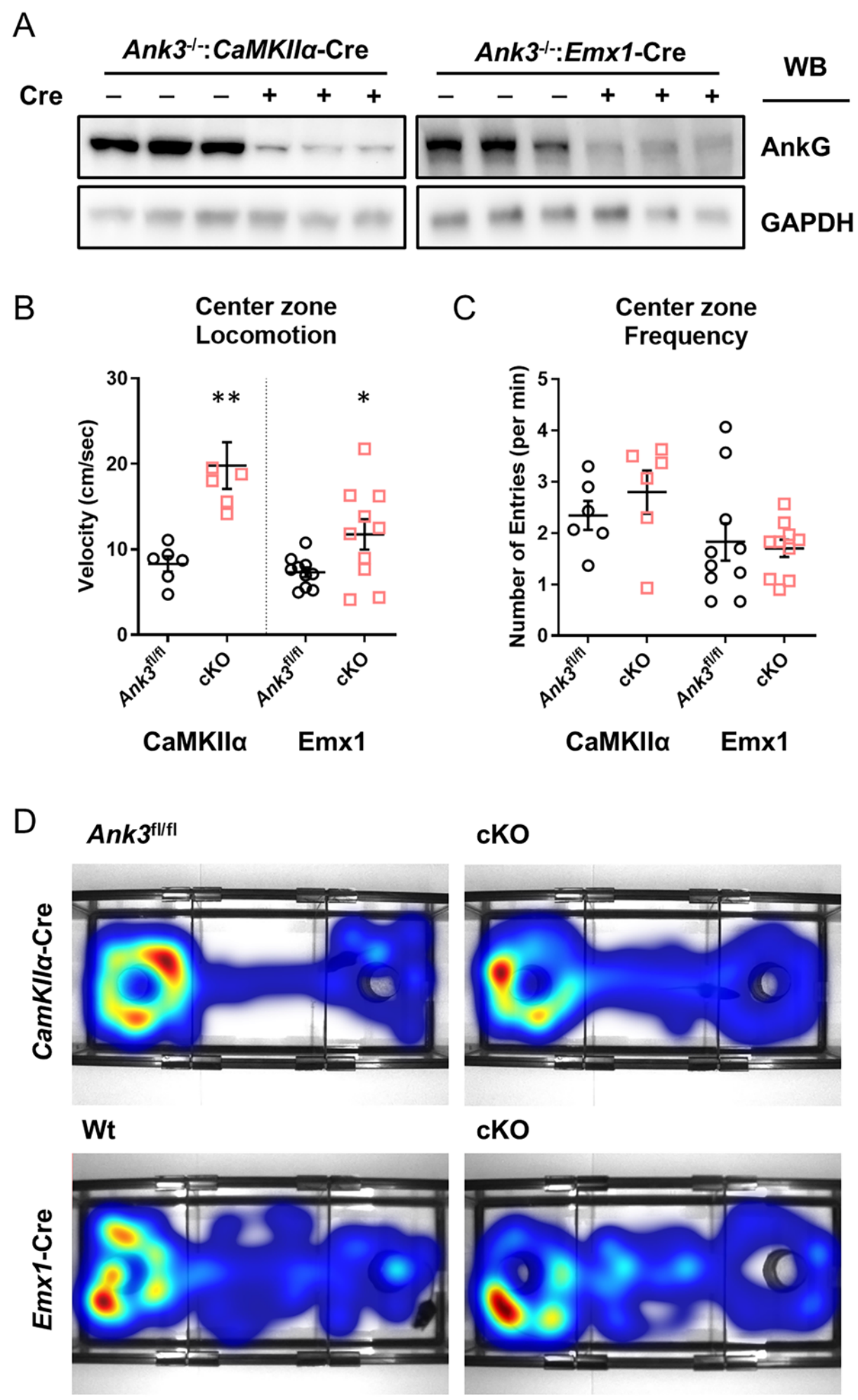

Fig. S2, related to Fig. 1. A Representative Western blots from the mouse cortex of different genotypes (n = 3 for each group). **B** Average locomotor velocity (cm/s) within the center zone (28 × 28 cm). **C** Frequency of center-zone entries (entries/min). Data are presented as mean ± SEM. $*p < 0.05$, $**p < 0.01$; two-tailed unpaired t-test. **D** Heatmaps of activity during social behavior.

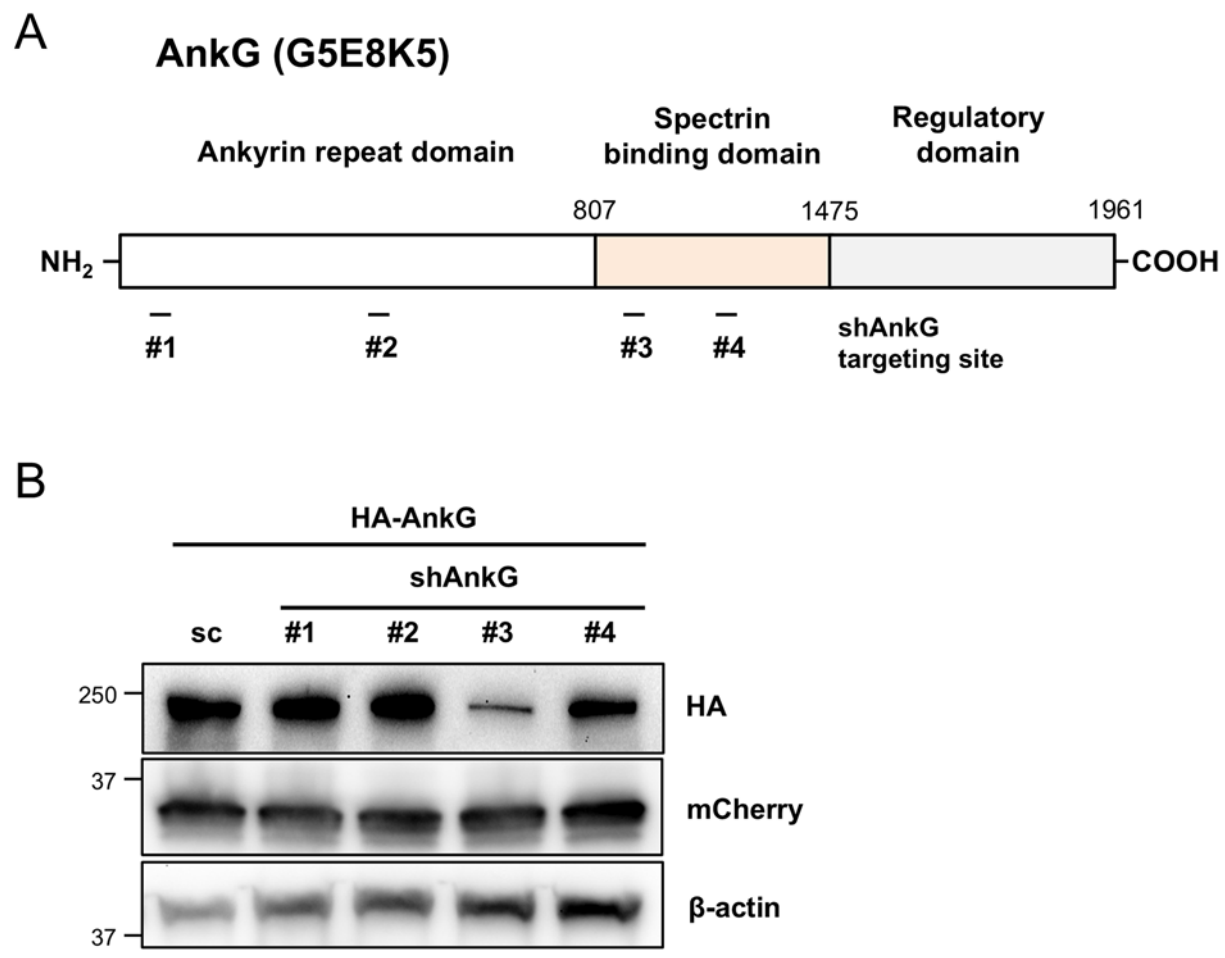

Fig. S3, related to Fig. 2. Validation of shAnkG efficiency. A Schematic illustration of the ankyrin-G (AnkG) protein and the target sites of four candidate shRNA constructs (#1-#4). The positions of the ankyrin repeat domain, spectrin-binding domain, regulatory domain, and the common shAnkG targeting region are indicated. **B** Validation of shAnkG-mediated knockdown in HEK293 cells. HEK293 cells were co-transfected with HA-tagged AnkG and either scrambled shRNA or four candidate shAnkG constructs (#1-#4). Cell lysates were analyzed by SDS-PAGE and Western blotting using antibodies against HA and mCherry, with β-actin serving as a loading control. Among the four candidates, shAnkG construct #3 exhibited the greatest knockdown efficiency and was therefore used in subsequent experiments.

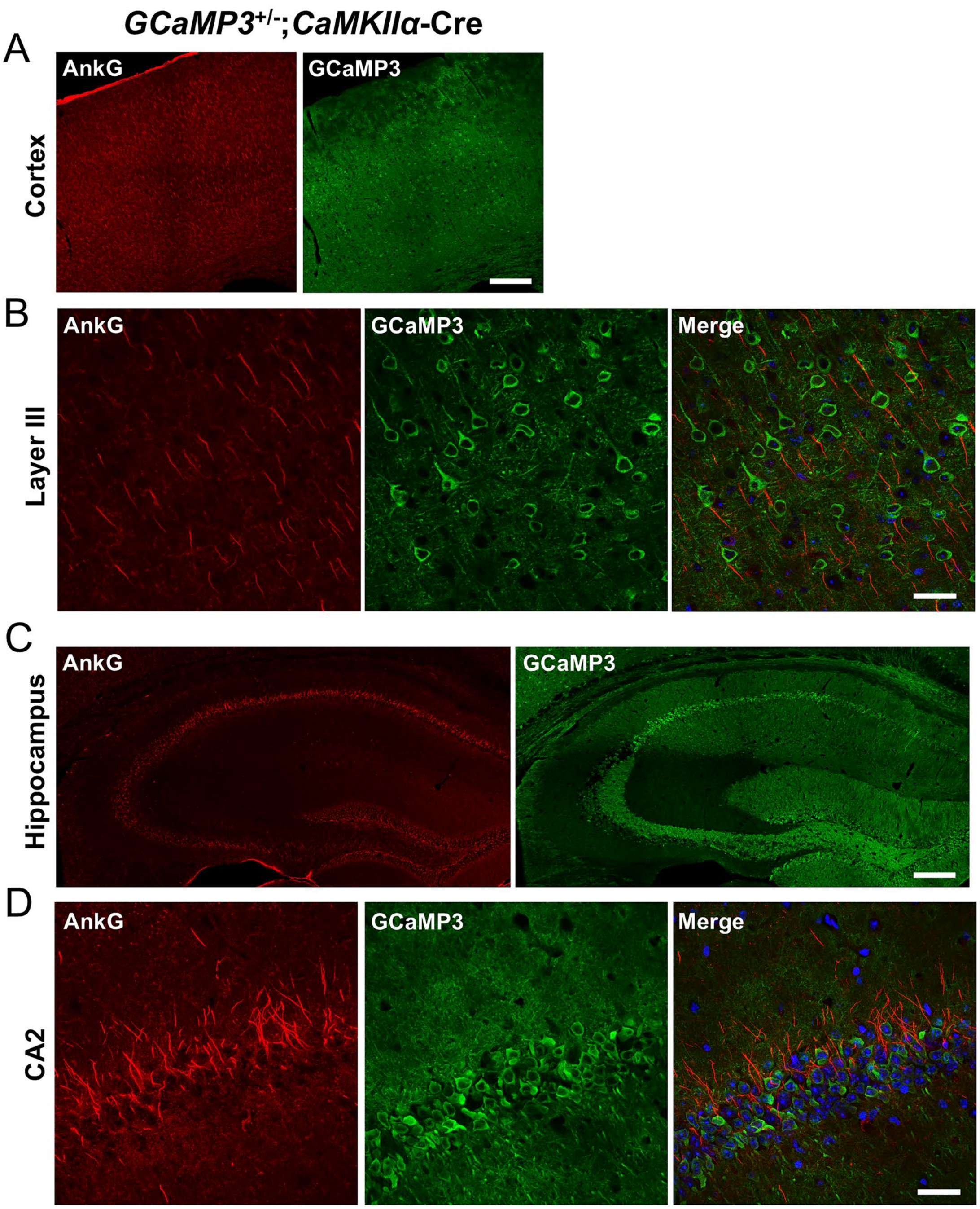

Fig. S4, related to Fig. 2. Expression patterns of ankyrin-G and GCaMP3 in the forebrain. A-D Representative images with ankyrin-G (detected in red), GCaMP3 (detected in green), and Hoechst (detected in blue) in the primary somatosensory cortex (A-B) or hippocampus (C-D) of the 18-week-old mouse (*GCaMP3*$^{+/-}$;*CaMKIIα*-Cre) brain. Magnified Layer 3 (B) or CA2 (D) images are shown. Scale bars: 200 μm (A, C) or 20 μm (B, D).

A

SynGO analysis

Ontology term	Category	Count	q-value	List
Synapse	CC	25	4.19E-09	Actr3, Atp6v0a1, Calr, Cnksr2, Creld1, Eif4e, Eif4g1, Git2, Grb2, Lsamp, Mlf2, Ptprn, Rps15a, Rps25, Slc6a7, Snx27, Taok2, Tnc, Wasf1, Wfs1, Ank3, Bin1, Cap2, Eif3d, Pfn2
Postsynapse	CC	15	1.46E-05	Actr3, Calr, Cnksr2, Eif4e, Eif4g1, Lsamp, Rps15a, Rps25, Snx27, Taok2, Wasf1, Ank3, Bin1, Cap2, Pfn2
Process in the synapse	BP	17	2.02E-05	Actr3, Arl8b, Atp6v0a1, Cnksr2, Eif4e, Git2, Nptxr, Rps15a, Slc6a7, Snx27, Taok2, Usp5, Wasf1, Ank3, Bin1, Cap2, Pfn2
Synapse organization	BP	9	1.98E-04	Actr3, Cnksr2, Nptxr, Snx27, Taok2, Wasf1, Ank3, Cap2, Pfn2
Modification of postsynaptic structure	BP	4	1.98E-04	Wasf1, Ank3, Cap2, Pfn2
Presynapse	CC	10	1.22E-03	Atp6v0a1, Git2, Grb2, Lsamp, Ptprn, Rps15a, Slc6a7, Wfs1, Bin1, Pfn2
Metabolism	BP	4	1.57E-03	Eif4e, Rps15a, Slc6a7, Usp5
Synaptic vesicle membrane	CC	4	2.36E-03	Atp6v0a1, Ptprn, Slc6a7, Wfs1
Integral component of synaptic vesicle membrane	CC	3	2.36E-03	Atp6v0a1, Slc6a7, Wfs1
Postsynapse organization	BP	3	4.85E-03	Actr3, Cnksr2, Taok2

B

STRING analysis (Diffusion output from Ank3; synaptic proteins are shown in bold.)

Layer	Heat threshold	List
1st	0.05 <	Cdk17, Fscn1, Gfap, Pdp1, **Ptprn**, Ryr2, **Bin1**, Mbp
2nd	5.50E-04 <	Actr3, **Calr**, **Grb2**, Nptxr, Pdia6, Rabgap1l, Rin1, Synpo2, Tagln2, Asph, **Cap2**, Pgls
3rd	9.40E-05 <	Arl8b, Asah1, Cnpy2, **Eif4e**, Gaa, **Git2**, Ktn1, Lgals1, Ncl, P4hb, Parp14, Pebp1, Psma6, Rpn1, **Slc6a7**, Tars, Tln1, **Tnc**, **Wasf1**, Impa1, **Pfn2**, Tbcb
	< 3.00E-05	**Atp6v0a1**, **Cnksr2**, **Creld1**, Ddx17, **Eif4g1**, Gusb, Igf2r, **Lsamp**, **Mlf2**, Nploc4, Pcbd1, Pex5l, Rab11fip1, **Rps15a**, **Rps25**, Scai, Sec14l2, Sez6l2, **Snx27**, Srsf4, **Taok2**, Usp5, Vwa5a, **Wfs1**, Coa3, Echdc2, **Eif3d**, Ilvbl, Lancl2, Uqcrc2, Vat1l

C

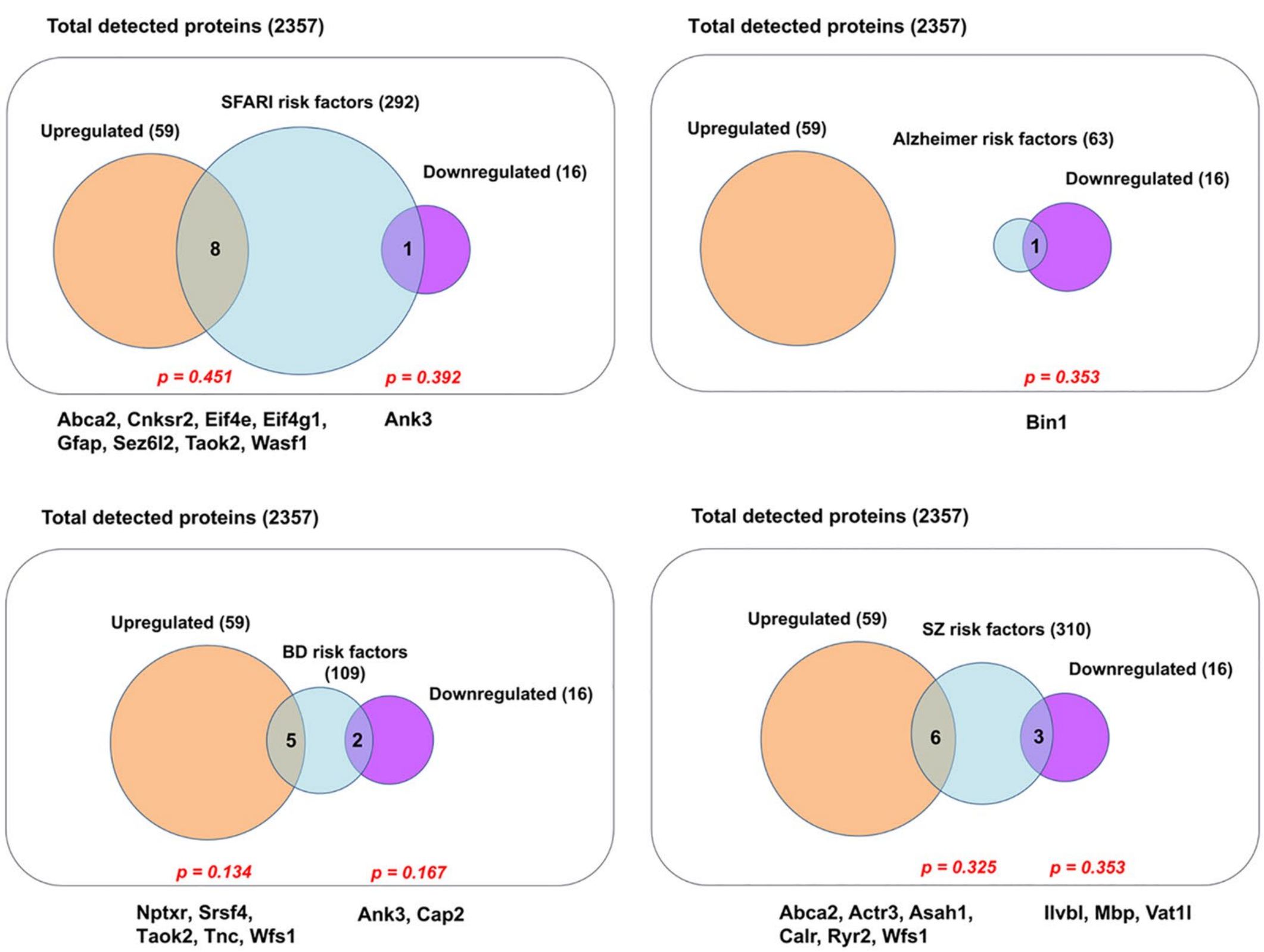

Fig. S5, related to Fig. 3. The altered protein levels in the forebrain of ankyrin-G knockout mice are associated with neurodevelopmental disorders or synaptic function. **A** Results of Gene Ontology analysis by SynGO of statistically overrepresented biological processes among the differentially upregulated (underlined in red) or downregulated (underlined in blue) proteins related to figure 3b. **B** The degree of network connectivity of ankyrin-G protein is categorized into three levels. The analysis was conducted using STRING v12.0 (https://string-db.org/). **C** Enrichment of, ASD risk factors (top left) from SFARI, Alzheimer's risk factors (top right), BD risk factors (bottom left), SZ risk factors (bottom right) identified through HGMD Professional among significantly altered protein expression levels in *Ank3*$^{-/-}$:*CaMKIIα*-Cre mice. SFARI, Simons Foundation Autism Research Initiative; ASD, autism spectrum disorder; SZ, schizophrenia; BD, bipolar disorder; HGMD, Human Gene Mutation Database.

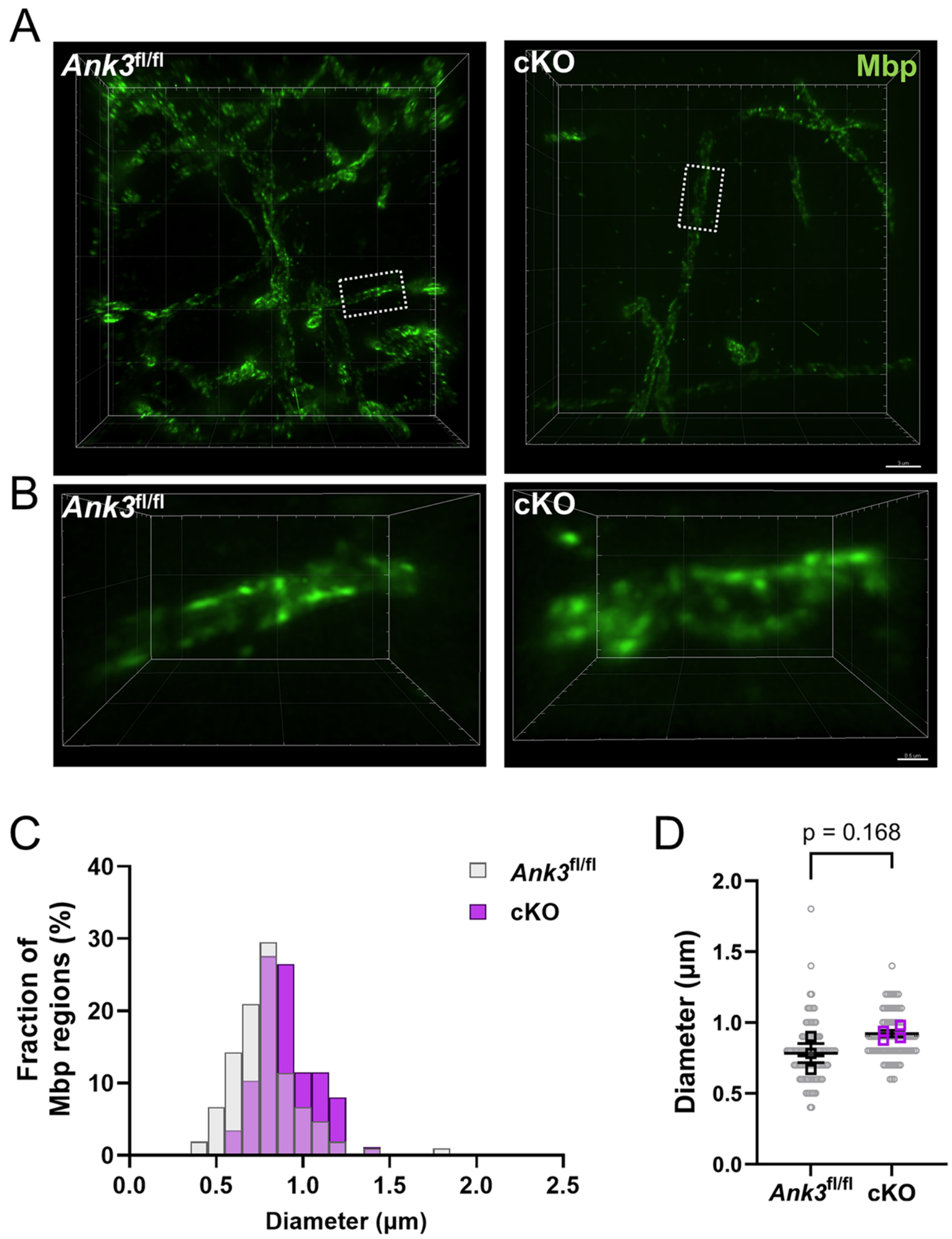

Fig. S6, related to Fig. 4. Myelin morphology in the primary somatosensory cortex of *Ank3* cKO mice. **A-B** Representative super-resolution images of Mbp immunofluorescence in layer 3 of the primary somatosensory cortex from 18-week-old *Ank3*$^{fl/fl}$ (left) and *Ank3*$^{-/-}$:*CaMKIIα*-Cre mice (right) acquired using a Nikon W1-SoRa microscope. Dashed boxes indicate the regions shown at higher magnification in (B). Scale bar, 3 µm (A) or 0.5 µm (B). **C** Distribution of Mbp-positive myelin sheath diameters. **D** Quantification of myelin sheath diameter. Data are presented as mean ± SEM (*Ank3*$^{fl/fl}$, n = 3; *Ank3* cKO, n = 4). Statistical significance was determined using an unpaired, two-tailed Student's t-test.

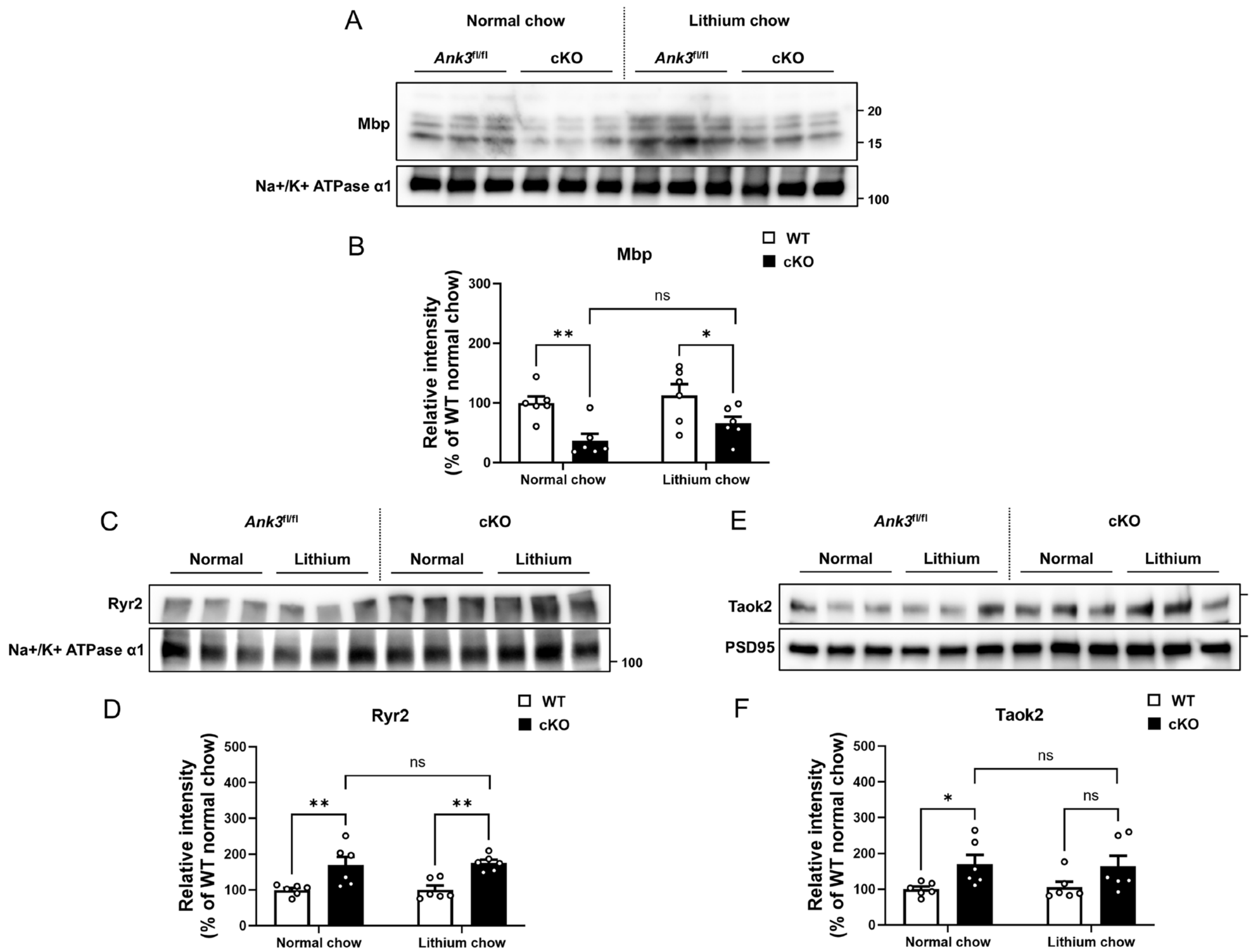

Fig. S7, related to Fig. 4. Lithium treatment partially restores Mbp expression levels in the membrane fraction of the forebrain of *Ank3*$^{-/-}$:*CaMKIIα*-Cre mice. A, C, E Representative immunoblots of the membrane (P2) or synaptosomal (P3) fraction prepared from the forebrains of 15-week-old *Ank3*$^{fl/fl}$ and *Ank3*$^{-/-}$:*CaMKIIα*-Cre mice fed either a normal chow or lithium-containing chow. Membranes were probed with antibodies against Mbp (A), RyR2 (C), and Taok2 (E). Na+/K+ ATPase α1 (as a membrane marker) and PSD95 (as a PSD marker) were used as loading controls. Each lane was loaded with 10 µg of protein. **B, D, F** Relative abundance of proteins. *p < 0.05; **p < 0.01; Two-way ANOVA followed by post hoc testing was performed. All data are presented as mean ± SEM.

Dataset S1 (separate file). Proteomic results from 10-plex TMT LC-MS multinotch analysis of forebrain tissue comparing *Ank3*$^{-/-}$:*CaMKIIα*-Cre and Ank3$^{flox/flox}$ male mice.

SI References

1. D. Zhou *et al.*, AnkyrinG is required for clustering of voltage-gated Na channels at axon initial segments and for normal action potential firing. *J Cell Biol* **143**, 1295-1304 (1998).
2. M. P. Leussis *et al.*, The ANK3 bipolar disorder gene regulates psychiatric-related behaviors that are modulated by lithium and stress. *Biol Psychiatry* **73**, 683-690 (2013).
3. M. G. Gottschalk *et al.*, Lithium reverses behavioral and axonal transport-related changes associated with ANK3 bipolar disorder gene disruption. *Eur Neuropsychopharmacol* **27**, 274-288 (2017).
4. C. Liu *et al.*, AnkG hemizygous mice present cognitive impairment and elevated anxiety/depressive-like traits associated with decreased expression of GABA receptors and postsynaptic density protein. *Exp Brain Res* **235**, 3375-3390 (2017).
5. I. M. van der Werf *et al.*, Behavioural characterization of AnkyrinG deficient mice, a model for ANK3 related disorders. *Behav Brain Res* **328**, 218-226 (2017).
6. S. Zhu *et al.*, Genetic disruption of ankyrin-G in adult mouse forebrain causes cortical synapse alteration and behavior reminiscent of bipolar disorder. *Proc Natl Acad Sci U S A* **114**, 10479-10484 (2017).
7. S. Yoon, K. Myczek, P. Penzes, cAMP Signaling-Mediated Phosphorylation of Diacylglycerol Lipase alpha Regulates Interaction With Ankyrin-G and Dendritic Spine Morphology. *Biol Psychiatry* **90**, 263-274 (2021).
