# Supplementary material for: Ank3 loss in adult forebrain excitatory neurons disrupts behavior, neuronal activity, membrane proteome, and myelination
Source: Proc Natl Acad Sci U S A. 2026 Aug 25;123(35):e2606900123. doi: 10.1073/pnas.2606900123 (PMC13535106; doi:10.1073/pnas.2606900123)
Supplement: Supplementary file 2 [file pnas.2606900123.sd01.pdf]

				Norm Int																	
Gene Nam	Accession	Spec Coun	Peptide Nu	WT	WT	WT	WT	WT	KO	KO	KO	KO	KO	t-test	T-Test p va	WT	KO		Fold Chang	q value	Description
Rabgap1	A2AWA9	1	1	NA	34982	NA	NA		34270	49182	44465	82561			#NUM!	34982	52619.5	Up	0.588985		Rab GTPase-activating protein 1 OS=Mus musculus GN=Rabgap1 PE=1 SV=1
Cacna1b	O55017	1	1	NA	NA	13621	14840	28052	NA	19746	NA	NA			#NUM!	18837.67	19746	Up	0.06794		Voltage-dependent N-type calcium channel subunit alpha-1B OS=Mus musculus GN=Cacna1b PE=1 SV=1
Cap2	Q9CYT6	1	1	NA	17167	15882	15096	19325	8016	9679	9634	6928	8158	0.000594	3.226214	16867.5	8483	Down	-0.9916	0.507746	Adenylyl cyclase-associated protein 2 OS=Mus musculus GN=Cap2 PE=1 SV=1
Tcea1	P10711	2	2	NA	4670	2764	3711	4224	NA	NA	NA	NA	5722		#NUM!	3842.25	5722	Up	0.574568		Transcription elongation factor A protein 1 OS=Mus musculus GN=Tcea1 PE=1 SV=2
Th	P24529	1	1	NA	NA	NA	5159	NA	7809	4832	4790	6601	16942		#NUM!	5159	8194.8	Up	0.667617		Tyrosine 3-monooxygenase OS=Mus musculus GN=Th PE=1 SV=3
Lancl2	Q9JJK2	1	1	NA	115823	105363	120152	116234	64111	67032	83444	97311	71494	0.001672	2.776764	114393	76678.4	Down	-0.57711	0.714227	LanC-like protein 2 OS=Mus musculus GN=Lancl2 PE=1 SV=1
Grn	P28798	1	1	NA	NA	NA	8234	NA	18677	8584	14587	7985	7955		#NUM!	8234	11557.6	Up	0.489176		Granulins OS=Mus musculus GN=Grn PE=1 SV=2
Dnajc5	P60904	4	4	NA	1980.5	NA	NA		4936	1367.5	2320.5	1691.5	1715		#NUM!	1980.5	2406.1	Up	0.280832		DnaJ homolog subfamily C member 5 OS=Mus musculus GN=Dnajc5 PE=1 SV=1
Vamp2	P63044	1	1	NA	NA	62808	NA	NA	NA	128858	NA	89744	NA		#NUM!	62808	109301	Up	0.799286		Vesicle-associated membrane protein 2 OS=Mus musculus GN=Vamp2 PE=1 SV=2
Ptn	P63089	1	1	NA	NA	2586	NA	NA	3428	NA	4413	NA			#NUM!	2586	3920.5	Up	0.600315		Pleiotrophin OS=Mus musculus GN=Ptn PE=2 SV=1
Gsk3a	Q2NL51	3	3	NA	43588.67	36922	41938.67	38228.67	35793.67	31061.33	30249	27312.33	30696.33	0.003704	2.431329	40169.5	31022.53	Down	-0.37278	0.731195	Glycogen synthase kinase-3 alpha OS=Mus musculus GN=Gsk3a PE=1 SV=2
Dbn1	Q9QXS6	5	5	303850.5	258802.8	250263	245954.3	286484.5	226734.3	227697.3	222349	197252	182242.8	0.004276	2.368962	269071	211255.1	Down	-0.349	0.731195	Drebrin OS=Mus musculus GN=Dbn1 PE=1 SV=4
Dbn1	Q9QXS6-2	4	4	303850.5	258802.8	250263	245954.3	286484.5	226734.3	227697.3	222349	197252	182242.8	0.004276	2.368962	269071	211255.1	Down	-0.349	0.731195	Isoform A2 of Drebrin OS=Mus musculus GN=Dbn1
Fbxo2	Q80UW2	3	3	265994	255263.7	247421.7	274974	245408.3	220739.7	232854.3	228449.3	230802	232182.3	0.004485	2.348238	257812.3	229005.5	Down	-0.17094	0.731195	F-box only protein 2 OS=Mus musculus GN=Fbxo2 PE=1 SV=1
Atp2a3	Q64518-3	1	1	NA	12495	15526	NA	NA				14039			#NUM!	14010.5	14039	Up	0.002932		Isoform SERCA3C of Sarcoplasmic/endoplasmic reticulum calcium ATPase 3 OS=Mus musculus GN=Atp2a3
Sez6	Q7TSK2-3	1	1	NA	NA	8795	NA	NA	13930	NA	NA	33410			#NUM!	8795	23670	Up	1.428304		Isoform 3 of Seizure protein 6 OS=Mus musculus GN=Sez6
Gopc	Q8BH60	1	1	NA	NA	NA	15507	NA	NA	NA	NA	23451	NA		#NUM!	15507	23451	Up	0.59673		Golgi-associated PDZ and coiled-coil motif-containing protein OS=Mus musculus GN=Gopc PE=1 SV=1
Wdr37	Q8CBE3	1	1	NA	NA	NA	11259	NA	NA	NA	58458	124992	NA		#NUM!	11259	91725	Up	3.026236		WD repeat-containing protein 37 OS=Mus musculus GN=Wdr37 PE=2 SV=1
Nup133	Q8R0G9	1	1	NA	NA	NA	NA	5139	NA	NA	8505	7375	NA		#NUM!	5139	7940	Up	0.627651		Nuclear pore complex protein Nup133 OS=Mus musculus GN=Nup133 PE=1 SV=2
Sh3bgrl3	Q91VW3	5	5	NA	172935	NA	142612	200034	NA	NA	NA	178382	NA		#NUM!	171860.3	178382	Up	0.053733		SH3 domain-binding glutamic acid-rich-like protein 3 OS=Mus musculus GN=Sh3bgrl3 PE=1 SV=1
L2hgdh	Q91YP0	1	1	NA	NA	8780	NA	NA		15502	16585	NA			#NUM!	8780	16043.5	Up	0.869696		L-2-hydroxyglutarate dehydrogenase, mitochondrial OS=Mus musculus GN=L2hgdh PE=1 SV=1
Psma8	Q9CWH6	1	1	NA	33264	NA	NA	NA	NA	NA	NA	39200	NA		#NUM!	33264	39200	Up	0.236892		Proteasome subunit alpha type-7-like OS=Mus musculus GN=Psma8 PE=2 SV=1
Ank3	G5E8K5-3	11	11	142129.6	177963.2	278537.6	259300.1	339523.7	88919.64	70942.82	83515.45	53558.55	74427.27	0.008836	2.053744	239490.9	74272.75	Down	-1.68907	0.731195	Isoform 3 of Ankyrin-3 OS=Mus musculus GN=Ank3
Ank3	G5E8K5-5	6	6	214093.8	258040.3	438586.8	401726.8	539198.8	119546	91250	114380.7	60215.17	83347.33	0.00889	2.051098	370329.3	93747.83	Down	-1.98195	0.731195	Isoform 5 of Ankyrin-3 OS=Mus musculus GN=Ank3
Cops7a	Q9CZ04	1	1	NA	NA	NA	NA	7312	NA	15422	NA	NA			#NUM!	7312	15422	Up	1.076652		COP9 signalosome complex subunit 7a OS=Mus musculus GN=Cops7a PE=1 SV=2
Mrpl13	Q9D1P0	1	1	NA	NA	NA	26863	NA	NA	30767	NA	NA			#NUM!	26863	30767	Up	0.195763		39S ribosomal protein L13, mitochondrial OS=Mus musculus GN=Mrpl13 PE=2 SV=1
Gda	Q9R111	1	1	NA	NA	NA	24579	NA	NA	39417	NA	NA			#NUM!	24579	39417	Up	0.681392		Guanine deaminase OS=Mus musculus GN=Gda PE=1 SV=1
Fscn1	Q61553	11	11	138426.3	129911.3	150589.5	156434.4	137844.8	188245.6	194909.1	175755.2	189988.2	207815.9	0.000128	3.89279	142641.2	191342.8	Up	0.423769	0.167254	Fascin OS=Mus musculus GN=Fscn1 PE=1 SV=4
Ptprn	Q60673	2	2	18220.5	25766	23421	32928.5	25199	46586.5	44973.5	52569	47481	41048	0.000131	3.882729	25107	46531.6	Up	0.890121	0.167254	Receptor-type tyrosine-protein phosphatase-like N OS=Mus musculus GN=Ptprn PE=1 SV=2
Cul3	Q9JLV5	2	2	531767	536907	490773	435771.5	496630.5	345295	470859.5	410150.5	390810	323896	0.009666	2.014753	498369.8	388202.2	Down	-0.36041	0.731195	Cullin-3 OS=Mus musculus GN=Cul3 PE=1 SV=1
Tbcb	Q9D1E6	4	4	32199.25	18880.25	38625	34350.25	41299	21099.25	13341.75	15841.5	13246.25	21291.25	0.01056	1.976336	33070.75	16964	Down	-0.96308	0.731195	Tubulin-folding cofactor B OS=Mus musculus GN=Tbcb PE=1 SV=2
Git2	Q9JLQ2	1	1	19053	21789	24626	23269	20064	26757	31670	26609	29720	29289	0.001003	2.998699	21760.2	28809	Up	0.404828	0.642589	ARF GTPase-activating protein GIT2 OS=Mus musculus GN=Git2 PE=1 SV=2
Bin1	O08539	17	17	997549.5	777068.4	770259.6	744527.8	729882.3	685819.2	606515.7	563383.6	579551.3	574720.1	0.011031	1.957385	803857.5	601998	Down	-0.41718	0.731195	Myc box-dependent-interacting protein 1 OS=Mus musculus GN=Bin1 PE=1 SV=1
Nrcam	Q810U4-5	1	1	239282	321024	311320	284631	279627	408733	360358	376993	346395	382102	0.001449	2.838932	287176.8	374916.2	Up	0.384629	0.714227	Isoform 5 of Neuronal cell adhesion molecule OS=Mus musculus GN=Nrcam
Nptxr	Q99J85	5	5	54728.75	70396	66281.25	76674.5	56727.25	116594	86698.75	100880	89523.5	87858.5	0.002599	2.585194	64961.55	96310.95	Up	0.568114	0.731195	Neuronal pentraxin receptor OS=Mus musculus GN=Nptxr PE=1 SV=1
Taok2	Q6ZQ29-2	1	1	8476	21421	18316	25289	16850	32302	29465	33312	37580	34668	0.002904	2.537003	18070.4	33465.4	Up	0.889042	0.731195	Isoform 2 of Serine/threonine-protein kinase TAO2 OS=Mus musculus GN=Taok2
Ddx17	Q501J6	4	4	39053.25	60596	58927.25	53263.25	62720	64460.75	75821.25	83386.25	84752.5	84136.75	0.003508	2.45494	54911.95	78511.5	Up	0.515784	0.731195	Probable ATP-dependent RNA helicase DDX17 OS=Mus musculus GN=Ddx17 PE=2 SV=1
Scai	Q8C8N2	1	1	36874	86533	77068	88615	57776	109538	118002	102001	130124	144985	0.003606	2.442974	69373.2	120930	Up	0.801722	0.731195	Protein SCAI OS=Mus musculus GN=Scai PE=1 SV=2
Clint1	Q99KN9	1	1	78186	66266	61311	65461	64168	80177	81419	80570	76207	89053	0.004631	2.334325	67078.4	81485.2	Up	0.28069	0.731195	Clathrin interactor 1 OS=Mus musculus GN=Clint1 PE=1 SV=2
Cdk17	Q8K0D0	1	1	26350	31696	24532	25723	27519	40022	29380	35443	37434	37654	0.004849	2.314348	27164	35986.6	Up	0.405764	0.731195	Cyclin-dependent kinase 17 OS=Mus musculus GN=Cdk17 PE=1 SV=2
Rab11fip1	Q9D620	1	1	26815	54526	38406	39343	25976	57533	59082	63763	55588	74127	0.005139	2.291281	37013.2	62018.6	Up	0.744661	0.731195	Rab11 family-interacting protein 1 OS=Mus musculus GN=Rab11fip1 PE=1 SV=2
Tln1	P26039	9	9	35474	60462.56	51455.44	52253.22	52543.56	62567.67	66723.11	70624	77585.11	71585.33	0.005466	2.26233	50457.76	69817.04	Up	0.469075	0.731195	Talin-1 OS=Mus musculus GN=Tln1 PE=1 SV=2
Calr	P14211	12	12	499782.7	736809.8	403384.4	568573.5	499203.7	812894.8	761541.5	759843.5	908927.7	1116603	0.00565	2.255707	541550.8	871962	Up	0.687169	0.731195	Calreticulin OS=Mus musculus GN=Calr PE=1 SV=1
Myh9	Q8VDD5	10	10	81642.7	106343.6	97019.8	93496.5	89971.4	107043.8	109483.4	108761.3	119810.5	127993.9	0.006414	2.192871	93694.8	114618.6	Up	0.2908	0.731195	Myosin-9 OS=Mus musculus GN=Myh9 PE=1 SV=4
Wfs1	P56695	3	3	315341.7	431502	566897	455463.7	448862.3	630611.3	637606.3	665527.3	648382.3	540063.7	0.006915	2.160208	443613.3	624438.2	Up	0.493256	0.731195	Wolframin OS=Mus musculus GN=Wfs1 PE=1 SV=1
Gusb	P12265	1	1	48087	71869	70331	70217	73754	106367	90511	108793	74004	102036	0.006975	2.156456	66851.6	96342.2	Up	0.527206	0.731195	Beta-glucuronidase OS=Mus musculus GN=Gusb PE=2 SV=2
Ckap4	Q8BMK4	1	1	167108	248948	234386	189421	211315	236936	279084	273632	304549	280901	0.008906	2.050317	210235.6	275020.4	Up	0.387532	0.731195	Cytoskeleton-associated protein 4 OS=Mus musculus GN=Ckap4 PE=1 SV=2
Dhodh	O35435	1	1	NA	66713	80888	87230	74668	62125	57340	51913	61989	61693	0.017017	1.769117	77374.75	59012	Down	-0.39085	0.731195	Dihydroorotate dehydrogenase (quinone), mitochondrial OS=Mus musculus GN=Dhodh PE=2 SV=2
Gfap	P03995	8	8	142031.1	154449	172193.1	209212.9	154617.1	178393.3	242279	230254	230599	262077.6	0.009319	2.030631	166500.7	228720.6	Up	0.458058	0.731195	Glial fibrillary acidic protein OS=Mus musculus GN=Gfap PE=1 SV=4
Actr3	Q99JY9	4	4	49006	81116.75	66011.75	68947.25	65417.25	91248.5	94867.5	87973	77556.75	87428	0.00936	2.028724	66099.8	87814.75	Up	0.409817	0.731195	Actin-related protein 3 OS=Mus musculus GN=Actr3 PE=1 SV=3
Palm	Q9Z0P4-2	9	9	266677.4	365911.1	368125.6	323008.6	375976.4	453478	384263.1	461307.4	408333.7	432708.3	0.009385	2.027566	339939.8	428018.1	Up	0.332392	0.731195	Isoform 2 of Paralemmin-1 OS=Mus musculus GN=Palm
Klc1	O88447	4	4	135971.3	165184.8	170956.3	177051.8	177445.5	180612	208538.5	187216.3	215070.3	211226	0.009464	2.023925	165321.9	200532.6	Up	0.278559	0.731195	Kinesin light chain 1 OS=Mus musculus GN=Klc1 PE=1 SV=3
Rabgap1l	A6H6A9-3	2	2	80021.5	57418.5	71747	45864	60142.5	80446.5	78492.5	96045.5	84782.5	98141.5	0.010846	1.96473	63038.7	87581.7	Up	0.474392	0.731195	Isoform 3 of Rab GTPase-activating protein 1-like OS=Mus musculus GN=Rabgap1l
Psma6	Q9QUM9	4	4	70162.67	113735	75860.67	107890.3	110346.3	123775.3	125938	124492	139798.7	163426	0.011124	1.953739	95599	135486	Up	0.503076	0.731195	Proteasome subunit alpha type-6 OS=Mus musculus GN=Psma6 PE=1 SV=1
Gspt1	Q8R050	2	2	210533	287694.5	257964.5	263092	297175	269527.5	304414	341309	318887.5	367992.5	0.011215	1.950201	263291.8	320426.1	Up	0.283493	0.731195	Eukaryotic peptide chain release factor GTP-binding subunit ERF3A OS=Mus musculus GN=Gspt1 PE=1 SV=2
Pfkp	Q9WUA3	11	11	342589.4	329344.7	325254.7	355359.6	302064.1	269952.5	332780.2	270324.6	284180.8	278675.9	0.019314	1.714128	330922.5	287182.8	Down	-0.20452	0.731195	6-phosphofructokinase type C OS=Mus musculus GN=Pfkp PE=1 SV=1
Sec14l2	Q99J08	1	1	127085	256922	195820	190141	191555	226801	276267	318568	268875	302089	0.011372	1.944163	192304.6	278520	Up	0.534388	0.731195	SEC14-like protein 2 OS=Mus musculus GN=Sec14l2 PE=1 SV=1
Pcbp2	Q61990	9	9	370102.6	361792.9	357673.4	331783.9	327552.3	359679.6	410122.3	397397	408486.9	381294	0.011412	1.942638	349781	391395.9	Up	0.162177	0.731195	Poly(rC)-binding protein 2 OS=Mus musculus GN=Pcbp2 PE=1 SV=1
Ndst3	Q9EQH7	1	1	20517	19258	19555	18246	18795	20233	22857	21975	21520	20282	0.011631	1.934383	19274.2	21373.4	Up	0.149145	0.731195	Bifunctional heparan sulfate N-deacetylase/N-sulfotransferase 3 OS=Mus musculus GN=Ndst3 PE=2 SV=2
Pdp1	Q3UV70	1	1	32787	37195	38189	36799	43063	48993	50085	60986	46877	42486	0.011943	1.922887	37606.6	49885.4	Up	0.407632	0.731195	[Pyruvate dehydrogenase [acetyl-transferring]]-phosphatase 1, mitochondrial OS=Mus musculus GN=Pdp1 PE=2 SV=1
Tnc	Q80YX1-3	1	1	3226	6035	5585	6653	4530	6576	8910	6640	8359	8104	0.012161	1.915031	5205.8	7717.8	Up	0.56807	0.731195	Isoform 3 of Tenascin OS=Mus musculus GN=Tnc
Rps18	P62270	2	2	594740	693282	651595.5	526174	620285.5	783127.5	724382	707045.5	695228	733847.5	0.012411	1.906193	617215.4	728726.1	Up	0.239603	0.731195	40S ribosomal protein S18 OS=Mus musculus GN=Rps18 PE=1 SV=3
P4hb	P09103	8	8	117230.8	155396.5	169290.8	189024.8	137059.3	193217.5	196973.3	205623	196395.7	223829.3	0.012513	1.902639	153600.5	203207.8	Up	0.403773	0.731195	Protein disulfide-isomerase OS=Mus musculus GN=P4hb PE=1 SV=2
Srsf4	Q8VE97	1	1	45195	83892	82419	85530	83579	95921	97126	117170	118141	146845	0.00555	1.897738	76123	115040.6	Up	0.595739	0.731195	Serine/arginine-rich splicing factor 4 OS=Mus musculus GN=Srsf4 PE=2 SV=1
Creld1	Q91XD7	2	2	NA	1808	3107	1692	3493	5675	3355	4934	6887	8400	0.014201	1.847681	2525	5850.2	Up	1.212203	0.731195	Cysteine-rich with EGF-like domain protein 1 OS=Mus musculus GN=Creld1 PE=2 SV=1
Impa1	O55023	1	1	98177	87367	71388	67439	96823	66849	74629	57554	62242	42966	0.022977	1.638707	84238.8	60848	Down	-0.46928	0.731195	Inositol monophosphatase 1 OS=Mus musculus GN=Impa1 PE=1 SV=1
Eif3i	Q9QZD9	1	1	21635	25750	27656	21918	27056	34273	28872	28942	35520	28025	0.014523	1.837944	24803	31126.4	Up	0.327624	0.731195	Eukaryotic translation initiation factor 3 subunit I OS=Mus musculus GN=Eif3i PE=1 SV=1
Lrpap1	P55302	6	6	82840.33	118219.8	103359.7	98148.83	130213.8	130151	132388.7	151997	140624.5	130036.2	0.016235	1.789548	106556.5	137039.5	Up	0.362973	0.731195	Alpha-2-macroglobulin receptor-associated protein OS=Mus musculus GN=Lrpap1 PE=1 SV=1
Mlf2	Q99KX1	4	4	63875	98825.75	105529.3	86048.75	98473	130410.8	112586	129386.8	191199.5	136702.3	0.016856	1.773245	90550.35	140057.1	Up	0.629222	0.731195	Myeloid leukemia factor 2 OS=Mus musculus GN=Mlf2 PE=1 SV=1
Rpn1	Q91YQ5	3	3	715538	1128219	1059117	872930	964783	1315374	1266066	1554687	1204015	1050263	0.017109	1.766775	948117.4	1278081	Up	0.430842	0.731195	Dolichyl-diphosphooligosaccharide--protein glycosyltransferase subunit 1 OS=Mus musculus GN=Rpn1 PE=1 SV=1
Ddx5	Q61656	6	6	82634.33	88389.5	101216	93516.67	97835	93731.5	134371.5	113806	127319.5	129422.5	0.017172	1.765179	92718.3	119730.2	Up	0.368861	0.731195	Probable ATP-dependent RNA helicase DDX5 OS=Mus musculus GN=Ddx5 PE=1 SV=2
Aplp2	Q06335	1	1	101942	105766	108078	79421	93766	106458	108218	125822	133298	134948	0.017749	1.750826	97794.6	121748.8	Up	0.316081	0.731195	Amyloid-like protein 2 OS=Mus musculus GN=Aplp2 PE=1 SV=4
Hecw1	Q8K4P8	1	1	NA	70031	61871	60342	50622	70565	77383	73915	81952	80456	0.017992	1.744921	60716.5	76854.2	Up	0.340035	0.731195	E3 ubiquitin-protein ligase HECW1 OS=Mus musculus GN=Hecw1 PE=1 SV=3
Palm	Q9Z0P4	11	11	234460.8	297389.3	298467.6	276587.8	321432.7	364412.9	300575.1	358364.9	336892.4	343745.9	0.01866	1.729088	285667.6	340798.2	Up	0.25458	0.731195	Paralemmin-1 OS=Mus musculus GN=Palm PE=1 SV=1
Ddx17	Q501J6-2	1	1	NA	37824	28804	38106	22270	111017	56085	59654	66985	55454	0.018967	1.722001	31751	69839	Up	1.137231	0.731195	Isoform 2 of Probable ATP-dependent RNA helicase DDX17 OS=Mus musculus GN=Ddx17
Ryr2	E9Q401	1	1	3332	8289	7705	12701	10029	10925	12631	13803	17374	19553	0.019209	1.716495	8411.2	14857.2	Up	0.820779	0.731195	Ryanodine receptor 2 OS=Mus musculus GN=Ryr2 PE=1 SV=1
Ncl	P09405	3	3	53952.67	126409.7	105453	123588	113244.7	130267.3	146376.7	155653.7	175171.7	149579.7	0.019771	1.703971	104529.6	151409.8	Up	0.534547	0.731195	Nucleolin OS=Mus musculus GN=Ncl PE=1 SV=2
Snd1	Q78PY7	8	8	284395.3	271493.8	263580.4	217521.9	241386.1	307913.5	291266	292968.9	311539.1	283383.9	0.020249	1.693596	255675.5	297414.3	Up	0.21816	0.731195	Staphylococcal nuclease domain-containing protein 1 OS=Mus musculus GN=Snd1 PE=1 SV=1
Gphn	Q8BUV3	14	14	130949.8	119942	124530.6	129424.3	128153.9	122031	119334.9	113372.9	113455.3	98100.42	0.028641	1.543012	126600.1	113258.9	Down	-0.16065	0.731195	Gephyrin OS=Mus musculus GN=Gphn PE=1 SV=2
Smap2	Q7TN29	1	1	13664	14935	14275	13198	15020	16816	14053	16433	16331	16497	0.020685	1.684344	14218.4	16026	Up	0.172655	0.731195	Stromal membrane-associated protein 2 OS=Mus musculus GN=Smap2 PE=1 SV=1
Pde12	Q3TIU4	1	1	NA	517546	479634	453139	407260	651416	541964	568590	495190	677432	0.021819	1.661165	464394.8	586918.4	Up	0.337808	0.731195	2',5'-phosphodiesterase 12 OS=Mus musculus GN=Pde12 PE=2 SV=2
Atp5a1	Q03265	38	38	443130.4	329547.7	351784.8	362507.3	364988.3	349808.3	309060.6	317020.4	289394	265252.8	0.029535	1.529663	370391.7	306107.2	Down	-0.27501	0.731195	ATP synthase subunit alpha, mitochondrial OS=Mus musculus GN=Atp5a1 PE=1 SV=1
Actr1a	P61164	4	4	191727.8	195975.3	215616.3	181509.8	191776.3	177618.3	192901.3	175921.5	162197.8	166070.5	0.030208	1.519878	195321.1	174941.9	Down	-0.15897	0.731195	Alpha-centractin OS=Mus musculus GN=Actr1a PE=2 SV=1
Kif3b	Q61771	2	2	347241	293196	246931	254561	238392	396385	345954	316560	319759	362973	0.021925	1.65906	276064.2	348326.2	Up	0.335435	0.731195	Kinesin-like protein KIF3B OS=Mus musculus GN=Kif3b PE=1 SV=1
Rtn4rl1	Q8K0S5	2	2	17094.5	26076	24371.5	24556.5	24617	30750	28146.5	29557	28886	26856	0.022184	1.65396	23343.1	28839.1	Up	0.30503	0.731195	Reticulon-4 receptor-like 1 OS=Mus musculus GN=Rtn4rl1 PE=2 SV=1
Cdh13	Q9WTR5	1	1	13154	15600	13562	10966	11550	17488	13770	15759	15596	17347	0.022245	1.652768	12966.4	15992	Up	0.302572	0.731195	Cadherin-13 OS=Mus musculus GN=Cdh13 PE=1 SV=2
Dlg1	Q811D0-3	11	11	60838.9	77626.1	84735.8	76072.5	89324	102681.9	102820.8	98157.7	82704.9	90679.1	0.022601	1.645872	77719.46	95408.88	Up	0.295848	0.731195	Isoform 3 of Disks large homolog 1 OS=Mus musculus GN=Dlg1
Vdac3	Q60931	18	18	303907.3	246357.5	247602.2	271013.1	270132.2	251185.2	204266.6	207021.3	258146.9	216318.3	0.030997	1.50868	267802.4	227387.7	Down	-0.23602	0.731195	Voltage-dependent anion-selective channel protein 3 OS=Mus musculus GN=Vdac3 PE=1 SV=1
Ilvbl	Q8BU33	2	2	35240.5	50268	52834	49397.5	71629.5	27419	20154	31191	44775	39554.5	0.031326	1.504095	51873.9	32618.7	Down	-0.66931	0.731195	Acetolactate synthase-like protein OS=Mus musculus GN=Ilvbl PE=2 SV=1
Ldha	P06151	1	1	1388961	1543442	1119617	1257793	1419576	1279219	1115486	1047715	1050295	1085512	0.031475	1.502034	1345878	1115645	Down	-0.27067	0.731195	L-lactate dehydrogenase A chain OS=Mus musculus GN=Ldha PE=1 SV=3
Rps25	P62852	2	2	134513	246488	235202	239116	184596	258185	307788	230030	320801	325663	0.022744	1.643133	207983	288493.4	Up	0.472073	0.731195	40S ribosomal protein S25 OS=Mus musculus GN=Rps25 PE=1 SV=1
Clta	O08585	11	11	98518	73198	79605.5	79399.5	85771.5	72253	76769	68335	70807	59433.5	0.03217	1.492549	83298.5	69519.5	Down	-0.26087	0.731195	Clathrin light chain A OS=Mus musculus GN=Clta PE=1 SV=2
Cyc1	Q9D0M3	2	2	547200	561852	642060	573617	625381	572294.5	517342	514922.5	435493	532970.5	0.032344	1.490206	590022	514604.5	Down	-0.1973	0.731195	Cytochrome c1, heme protein, mitochondrial OS=Mus musculus GN=Cyc1 PE=1 SV=1
Arl8b	Q9CQW2	1	1	NA	65234	50535	39410	NA	NA	129001	67396	108690	131457	0.022991	1.638442	51726.33	109136	Up	1.077156	0.731195	ADP-ribosylation factor-like protein 8B OS=Mus musculus GN=Arl8b PE=2 SV=1
Echdc2	Q3TLP5-3	1	1	149638	95591	115793	94514	113771	70288	93503	82590	85633	82155	0.032558	1.487342	113861.4	82833.8	Down	-0.45899	0.731195	Isoform 3 of Enoyl-CoA hydratase domain-containing protein 2, mitochondrial OS=Mus musculus GN=Echdc2
Nme2	Q01768	6	6	552620.7	443062.3	528237.2	471548.2	450618.7	555015.7	570285.2	557991.8	579026.7	561728	0.02405	1.618885	489217.4	564809.5	Up	0.207289	0.731195	Nucleoside diphosphate kinase B OS=Mus musculus GN=Nme2 PE=1 SV=1
Eif4g1	Q6NZJ6-2	4	4	89548	164149.8	179783	169335	193251	207788.3	221997.8	225958.5	244213.5	202057.5	0.024074	1.618452	159213.4	220403.1	Up	0.469183	0.731195	Isoform 2 of Eukaryotic translation initiation factor 4 gamma 1 OS=Mus musculus GN=Eif4g1
Nrcam	Q810U4-3	11	11	350141.5	462257.9	412311.1	391675.6	390988.5	457249.9	458396.8	443202.9	478673.6	477974.4	0.024332	1.613822	401474.9	463099.5	Up	0.206012	0.731195	Isoform 3 of Neuronal cell adhesion molecule OS=Mus musculus GN=Nrcam
Pgls	Q9CQ60	1	1	75710	53876	77575	65752	96733	76873	37943	43607	41147	39308	0.033498	1.474981	73929.2	47775.6	Down	-0.62987	0.731195	6-phosphogluconolactonase OS=Mus musculus GN=Pgls PE=2 SV=1
Gapdh	P16858	65	65	826212.5	978045.3	961046	958315.1	872135.7	1126572	1008514	937959.9	1205934	1169290	0.024567	1.609648	919150.9	1089654	Up	0.245496	0.731195	Glyceraldehyde-3-phosphate dehydrogenase OS=Mus musculus GN=Gapdh PE=1 SV=2
Eif4g1	Q6NZJ6	5	5	71638.4	132743.6	144943.8	136571.8	156114.4	166807.6	179356	182550.4	196623.4	163117.2	0.02505	1.601192	128402.4	177690.9	Up	0.468698	0.731195	Eukaryotic translation initiation factor 4 gamma 1 OS=Mus musculus GN=Eif4g1 PE=1 SV=1
Eif4e	P63073	1	1	513595	600992	527348	595343	393874	727154	691803	537275	742242	885353	0.025056	1.601088	526230.4	716765.4	Up	0.445806	0.731195	Eukaryotic translation initiation factor 4E OS=Mus musculus GN=Eif4e PE=1 SV=1
Ktn1	O61595-1:	6	6	58689.2	81336	92135	68673.6	100437.8	112091.8	104978.4	118496.4	96587.6	96033	0.025375	1.595594	80254.32	105637.4	Up	0.39647	0.731195	Isoform 11 of Kinectin OS=Mus musculus GN=Ktn1
Lsamp	Q8BLK3	12	12	85272.9	197707	162076.1	190788.8	154261.7	231147.8	207038.6	214583.5	220738.9	249854.6	0.02539	1.595337	158021.3	224672.7	Up	0.507706	0.731195	Limbic system-associated membrane protein OS=Mus musculus GN=Lsamp PE=1 SV=1
Sec63	Q8VHE0	1	1	50126	51634	70352	62360	69713	68139	85614	76789	86457	67568	0.026606	1.57502	60837	76913.4	Up	0.338286	0.731195	Translocation protein SEC63 homolog OS=Mus musculus GN=Sec63 PE=2 SV=4
Grm2	Q14BI2	4	4	88302	91786	94705.25	100903.5	97565.25	72574.25	89831.75	85155.75	74396.75	91332.75	0.034561	1.461414	94652.4	82658.25	Down	-0.19548	0.731195	Metabotropic glutamate receptor 2 OS=Mus musculus GN=Grm2 PE=1 SV=2
Sez6l2	Q4V9Z5-3	4	4	NA	26588.5	35879.5	25047.25	20637	47921	31043.5	45152.75	35917.25	37416.5	0.027616	1.558839	27038.06	39490.2	Up	0.546503	0.731195	Isoform 3 of Seizure 6-like protein 2 OS=Mus musculus GN=Sez6l2
Ak3	Q9WTP7	5	5	113140.8	122446.6	111787	137929	120443.6	122228	101463	100899.8	98729.6	101880.2	0.035395	1.451058	121149.4	105040.1	Down	-0.20585	0.731195	GTP:AMP phosphotransferase AK3, mitochondrial OS=Mus musculus GN=Ak3 PE=1 SV=3
Grm3	Q9QYS2	1	1	NA	93772	88352	94210	105358	57965	87406	91460	73208	75543	0.035895	1.444966	95423	77116.4	Down	-0.3073	0.731195	Metabotropic glutamate receptor 3 OS=Mus musculus GN=Grm3 PE=2 SV=1
Aars	Q8BGQ7	6	6	132265.4	101785.8	122639	125261	146819.6	111614.8	116575.4	100832.6	94275.8	73903	0.036227	1.440968	125754.2	99440.32	Down	-0.3387	0.731195	Alanine--tRNA ligase, cytoplasmic OS=Mus musculus GN=Aars PE=1 SV=1
Klc4	Q9DBS5	2	2	271942.5	269207	270591	305052	276889.5	289608.5	347472.5	309232	318563.5	376780	0.02778	1.556268	278736.4	328331.3	Up	0.236251	0.731195	Kinesin light chain 4 OS=Mus musculus GN=Klc4 PE=1 SV=1
Mdh2	P08249	84	84	444872.7	414973.8	330571.8	417987.6	387255.4	363073.6	339694	353319.5	312621.2	335093.2	0.036422	1.438636	399132.3	340760.3	Down	-0.22811	0.731195	Malate dehydrogenase, mitochondrial OS=Mus musculus GN=Mdh2 PE=1 SV=3
Gaa	P70699	3	3	NA	17205.67	52157	30323.67	16084.33	99710	100292.3	46639	76746.33	37966	0.028691	1.542254	28942.67	72270.73	Up	1.320214	0.731195	Lysosomal alpha-glucosidase OS=Mus musculus GN=Gaa PE=1 SV=2
Syt13	Q9EQT6	1	1	136503	108052	125146	115036	154962	172465	160063	193152	139133	145454	0.02877	1.54106	127939.8	162053.4	Up	0.341004	0.731195	Synaptotagmin-13 OS=Mus musculus GN=Syt13 PE=1 SV=1
Dynll2	Q9D0M5	2	2	459278.5	364531	423847	421326	424032.5	474983	499530.5	478866.5	464706	429454.5	0.030415	1.516912	418603	469508.1	Up	0.165567	0.731195	Dynein light chain 2, cytoplasmic OS=Mus musculus GN=Dynll2 PE=1 SV=1
Suclg1	Q9WUM5	26	26	1010355	748068.9	773735.1	827776.3	798306.8	810868.9	703818.3	649915.5	560470.8	659817.4	0.037526	1.425668	831648.5	676978.2	Down	-0.29686	0.731195	Succinyl-CoA ligase [ADP/GDP-forming] subunit alpha, mitochondrial OS=Mus musculus GN=Suclg1 PE=1 SV=4
Ap2b1	Q9DBG3	6	6	156693.2	206184.3	199630.2	227309.7	181833.2	214721	223160	226806.7	261373.2	239753.5	0.03054	1.515131	194330.1	233162.9	Up	0.262829	0.731195	AP-2 complex subunit beta OS=Mus musculus GN=Ap2b1 PE=1 SV=1
Eif3d	O70194	1	1	31893	22299	23538	22299	27331	13003	22592	15845	20673	22278	0.037855	1.421877	25472	18878.2	Down	-0.43219	0.731195	Eukaryotic translation initiation factor 3 subunit D OS=Mus musculus GN=Eif3d PE=1 SV=2
Blvra	Q9CY64	1	1	52696	64184	62073	60499	52949	60872	66756	65536	76223	79268	0.030938	1.509508	58480.2	69731	Up	0.253852	0.731195	Biliverdin reductase A OS=Mus musculus GN=Blvra PE=2 SV=1
Tars	Q9D0R2	1	1	NA	66380	96568	80248	52587	86187	112274	117604	109144	107241	0.030988	1.508806	73945.75	106490	Up	0.526179	0.731195	Threonine--tRNA ligase, cytoplasmic OS=Mus musculus GN=Tars PE=1 SV=2
Sec23a	Q01405	2	2	86191	98198	90553.5	112609	132494.5	148042.5	125555	158224.5	127526.5	113129.5	0.031632	1.499873	104009.2	134495.6	Up	0.370848	0.731195	Protein transport protein Sec23A OS=Mus musculus GN=Sec23a PE=1 SV=2
Asah1	Q9WV54	4	4	45905.75	139033.8	154853.5	148406.8	133052	192329.3	165737.3	190229.8	160193	226734.8	0.032474	1.488464	124250.4	187044.8	Up	0.590134	0.731195	Acid ceramidase OS=Mus musculus GN=Asah1 PE=1 SV=1
Cnksr2	Q80YA9-2	5	5	68805.25	91780.5	139924.3	137909.3	108798.3	113608.8	138791.5	207002.5	199722.8	221945.5	0.033254	1.478156	109443.5	176214.2	Up	0.687144	0.731195	Isoform 2 of Connector enhancer of kinase suppressor of ras 2 OS=Mus musculus GN=Cnksr2
Akap2	O54931-3	1	1	108917	87563	96418	77489	92037	83324	77700	81917	69465	73485	0.038878	1.410296	92484.8	77178.2	Down	-0.26102	0.731195	Isoform 3 of A-kinase anchor protein 2 OS=Mus musculus GN=Akap2
Prkar2b	P31324	12	12	126402.8	198468.9	210688.1	208192.8	220660.2	244237.2	219856.6	224755.8	272323.4	273480.8	0.033297	1.477595	192882.6	246930.8	Up	0.356384	0.731195	cAMP-dependent protein kinase type II-beta regulatory subunit OS=Mus musculus GN=Prkar2b PE=1 SV=3
Asph	Q8BSY0	1	1	NA	65382	54557	41963	83250	58533	31207	30559	13973	25427	0.040182	1.395968	61288	31939.8	Down	-0.94025	0.731195	Aspartyl/asparaginyl beta-hydroxylase OS=Mus musculus GN=Asph PE=2 SV=1
Ktn1	Q61595-5	5	5	30797.25	57011.25	52914.25	42382.75	72424.75	63468	67896	85363.5	70696.5	76657.75	0.033493	1.475046	51106.05	72816.35	Up	0.510768	0.731195	Isoform 5 of Kinectin OS=Mus musculus GN=Ktn1
Ppm1e	Q80TL0	2	2	226816.5	369233.5	353343	394420.5	288889.5	476883	408491.5	424528.5	400650.5	391566	0.033503	1.474916	326540.6	420423.9	Up	0.364582	0.731195	Protein phosphatase 1E OS=Mus musculus GN=Ppm1e PE=1 SV=2
Kifc2	O08672	1	1	75020	108172	92556	95020	78267	119515	130053	106966	114487	91283	0.033789	1.471225	89807	112460.8	Up	0.324522	0.731195	Kinesin-like protein KIFC2 OS=Mus musculus GN=Kifc2 PE=2 SV=1
Tpp2	Q64514	6	6	470142.7	465175.3	461708.7	454927.7	455383.7	433464	444970.3	366804	346060.7	288975	0.040993	1.38729	461467.6	376054.8	Down	-0.29529	0.731195	Tripeptidyl-peptidase 2 OS=Mus musculus GN=Tpp2 PE=1 SV=3
Abca2	P41234	1	1	NA	NA	4612	4873	6608	NA	NA	NA	8293	8581	0.033817	1.470865	5364.333	8437	Up	0.653331	0.731195	ATP-binding cassette sub-family A member 2 OS=Mus musculus GN=Abca2 PE=1 SV=4
Lgals1	P16045	1	1	179879	425295	375994	431250	517329	637726	449788	512121	558624	744087	0.03403	1.468138	385949.4	580469.2	Up	0.588808	0.731195	Galectin-1 OS=Mus musculus GN=Lgals1 PE=1 SV=3
Cnpy2	Q9QXT0	5	5	65159.5	78865.75	67256.25	53692.25	61993.5	65054.75	93647.25	94624.25	104526.3	77649.75	0.034102	1.46722	65393.45	87100.45	Up	0.413534	0.731195	Protein canopy homolog 2 OS=Mus musculus GN=Cnpy2 PE=1 SV=1
Vat1l	Q80TB8	2	2	612355.5	412645.5	496050	395845	392667	375452	359664.5	385754.5	327480	243665	0.042497	1.371642	461912.6	338403.2	Down	-0.44888	0.731195	Synaptic vesicle membrane protein VAT-1 homolog-like OS=Mus musculus GN=Vat1l PE=2 SV=2
Uqcrc2	Q9DB77	24	24	184093.2	108857.4	126631.2	143656.6	153095.6	116205.4	116974.5	96991.35	98571.71	105391.2	0.042738	1.369186	143266.8	106826.8	Down	-0.42343	0.731195	Cytochrome b-c1 complex subunit 2, mitochondrial OS=Mus musculus GN=Uqcrc2 PE=1 SV=1
Pebp1	P70296	12	12	28298.29	106741.7	88006.71	109871.6	83230.86	135675.9	116898.3	111032.9	157825.7	121316.4	0.034395	1.463505	83229.83	128549.8	Up	0.627155	0.731195	Phosphatidylethanolamine-binding protein 1 OS=Mus musculus GN=Pebp1 PE=1 SV=3
Vwa5a	Q99KC8	2	2	86544	178379	185838.5	156466	145718	183981	226274.5	221293	181429.5	209003.5	0.034818	1.458196	150589.1	204396.3	Up	0.440752	0.731195	von Willebrand factor A domain-containing protein 5A OS=Mus musculus GN=Vwa5a PE=1 SV=2
Parp14	Q2EMV9	1	1	194535	462193	388666	340766	395261	530804	484911	463006	505898	477309	0.036303	1.440057	356284.2	492385.6	Up	0.46676	0.731195	Poly [ADP-ribose] polymerase 14 OS=Mus musculus GN=Parp14 PE=1 SV=3
Nol6	Q8R5K4	1	1	NA	33500	40520	40221	35833	43972	39008	46621	59080	57253	0.036687	1.435488	37518.5	49186.8	Up	0.390669	0.731195	Nucleolar protein 6 OS=Mus musculus GN=Nol6 PE=2 SV=2
Mbp	P04370-9	23	23	991094.2	1007054	1667144	1482058	1087149	348949.5	935375.6	1054262	541705.7	1008209	0.044245	1.354136	1246900	777700.4	Down	-0.68106	0.731195	Isoform 9 of Myelin basic protein OS=Mus musculus GN=Mbp
Snx27	Q3UHD6	1	1	17502	18997	21131	12595	13929	28010	20378	21634	21407	19821	0.036834	1.433751	16830.8	22250	Up	0.402702	0.731195	Sorting nexin-27 OS=Mus musculus GN=Snx27 PE=1 SV=2
Ctnna2	Q61301-3	10	10	273464.6	238728.4	206452.3	260233.1	246102.4	216969.3	228410.6	179460.8	211960.6	218250.3	0.04472	1.349498	244996.2	211010.3	Down	-0.21545	0.731195	Isoform 3 of Catenin alpha-2 OS=Mus musculus GN=Ctnna2
Vat1	Q62465	6	6	412146	332660.7	378887.8	384765.7	416594	390774.2	318709	341070.5	312449.2	278397.8	0.04622	1.33517	385010.8	328280.1	Down	-0.22997	0.731195	Synaptic vesicle membrane protein VAT-1 homolog OS=Mus musculus GN=Vat1 PE=1 SV=3
Pfn2	Q9JJV2-2	2	2	94149	65992.5	77646	52043.5	79512.5	47073.5	72723	28676.5	60096.5	31341.5	0.046816	1.329606	73868.7	47982.2	Down	-0.62246	0.731195	Isoform 2 of Profilin-2 OS=Mus musculus GN=Pfn2
Nploc4	P60670	1	1	13158	44013	33002	34652	28866	37890	45952	41376	51555	53302	0.037264	1.428711	30738.2	46015	Up	0.582072	0.731195	Nuclear protein localization protein 4 homolog OS=Mus musculus GN=Nploc4 PE=1 SV=3
Cnksr2	Q80YA9	6	6	59056.2	75470.8	113705.2	112131.2	89754.8	90887	112185.2	167078.6	161289.2	178527.6	0.037586	1.424974	90023.64	141993.5	Up	0.657449	0.731195	Connector enhancer of kinase suppressor of ras 2 OS=Mus musculus GN=Cnksr2 PE=1 SV=1
Ppfia3	P60469	11	11	117685.4	187252.6	131246.4	154168.5	171257.3	201621.9	187414.4	215862.6	182076.1	168233.8	0.038193	1.418016	152322	191041.8	Up	0.326763	0.731195	Liprin-alpha-3 OS=Mus musculus GN=Ppfia3 PE=1 SV=1
Gprasp1	Q5U4C1	1	1	196489	209501	151181	165901	200037	157103	172655	140460	135521	160390	0.048563	1.313694	184621.8	153225.8	Down	-0.26891	0.731195	G-protein coupled receptor-associated sorting protein 1 OS=Mus musculus GN=Gprasp1 PE=1 SV=1
Grb2	Q60631	1	1	3707	12876	6159	9307	6975	10362	10712	13811	13553	13663	0.038206	1.417868	7804.8	12420.2	Up	0.670255	0.731195	Growth factor receptor-bound protein 2 OS=Mus musculus GN=Grb2 PE=1 SV=1
Fmnl1	Q9JL26	1	1	31041	35287	40941	50472	38421	41635	54968	45187	55980	51868	0.038285	1.416971	39232.4	49927.6	Up	0.347792	0.731195	Formin-like protein 1 OS=Mus musculus GN=Fmnl1 PE=1 SV=1
Pcbd1	P61458	1	1	51085	67438	75869	59746	42972	94451	62173	68592	88980	91688	0.038701	1.412278	59422	81176.8	Up	0.45007	0.731195	Pterin-4-alpha-carbinolamine dehydratase OS=Mus musculus GN=Pcbd1 PE=1 SV=2
Slc6a7	Q6PGE7	2	2	72371.5	118560	142150	147325.5	158664	181444.5	160727	177290	185200	160897.5	0.038833	1.410799	127814.2	173111.8	Up	0.437656	0.731195	Sodium-dependent proline transporter OS=Mus musculus GN=Slc6a7 PE=2 SV=1
Nosip	Q9D6T0	2	2	45215.5	70319.5	66910.5	75828	47349	82243	69480	78773	79548.5	87040.5	0.039673	1.401505	61124.5	79417	Up	0.377697	0.731195	Nitric oxide synthase-interacting protein OS=Mus musculus GN=Nosip PE=2 SV=1
Csrp1	P97315	4	4	46485	53988	41710.5	53280.75	49399.5	34900.75	41182	42825.25	43209.5	47455.25	0.049585	1.30465	48972.75	41914.55	Down	-0.22453	0.731195	Cysteine and glycine-rich protein 1 OS=Mus musculus GN=Csrp1 PE=1 SV=3
Hsp90aa1	P07901	5	5	1014103	1026321	930882.5	899119.5	941759.5	1097769	984178.5	1129620	987562.5	1096811	0.040251	1.395223	962437.1	1059188	Up	0.138195	0.731195	Heat shock protein HSP 90-alpha OS=Mus musculus GN=Hsp90aa1 PE=1 SV=4
Luc7l2	Q7TNC4-3	1	1	71684	80563	63655	54024	67735	81104	74013	88622	77788	107235	0.040282	1.394889	67532.2	85752.4	Up	0.344601	0.731195	Isoform 3 of Putative RNA-binding protein Luc7-like 2 OS=Mus musculus GN=Luc7l2

Coa3	Q9D2R6	1	1	157934	169464	96223	164611	166808	82733	97590	131082	130454	115275	0.049692	1.303714	151008	111426.8	Down	-0.43853	0.731195	Cytochrome c oxidase assembly factor 3 homolog, mitochondrial OS=Mus musculus GN=Coa3 PE=2 SV=1
Wasf1	Q8R5H6	10	10	66567.22	110327.6	78558.78	92947.67	94907.22	105445.8	97957.78	110567.3	135272.4	154632.8	0.040767	1.389691	88661.69	120775.2	Up	0.445942	0.731195	Wiskott-Aldrich syndrome protein family member 1 OS=Mus musculus GN=Wasf1 PE=1 SV=2
Ola1	Q9CZ30	4	4	259278.3	360674.8	282676.3	336145	226401.5	369096	348914	321193	372017.5	423694.8	0.041915	1.377631	293035.2	366983.1	Up	0.32464	0.731195	Obg-like ATPase 1 OS=Mus musculus GN=Ola1 PE=1 SV=1
Pkm	P52480-2	67	67	214545.7	288254	278231.8	265908.7	261766.3	282733.4	287891.6	289943.2	308552.1	331504.3	0.041922	1.377558	261741.3	300124.9	Up	0.197422	0.731195	Isoform M1 of Pyruvate kinase PKM OS=Mus musculus GN=Pkm
Pex5l	Q8C437	4	4	145269	249748	199054	193864	128066	227906	282398	190801	268552	296278	0.042292	1.373742	183200.2	253187	Up	0.466782	0.731195	PEX5-related protein OS=Mus musculus GN=Pex5l PE=1 SV=2
Kif5b	Q61768	8	8	28085.86	53939	51337.14	51027.57	51443.71	67031	57842	58120.57	60443.43	61061.14	0.043054	1.365986	47166.66	60899.63	Up	0.368666	0.731195	Kinesin-1 heavy chain OS=Mus musculus GN=Kif5b PE=1 SV=3
Mapt	P10637-4	16	16	83127.54	88469.31	73062.31	61493.54	73175.62	63461.31	52955.23	66591.46	59729.08	71147.46	0.051858	1.285184	75865.66	62776.91	Down	-0.27321	0.731195	Isoform Tau-C of Microtubule-associated protein tau OS=Mus musculus GN=Mapt
Dgkq	Q6P5E8	1	1	127766	68238	86217	62254	77996	30494	75972	65321	38069	46742	0.052241	1.281989	84494.2	51319.6	Down	-0.71934	0.731195	Diacylglycerol kinase theta OS=Mus musculus GN=Dgkq PE=2 SV=1
Klc2	O88448	6	6	91489.67	109176.2	108251.3	130034.8	115055.5	117358	136336.8	123096	128162.5	143624	0.043104	1.365482	110801.5	129715.5	Up	0.227373	0.731195	Kinesin light chain 2 OS=Mus musculus GN=Klc2 PE=1 SV=1
Rin1	Q921Q7	1	1	13978	23191	22397	21077	16380	21827	30258	33843	28521	20508	0.043765	1.358873	19404.6	26991.4	Up	0.476101	0.731195	Ras and Rab interactor 1 OS=Mus musculus GN=Rin1 PE=1 SV=1
Akt1s1	Q9D1F4	1	1	1282858	808352	889498	818655	921696	729011	802337	772228	660694	581687	0.052742	1.277843	944211.8	709191.4	Down	-0.41294	0.731195	Proline-rich AKT1 substrate 1 OS=Mus musculus GN=Akt1s1 PE=1 SV=1
Camkv	Q3UHL1	7	7	NA	4209	4141.5	4312	7016.5	3728.5	4411.5	2251	2161.5	1328.5	0.052949	1.276142	4919.75	2776.2	Down	-0.82547	0.731195	CaM kinase-like vesicle-associated protein OS=Mus musculus GN=Camkv PE=1 SV=2
Lsm12	Q9D0R8	3	3	82118	118856.7	74717.67	85774	78029	114703.3	101388	119029.7	96281.33	127695.7	0.043766	1.358863	87899.07	111819.6	Up	0.347253	0.731195	Protein LSM12 homolog OS=Mus musculus GN=Lsm12 PE=1 SV=1
Chchd3	Q9CRB9	5	5	12700.75	42209.75	24204	34308	26982.5	7538	9619.5	7156.25	10181.25	30530.75	0.053276	1.273468	28081	13005.15	Down	-1.11051	0.731195	Coiled-coil-helix-coiled-coil-helix domain-containing protein 3, mitochondrial OS=Mus musculus GN=Chchd3 PE=1 SV=1
Pdia6	Q922R8	5	5	55638	164075.3	76222	85123.5	154495.5	170243.8	151979.3	197891.3	131830.5	200564.5	0.04438	1.352813	107110.9	170501.9	Up	0.670683	0.731195	Protein disulfide-isomerase A6 OS=Mus musculus GN=Pdia6 PE=1 SV=3
Erc2	Q6PH08	9	9	161441	180558.1	173766.5	183031.1	157568.8	187755.3	172437.1	197078.3	183890.3	193234.9	0.047198	1.326076	171273.1	186879.2	Up	0.125807	0.731195	ERC protein 2 OS=Mus musculus GN=Erc2 PE=2 SV=2
Txnrd1	Q9JMH6	1	1	NA	91761	102520	94029	92270	95739	122272	123928	102498	107853	0.048218	1.316791	95145	110458	Up	0.215298	0.731195	Thioredoxin reductase 1, cytoplasmic OS=Mus musculus GN=Txnrd1 PE=1 SV=3
Igf2r	Q07113	2	2	11098.5	21075	27186	5612.5	10414	24898.5	19401	31917	23160	31993	0.04833	1.315783	15077.2	26273.9	Up	0.801262	0.731195	Cation-independent mannose-6-phosphate receptor OS=Mus musculus GN=Igf2r PE=1 SV=1
Mapre3	Q6PER3	3	3	270668.7	339646	403764	341274	361234	361892	431456.7	394030	448142.7	393242.7	0.048958	1.310176	343317.3	405752.8	Up	0.241058	0.731195	Microtubule-associated protein RP/EB family member 3 OS=Mus musculus GN=Mapre3 PE=1 SV=1
Usp5	P56399	7	7	29242.8	74624.8	59867.4	70343	48901.4	86694.4	74894.6	80823.6	67537.6	85052.8	0.049279	1.307338	56595.88	79000.6	Up	0.481167	0.731195	Ubiquitin carboxyl-terminal hydrolase 5 OS=Mus musculus GN=Usp5 PE=1 SV=1
Hspd1	P63038	56	56	1454050	1144888	1165146	1204979	1234883	1254774	1086827	1081377	905782.7	941839.5	0.055462	1.256004	1240789	1054120	Down	-0.23522	0.731195	60 kDa heat shock protein, mitochondrial OS=Mus musculus GN=Hspd1 PE=1 SV=1
Atp6v0a1	Q9Z1G4	9	9	58340.33	113755.8	126682.5	134323.5	108902.3	129951.7	149183.3	147976.8	145495.5	152157.2	0.049304	1.307118	108400.9	144952.9	Up	0.419207	0.731195	V-type proton ATPase 116 kDa subunit a isoform 1 OS=Mus musculus GN=Atp6v0a1 PE=1 SV=3
Synpo2	Q91YE8	2	2	23142	33132.5	33538	45316	28168.5	66652	28497.5	48228	53622.5	53050.5	0.049338	1.306818	32659.4	50010.1	Up	0.614721	0.731195	Synaptopodin-2 OS=Mus musculus GN=Synpo2 PE=1 SV=2
Tagln2	Q9WVA4	1	1	215946	403093	186673	139345	257226	356634	324773	328894	421098	386994	0.049422	1.30608	240456.6	363678.6	Up	0.596888	0.731195	Transgelin-2 OS=Mus musculus GN=Tagln2 PE=1 SV=4
Atp6v0a1	Q9Z1G4-3	10	10	50006	97648.29	108888.1	115404.6	93436.29	111635.3	127970.3	127161	125000.4	130640.7	0.049622	1.304326	93076.66	124481.5	Up	0.419441	0.731195	Isoform A1-III of V-type proton ATPase 116 kDa subunit a isoform 1 OS=Mus musculus GN=Atp6v0a1
Rps15a	P62245	1	1	49196	67756	82026	68784	38729	66432	79067	114219	100555	79398	0.049638	1.304186	61298.2	87934.2	Up	0.52058	0.731195	40S ribosomal protein S15a OS=Mus musculus GN=Rps15a PE=1 SV=2
Arhgap35	Q91YM2	2	2	81608.5	64591	79181	77466.5	70417.5	93364	85619.5	75738	86980	81096.5	0.050394	1.297621	74652.9	84559.6	Up	0.17977	0.731195	Rho GTPase-activating protein 35 OS=Mus musculus GN=Arhgap35 PE=1 SV=3
Hyou1	Q9JKR6	13	13	271227.8	377696.6	257417.6	320204.4	262604.9	384676.2	353396.3	339353.1	328065.1	455568.2	0.050601	1.295841	297830.2	372211.8	Up	0.321634	0.731195	Hypoxia up-regulated protein 1 OS=Mus musculus GN=Hyou1 PE=1 SV=1
Vdac1	Q60932	66	66	535069.9	729709.2	491947.8	751899.3	511962	792821.1	762070.8	709345.5	705922	814269.2	0.050986	1.292549	604117.6	756885.7	Up	0.325246	0.731195	Voltage-dependent anion-selective channel protein 1 OS=Mus musculus GN=Vdac1 PE=1 SV=3
Coro7	Q9D2V7	1	1	27015	20344	14912	14544	26441	19449	11721	14228	11400	10300	0.057279	1.242005	20651.2	13419.6	Down	-0.62188	0.731195	Coronin-7 OS=Mus musculus GN=Coro7 PE=2 SV=2
U2af2	P26369	1	1	14496	22051	23405	21280	17361	20313	32140	20952	33484	32111	0.051486	1.288311	19718.6	27800	Up	0.495528	0.731195	Splicing factor U2AF 65 kDa subunit OS=Mus musculus GN=U2af2 PE=1 SV=3
Rab3gap2	Q8BMG7	1	1	32933	117414	133385	131388	106723	128263	170302	139191	169648	165342	0.051782	1.285821	104368.6	154549.2	Up	0.566378	0.731195	Rab3 GTPase-activating protein non-catalytic subunit OS=Mus musculus GN=Rab3gap2 PE=1 SV=2
Pcmt1	P23506	6	6	504746.8	515846.2	371680.2	473680.4	440103.2	430318.6	379416.4	352439.6	383350	416599.4	0.057471	1.240551	461211.4	392424.8	Down	-0.23301	0.731195	Protein-L-isoaspartate(D-aspartate) O-methyltransferase OS=Mus musculus GN=Pcmt1 PE=1 SV=3
Mbp	P04370-13	17	17	1179611	1313592	2174476	1902463	1392133	440019.1	1210045	1366231	721422.9	1306092	0.057483	1.240461	1592455	1008762	Down	-0.65867	0.731195	Isoform 12 of Myelin basic protein OS=Mus musculus GN=Mbp
Oxr1	Q4KMM3	13	13	41409.8	50705.8	33955.8	53213.8	35937	47309.5	54426.5	47020.6	67725	64461.7	0.05252	1.279675	43044.44	56188.66	Up	0.384452	0.731195	Oxidation resistance protein 1 OS=Mus musculus GN=Oxr1 PE=2 SV=3
Nlgn4l	B0F2B4	2	2	41895	51342	51741	48034.5	47964.5	58437	78386.5	82536	55829	51144	0.052649	1.27861	48195.4	65266.5	Up	0.437447	0.731195	Neuroligin 4-like OS=Mus musculus GN=Nlgn4l PE=1 SV=1
Prrc2c	Q3TLH4	2	2	95484.5	143898	95216	117353	119811	147714.5	128790.5	154291.5	121478	146570	0.053233	1.273819	114352.5	139768.9	Up	0.289555	0.731195	Protein PRRC2C OS=Mus musculus GN=Prrc2c PE=1 SV=3
Ccdc177	Q3UHB8	4	4	309892.3	164079	165693.3	150534.3	211150.3	148993	118145.7	138573	102023	115269.7	0.059028	1.228942	200269.9	124600.9	Down	-0.68463	0.731195	Coiled-coil domain-containing protein 177 OS=Mus musculus GN=Ccdc177 PE=1 SV=1
Dnpep	Q9Z2W0	3	3	31461	54335	55242.33	52753.33	36675	51436.33	60132	57662.33	64847.67	63482.33	0.053406	1.27241	46093.33	59512.13	Up	0.368626	0.731195	Aspartyl aminopeptidase OS=Mus musculus GN=Dnpep PE=2 SV=2
Dnajb6	O54946-2	1	1	476309	416176	339501	390006	405847	417707	502378	463104	455619	501749	0.053437	1.272158	405567.8	468111.4	Up	0.206909	0.731195	Isoform B of DnaJ homolog subfamily B member 6 OS=Mus musculus GN=Dnajb6
Prrt1	O35449	3	3	87773.5	75658	95468	100754	105051	120977	106033	125790	119062	89004.5	0.054028	1.267381	92940.9	112173.3	Up	0.271344	0.731195	Proline-rich transmembrane protein 1 OS=Mus musculus GN=Prrt1 PE=1 SV=1
Osbpl11	Q8CI95	1	1	NA	38493	38773	29210	34454	45891	36276	63788	44798	45810	0.054557	1.26315	35232.5	47312.6	Up	0.425318	0.731195	Oxysterol-binding protein-related protein 11 OS=Mus musculus GN=Osbpl11 PE=1 SV=2
Gapdhs	Q64467	10	10	90883.7	108539.8	122783.1	126838.8	120636.3	115551.3	143563.8	133680	148492.1	127183.9	0.054671	1.262243	113936.3	133694.2	Up	0.230709	0.731195	Glyceraldehyde-3-phosphate dehydrogenase, testis-specific OS=Mus musculus GN=Gapdhs PE=2 SV=1
Slc25a31	Q3V132	1	1	NA	26696	49893	35413	37383	42151	74931	94240	43026	64249	0.055384	1.256616	37346.25	63719.4	Up	0.770769	0.731195	ADP/ATP translocase 4 OS=Mus musculus GN=Slc25a31 PE=2 SV=1
Got2	P05202	24	24	810030.5	654426.5	585781	767250.3	633961	658479.8	552063.9	553922	541143.3	598900.6	0.060945	1.215062	690289.8	580901.9	Down	-0.24891	0.731195	Aspartate aminotransferase, mitochondrial OS=Mus musculus GN=Got2 PE=1 SV=1
Gfm2	Q8R2Q4-3	1	1	168458	358664	341360	348851	234618	476324	372194	433469	329545	366645	0.055473	1.255918	290390.2	395635.4	Up	0.446179	0.731195	Isoform 3 of Ribosome-releasing factor 2, mitochondrial OS=Mus musculus GN=Gfm2
Nfasc	Q810U3	15	15	43765.43	73060.07	65000.29	74574.57	62984.5	69300.36	86421.64	67365.36	88161.93	94749.71	0.055552	1.2553	63876.97	81199.8	Up	0.34618	0.731195	Neurofascin OS=Mus musculus GN=Nfasc PE=1 SV=1
Erc2	Q6PH08-3	8	8	168105.9	190834.9	185110.3	196290.7	166641.1	201163.4	181859.4	212162.6	194866.3	205445.4	0.056183	1.250395	181396.6	199099.4	Up	0.134342	0.731195	Isoform 3 of ERC protein 2 OS=Mus musculus GN=Erc2
Puf60	Q3UEB3-3	1	1	42246	77614	47827	92557	61153	90590	87619	64111	110069	110253	0.056288	1.249584	64279.4	92528.4	Up	0.52554	0.731195	Isoform 3 of Poly(U)-binding-splicing factor PUF60 OS=Mus musculus GN=Puf60
Txlna	Q6PAM1	1	1	NA	69705	80160	53688	62485	87736	74375	75297	84702	90899	0.056435	1.248451	66509.5	82601.8	Up	0.312613	0.731195	Alpha-taxilin OS=Mus musculus GN=Txlna PE=2 SV=1
Slc6a17	Q8BJI1	1	1	73144	139115	149369	117533	167566	227231	151879	133881	211085	280967	0.056814	1.245545	129345.4	201008.6	Up	0.636028	0.731195	Sodium-dependent neutral amino acid transporter SLC6A17 OS=Mus musculus GN=Slc6a17 PE=1 SV=1
Nde1	Q9CZA6-3	2	2	207494.5	155429.5	150728.5	132911	177894.5	159882	139070.5	128330	114547.5	110484.5	0.062899	1.201356	164891.6	130462.9	Down	-0.33788	0.731195	Isoform 3 of Nuclear distribution protein nudE homolog 1 OS=Mus musculus GN=Nde1
Trim3	Q9R1R2	1	1	53594	96167	75880	99030	57700	68197	157387	112842	116753	121328	0.05704	1.24382	76474.2	115301.4	Up	0.592365	0.731195	Tripartite motif-containing protein 3 OS=Mus musculus GN=Trim3 PE=1 SV=1
Acox1	Q9R0H0	1	1	37416	49692	50442	47759	41037	63440	54737	53234	54755	89484	0.057061	1.243661	45269.2	63130	Up	0.479796	0.731195	Peroxisomal acyl-coenzyme A oxidase 1 OS=Mus musculus GN=Acox1 PE=1 SV=5
Mapt	P10637-5	31	31	264363.2	221853.4	164183	185854.5	214578.2	186542.2	158801.3	163742.8	149935.9	176973.6	0.063556	1.196843	210166.5	167199.2	Down	-0.32996	0.731195	Isoform Tau-D of Microtubule-associated protein tau OS=Mus musculus GN=Mapt
Chmp3	Q9CQ10	1	1	212691	267592	257750	245116	244265	299381	313482	293810	232881	277593	0.057311	1.241762	245482.8	283429.4	Up	0.207367	0.731195	Charged multivesicular body protein 3 OS=Mus musculus GN=Chmp3 PE=1 SV=3
Ola1	Q9CZ30-2	3	3	324754.7	398571.3	315385.3	374467	247655	433792.3	388352.7	345629.3	412717	434580	0.057263	1.241368	332166.7	403014.3	Up	0.278924	0.731195	Isoform 2 of Obg-like ATPase 1 OS=Mus musculus GN=Ola1
Tkt	P40142	18	18	470157.2	298598.7	310609.9	328860	309417	284210.8	280513.4	275784.2	246362.6	228439.1	0.064179	1.192607	343528.6	263062	Down	-0.38503	0.731195	Transketolase OS=Mus musculus GN=Tkt PE=1 SV=1
Ube2q1	Q7TSS2	2	2	96821	118989.5	107979	91525	87922	115912	112025	116936.5	112965	119913.5	0.057506	1.240287	100647.3	115550.4	Up	0.199214	0.731195	Ubiquitin-conjugating enzyme E2 Q1 OS=Mus musculus GN=Ube2q1 PE=2 SV=2
Agtpbp1	Q641K1-3	2	2	296844.5	266598.5	282484.5	313601	365747.5	305359.5	259258.5	267980	227614.5	201615.5	0.064615	1.189667	305055.2	252365.6	Down	-0.27356	0.731195	Isoform 3 of Cytosolic carboxypeptidase 1 OS=Mus musculus GN=Agtpbp1
Ndel1	Q9ERR1	3	3	138329.7	103619.7	101120.7	89476.33	118596.3	108293	92713.67	85553.33	77544.33	74210	0.064802	1.188412	110228.5	87662.87	Down	-0.33046	0.731195	Nuclear distribution protein nudE-like 1 OS=Mus musculus GN=Ndel1 PE=1 SV=2
Cd200	O54901	3	3	107891.7	62701	83844.33	65551	100082.3	72183.33	70485.67	66374.67	52662.67	29786.33	0.064849	1.188097	84014.07	58298.53	Down	-0.52717	0.731195	OX-2 membrane glycoprotein OS=Mus musculus GN=Cd200 PE=1 SV=1
Myl6b	Q8CI43	2	2	177205.5	132349	118042	116061	139274	114860.5	137626	95615	94313.5	84829.5	0.065021	1.186946	136586.3	105448.9	Down	-0.37327	0.731195	Myosin light chain 6B OS=Mus musculus GN=Myl6b PE=2 SV=1
Cryz	P47199	3	3	328311.7	517564	360620.3	414972	388035.7	479799	404492.3	546042	493043	555887.3	0.057527	1.240128	401900.7	495852.7	Up	0.303072	0.731195	Quinone oxidoreductase OS=Mus musculus GN=Cryz PE=1 SV=1
Atp5k	Q06185	7	7	166408.7	110544.3	149641	138361	151588.9	136228	124179.7	127281	104921	90587.71	0.065393	1.184469	143308.8	116639.5	Down	-0.29707	0.731195	ATP synthase subunit e, mitochondrial OS=Mus musculus GN=Atp5k PE=1 SV=2
Psmc1	P62192	3	3	24167.67	20623.67	20508.67	18795.67	16177	21189.33	22781.67	23023.33	25638.33	25249.67	0.057967	1.236819	20054.54	23576.47	Up	0.233419	0.731195	26S protease regulatory subunit 4 OS=Mus musculus GN=Psmc1 PE=1 SV=1
Dnm1l	Q8K1M6-5	8	8	10092.38	36894.25	21331	32459.12	24035.88	34360.12	33277.38	33395	37074.75	50112.62	0.05911	1.228339	24962.53	37643.97	Up	0.592655	0.731195	Isoform 5 of Dynamin-1-like protein OS=Mus musculus GN=Dnm1l
Ppt1	O88531	3	3	153650.3	262391	156607	194704	122134.7	207729.3	252253.3	238138.3	248960.3	252273.7	0.0596	1.224754	177897.4	239871	Up	0.431213	0.731195	Palmitoyl-protein thioesterase 1 OS=Mus musculus GN=Ppt1 PE=2 SV=2
Dlgap4	B1AZP2-3	3	3	43230.33	41944	54545.33	43255.67	36129.67	61122.67	48642	45635	52443.67	55369.67	0.059763	1.223568	43821	52642.6	Up	0.264608	0.731195	Isoform 3 of Disks large-associated protein 4 OS=Mus musculus GN=Dlgap4
Glcci1	Q8K3I9-3	2	2	108419	160322	126958	122451.5	126694.5	157882.5	156302.5	152013	131261.5	195617	0.060038	1.221574	128969	158615.3	Up	0.298508	0.731195	Isoform 3 of Glucocorticoid-induced transcript 1 protein OS=Mus musculus GN=Glcci1
Gpd1	P13707	9	9	112630.3	356549.4	276066.3	322999.6	264866.8	418875.4	385600.6	340052.1	343544.2	382178.8	0.060362	1.219236	266622.5	374050.2	Up	0.488434	0.731195	Glycerol-3-phosphate dehydrogenase [NAD(+)], cytoplasmic OS=Mus musculus GN=Gpd1 PE=1 SV=3
Tmcc2	Q80W04	2	2	281603.5	397834.5	374273	384947.5	341555	506505.5	409780.5	487252	366913	390180.5	0.060674	1.216997	356042.7	432126.3	Up	0.279403	0.731195	Transmembrane and coiled-coil domains protein 2 OS=Mus musculus GN=Tmcc2 PE=1 SV=1
Atad3a	Q925I1-2	3	3	129232.5	93547	123877.5	116312	113211.5	106461.5	111125	101059.5	87086.5	76443.5	0.066775	1.175386	115236.1	96435.2	Down	-0.25696	0.731195	Isoform 2 of ATPase family AAA domain-containing protein 3 OS=Mus musculus GN=Atad3
Acaa2	Q8BWT1	11	11	268232.8	203600.1	243777.7	235117.3	245910.6	251694.6	210061.2	193819.4	179376.3	165758.5	0.066915	1.174477	239327.7	200142	Down	-0.25796	0.731195	3-ketoacyl-CoA thiolase, mitochondrial OS=Mus musculus GN=Acaa2 PE=1 SV=3
Pfkm	P47857	15	15	365552.5	305245.7	327541.7	325120.9	288418.2	280139.5	323021.7	281120.1	286150.5	271817.2	0.06723	1.172437	322375.8	288449.8	Down	-0.16042	0.731195	6-phosphofructokinase, muscle type OS=Mus musculus GN=Pfkm PE=1 SV=3
Ncs1	Q8BNY6	1	1	NA	11466	8907	7857	6740	14041	10915	9603	10507	15303	0.061021	1.214521	8742.5	12073.8	Up	0.465762	0.731195	Neuronal calcium sensor 1 OS=Mus musculus GN=Ncs1 PE=2 SV=3
Srgap1	Q91Z69	1	1	20737	22413	33770	27731	25959	31567	34902	31876	29058	32233	0.061372	1.21203	26122	31947.2	Up	0.290424	0.731195	SLIT-ROBO Rho GTPase-activating protein 1 OS=Mus musculus GN=Srgap1 PE=1 SV=2
Ddx3x	Q62167	7	7	134528.6	136640.1	151869.6	138432	176976.1	160481.6	175458.1	187489	166691.9	155841	0.061496	1.211153	147689.3	169192.3	Up	0.196099	0.731195	ATP-dependent RNA helicase DDX3X OS=Mus musculus GN=Ddx3x PE=1 SV=3
Add2	Q9QYB8	7	7	56994	27930.8	42898	24602	49940.4	16888	26546.6	27350.8	25901.8	28837	0.067843	1.168495	40473.04	25104.84	Down	-0.689	0.731195	Beta-adducin OS=Mus musculus GN=Add2 PE=1 SV=4
Atp6v1b2	P62814	52	52	1336328	847999.1	987157.9	827029	988014.9	862480.2	800328.9	788563.2	767252.4	641859.1	0.067928	1.167951	997305.8	772096.7	Down	-0.36925	0.731195	V-type proton ATPase subunit B, brain isoform OS=Mus musculus GN=Atp6v1b2 PE=1 SV=1
Elavl4	Q61701	2	2	62129	59626	73596.5	65823	70566	65833.5	83953.5	70680	77143	79020.5	0.061503	1.211104	66348.1	75326.1	Up	0.183095	0.731195	ELAV-like protein 4 OS=Mus musculus GN=Elavl4 PE=1 SV=1
Grm5	Q3UVX5	1	1	149279	244955	318559	290855	269345	448926	309948	324263	309926	310165	0.062515	1.204016	254598.6	340645.6	Up	0.420047	0.731195	Metabotropic glutamate receptor 5 OS=Mus musculus GN=Grm5 PE=1 SV=2
Nme1	P15532	6	6	554905.7	371050	450527.3	422819.3	429340.3	511915.7	498841.8	563861.2	552150	489063.2	0.062599	1.203433	445728.5	523166.4	Up	0.231104	0.731195	Nucleoside diphosphate kinase A OS=Mus musculus GN=Nme1 PE=1 SV=1
Cat	P24270	2	2	98318	100929.5	116654	119192.5	121815	138766.5	107469.5	139420	130069	116607.5	0.063016	1.200549	110981.8	126466.5	Up	0.207485	0.731195	Catalase OS=Mus musculus GN=Cat PE=1 SV=4
Fgf1	P61148	1	1	163971	126296	129830	113027	130425	122890	118458	121959	83730	93496	0.068953	1.161447	132709.8	108106.6	Down	-0.29582	0.731195	Fibroblast growth factor 1 OS=Mus musculus GN=Fgf1 PE=2 SV=1
Dlat	Q8BMF4	19	19	686033.2	453508.4	465451.4	500684.1	570968.3	537903.7	431654.1	441845.7	332845.1	302151.1	0.069149	1.160214	535329.1	409279.9	Down	-0.38734	0.731195	Dihydrolipoyllysine-residue acetyltransferase component of pyruvate dehydrogenase complex, mitochondrial OS=Mus musculus GN=Dlat PE=1 SV=2
Htatip2	Q9Z2G9	2	2	47893	49813.5	43098.5	53231.5	50628.5	61759.5	60259	45540	62938.5	55120	0.063125	1.199799	48933	57123.4	Up	0.223274	0.731195	Oxidoreductase HTATIP2 OS=Mus musculus GN=Htatip2 PE=1 SV=3
Cnn3	Q9DAW9	5	5	105351.3	97129.5	76874	94949	75506	76203.5	86103.25	76775.75	74129.25	57974.25	0.069842	1.155883	89961.95	74237.2	Down	-0.27717	0.733632	Calponin-3 OS=Mus musculus GN=Cnn3 PE=1 SV=1
Anxa2	P07356	1	1	NA	4286	3978	8517	10903	6544	11780	13477	14812	12661	0.063705	1.195826	6921	11854.8	Up	0.776419	0.731195	Annexin A2 OS=Mus musculus GN=Anxa2 PE=1 SV=2
Park7	Q99LX0	9	9	1116280	831969.7	874442.4	693268.9	884764.9	845161.8	715587.8	787395.4	542602.8	560503.1	0.070996	1.148766	880145.1	690250.2	Down	-0.35062	0.734831	Protein DJ-1 OS=Mus musculus GN=Park7 PE=1 SV=1
Hnrnpll	Q921F4	2	2	199885	197175	225113	116863	235607	299640	218370	287348	219947	233269	0.063727	1.195727	193128.6	251714.8	Up	0.382228	0.731195	Heterogeneous nuclear ribonucleoprotein L-like OS=Mus musculus GN=Hnrnpll PE=1 SV=3
Ddx3y	Q62095	6	6	154606.8	154776.8	174181.7	157744	202414	182363.8	200679	215621.3	190516.7	178150.3	0.064493	1.190487	168744.7	193466.2	Up	0.19724	0.731195	ATP-dependent RNA helicase DDX3Y OS=Mus musculus GN=Ddx3y PE=1 SV=2
Stx5a	Q8K1E0	2	2	188360	173767	121213	125305	206510	122628.5	114193	118133.5	131370.5	122043	0.071653	1.144766	163031	121673.7	Down	-0.42213	0.734831	Syntaxin-5 OS=Mus musculus GN=Stx5 PE=1 SV=3
Ctnnd1	P30999-3	6	6	21239.33	67311.5	59843.83	69736	60609	72555	77599.5	79867.5	74079	85184.67	0.065093	1.186466	55747.93	77857.13	Up	0.481911	0.731195	Isoform 3 of Catenin delta-1 OS=Mus musculus GN=Ctnnd1
Eif4g3	Q80XI3	2	2	679798	646809	599542	600450.5	656884.5	793856.5	697489.5	796463.5	709703	613836	0.065511	1.183686	636696.8	722269.7	Up	0.181931	0.731195	Eukaryotic translation initiation factor 4 gamma 3 OS=Mus musculus GN=Eif4g3 PE=1 SV=2
Eif4g3	Q80XI3-4	3	3	679798	646809	599542	600450.5	656884.5	793856.5	697489.5	796463.5	709703	613836	0.065511	1.183686	636696.8	722269.7	Up	0.181931	0.731195	Isoform 4 of Eukaryotic translation initiation factor 4 gamma 3 OS=Mus musculus GN=Eif4g3
Oxr1	Q4KMM3-3	8	8	18426.83	45337.67	26601.5	55349.17	21902.83	39747.83	50950.33	40975.17	77930	71269.5	0.065795	1.181807	33523.6	56174.57	Up	0.74474	0.731195	Isoform 3 of Oxidation resistance protein 1 OS=Mus musculus GN=Oxr1
Cyld	Q80TQ2	1	1	201971	408732	276775	366439	282806	480222	371189	331515	380928	475987	0.065998	1.180469	307344.6	407968.2	Up	0.4086	0.731195	Ubiquitin carboxyl-terminal hydrolase CYLD OS=Mus musculus GN=Cyld PE=1 SV=2
Lysmd1	Q9D0E3	1	1	NA	10663	12004	11064	10784	14922	11389	14960	10983	14374	0.066053	1.180107	11128.75	13325.6	Up	0.259909	0.731195	LysM and putative peptidoglycan-binding domain-containing protein 1 OS=Mus musculus GN=Lysmd1 PE=2 SV=1
Cacnb1	Q8R3Z5-3	2	2	130703	206012	210377	200903.5	189777	218965	256602.5	233306.5	263589	183325.5	0.06627	1.178683	187554.5	231157.7	Up	0.301568	0.731195	Isoform 3 of Voltage-dependent L-type calcium channel subunit beta-1 OS=Mus musculus GN=Cacnb1
Ftl2-ps	P49945	1	1	812590	1183961	1081293	1010888	1380418	1164476	1337431	1483432	1213473	1682903	0.066701	1.175868	1093830	1376343	Up	0.331452	0.731195	Ferritin light chain 2 OS=Mus musculus GN=Ftl2 PE=2 SV=2
Ddah2	Q99LD8	5	5	111775.5	87513	99062.25	95572.5	123239.3	83739.75	77599	112259.8	58689.5	71728.5	0.075328	1.123044	103432.5	80803.3	Down	-0.3562	0.738751	N(G),N(G)-dimethylarginine dimethylaminohydrolase 2 OS=Mus musculus GN=Ddah2 PE=1 SV=1
Pcbp2	Q61990-2	8	8	410888.3	380282	382276	354605.8	350484.7	379729.7	437442.8	427367.8	435271.7	380439.2	0.067392	1.171392	375707.4	412050.2	Up	0.133211	0.731195	Isoform 2 of Poly(rC)-binding protein 2 OS=Mus musculus GN=Pcbp2
Fasn	P19096	6	6	83188.33	160764.2	148813.7	182410.8	160164.2	163802.3	205442.2	179865.8	189413.3	207055.2	0.06764	1.169796	147068.2	189115.8	Up	0.362784	0.731195	Fatty acid synthase OS=Mus musculus GN=Fasn PE=1 SV=2
Prkar2a	P12367	5	5	94382.2	170510.4	199766.4	209949.6	177884.2	193270	181862	232008.4	256306.2	275671.2	0.067832	1.168565	170498.6	227823.6	Up	0.418157	0.731195	cAMP-dependent protein kinase type II-alpha regulatory subunit OS=Mus musculus GN=Prkar2a PE=1 SV=2
Arsb	P50429	2	2	25775	64870.5	55448.5	50658.5	42221.5	71222	57116.5	69285.5	55797.5	67907	0.068006	1.167453	47794.8	64265.7	Up	0.427195	0.731195	Arylsulfatase B OS=Mus musculus GN=Arsb PE=2 SV=3
Uba1y	P31254	4	4	230382.5	276160.3	236734.3	260251.5	243630	307962	272509.8	279732	249261.5	270819.3	0.068211	1.166146	249431.7	276056.9	Up	0.146321	0.731195	Ubiquitin-like modifier-activating enzyme 1 Y OS=Mus musculus GN=Uba1y PE=2 SV=2
Stmn1	P54227	9	9	849609.4	586183.1	502365.8	552486.8	647659.1	621874.4	471909.3	462443.9	428602.8	417395.3	0.077945	1.108212	627660.8	480445.2	Down	-0.38561	0.738751	Stathmin OS=Mus musculus GN=Stmn1 PE=1 SV=2
Xpnpep1	Q6P1B1	1	1	756993	698834	667222	742788	753157	707959	773253	814766	783657	822411	0.068665	1.163265	723798.8	780409.2	Up	0.108642	0.731195	Xaa-Pro aminopeptidase 1 OS=Mus musculus GN=Xpnpep1 PE=2 SV=1
Mcee	Q9D1I5	3	3	2569667	2682543	2178080	2698160	2246639	2307767	2285352	2361605	1835751	2109580	0.078563	1.104782	2475018	2180011	Down	-0.1831	0.738751	Methylmalonyl-CoA epimerase, mitochondrial OS=Mus musculus GN=Mcee PE=1 SV=1
Slc4a1	P04919	2	2	157951	364089.5	350042.5	282993.5	345964.5	329956.5	360770.5	385038.5	459395	567890	0.068837	1.162178	300208.2	420610.1	Up	0.48652	0.731195	Band 3 anion transport protein OS=Mus musculus GN=Slc4a1 PE=1 SV=1
Tigar	Q8BZA9	2	2	NA	64590	63117.5	92762	117219.5	38975	60670	34772.5	52186.5	72231.5	0.079447	1.099922	84422.25	51767.1	Down	-0.70559	0.738751	Fructose-2,6-bisphosphatase TIGAR OS=Mus musculus GN=Tigar PE=1 SV=1
D1Pas1	P16381	5	5	184028.8	182435.4	206171.4	187796.8	240516.2	217423.2	238187	255111.2	224610.6	209787.2	0.069325	1.15911	200189.7	229023.8	Up	0.19413	0.731195	Putative ATP-dependent RNA helicase Pl10 OS=Mus musculus GN=D1Pas1 PE=2 SV=1
Mapk1	P63085	4	4	NA	179131.5	128701.5	137696	70271	209950.5	184670.5	220251.5	145612.5	181667	0.070935	1.149139	128950	188430.4	Up	0.54722	0.734831	Mitogen-activated protein kinase 1 OS=Mus musculus GN=Mapk1 PE=1 SV=3
Adgrl3	Q80TS3-3	3	3	10197.5	19516.5	30071.5	28289.5	19700.5	27911	35017	32028	30920	25517	0.071074	1.148289	21555.1	30278.6	Up	0.490269	0.734831	Isoform 3 of Latrophilin-3 OS=Mus musculus GN=Lphn3
Anln	Q8K298	1	1	544248	207054	391140	302531	380005	330126	252020	224502	172005	191225	0.080572	1.093816	364995.6	233975.6	Down	-0.64152	0.738751	Actin-binding protein anillin OS=Mus musculus GN=Anln PE=1 SV=2
Slc4a10	Q5DTL9	2	2	693048.5	884939.5	730083	836618	742382.5	849573.5	809539.5	838365	934444.5	1057937	0.071339	1.146673	777414.3	897971.8	Up	0.207986	0.734831	Sodium-driven chloride bicarbonate exchanger OS=Mus musculus GN=Slc4a10 PE=1 SV=2
Aldh3a2	P47740	1	1	NA	120054	127157	120311	129971	125556	139884	135759	126734	150042	0.071677	1.14462	124373.3	135595	Up	0.124628	0.734831	Fatty aldehyde dehydrogenase OS=Mus musculus GN=Aldh3a2 PE=2 SV=2
Pld3	O35405	4	4	74609	43715.33	47121.67	60601.33	60001.67	49661	45579.33	46204.33	39732	41429.67	0.081679	1.08789	57209.8	44521.27	Down	-0.36177	0.738751	Phospholipase D3 OS=Mus musculus GN=Pld3 PE=2 SV=1
Actr1b	Q8R5C5	5	5	153382.2	184640	200715.6	165935.8	197707	155792.6	178154.6	153436.2	154921.6	152549.8	0.082054	1.0859	180476.1	158971	Down	-0.18304	0.738751	Beta-centractin OS=Mus musculus GN=Actr1b PE=1 SV=1
Psap	Q61207	2	2	64856	37324	43002	49307.5	59921	66113.5	67485.5	72111	55005	57905	0.072427	1.1401	50882.1	63724	Up	0.324679	0.738024	Sulfated glycoprotein 1 OS=Mus musculus GN=Psap PE=1 SV=2
Pygb	Q8CI94	5	5	52611.6	85991.2	100599	91467.6	87601.2	86522.6	103160.4	117896.4	100450.4	114320.4	0.072716	1.13837	83654.12	104470	Up	0.320581	0.738024	Glycogen phosphorylase, brain form OS=Mus musculus GN=Pygb PE=1 SV=3
Eea1	Q8BL66	9	9	51018.5	137081	63421.17	86495	69753.5	110177.3	98007.17	111131.3	130709	141414	0.072852	1.137559	81553.83	118287.8	Up	0.536476	0.738024	Early endosome antigen 1 OS=Mus musculus GN=Eea1 PE=1 SV=2
Tfe3	Q64092	1	1	NA	NA	59859	34325	81314	NA	13530	12019	16666	16992	0.083383	1.078922	58499.33	14801.75	Down	-1.98265	0.738751	Transcription factor E3 OS=Mus musculus GN=Tfe3 PE=1 SV=2
Abcb7	Q61102	1	1	NA	945	1004	475	576	849	2102	716	1566	1786	0.073633	1.132928	750	1403.8	Up	0.904375	0.738751	ATP-binding cassette sub-family B member 7, mitochondrial OS=Mus musculus GN=Abcb7 PE=1 SV=3
Syn2	Q64332-2	19	19	159245.5	208940.5	162766.1	183287	164684.6	195128.1	185317.3	198438.4	202453.2	209097.6	0.07393	1.131179	175784.7	198086.9	Up	0.172324	0.738751	Isoform IIb of Synapsin-2 OS=Mus musculus GN=Syn2
Cfl2	P45591	8	8	66367.62	47685.25	50819	43028.5	55739	46096.75	48257.62	41858.88	45975	33027.25	0.084324	1.074049	52727.87	43043.1	Down	-0.29278	0.738751	Cofilin-2 OS=Mus musculus GN=Cfl2 PE=1 SV=1
Pkm	P52480	66	66	187571.6	272858.2	260037	255728.4	252162	268822.8	270695.8	269606.1	288124.5	315186.1	0.07502	1.124823	245671.4	282487.1	Up	0.201455	0.738751	Pyruvate kinase PKM OS=Mus musculus GN=Pkm PE=1 SV=4
Hnrnph2	P70333	6	6	149632	346101	251833.3	274381	441124.8	288095.8	397787	452172.3	471576	476437.8	0.075059	1.124597	292614.4	417213.8	Up	0.511786	0.738751	Heterogeneous nuclear ribonucleoprotein H2 OS=Mus musculus GN=Hnrnph2 PE=2 SV=1
Arhgef12	Q8R4H2	2	2	466151.5	361800.5	399348.5	328647.5	354431	369380	340960	316814.5	309579	294478.5	0.084696	1.072137	382075.8	326242.4	Down	-0.22791	0.738751	Rho guanine nucleotide exchange factor 12 OS=Mus musculus GN=Arhgef12 PE=1 SV=2
Mark3	Q03141	6	6	98610.17	61478.5	82976.67	60499.83	79411	74943.5	62695.5	42312.5	63347	48751.5	0.084787	1.071671	76595.23	58410	Down	-0.39104	0.738751	MAP/microtubule affinity-regulating kinase 3 OS=Mus musculus GN=Mark3 PE=1 SV=2
Mark3	Q03141-3	6	6	98610.17	61478.5	82976.67	60499.83	79411	74943.5	62695.5	42312.5	63347	48751.5	0.084787	1.071671	76595.23	58410	Down	-0.39104	0.738751	Isoform 3 of MAP/microtubule affinity-regulating kinase 3 OS=Mus musculus GN=Mark3
Mtpn	P62774	1	1	192598	247791	139846	177135	83244	281751	206163	234814	237340	210525	0.075355	1.122888	168122.8	234118.6	Up	0.477724	0.738751	Myotrophin OS=Mus musculus GN=Mtpn PE=1 SV=2
Fxr1	Q61584-3	2	2	9956.5	27319.5	23798	21128	18361.5	24092	26916.5	25841	26162	33042.5	0.075767	1.12052	20112.7	27210.8	Up	0.436073	0.738751	Isoform B of Fragile X mental retardation syndrome-related protein 1 OS=Mus musculus GN=Fxr1
Ndufa13	F6W8I0	3	3	12648.5	29336.5	23794.5	28912	24399.5	27686	36123.5	29771	30814	30643.5	0.076398	1.116918	23818.2	31007.6	Up	0.380557	0.738751	YjeF N-terminal domain-containing protein 3 OS=Mus musculus GN=Yjefn3 PE=2 SV=2
Matk	P41242-2	1	1	376777	415303	388583	356284	323118	436936	418243	465320	376287	389433	0.077281	1.111927	372013	417243.8	Up	0.165538	0.738751	Isoform 2 of Megakaryocyte-associated tyrosine-protein kinase OS=Mus musculus GN=Matk
Tomm34	Q9CYG7	2	2	244511.5	240531.5	301656.5	243862.5	231634	267354.5	302237.5	260481	281567.5	327211	0.077682	1.10968	252439.2	287770.3	Up	0.188982	0.738751	Mitochondrial import receptor subunit TOM34 OS=Mus musculus GN=Tomm34 PE=2 SV=1
BC003331	Q4PJX1	1	1	84488	190290	224402	232381	124400	276301	235913	226301	222588	233087	0.078059	1.107577	171192.2	238838	Up	0.480415	0.738751	Protein odr-4 homolog OS=Mus musculus GN=Odr4 PE=2 SV=2
Rplp2	P99027	10	10	72830.8	108137.3	70529.8	78778.6	69596.6	89474.9	101488	91197.6	91222.7	119173.7	0.079008	1.102329	79974.62	98511.38	Up	0.300748	0.738751	60S acidic ribosomal protein P2 OS=Mus musculus GN=Rplp2 PE=1 SV=3
Pafah1b1	P63005	1	1	541071	509650	515529	508947	457717	505832	506291	597783	622497	595684	0.079992	1.096953	506582.8	565617.4	Up	0.159028	0.738751	Platelet-activating factor acetylhydrolase IB subunit alpha OS=Mus musculus GN=Pafah1b1 PE=1 SV=2
Csnk2a2	O54833	1	1	8512	9475	14406	13822	8069	14451	15901	12131	15405	12532	0.080277	1.095409	10856.8	14084	Up	0.375458	0.738751	Casein kinase II subunit alpha' OS=Mus musculus GN=Csnk2a2 PE=2 SV=1
Shank3	Q4ACU6-6	6	6	88737.5	94287	110189.7	110470	127091.7	126311.3	127261.8	155343.8	108207.8	117873.2	0.080474	1.094344	106155.2	126999.6	Up	0.258649	0.738751	Isoform 5 of SH3 and multiple ankyrin repeat domains protein 3 OS=Mus musculus GN=Shank3
Hnrnph1	O35737	3	3	199509.3	461468	335777.7	365841.3	588166.3	373377.3	530382.7	602896.3	628768	635250.3	0.080903	1.092035	390152.5	554134.9	Up	0.506199	0.738751	Heterogeneous nuclear ribonucleoprotein H OS=Mus musculus GN=Hnrnph1 PE=1 SV=3
Setd3	Q91WC0	1	1	NA	43633	44152	49952	37621	41160	66991	59923	46876	61637	0.08112	1.090872	43839.5	55317.4	Up	0.335502	0.738751	Histone-lysine N-methyltransferase setd3 OS=Mus musculus GN=Setd3 PE=2 SV=1
Rims1	Q99NE5-4	6	6	274237	293713	280193.5	253579	267737	303847	287691	300160	277985	283050	0.082309	1.084553	273891.9	290546.6	Up	0.085163	0.738751	Isoform 4 of Regulating synaptic membrane exocytosis protein 1 OS=Mus musculus GN=Rims1
Mpp5	Q9JLB2	1	1	132575	215176	142081	120948	187939	203911	229984	196554	202874	174228	0.083024	1.080796	159743.8	201510.2	Up	0.335093	0.738751	MAGUK p55 subfamily member 5 OS=Mus musculus GN=Mpp5 PE=1 SV=1
Adcy5	P84309-2	1	1	91940	178520	281046	173126	280344	338079	295135	264895	267719	253814	0.083114	1.080326	200995.2	283928.4	Up	0.498366	0.738751	Isoform 2 of Adenylate cyclase type 5 OS=Mus musculus GN=Adcy5
Mpp2	Q9WV34	8	8	178088.3	130251.4	179244	162468.7	137981.3	167346.9	197192.4	168224.6	191866.6	183307.7	0.084178	1.074801	157606.7	181587.6	Up	0.204337	0.738751	MAGUK p55 subfamily member 2 OS=Mus musculus GN=Mpp2 PE=1 SV=1
Mpp2	Q9WV34-2	7	7	178088.3	130251.4	179244	162468.7	137981.3	167346.9	197192.4	168224.6	191866.6	183307.7	0.084178	1.074801	157606.7	181587.6	Up	0.204337	0.738751	Isoform 2 of MAGUK p55 subfamily member 2 OS=Mus musculus GN=Mpp2
Ctsd	P18242	7	7	994058.3	1294651	659112.2	884419.5	804558	1205857	930735.3	1229768	1155292	1532903	0.084582	1.072722	927359.9	1210911	Up	0.384892	0.738751	Cathepsin D OS=Mus musculus GN=Ctsd PE=1 SV=1
Gprin3	Q8BWS5	2	2	20682.5	39089.5	15085	30778	19343.5	41965	25569.5	30192	37609.5	48481	0.084639	1.072429	24995.7	36763.4	Up	0.55659	0.738751	G protein-regulated inducer of neurite outgrowth 3 OS=Mus musculus GN=Gprin3 PE=1 SV=1
Dlg3	P70175	11	11	241935.6	181657.8	196959.1	197250.3	204165.8	151312.5	200757.3	207003.1	155918	151641.6	0.091268	1.039681	204393.7	173326.5	Down	-0.23786	0.738751	Disks large homolog 3 OS=Mus musculus GN=Dlg3 PE=1 SV=1
Ganab	Q8BHN3-3	3	3	329372.3	473671.3	455551	546292	574919.7	671345.3	543301	600026.7	604902.7	487187	0.084915	1.071016	475961.3	581352.5	Up	0.288569	0.738751	Isoform 3 of Neutral alpha-glucosidase AB OS=Mus musculus GN=Ganab
Tnik	P83510	5	5	33464	72487	71002	80782.5	83981	115169	106673.8	71670.5	83148.75	84417.5	0.085004	1.070561	68343.3	92215.9	Up	0.432216	0.738751	Traf2 and NCK-interacting protein kinase OS=Mus musculus GN=Tnik PE=1 SV=2
Tnik	P83510-2	4	4	33464	72487	71002	80782.5	83981	115169	106673.8	71670.5	83148.75	84417.5	0.085004	1.070561	68343.3	92215.9	Up	0.432216	0.738751	Isoform 2 of Traf2 and NCK-interacting protein kinase OS=Mus musculus GN=Tnik
Mbnl1	Q9JKP5	1	1	49315	127636	92885	109299	99066	113381	107270	122107	133908	165942	0.085073	1.070208	95640.2	128521.6	Up	0.426322	0.738751	Muscleblind-like protein 1 OS=Mus musculus GN=Mbnl1 PE=1 SV=1
Rab11fip5	Q8R361	3	3	285597	237004	241061.5	197258	236597	212621	251938	176248	190544.5	175772	0.093648	1.028501	239503.5	201424.7	Down	-0.24981	0.738751	Rab11 family-interacting protein 5 OS=Mus musculus GN=Rab11fip5 PE=1 SV=2
Cacna1c	Q01815-3	1	1	NA	25638	24541	16098	24604	NA	59152	19019	58985	47586	0.085909	1.065961	22720.25	46185.5	Up	1.023461	0.738751	Isoform 3 of Voltage-dependent L-type calcium channel subunit alpha-1C OS=Mus musculus GN=Cacna1c

Pabpc1	P29341	8	8	355132.9	485057.3	524692.1	505406.1	541504.6	740733	558248.4	627217.6	540068.6	487321	0.086192	1.064533	482358.6	590717.7	Up	0.292363	0.738751	Polyadenylate-binding protein 1 OS=Mus musculus GN=Pabpc1 PE=1 SV=2
Atp6v1e1	P50518	16	16	614337.1	456181.1	434851.8	378086.6	472282.3	464877.6	397531	367050.9	332427.6	348438.6	0.094809	1.02315	471147.8	382065.1	Down	-0.30236	0.738751	V-type proton ATPase subunit E 1 OS=Mus musculus GN=Atp6v1e1 PE=1 SV=2
Rims1	Q99NE5-7	7	7	182824.7	195808.7	188504	169052.7	180132.3	204333.7	193575.7	200106.7	185323.3	188700	0.087038	1.060291	183264.5	194407.9	Up	0.08516	0.738751	Isoform 7 of Regulating synaptic membrane exocytosis protein 1 OS=Mus musculus GN=Rims1
Mark3	Q03141-2	5	5	82065	53834.8	76464	54275.4	71968.4	69588.8	57649	24904.8	55290.8	36528.8	0.094866	1.022889	67721.52	48792.44	Down	-0.47296	0.738751	Isoform 2 of MAP/microtubule affinity-regulating kinase 3 OS=Mus musculus GN=Mark3
Syap1	Q9D5V6	2	2	233498	215942	255177.5	236487	237927	353589	244297	231715.5	292112.5	303116.5	0.087344	1.058767	235806.3	284966.1	Up	0.273188	0.738751	Synapse-associated protein 1 OS=Mus musculus GN=Syap1 PE=1 SV=1
4933434E	Q8R092-2	1	1	2382	3854	3812	2989	3114	3615	4521	5621	3515	3676	0.088481	1.05315	3230.2	4189.6	Up	0.375189	0.738751	Isoform 3 of Uncharacterized protein C1orf43 homolog OS=Mus musculus
Ndufa5	Q9CPP6	6	6	112184.5	85812	87992.5	89166	96054.25	99567.75	76246	86905.5	61033.75	55269.25	0.095579	1.019638	94241.85	75804.45	Down	-0.31409	0.738751	NADH dehydrogenase [ubiquinone] 1 alpha subcomplex subunit 5 OS=Mus musculus GN=Ndufa5 PE=1 SV=3
Hist1h3g	P68433	1	1	NA	NA	7887	9070	NA	NA	12753	27989	20419	11064	0.089107	1.050088	8478.5	18056.25	Up	1.090617	0.738751	Histone H3.1 OS=Mus musculus GN=Hist1h3a PE=1 SV=2
Map6	Q7TSJ2	35	35	243854	178943	150728.8	158570	202223.2	173248.9	150498	151916.7	142334.9	132751.2	0.09571	1.019043	186863.8	150150	Down	-0.31558	0.738751	Microtubule-associated protein 6 OS=Mus musculus GN=Map6 PE=1 SV=2
G3bp2	P97379	4	4	98675.75	111996.3	142448	106857.3	110791.8	111499.8	138613	142333.5	134465.8	133950.8	0.08916	1.04983	114153.8	132172.6	Up	0.211444	0.738751	Ras GTPase-activating protein-binding protein 2 OS=Mus musculus GN=G3bp2 PE=1 SV=2
Rpl32-ps	P17932	1	1	1556016	1274351	1086774	1194514	1586549	2005444	1501792	1878921	1476783	1377196	0.089185	1.049708	1339641	1648027	Up	0.298894	0.738751	Putative 60S ribosomal protein L32' OS=Mus musculus GN=Rpl32-ps PE=5 SV=2
Dopey2	Q3UHQ6	1	1	NA	98585	89777	90631	96052	152544	112972	121690	85797	114652	0.089389	1.048716	93761.25	117531	Up	0.325978	0.738751	Protein dopey-2 OS=Mus musculus GN=Dopey2 PE=1 SV=3
Rdx	P26043	4	4	448579.3	450322.3	489037.5	506015.8	434255.5	454433	591148.8	517655	596941.5	487298.3	0.089399	1.048667	465642.1	529495.3	Up	0.185397	0.738751	Radixin OS=Mus musculus GN=Rdx PE=1 SV=3
Map4	P27546-3	8	8	75520.25	30906.5	39769	38901.12	63799.88	37893.75	31226.38	35668.75	29385.62	23374.12	0.097034	1.013076	49779.35	31509.72	Down	-0.65975	0.738751	Isoform 3 of Microtubule-associated protein 4 OS=Mus musculus GN=Map4
Fan1	Q69ZT1	1	1	11176	19523	19526	16746	16800	19101	20599	26968	18893	19110	0.089763	1.046903	16754.2	20934.2	Up	0.321339	0.738751	Fanconi-associated nuclease 1 OS=Mus musculus GN=Fan1 PE=2 SV=2
Stum	Q0VBF8	1	1	NA	33697	57600	55826	NA	155573	37886	51612	128411	154481	0.09001	1.045709	49041	105592.6	Up	1.106448	0.738751	Uncharacterized membrane protein C1orf95 homolog OS=Mus musculus GN=Stum PE=2 SV=1
Rabggta	Q9JHK4	1	1	301748	161875	146693	109096	233228	125983	NA	61879	175628	63819	0.098429	1.006877	190528	106827.3	Down	-0.83472	0.738751	Geranylgeranyl transferase type-2 subunit alpha OS=Mus musculus GN=Rabggta PE=2 SV=1
Lysmd2	Q9D7V2	1	1	501215	314804	322655	344378	341723	325931	347915	281733	265668	177720	0.098443	1.006815	364955	279793.4	Down	-0.38336	0.738751	LysM and putative peptidoglycan-binding domain-containing protein 2 OS=Mus musculus GN=Lysmd2 PE=2 SV=1
Cxcl12	P40224	1	1	63428	41914	39648	41215	55973	70358	58323	64270	54720	51516	0.090524	1.043236	48435.6	59837.4	Up	0.30498	0.738751	Stromal cell-derived factor 1 OS=Mus musculus GN=Cxcl12 PE=2 SV=2
Unc13a	Q4KUS2	1	1	42698	65711	71239	64325	79350	36840	21475	65492	57283	49617	0.099471	1.002304	64664.6	46141.4	Down	-0.48691	0.738751	Protein unc-13 homolog A OS=Mus musculus GN=Unc13a PE=1 SV=3
Ncdn	Q9Z0E0	3	3	65118.33	122070.3	173265	146243	127858	151220	179678	161809.3	180949	158624.3	0.090789	1.041967	126910.9	166456.1	Up	0.391326	0.738751	Neurochondrin OS=Mus musculus GN=Ncdn PE=1 SV=1
Mrpl58	Q8R035	1	1	2843	4099	3389	3151	3150	5213	3216	5185	3868	3740	0.090906	1.041407	3326.4	4244.4	Up	0.351599	0.738751	Peptidyl-tRNA hydrolase ICT1, mitochondrial OS=Mus musculus GN=Ict1 PE=1 SV=1
Kif3a	P28741	2	2	670977	892839.5	770666.5	961706	652275	923625.5	990322.5	784877.5	1054084	925467.5	0.091892	1.036722	789692.8	935675.3	Up	0.244716	0.738751	Kinesin-like protein KIF3A OS=Mus musculus GN=Kif3a PE=1 SV=2
Sepw1	P63300	1	1	609790	347761	461710	414309	478243	380943	407691	438614	314180	272227	0.100985	0.995743	462314.6	362731	Down	-0.34997	0.738751	Selenoprotein W OS=Mus musculus GN=Sepw1 PE=1 SV=1
Git1	Q68FF6	13	13	43586.31	116269.2	122343.1	130448.9	105874.9	123819.5	140393.5	153212.5	129149.2	143432.5	0.092134	1.03558	103704.5	138001.4	Up	0.412205	0.738751	ARF GTPase-activating protein GIT1 OS=Mus musculus GN=Git1 PE=1 SV=1
Rps3	P62908	3	3	79340.5	93985.5	87725	85122.5	88831.5	41699.5	80076.5	28193	81673	81468	0.101062	0.995412	87001	62622	Down	-0.47436	0.738751	40S ribosomal protein S3 OS=Mus musculus GN=Rps3 PE=1 SV=1
Pitpnm2	Q6ZPQ6	2	2	1795442	1983511	1449844	1487085	1533024	2749905	1966989	1859987	1843683	1841613	0.092308	1.034761	1649781	2052435	Up	0.315062	0.738751	Membrane-associated phosphatidylinositol transfer protein 2 OS=Mus musculus GN=Pitpnm2 PE=1 SV=2
Ppp1r7	Q3UM45	8	8	201169.8	232197.8	207669.4	183828.9	223483	260424.4	208524.1	226489.9	231180.3	235133	0.093576	1.028836	209669.8	232350.3	Up	0.148183	0.738751	Protein phosphatase 1 regulatory subunit 7 OS=Mus musculus GN=Ppp1r7 PE=1 SV=2
Map2k7	Q8CE90-5	2	2	228036	274859.5	251727	283808.5	230715	320406.5	269149	289987.5	249441	297819	0.094042	1.026678	253829.2	285360.6	Up	0.168928	0.738751	Isoform 5 of Dual specificity mitogen-activated protein kinase kinase 7 OS=Mus musculus GN=Map2k7
Aspa	Q8R3P0	2	2	144910.5	152655	179449.5	187010	138507	80202	163486	140681	135131	126180.5	0.102009	0.991362	160506.4	129136.1	Down	-0.31374	0.738751	Aspartoacylase OS=Mus musculus GN=Aspa PE=1 SV=1
Map6	Q7TSJ2-2	29	29	301240.5	217080	182355.4	190745.1	241130.8	207729.3	181872.7	183382.2	172329.4	160697	0.102226	0.990439	226510.4	181202.1	Down	-0.32198	0.738751	Isoform 2 of Microtubule-associated protein 6 OS=Mus musculus GN=Map6
Opalin	Q7M750	1	1	33239	35928	68171	57832	38569	27077	29326	45146	18133	36421	0.102287	0.99018	46747.8	31220.6	Down	-0.5824	0.738751	Opalin OS=Mus musculus GN=Opalin PE=1 SV=1
Map2k7	Q8CE90-3	1	1	172831	358733	301002	342794	250976	432007	287602	421620	321685	381208	0.094629	1.023976	285267.2	368824.4	Up	0.37062	0.738751	Isoform 3 of Dual specificity mitogen-activated protein kinase kinase 7 OS=Mus musculus GN=Map2k7
Madd	Q80U28-2	4	4	52816.75	157044.3	153375.3	157186.5	121841.3	156641.3	182266.5	178527.3	172355.5	170325.3	0.094824	1.023082	128452.8	172023.2	Up	0.421364	0.738751	Isoform 2 of MAP kinase-activating death domain protein OS=Mus musculus GN=Madd
Hnrnpf	Q9Z2X1	5	5	121603.8	283812.2	214397.8	223241.2	356767.4	223733.2	322490.8	368959.6	378361.4	372465.2	0.094959	1.022464	239964.5	333202	Up	0.473576	0.738751	Heterogeneous nuclear ribonucleoprotein F OS=Mus musculus GN=Hnrnpf PE=1 SV=3
Sipa1l1	Q8C0T5	5	5	318341.3	253386	270882	207433.5	262603	252282.8	218570.8	240045.5	207022	201669.5	0.103333	0.985761	262529.2	223918.1	Down	-0.22951	0.738751	Signal-induced proliferation-associated 1-like protein 1 OS=Mus musculus GN=Sipa1l1 PE=1 SV=2
Lyrm4	Q8K215	1	1	112841	302503	266478	328347	252868	345952	334239	289679	322658	379684	0.094964	1.022441	252607.4	334442.4	Up	0.404861	0.738751	LYR motif-containing protein 4 OS=Mus musculus GN=Lyrm4 PE=1 SV=1
Cs	Q9CZU6	6	6	73618.33	138613.7	131117.5	155463.2	108071.7	176557.3	144450.2	138093.2	139368.5	167241.7	0.095666	1.019242	121376.9	153142.2	Up	0.335378	0.738751	Citrate synthase, mitochondrial OS=Mus musculus GN=Cs PE=1 SV=1
Tubal3	Q3UX10	3	3	190454	214983.3	159647.3	164910.3	159133	178930.7	150001	158272.7	137473	141584	0.104204	0.982116	177825.6	153252.3	Down	-0.21455	0.738751	Tubulin alpha chain-like 3 OS=Mus musculus GN=Tubal3 PE=2 SV=2
Camk2a	P11798	30	30	1016795	1840013	1982188	1974559	1693190	1970328	2158081	2128967	2070839	2117319	0.095899	1.018186	1701349	2089107	Up	0.296207	0.738751	Calcium/calmodulin-dependent protein kinase type II subunit alpha OS=Mus musculus GN=Camk2a PE=1 SV=2
Erc2	Q6PH08-2	6	6	36011	57402	69591.6	78969.4	54388.4	64813.6	78777.2	74119.2	78023	79810.2	0.096513	1.015414	59272.48	75108.64	Up	0.341616	0.738751	Isoform 2 of ERC protein 2 OS=Mus musculus GN=Erc2
Sec31a	Q3UPL0	3	3	136806.7	300475	234592	290787.3	187621.7	270914.3	298386	260408	315167	347577.3	0.096607	1.014991	230056.5	298490.5	Up	0.375697	0.738751	Protein transport protein Sec31A OS=Mus musculus GN=Sec31a PE=1 SV=2
Anxa4	P97429	1	1	NA	4323	3524	3589	2820	4562	3929	5214	3469	6220	0.096622	1.014924	3564	4678.8	Up	0.392641	0.738751	Annexin A4 OS=Mus musculus GN=Anxa4 PE=2 SV=4
Syn2	Q64332	24	24	141261.1	191509.3	154044.9	167468.7	153725.7	172949.2	171673.6	182013.8	183772.1	190281.2	0.097778	1.009759	161601.9	180138	Up	0.156658	0.738751	Synapsin-2 OS=Mus musculus GN=Syn2 PE=1 SV=2
Cadm2	Q8BLQ9-3	11	11	334106	1153209	1156962	1201405	1126895	1310856	1360277	1231956	1295142	1554841	0.098168	1.00803	994515.3	1350614	Up	0.44155	0.738751	Isoform 3 of Cell adhesion molecule 2 OS=Mus musculus GN=Cadm2
Mbp	P04370-4	28	28	1081184	1266346	2034856	2270314	1483887	718348.1	1364814	1389288	704974.7	1372275	0.104693	0.980082	1627317	1109940	Down	-0.55201	0.738751	Isoform 4 of Myelin basic protein OS=Mus musculus GN=Mbp
Madd	Q80U28-1	3	3	70422.33	209392.3	199256.7	201202.3	154509.3	208855	234157.3	227121	221162	221158.7	0.098959	1.004545	166956.6	222490.8	Up	0.414273	0.738751	Isoform 11 of MAP kinase-activating death domain protein OS=Mus musculus GN=Madd
Ctnnd2	O35927	12	12	196261.8	181181.1	182997.4	169746.6	167247.4	235888.9	188609	195744.6	198350.1	182280.6	0.099613	1.001684	179486.9	200174.6	Up	0.157381	0.738751	Catenin delta-2 OS=Mus musculus GN=Ctnnd2 PE=1 SV=1
Hadh	Q61425	3	3	42916.33	50055	46847.33	58929	47800	58200.67	54897.33	52325.33	56445.67	80434.67	0.099836	1.000713	49309.53	60460.73	Up	0.294132	0.738751	Hydroxyacyl-coenzyme A dehydrogenase, mitochondrial OS=Mus musculus GN=Hadh PE=1 SV=2
Ubp1	Q811S7	1	1	128904	222291	168248	162445	132612	181157	173937	183969	248096	273844	0.100196	0.99915	162900	212200.6	Up	0.381442	0.738751	Upstream-binding protein 1 OS=Mus musculus GN=Ubp1 PE=1 SV=1
Rpl28	P41105	2	2	343294	568324	379711.5	425037.5	480404	595484.5	478998	443870.5	553564.5	752852	0.100987	0.995735	439354.2	564955.9	Up	0.362754	0.738751	60S ribosomal protein L28 OS=Mus musculus GN=Rpl28 PE=1 SV=2
Znrf2	Q71FD5	1	1	140427	235616	191406	250178	229169	269950	258285	210746	270128	254780	0.101275	0.994498	209359.2	252777.8	Up	0.271889	0.738751	E3 ubiquitin-protein ligase ZNRF2 OS=Mus musculus GN=Znrf2 PE=1 SV=1
Mrps18a	Q99N85	1	1	NA	51968	41633	52671	58172	22965	54086	44577	41581	34255	0.108143	0.966002	51111	39492.8	Down	-0.37204	0.738751	28S ribosomal protein S18a, mitochondrial OS=Mus musculus GN=Mrps18a PE=2 SV=1
Pafah1b2	Q61206	4	4	44640.33	125678.7	107428.3	63498.67	117970.3	124265.7	134355.7	102942.7	169827.7	112541.7	0.101368	0.994099	91843.27	128786.7	Up	0.487737	0.738751	Platelet-activating factor acetylhydrolase IB subunit beta OS=Mus musculus GN=Pafah1b2 PE=1 SV=2
Cacnb3	P54285	3	3	131417	207567	202729	207407.5	203778	276328	250512	235279.5	226538	173421	0.101385	0.994026	190579.7	232415.7	Up	0.286313	0.738751	Voltage-dependent L-type calcium channel subunit beta-3 OS=Mus musculus GN=Cacnb3 PE=2 SV=2
Atxn2	O70305	3	3	NA	10175.5	17159	12526.5	17015.5	17006	21689	12467	21032	23574	0.102678	0.988523	14219.13	19153.6	Up	0.429783	0.738751	Ataxin-2 OS=Mus musculus GN=Atxn2 PE=1 SV=1
Amph	Q7TQF7	21	21	686379.3	632361.7	538134.7	554042.5	589933.7	611975.3	509549.5	563793.2	489992	506655.8	0.108663	0.963918	600170.4	536393.1	Down	-0.16208	0.738751	Amphiphysin OS=Mus musculus GN=Amph PE=1 SV=1
Mrps23	Q8VE22	3	3	109431	92625	105175.3	103598.7	58762.33	63087.67	94110	59316.33	75964	75684	0.109461	0.960741	93918.47	73632.4	Down	-0.35107	0.738751	28S ribosomal protein S23, mitochondrial OS=Mus musculus GN=Mrps23 PE=1 SV=1
Tom1l2	Q5SRX1-3	5	5	NA	1366.33	8179.67	8232.33	11932.33	10105	11988	13836.33	15801	10542.33	0.102955	0.987353	7427.665	12454.53	Up	0.74569	0.738751	Isoform 3 of TOM1-like protein 2 OS=Mus musculus GN=Tom1l2
Emc8	O70378	1	1	66548	130526	66184	108804	102680	112081	94708	127016	143491	142723	0.103327	0.985786	94948.4	124003.8	Up	0.385169	0.738751	ER membrane protein complex subunit 8 OS=Mus musculus GN=Emc8 PE=1 SV=1
Sult4a1	P63046	1	1	152905	295749	270696	238040	148982	210202	308937	275373	308909	369379	0.103572	0.984758	221274.4	294560	Up	0.412725	0.738751	Sulfotransferase 4A1 OS=Mus musculus GN=Sult4a1 PE=2 SV=1
Gnb5	P62881	5	5	325784	479924.2	429857.8	455462.6	415863.6	451071.6	478418.8	482512.2	477401.8	493580.4	0.103614	0.984582	421378.4	476597	Up	0.177653	0.738751	Guanine nucleotide-binding protein subunit beta-5 OS=Mus musculus GN=Gnb5 PE=1 SV=1
Tns3	Q5SSZ5	1	1	NA	21593	25773	36529	31366	36216	47996	50317	22936	42009	0.104218	0.982057	28815.25	39894.8	Up	0.469368	0.738751	Tensin-3 OS=Mus musculus GN=Tns3 PE=1 SV=1
Scp2	P32020	6	6	1673778	1385832	1333631	1309228	1429702	1512528	1173461	1236287	1206655	1190357	0.11096	0.954834	1426434	1263857	Down	-0.17458	0.738751	Non-specific lipid-transfer protein OS=Mus musculus GN=Scp2 PE=1 SV=3
Scfd1	Q8BRF7	2	2	216034	61056.5	174270	109556.5	134205.5	89414.5	103978.5	107347	65858	54976	0.111276	0.953598	139024.5	84314.8	Down	-0.72148	0.738751	Sec1 family domain-containing protein 1 OS=Mus musculus GN=Scfd1 PE=1 SV=1
Slmap	Q3URD3-4	1	1	NA	47924	50930	40282	44124	100406	61240	59906	46769	57004	0.104305	0.981695	45815	65065	Up	0.506062	0.738751	Isoform 4 of Sarcolemmal membrane-associated protein OS=Mus musculus GN=Slmap
Dpp9	Q8BVG4	3	3	7690	19714.33	20573.33	24334	17938.67	20341.33	25909.33	25452.67	23589.33	24302.33	0.104538	0.980726	18050.07	23919	Up	0.406153	0.738751	Dipeptidyl peptidase 9 OS=Mus musculus GN=Dpp9 PE=2 SV=2
Mpst	Q99J99	7	7	96028	111249.6	113950	116060.7	131577.1	122458.7	102537.9	87160.86	78028	93678.14	0.112994	0.946945	113773.1	96772.71	Down	-0.23349	0.738751	3-mercaptopyruvate sulfurtransferase OS=Mus musculus GN=Mpst PE=1 SV=3
Spata5	Q3UMC0	1	1	NA	2700	2853	1776	2631	3045	2878	2484	3510	3480	0.10454	0.980718	2490	3079.4	Up	0.306504	0.738751	Spermatogenesis-associated protein 5 OS=Mus musculus GN=Spata5 PE=1 SV=2
Cspg4	Q8VHY0	1	1	128345	202430	173522	154298	160941	160974	191822	194013	220958	193830	0.1046	0.980468	163907.2	192319.4	Up	0.230625	0.738751	Chondroitin sulfate proteoglycan 4 OS=Mus musculus GN=Cspg4 PE=1 SV=3
Ablim2	Q8BL65-5	3	3	332077	217156.5	266554.5	206653	318258.5	217700	221190.5	256957	210837.5	164927	0.114025	0.943	268139.9	214322.4	Down	-0.3232	0.738751	Isoform 5 of Actin-binding LIM protein 2 OS=Mus musculus GN=Ablim2
Asrgl1	Q8C0M9	5	5	745018	440125.8	523363.8	430576	542484.4	472111.6	521551	394988.4	367424.6	314400.4	0.114566	0.940944	536313.6	414095.2	Down	-0.37311	0.738751	Isoaspartyl peptidase/L-asparaginase OS=Mus musculus GN=Asrgl1 PE=1 SV=1
C2cd2l	Q80X80	5	5	20383.4	43529.8	49751.8	57495.6	30141.2	48815	57555.8	42298.6	69018.8	59025.2	0.104647	0.980273	40260.36	55342.68	Up	0.459032	0.738751	C2 domain-containing protein 2-like OS=Mus musculus GN=C2cd2l PE=1 SV=3
Ruvbl1	P60122	1	1	17547	19213	23697	22496	9196	27356	23286	17794	24088	29509	0.105041	0.978641	18429.8	24406.6	Up	0.405231	0.738751	RuvB-like 1 OS=Mus musculus GN=Ruvbl1 PE=1 SV=1
Elavl3	Q60900-2	1	1	124258	113569	144070	125198	135932	123851	162820	135256	146509	154196	0.105413	0.977106	128605.4	144526.4	Up	0.168382	0.738751	Isoform HuC-S of ELAV-like protein 3 OS=Mus musculus GN=Elavl3
Amer2	Q8CCJ4	5	5	369000.8	284020.6	283272.2	272056.2	279262	379041.6	333493.2	313231.8	334415	325377.8	0.105696	0.975941	297522.4	337111.9	Up	0.180229	0.738751	APC membrane recruitment protein 2 OS=Mus musculus GN=Amer2 PE=1 SV=2
Map6d1	Q148B9	6	6	361626.3	279051	261795.3	315332.3	261451.3	259786.3	278420.3	243936	256152.3	251115.3	0.115236	0.938412	295851.3	257271.3	Down	-0.20158	0.738751	MAP6 domain-containing protein 1 OS=Mus musculus GN=Map6d1 PE=1 SV=1
Hspd1	P63038-2	13	13	3271153	1594210	2034830	1801807	2165843	2046141	1656168	1621163	1251978	1292091	0.115695	0.936685	2173569	1573508	Down	-0.46608	0.738751	Isoform 2 of 60 kDa heat shock protein, mitochondrial OS=Mus musculus GN=Hspd1
Iqsec2	Q5DU25	12	12	5783.4	19457	24588.3	26411.8	20525.5	28493.6	27610.6	26496.2	26495.2	25318.8	0.106133	0.97415	19353.2	26882.88	Up	0.474116	0.738751	IQ motif and SEC7 domain-containing protein 2 OS=Mus musculus GN=Iqsec2 PE=1 SV=3
Gak	Q99KY4	3	3	72516	82842.33	135761	125538	118384	171814.7	139333	123793.3	123533	120319.7	0.106382	0.973132	107008.3	135758.7	Up	0.343323	0.738751	Cyclin-G-associated kinase OS=Mus musculus GN=Gak PE=1 SV=2
Cacng8	Q8VHW2	4	4	NA	25682.75	21448.5	25080.25	26665.25	22026	27724.75	49608.5	35598.75	37174	0.106739	0.971677	24719.19	34426.4	Up	0.477884	0.738751	Voltage-dependent calcium channel gamma-8 subunit OS=Mus musculus GN=Cacng8 PE=1 SV=1
Nudt9	Q8BVU5	1	1	153549	304948	218751	348048	230340	307416	393566	270371	289154	381084	0.108446	0.964786	251127.2	328318.2	Up	0.386676	0.738751	ADP-ribose pyrophosphatase, mitochondrial OS=Mus musculus GN=Nudt9 PE=2 SV=1
Map4	P27546	9	9	115934.8	45874.56	57998.22	55579.11	93876.11	57141.67	47152	56827.78	42883.67	31513.33	0.116842	0.932401	73852.56	47103.69	Down	-0.64881	0.738751	Microtubule-associated protein 4 OS=Mus musculus GN=Map4 PE=1 SV=3
Dap3	Q9ER88	1	1	788862	560863	473302	485833	523765	486349	361220	477125	477582	448374	0.117054	0.931614	566525	450130	Down	-0.3318	0.738751	28S ribosomal protein S29, mitochondrial OS=Mus musculus GN=Dap3 PE=2 SV=1
Ablim2	Q8BL65-4	4	4	225159	147516.3	180355	139883.3	214677.7	148313	150348.7	174423.3	142967.3	112789.7	0.11805	0.927934	181518.3	145768.4	Down	-0.31644	0.738751	Isoform 4 of Actin-binding LIM protein 2 OS=Mus musculus GN=Ablim2
Ttyh3	Q6P5F7-2	1	1	NA	5207	11147	7936	8142	8125	11216	13085	11695	10433	0.108559	0.964334	8108	10910.8	Up	0.428339	0.738751	Isoform 2 of Protein tweety homolog 3 OS=Mus musculus GN=Ttyh3
Ostf1	Q62422	2	2	118266.5	127995	97937.5	101643.5	113380.5	155532	126399	150120	105914	121181.5	0.108662	0.963922	111844.6	131829.3	Up	0.237175	0.738751	Osteoclast-stimulating factor 1 OS=Mus musculus GN=Ostf1 PE=1 SV=2
Dpp6	Q9Z218	2	2	24898	47569	42804	39362	33355.5	36029	42417.5	53511.5	50211.5	52395	0.109519	0.960511	37597.7	46912.9	Up	0.31934	0.738751	Dipeptidyl aminopeptidase-like protein 6 OS=Mus musculus GN=Dpp6 PE=1 SV=1
Atp5o	Q9DB20	29	29	1616654	1431390	1424853	1573281	1555670	1591270	1392184	1322191	1146427	1410499	0.119227	0.923625	1520369	1372514	Down	-0.1476	0.738751	ATP synthase subunit O, mitochondrial OS=Mus musculus GN=Atp5o PE=1 SV=1
Dlst	Q9D2G2	4	4	523805.7	383700.3	329151	485772.3	405389	317730.7	377908.7	321659.7	418433.3	329389.3	0.119549	0.922454	425563.7	353024.3	Down	-0.26961	0.738751	Dihydrolipoyllysine-residue succinyltransferase component of 2-oxoglutarate dehydrogenase complex, mitochondrial OS=Mus musculus GN=Dlst PE=1 SV=1
Hectd3	Q3U487	1	1	26427	66299	54660	73714	40075	72242	63432	66163	69295	78408	0.109611	0.960146	52235	69908	Up	0.420441	0.738751	E3 ubiquitin-protein ligase HECTD3 OS=Mus musculus GN=Hectd3 PE=1 SV=2
S100a11	P50543	1	1	46773	114203	99434	97798	76026	87519	115761	109921	132221	115795	0.109823	0.959307	86846.8	112243.4	Up	0.370086	0.738751	Protein S100-A11 OS=Mus musculus GN=S100a11 PE=1 SV=1
Basp1	Q91XV3	12	12	157017	204498.5	125992.5	190189	178809.4	198195.1	193536.3	173016.6	210450.9	245654.6	0.109835	0.959259	171301.3	204170.7	Up	0.25324	0.738751	Brain acid soluble protein 1 OS=Mus musculus GN=Basp1 PE=1 SV=3
Chchd6	Q91VN4	3	3	249689	229674.7	269299	232105.3	293638.3	265096	227461	230328.3	208474	201072.3	0.121508	0.915395	254881.3	226486.3	Down	-0.1704	0.738751	Coiled-coil-helix-coiled-coil-helix domain-containing protein 6, mitochondrial OS=Mus musculus GN=Chchd6 PE=2 SV=2
Hmga1	P17095	1	1	892571	192347	774068	453529	1688106	212494	509714	446155	273946	58016	0.121584	0.915124	800124.2	300065	Down	-1.41495	0.738751	High mobility group protein HMG-I/HMG-Y OS=Mus musculus GN=Hmga1 PE=1 SV=4
Pip4k2b	Q80XI4	5	5	13511.5	34237.25	26829.5	45725.5	24706.75	44877.25	36075	29820	42253	50841.25	0.110631	0.956123	29002.1	40773.3	Up	0.491467	0.738751	Phosphatidylinositol 5-phosphate 4-kinase type-2 beta OS=Mus musculus GN=Pip4k2b PE=1 SV=1
Arhgef7	Q9ES28-3	1	1	264955	320510	351565	282487	390467	393944	515465	360644	380658	320224	0.112664	0.948215	321996.8	394187	Up	0.291834	0.738751	Isoform C of Rho guanine nucleotide exchange factor 7 OS=Mus musculus GN=Arhgef7
Tcrg-V3	P06334	1	1	164861	374128	384051	314071	280908	308526	407672	409719	519958	347942	0.112716	0.948014	303603.8	398763.4	Up	0.393343	0.738751	T-cell receptor gamma chain C region DFL12 OS=Mus musculus PE=1 SV=1
Bsg	P18572-2	7	7	178070.7	180462.5	196269	170603.5	177929.2	241704.8	203101.7	257811	187822.5	174111.8	0.11334	0.945617	180667	212910.4	Up	0.236913	0.738751	Isoform 2 of Basigin OS=Mus musculus GN=Bsg
Atp5e	P56382	2	2	NA	437289.5	386133	308700	143194	236410.5	143836.5	164214	NA	188972.5	0.12263	0.911403	318829.1	183358.4	Down	-0.79812	0.738751	ATP synthase subunit epsilon, mitochondrial OS=Mus musculus GN=Atp5e PE=1 SV=2
Acy1	Q99JW2	2	2	69202	94684.5	97318	119595.5	74359	121332.5	102147.5	114749	96666	113784.5	0.113625	0.944526	91031.8	109735.9	Up	0.269593	0.738751	Aminoacylase-1 OS=Mus musculus GN=Acy1 PE=1 SV=1
Picalm	Q7M6Y3-6	3	3	24663	159535.3	130539.3	165389.3	147056.7	170457.3	170350.7	188706	157067.3	201829	0.114647	0.940637	125436.7	177682.1	Up	0.502338	0.738751	Isoform 6 of Phosphatidylinositol-binding clathrin assembly protein OS=Mus musculus GN=Picalm
Mrpl45	Q9D0Q7	1	1	1282560	467673	564229	538183	681014	552216	469281	340139	409294	317834	0.123726	0.907539	706731.8	417752.8	Down	-0.75851	0.738751	39S ribosomal protein L45, mitochondrial OS=Mus musculus GN=Mrpl45 PE=2 SV=1
Ksr1	Q61097-2	4	4	229185.3	182818.3	190132.5	160428.8	201497.3	143611	185925.8	193306.5	150103.3	162504.5	0.12398	0.906648	192812.4	167090.2	Down	-0.20657	0.738751	Isoform 2 of Kinase suppressor of Ras 1 OS=Mus musculus GN=Ksr1
S100a13	P97352	2	2	977092	469799	499973.5	658979	729099	697625.5	497414	401765	394985	391579.5	0.124131	0.90612	666988.5	476673.8	Down	-0.48466	0.738751	Protein S100-A13 OS=Mus musculus GN=S100a13 PE=1 SV=1
Serbp1	Q9CY58-3	1	1	98393	303790	148569	178907	136319	278343	216398	241813	219194	265452	0.11478	0.940134	173195.6	244240	Up	0.495897	0.738751	Isoform 3 of Plasminogen activator inhibitor 1 RNA-binding protein OS=Mus musculus GN=Serbp1
Rps15	P62843	1	1	NA	33190	21552	24811	45460	120193	33530	44887	46796	66763	0.114807	0.940032	31253.25	62433.8	Up	0.998321	0.738751	40S ribosomal protein S15 OS=Mus musculus GN=Rps15 PE=2 SV=2
Sv2a	Q9JIS5	5	5	321769	494888.3	643746.3	672597.3	516490.3	602516	669856.7	700054.3	711348.7	599493	0.114847	0.93988	529898.3	656653.7	Up	0.309417	0.738751	Synaptic vesicle glycoprotein 2A OS=Mus musculus GN=Sv2a PE=1 SV=1
Dstn	Q9R0P5	9	9	918061.3	575121.2	484473.1	493134.7	517892.3	566100.2	438751.3	477868.9	374160.2	310414.3	0.12545	0.901529	597736.5	433459	Down	-0.46361	0.738751	Destrin OS=Mus musculus GN=Dstn PE=1 SV=3
Mark1	Q8VHJ5	3	3	214322.7	147911.7	179842.3	139483.7	175860.7	171921.3	150799.3	108414.3	150850.7	128336.7	0.125666	0.900782	171484.2	142064.5	Down	-0.27153	0.738751	Serine/threonine-protein kinase MARK1 OS=Mus musculus GN=Mark1 PE=1 SV=2
Vps29	Q9QZ88	1	1	35442	132641	134635	123635	105344	130693	115946	172535	139753	166974	0.115722	0.936584	106339.4	145180.2	Up	0.449168	0.738751	Vacuolar protein sorting-associated protein 29 OS=Mus musculus GN=Vps29 PE=1 SV=1
Picalm	Q7M6Y3-3	4	4	18497.25	120539	98225.25	124283.8	110292.5	127843	127763	141529.5	118276.3	151889.5	0.116443	0.933887	94367.55	133460.3	Up	0.500047	0.738751	Isoform 3 of Phosphatidylinositol-binding clathrin assembly protein OS=Mus musculus GN=Picalm
Prep	Q9QUR6	1	1	NA	7839	14128	8255	6257	10654	13124	14970	12147	13302	0.116478	0.933756	9119.75	12839.4	Up	0.493512	0.738751	Prolyl endopeptidase OS=Mus musculus GN=Prep PE=2 SV=1
Spock2	Q9ER58	1	1	218760	292980	285396	256643	258172	548136	361358	310637	311463	270878	0.116629	0.933193	262390.2	360494.4	Up	0.458263	0.738751	Testican-2 OS=Mus musculus GN=Spock2 PE=2 SV=1
Gnl1	P36916	4	4	94311.25	63443.75	72732	70273.75	88168	56999.25	79026.5	71964	64869.25	49333.25	0.126398	0.89826	77785.75	64438.45	Down	-0.27158	0.738751	Guanine nucleotide-binding protein-like 1 OS=Mus musculus GN=Gnl1 PE=2 SV=4
Fn3krp	Q8K274	1	1	304558	233725	274470	291126	315539	204010	230198	237109	300103	262948	0.126621	0.897494	283883.6	246873.6	Down	-0.20153	0.738751	Ketosamine-3-kinase OS=Mus musculus GN=Fn3krp PE=2 SV=2
Rwdd1	Q9CQK7	1	1	172268	269589	262942	183384	182753	281788	258002	234560	261438	249129	0.118181	0.927452	214187.2	256983.4	Up	0.262803	0.738751	RWD domain-containing protein 1 OS=Mus musculus GN=Rwdd1 PE=1 SV=1
Aldh5a1	Q8BWF0	20	20	872291.4	759027.1	868024.9	1076082	917794.3	870539.4	738786.8	806888.3	617906.4	865918.1	0.127601	0.894146	898643.9	780007.8	Down	-0.20426	0.738751	Succinate-semialdehyde dehydrogenase, mitochondrial OS=Mus musculus GN=Aldh5a1 PE=1 SV=1
Sco1	Q5SUC9	1	1	561050	686633	578415	684166	583106	590516	539815	538080	552830	601096	0.127825	0.893384	618674	564467.4	Down	-0.13229	0.738751	Protein SCO1 homolog, mitochondrial OS=Mus musculus GN=Sco1 PE=2 SV=1
Fnbp1l	Q8K012	2	2	2122792	1572676	1741021	1308787	1832033	1667572	1518175	1496008	1201478	1319134	0.127926	0.893041	1715462	1440473	Down	-0.25205	0.738751	Formin-binding protein 1-like OS=Mus musculus GN=Fnbp1l PE=1 SV=2
Npepps	Q11011	4	4	127062	147731.3	169874.7	153294.7	111022.7	158013	163313	165260.7	162637.3	161779.3	0.118506	0.92626	141797.1	162200.7	Up	0.193952	0.738751	Puromycin-sensitive aminopeptidase OS=Mus musculus GN=Npepps PE=1 SV=2
Pip4k2a	O70172	7	7	124350.4	132626	133403.3	138679.3	146066.1	144600.7	147574.6	141449.6	146023.9	133921.6	0.11881	0.925147	135025	142714.1	Up	0.079901	0.738751	Phosphatidylinositol 5-phosphate 4-kinase type-2 alpha OS=Mus musculus GN=Pip4k2a PE=1 SV=1
Rps20	P60867	4	4	222280.7	192463	148772.3	151199	138154	234723	203863.3	152387	228325	223663.3	0.119964	0.920949	170573.8	208592.3	Up	0.29029	0.738751	40S ribosomal protein S20 OS=Mus musculus GN=Rps20 PE=1 SV=1
Atp2b2	Q9R0K7	10	10	640591.4	1250679	1041562	1626916	1353131	1581111	1601665	1340655	1431544	1592598	0.120104	0.920443	1182576	1509515	Up	0.352152	0.738751	Plasma membrane calcium-transporting ATPase 2 OS=Mus musculus GN=Atp2b2 PE=1 SV=2
Atg3	Q9CPX6	2	2	463174.5	264761.5	357439	288705	349420.5	267048	291648.5	291191.5	260997.5	284037.5	0.129411	0.888029	344700.1	278984.6	Down	-0.30516	0.738751	Ubiquitin-like-conjugating enzyme ATG3 OS=Mus musculus GN=Atg3 PE=1 SV=1
Lpin2	Q99PI5	2	2	39013	78732.5	49411	57427.5	44915	61734.5	64580	62754.5	66257.5	90614.5	0.121497	0.915434	53899.8	69188.2	Up	0.360246	0.738751	Phosphatidate phosphatase LPIN2 OS=Mus musculus GN=Lpin2 PE=1 SV=2
Glod4	Q9CPV4	3	3	316336	231596.7	248954.7	248433	246756	298009	270645.3	269194.3	300704	303918.3	0.12161	0.915031	258415.3	288494.2	Up	0.158851	0.738751	Glyoxalase domain-containing protein 4 OS=Mus musculus GN=Glod4 PE=1 SV=1
Mrpl40	Q9Z2Q5	1	1	34679	21961	21012	22645	21171	21850	20899	21250	14067	17274	0.130062	0.88585	24293.6	19068	Down	-0.34942	0.738751	39S ribosomal protein L40, mitochondrial OS=Mus musculus GN=Mrpl40 PE=2 SV=2
Glrx2	Q923X4	1	1	443811	402967	481698	608669	492394	453216	423291	430237	312984	444624	0.130275	0.885139	485907.8	412870.4	Down	-0.23499	0.738751	Glutaredoxin-2, mitochondrial OS=Mus musculus GN=Glrx2 PE=1 SV=1
Dnajc11	Q5U458	2	2	116636.5	120306.5	169959.5	149143	134682	109499	118720	144684	108834	105970	0.131223	0.88199	138145.5	117541.4	Down	-0.23302	0.738751	DnaJ homolog subfamily C member 11 OS=Mus musculus GN=Dnajc11 PE=2 SV=2
Cand1	Q6ZQ38	2	2	140537	245004	301211	381855	224955	331754	341611	297436	350673	366686	0.121875	0.914085	258712.4	337632	Up	0.384102	0.738751	Cullin-associated NEDD8-dissociated protein 1 OS=Mus musculus GN=Cand1 PE=2 SV=2
Sec31a	Q3UPL0-3	2	2	205210	303672.5	239842.5	316073	201069.5	280030	286044.5	279950.5	322251	343889.5	0.121949	0.913822	253173.5	302433.1	Up	0.25649	0.738751	Isoform 3 of Protein transport protein Sec31A OS=Mus musculus GN=Sec31a
Lactb	Q9EP89	2	2	424197.5	360817	345398.5	335626.5	335307	393784	308894.5	259785	290659	313037	0.131613	0.880701	360269.3	313231.9	Down	-0.20184	0.738751	Serine beta-lactamase-like protein LACTB, mitochondrial OS=Mus musculus GN=Lactb PE=1 SV=1
Rgs7	O54829	1	1	36633	50964	43454	43414	46776	62527	50277	39318	54485	53912	0.122399	0.912222	44248.2	52103.8	Up	0.23577	0.738751	Regulator of G-protein signaling 7 OS=Mus musculus GN=Rgs7 PE=1 SV=2
Hdhd3	Q9CYW4	1	1	NA	15278	13511	16631	11045	26683	23809	14376	15604	15452	0.123163	0.90952	14116.25	19184.8	Up	0.442607	0.738751	Haloacid dehalogenase-like hydrolase domain-containing protein 3 OS=Mus musculus GN=Hdhd3 PE=1 SV=1
Gnal	Q8CGK7	3	3	5773621	1004252	4709211	2530731	6817907	2798717	2815740	3027516	1467896	383566	0.131966	0.879538	4167145	2098687	Down	-0.98957	0.738751	Guanine nucleotide-binding protein G(olf) subunit alpha OS=Mus musculus GN=Gnal PE=1 SV=1
Pgp	Q8CHP8	10	10	104769.6	140056.4	113620.8	103933.4	98898.4	108018.6	132272.6	136213.2	124283.2	142045.8	0.123204	0.909375	112255.7	128566.7	Up	0.195728	0.738751	Phosphoglycolate phosphatase OS=Mus musculus GN=Pgp PE=1 SV=1
Babam1	Q3UI43	1	1	66736	58631	60523	49135	70868	30511	67781	57643	47177	32455	0.13226	0.878571	61178.6	47113.4	Down	-0.37689	0.738751	BRISC and BRCA1-A complex member 1 OS=Mus musculus GN=Babam1 PE=2 SV=1
Gnas	P63094	4	4	4330216	767384.8	3545978	1917130	5126068	2116323	2129410	2287644	1120306	304115.5	0.13251	0.877751	3137355	1591559	Down	-0.97911	0.738751	Guanine nucleotide-binding protein G(s) subunit alpha isoforms short OS=Mus musculus GN=Gnas PE=1 SV=1
Ssr1	Q9CY50	1	1	NA	17137	4548	11231	3242	23989	6471	16610	13639	27523	0.124555	0.904639	9039.5	17646.4	Up	0.965059	0.738751	Translocon-associated protein subunit alpha OS=Mus musculus GN=Ssr1 PE=1 SV=1
Adgrl1	Q80TR1	10	10	123111.3	221886.4	217921.1	201894	253342	289898.9	243023.3	252220.4	236329.9	217305	0.124651	0.904304	203631	247755.5	Up	0.28296	0.738751	Latrophilin-1 OS=Mus musculus GN=Lphn1 PE=1 SV=2
Ksr1	Q61097	3	3	203161	201225.3	198388.3	168074.7	218152.3	122864.7	197768.3	200726	152918	170919	0.133006	0.876129	197800.3	169039.2	Down	-0.22669	0.738751	Kinase suppressor of Ras 1 OS=Mus musculus GN=Ksr1 PE=1 SV=1
Atp5h	Q9DCX2	13	13	1779155	462519.7	921373.9	624331.8	1061056	811940.7	613956.4	581360.8	391859.7	265768.3	0.133714	0.873823	969687.2	532977.2	Down	-0.86345	0.738751	ATP synthase subunit d, mitochondrial OS=Mus musculus GN=Atp5h PE=1 SV=3
L1cam	P11627	12	12	600336	197994.1	370817.6	224410.4	425335.8	294801.6	249640.3	248693.3	182299	150279.8	0.133788	0.873583	363778.8	225142.8	Down	-0.69222	0.738751	Neural cell adhesion molecule L1 OS=Mus musculus GN=L1cam PE=1 SV=1
Npc1	O35604	1	1	39052	50368	55155	56104	50752	46190	70716	79298	54558	59416	0.125348	0.901883	50286.2	62035.6	Up	0.302934	0.738751	Niemann-Pick C1 protein OS=Mus musculus GN=Npc1 PE=1 SV=2
Bin1	O08539-2	12	12	379952.4	281761.2	261388.8	295161.6	290849.1	275547.5	271001.4	260973.3	247931.1	261944.9	0.133816	0.873492	301822.6	263479.6	Down	-0.19601	0.738751	Isoform 2 of Myc box-dependent-interacting protein 1 OS=Mus musculus GN=Bin1
Cacnb4	Q8R0S4-2	2	2	71739	148874	153019	138525.5	136338.5	163892	176107	174467	163817	123765.5	0.125861	0.900109	129699.2	160409.7	Up	0.306592	0.738751	Isoform 2 of Voltage-dependent L-type calcium channel subunit beta-4 OS=Mus musculus GN=Cacnb4
Akr1b3	P45376	1	1	45138	122351	71351	103590	57543	87536	99524	98478	126664	190641	0.125955	0.899785	79994.6	120568.6	Up	0.59188	0.738751	Aldose reductase OS=Mus musculus GN=Akr1b1 PE=1 SV=3
Map4	P27546-4	10	10	69513	31835.33	38493.11	38487.22	61607	41784.89	33517.33	36593.33	31205.22	27052.11	0.134446	0.871452	47987.13	34030.58	Down	-0.49582	0.738751	Isoform 4 of Microtubule-associated protein 4 OS=Mus musculus GN=Map4
Stx16	Q8BVI5	3	3	76595	96060.67	98075.67	87623.67	95162.67	128993	108272.3	98514	108500	83575	0.126145	0.89913	90703.54	105570.9	Up	0.218981	0.738751	Syntaxin-16 OS=Mus musculus GN=Stx16 PE=1 SV=3
Fdxr	Q61578	1	1	287002	19145	66304	52065	143213	36509	24534	29018	12381	17186	0.134722	0.870561	113545.8	23925.6	Down	-2.24665	0.738751	NADPH:adrenodoxin oxidoreductase, mitochondrial OS=Mus musculus GN=Fdxr PE=2 SV=1
Gnai2	P08752	9	9	2064953	369491.2	1647948	895284.9	2395674	1009646	997405.4	1063516	544203	175410.3	0.135159	0.869155	1474670	758036.2	Down	-0.96005	0.738751	Guanine nucleotide-binding protein G(i) subunit alpha-2 OS=Mus musculus GN=Gnai2 PE=1 SV=5
Gnat2	P50149	4	4	4557707	753189.3	3618833	1922912	5288043	2178623	2153141	2320721	1146301	326795.8	0.135754	0.867247	3228137	1625116	Down	-0.99016	0.738751	Guanine nucleotide-binding protein G(t) subunit alpha-2 OS=Mus musculus GN=Gnat2 PE=2 SV=2
Gnai3	Q9DC51	7	7	2606377	440813.7	2074815	1110768	3030063	1254074	1240345	1333963	667305.1	198665	0.135781	0.867161	1852567	938870.4	Down	-0.98053	0.738751	Guanine nucleotide-binding protein G(k) subunit alpha OS=Mus musculus GN=Gnai3 PE=1 SV=3
Uba3	Q8C878	1	1	NA	5117	NA	2899	NA	9568	2834	10140	9294	NA	0.126175	0.899027	4008	7959	Up	0.989705	0.738751	NEDD8-activating enzyme E1 catalytic subunit OS=Mus musculus GN=Uba3 PE=1 SV=2
Tmem30a	Q8VEK0	3	3	15462	31251.67	37205	43485.33	25306.33	54055.67	39734.33	38682	36241.67	35020.67	0.127546	0.894333	30542.07	40746.87	Up	0.415892	0.738751	Cell cycle control protein 50A OS=Mus musculus GN=Tmem30a PE=1 SV=1
Gnai1	B2RSH2	8	8	2280580	386263.6	1816202	972663.3	2652022	1097893	1086022	1168056	584713.8	174713	0.135839	0.866976	1621546	822279.7	Down	-0.97967	0.738751	Guanine nucleotide-binding protein G(i) subunit alpha-1 OS=Mus musculus GN=Gnai1 PE=2 SV=1
Ube2o	Q6ZPJ3	8	8	224109.1	195977.1	243590.1	214691.4	213344.4	213041.4	212851.9	199685.5	202507	161485.6	0.136048	0.866308	218342.4	197914.3	Down	-0.14172	0.738751	Ubiquitin-conjugating enzyme E2 O OS=Mus musculus GN=Ube2o PE=1 SV=3

Rhot2	Q8JZN7	1	1	NA	31646	39816	38020	34072	38609	48883	34542	45616	39302	0.128186	0.892159	35888.5	41390.4	Up	0.205775	0.738751	Mitochondrial Rho GTPase 2 OS=Mus musculus GN=Rhot2 PE=2 SV=1
Grcc10	Q35127	4	4	71427	66181	62080.25	71750	70838.25	97379	74006.5	82695.25	70449.75	68686	0.128232	0.892004	68455.3	78643.3	Up	0.200162	0.738751	Protein C10 OS=Mus musculus GN=Grcc10 PE=2 SV=1
Sbf1	Q6ZPE2	10	10	77108	292688.3	238880.4	273060.7	234187.9	294498.4	318212.4	305895.9	278615.4	283296.6	0.128392	0.891462	223185.1	296103.8	Up	0.407862	0.738751	Myotubularin-related protein 5 OS=Mus musculus GN=Sbf1 PE=1 SV=2
Ranbp3	Q9CT10	1	1	952055	560651	551431	522869	678698	614997	487857	434670	444999	533825	0.137771	0.860842	653140.8	503269.6	Down	-0.37606	0.740187	Ran-binding protein 3 OS=Mus musculus GN=Ranbp3 PE=1 SV=2
Vdac2	Q60930	15	15	452141.9	538971.9	517327.6	670851.5	405229	486118.2	629791.1	565617.5	715085.1	737458.1	0.129231	0.888633	516904.4	626814	Up	0.27814	0.738751	Voltage-dependent anion-selective channel protein 2 OS=Mus musculus GN=Vdac2 PE=1 SV=2
Ndufs2	Q91WD5	4	4	267908.5	368589.8	304572.3	417948.3	303970.8	391064.3	370670	375764.8	404101	377190.3	0.129514	0.887683	332597.9	383758.1	Up	0.206418	0.738751	NADH dehydrogenase [ubiquinone] iron-sulfur protein 2, mitochondrial OS=Mus musculus GN=Ndufs2 PE=1 SV=1
Tuba1c	P68373	77	77	1274074	1566289	1395564	1464357	993608.2	1265279	1505051	1544713	1746334	1769372	0.129937	0.886267	1338779	1566150	Up	0.226305	0.738751	Tubulin alpha-1C chain OS=Mus musculus GN=Tuba1c PE=1 SV=1
Dnajc15	Q78YY6	1	1	722346	525978	475131	520121	525713	515390	453977	492665	443181	463474	0.139414	0.855694	553857.8	473737.4	Down	-0.22543	0.740187	DnaJ homolog subfamily C member 15 OS=Mus musculus GN=Dnajc15 PE=1 SV=1
Tuba1a	P68369	80	80	1217871	1499790	1334880	1399976	949863	1209609	1438825	1476684	1670046	1692334	0.131316	0.881682	1280476	1497500	Up	0.225875	0.738751	Tubulin alpha-1A chain OS=Mus musculus GN=Tuba1a PE=1 SV=1
Tuba1b	P05213	83	83	1193345	1465675	1301723	1366662	927126.4	1182902	1404345	1440902	1631856	1653363	0.131462	0.8812	1250906	1462674	Up	0.225634	0.738751	Tubulin alpha-1B chain OS=Mus musculus GN=Tuba1b PE=1 SV=2
Echdc1	Q9D9V3	1	1	NA	26954	18567	40334	25927	29081	NA	14520	15034	12900	0.140071	0.853652	27945.5	17883.75	Down	-0.64397	0.740187	Ethylmalonyl-CoA decarboxylase OS=Mus musculus GN=Echdc1 PE=1 SV=2
Sorbs2	Q3UTJ2-5	3	3	219351.7	234311.7	146771.7	151869.7	262511	171942.7	149672.7	149045.3	160757.7	171841.3	0.140246	0.85311	202963.1	160651.9	Down	-0.33728	0.740187	Isoform 5 of Sorbin and SH3 domain-containing protein 2 OS=Mus musculus GN=Sorbs2
Adgrb1	Q3UHD1	4	4	80361.25	349331.8	213617.8	267157.5	257100.5	334597.5	294061.3	276050.3	301593.3	388629.5	0.1317	0.880414	233513.8	318986.4	Up	0.449987	0.738751	Brain-specific angiogenesis inhibitor 1 OS=Mus musculus GN=Bai1 PE=1 SV=1
Mrps35	Q8BJZ4	3	3	504436.3	304977	372256.3	339971.3	400678.3	444811.3	324153.7	265860	244875.7	186432.7	0.140945	0.85095	384423.9	293226.7	Down	-0.39083	0.740187	28S ribosomal protein S35, mitochondrial OS=Mus musculus GN=Mrps35 PE=2 SV=2
Map7d2	A2AG50	4	4	223944.8	245089.3	198040	214376.8	254589.8	307507.3	252713.8	273672.3	223206.3	233874	0.131804	0.880071	227208.1	258194.7	Up	0.184445	0.738751	MAP7 domain-containing protein 2 OS=Mus musculus GN=Map7d2 PE=1 SV=1
Prps1	Q9D7G0	5	5	107513	196588.4	177350.2	167283.8	165080.8	184668.4	167031.8	195821	215044.6	197783.2	0.132221	0.8787	162763.2	192069.8	Up	0.238856	0.738751	Ribose-phosphate pyrophosphokinase 1 OS=Mus musculus GN=Prps1 PE=1 SV=4
Ube2v2	Q9D2M8	13	13	542779.3	374790.6	569538.6	442907.7	506747.4	246741.3	395698.4	435131.6	439926.4	481537.3	0.142912	0.844931	487352.7	399807	Down	-0.28566	0.740187	Ubiquitin-conjugating enzyme E2 variant 2 OS=Mus musculus GN=Ube2v2 PE=2 SV=4
Bcar1	Q61140	5	5	12700.33	73888.33	64342.33	63383	58710.33	68595	80217	80242.67	73518.33	71963.33	0.132838	0.876678	54604.86	74907.27	Up	0.456076	0.738751	Breast cancer anti-estrogen resistance protein 1 OS=Mus musculus GN=Bcar1 PE=1 SV=2
Ppp2r2b	Q6ZWR4-2	1	1	NA	26393	22474	25968		52520	31136	NA	26920	34985	0.132958	0.876286	24945	36390.25	Up	0.544801	0.738751	Isoform 2 of Serine/threonine-protein phosphatase 2A 55 kDa regulatory subunit B beta isoform OS=Mus musculus GN=Ppp2r2b
Sh3bgrl	Q9JJU8	2	2	400802	187578.5	274509.5	237131	317638	237988.5	263258	252945.5	176160	107242.5	0.143446	0.843312	283531.8	207518.9	Down	-0.45027	0.740187	SH3 domain-binding glutamic acid-rich-like protein OS=Mus musculus GN=Sh3bgrl PE=3 SV=1
Srgap3	Q812A2	3	3	117344.3	65441.33	73160.67	66569	98103	82365	61558.33	68763.67	65126	43718.33	0.143514	0.843106	84123.67	64306.27	Down	-0.38755	0.740187	SLIT-ROBO Rho GTPase-activating protein 3 OS=Mus musculus GN=Srgap3 PE=1 SV=1
Map2k2	Q63932	2	2	228273	309279	287652	282406	220681.5	264689.5	312516	291999.5	312305.5	319439	0.133808	0.873518	265658.3	300189.9	Up	0.176304	0.738751	Dual specificity mitogen-activated protein kinase kinase 2 OS=Mus musculus GN=Map2k2 PE=1 SV=2
Agap2	Q3UHD9	16	16	146223.8	90626.07	97434.67	91918.4	124725.1	97914.07	91874.6	99672.8	84842.47	75476.73	0.143944	0.841806	110185.6	89956.13	Down	-0.29264	0.740187	Arf-GAP with GTPase, ANK repeat and PH domain-containing protein 2 OS=Mus musculus GN=Agap2 PE=2 SV=1
Calb1	P12658	2	2	123545.5	130630	97612	108474.5	148753.5	42134.5	84425	125141.5	108433.5	[illegible]	0.144249	0.840887	121802.5	93755.5	Down	-0.37757	0.740187	Calbindin OS=Mus musculus GN=Calb1 PE=1 SV=2
Gsto1	O09131	1	1	403294	152324	259426	189324	256008	181821	214453	189670	166516	112422	0.144713	0.839492	252075.2	172976.4	Down	-0.54328	0.740187	Glutathione S-transferase omega-1 OS=Mus musculus GN=Gsto1 PE=1 SV=2
Srgap2	Q91Z67	3	3	354392.3	233690	206833.3	217428.3	279833.7	217252	221362.7	210655.3	217371	179616.3	0.145014	0.83859	258435.5	209251.5	Down	-0.30457	0.740187	SLIT-ROBO Rho GTPase-activating protein 2 OS=Mus musculus GN=Srgap2 PE=1 SV=2
Ksr2	Q3UVC0	3	3	58959	69827	91470	63555.33	78573.67	58418.33	68155.33	58544	60893	63695.67	0.145041	0.838509	72477	61941.27	Down	-0.22662	0.740187	Kinase suppressor of Ras 2 OS=Mus musculus GN=Ksr2 PE=2 SV=2
Mrps9	Q9D7N3	2	2	16709.5	88186	77341	104945.5	60405.5	89163	114039.5	85117.5	91820.5	110792	0.134356	0.871743	69517.5	98186.5	Up	0.498148	0.738751	28S ribosomal protein S9, mitochondrial OS=Mus musculus GN=Mrps9 PE=2 SV=3
Stx8	O88983	1	1	NA	5110	3913	7752	5057	6454	12672	4104	9541	8847	0.134379	0.871669	5458	8323.6	Up	0.608835	0.738751	Syntaxin-8 OS=Mus musculus GN=Stx8 PE=1 SV=1
Hcfc1	Q61191	4	4	93367	39937.25	52941.25	58471.75	99763.25	57689.5	48075.25	64441.75	39330.25	19289.25	0.145641	0.836716	68856.1	45765.2	Down	-0.59017	0.740187	Host cell factor 1 OS=Mus musculus GN=Hcfc1 PE=1 SV=2
Ssb	P32067	2	2	159578	119228.5	134399.5	127485.5	150241.5	124801.5	172053.5	148693	194649.5	168926	0.134687	0.870674	138186.6	161824.7	Up	0.227814	0.738751	Lupus La protein homolog OS=Mus musculus GN=Ssb PE=1 SV=1
Ank1	Q02357-3	4	4	35454.5	135319.8	128742.5	115092	116681.5	119654.3	136732	138302	137720	172889.5	0.135816	0.867049	106258.1	141059.6	Up	0.408732	0.738751	Isoform Er3 of Ankyrin-1 OS=Mus musculus GN=Ank1
Mtmr1	Q9Z2C4	2	2	137909	223125.5	255533	248557.5	227684	337116	251559	294727	241756	216354	0.135839	0.866976	218561.8	268302.4	Up	0.295819	0.738751	Myotubularin-related protein 1 OS=Mus musculus GN=Mtmr1 PE=2 SV=1
Rock1	P70335	1	1	525195	188071	347455	230282	362215	267373	255555	277442	194820	96965	0.145563	0.833976	330643.6	218431	Down	-0.5981	0.740187	Rho-associated protein kinase 1 OS=Mus musculus GN=Rock1 PE=1 SV=1
Hist1h1a	P43275	4	4	717638.5	492145.5	709369.5	691529	1184035	458576.3	583110.3	574608.5	671963.5	467753	0.146843	0.833147	758943.6	551202.3	Down	-0.46141	0.740187	Histone H1.1 OS=Mus musculus GN=Hist1h1a PE=1 SV=2
Clstn3	Q99JH7	1	1	191815	216506	160051	167454	180444	260260	170718	256282	233381	179424	0.136431	0.865087	183254	220013	Up	0.263744	0.739264	Calsyntenin-3 OS=Mus musculus GN=Clstn3 PE=1 SV=1
Hdac6	Q9Z2V5	1	1	919582	692823	625512	746589	634525	748262	592442	662978	550228	519655	0.147556	0.831043	723806.2	614713	Down	-0.23569	0.740187	Histone deacetylase 6 OS=Mus musculus GN=Hdac6 PE=1 SV=3
Exoc8	Q6PGF7	1	1	77248	24405	92368	50615	81622	39496	25563	53201	43147	52711	0.149317	0.825891	65251.6	42823.6	Down	-0.60761	0.740187	Exocyst complex component 8 OS=Mus musculus GN=Exoc8 PE=1 SV=1
Asna1	O54984	4	4	36682	24525	29806	34419	50268	67061	39886	66721	40033	33383	0.137067	0.863067	35140	49416.8	Up	0.491887	0.739866	ATPase Asna1 OS=Mus musculus GN=Asna1 PE=1 SV=2
Tmed10	Q9D1D4	1	1	8496	38233	21442	26044	18998	26944	28908	28967	32044	53946	0.137119	0.862902	22642.6	34161.8	Up	0.593344	0.739866	Transmembrane emp24 domain-containing protein 10 OS=Mus musculus GN=Tmed10 PE=2 SV=1
Cnp	P16330	9	9	159104.4	286876.2	429034	405199.8	229525.3	110290	252439.3	208579.8	166119.8	284350.8	0.150093	0.82364	301948	204355.9	Down	-0.56322	0.740187	2',3'-cyclic-nucleotide 3'-phosphodiesterase OS=Mus musculus GN=Cnp PE=1 SV=3
Ablim2	Q8BL65	3	3	294966.5	160506	207428.5	158248	247178	196885	167883	185544.5	162286.5	108725	0.150155	0.82346	213665.4	164264.8	Down	-0.37933	0.740187	Actin-binding LIM protein 2 OS=Mus musculus GN=Ablim2 PE=1 SV=1
Memo1	Q91VH6	1	1	123654	90441	98427	86243	98496	85051	92056	84933	95751	80382	0.150443	0.822628	99452.2	87634.6	Down	-0.1825	0.740187	Protein MEMO1 OS=Mus musculus GN=Memo1 PE=1 SV=1
Alcam	Q61490	9	9	91199.56	188463.8	165041.3	203192.3	186668.7	185590.3	203414.1	198395.3	229283.6	204010.9	0.138125	0.859728	166913.1	204138.8	Up	0.290453	0.740187	CD166 antigen OS=Mus musculus GN=Alcam PE=1 SV=3
Golga2	Q921M4	1	1	80156	64703	53346	71746	64141	73385	67305	77429	77431	81467	0.138881	0.857357	66818.4	75403.4	Up	0.174384	0.740187	Golgin subfamily A member 2 OS=Mus musculus GN=Golga2 PE=2 SV=3
Arpin	Q9D0A3	1	1	2602	3739	3788	2287	4081	4395	3366	4499	4238	3557	0.139079	0.856738	3299.4	4011	Up	0.281758	0.740187	Arpin OS=Mus musculus GN=Arpin PE=1 SV=1
Gnao1	P18872	19	19	1431372	553876.2	1365764	887697.2	1898909	918111.8	986727.7	1002485	662758.8	444483.3	0.151863	0.818548	1227524	802913.4	Down	-0.61243	0.740187	Guanine nucleotide-binding protein G(o) subunit alpha OS=Mus musculus GN=Gnao1 PE=1 SV=3
Lap3	Q9CPY7	9	9	457500.7	340513.1	359531.4	385154.9	369711.7	387242.3	351340.6	337649.7	328665.4	317980.9	0.152539	0.816619	382482.4	344575.8	Down	-0.15057	0.740187	Cytosol aminopeptidase OS=Mus musculus GN=Lap3 PE=1 SV=3
Slc25a3	Q8VEM8	2	2	NA	115743	79693.5	113108.5	89324.5	54750	98364.5	99486.5	72271.5	73335	0.152869	0.815681	99467.38	79641.5	Down	-0.3207	0.740187	Phosphate carrier protein, mitochondrial OS=Mus musculus GN=Slc25a3 PE=1 SV=1
Ccdc88a	Q5SNZ0-3	1	1	390866	271081	297580	184260	388097	312128	262498	238873	190840	139129	0.152937	0.815487	306376.8	228693.6	Down	-0.42189	0.740187	Isoform 3 of Girdin OS=Mus musculus GN=Ccdc88a
Vapa	Q9WV55	7	7	190045.7	350642.3	262259.6	278167.1	216408.9	274550.3	334732.7	275446.7	310188.7	399372.4	0.139581	0.855174	259504.7	318858.2	Up	0.297154	0.740187	Vesicle-associated membrane protein-associated protein A OS=Mus musculus GN=Vapa PE=1 SV=2
Atpif1	O35143	3	3	435793.3	523174	429342.7	551577.3	448631.3	480142.3	463880.3	439301	358153	369986	0.153365	0.814274	477703.7	422292.5	Down	-0.17787	0.740187	ATPase inhibitor, mitochondrial OS=Mus musculus GN=Atpif1 PE=1 SV=2
C77080	A2A7S8-3	1	1	50126	38858	53276	39055	70670	36253	42325	42588	32832	45575	0.153431	0.814087	50397	39914.6	Down	-0.33642	0.740187	Isoform 3 of Uncharacterized protein KIAA1522 OS=Mus musculus GN=Kiaa1522
Ap2a2	P17427	10	10	151752.6	226971.9	284898.4	241413.9	250560.1	286428.9	285887.1	279442.6	274624.6	236178	0.139717	0.854751	231119.4	272512.2	Up	0.237683	0.740187	AP-2 complex subunit alpha-2 OS=Mus musculus GN=Ap2a2 PE=1 SV=2
Fam49b	Q921M7	3	3	90507.33	177099.7	220626.3	189656	199071	193401.3	203411.7	220514.3	241985.7	226012.3	0.140817	0.851345	175392.1	217065.1	Up	0.307544	0.740187	Protein FAM49B OS=Mus musculus GN=Fam49b PE=2 SV=1
Nol3	Q9D1X0	3	3	NA	5122	4180	4312	5438	4743	5805	4733	5915	7226	0.14216	0.847223	4763	5684.4	Up	0.255138	0.740187	Nucleolar protein 3 OS=Mus musculus GN=Nol3 PE=1 SV=1
Lamtor3	O88653	1	1	60454	157420	143499	127212	148808	212529	129201	163345	150757	163112	0.142483	0.846237	127478.6	163788.8	Up	0.361582	0.740187	Ragulator complex protein LAMTOR3 OS=Mus musculus GN=Lamtor3 PE=1 SV=1
Fbxo41	Q6NS60	2	2	NA	18086	32588	25131.5	33035.5	11383	25606.5	17824.5	17580	26780	0.154096	0.812209	27210.25	19834.8	Down	-0.45612	0.740187	F-box only protein 41 OS=Mus musculus GN=Fbxo41 PE=1 SV=3
Nedd8	P29595	1	1	656266	311854	308335	315341	422443	374114	281150	295102	264226	197850	0.154177	0.81198	402847.8	282488.4	Down	-0.51204	0.740187	NEDD8 OS=Mus musculus GN=Nedd8 PE=1 SV=2
Synpo	Q8CC35-3	32	32	294158.2	261612.1	223298.4	253882.1	317214.1	265347.6	223147.3	252597.4	217360.1	240860.8	0.154376	0.81142	270033	239862.7	Down	-0.17093	0.740187	Isoform 3 of Synaptopodin OS=Mus musculus GN=Synpo
Hebp1	Q9R257	4	4	345689	528307.3	458940.8	401293.5	402728.5	548263.5	520303	467179.8	433121.3	473887	0.143301	0.843751	427391.8	488550.9	Up	0.19295	0.740187	Heme-binding protein 1 OS=Mus musculus GN=Hebp1 PE=1 SV=2
Mrpl55	Q9CZ83	1	1	512897	557829	435833	436929	431941	516530	388284	360284	341026	446270	0.154821	0.81017	475085.8	410478.8	Down	-0.21088	0.740187	39S ribosomal protein L55, mitochondrial OS=Mus musculus GN=Mrpl55 PE=2 SV=1
Snrpd3	P62320	1	1	2138171	1239366	1592693	1223595	1867798	1071810	1341775	1475127	1308498	1282177	0.154941	0.809834	1612325	1295877	Down	-0.31521	0.740187	Small nuclear ribonucleoprotein Sm D3 OS=Mus musculus GN=Snrpd3 PE=1 SV=1
Acly	Q91V92	3	3	580555.7	404212.3	438333.3	426891.7	395562	414927	425842.7	417248.3	367705	258936	0.155149	0.809251	449111	376931.8	Down	-0.25277	0.740187	ATP-citrate synthase OS=Mus musculus GN=Acly PE=1 SV=1
Tecr	Q9CY27	2	2	NA	234421	229282.5	194804.5	231613.5	206573.5	292273.5	262723	225773	320067.5	0.143339	0.843636	222530.4	261482.1	Up	0.23271	0.740187	Very-long-chain enoyl-CoA reductase OS=Mus musculus GN=Tecr PE=1 SV=1
Scrn1	Q9CZC8	3	3	1041684	1136500	1037126	1086996	1040424	1190452	1043924	1160241	1090261	1120875	0.143665	0.842649	1068546	1121151	Up	0.069331	0.740187	Secernin-1 OS=Mus musculus GN=Scrn1 PE=1 SV=1
Etfa	Q99LC5	10	10	125130.8	146109.8	117973.9	130875.3	136179.8	135795.5	131677.9	97504.25	117735.1	99177.25	0.155964	0.806976	131253.9	116378	Down	-0.17354	0.740187	Electron transfer flavoprotein subunit alpha, mitochondrial OS=Mus musculus GN=Etfa PE=1 SV=2
H1f0	P10922	2	2	931105	531660	1092329	1122013	2048253	358345.5	949225	830259	896377.5	432548	0.156634	0.805114	1145072	693351	Down	-0.72378	0.740187	Histone H1.0 OS=Mus musculus GN=H1f0 PE=2 SV=4
Dnajc3	Q91YW3	2	2	NA	7700.5	5312	7267	NA	NA	NA	5973.5	5384.5	3763.5	0.157624	0.802378	6759.833	5040.5	Down	-0.42342	0.740187	DnaJ homolog subfamily C member 3 OS=Mus musculus GN=Dnajc3 PE=1 SV=1
Tecpr1	Q80VP0	3	3	17587	81584	62430.67	77984	69373.33	91320.33	79333	88517.67	67157.67	88761.33	0.145088	0.838369	61791.8	83018	Up	0.426009	0.740187	Tectonin beta-propeller repeat-containing protein 1 OS=Mus musculus GN=Tecpr1 PE=2 SV=1
Sfpq	Q8VIJ6	10	10	724004.8	342278.2	443931.3	339694.3	645051.8	293339.7	398482.8	430747.2	390090.7	284925.5	0.158137	0.800967	498992.1	359517.2	Down	-0.47296	0.740187	Splicing factor, proline- and glutamine-rich OS=Mus musculus GN=Sfpq PE=1 SV=1
Uqcc2	Q9CQY6	1	1	452153	475759	363163	448264	422137	443482	389028	378306	358053	404140	0.158227	0.800719	432295.2	394601.8	Down	-0.13162	0.740187	Ubiquinol-cytochrome-c reductase complex assembly factor 2 OS=Mus musculus GN=Uqcc2 PE=1 SV=1
Dlg4	Q62108-3	9	9	85684	65106.2	92592.8	89902.6	88734.8	79311.8	78923.8	77251.4	69391.8	73440.2	0.158249	0.800659	84404.08	75663.8	Down	-0.15771	0.740187	Isoform 3 of Disks large homolog 4 OS=Mus musculus GN=Dlg4
Slc44a1	Q6X893	2	2	587428.5	129813.5	238423.5	220246	370513	239440	140193.5	203460	128189.5	141830	0.158254	0.800645	309284.9	170622.6	Down	-0.85813	0.740187	Choline transporter-like protein 1 OS=Mus musculus GN=Slc44a1 PE=1 SV=3
Nrxn3	Q8C985	2	2	256540	354307	387606.5	423201.5	461991	408466	295437.5	305309	283893.5	242477.5	0.158558	0.799812	376729.2	307116.7	Down	-0.29474	0.740187	Neurexin-3-beta OS=Mus musculus GN=Nrxn3 PE=1 SV=2
Prps2	Q9CS42	3	3	172591.3	274973.7	254166.3	241569.3	240309	279920.7	236099.3	263800	307989.3	266128	0.145268	0.83783	236721.9	270787.5	Up	0.193968	0.740187	Ribose-phosphate pyrophosphokinase 2 OS=Mus musculus GN=Prps2 PE=1 SV=4
Kit	P05532	1	1	NA	32603	79842	50085	105139	48970	32655	30773	56086	15824	0.158962	0.798707	66917.25	36861.6	Down	-0.86026	0.740187	Mast/stem cell growth factor receptor Kit OS=Mus musculus GN=Kit PE=1 SV=3
Mrps36	Q9CQX8	2	2	435578	270561.5	291009	266603.5	495421	325221	299801	272665	240754	193940	0.158971	0.798682	351834.6	266476.2	Down	-0.40089	0.740187	28S ribosomal protein S36, mitochondrial OS=Mus musculus GN=Mrps36 PE=1 SV=1
Slc9a3r1	P70441	2	2	33847.5	211501.5	130565.5	155741	124767	128499.5	168714.5	180145	213408.5	342325	0.146051	0.835495	131284.5	206618.5	Up	0.654273	0.740187	Na(+)/H(+) exchange regulatory cofactor NHE-RF1 OS=Mus musculus GN=Slc9a3r1 PE=1 SV=3
Itpr1	P11881-3	1	1	NA	60491	128952	42786	73338	104660	103865	142757	101863	109705	0.14627	0.834845	76391.75	112570	Up	0.559334	0.740187	Isoform 3 of Inositol 1,4,5-trisphosphate receptor type 1 OS=Mus musculus GN=Itpr1
Ddx50	Q99MJ9	2	2	14706.5	51252.5	40506	56980.5	58654.5	23630	80219.5	61379	102371	82437	0.146474	0.834239	44420	70007.3	Up	0.656296	0.740187	ATP-dependent RNA helicase DDX50 OS=Mus musculus GN=Ddx50 PE=2 SV=1
Zadh2	Q8BGC4	2	2	220584	114009.5	178911	158804	179923.5	177888.5	142105.5	139587	93638	127785	0.160023	0.795818	170446.4	136200.8	Down	-0.32358	0.740322	Zinc-binding alcohol dehydrogenase domain-containing protein 2 OS=Mus musculus GN=Zadh2 PE=1 SV=1
6-Sep	Q9R1T4-3	8	8	457116.5	238386.1	243988.5	248651.6	323427.8	277486.1	241915.6	237390.6	206583.6	183920.5	0.162054	0.79034	302314.1	229459.3	Down	-0.39781	0.745962	Isoform V of Septin-6 OS=Mus musculus GN=Sept6
Kpna3	O35344	1	1	237410	261725	259435	288246	171549	94281	242579	214350	217365	198044	0.162062	0.790319	243673	193323.8	Down	-0.33393	0.745962	Importin subunit alpha-4 OS=Mus musculus GN=Kpna3 PE=1 SV=1
Rpl29	P47915	7	7	203109.4	296757.7	167688.6	190855.7	204776.1	282086.3	221066	269757	225796.9	275187	0.146955	0.832816	212637.5	254778.6	Up	0.260848	0.740187	60S ribosomal protein L29 OS=Mus musculus GN=Rpl29 PE=2 SV=2
Nrxn3	Q6P9K9	6	6	128270	178191.3	193803.3	212952.8	230995.5	204233	149446	154198.3	143022	121238.8	0.162783	0.788391	188842.6	154427.6	Down	-0.29025	0.745962	Neurexin-3 OS=Mus musculus GN=Nrxn3 PE=1 SV=2
Mia3	Q8BI84	2	2	21537	26901	25257.5	26622.5	26895.5	25798	33054	27951	24806	30320.5	0.149343	0.825815	25442.7	28385.9	Up	0.157923	0.740187	Melanoma inhibitory activity protein 3 OS=Mus musculus GN=Mia3 PE=1 SV=2
Rufy1	Q8BIJ7	1	1	NA	89150	83054	45540	130380	38544	69503	72206	64884	27762	0.163377	0.786809	87031	54579.8	Down	-0.67316	0.745962	RUN and FYVE domain-containing protein 1 OS=Mus musculus GN=Rufy1 PE=1 SV=1
Atp6v1e2	Q9D593	1	1	4581143	2804023	3033784	2643604	2940898	3120838	2747016	2413906	2086795	2515174	0.16352	0.786429	3200690	2576746	Down	-0.31283	0.745962	V-type proton ATPase subunit E 2 OS=Mus musculus GN=Atp6v1e2 PE=2 SV=1
Bad	Q61337	1	1	NA	32678	69479	105081	71929	25007	81193	29973	35666	33216	0.164094	0.784907	69791.75	41011	Down	-0.76705	0.745962	Bcl2 antagonist of cell death OS=Mus musculus GN=Bad PE=1 SV=1
Sdhb	Q9CQA3	5	5	1735873	697975.4	1259278	1041703	1439140	1303412	933839.6	946219	768748	567678.8	0.164783	0.783088	1234794	903979.6	Down	-0.44991	0.745962	Succinate dehydrogenase [ubiquinone] iron-sulfur subunit, mitochondrial OS=Mus musculus GN=Sdhb PE=1 SV=1
Abcb9	Q9JJ59	1	1	65786	109939	189282	151661	124913	173638	140095	191511	175067	150282	0.149745	0.824648	128316.2	166118.6	Up	0.37251	0.740187	ATP-binding cassette sub-family B member 9 OS=Mus musculus GN=Abcb9 PE=2 SV=1
Smpd1	Q04519	3	3	172610.5	174274	135195.5	186872.5	164939	166161	192975.5	234135.5	179494	178332	0.15053	0.822377	166778.3	190219.6	Up	0.189734	0.740187	Sphingomyelin phosphodiesterase OS=Mus musculus GN=Smpd1 PE=2 SV=2
Tnks1bp1	P58871	2	2	1125319	722564	857796	708091	850838	875999	745741	758887	600359	561663	0.166437	0.77875	852921.6	708529.8	Down	-0.26758	0.745962	182 kDa tankyrase-1-binding protein OS=Mus musculus GN=Tnks1bp1 PE=1 SV=2
Plekha6	Q7TQG1	1	1	430306	221188	271545	275147	247678	284894	256680	209704	199215	165568	0.166675	0.77813	289172.8	223212.2	Down	-0.37352	0.745962	Pleckstrin homology domain-containing family A member 6 OS=Mus musculus GN=Plekha6 PE=1 SV=1
Dpp9	Q8BVG4-2	2	2	8943	25690.5	27873.5	33274	24048.5	25898.5	35344.5	33454	30490	31414	0.150713	0.821849	23965.9	31320.2	Up	0.38611	0.740187	Isoform 2 of Dipeptidyl peptidase 9 OS=Mus musculus GN=Dpp9
Kif1a	P33173	3	3	222392.3	212047.3	204105	180053	189055.7	221681.3	208738.3	208442	209337.3	232459.7	0.151303	0.820152	201530.7	216131.7	Up	0.100912	0.740187	Kinesin-like protein KIF1A OS=Mus musculus GN=Kif1a PE=1 SV=2
Ctsg	P28293	1	1	NA	123141	129222	118803	86802	73584	133002	75298	106162	53587	0.167441	0.776138	114492	88326.6	Down	-0.37433	0.745962	Cathepsin G OS=Mus musculus GN=Ctsg PE=1 SV=2
Man2b1	O09159	3	3	136874.7	98152.33	117436.3	115213	137828.7	137140	140673.7	149034.3	134593	116858.7	0.153142	0.814906	121101	135659.9	Up	0.163784	0.740187	Lysosomal alpha-mannosidase OS=Mus musculus GN=Man2b1 PE=2 SV=4
Cacnb2	Q8CC27-3	2	2	133731.5	178736.5	200360	173806.5	180052	179878	229880	216245.5	207943.5	162508.5	0.153466	0.813988	173337.3	199291.1	Up	0.201295	0.740187	Isoform 3 of Voltage-dependent L-type calcium channel subunit beta-2 OS=Mus musculus GN=Cacnb2
Nceh1	Q8BLF1	3	3	19639	53092.33	73656	91659	96594	101308	127174	72267.33	98455.67	73609.67	0.15376	0.813157	66928.07	94562.93	Up	0.498663	0.740187	Neutral cholesterol ester hydrolase 1 OS=Mus musculus GN=Nceh1 PE=1 SV=1
Dnaja3	Q99M87	3	3	75736.5	74322.5	72808	79038	70663.5	95970	72033.5	81955	74517.5	86217	0.153905	0.812747	74513.7	82138.6	Up	0.140555	0.740187	DnaJ homolog subfamily A member 3, mitochondrial OS=Mus musculus GN=Dnaja3 PE=1 SV=1
Dnaja3	Q99M87-2	2	2	75736.5	74322.5	72808	79038	70663.5	95970	72033.5	81955	74517.5	86217	0.153905	0.812747	74513.7	82138.6	Up	0.140555	0.740187	Isoform 2 of DnaJ homolog subfamily A member 3, mitochondrial OS=Mus musculus GN=Dnaja3
Vbp1	P61759	5	5	971172	508863.5	519656.8	519288	619503	648476.8	444147.8	459132	433411.8	373996.5	0.168478	0.773457	627696.7	471833	Down	-0.41179	0.745962	Prefoldin subunit 3 OS=Mus musculus GN=Vbp1 PE=2 SV=2
Kpnb1	P70168	3	3	228387	446462.3	487601.7	506244	487117	478186.7	557060	616472.3	502656.3	475284.7	0.154599	0.810793	431162.4	525932	Up	0.286645	0.740187	Importin subunit beta-1 OS=Mus musculus GN=Kpnb1 PE=1 SV=2
Rps14	P62264	2	2	5614.5	4621	3737.5	16699.5	4025.5	37806.5	4239	4092.5	17603.5	36929	0.155212	0.809075	6939.6	20134.1	Up	1.536717	0.740187	40S ribosomal protein S14 OS=Mus musculus GN=Rps14 PE=2 SV=3
Ap1b1	O35643	3	3	63473	93616.33	123599.7	118758	96762	110335	118516.7	127782	125226	109164.3	0.155864	0.807254	99241.8	118204.8	Up	0.252269	0.740187	AP-1 complex subunit beta-1 OS=Mus musculus GN=Ap1b1 PE=2 SV=2
D10Jhu81e	Q9D172	18	18	208194.1	173159.2	178641.1	207973.7	233529.5	227249.1	179393.2	153222.5	147841.9	152872.4	0.169139	0.771756	200299.5	172115.8	Down	-0.21878	0.745962	ES1 protein homolog, mitochondrial OS=Mus musculus GN=D10Jhu81e PE=1 SV=1
Cdc42	P60766	1	1	142030	207828	188557	228018	148693	216920	211032	196218	192271	261401	0.157727	0.802094	183025.2	215568.4	Up	0.236103	0.740187	Cell division control protein 42 homolog OS=Mus musculus GN=Cdc42 PE=1 SV=2
Ube2v1	Q9CZY3	13	13	510249.8	354919.6	568950.9	437171.2	504832.3	247104.3	392687.9	428823.3	432335	472115	0.170199	0.769043	475224.8	394613.1	Down	-0.26817	0.745962	Ubiquitin-conjugating enzyme E2 variant 1 OS=Mus musculus GN=Ube2v1 PE=1 SV=1
Itch	Q8C863	2	2	NA	17858	14093.5	13319	12384.5	11000.5	10836	16055	11914	9819	0.170366	0.768617	14413.75	11924.9	Down	-0.27347	0.745962	E3 ubiquitin-protein ligase Itchy OS=Mus musculus GN=Itch PE=1 SV=2
Fam49a	Q8BHZ0	2	2	13662.5	106003.5	116212.5	129486	115188.5	140478.5	110035	133257.5	149759	130230.5	0.158755	0.799273	96110.6	132752.1	Up	0.465967	0.740187	Protein FAM49A OS=Mus musculus GN=Fam49a PE=2 SV=1
Sestd1	Q80UK0	1	1	NA	1790	1429	1508	1520	NA	1266	2113	2664	2897	0.159127	0.798256	1561.75	2235	Up	0.517111	0.740187	SEC14 domain and spectrin repeat-containing protein 1 OS=Mus musculus GN=Sestd1 PE=2 SV=1
Grin1	P35438-2	1	1	39129	104531	124337	103703	118721	115449	137683	123438	119500	126832	0.159603	0.796959	98084.2	124580.4	Up	0.344984	0.740322	Isoform 2 of Glutamate receptor ionotropic, NMDA 1 OS=Mus musculus GN=Grin1
Tubb4a	Q9D6F9	78	78	1165282	1564404	1426535	1503851	1165829	1364335	1547308	1424066	1675081	1619778	0.159944	0.796032	1365180	1526114	Up	0.160771	0.740322	Tubulin beta-4A chain OS=Mus musculus GN=Tubb4a PE=1 SV=3
Fmn2	Q9JL04	1	1	257519	182513	183645	195772	208119	221595	171924	116122	199681	137599	0.171138	0.766654	205513.6	169384.2	Down	-0.27893	0.745962	Formin-2 OS=Mus musculus GN=Fmn2 PE=1 SV=2
Ctbp2	P56546	1	1	764943	382699	520221	525623	507071	574766	395632	418317	380600	375891	0.171522	0.76568	540111.4	429041.2	Down	-0.33214	0.746369	C-terminal-binding protein 2 OS=Mus musculus GN=Ctbp2 PE=1 SV=2
Mtx2	O88441	2	2	39274	39490	35975.5	41299	38420	20293.5	30663.5	37078.5	34926	41453	0.172193	0.763985	38891.7	32882.9	Down	-0.24212	0.747064	Metaxin-2 OS=Mus musculus GN=Mtx2 PE=1 SV=1
Clu	Q06890	6	6	115445.4	91339	74850.2	92378.2	98263.6	108914.2	101182	106548.4	95746.2	120606.6	0.162485	0.789187	94455.28	106599.5	Up	0.174497	0.745962	Clusterin OS=Mus musculus GN=Clu PE=1 SV=1
Rab11a	P62492	3	3	655499.3	290781	445730.3	315680.3	600517.7	387847	363147.7	430185.3	300968.7	165685	0.172729	0.762635	461641.7	329566.7	Down	-0.4862	0.747243	Ras-related protein Rab-11A OS=Mus musculus GN=Rab11a PE=1 SV=3
Psma7	Q9Z2U0	3	3	77380	33468.67	48594	24462.67	75297	36880	32722.33	57556.33	23397.67	5930.67	0.172889	0.762233	51840.47	31297.4	Down	-0.72804	0.747243	Proteasome subunit alpha type-7 OS=Mus musculus GN=Psma7 PE=1 SV=1
Syt5	Q9R0N5	1	1	261406	143611	266445	212783	213425	253962	284578	249266	257125	261527	0.163192	0.787301	223134	261291.6	Up	0.22775	0.745962	Synaptotagmin-5 OS=Mus musculus GN=Syt5 PE=1 SV=1
Gng13	Q9JMF3	1	1	50950	28111	42027	44184	53848	39129	42191	39297	32145	25371	0.173906	0.759685	43824	35626.6	Down	-0.29877	0.747784	Guanine nucleotide-binding protein G(I)/G(S)/G(O) subunit gamma-13 OS=Mus musculus GN=Gng13 PE=3 SV=1
Tsc2	Q61037-3	2	2	25642.5	17456	28694.5	12225.5	14795	31960.5	28403	25124	20470.5	22280.5	0.164941	0.782671	19762.7	25647.7	Up	0.376049	0.745962	Isoform B of Tuberin OS=Mus musculus GN=Tsc2
Rasal1	Q9Z268	9	9	146166.6	142250.3	147538.4	114967.7	138324.3	135202.3	143066	123534.3	113503.1	108146.3	0.174208	0.758932	137849.5	124690.4	Down	-0.14474	0.747784	RasGAP-activating-like protein 1 OS=Mus musculus GN=Rasal1 PE=2 SV=2
Syt7	Q9R0N7	2	2	357112	261848	272209.5	308068.5	281220	334840	266397	224926	222989	226263	0.175719	0.755181	296091.6	255083	Down	-0.21508	0.747784	Synaptotagmin-7 OS=Mus musculus GN=Syt7 PE=1 SV=1
Ogdh	Q60597-3	15	15	256475.2	245995.2	215996.7	301555.1	226712.9	222675	241214.7	210401.9	224298.4	224437.3	0.1762	0.753994	249347	224605.5	Down	-0.15076	0.747784	Isoform 3 of 2-oxoglutarate dehydrogenase, mitochondrial OS=Mus musculus GN=Ogdh
Sptan1	P16546	144	144	577731.9	548633.1	551585.9	624950.7	624420.3	594074.5	560854.1	541748.4	547016.9	533689.2	0.176463	0.753346	585464.4	555476.6	Down	-0.07586	0.747784	Spectrin alpha chain, non-erythrocytic 1 OS=Mus musculus GN=Sptan1 PE=1 SV=4
Lmnb1	P14733	3	3	75602.67	54561	60643	62061	82260.33	44401.67	59995.67	67760.67	64492	46871.33	0.176529	0.753184	67025.6	56704.27	Down	-0.24125	0.747784	Lamin-B1 OS=Mus musculus GN=Lmnb1 PE=1 SV=3
Gbe1	Q9D6Y9	1	1	49645	112583	129234	94138	101765	143194	137565	127432	95784	107774	0.165473	0.781273	97473	122349.8	Up	0.327937	0.745962	1,4-alpha-glucan-branching enzyme OS=Mus musculus GN=Gbe1 PE=2 SV=1
Stac2	Q8R1B0	1	1	339760	39314	100991	67706	181005	23162	84820	84658	53343	38344	0.177051	0.751902	145755.2	56865.4	Down	-1.35792	0.747784	SH3 and cysteine-rich domain-containing protein 2 OS=Mus musculus GN=Stac2 PE=2 SV=1
Diaph2	O70566	1	1	72577	154489	131774	136564	157877	167589	139148	155200	165834	156922	0.166745	0.777947	130656.2	156938.6	Up	0.264425	0.745962	Protein diaphanous homolog 2 OS=Mus musculus GN=Diaph2 PE=1 SV=2
Uros	P51163	1	1	19020	18200	15472	13990	13667	20167	18552	14314	18628	20402	0.167334	0.776416	16069.8	18412.6	Up	0.196341	0.745962	Uroporphyrinogen-III synthase OS=Mus musculus GN=Uros PE=2 SV=1
Pir	Q9D711	1	1	1184688	632541	857758	652194	756554	723001	703220	685649	588425	551823	0.178527	0.748296	816747	652423.6	Down	-0.32851	0.745962	Pirin OS=Mus musculus GN=Pir PE=2 SV=1
Exoc7	O35250	1	1	NA	81194	62848	63445	118585	60190	80128	41717	73459	27630	0.178774	0.747696	81518	56624.8	Down	-0.52568	0.747784	Exocyst complex component 7 OS=Mus musculus GN=Exoc7 PE=1 SV=2
Anxa7	Q07076	1	1	591936	272835	332150	203592	272318	245946	182821	276753	216034	198702	0.179417	0.746136	334566.2	224051.2	Down	-0.57846	0.747784	Annexin A7 OS=Mus musculus GN=Anxa7 PE=2 SV=2
Nedd4	P46935	1	1	239192	188193	274140	201661	287810	181660	226403	217913	219318	179152	0.17979	0.745234	238199.2	204889.2	Down	-0.21732	0.747784	E3 ubiquitin-protein ligase NEDD4 OS=Mus musculus GN=Nedd4 PE=1 SV=3
Slc25a11	Q9CR62	2	2	318121	214801	253862.5	253864.5	286535	259948	248222	229698.5	241713.5	191324	0.180234	0.744163	265436.8	234181.2	Down	-0.18074	0.747784	Mitochondrial 2-oxoglutarate/malate carrier protein OS=Mus musculus GN=Slc25a11 PE=1 SV=3
Gnas	P63094-3	1	1	NA	56782	56280	76324	50549	69138	70417	68028	77534	65764	0.167581	0.775775	59983.75	70176.2	Up	0.22641	0.745962	Isoform Gnas-3 of Guanine nucleotide-binding protein G(s) subunit alpha isoforms short OS=Mus musculus GN=Gnas
Bsdc1	Q80Y55	1	1	NA	42190	17225	37195	39110	92695	66491	44563	33641	32751	0.168109	0.774409	33930	54028.2	Up	0.671151	0.745962	BSD domain-containing protein 1 OS=Mus musculus GN=Bsdc1 PE=2 SV=1
Ppfia2	Q8BSS9	7	7	81976.43	84690.14	72191.43	69303	82010.86	95837.14	78133.86	89831.29	83154.71	78158.14	0.168172	0.774246	78034.37	85023.03	Up	0.123744	0.745962	Liprin-alpha-2 OS=Mus musculus GN=Ppfia2 PE=1 SV=2
Pacsin1	Q61644	25	25	176430.3	130713.9	102610.4	107212.5	136534.4	138856.4	101131	109563.5	100092.3	86428.05	0.181034	0.74224	130700.3	107214.2	Down	-0.28577	0.747784	Protein kinase C and casein kinase substrate in neurons protein 1 OS=Mus musculus GN=Pacsin1 PE=1 SV=1
2310035C	Q148V7-3	1	1	142858	164793	206803	169443	174054	182485	217494	237348	227684	145804	0.168196	0.774184	171590.2	202163	Up	0.236552	0.745962	Isoform 3 of LisH domain and HEAT repeat-containing protein KIAA1468 OS=Mus musculus GN=Kiaa1468
Bpgm	P15327	1	1	138212	74976	76402	94955	136488	102104	82341	78245	66387	75769	[illegible]	[illegible]	104206.6	80969.2	Down	-0.364	0.747784	Bisphosphoglycerate mutase OS=Mus musculus GN=Bpgm PE=2 SV=3
Dnm3	Q8BZ98	11	11	1043224	621172.6	876900	716950.5	785894.6	690045.4	823417.1	643709.6	720117.5	521074.5	0.182297	0.73922	808828.3	679672.8	Down	-0.25099	0.747784	Dynamin-3 OS=Mus musculus GN=Dnm3 PE=1 SV=1
Hdac4	Q6NZM9	1	1	169935	376539	412782	310193	356618	377247	380860	415452	358769	457640	0.168385	0.773697	325213.4	397993.6	Up	0.291359	0.745962	Histone deacetylase 4 OS=Mus musculus GN=Hdac4 PE=1 SV=1
Astn1	Q61137	15	15	50906.82	134166.8	136726.9	130205	130810.6	131888.7	150174.6	137909.3	149392.4	152403.3	0.168612	0.773112	116563.2	144353.7	Up	0.308495	0.745962	Astrotactin-1 OS=Mus musculus GN=Astn1 PE=2 SV=4
Fabp5	Q05816	2	2	NA	19667	42338	33958	31732	29567	23362	29977.5	16488	18186.5	0.18317	0.737146	31923.75	23516.2	Down	-0.44098	0.747784	Fatty acid-binding protein, epidermal OS=Mus musculus GN=Fabp5 PE=1 SV=3
15-Sep	Q9ERR7	1	1	NA	12496	15097	28375	9588	NA	33861	56403	10044	34202	0.168729	0.77281	16389	33627.5	Up	1.036914	0.745962	15 kDa selenoprotein OS=Mus musculus GN=Sep15 PE=1 SV=3
Slc30a9	Q5IRJ6	1	1	NA	24373	33912	44672	11732	30950	50325	35724	46799	43895	0.168777	0.772687	28672.25	41538.6	Up	0.534797	0.745962	Zinc transporter 9 OS=Mus musculus GN=Slc30a9 PE=1 SV=1
Glud1	P26443	14	14	485608.3	400290.5	367213.5	486767	384207.3	364103.2	418835.3	350220.2	374901.8	401525.5	0.18434	0.73438	424817.3	381917.2	Down	-0.15358	0.747784	Glutamate dehydrogenase 1, mitochondrial OS=Mus musculus GN=Glud1 PE=1 SV=1
Phb2	O35129	7	7	483352.3	595410	468145.5	605570.3	476841.7	486047.2	491499.5	478643	496201.8	423854.3	0.184605	0.733757	525864.1	475249.2	Down	-0.14601	0.747784	Prohibitin-2 OS=Mus musculus GN=Phb2 PE=1 SV=1
Rgs14	P97492	2	2	46794	81124	95002	59276	101120	66292	59389	75992	55924	33672	0.184925	0.733004	76663.2	58253.8	Down	-0.39618	0.747784	Regulator of G-protein signaling 14 OS=Mus musculus GN=Rgs14 PE=1 SV=2
Anxa3	O35639	1	1	NA	59392	NA	30888	46468	59689	127147	34977	51008	111429	0.170015	0.769513	45582.67	76850	Up	0.75356	0.745962	Annexin A3 OS=Mus musculus GN=Anxa3 PE=1 SV=4
Luc7l3	Q5SUF2-3	1	1	NA	16200	63288	41258	64124	35100	23205	39797	16793	19233	0.187585	0.726802	46217.5	26825.6	Down	-0.78483	0.747784	Isoform 3 of Luc7-like protein 3 OS=Mus musculus GN=Luc7l3
Otud6b	Q8K2H2	1	1	151669	111903	109532	79659	102573	115385	89942	91008	80830	79869	0.187804	0.726295	111067.2	91406.8	Down	-0.28106	0.747784	OTU domain-containing protein 6B OS=Mus musculus GN=Otud6b PE=2 SV=1
Cad	B2RQC6	1	1	NA	60228	90657	89372	79015	106323	69770	132396	92254	93680	0.170679	0.76782	79818	98884.6	Up	0.309032	0.745962	CAD protein OS=Mus musculus GN=Cad PE=2 SV=1
Ocrl	Q6NVF0	2	2	75824	206718.5	129807.5	152961	158293.5	202328.5	154653.5	183637	166179	199889.5	0.170807	0.767494	144720.9	181337.5	Up	0.325404	0.745962	Inositol polyphosphate 5-phosphatase OCRL-1 OS=Mus musculus GN=Ocrl PE=1 SV=1

Stxbp5l	Q5DQR4-4	3	3	NA	18564	23383.5	18086.5	17300	21427.5	29632.5	16811.5	23284.5	24769.5	0.171014	0.766968	19333.5	23185.1	Up	0.262095	0.745962	Isoform 4 of Syntaxin-binding protein 5-like OS=Mus musculus GN=Stxbp5l
Os9	Q8K2C7	1	1	31869	21656	25618	21528	24506	9928	25428	10147	27568	21054	0.189207	0.723063	25035.4	18825	Down	-0.41162	0.747784	Protein OS-9 OS=Mus musculus GN=Os9 PE=1 SV=2
Uchl1	Q9R0P9	7	7	812518.7	386598.6	478646.6	378809.1	508960.1	480412.1	412648	392351.6	363180.7	272053.3	0.18954	0.722299	513106.6	384129.1	Down	-0.41767	0.747784	Ubiquitin carboxyl-terminal hydrolase isozyme L1 OS=Mus musculus GN=Uchl1 PE=1 SV=1
Cct5	P80316	13	13	237367.5	83108.62	137010.1	100216.1	152955	130475.4	110058.8	102012.9	86208.75	62899.12	0.189637	0.722077	142131.5	98330.98	Down	-0.53151	0.747784	T-complex protein 1 subunit epsilon OS=Mus musculus GN=Cct5 PE=1 SV=1
Iscu	Q9D7P6	1	1	697656	528195	440113	492790	419225	500987	472463	323083	458834	394488	0.189709	0.721912	515595.8	429971	Down	-0.262	0.747784	Iron-sulfur cluster assembly enzyme ISCU, mitochondrial OS=Mus musculus GN=Iscu PE=1 SV=2
Plppr4	Q7TME0	4	4	213977.3	197229.3	226043.3	159401	258947.3	240986	154115.3	187041	166241	124714	0.190017	0.721208	211119.7	174619.5	Down	-0.27385	0.747784	Lipid phosphate phosphatase-related protein type 4 OS=Mus musculus GN=Lppr4 PE=2 SV=2
Stxbp5l	Q5DQR4-6	2	2	NA	18564	23383.5	18086.5	17300	21427.5	29632.5	16811.5	23284.5	24769.5	0.171014	0.766968	19333.5	23185.1	Up	0.262095	0.745962	Isoform 6 of Syntaxin-binding protein 5-like OS=Mus musculus GN=Stxbp5l
Neo1	P97798-3	4	4	62711.67	98229	95911.33	89940.67	73606.67	105599.7	81514.67	102114	92516.67	100487.3	0.172265	0.763803	84079.87	96446.47	Up	0.197968	0.747064	Isoform 3 of Neogenin OS=Mus musculus GN=Neo1
Slc1a3	P56564	3	3	NA	43156	73118	81498	47719	58280	78578	80874	86403	89144	0.17328	0.761252	61372.75	78655.8	Up	0.357955	0.747672	Excitatory amino acid transporter 1 OS=Mus musculus GN=Slc1a3 PE=1 SV=2
Map7d2	A2AG50-2	3	3	142525	214134.7	156950.3	197982.3	206443.3	224387.3	193289	217485.7	196167	207692.7	0.174087	0.759234	183607.1	207804.3	Up	0.178604	0.747784	Isoform 2 of MAP7 domain-containing protein 2 OS=Mus musculus GN=Map7d2
Ppp1r9b	Q6R891	8	8	154920.8	140067.2	118282.5	102755.8	168657	118526.5	101004.2	132215.8	123274	112747	0.19237	0.715863	136936.7	117553.5	Down	-0.22019	0.747784	Neurabin-2 OS=Mus musculus GN=Ppp1r9b PE=1 SV=1
Spry2	Q9QXV8	1	1	NA	142382	150397	151924	160120	212391	207992	133781	157890	167974	0.176824	0.752459	151205.8	176005.6	Up	0.219108	0.747784	Protein sprouty homolog 2 OS=Mus musculus GN=Spry2 PE=2 SV=1
Hist1h1b	P43276	3	3	1162666	786640.7	1629565	1093960	2451756	660170.7	1261776	1135630	944318.7	799026.7	0.19269	0.715141	1424917	960184.5	Down	-0.56949	0.747784	Histone H1.5 OS=Mus musculus GN=Hist1h1b PE=1 SV=2
Homer2	Q9QWW1	7	7	45866.29	44660.29	64881.71	56146.86	64004	45286.29	46441	53194	50368.86	45908.43	0.192696	0.715127	55111.83	48239.72	Down	-0.19214	0.747784	Homer protein homolog 2 OS=Mus musculus GN=Homer2 PE=1 SV=1
Shisa6	Q3UH99	2	2	NA	139514	115178	130525	151013	190097.5	123718.5	165455.5	139268.5	154515	0.177523	0.750745	134057.5	154611	Up	0.205791	0.747784	Protein shisa-6 homolog OS=Mus musculus GN=Shisa6 PE=1 SV=1
Clasp2	Q8BRT1	8	8	233804.7	176417.3	203746.9	181044.9	251250.3	188091.4	174466.7	206999.6	183245.1	176140.1	0.193478	0.713368	209252.8	185786.6	Down	-0.17158	0.747784	CLIP-associating protein 2 OS=Mus musculus GN=Clasp2 PE=1 SV=1
Fermt3	Q8K1B8	1	1	314095	262430	241106	271385	207218	211024	211243	281696	190025	234791	0.193726	0.712812	259246.8	225755.8	Down	-0.19956	0.747784	Fermitin family homolog 3 OS=Mus musculus GN=Fermt3 PE=1 SV=1
Rad23b	P54728	3	3	NA	90492.33	76016.67	92023.33	79473	80174.33	86692	102571.3	99817.67	96615	0.178442	0.748503	84501.33	93174.07	Up	0.140954	0.747784	UV excision repair protein RAD23 homolog B OS=Mus musculus GN=Rad23b PE=1 SV=1
Pdpk1	Q9Z2A0	2	2	NA	81217	76817	96460	100011	37032	81753	110562	53657	56230	0.194562	0.710942	88626.25	67846.8	Down	-0.38545	0.747784	3-phosphoinositide-dependent protein kinase 1 OS=Mus musculus GN=Pdpk1 PE=1 SV=2
Slc20a2	Q80UP8	1	1	NA	7971	14998	25724	21180	11969	13409	7869	10680	11389	0.195027	0.709905	17468.25	11063.2	Down	-0.65897	0.747784	Sodium-dependent phosphate transporter 2 OS=Mus musculus GN=Slc20a2 PE=1 SV=2
Glo1	Q9CPU0	4	4	68436	50263.25	53954.75	54185.5	51872.25	58952.75	57700.75	50645.75	45299.25	39816.25	0.195599	0.708633	55742.35	49282.95	Down	-0.17769	0.747784	Lactoylglutathione lyase OS=Mus musculus GN=Glo1 PE=1 SV=3
1700025G	Q8K207	1	1	1306177	446368	630335	497649	803172	696675	510016	587365	374859	247673	0.196131	0.707454	736740.2	483317.6	Down	-0.60818	0.747784	Uncharacterized protein C1orf21 homolog OS=Mus musculus PE=1 SV=1
Rpl5	P47962	6	6	467690.3	517508.5	370777	407432.2	298313.2	544446.5	426789.2	456018.2	454988.7	506858.7	0.180628	0.743215	412344.2	477820.2	Up	0.212619	0.747784	60S ribosomal protein L5 OS=Mus musculus GN=Rpl5 PE=1 SV=3
Gpx4	O70325	1	1	129448	141909	98918	173275	172368	108602	133334	151466	122215	96133	0.19683	0.705909	143183.6	122350	Down	-0.58228	0.747784	Phospholipid hydroperoxide glutathione peroxidase, mitochondrial OS=Mus musculus GN=Gpx4 PE=1 SV=4
Grpel1	Q99LP6	3	3	990234.3	565236.7	733148	586682	624003.7	637068	570522.3	603868.3	588110.3	486074	0.19731	0.704851	699860.9	577128.6	Down	-0.27818	0.747784	GrpE protein homolog 1, mitochondrial OS=Mus musculus GN=Grpel1 PE=1 SV=1
Pacsin3	Q99JB8	1	1	294801	108422	182333	109846	175835	171570	125446	131815	92443	74315	0.197379	0.704699	174247.4	119117.8	Down	-0.54875	0.747784	Protein kinase C and casein kinase II substrate protein 3 OS=Mus musculus GN=Pacsin3 PE=1 SV=1
Zfyve19	Q9DAZ9	1	1	36192	32715	30458	27057	34746	37705	32337	34834	33970	36317	0.180687	0.743073	32233.6	35032.6	Up	0.120133	0.747784	Zinc finger FYVE domain-containing protein 19 OS=Mus musculus GN=Zfyve19 PE=1 SV=2
Adgrl1	Q80TR1-2	8	8	72268.17	235270	228736.3	217167.2	270439.8	303289.5	259435.5	251225	257885.3	234032.2	0.180994	0.742336	204776.3	261173.5	Up	0.35096	0.747784	Isoform 2 of Latrophilin-1 OS=Mus musculus GN=Lphn1
Ppia	P17742	28	28	2155467	1446129	1422132	1570995	1887116	1833631	1452582	1491637	1185478	1233052	0.198063	0.703197	1696368	1439276	Down	-0.23711	0.747784	Peptidyl-prolyl cis-trans isomerase A OS=Mus musculus GN=Ppia PE=1 SV=2
Wars	P32921	5	5	219769.6	204662.4	221036.4	202110.4	195758.6	192212.6	219298	221899	264361.2	250825	0.18113	0.74201	208667.5	229719.2	Up	0.138665	0.747784	Tryptophan--tRNA ligase, cytoplasmic OS=Mus musculus GN=Wars PE=1 SV=2
Vcan	Q62059-3	2	2	269087	59747	174689	44307	263152	109683	90765	117837	67050	51953	0.198301	0.702675	162196.4	87457.6	Down	-0.89109	0.747784	Isoform V2 of Versican core protein OS=Mus musculus GN=Vcan
Prkcb	P68404	3	3	36210.33	77454.33	68180.67	73416.67	101872.7	104883.7	79622.67	108513	94099	66561.67	0.182894	0.737801	71426.93	90736	Up	0.345207	0.747784	Protein kinase C beta type OS=Mus musculus GN=Prkcb PE=1 SV=4
Rbm15b	Q6PHZ5	2	2	34642.5	24928.5	19742	32714	37750.5	31011	23513	31429	16088	11039.5	0.198912	0.701339	29955.5	22616.1	Down	-0.40547	0.747784	Putative RNA-binding protein 15B OS=Mus musculus GN=Rbm15b PE=1 SV=2
Slc3a2	P10852	9	9	22659.75	351109.3	379428.4	392493.3	349110.8	468072.1	396498.4	407979.8	414970.1	370279.8	0.183092	0.737331	298960.3	411560	Up	0.461149	0.747784	4F2 cell-surface antigen heavy chain OS=Mus musculus GN=Slc3a2 PE=1 SV=1
4931406C	Q91V76	2	2	93770	210729.5	117984.5	161889.5	115608.5	165994	152602.5	143490.5	205512	222449.5	0.183559	0.736224	139996.4	178009.7	Up	0.346566	0.747784	Ester hydrolase C11orf54 homolog OS=Mus musculus PE=2 SV=1
6430548M	Q8R0A7	2	2	203890	355846.5	360808.5	355492.5	307461	361190	377415	324580.5	365377.5	401408.5	0.184054	0.735055	316699.7	365994.3	Up	0.208706	0.747784	Uncharacterized protein KIAA0513 OS=Mus musculus GN=Kiaa0513 PE=2 SV=1
Ankrd34b	Q3UUF8	1	1	NA	102868	81220	46356	115922	35973	65128	91802	57442	51866	0.199987	0.698998	86591.5	60442.2	Down	-0.51867	0.747784	Ankyrin repeat domain-containing protein 34B OS=Mus musculus GN=Ankrd34b PE=1 SV=1
Cfl1	P18760	22	22	537152.2	210097.9	229979.8	247751.9	345164.4	298986.1	218372.2	237795.1	196168.4	140011.7	0.200662	0.697535	314029.2	218266.7	Down	-0.52481	0.747784	Cofilin-1 OS=Mus musculus GN=Cfl1 PE=1 SV=3
Epb41l3	Q9WV92-7	6	6	72576	94334.67	53035	87581	90072	97382	90465.33	79928.33	90156.33	103200	0.186421	0.729505	79519.73	92226.4	Up	0.213867	0.747784	Isoform 7 of Band 4.1-like protein 3 OS=Mus musculus GN=Epb41l3
Capza1	P47753	2	2	599348	159687	290236	187722	431531	337511	239874	234911	124270	53018	0.200909	0.697001	333704.8	197916.8	Down	-0.75368	0.747784	F-actin-capping protein subunit alpha-1 OS=Mus musculus GN=Capza1 PE=1 SV=4
Sst	P60041	4	4	1637042	496944.5	875546.5	423726.5	1027275	660110.5	698135.5	663590.5	473683.5	255805.5	0.201055	0.696685	892106.8	550265.1	Down	-0.69709	0.747784	Somatostatin OS=Mus musculus GN=Sst PE=2 SV=1
Mmab	Q9D273	2	2	295236	413112.5	326709.5	426630.5	348971.5	410785	423723.5	389560.5	397816	390073	0.188044	0.725741	362132	402391.6	Up	0.152085	0.747784	Cob(I)yrinic acid a,c-diamide adenosyltransferase, mitochondrial OS=Mus musculus GN=Mmab PE=1 SV=1
Tubb2a	Q7TMM9	96	96	953089.6	1404759	1430303	1319747	1195578	1244582	1373372	1418735	1512604	1486539	0.189002	0.723534	1260695	1407167	Up	0.158574	0.747784	Tubulin beta-2A chain OS=Mus musculus GN=Tubb2a PE=1 SV=1
Tpm4	Q6IRU2	1	1	869292	379091	501041	471502	634740	547045	436956	433683	403778	372579	0.202086	0.694464	571133.2	438808.2	Down	-0.38024	0.747784	Tropomyosin alpha-4 chain OS=Mus musculus GN=Tpm4 PE=2 SV=3
Arhgap32	Q811P8	5	5	206280.6	241875.2	233650.8	217377	225651	217617.8	220589.8	211382.6	199350.6	223094.4	0.202148	0.694335	224966.9	214407	Down	-0.06936	0.747784	Rho GTPase-activating protein 32 OS=Mus musculus GN=Arhgap32 PE=1 SV=2
Atp8a1	P70704	1	1	NA	11792	23015	27177	23319	19192	28658	22738	37737	32516	0.189132	0.723235	21325.75	28168.2	Up	0.401471	0.747784	Probable phospholipid-transporting ATPase IA OS=Mus musculus GN=Atp8a1 PE=1 SV=1
St13	Q99L47	2	2	194346	220530	276010.5	222046	313138	299593.5	161305.5	214710.5	171386	105956.5	0.202417	0.693753	245214.1	190590.4	Down	-0.36357	0.747784	Hsc70-interacting protein OS=Mus musculus GN=St13 PE=1 SV=1
7-Sep	O55131	28	28	157720	148118	126635.4	158194.4	165456	149055.3	140177.2	134727.6	141343.3	138484.2	0.202779	0.692977	151224.8	140757.5	Down	-0.10348	0.747784	Septin-7 OS=Mus musculus GN=Sept7 PE=1 SV=1
Mocs3	A2BDX3	1	1	274671	108757	165822	111234	160703	157142	104181	128762	96276	100150	0.203117	0.692254	164237.4	117302.2	Down	-0.48555	0.747784	Adenylyltransferase and sulfurtransferase MOCS3 OS=Mus musculus GN=Mocs3 PE=2 SV=1
Itgb2	P11835	1	1	NA	31236	27800	71285	41460	NA	32068	84053	71959	73118	0.190049	0.721134	42945.25	65299.5	Up	0.604573	0.747784	Integrin beta-2 OS=Mus musculus GN=Itgb2 PE=1 SV=2
5-Sep	Q9Z2Q6	10	10	713797	447768.3	317083.4	409769.4	476697.6	373128.4	381930.9	310221.6	435465.9	355582.1	0.203157	0.692168	473023.1	371265.8	Down	-0.34946	0.747784	Septin-5 OS=Mus musculus GN=Sept5 PE=1 SV=1
Mydgf	Q9CPT4	1	1	79695	32194	69456	66170	36241	52397	21093	29784	49096	51105	0.203293	0.691878	56751.2	40695	Down	-0.4798	0.747784	UPF0556 protein C19orf10 homolog OS=Mus musculus GN=D17Wsu104e PE=2 SV=1
Kcnq2	Q9Z351-1	1	1	NA	58700	16672	66765	53110	38798	52985	96155	75449	96957	0.190351	0.720445	48811.75	72068.8	Up	0.562146	0.747784	Isoform 13 of Potassium voltage-gated channel subfamily KQT member 2 OS=Mus musculus GN=Kcnq2
Mdh1	P14152	21	21	681261.1	280531	390578	327929.6	456834	392131.2	348803.2	328262.9	310587	205335.3	0.203907	0.690568	427426.7	317023.9	Down	-0.43109	0.747784	Malate dehydrogenase, cytoplasmic OS=Mus musculus GN=Mdh1 PE=1 SV=3
Uqcrh	P99028	2	2	12695.5	NA	8649	50744	12210	8107	5148	3620.5	3150	NA	0.203997	0.690376	21074.63	5006.375	Down	-2.07367	0.747784	Cytochrome b-c1 complex subunit 6, mitochondrial OS=Mus musculus GN=Uqcrh PE=1 SV=2
Ndufa2	Q9CQ75	3	3	647826.3	467302.7	408833.3	468987.7	458114.3	549800.7	415900.3	412577	311654.3	368595.7	0.204029	0.690308	490212.9	411705.6	Down	-0.2518	0.747784	NADH dehydrogenase [ubiquinone] 1 alpha subcomplex subunit 2 OS=Mus musculus GN=Ndufa2 PE=1 SV=3
Aldh4a1	Q8CHT0	3	3	83556	112711	118327.5	107703	128564.5	89619	114340.5	110785.5	76301	87695	0.204322	0.689685	110172.4	95748.2	Down	-0.20245	0.747784	Delta-1-pyrroline-5-carboxylate dehydrogenase, mitochondrial OS=Mus musculus GN=Aldh4a1 PE=1 SV=3
Tubb2b	Q9CWF2	98	98	925241	1364908	1391357	1283336	1159861	1207005	1334620	1377375	1471446	1445881	0.191422	0.718008	1224940	1367266	Up	0.158582	0.747784	Tubulin beta-2B chain OS=Mus musculus GN=Tubb2b PE=1 SV=1
Eno3	P21550	25	25	247049.3	314758.7	246224.3	276719.8	224778.6	294863.5	298320.3	279277	298698.4	264683.8	0.191842	0.717056	261906.1	287168.6	Up	0.132848	0.747784	Beta-enolase OS=Mus musculus GN=Eno3 PE=1 SV=3
Atp6v1g2	Q9WTT4	5	5	380871.3	280076.3	262422.5	279816.5	244421.3	326243.8	234329	240969	228698.3	195019.3	0.205417	0.687364	289521.6	245051.9	Down	-0.24058	0.747784	V-type proton ATPase subunit G 2 OS=Mus musculus GN=Atp6v1g2 PE=1 SV=1
Gdap1l1	Q8VE33	2	2	NA	15425	7552	8349	5348	11382	23043	NA	21670	7390	0.192647	0.715238	9168.5	15871.25	Up	0.791658	0.747784	Ganglioside-induced differentiation-associated protein 1-like 1 OS=Mus musculus GN=Gdap1l1 PE=1 SV=1
Osbpl6	Q8BXR9	2	2	19854	21250.5	19882	16063.5	23958.5	16928.5	23226.5	34484.5	23992.5	25260	0.193463	0.713402	20201.7	24778.4	Up	0.294606	0.747784	Oxysterol-binding protein-related protein 6 OS=Mus musculus GN=Osbpl6 PE=2 SV=1
Ube2v2	Q9D2M8-2	12	12	514497.5	382766.6	609199.3	471380.3	536523.6	240730.1	414949.1	454258.5	462263.6	523023.4	0.206794	0.684462	502873.4	419044.9	Down	-0.26309	0.747784	Isoform 2 of Ubiquitin-conjugating enzyme E2 variant 2 OS=Mus musculus GN=Ube2v2
Tppp3	Q9CRB6	2	2	529279	418279.5	392106	506341	408521.5	480347	390552.5	420543.5	373090.5	336008.5	0.20726	0.683485	450905.4	400108.4	Down	-0.17243	0.747784	Tubulin polymerization-promoting protein family member 3 OS=Mus musculus GN=Tppp3 PE=1 SV=1
Twf2	Q9Z0P5	1	1	10487	4923	5056	4840	6884	5646	3700	5296	4586	4714	0.207355	0.683285	6438	4788.4	Down	-0.42707	0.747784	Twinfilin-2 OS=Mus musculus GN=Twf2 PE=1 SV=1
Apoc1	P34928	1	1	964373	371764	385437	358125	367893	323075	349745	300225	226886	351828	0.20794	0.682062	489518.4	310351.8	Down	-0.65746	0.747784	Apolipoprotein C-I OS=Mus musculus GN=Apoc1 PE=1 SV=1
Sod2	P09671	4	4	43083.67	82073	82675.67	100129.7	122840.7	95158.33	106906	116190	122429.7	95261.67	0.194529	0.711016	86160.54	107189.1	Up	0.31506	0.747784	Superoxide dismutase [Mn], mitochondrial OS=Mus musculus GN=Sod2 PE=1 SV=3
Txndc12	Q9CQU0	2	2	105609	269217	186448	191949	136650	192898	228145	221681	206717	263543	0.196618	0.706377	177974.6	222596.8	Up	0.322761	0.747784	Thioredoxin domain-containing protein 12 OS=Mus musculus GN=Txndc12 PE=2 SV=1
Adcy1	O88444	2	2	79371	274568	242680.5	239555	256970	244494	257404.5	344550.5	259164.5	274697	0.197758	0.703866	218628.9	276062.1	Up	0.336509	0.747784	Adenylate cyclase type 1 OS=Mus musculus GN=Adcy1 PE=2 SV=2
Hmgn2	P09602	2	2	48146	18040	25055	33833	59018	24905	32204	29205.5	19296	21604	0.20958	0.67865	36818.4	25442.9	Down	-0.53316	0.747784	Non-histone chromosomal protein HMG-17 OS=Mus musculus GN=Hmgn2 PE=1 SV=2
Tubb6	Q922F4	37	37	945245.1	2104353	2141756	1938250	1741288	1851140	1961756	2203992	2225833	2379016	0.197785	0.703807	1774179	2124347	Up	0.259868	0.747784	Tubulin beta-6 chain OS=Mus musculus GN=Tubb6 PE=1 SV=1
Atp5c1	Q91VR2	4	4	129199	81819.25	72813	135029.3	76806.75	70689.5	84528.75	69165.75	92754.25	75326.5	0.209796	0.678203	99133.45	78492.95	Down	-0.33681	0.747784	ATP synthase subunit gamma, mitochondrial OS=Mus musculus GN=Atp5c1 PE=1 SV=1
Phactr1	Q2M3X8	4	4	93554.5	111029.5	83543.25	76289.5	66908	113181.5	91244.25	92496.25	88215.25	112261	0.198262	0.702761	86264.95	99479.65	Up	0.205627	0.747784	Phosphatase and actin regulator 1 OS=Mus musculus GN=Phactr1 PE=1 SV=1
Mcts1	Q9DB27	1	1	70701	57810	51208	47603	46018	54482	67180	49177	74200	96511	0.198504	0.702231	54668	68310	Up	0.3214	0.747784	Malignant T-cell-amplified sequence 1 OS=Mus musculus GN=Mcts1 PE=2 SV=1
Sorbs2	Q3UTJ2	6	6	139724.3	153322.8	92988	103796.7	174272.2	122604.8	100323.8	107345	109290.3	110439.3	0.210659	0.67642	132820.8	110000.7	Down	-0.27197	0.747784	Sorbin and SH3 domain-containing protein 2 OS=Mus musculus GN=Sorbs2 PE=1 SV=2
Idi1	P58044	2	2	14247.5	15237	28131	26886	19555	18911	32408.5	33858.5	23415.5	23868	0.199072	0.70099	20811.3	26492.3	Up	0.348206	0.747784	Isopentenyl-diphosphate Delta-isomerase 1 OS=Mus musculus GN=Idi1 PE=2 SV=1
Mbp	P04370-2	11	11	894576.2	1835074	2610688	2490746	1560653	510962.5	1550222	1785705	974156	1813162	0.211242	0.67522	1878387	1326842	Down	-0.5015	0.747784	Isoform 2 of Myelin basic protein OS=Mus musculus GN=Mbp
Clasp1	Q80TV8	1	1	NA	132045	141907	91079	128544	122741	164965	125935	145433	158806	0.199607	0.699827	123393.8	143576	Up	0.218545	0.747784	CLIP-associating protein 1 OS=Mus musculus GN=Clasp1 PE=1 SV=2
Rps5	P97461	2	2	37944.5	109777	112926	69512	100252.5	72089.5	139407.5	161780.5	91819	115637	0.199757	0.699498	86082.4	116146.7	Up	0.432158	0.747784	40S ribosomal protein S5 OS=Mus musculus GN=Rps5 PE=1 SV=3
Srcin1	Q9QWI6-3	33	33	237543.5	172985	179149.3	151559.2	205092.4	199028.9	159082.9	176972.3	151877	132652.1	0.212632	0.672371	189265.9	163922.6	Down	-0.2074	0.747784	Isoform 3 of SRC kinase signaling inhibitor 1 OS=Mus musculus GN=Srcin1
Sars	P26638	3	3	NA	27330.5	45466.5	23233	19269	61413	30314.5	30281	58177	29726	0.20067	0.697518	28824.75	41982.3	Up	0.542473	0.747784	Serine--tRNA ligase, cytoplasmic OS=Mus musculus GN=Sars PE=2 SV=3
Uba1	Q02053	19	19	118014.9	250101.7	247574.8	253731.4	216888.5	258880.2	260207.6	260565.9	253197.4	249575	0.201532	0.695656	217262.3	256485.2	Up	0.239438	0.747784	Ubiquitin-like modifier-activating enzyme 1 OS=Mus musculus GN=Uba1 PE=1 SV=1
Emc10	Q3TAS6	1	1	NA	11182	7501	4479	6734	NA	7681	6724	19063	21883	0.201873	0.694922	7474	13837.75	Up	0.888657	0.747784	ER membrane protein complex subunit 10 OS=Mus musculus GN=Emc10 PE=2 SV=2
Tpd52l2	Q9CYZ2	1	1	1209456	568169	723387	654888	812031	857090	642230	650089	460726	433992	0.213821	0.66995	793586.2	609025.4	Down	-0.38188	0.747784	Tumor protein D54 OS=Mus musculus GN=Tpd52l2 PE=1 SV=1
Sfxn3	Q91V61-2	2	2	90878	111885.5	145304.5	156718.5	125144.5	165501	148695.5	118667.5	137604.5	160683.5	0.202194	0.694232	125986.2	146230.4	Up	0.214978	0.747784	Isoform 2 of Sideroflexin-3 OS=Mus musculus GN=Sfxn3
Sfxn1	Q99JR1	1	1	181756	223771	243999	280445	250289	248729	259090	237335	275209	295838	0.203142	0.6922	236052	263240.2	Up	0.157275	0.747784	Sideroflexin-1 OS=Mus musculus GN=Sfxn1 PE=1 SV=3
Rps9	Q6ZWN5	2	2	88963	143169	151562.5	155883	221167.5	216770	159355	154235.5	208073	195951.5	0.203416	0.691615	152149	186877	Up	0.296604	0.747784	40S ribosomal protein S9 OS=Mus musculus GN=Rps9 PE=1 SV=3
Rasgrp2	Q9QUG9	1	1	147900	281399	251382	257970	253472	262680	332303	257734	225917	330889	0.20513	0.687971	238424.6	281904.6	Up	0.241674	0.747784	RAS guanyl-releasing protein 2 OS=Mus musculus GN=Rasgrp2 PE=1 SV=1
Tuba3a	P05214	69	69	1405044	1734355	1497692	1580033	1108629	1358185	1617116	1560296	1868519	1957340	0.205354	0.687497	1465150	1672291	Up	0.190777	0.747784	Tubulin alpha-3 chain OS=Mus musculus GN=Tuba3a PE=1 SV=1
Hexa	P29416	2	2	50226.5	70630.5	97663	49171.5	45166	93698	71758	95868	74508.5	60244	0.205647	0.686878	62571.5	79215.3	Up	0.340273	0.747784	Beta-hexosaminidase subunit alpha OS=Mus musculus GN=Hexa PE=2 SV=2
Spock1	Q62288	2	2	56486.5	106087	89887	89532	96665.5	82698	109322.5	109515	106451.5	96874	0.205792	0.686572	88131.6	101372.2	Up	0.201931	0.747784	Testican-1 OS=Mus musculus GN=Spock1 PE=2 SV=2
Upf1	Q9EPU0	3	3	69477.33	54754	70826.33	61545	94937	161165.7	95851.67	70811.33	77935.67	75465.67	0.208362	0.681181	70307.93	96246	Up	0.453039	0.747784	Regulator of nonsense transcripts 1 OS=Mus musculus GN=Upf1 PE=1 SV=2
Abat	P61922	8	8	244335.5	215059.4	221362.3	233560.4	170769	203009.3	181996.8	203564.8	201424.4	200907.3	0.216541	0.66446	217017.3	198180.5	Down	-0.131	0.747784	4-aminobutyrate aminotransferase, mitochondrial OS=Mus musculus GN=Abat PE=1 SV=1
Prkcd	P28867	2	2	173653	81032	80647	70788	97303.5	70838	97833.5	71242.5	62691	60397.5	0.216937	0.663666	100684.7	72600.5	Down	-0.47179	0.747784	Protein kinase C delta type OS=Mus musculus GN=Prkcd PE=1 SV=3
Habp4	Q9JKS5	4	4	207685	120481.8	131647.3	125010.8	183022	143478.5	135867.3	150904	111399.5	77014.25	0.217016	0.663508	153569.4	123732.7	Down	-0.31166	0.747784	Intracellular hyaluronan-binding protein 4 OS=Mus musculus GN=Habp4 PE=1 SV=2
Rap1b	Q99JI6	2	2	806487.5	602729	625524.5	453087.5	402264	554571	389696	465656	457475.5	487513.5	0.2173	0.66294	578018.5	470982.4	Down	-0.29544	0.747784	Ras-related protein Rap-1b OS=Mus musculus GN=Rap1b PE=2 SV=2
Snrnp70	Q62376	3	3	306863	390794.3	478681.3	335846.7	509525	415474.7	468706	498473.3	435798	510424.3	0.208907	0.680047	404342.1	465775.3	Up	0.204058	0.747784	U1 small nuclear ribonucleoprotein 70 kDa OS=Mus musculus GN=Snrnp70 PE=1 SV=2
Sfxn3	Q91V61	5	5	36351.2	50713.6	64554.8	70967.2	55869.6	75152.4	65900.4	53785.2	61891.4	70359.6	0.209095	0.679656	55691.28	65417.8	Up	0.232232	0.747784	Sideroflexin-3 OS=Mus musculus GN=Sfxn3 PE=1 SV=1
Naa35	Q6PHQ8	1	1	207988	339540	314992	249179	254292	433694	365407	352604	312945	207490	0.209583	0.678644	273198.2	334428	Up	0.291748	0.747784	N-alpha-acetyltransferase 35, NatC auxiliary subunit OS=Mus musculus GN=Naa35 PE=1 SV=1
Eci1	P42125	3	3	212437	342204	294933	327660.7	306058	266361.7	277442	255358.3	240114.7	276011.7	0.217616	0.662309	296658.5	263057.7	Down	-0.17342	0.747784	Enoyl-CoA delta isomerase 1, mitochondrial OS=Mus musculus GN=Eci1 PE=1 SV=2
Psmc5	P62196	1	1	NA	32226	61073	64585	49690	31409	89913	59905	76319	101451	0.209846	0.678099	51893.5	71799.4	Up	0.468418	0.747784	26S protease regulatory subunit 8 OS=Mus musculus GN=Psmc5 PE=1 SV=1
Hnrnpu	Q8VEK3	8	8	212054.1	101756.4	160838.3	121400.6	272534.1	99030	150143.4	158783.3	135335.4	88788.57	0.217943	0.661657	173716.7	126416.1	Down	-0.45856	0.747784	Heterogeneous nuclear ribonucleoprotein U OS=Mus musculus GN=Hnrnpu PE=1 SV=1
Cc2d1a	Q8K1A6	2	2	733433	422498	576287	418786	791294	601998	551343	451560	420554	271441	0.218571	0.660407	588459.6	459379.2	Down	-0.35726	0.747784	Coiled-coil and C2 domain-containing protein 1A OS=Mus musculus GN=Cc2d1a PE=1 SV=2
Sh3glb2	Q8R3V5	11	11	123480.8	103411.8	103535.4	103109.1	113085.9	84425	105670.8	117931.5	97299.75	94830.5	0.218619	0.660312	109324.6	100031.5	Down	-0.12816	0.747784	Endophilin-B2 OS=Mus musculus GN=Sh3glb2 PE=2 SV=2
Sh3glb2	Q8R3V5-3	10	10	123480.8	103411.8	103535.4	103109.1	113085.9	84425	105670.8	117931.5	97299.75	94830.5	0.218619	0.660312	109324.6	100031.5	Down	-0.12816	0.747784	Isoform 3 of Endophilin-B2 OS=Mus musculus GN=Sh3glb2
Ei24	Q61070	1	1	176739	171857	183619	208312	293406	88047	199489	218572	163998	151065	0.218809	0.659935	206786.6	164234.2	Down	-0.33239	0.747784	Etoposide-induced protein 2.4 OS=Mus musculus GN=Ei24 PE=1 SV=3
Erlin2	Q8BFZ9	2	2	70953.5	155127	115457.5	117345	145088.5	104685	174655.5	170307	147853.5	138239	0.210602	0.676538	120794.3	147148	Up	0.284716	0.747784	Erlin-2 OS=Mus musculus GN=Erlin2 PE=1 SV=1
Hsp90b1	P08113	15	15	278925.9	154887.3	198009.8	136340.5	180159.6	222173.7	213897.2	251054.9	232141.1	215046.9	0.210862	0.676002	189664.6	226862.8	Up	0.258369	0.747784	Endoplasmin OS=Mus musculus GN=Hsp90b1 PE=1 SV=2
Mapk3	Q63844	1	1	303587	553948	424408	410565	446670	381757	302849	444158	340316	354492	0.219485	0.658595	427835.6	364714.4	Down	-0.23029	0.747784	Mitogen-activated protein kinase 3 OS=Mus musculus GN=Mapk3 PE=1 SV=5
Crip2	Q9DCT8	4	4	591275	182592	204062	208127	386496	269548	222338	199225	174025	129570	0.220024	0.65753	314510.4	198941.2	Down	-0.66077	0.747784	Cysteine-rich protein 2 OS=Mus musculus GN=Crip2 PE=1 SV=1
Auh	Q9JLZ3-3	1	1	2423391	492352	1166265	779150	949457	942825	777516	735376	517683	376044	0.220515	0.656562	1162123	669888.8	Down	-0.79477	0.747784	Isoform 3 of Methylglutaconyl-CoA hydratase, mitochondrial OS=Mus musculus GN=Auh
Arpc1a	Q9R0Q6	2	2	267934.5	193409.5	187426	205227.5	237290	174241	188794.5	209299.5	226796	166539.5	0.220617	0.656361	218257.5	193134.1	Down	-0.17643	0.747784	Actin-related protein 2/3 complex subunit 1A OS=Mus musculus GN=Arpc1a PE=1 SV=1
Ankrd49	Q8VE42	1	1	NA	116763	75322	98587	32590	114992	99442	94172	109383	131144	0.211408	0.674879	80815.5	109826.6	Up	0.442524	0.747784	Ankyrin repeat domain-containing protein 49 OS=Mus musculus GN=Ankrd49 PE=2 SV=1
Anks1b	Q8BIZ1-4	3	3	216615	234444.7	236031	226299	224037.3	238561.7	249890.3	222086.7	225123.3	248468	0.212128	0.673402	227485.4	236826	Up	0.058054	0.747784	Isoform 4 of Ankyrin repeat and sterile alpha motif domain-containing protein 1B OS=Mus musculus GN=Anks1b
Tceal3	Q8R0A5	3	3	189046.7	162142.7	146273	137642.3	169684.7	179529	155107	196874	170943.7	179232	0.212725	0.672181	160957.9	176337.1	Up	0.131653	0.747784	Transcription elongation factor A protein-like 3 OS=Mus musculus GN=Tceal3 PE=2 SV=2
Magi2	Q9WVQ1-4	1	1	632608	589028	442935	577236	488279	681927	501054	561024	624162	718681	0.213009	0.671602	546017.2	617369.6	Up	0.177188	0.747784	Isoform 4 of Membrane-associated guanylate kinase, WW and PDZ domain-containing protein 2 OS=Mus musculus GN=Magi2
Csnk2b	P67871	4	4	229880	192216.3	208632.3	212153.8	214239.5	290019.3	212554.8	232759.5	220738.8	212410.3	0.213218	0.671176	211424.4	233696.5	Up	0.144495	0.747784	Casein kinase II subunit beta OS=Mus musculus GN=Csnk2b PE=1 SV=1
Ak4	Q9WUR9	5	5	68453.4	132174.4	117193.4	127811.4	89297.2	156135.6	126046.8	116697.6	130078	106626.2	0.21419	0.669201	106986	127116.8	Up	0.248734	0.747784	Adenylate kinase 4, mitochondrial OS=Mus musculus GN=Ak4 PE=1 SV=1
Nav3	Q80TN7	1	1	64982	50482	63760	48524	97282	37560	40793	75170	57442	39016	0.223662	0.650408	65006	49996.2	Down	-0.37875	0.747784	Neuron navigator 3 OS=Mus musculus GN=Nav3 PE=1 SV=2
2610301B	Q3UJP5	1	1	NA	NA	31622	24792	NA	78565	43533	NA	NA	34232	0.214273	0.669033	28207	52110	Up	0.885507	0.747784	Protein C8orf37 homolog OS=Mus musculus PE=1 SV=1
Camk2b	P28652	30	30	398641.5	1148711	1119304	1062969	866614.5	1071361	1099149	1166159	1077917	1212009	0.214861	0.667842	919248	1125319	Up	0.291808	0.747784	Calcium/calmodulin-dependent protein kinase type II subunit beta OS=Mus musculus GN=Camk2b PE=1 SV=2
Snx12	O70493	6	6	1190251	1509124	1078399	1086192	1248576	1378347	1290108	1405791	1272928	1353811	0.215087	0.667386	1222508	1340197	Up	0.132601	0.747784	Sorting nexin-12 OS=Mus musculus GN=Snx12 PE=1 SV=1
Kif5a	P33175	3	3	135969	436502.5	188127.5	331616.5	248807.5	312376.5	333269.5	244292	419042.5	499251.5	0.215219	0.667119	268204.6	361646.4	Up	0.431246	0.747784	Kinesin heavy chain isoform 5A OS=Mus musculus GN=Kif5a PE=1 SV=3
Nars	Q8BP47	1	1	NA	33062	25016	42500	NA	NA	36043	49300	37804	47067	0.216055	0.665436	33526	42553.5	Up	0.343997	0.747784	Asparagine--tRNA ligase, cytoplasmic OS=Mus musculus GN=Nars PE=1 SV=2
Hibch	Q8QZS1	3	3	64673.33	135898.7	130431.7	136824.7	115458.3	145143.7	138167.7	130737.7	135682.7	133464	0.216068	0.66541	116657.3	136639.1	Up	0.228094	0.747784	3-hydroxyisobutyryl-CoA hydrolase, mitochondrial OS=Mus musculus GN=Hibch PE=1 SV=1
Psmc4	P54775	4	4	87441.5	83989	125693.5	85688.5	125212.5	104540	123222.5	139842.5	109111	110646.5	0.216322	0.664899	101605	117472.5	Up	0.209352	0.747784	26S protease regulatory subunit 6B OS=Mus musculus GN=Psmc4 PE=1 SV=2
Prrc2b	Q7TPM1	1	1	NA	NA	5896	6821	NA	13599	9709	7291	5649	NA	0.217314	0.662912	6358.5	9062	Up	0.511143	0.747784	Protein PRRC2B OS=Mus musculus GN=Prrc2b PE=1 SV=1
Mtcl1	O3UHU5-3	4	4	90976.75	161993.8	175332.8	159695.8	123014.8	146665.3	186612.5	170957.8	180132	147258.3	0.21753	0.662481	142202.8	166325.2	Up	0.226057	0.747784	Isoform 3 of Protein SOGA2 OS=Mus musculus GN=Soga2
Dip2a	Q8BWT5	1	1	NA	207808	262831	194335	265776	232256	246780	200912	155625	148004	0.226004	0.645884	232687.5	196715.4	Down	-0.24228	0.747784	Disco-interacting protein 2 homolog A OS=Mus musculus GN=Dip2a PE=2 SV=3
Ubap2l	Q80X50-3	5	5	112932.3	137211	101345	85811	124893.7	154217.7	118263.3	120462	106205.7	150203.3	0.217538	0.662465	112438.6	129870.4	Up	0.207935	0.747784	Isoform 3 of Ubiquitin-associated protein 2-like OS=Mus musculus GN=Ubap2l
Pitpnm1	O35954	4	4	51045.67	255363.7	164281.3	187351	115143.7	170585	201758.3	203053	225665.3	226915	0.217913	0.661717	154637.1	205595.3	Up	0.410921	0.747784	Membrane-associated phosphatidylinositol transfer protein 1 OS=Mus musculus GN=Pitpnm1 PE=1 SV=1
Cdk18	Q04899	1	1	228917	324683	301329	362169	471867	404496	345842	525343	384349	369355	0.218935	0.659685	337793	405877	Up	0.264903	0.747784	Cyclin-dependent kinase 18 OS=Mus musculus GN=Cdk18 PE=1 SV=1
Dnm1	P39053-3	41	41	424158.6	317111.9	378260.2	338769.2	350892.6	328310.1	370991.2	306949.6	349481.4	305880	0.227139	0.643708	361838.5	332322.4	Down	-0.12276	0.747784	Isoform 3 of Dynamin-1 OS=Mus musculus GN=Dnm1
Rab3gap1	Q80UJ7	1	1	103317	421714	334008	375713	410216	551626	320948	402094	403308	436538	0.219176	0.659207	328993.6	422902.8	Up	0.362267	0.747784	Rab3 GTPase-activating protein catalytic subunit OS=Mus musculus GN=Rab3gap1 PE=2 SV=4
Bag6	Q9Z1R2	4	4	214536.3	99684	152004	138998	181200.7	155127.7	121157.3	127138.3	125606.7	115751.3	0.227212	0.643569	157284.6	128956.3	Down	-0.2865	0.747784	Large proline-rich protein BAG6 OS=Mus musculus GN=Bag6 PE=1 SV=1
Ndufa11	Q9D8B4	2	2	748125	374679.5	364583	494041.5	377256	424849.5	325860	400200	369170	309926	0.227426	0.64316	471737	366001.1	Down	-0.36613	0.747784	NADH dehydrogenase [ubiquinone] 1 alpha subcomplex subunit 11 OS=Mus musculus GN=Ndufa11 PE=2 SV=2
Etf1	Q8BWY3	1	1	NA	32520	68990	55491	38468	NA	86962	75490	68668	37897	0.220618	0.656359	48867.25	67254.25	Up	0.460758	0.747784	Eukaryotic peptide chain release factor subunit 1 OS=Mus musculus GN=Etf1 PE=1 SV=4
Dctn1	O08788	10	10	190082.3	199917	224651	231571.8	224992.8	250381.1	237722.9	221562.6	209389.1	223703.3	0.220691	0.656215	214243	228551.8	Up	0.093273	0.747784	Dynactin subunit 1 OS=Mus musculus GN=Dctn1 PE=1 SV=3
Dst	Q91ZU6-5	2	2	9865	38594.5	36663	50939.5	54196	46793	44118.5	59220	41496	57965	0.220999	0.65561	38051.6	49918.5	Up	0.391617	0.747784	Isoform 5 of Dystonin OS=Mus musculus GN=Dst
Sirt5	Q8K2C6	3	3	705380	388825.3	419053.3	388367	526352	500398	430588	377019.3	359208.3	297827	0.228841	0.640466	485595.5	393008.1	Down	-0.3052	0.747784	NAD-dependent protein deacylase sirtuin-5, mitochondrial OS=Mus musculus GN=Sirt5 PE=1 SV=1
2310035C	Q148V7	2	2	104415.5	168462	194460.5	141366	180685.5	157067.5	200269	188551	201390.5	166500	0.221131	0.65535	157877.9	182755.6	Up	0.211106	0.747784	LisH domain and HEAT repeat-containing protein KIAA1468 OS=Mus musculus GN=Kiaa1468 PE=1 SV=1
Bag5	Q8CI32	1	1	NA	85308	97554	79716	63498	63285	118232	108483	101481	94543	0.223083	0.651534	81519	97204.8	Up	0.253891	0.747784	BAG family molecular chaperone regulator 5 OS=Mus musculus GN=Bag5 PE=1 SV=1
Grin1	P35438	3	3	149799.7	145356	175988	181449.7	261203.7	239850.7	206823.3	246775.3	183352.7	199359.3	0.223571	0.650585	182759.4	215232.3	Up	0.235949	0.747784	Glutamate receptor ionotropic, NMDA 1 OS=Mus musculus GN=Grin1 PE=1 SV=1
Mia2	Q8R311	3	3	20550	39563.5	94166	69365	80144	90590	61404	86102	80496.5	84830	0.223815	0.650111	60757.7	80684.5	Up	0.409224	0.747784	cTAGE family member 5 OS=Mus musculus GN=Ctage5 PE=1 SV=1
Dmxl2	Q8BPN8-3	1	1	19697	90666	82076	108699	77705	76548	120661	89038	117583	91323	0.224112	0.649535	75768.6	99030.6	Up	0.386274	0.747784	Isoform 3 of DmX-like protein 2 OS=Mus musculus GN=Dmxl2
Dclk2	Q6PGN3-3	3	3	233247.7	144445.7	127383.3	147225.7	161422.7	156489.7	132944.3	137953.7	127163.3	126523	0.229794	0.638661	162745	136214.8	Down	-0.25673	0.747784	Isoform 3 of Serine/threonine-protein kinase DCLK2 OS=Mus musculus GN=Dclk2
Rtca	Q9D7H3	1	1	NA	NA	24655	15114	14258	22059	21235	27815	35745	15811	0.224137	0.649486	18009	24533	Up	0.446006	0.747784	RNA 3'-terminal phosphate cyclase OS=Mus musculus GN=RtcA PE=2 SV=2
Apba1	B2RUJ5	3	3	217243	236191	194291	224184	218160	199032	253874	256825	218606	251691	0.224964	0.647887	218013.8	236005.6	Up	0.114402	0.747784	Amyloid beta A4 precursor protein-binding family A member 1 OS=Mus musculus GN=Apba1 PE=1 SV=2
Atic	Q9CWJ9	5	5	145864.8	134352.6	145130.6	117217	123150.4	128800.8	130033.6	127483.6	123848.4	111525.6	0.23108	0.636238	133143.1	124338.4	Down	-0.09871	0.747784	Bifunctional purine biosynthesis protein PURH OS=Mus musculus GN=Atic PE=1 SV=2
Apba1	B2RUJ5-2	1	1	217243	236191	194291	224184	218160	199032	253874	256825	218606	251691	0.224964	0.647887	218013.8	236005.6	Up	0.114402	0.747784	Isoform 2 of Amyloid beta A4 precursor protein-binding family A member 1 OS=Mus musculus GN=Apba1
Arhgef10l	A2AWP8-3	1	1	33088	39918	54156	35866	46633	76658	53604	35086	42806	55456	0.22507	0.647682	41932.2	52722	Up	0.330347	0.747784	Isoform 3 of Rho guanine nucleotide exchange factor 10-like protein OS=Mus musculus GN=Arhgef10l
Slc6a11	P31650	2	2	NA	154512	237847	210055.5	202996.5	197245	217138	306040.5	253894.5	207743	0.225082	0.647659	201352.8	236412.2	Up	0.231579	0.747784	Sodium- and chloride-dependent GABA transporter 3 OS=Mus musculus GN=Slc6a11 PE=1 SV=2
Ddx42	Q810A7	1	1	87951	86311	98725	67646	106365	148920	81667	102458	101184	99758	0.225119	0.647588	89399.6	106797.4	Up	0.256536	0.747784	ATP-dependent RNA helicase DDX42 OS=Mus musculus GN=Ddx42 PE=1 SV=3
Ndufa7	Q9Z1P6	3	3	162954.3	63670.33	88785.33	110118.3	127497.7	118209.7	82934	98094.67	63806	57620.67	0.232303	0.633945	110605.2	84133	Down	-0.39468	0.747784	NADH dehydrogenase [ubiquinone] 1 alpha subcomplex subunit 7 OS=Mus musculus GN=Ndufa7 PE=1 SV=3
Asphd1	Q2TA57	1	1	NA	92530	95575	87013	100237	51225	91286	92322	92200	85131	0.232542	0.633499	93838.75	82432.8	Down	-0.18697	0.747784	Aspartate beta-hydroxylase domain-containing protein 1 OS=Mus musculus GN=Asphd1 PE=2 SV=2
Pspc1	Q8R326	3	3	84702.67	48491.33	66608.33	55683.67	132264.3	47589.67	69643	74090	51492	33667.67	0.23259	0.633409	77550.07	55296.47	Down	-0.48794	0.747784	Paraspeckle component 1 OS=Mus musculus GN=Pspc1 PE=1 SV=1
Sptan1	P16546-2	140	140	575212.6	560105.1	562255.8	639174	631716	604470	572757.5	553398.1	558438.9	547396.7	0.232851	0.632922	593692.7	567292.2	Down	-0.06562	0.747784	Isoform 2 of Spectrin alpha chain, non-erythrocytic 1 OS=Mus musculus GN=Sptan1
Srcin1	Q9QWI6-2	33	33	237543.5	170797.1	179108	149563.4	207090.7	197541.5	159831.9	179453.7	151578.5	132685.5	0.233544	0.631631	188820.5	164218.2	Down	-0.2014	0.747784	Isoform 2 of SRC kinase signaling inhibitor 1 OS=Mus musculus GN=Srcin1

Arrb1	Q8BWG8	2	2	43304.5	82198.5	69194.5	64481.5	86580	58120.5	91991.5	80716.5	95359.5	85840.5	0.225328	0.647185	69151.8	82405.7	Up	0.252977	0.747784	Beta-arrestin-1 OS=Mus musculus GN=Arrb1 PE=1 SV=1
Clybl	Q8R4N0	2	2	237414	414676.5	301045	469136.5	266537.5	402150	378645	352835.5	432445	451262.5	0.22633	0.645258	337761.9	403467.6	Up	0.256446	0.747784	Citrate lyase subunit beta-like protein, mitochondrial OS=Mus musculus GN=Clybl PE=1 SV=2
Zc3h15	Q3TIV5-3	1	1	2771489	350744	1275818	433630	1881527	946648	828675	867363	555886	289794	0.234588	0.629694	1342642	697673.2	Down	-0.94445	0.747784	Isoform 3 of Zinc finger CCCH domain-containing protein 15 OS=Mus musculus GN=Zc3h15
Nfs1	Q9Z1J3	3	3	261466.3	250462	273966.7	295526.7	238046	266041	284276	291581	273517	277699.3	0.226409	0.645106	263893.5	278622.9	Up	0.078358	0.747784	Cysteine desulfurase, mitochondrial OS=Mus musculus GN=Nfs1 PE=2 SV=3
Sacs	Q9JLC8-3	1	1	51407	127662	172021	169377	146390	124564	190102	165956	159959	196578	0.226533	0.644869	133371.4	167431.8	Up	0.328124	0.747784	Isoform 3 of Sacsin OS=Mus musculus GN=Sacs
Capza2	P47754	3	3	577065.7	253813.7	344849.7	250646.7	412232.3	371940.7	308182.3	281765.7	234569.3	194577.7	0.234798	0.629306	367721.6	278207.1	Down	-0.40245	0.747784	F-actin-capping protein subunit alpha-2 OS=Mus musculus GN=Capza2 PE=1 SV=3
Camk4	P08414	2	2	453313.5	570768.5	453129.5	434499	576798.5	441100	437835	496704	459043	428568	0.234841	0.629226	497701.8	452650	Down	-0.13689	0.747784	Calcium/calmodulin-dependent protein kinase type IV OS=Mus musculus GN=Camk4 PE=1 SV=2
Acads	Q07417	1	1	153558	154047	179004	165186	155380	180488	150287	151538	208978	232712	0.227157	0.643674	161435	184800.6	Up	0.195016	0.747784	Short-chain specific acyl-CoA dehydrogenase, mitochondrial OS=Mus musculus GN=Acads PE=1 SV=2
Map1s	Q8C052	1	1	175494	132510	149926	121480	125272	142623	130572	133768	106128	113988	0.235748	0.627552	140936.4	125415.8	Down	-0.16833	0.747784	Microtubule-associated protein 1S OS=Mus musculus GN=Map1s PE=1 SV=2
Tubb5	P99024	98	98	907294.9	1554084	1553729	1468050	1245876	1378830	1510688	1469771	1640523	1636017	0.228057	0.641957	1345807	1527166	Up	0.182385	0.747784	Tubulin beta-5 chain OS=Mus musculus GN=Tubb5 PE=1 SV=1
Srcin1	Q9QWI6	34	34	229352.3	169551.1	177183.3	148169.7	203487.8	193928.5	158497.2	178050.4	151123.8	132210.3	0.236085	0.626932	185548.8	162762	Down	-0.18903	0.747784	SRC kinase signaling inhibitor 1 OS=Mus musculus GN=Srcin1 PE=1 SV=2
Fhl1	P97447	1	1	NA	59457	41401	75239	113823	67308	122414	92081	108011	95604	0.228123	0.641831	72480	97083.6	Up	0.421645	0.747784	Four and a half LIM domains protein 1 OS=Mus musculus GN=Fhl1 PE=1 SV=3
Vps50	Q8CI71	1	1	23368	41280	35086	46197	48304	58030	41795	43648	35639	55429	0.228299	0.641496	38847	46908.2	Up	0.272037	0.747784	Coiled-coil domain-containing protein 132 OS=Mus musculus GN=Ccdc132 PE=2 SV=2
Cog4	Q8R1U1	1	1	NA	25397	10388	18861	11575	12109	9916	6801	18915	7504	0.236482	0.626202	16555.25	11049	Down	-0.58337	0.747784	Conserved oligomeric Golgi complex subunit 4 OS=Mus musculus GN=Cog4 PE=2 SV=1
Tubb3	Q9ERD7	62	62	707040	1667952	1667983	1567554	1346711	1489556	1590166	1603909	1752178	1833590	0.228907	0.640341	1391448	1653880	Up	0.249268	0.747784	Tubulin beta-3 chain OS=Mus musculus GN=Tubb3 PE=1 SV=1
Ppid	Q9CR16	5	5	325467.8	179106	168857.6	192482.2	211628.2	229135.4	158828.2	188913.4	154432.8	130991.6	0.236925	0.625389	215508.4	172460.3	Down	-0.32148	0.747784	Peptidyl-prolyl cis-trans isomerase D OS=Mus musculus GN=Ppid PE=1 SV=3
Rac3	P60764	2	2	32073	47662.5	86816.5	61422	57507.5	45921	75896	79532	78966	77826.5	0.229274	0.639645	57096.3	71628.3	Up	0.327132	0.747784	Ras-related C3 botulinum toxin substrate 3 OS=Mus musculus GN=Rac3 PE=1 SV=1
Pitpnc1	Q8K4R4-3	1	1	256866	360414	265191	298427	323605	256256	254495	277441	325326	232808	0.237469	0.624393	300900.6	269265.2	Down	-0.16026	0.747784	Isoform 3 of Cytoplasmic phosphatidylinositol transfer protein 1 OS=Mus musculus GN=Pitpnc1
Endog	O08600	3	3	116239	85942.33	88089.33	84166.67	88029	90411.33	88581.67	96208	60168	68533.67	0.238107	0.623228	92493.27	80780.53	Down	-0.19534	0.747784	Endonuclease G, mitochondrial OS=Mus musculus GN=Endog PE=2 SV=1
Ephx2	P34914	1	1	41834	83137	71935	82479	76532	83377	83786	84802	86248	72721	0.229427	0.639355	71183.4	82186.8	Up	0.207366	0.747784	Bifunctional epoxide hydrolase 2 OS=Mus musculus GN=Ephx2 PE=1 SV=2
Ahsa1	Q8BK64	1	1	747395	342155	446938	356709	411541	434770	293883	407661	356437	280969	0.238647	0.622244	460947.6	354744	Down	-0.37782	0.747784	Activator of 90 kDa heat shock protein ATPase homolog 1 OS=Mus musculus GN=Ahsa1 PE=2 SV=2
Pdcd10	Q8VE70	2	2	281813.5	166634.5	182849	220790.5	206278.5	98840	248503.5	65579	224645	158382	0.238735	0.622084	211673.2	159189.9	Down	-0.41109	0.747784	Programmed cell death protein 10 OS=Mus musculus GN=Pdcd10 PE=1 SV=1
Bdh1	Q80XN0	5	5	89860	43989	124685.8	132995.5	197234.5	74177.5	69016.5	91422.5	80228	97475.5	0.238757	0.622044	117753	82464	Down	-0.51393	0.747784	D-beta-hydroxybutyrate dehydrogenase, mitochondrial OS=Mus musculus GN=Bdh1 PE=1 SV=2
Phyhipl	Q8BGT8	2	2	96372	70808	81396.5	78773.5	99352.5	85248.5	81694.5	84595	69702	55103.5	0.239143	0.621342	85340.5	75268.7	Down	-0.18118	0.747784	Phytanoyl-CoA hydroxylase-interacting protein-like OS=Mus musculus GN=Phyhipl PE=2 SV=1
Zc3h15	Q3TIV5	2	2	1421105	199091.5	666287	249143.5	963658	500823	461902	455763.5	312389	167804	0.239286	0.621083	699857	379736.3	Down	-0.88206	0.747784	Zinc finger CCCH domain-containing protein 15 OS=Mus musculus GN=Zc3h15 PE=1 SV=2
Gm2a	Q60648	2	2	630825	233030	281296	243549	312809	227151	299018	241632	203300	215325	0.239834	0.620089	340301.8	237285.2	Down	-0.52019	0.747784	Ganglioside GM2 activator OS=Mus musculus GN=Gm2a PE=1 SV=2
Tubb4b	P68372	99	99	906590.5	1563018	1566171	1463589	1244002	1370844	1526334	1488153	1639894	1633639	0.229507	0.639204	1348674	1531773	Up	0.183661	0.747784	Tubulin beta-4B chain OS=Mus musculus GN=Tubb4b PE=1 SV=1
Dab2ip	Q3UHC7-3	3	3	146266.5	151406	122625	132960	130504	183576	157490	153004.5	122198.5	142954	0.229753	0.638739	136752.3	151844.6	Up	0.151031	0.747784	Isoform 3 of Disabled homolog 2-interacting protein OS=Mus musculus GN=Dab2ip
Ctbp1	O88712-3	3	3	342402	196197	264324.3	251587	260552	287207	216863.3	219140.7	210616	201879.7	0.240346	0.619163	263012.5	227141.3	Down	-0.21154	0.747784	Isoform 3 of C-terminal-binding protein 1 OS=Mus musculus GN=Ctbp1
Add1	Q9QYC0	12	12	63408.22	55985.67	58539.22	54285	73589.56	62566.44	58728.78	53204.22	47892.56	56141.44	0.240502	0.618881	61161.53	55706.69	Down	-0.13477	0.747784	Alpha-adducin OS=Mus musculus GN=Add1 PE=1 SV=2
Arpc5	Q9CPW4	2	2	119363	122254	98798.5	99015	132739	26609	100749.5	116925	79267.5	124171.5	0.240508	0.61887	114433.9	89544.5	Down	-0.35384	0.747784	Actin-related protein 2/3 complex subunit 5 OS=Mus musculus GN=Arpc5 PE=2 SV=3
Dgkb	Q6NS52	7	7	54582.57	73982.43	87078	72031.43	95564.29	119939.3	71231.71	108099	88507.29	73080.43	0.230879	0.636616	76647.74	92171.54	Up	0.266078	0.747784	Diacylglycerol kinase beta OS=Mus musculus GN=Dgkb PE=2 SV=2
Arpc3	Q9JM76	1	1	107967	188437	241374	266472	113633	162894	222471	293669	217360	283882	0.231066	0.636264	183576.6	236055.2	Up	0.362742	0.747784	Actin-related protein 2/3 complex subunit 3 OS=Mus musculus GN=Arpc3 PE=1 SV=3
Cltb	Q6IRU5-2	4	4	1265007	672610	658501.8	640473.3	632751.3	645090.3	654676.8	644951	623091.5	435711.8	0.240844	0.618264	773868.7	600704.3	Down	-0.36543	0.747784	Isoform 2 of Clathrin light chain B OS=Mus musculus GN=Cltb
Wdr1	O88342	4	4	1127697	570622.3	626270.8	591557.8	669362.3	638642.5	608266.5	570564.8	535618.5	510411.3	0.240862	0.618232	717102	572700.7	Down	-0.3244	0.747784	WD repeat-containing protein 1 OS=Mus musculus GN=Wdr1 PE=1 SV=3
Tuba4a	P68368	82	82	1292476	1461806	1303017	1353011	965117.3	1192269	1379222	1335533	1587251	1641743	0.231193	0.636025	1275085	1427204	Up	0.162597	0.747784	Tubulin alpha-4A chain OS=Mus musculus GN=Tuba4a PE=1 SV=1
Aup1	P70295	1	1	NA	729079	496594	576328	701782	816009	650656	798335	658144	651255	0.231465	0.635515	625945.8	714879.8	Up	0.191663	0.747784	Ancient ubiquitous protein 1 OS=Mus musculus GN=Aup1 PE=2 SV=1
Rps23	P62267	9	9	750306.7	683078.5	662082.3	676233.3	634319.3	882507.5	693955.2	708429.5	652326.2	763981.8	0.231875	0.634746	681204	740240	Up	0.119906	0.747784	40S ribosomal protein S23 OS=Mus musculus GN=Rps23 PE=1 SV=3
Ptbp2	Q91Z31	1	1	38619	54955	111486	86798	104985	67032	86662	117465	129527	114048	0.231898	0.634703	79368.6	102946.8	Up	0.375259	0.747784	Polypyrimidine tract-binding protein 2 OS=Mus musculus GN=Ptbp2 PE=1 SV=1
Gja1	P23242	1	1	115994	148510	195367	128926	161934	235597	152838	190617	122680	206549	0.233576	0.631572	150146.2	181656.2	Up	0.274843	0.747784	Gap junction alpha-1 protein OS=Mus musculus GN=Gja1 PE=1 SV=2
Cltb	Q6IRU5	8	8	843770.3	450139.2	439556.2	428079.5	422653	430897	437434.2	433883.2	416236.8	291498	0.2425	0.615288	516839.6	401989.8	Down	-0.36256	0.749236	Clathrin light chain B OS=Mus musculus GN=Cltb PE=1 SV=1
Gclm	O09172	1	1	NA	4795	15313	10405	5196	9304	13338	13869	12967	13801	0.233792	0.63117	8927.25	12655.8	Up	0.503511	0.747784	Glutamate--cysteine ligase regulatory subunit OS=Mus musculus GN=Gclm PE=2 SV=1
Pdhx	Q8BKZ9	10	10	347564.8	354337	284545	373545.7	331876.9	358134.1	302666.9	268470	267861	343310.8	0.243315	0.613831	338373.9	308088.6	Down	-0.13527	0.74999	Pyruvate dehydrogenase protein X component, mitochondrial OS=Mus musculus GN=Pdhx PE=1 SV=1
Aimp1	P31230	1	1	1398273	682330	666632	631590	785935	790857	675719	624582	551746	517833	0.243676	0.613187	832952	632147.4	Down	-0.39797	0.74999	Aminoacyl tRNA synthase complex-interacting multifunctional protein 1 OS=Mus musculus GN=Aimp1 PE=1 SV=2
Dst	Q91ZU6-3	6	6	52218.5	93225.33	78757	103518.2	98861	96274.5	86718.5	106180.3	92326.33	114277	0.23465	0.629579	85316	99155.33	Up	0.216874	0.747784	Isoform 3 of Dystonin OS=Mus musculus GN=Dst
Dctn2	Q99KJ8	15	15	514694.1	172909.1	265090.9	214546.1	392586	290980.4	208713.2	269144.5	207396.5	139158.2	0.244863	0.611077	311965.2	223078.5	Down	-0.48383	0.7525	Dynactin subunit 2 OS=Mus musculus GN=Dctn2 PE=1 SV=3
Hadha	Q8BMS1	21	21	59437.35	76003.5	79618.9	88606.55	86168.75	71162.65	66889.15	75720.9	69984.5	70524.3	0.24525	0.610391	77967.01	70856.3	Down	-0.13797	0.752786	Trifunctional enzyme subunit alpha, mitochondrial OS=Mus musculus GN=Hadha PE=1 SV=1
Ptprz1	B9EKR1	9	9	321065.8	237957.6	221500.8	286295.5	308143.1	239196.8	252420.6	202986.1	272271.8	262602.6	0.246331	0.608481	274992.5	245895.6	Down	-0.16135	0.754108	Receptor-type tyrosine-protein phosphatase zeta OS=Mus musculus GN=Ptprz1 PE=1 SV=1
Nbea	Q9EPN1-4	5	5	16270.2	50392.4	53582.4	49934.6	60721.2	58344.8	69674	54065.4	63921.2	61196	0.234723	0.629444	49180.16	61440.28	Up	0.321108	0.747784	Isoform 4 of Neurobeachin OS=Mus musculus GN=Nbea
Ppif	Q99KR7	6	6	1190412	203879	472591.6	321055.8	643177.4	531866.6	374669.4	379001.2	230499.4	96519.8	0.246754	0.607736	566223.1	322511.3	Down	-0.81202	0.754108	Peptidyl-prolyl cis-trans isomerase F, mitochondrial OS=Mus musculus GN=Ppif PE=1 SV=1
Psmb7	P70195	2	2	NA	16193	18667	27744	17667.5	24623.5	12709.5	15429	12492	12972.5	0.247401	0.606599	20067.88	15645.3	Down	-0.35916	0.754108	Proteasome subunit beta type-7 OS=Mus musculus GN=Psmb7 PE=1 SV=1
Mtpap	Q9D0D3	1	1	35621	78079	48006	63179	61129	57440	43973	42900	42773	48602	0.247436	0.606537	57202.8	47137.6	Down	-0.27921	0.754108	Poly(A) RNA polymerase, mitochondrial OS=Mus musculus GN=Mtpap PE=2 SV=1
Rnf214	Q8BFU3-3	7	7	38623.33	57624.83	60549.17	59508.5	69415	87018.17	80002.5	77653.5	55030.17	46551.67	0.235098	0.628751	57144.17	69251.2	Up	0.277233	0.747784	Isoform 3 of RING finger protein 214 OS=Mus musculus GN=Rnf214
Kcnh2	O35219-3	1	1	23531	23771	21446	20448	31023	33247	29994	24347	21575	28904	0.236005	0.627079	24043.8	27613.4	Up	0.199704	0.747784	Isoform 3 of Potassium voltage-gated channel subfamily H member 2 OS=Mus musculus GN=Kcnh2
Crkl	P47941	2	2	2282770	1511888	1572046	1385632	1679027	1687390	1610807	1406515	1379084	1215052	0.249055	0.603705	1686272	1459769	Down	-0.2081	0.754108	Crk-like protein OS=Mus musculus GN=Crkl PE=1 SV=2
Agap3	Q8VHH5	3	3	366490	311539	340468	348320	408195.7	332370	382901	333376.3	309068	214879	0.249253	0.60336	355002.5	314518.9	Down	-0.17468	0.754108	Arf-GAP with GTPase, ANK repeat and PH domain-containing protein 3 OS=Mus musculus GN=Agap3 PE=1 SV=1
Iqsec1	Q8R0S2	5	5	296294	542498.4	495592	417197.6	425951.6	478184.8	539422.2	530548	472425.6	457328.6	0.236185	0.626748	435506.7	495581.8	Up	0.186428	0.747784	IQ motif and SEC7 domain-containing protein 1 OS=Mus musculus GN=Iqsec1 PE=1 SV=2
Eif3f	Q9DCH4	1	1	84394	98970	108991	93252	99632	116887	90770	128920	114964	90491	0.236353	0.626439	97047.8	108406.4	Up	0.159683	0.747784	Eukaryotic translation initiation factor 3 subunit F OS=Mus musculus GN=Eif3f PE=1 SV=2
Pitpnb	P53811	1	1	NA	53800	13478	57028	14301	52738	54272	51115	48856	54848	0.236653	0.625888	34651.75	52365.8	Up	0.595697	0.747784	Phosphatidylinositol transfer protein beta isoform OS=Mus musculus GN=Pitpnb PE=1 SV=2
Hspa1a	Q61696	6	6	1037488	1929039	1593193	1719464	1687299	1836511	1632835	1718291	1782227	2109050	0.236978	0.625292	1593296	1815783	Up	0.188577	0.747784	Heat shock 70 kDa protein 1A OS=Mus musculus GN=Hspa1a PE=1 SV=2
Kcmf1	Q80UY2	2	2	51024	55047	41051	40829	39179.5	47882	45292	40274.5	36177.5	28286	0.250444	0.601289	45426.1	39582.4	Down	-0.19866	0.754108	E3 ubiquitin-protein ligase KCMF1 OS=Mus musculus GN=Kcmf1 PE=2 SV=1
Abcb8	Q9CXJ4	1	1	NA	26876	52427	22009	16829	NA	17181	24474	10229	20579	0.25079	0.60069	29535.25	18115.75	Down	-0.70519	0.754108	ATP-binding cassette sub-family B member 8, mitochondrial OS=Mus musculus GN=Abcb8 PE=2 SV=1
Slk	O54988	3	3	181732.7	284392.7	243475.3	253081	299436.7	272976.3	238870.7	259229	372439.3	319470.7	0.238284	0.622905	252423.7	292597.2	Up	0.213069	0.747784	STE20-like serine/threonine-protein kinase OS=Mus musculus GN=Slk PE=1 SV=2
Itpa	Q9D892	3	3	639109.7	372010.3	429160	333882.7	430665.7	437404.3	409397.3	357444.3	346126.7	275797.3	0.251136	0.600091	440965.7	365234	Down	-0.27185	0.754108	Inosine triphosphate pyrophosphatase OS=Mus musculus GN=Itpa PE=1 SV=2
Napg	Q9CWZ7	22	22	630689.1	415071.8	366327.8	364234.9	445382.3	429038.3	394495.1	387411.1	343180.1	329189.3	0.251202	0.599977	444341.2	376662.8	Down	-0.23839	0.754108	Gamma-soluble NSF attachment protein OS=Mus musculus GN=Napg PE=1 SV=1
Tcerg1	Q8CGF7-3	1	1	NA	49079	36370	49596	37441	NA	33190	83794	47441	83744	0.240271	0.619299	43121.5	62042.25	Up	0.524844	0.747784	Isoform 3 of Transcription elongation regulator 1 OS=Mus musculus GN=Tcerg1
Ndufb10	Q9DCS9	10	10	30103.5	40175.7	31234.1	35826.7	35133.4	44146.3	39913.4	33366.4	35961.8	35670.7	0.240281	0.619281	34494.68	37811.72	Up	0.13246	0.747784	NADH dehydrogenase [ubiquinone] 1 beta subcomplex subunit 10 OS=Mus musculus GN=Ndufb10 PE=1 SV=3
Ak1	Q9R0Y5	10	10	558975	221483.8	321108.8	250617.5	381226.5	381050.5	274695.3	263534.3	223776.7	145861	0.252349	0.597998	346682.3	257783.6	Down	-0.42745	0.754993	Adenylate kinase isoenzyme 1 OS=Mus musculus GN=Ak1 PE=1 SV=1
Snx6	Q6P8X1	5	5	83074.75	78372.5	68723.25	71291.75	80556	64290.5	92461.25	76109.5	111924.3	94930.25	0.240725	0.618479	76403.65	87943.15	Up	0.20293	0.747784	Sorting nexin-6 OS=Mus musculus GN=Snx6 PE=1 SV=2
Msn	P26041	3	3	510542	494970.3	529760	579063	451214	500141	644426.7	537930.7	657448.3	491593.7	0.240829	0.618291	513109.9	566308.1	Up	0.142319	0.747784	Moesin OS=Mus musculus GN=Msn PE=1 SV=3
Ecsit	Q9QZH6	1	1	99266	100245	108498	139328	164613	63814	111584	113951	113871	109134	0.253039	0.596813	122390	102470.8	Down	-0.25627	0.754993	Evolutionarily conserved signaling intermediate in Toll pathway, mitochondrial OS=Mus musculus GN=Ecsit PE=1 SV=2
Igsf21	Q7TNR6	3	3	70281	243344	212403	207198	182844	261595	184091.5	282908.5	217124.5	197178	0.241106	0.617792	183214	228579.5	Up	0.319166	0.747784	Immunoglobulin superfamily member 21 OS=Mus musculus GN=Igsf21 PE=2 SV=1
Dst	Q91ZU6-4	7	7	44758.71	79907.43	69328.71	88729.86	86967.29	86232.43	74330.14	91011.71	79136.86	97951.71	0.241383	0.617293	73938.4	85732.57	Up	0.21352	0.747784	Isoform 4 of Dystonin OS=Mus musculus GN=Dst
Golga5	Q9QYE6	1	1	93592	92409	103193	105597	144313	97592	87600	95508	93603	101283	0.255825	0.592057	107820.8	95117.2	Down	-0.18086	0.755265	Golgin subfamily A member 5 OS=Mus musculus GN=Golga5 PE=1 SV=2
Sash1	P59808	2	2	24682	47796	46024.5	43570.5	50678.5	18448.5	39017.5	47783.5	34042	32782.5	0.255829	0.59205	42550.3	34414.8	Down	-0.30614	0.755265	SAM and SH3 domain-containing protein 1 OS=Mus musculus GN=Sash1 PE=1 SV=1
Cst3	P21460	5	5	164212.8	286892.2	221571.4	215146	260134.2	287995	230960.8	235539	245608.6	319808	0.241461	0.617153	229591.3	263982.3	Up	0.201373	0.747784	Cystatin-C OS=Mus musculus GN=Cst3 PE=2 SV=2
Scrn2	Q8VCA8	1	1	35511	46218	48344	33827	42969	36536	51390	43480	49989	51294	0.241535	0.61702	41373.8	46537.8	Up	0.169686	0.747784	Secernin-2 OS=Mus musculus GN=Scrn2 PE=2 SV=1
Rab8a	P55258	1	1	NA	20156	16263	26979	52148	29530	13696	15671	14413	13707	0.256094	0.591601	28886.5	17403.4	Down	-0.73103	0.755265	Ras-related protein Rab-8A OS=Mus musculus GN=Rab8a PE=1 SV=2
Capns1	O88456	2	2	82591	49847	59442.5	46075.5	60048.5	56223.5	55177.5	56938.5	47055.5	36272.5	0.256752	0.590486	59600.9	50333.5	Down	-0.24382	0.755265	Calpain small subunit 1 OS=Mus musculus GN=Capns1 PE=2 SV=1
Vcl	Q64727	6	6	46108	31383.5	34773.17	32584.17	37360.67	31730.67	31982.67	33091.33	36438.67	31161.5	0.25724	0.589661	36441.9	32880.97	Down	-0.14835	0.755265	Vinculin OS=Mus musculus GN=Vcl PE=1 SV=4
Clpx	Q9JHS4	1	1	65064	19766	14730	31510	40952	NA	30008	18093	26880	13396	0.257278	0.589597	34404.4	22094.25	Down	-0.63892	0.755265	ATP-dependent Clp protease ATP-binding subunit clpX-like, mitochondrial OS=Mus musculus GN=Clpx PE=1 SV=2
Pdzd11	Q9CZG9	1	1	466650	133452	185813	169598	256531	152240	156766	197303	134979	176009	0.257804	0.58871	242408.8	163459.4	Down	-0.56851	0.755265	PDZ domain-containing protein 11 OS=Mus musculus GN=Pdzd11 PE=1 SV=1
Hint2	Q9D0S9	5	5	NA	64128.33	74841.33	104547	80100.33	99909.67	69360.33	263310.7	117493	94064.67	0.241578	0.616943	80904.25	128827.7	Up	0.671155	0.747784	Histidine triad nucleotide-binding protein 2, mitochondrial OS=Mus musculus GN=Hint2 PE=1 SV=1
Ina	P46660	27	27	298741.3	328343.9	341386.9	342353.1	228231.8	317382.8	375057.5	335910	339215.8	325338.7	0.242632	0.615052	307811.4	338580.9	Up	0.137454	0.749236	Alpha-internexin OS=Mus musculus GN=Ina PE=1 SV=3
Vapb	Q9QY76	2	2	70348.5	504360.5	384208.5	464575	310622	417211	469976.5	408614	461097.5	506743.5	0.243754	0.613048	346822.9	452728.5	Up	0.384447	0.74999	Vesicle-associated membrane protein-associated protein B OS=Mus musculus GN=Vapb PE=2 SV=3
Lzts1	P60853	3	3	NA	8901.33	10986.33	7867.33	11051	14892.67	9764.67	9041.33	13898.33	10012.33	0.24661	0.607989	9701.498	11521.87	Up	0.248095	0.754108	Leucine zipper putative tumor suppressor 1 OS=Mus musculus GN=Lzts1 PE=2 SV=3
Mars	Q68FL6	1	1	175980	223470	162442	266291	253851	138413	187569	218154	183903	202665	0.258456	0.587613	216406.8	186140.8	Down	-0.21735	0.755265	Methionine--tRNA ligase, cytoplasmic OS=Mus musculus GN=Mars PE=2 SV=1
Map6	Q7TSJ2-3	6	6	87040.6	18919	38060	39438.4	58221.4	57301.4	33035.4	30026	23068.4	14597.6	0.258543	0.587467	48335.88	31605.76	Down	-0.61291	0.755265	Isoform 3 of Microtubule-associated protein 6 OS=Mus musculus GN=Map6
Cog2	Q921L5	1	1	NA	NA	8297	5860	NA	NA	NA	9235	10154	10154	0.248023	0.605508	7078.5	9847.667	Up	0.476338	0.754108	Conserved oligomeric Golgi complex subunit 2 OS=Mus musculus GN=Cog2 PE=2 SV=2
Lrp1	Q91ZX7	3	3	220935.7	173050.3	194778	168627.7	268120	137700.7	209576.7	191845.7	187052	163704	0.259427	0.585985	205102.3	177975.8	Down	-0.20466	0.755265	Prolow-density lipoprotein receptor-related protein 1 OS=Mus musculus GN=Lrp1 PE=1 SV=1
Mrpl14	Q9D1I6	2	2	448312.5	537038.5	527061.5	508402.5	599338	558587.5	500491	508965	386751.5	426388	0.259463	0.585925	524030.6	476236.6	Down	-0.13797	0.755265	39S ribosomal protein L14, mitochondrial OS=Mus musculus GN=Mrpl14 PE=2 SV=1
Nucb1	Q02819	4	4	55908.25	112788.8	149464.8	94220.75	155489	182272.8	125253.3	129233	129389	136150.3	0.249006	0.60379	113574.3	140459.7	Up	0.306519	0.754108	Nucleobindin-1 OS=Mus musculus GN=Nucb1 PE=1 SV=2
S100a9	P31725	1	1	3448514	780501	665333	423096	694292	470375	479658	134081	419293	795704	0.26151	0.582512	1202347	459822.2	Down	-1.38671	0.755265	Protein S100-A9 OS=Mus musculus GN=S100a9 PE=1 SV=3
Eif4e2	Q8BMB3	2	2	76650	80234	75874	90730	63952	125808.5	68463	94881	88712	78196.5	0.249511	0.60291	77488	91212.2	Up	0.235254	0.754108	Eukaryotic translation initiation factor 4E type 2 OS=Mus musculus GN=Eif4e2 PE=1 SV=1
Syn1	O88935-1	56	56	644982.4	481241.6	402131.4	434751	527478.3	556400.8	615315	494393.4	592647.3	534678.9	0.24968	0.602616	498117	558687.1	Up	0.165556	0.754108	Isoform Ib of Synapsin-1 OS=Mus musculus GN=Syn1
Cpne9	Q1RLL3	1	1	167385	184528	184135	140216	195812	163967	156807	168872	136525	174688	0.263147	0.579802	174415.2	160171.8	Down	-0.12291	0.755265	Copine-9 OS=Mus musculus GN=Cpne9 PE=2 SV=1
Soga3	Q6NZL0	8	8	76726.62	199957.4	179555.5	174621.6	162276.8	172329.3	197243.9	204263.5	194200.8	171516	0.250255	0.601617	158627.6	187910.7	Up	0.244403	0.754108	Protein SOGA3 OS=Mus musculus GN=Soga3 PE=2 SV=2
Camk2d	Q6PHZ2-4	17	17	302535.8	1347016	1438110	1303247	1030992	1279582	1351423	1455386	1273219	1461593	0.250423	0.601326	1084380	1364241	Up	0.331228	0.754108	Isoform 3 of Calcium/calmodulin-dependent protein kinase type II subunit delta OS=Mus musculus GN=Camk2d
Immt	Q8CAQ8-3	14	14	156962.3	207009.5	164062.7	189273.4	186012.5	187160.6	182576.5	197189.6	192949	207268.8	0.251079	0.60019	180664.1	193428.9	Up	0.098494	0.754108	Isoform 3 of Mitochondrial inner membrane protein OS=Mus musculus GN=Immt
Syt3	O35681	1	1	24934	36219	24679	27987	45471	40047	35393	37346	36209	37239	0.251271	0.599858	31858	37246.8	Up	0.225461	0.754108	Synaptotagmin-3 OS=Mus musculus GN=Syt3 PE=1 SV=2
Arhgap39	P59281	1	1	121713	314507	406230	330042	336440	323210	356233	425205	321894	418274	0.252072	0.598475	301786.4	368963.2	Up	0.289949	0.754993	Rho GTPase-activating protein 39 OS=Mus musculus GN=Arhgap39 PE=1 SV=2
Mtfr1l	Q9CWE0	3	3	512432.5	389468.5	353240	309894	348654.5	406978.5	319325	325794	293939	324134.5	0.264293	0.577914	382737.9	334034.2	Down	-0.19636	0.755265	Mitochondrial fission regulator 1-like OS=Mus musculus GN=Mtfr1l PE=1 SV=1
Armt1	A6H630	1	1	3548	5892	8221	6076	5867	6205	7144	8782	6472	6473	0.252857	0.597125	5920.8	7015.2	Up	0.244692	0.754993	UPF0364 protein C6orf211 homolog OS=Mus musculus GN= PE=2 SV=1
Plxnc1	Q9QZC2	1	1	52806	17545	60241	46206	54295	24137	59893	25627	39368	18824	0.264607	0.577399	46218.6	33569.8	Down	-0.46131	0.755265	Plexin-C1 OS=Mus musculus GN=Plxnc1 PE=1 SV=1
3-Sep	Q9Z1S5	15	15	676121	631575.4	525085.9	652304.3	500073.9	576775.7	629169.4	593220.9	728353.1	801356.9	0.252866	0.59711	597032.1	665775.2	Up	0.157227	0.754993	Neuronal-specific septin-3 OS=Mus musculus GN=Sept3 PE=1 SV=2
Gldc	Q91W43	1	1	545525	163058	203087	198019	232599	182332	209087	205293	178793	105975	0.265	0.576754	268457.6	176296	Down	-0.60669	0.755265	Glycine dehydrogenase (decarboxylating), mitochondrial OS=Mus musculus GN=Gldc PE=1 SV=1
Syn1	O88935	59	59	635388.4	473685.8	396578.3	427766.6	518807.7	547503	605001.7	486439.8	582414	525701.2	0.254092	0.595009	490445.4	549411.9	Up	0.163796	0.755265	Synapsin-1 OS=Mus musculus GN=Syn1 PE=1 SV=2
Hmgcl	P38060	4	4	33515	46572.5	49268.5	44625	36412.25	52799	44256.25	37333.25	53669.25	48481.75	0.255522	0.592572	42078.65	47307.9	Up	0.168993	0.755265	Hydroxymethylglutaryl-CoA lyase, mitochondrial OS=Mus musculus GN=Hmgcl PE=1 SV=2
Dync1h1	Q9JHU4	15	15	15894.9	92155.6	87805.3	84309.1	83615.7	79470.7	100476.3	96983.1	90054.3	92645.8	0.255988	0.59178	72756.12	91926.04	Up	0.337405	0.755265	Cytoplasmic dynein 1 heavy chain 1 OS=Mus musculus GN=Dync1h1 PE=1 SV=2
Map7d1	A2AJI0	1	1	NA	334889	369101	308846	365091	391446	296638	301402	311643	271055	0.266577	0.574177	344481.8	314436.8	Down	-0.13166	0.755265	MAP7 domain-containing protein 1 OS=Mus musculus GN=Map7d1 PE=1 SV=1
Snta1	Q61234	2	2	69528	180427.5	99753	122105.5	132285.5	164352.5	118902.5	142721	152328	152374	0.256023	0.591721	120819.9	146135.6	Up	0.27445	0.755265	Alpha-1-syntrophin OS=Mus musculus GN=Snta1 PE=1 SV=1
Dnm1	P39053-4	38	38	434881.6	338158.5	396697.2	354807.4	361945.1	341483.6	396066.7	322759.7	367484.4	326359.1	0.266865	0.573708	377298	350830.7	Down	-0.10493	0.755265	Isoform 4 of Dynamin-1 OS=Mus musculus GN=Dnm1
Gfod1	Q3UHD2	1	1	320813	185076	215301	221601	275810	184019	257557	195070	233654	173718	0.267178	0.573199	243720.2	208803.6	Down	-0.22308	0.755265	Glucose-fructose oxidoreductase domain-containing protein 1 OS=Mus musculus GN=Gfod1 PE=2 SV=1
Tpp1	O89023	1	1	NA	67839	27691	19358	75114	116371	65558	67590	48573	54036	0.257937	0.588486	47500.5	70425.6	Up	0.568157	0.755265	Tripeptidyl-peptidase 1 OS=Mus musculus GN=Tpp1 PE=1 SV=2
Eef1b2	O70251	4	4	40206.5	38015.5	44850	46156.75	52334.5	56623.5	51172.5	49406.75	48696.5	38882.5	0.258056	0.588286	44312.65	48956.35	Up	0.143777	0.755265	Elongation factor 1-beta OS=Mus musculus GN=Eef1b PE=1 SV=5
Tmcc1	Q69ZZ6-3	1	1	143034	256343	212631	214995	253922	268865	202750	307325	219056	251033	0.258223	0.588005	216185	249805.8	Up	0.208541	0.755265	Isoform 3 of Transmembrane and coiled-coil domains protein 1 OS=Mus musculus GN=Tmcc1
Eef1g	Q9D8N0	2	2	76976.5	180435.5	162480.5	149711	167176.5	174181	168081	178611	165481.5	171041.5	0.258315	0.58785	147356	171479.2	Up	0.218728	0.755265	Elongation factor 1-gamma OS=Mus musculus GN=Eef1g PE=1 SV=3
Cep170	Q6A065-3	1	1	115811	69692	67969	52692	29327	54597	34538	21159	27323	87319	0.268523	0.571019	67098.2	44987.2	Down	-0.57676	0.755265	Isoform 3 of Centrosomal protein of 170 kDa OS=Mus musculus GN=Cep170
Gpt	Q8QZR5	1	1	87319	180619	204831	211621	165882	194830	240954	204836	189501	175511	0.258702	0.5872	170054.4	201126.4	Up	0.242106	0.755265	Alanine aminotransferase 1 OS=Mus musculus GN=Gpt PE=2 SV=3
Atg7	Q9D906	1	1	107338	112572	151383	143645	171530	145345	164662	143752	179668	140660	0.259511	0.585844	137293.6	154817.4	Up	0.173303	0.755265	Ubiquitin-like modifier-activating enzyme ATG7 OS=Mus musculus GN=Atg7 PE=1 SV=1
Sgtb	Q8VD33	1	1	119694	104713	84784	97822	116767	108667	82604	113377	82455	78896	0.269612	0.569261	104756	93199.8	Down	-0.16863	0.755265	Small glutamine-rich tetratricopeptide repeat-containing protein beta OS=Mus musculus GN=Sgtb PE=2 SV=1
Copa	Q8CIE6	2	2	NA	69823	55903.5	48712.5	67578	61978.5	80316.5	68542	71824.5	58671.5	0.261552	0.582442	60504.25	68266.6	Up	0.174143	0.755265	Coatomer subunit alpha OS=Mus musculus GN=Copa PE=1 SV=2
Metap2	O08663	3	3	135092.3	64886	48591.33	65299	81775	81136.67	43177	63244.67	59269	48797.67	0.270323	0.568117	79128.73	59125	Down	-0.42043	0.755265	Methionine aminopeptidase 2 OS=Mus musculus GN=Metap2 PE=1 SV=1
Ap2s1	P62743	2	2	115203	58114	118278.5	82666.5	128911.5	85062	107095.5	82145.5	83681.5	49292.5	0.270586	0.567695	100634.7	81455.4	Down	-0.30505	0.755265	AP-2 complex subunit sigma OS=Mus musculus GN=Ap2s1 PE=1 SV=1
Hist1h1e	P43274	13	13	336397.7	273532	513580.9	395821.9	770636.1	221624.4	433838	380463.5	366963.4	305169.5	0.271149	0.566792	457993.7	341611.8	Down	-0.42297	0.755265	Histone H1.4 OS=Mus musculus GN=Hist1h1e PE=1 SV=2
Aars2	Q14CH7	1	1	66267	66591	90298	107982	87676	34957	82453	82260	72913	75989	0.271418	0.566361	83762.8	69714.4	Down	-0.26485	0.755265	Alanine--tRNA ligase, mitochondrial OS=Mus musculus GN=Aars2 PE=2 SV=1
Brsk1	Q5RJI5	2	2	60361	92285	98816	67498	65529	89154	84112	82907	80897	100735	0.261907	0.581853	76897.8	87561	Up	0.187346	0.755265	Serine/threonine-protein kinase BRSK1 OS=Mus musculus GN=Brsk1 PE=1 SV=1
Map2	P20357	36	36	375570.2	174176.1	236343	196096	309238.6	305454.6	198023.9	212165.2	164814.8	128931.9	0.271704	0.565904	258284.8	201878.1	Down	-0.35548	0.755265	Microtubule-associated protein 2 OS=Mus musculus GN=Map2 PE=1 SV=2
Snap47	Q8R570	2	2	190762	154514	232480	244657	233528	176679	226326	174319	205377	142176	0.272103	0.565267	211188.2	184975.4	Down	-0.1912	0.755265	Synaptosomal-associated protein 47 OS=Mus musculus GN=Snap47 PE=1 SV=1
Snap47	Q8R570-2	1	1	190762	154514	232480	244657	233528	176679	226326	174319	205377	142176	0.272103	0.565267	211188.2	184975.4	Down	-0.1912	0.755265	Isoform 2 of Synaptosomal-associated protein 47 OS=Mus musculus GN=Snap47
Got1	P05201	7	7	530351.6	608046.3	659084.3	593448.3	456801.6	629968.3	684124.3	667319.7	598276	533811.6	0.26326	0.579615	569546.4	622700	Up	0.128724	0.755265	Aspartate aminotransferase, cytoplasmic OS=Mus musculus GN=Got1 PE=1 SV=3
Nebl	Q9DC07	6	6	513892.8	746538	476825.7	603275.8	454013.8	575665.2	612767.8	605251.2	623719.3	755982.3	0.263563	0.579116	558909.2	634677.2	Up	0.183409	0.755265	LIM zinc-binding domain-containing Nebulette OS=Mus musculus GN=Nebl PE=2 SV=1
Cit	P49025-4	2	2	NA	65913	59918	81349.5	73354.5	119020	78000	74699	66854.5	75934.5	0.264102	0.578228	70133.75	82901.6	Up	0.241291	0.755265	Isoform 4 of Citron Rho-interacting kinase OS=Mus musculus GN=Cit
Serpinh1	P19324	2	2	NA	12959.5	19221	16369	29332	14243	24858.5	30338	26003.5	29951.5	0.264118	0.578202	19470.38	25078.9	Up	0.365193	0.755265	Serpin H1 OS=Mus musculus GN=Serpinh1 PE=1 SV=3
Nifk	Q91VE6	1	1	140068	162367	124129	140731	219854	71588	143046	183486	134389	107224	0.273767	0.562619	157429.8	127946.6	Down	-0.29917	0.755265	MKI67 FHA domain-interacting nucleolar phosphoprotein OS=Mus musculus GN=Nifk PE=1 SV=1
Pfdn6	Q03958	2	2	276766	435119	424454	454562	628747.5	491358.5	481107	482324.5	573894	565731	0.264191	0.578082	443929.7	518883	Up	0.225078	0.755265	Prefoldin subunit 6 OS=Mus musculus GN=Pfdn6 PE=2 SV=1
Ptprd	Q64487-1	1	1	44636	65580	58134	76498	40274	87013	50017	61768	66238	74782	0.264425	0.577697	57024.4	67963.6	Up	0.253183	0.755265	Isoform K of Receptor-type tyrosine-protein phosphatase delta OS=Mus musculus GN=Ptprd
Rock2	P70336	4	4	131298.8	83606.75	133223.8	108260.3	127452.8	122626	103325.5	118107	92720.75	65956	0.274997	0.560672	116768.5	100547.1	Down	-0.21578	0.755265	Rho-associated protein kinase 2 OS=Mus musculus GN=Rock2 PE=1 SV=1
Me2	Q99KE1	2	2	89562	266199	171354	234970	163371	203031	185875	236710	228238	287057	0.264975	0.576795	185091.2	228182.2	Up	0.30195	0.755265	NAD-dependent malic enzyme, mitochondrial OS=Mus musculus GN=Me2 PE=2 SV=1
Actbl2	Q8BFZ3	5	5	762686	929039.7	724292.3	1047337	828089	803753	848753.3	902640.7	1144586	1176564	0.265282	0.576292	858288.8	975259.3	Up	0.184323	0.755265	Beta-actin-like protein 2 OS=Mus musculus GN=Actbl2 PE=1 SV=1
Prdx5	P99029	43	43	1476857	1086812	975888.4	1253331	1149957	1284481	1011405	1045399	1012037	991890.7	0.276102	0.55893	1188569	1069042	Down	-0.15291	0.755265	Peroxiredoxin-5, mitochondrial OS=Mus musculus GN=Prdx5 PE=1 SV=2
Hnrnpdl	Q9Z130	1	1	170677	711776	598301	537330	599689	340904	710476	725196	694754	955382	0.265707	0.575597	523554.6	685342.4	Up	0.388485	0.755265	Heterogeneous nuclear ribonucleoprotein D-like OS=Mus musculus GN=Hnrnpdl PE=1 SV=1
Rpl35	Q6ZWV7	4	4	1053610	450616.3	526656	448848.8	515179.3	628790.8	476795.8	427678.8	387694.5	312711	0.27651	0.558289	599982	446734.2	Down	-0.4231	0.755265	60S ribosomal protein L35 OS=Mus musculus GN=Rpl35 PE=2 SV=1
Pgk2	P09041	3	3	189395.5	261232	205414	166529.5	217387	176411.5	182398	204313.5	172006	201190.5	0.27653	0.558258	207993.6	187263.9	Down	-0.15147	0.755265	Phosphoglycerate kinase 2 OS=Mus musculus GN=Pgk2 PE=1 SV=1
COX2	P00405	2	2	42480.5	213163	264160.5	274701.5	264720	285353.5	258676.5	274036	264439.5	259567.5	0.266048	0.57504	211845.1	268414.6	Up	0.341453	0.755265	Cytochrome c oxidase subunit 2 OS=Mus musculus GN=Mtco2 PE=1 SV=1
Adgrl2	Q8JZZ7	1	1	NA	478493	476868	498163	685126	658371	619821	531877	653233	566499	0.266638	0.574078	534662.5	605960.2	Up	0.180595	0.755265	Latrophilin-2 (Fragment) OS=Mus musculus GN=Lphn2 PE=1 SV=2
Syne1	Q6ZWR6-3	1	1	NA	27019	47877	32305	87670	41991	22210	29291	36671	21761	0.277542	0.556671	48717.75	30384.8	Down	-0.6811	0.755265	Isoform 3 of Nesprin-1 OS=Mus musculus GN=Syne1
Dync1li2	Q6PDL0	7	7	175677.4	73670.8	101155.4	101533.2	128450.4	117300.2	107985.8	90117.6	84227.6	64270.8	0.277555	0.556651	116097.4	92780.4	Down	-0.32344	0.755265	Cytoplasmic dynein 1 light intermediate chain 2 OS=Mus musculus GN=Dync1li2 PE=1 SV=2
Prkca	P20444	1	1	NA	5362	5928	5726	6996	12861	NA	8254	6030	6030	0.267465	0.572733	6003	9048.333	Up	0.591968	0.755265	Protein kinase C alpha type OS=Mus musculus GN=Prkca PE=1 SV=3
Apip	Q9WVQ5	1	1	205218	177322	148098	156549	160329	179282	162472	156828	225637	254520	0.268312	0.57136	169503.2	195747.8	Up	0.207684	0.755265	Methylthioribulose-1-phosphate dehydratase OS=Mus musculus GN=Apip PE=1 SV=1
Hmbs	P22907	4	4	109607.3	72410.25	89387.5	75067.25	105501.5	87725.25	93097	80185.75	76250.75	58220.75	0.278312	0.555468	90394.75	79095.9	Down	-0.19264	0.755265	Porphobilinogen deaminase OS=Mus musculus GN=Hmbs PE=1 SV=2
Magi3	Q9EQJ9	4	4	131546.5	49097.5	81951.25	59061	113606.5	97459.25	57812.75	73017.5	63286.5	32582.25	0.278597	0.555024	87052.55	64831.65	Down	-0.42519	0.755265	Membrane-associated guanylate kinase, WW and PDZ domain-containing protein 3 OS=Mus musculus GN=Magi3 PE=1 SV=2
Syn1	O88935-3	49	49	779495.4	561450.8	472176.8	512158.8	618618.7	659149.5	734298.8	583584.6	704583.7	628419.9	0.268396	0.571224	588780.1	662007.3	Up	0.169118	0.755265	Isoform 3 of Synapsin-1 OS=Mus musculus GN=Syn1
Dpysl5	Q9EQF6	4	4	390798.5	697302.8	699594.5	715287.5	606208	745176.3	593872.5	711782.8	705301.3	767856.8	0.268515	0.571031	621838.3	704797.9	Up	0.18067	0.755265	Dihydropyrimidinase-related protein 5 OS=Mus musculus GN=Dpysl5 PE=1 SV=1
Vamp1	Q62442	1	1	NA	NA	11742	NA	15889	14722	6524	NA	9169	9980	0.279379	0.553806	13815.5	10098.75	Down	-0.45211	0.755265	Vesicle-associated membrane protein 1 OS=Mus musculus GN=Vamp1 PE=1 SV=1
Ezr	P26040	6	6	344829.2	324150	337619	373148.2	311901.5	344624.8	384480.2	356445.3	396017.7	315814	0.269068	0.570138	338329.6	359476.4	Up	0.087468	0.755265	Ezrin OS=Mus musculus GN=Ezr PE=1 SV=3
Ccdc127	Q3TC33	1	1	NA	49231	25985	30053	36533	115592	81097	32223	26235	35430	0.269478	0.569477	35450.5	58115.4	Up	0.713115	0.755265	Coiled-coil domain-containing protein 127 OS=Mus musculus GN=Ccdc127 PE=2 SV=2
Fam136a	Q9CR98	1	1	603631	307708	387715	314508	370118	395269	320326	379007	280235	248647	0.280977	0.551329	396736	324696.8	Down	-0.28909	0.755265	Protein FAM136A OS=Mus musculus GN=Fam136a PE=2 SV=1

Efhd1	Q9D4J1	1	1	2909511	3349834	2609547	2720574	2022609	2096511	2738270	2172453	2802513	2277781	0.281028	0.55125	2722415	2417506	Down	-0.17137	0.755265	EF-hand domain-containing protein D1 OS=Mus musculus GN=Efhd1 PE=2 SV=1
Nkiras2	Q9CR56	1	1	431599	260260	211285	209501	219626	229562	225228	205786	181239	225171	0.282022	0.549717	266454.2	213397.2	Down	-0.32035	0.755265	NF-kappa-B inhibitor-interacting Ras-like protein 2 OS=Mus musculus GN=Nkiras2 PE=2 SV=1
Lmtk3	Q5XJV6	1	1	167520	405641	318773	292592	486180	469065	367006	455057	349946	390411	0.269966	0.568691	334141.2	406297	Up	0.282077	0.755265	Serine/threonine-protein kinase LMTK3 OS=Mus musculus GN=Lmtk3 PE=1 SV=1
Aldh9a1	Q9JLJ2	3	3	85765	36904.67	58166.67	52246	64813	46925.67	61607	49395.33	50646.33	36238	0.282258	0.549354	59579.07	48962.47	Down	-0.28313	0.755265	4-trimethylaminobutyraldehyde dehydrogenase OS=Mus musculus GN=Aldh9a1 PE=1 SV=1
Ppa1	Q9D819	5	5	21712.5	76164.5	65933.5	65811.75	41384.25	48638	54830	75924	78379.5	86386.75	0.271562	0.566131	54201.3	68831.65	Up	0.344745	0.755265	Inorganic pyrophosphatase OS=Mus musculus GN=Ppa1 PE=1 SV=1
Nhp2l1	Q9D0T1	2	2	128539.5	141705	151582.5	161077	191021	159443	154469	175905.5	183159	172405	0.272118	0.565243	154785	169076.3	Up	0.127409	0.755265	NHP2-like protein 1 OS=Mus musculus GN=Nhp2l1 PE=1 SV=4
Garem1	Q3UFT3	1	1	NA	NA	3595	2625	3314	3294	5005	4648	2949	3100	0.273112	0.563659	3178	3799.2	Up	0.257577	0.755265	GRB2-associated and regulator of MAPK protein OS=Mus musculus GN=Garem PE=2 SV=2
Erlec1	Q8VEH8	2	2	3905.5	12004.5	11172	10442	6692.5	11958.5	9270	12633	14456.5	7513	0.273425	0.563162	8843.3	11166.2	Up	0.336482	0.755265	Endoplasmic reticulum lectin 1 OS=Mus musculus GN=Erlec1 PE=2 SV=1
Chid1	Q922Q9	2	2	109508	156251	147487	111129	139362	77949	155025	72338	137182	111609	0.283393	0.547611	132747.4	110820.6	Down	-0.26046	0.755265	Chitinase domain-containing protein 1 OS=Mus musculus GN=Chid1 PE=2 SV=1
Glg1	Q61543	5	5	88897.4	67780.4	92964.4	94071.2	138765.4	100879.4	89293	76941.6	84901.6	43169.8	0.283571	0.547338	96495.76	79037.08	Down	-0.28794	0.755265	Golgi apparatus protein 1 OS=Mus musculus GN=Glg1 PE=1 SV=1
Cyfip2	Q5SQX6	2	2	124735.5	424913	352594	447970.5	299214.5	345332	454116.5	351099.5	438037	441546	0.273532	0.562992	329885.5	406026.2	Up	0.299607	0.755265	Cytoplasmic FMR1-interacting protein 2 OS=Mus musculus GN=Cyfip2 PE=1 SV=2
Rab11fip2	G3XA57	3	3	8938.33	18175.33	54626.33	30430.33	16972.67	43620.33	30089.67	43055.67	33690	30843	0.27392	0.562376	25828.6	36259.73	Up	0.489399	0.755265	Rab11 family-interacting protein 2 OS=Mus musculus GN=Rab11fip2 PE=1 SV=1
Ppp1r9b	Q6R891-2	4	4	61792.5	80313.5	52852	69327	56029	99578.5	67271	55077	82833	69389	0.274005	0.562242	64062.8	74829.7	Up	0.224124	0.755265	Isoform 2 of Neurabin-2 OS=Mus musculus GN=Ppp1r9b
Tomm40	Q9QYA2	1	1	NA	47054	19822	58978	16670	51960	53711	62573	38897	41649	0.275345	0.560123	35631	49758	Up	0.481796	0.755265	Mitochondrial import receptor subunit TOM40 homolog OS=Mus musculus GN=Tomm40 PE=1 SV=3
Ass1	P16460	1	1	61788	203917	203241	211604	99815	152339	230773	250833	161496	202802	0.27606	0.558997	156073	199648.6	Up	0.355242	0.755265	Argininosuccinate synthase OS=Mus musculus GN=Ass1 PE=1 SV=1
1-Sep	P42209	2	2	773251.5	738463.5	488463.5	637032	616654	681080	693642	606737	778850.5	928647.5	0.27636	0.558525	650772.9	737791.4	Up	0.181059	0.755265	Septin-1 OS=Mus musculus GN=Sept1 PE=2 SV=2
Ran	P62827	5	5	165341.2	144114.8	198957	145225	157997	123844.8	147072.8	172666.2	165608.8	118298.2	0.286921	0.542238	162327	145498.2	Down	-0.1579	0.755265	GTP-binding nuclear protein Ran OS=Mus musculus GN=Ran PE=1 SV=3
Nampt	Q99KQ4	2	2	99415	143451.5	149385	168411	140177	136400.5	168915.5	133421	170045.5	177576.5	0.276695	0.557999	140167.9	157271.8	Up	0.166104	0.755265	Nicotinamide phosphoribosyltransferase OS=Mus musculus GN=Nampt PE=1 SV=1
Mrps18c	Q8R2L5	1	1	NA	40894	38369	38402	43671	25553	45738	30643	39887	37492	0.28718	0.541846	40334	35862.6	Down	-0.16952	0.755265	28S ribosomal protein S18c, mitochondrial OS=Mus musculus GN=Mrps18c PE=2 SV=1
Cct4	P80315	5	5	7364.5	10390.75	7590.25	10401.75	8128.75	6033	12527.75	15636.75	8144.25	12818	0.276888	0.557696	8775.2	11031.95	Up	0.330184	0.755265	T-complex protein 1 subunit delta OS=Mus musculus GN=Cct4 PE=1 SV=3
Vps13a	Q5H8C4	1	1	NA	11881	28270	24195	40938	23033	16171	13809	25831	13030	0.288135	0.540404	26321	18374.8	Down	-0.51849	0.755265	Vacuolar protein sorting-associated protein 13A OS=Mus musculus GN=Vps13a PE=2 SV=1
Ckmt2	Q6P8J7	1	1	NA	24006	60397	84986	132310	NA	81066	20953	56284	13136	0.289186	0.538823	75424.75	42859.75	Down	-0.81541	0.755265	Creatine kinase S-type, mitochondrial OS=Mus musculus GN=Ckmt2 PE=1 SV=1
Clstn1	Q9EPL2	1	1	NA	52144	40036	118595	54717	50705	108281	99642	112267	87512	0.277638	0.556521	66373	91681.4	Up	0.466033	0.755265	Calsyntenin-1 OS=Mus musculus GN=Clstn1 PE=1 SV=1
Tmod2	Q9JKK7	12	12	685633.5	323506.4	342770.8	329232.7	408988.1	411117.4	355926.2	306272.3	308459.9	278049.1	0.289378	0.538534	418026.3	331965	Down	-0.33256	0.755265	Tropomodulin-2 OS=Mus musculus GN=Tmod2 PE=1 SV=2
Tmod2	Q9JKK7-3	10	10	685633.5	323506.4	342770.8	329232.7	408988.1	411117.4	355926.2	306272.3	308459.9	278049.1	0.289378	0.538534	418026.3	331965	Down	-0.33256	0.755265	Isoform 3 of Tropomodulin-2 OS=Mus musculus GN=Tmod2
Dlst	Q9D2G2-2	2	2	201557	293496	248818.5	354330.5	233765	189092.5	261262	229691	261342.5	217378	0.28991	0.537737	266393.4	231753.2	Down	-0.20097	0.755265	Isoform 2 of Dihydrolipoyllysine-residue succinyltransferase component of 2-oxoglutarate dehydrogenase complex, mitochondrial OS=Mus musculus GN=Dlst
Msrb2	Q78J03	2	2	349653.5	256841.5	347379	344164	223651	209273.5	317293	240677.5	316912	240979	0.2901	0.537452	304337.8	265027	Down	-0.19953	0.755265	Methionine-R-sulfoxide reductase B2, mitochondrial OS=Mus musculus GN=Msrb2 PE=1 SV=1
Map7d2	A2AG50-3	1	1	100098	184254	125064	91220	106995	183869	104953	135792	156937	149279	0.278108	0.555787	121526.2	146166	Up	0.26634	0.755265	Isoform 3 of MAP7 domain-containing protein 2 OS=Mus musculus GN=Map7d2
Hdlbp	Q8VDJ3	3	3	86097.5	90718	91564	60274	109824	145765	92197.5	108153.5	92750.5	80762	0.278639	0.554958	87695.5	103925.7	Up	0.244978	0.755265	Vigilin OS=Mus musculus GN=Hdlbp PE=1 SV=1
Smap1	Q91VZ6	1	1	405012	167180	172992	231402	201917	222889	219830	133457	184475	137940	0.290563	0.53676	235700.6	179718.2	Down	-0.39122	0.755265	Stromal membrane-associated protein 1 OS=Mus musculus GN=Smap1 PE=1 SV=1
Pgam2	O70250	2	2	219627.5	211117	198342.5	139192	148142.5	190548	153630	178304	137519.5	144992	0.290821	0.536374	183284.3	160998.7	Down	-0.18703	0.755265	Phosphoglycerate mutase 2 OS=Mus musculus GN=Pgam2 PE=1 SV=3
10-Sep	Q8C650	1	1	230447	294903	192309	243498	274038	236157	204170	265213	212740	191857	0.291445	0.535443	247039	222027.4	Down	-0.154	0.755265	Septin-10 OS=Mus musculus GN=Sept10 PE=2 SV=1
Ndufb7	Q9CR61	2	2	765775	418759.5	414116	402475.5	339742	392187	356415	353442.5	416590.5	359779.5	0.291447	0.53544	468173.6	375682.9	Down	-0.31753	0.755265	NADH dehydrogenase [ubiquinone] 1 beta subcomplex subunit 7 OS=Mus musculus GN=Ndufb7 PE=1 SV=3
Gcat	O88986	1	1	75237	71084	67354	87941	59403	67553	59960	72942	65715	64744	0.291741	0.535003	72203.8	66182.8	Down	-0.12562	0.755265	2-amino-3-ketobutyrate coenzyme A ligase, mitochondrial OS=Mus musculus GN=Gcat PE=1 SV=2
Lrrc40	Q9CRC8	3	3	NA	39078.33	36400.67	45931	44904.67	50424	50867	36011	41857.67	51211	0.278892	0.554564	41578.67	46074.13	Up	0.148114	0.755265	Leucine-rich repeat-containing protein 40 OS=Mus musculus GN=Lrrc40 PE=2 SV=2
Hmgn3	Q9DCB1-3	1	1	44171	18402	32801	29682	59379	27608	31871	34743	25643	21206	0.292148	0.534397	36887	28214.2	Down	-0.38669	0.755265	Isoform 3 of High mobility group nucleosome-binding domain-containing protein 3 OS=Mus musculus GN=Hmgn3
Wasl	Q91YD9	6	6	10981.17	58871.33	36095.83	46711	48509.5	56621	39330	52217	48546.83	57461.83	0.279431	0.553725	40233.77	50835.33	Up	0.337425	0.755265	Neural Wiskott-Aldrich syndrome protein OS=Mus musculus GN=Wasl PE=1 SV=1
Sptbn1	Q62261-2	55	55	109050.6	215927.7	199409.7	253029.2	192581.6	181234.2	223529.2	249336.3	256039.4	220184.7	0.279867	0.553048	193999.8	226064.7	Up	0.220681	0.755265	Isoform 2 of Spectrin beta chain, non-erythrocytic 1 OS=Mus musculus GN=Sptbn1
Acsf3	Q3URE1	1	1	4747	6337	5902	8071	5179	7605	6757	7703	5265	7109	0.282164	0.549498	6047.2	6887.8	Up	0.187776	0.755265	Acyl-CoA synthetase family member 3, mitochondrial OS=Mus musculus GN=Acsf3 PE=2 SV=2
Caskin1	Q6P9K8-4	3	3	NA	399912.3	431488.7	401247.7	519284	436565	433347	570042.3	435586	563134.3	0.282374	0.549175	437983.2	487734.9	Up	0.155222	0.755265	Isoform 4 of Caskin-1 OS=Mus musculus GN=Caskin1
Pygm	Q9WUB3	1	1	NA	61406	47834	55289	35243	47793	42743	31343	48836	41079	0.293984	0.531676	49943	42358.8	Down	-0.23762	0.755265	Glycogen phosphorylase, muscle form OS=Mus musculus GN=Pygm PE=1 SV=3
Actn2	Q9JI91	7	7	110311	128926.5	164419.8	166814.8	163992.8	157469.8	181594.5	169628.7	154950.2	149705.5	0.282576	0.548865	147253	162669.7	Up	0.143649	0.755265	Alpha-actinin-2 OS=Mus musculus GN=Actn2 PE=1 SV=2
Dnajb4	Q9D832	2	2	123263	220410.5	216740.5	194544	237909.5	345386.5	203906	224278.5	187540.5	230635	0.282796	0.548527	198573.5	238349.3	Up	0.263404	0.755265	DnaJ homolog subfamily B member 4 OS=Mus musculus GN=Dnajb4 PE=2 SV=1
Plcb1	Q9Z1B3-2	9	9	651764.3	536964.3	618997.7	567725.3	703489	719453.3	562198.7	501718.3	537038.8	452210.2	0.294534	0.530865	615788.1	554529.9	Down	-0.15118	0.755265	Isoform B of 1-phosphatidylinositol 4,5-bisphosphate phosphodiesterase beta-1 OS=Mus musculus GN=Plcb1
Dgkg	Q91WG7	1	1	228856	347116	303388	292374	408633	533834	352831	332703	407790	266658	0.283116	0.548036	316073.4	378763.2	Up	0.261037	0.755265	Diacylglycerol kinase gamma OS=Mus musculus GN=Dgkg PE=2 SV=1
Aamdc	Q8R0P4	2	2	192290	145265	138038	149109.5	207555	181951	138846.5	158295.5	126777.5	127862.5	0.294567	0.530816	166451.5	146746.6	Down	-0.18177	0.755265	Mth938 domain-containing protein OS=Mus musculus GN=Aamdc PE=1 SV=1
Dhdh	Q9DBB8	2	2	4287	4022.5	6386	3168	1179.5	4055.5	8526	4649.5	5827	3181	0.283983	0.546708	3808.6	5247.8	Up	0.462452	0.755265	Trans-1,2-dihydrobenzene-1,2-diol dehydrogenase OS=Mus musculus GN=Dhdh PE=2 SV=1
Ufd1l	P70362	1	1	235958	105929	116015	108101	162681	146509	101527	142163	92371	86186	0.295318	0.52971	145736.8	113751.2	Down	-0.35748	0.755265	Ubiquitin fusion degradation protein 1 homolog OS=Mus musculus GN=Ufd1l PE=1 SV=2
Sugt1	Q9CX34	3	3	NA	138155	113082.5	99409.5	88179	102709.5	96895.5	142490.5	132196	190667	0.284006	0.546672	109706.5	132991.7	Up	0.277687	0.755265	Suppressor of G2 allele of SKP1 homolog OS=Mus musculus GN=Sugt1 PE=2 SV=3
Fbxo38	Q8BMI0	1	1	1065920	929258	995674	998658	819044	1109653	951697	741882	798237	731819	0.295901	0.528854	961710.8	866657.6	Down	-0.15014	0.755265	F-box only protein 38 OS=Mus musculus GN=Fbxo38 PE=1 SV=1
Stx17	Q9D0I4	1	1	332104	336472	196341	255824	214167	385377	266791	281116	279356	333379	0.284604	0.545759	266981.6	309203.8	Up	0.211818	0.755265	Syntaxin-17 OS=Mus musculus GN=Stx17 PE=1 SV=1
Commd3	Q63829	2	2	80146	41731	57919.5	44744.5	61972.5	69959.5	63582	57142	68322	71349	0.284838	0.545402	57302.7	66070.9	Up	0.205412	0.755265	COMM domain-containing protein 3 OS=Mus musculus GN=Commd3 PE=1 SV=1
Stub1	Q9WUD1	1	1	NA	13493	18599	24066	14569	20048	20401	27164	14223	25926	0.28517	0.544896	17681.75	21552.4	Up	0.285587	0.755265	STIP1 homology and U box-containing protein 1 OS=Mus musculus GN=Stub1 PE=1 SV=1
Auh	Q9JLZ3	5	5	761960.4	267237.8	396972.4	329223.8	414192	387792.6	345623.8	360268.2	288456.4	262776.6	0.296596	0.527835	433917.3	328983.9	Down	-0.3994	0.755265	Methylglutaconyl-CoA hydratase, mitochondrial OS=Mus musculus GN=Auh PE=1 SV=1
Nfix	P70257-2	1	1	458855	115655	328141	222775	316634	329691	215169	247459	165273	92693	0.297239	0.526894	288412	210057	Down	-0.45735	0.755265	Isoform NFIX2 of Nuclear factor 1 X-type OS=Mus musculus GN=Nfix
Aldh1l1	Q8R0Y6	14	14	139561.1	92803.5	124181.8	119598.5	111363.6	123438.1	111710.4	103403.4	99446.14	98659.14	0.297277	0.526839	117501.7	107331.4	Down	-0.13061	0.755265	Cytosolic 10-formyltetrahydrofolate dehydrogenase OS=Mus musculus GN=Aldh1l1 PE=1 SV=1
Fkbp8	O35465	1	1	NA	5291	4049	NA		5667	7436	11178	3274	NA	0.285315	0.544675	4670	6888.75	Up	0.56082	0.755265	Peptidyl-prolyl cis-trans isomerase FKBP8 OS=Mus musculus GN=Fkbp8 PE=1 SV=2
Cntnap1	O54991	1	1	NA	29403	50645	66151	93364	NA	27300	58564	55251	23046	0.297402	0.526656	59890.75	41040.25	Down	-0.54529	0.755265	Contactin-associated protein 1 OS=Mus musculus GN=Cntnap1 PE=2 SV=2
Mprip	P97434-3	1	1	182237	237895	207934	230712	200753	189901	212827	244269	244812	261544	0.286951	0.542192	211906.2	230670.6	Up	0.122408	0.755265	Isoform 3 of Myosin phosphatase Rho-interacting protein OS=Mus musculus GN=Mprip
Car2	P00920	3	3	169444	107301.7	183427	120043.7	84779.33	96413.33	91891.33	152099.3	104008	97532.33	0.298484	0.525079	132999.1	108388.9	Down	-0.2952	0.755265	Carbonic anhydrase 2 OS=Mus musculus GN=Ca2 PE=1 SV=4
Rab5a	Q9CQD1	1	1	162572	289990	527589	590365	496498	447971	588084	527696	627547	403998	0.287899	0.54076	413402.8	519059.2	Up	0.328351	0.755265	Ras-related protein Rab-5A OS=Mus musculus GN=Rab5a PE=1 SV=1
Mrps28	Q9CY16	3	3	3603.5	3000.5	2493.5	2927	1592	2712	3048.5	3437	10222.5	3063	0.289363	0.538557	2723.3	4496.6	Up	0.723479	0.755265	28S ribosomal protein S28, mitochondrial OS=Mus musculus GN=Mrps28 PE=1 SV=1
Epb41l1	Q9Z2H5	8	8	195246.4	29111.12	55879.88	31239.12	83005	54149.88	40295.38	49423.88	38640.62	28102.38	0.298883	0.524499	78896.3	42122.43	Down	-0.90537	0.755265	Band 4.1-like protein 1 OS=Mus musculus GN=Epb41l1 PE=1 SV=2
Cdkn2aip	Q8BI72	2	2	110102	168477	136303	144046	199153	249592	156843.5	192616	163090.5	136712	0.290412	0.536985	151616.2	179770.8	Up	0.245735	0.755265	CDKN2A-interacting protein OS=Mus musculus GN=Cdkn2aip PE=2 SV=1
Atp1b1	P14094	10	10	97446.6	349435.4	359160.2	367984.2	327951.2	301596.6	376670.1	371128.4	359946.4	413480.5	0.290467	0.536903	300395.5	364564.4	Up	0.27931	0.755265	Sodium/potassium-transporting ATPase subunit beta-1 OS=Mus musculus GN=Atp1b1 PE=1 SV=1
Grhpr	Q91Z53	2	2	529819	469248.5	502793.5	398567	445193.5	461722.5	449358.5	486957	404628.5	372795.5	0.299346	0.523827	469124.3	435092.4	Down	-0.10865	0.755265	Glyoxylate reductase/hydroxypyruvate reductase OS=Mus musculus GN=Grhpr PE=1 SV=1
11-Sep	Q8C1B7-3	8	8	343907	268658.5	178608.5	246621.5	263603.5	290050.5	195601	205235.9	226252.1	208144	0.29968	0.523342	260279.8	225056.7	Down	-0.20977	0.755265	Isoform 3 of Septin-11 OS=Mus musculus GN=Sept11
Prkaa2	Q8BRK8	2	2	NA	16645.5	18375.5	20163	17548.5	NA	18582.5	15509	18505	12188	0.299689	0.523329	18183.13	16196.13	Down	-0.16695	0.755265	5'-AMP-activated protein kinase catalytic subunit alpha-2 OS=Mus musculus GN=Prkaa2 PE=1 SV=3
Wnk1	P83741-3	1	1	146709	165103	180702	152190	196911	228858	187550	222721	133964	180289	0.291949	0.534693	168323	190676.4	Up	0.179894	0.755265	Isoform 3 of Serine/threonine-protein kinase WNK1 OS=Mus musculus GN=Wnk1
Sptbn1	Q62261	68	68	100302.4	192179.9	177291.5	221756.6	174292.8	165590	195303.2	218383.2	223390.2	193380.5	0.29241	0.534008	173164.6	199209.4	Up	0.202142	0.755265	Spectrin beta chain, non-erythrocytic 1 OS=Mus musculus GN=Sptbn1 PE=1 SV=2
Jph3	Q9ET77	1	1	694968	143879	356252	180855	375772	322379	282390	241340	220498	78210	0.300942	0.521517	350345.2	228963.4	Down	-0.61366	0.755265	Junctophilin-3 OS=Mus musculus GN=Jph3 PE=1 SV=1
Prickle1	Q3U5C7	3	3	44654.5	34087.5	22027	28627	47764	23073	36818.5	33973.5	30230	21167	0.301016	0.52141	35432	29052.4	Down	-0.2864	0.755265	Prickle-like protein 1 OS=Mus musculus GN=Prickle1 PE=2 SV=1
Hexb	P20060	3	3	95958	116288.3	119701	141144.3	120748	119329	109107.7	150759.7	128050.3	147101.7	0.292721	0.533546	118767.9	130869.7	Up	0.139985	0.755265	Beta-hexosaminidase subunit beta OS=Mus musculus GN=Hexb PE=2 SV=2
Zfand1	Q8BFR6	1	1	19122	30784	31969	34705	24484	28585	28778	36839	30577	35075	0.29334	0.532629	28212.8	31970.8	Up	0.180405	0.755265	AN1-type zinc finger protein 1 OS=Mus musculus GN=Zfand1 PE=1 SV=1
Fen1	P39749	1	1	231879	192442	180801	169004	229464	191317	188978	191670	186461	167138	0.30242	0.519389	200718	185112.8	Down	-0.11677	0.755265	Flap endonuclease 1 OS=Mus musculus GN=Fen1 PE=1 SV=1
Ptk2b	Q9QVP9	3	3	824341	908228	762510	883103.5	650172.5	781185.5	896573.5	825226	849029	1031376	0.293632	0.532197	805671	876677.9	Up	0.121856	0.755265	Protein-tyrosine kinase 2-beta OS=Mus musculus GN=Ptk2b PE=1 SV=2
Ppih	Q9D868	1	1	755047	602995	556127	452772	483245	681256	533402	395437	443633	368064	0.302924	0.518666	570037.2	484358.4	Down	-0.23498	0.755265	Peptidyl-prolyl cis-trans isomerase H OS=Mus musculus GN=Ppih PE=2 SV=1
Purb	O35295	20	20	466807.6	286120.9	293211.4	302537.8	324497.8	336487.1	276578	301888.3	288528.4	264026.2	0.302991	0.51857	334635.1	293501.6	Down	-0.18922	0.755265	Transcriptional activator protein Pur-beta OS=Mus musculus GN=Purb PE=1 SV=3
Epb41l1	Q9Z2H5-3	7	7	210337.7	32747.57	59131.29	33154.29	87322.14	56259.86	43911.86	53903.14	41704.86	31283	0.303285	0.518149	84538.6	45412.54	Down	-0.89652	0.755265	Isoform 3 of Band 4.1-like protein 1 OS=Mus musculus GN=Epb41l1
Agfg1	Q8K2K6-2	4	4	118659	208836.3	154412.5	178664.8	196197	234514.3	177895	242445.8	159372.3	173779.5	0.294213	0.531338	171353.9	197601.4	Up	0.205614	0.755265	Isoform 2 of Arf-GAP domain and FG repeat-containing protein 1 OS=Mus musculus GN=Agfg1
Trmt112	Q9DCG9	2	2	387508.5	251805	282615.5	253776	322693.5	327165.5	243889.5	252410.5	235343	271230	0.303428	0.517944	299679.7	266007.7	Down	-0.17195	0.755265	tRNA methyltransferase 112 homolog OS=Mus musculus GN=Trmt112 PE=2 SV=1
Grm4	Q68EF4	1	1	17209	98684	73650	90033	62857	75346	79750	88156	81331	107073	0.294414	0.531042	68486.6	86331.2	Up	0.33406	0.755265	Metabotropic glutamate receptor 4 OS=Mus musculus GN=Grm4 PE=2 SV=2
Rpl24	Q8BP67	10	10	1003541	326202.4	367148.5	310498.7	512440.2	470186.3	342364.7	364127.4	321991.5	240105.9	0.303456	0.517904	503966.2	347755.2	Down	-0.53526	0.755265	60S ribosomal protein L24 OS=Mus musculus GN=Rpl24 PE=1 SV=2
Syt1	P46096	12	12	122967.1	112634.6	153840.8	152257	150428.7	134776.1	168488.5	171777.6	152298.8	131718.1	0.294553	0.530837	138425.6	151811.8	Up	0.133173	0.755265	Synaptotagmin-1 OS=Mus musculus GN=Syt1 PE=1 SV=1
Fez1	Q8K0X8	1	1	NA	10096	15397	40274	77127	24576	NA	16557	11595	14347	0.304814	0.515965	35723.5	16768.75	Down	-1.0911	0.755265	Fasciculation and elongation protein zeta-1 OS=Mus musculus GN=Fez1 PE=1 SV=2
Tcp1	P11983	3	3	169045.5	189353	175148.5	202757.5	160700	188192	173361	177590.5	198685	230680	0.294969	0.530224	179400.9	193701.7	Up	0.110649	0.755265	T-complex protein 1 subunit alpha OS=Mus musculus GN=Tcp1 PE=1 SV=3
Cntn4	Q69Z26-3	1	1	NA	53162	50921	52156	59050	39292	24436	67358	47602	48739	0.304985	0.515722	53822.25	45485.4	Down	-0.2428	0.755265	Isoform 3 of Contactin-4 OS=Mus musculus GN=Cntn4
Gmps	Q3THK7	5	5	29764	45391.2	64011	49051.2	52190.8	48244.2	59412	55341.8	60295.4	51831.2	0.295882	0.528881	48081.64	55024.92	Up	0.194599	0.755265	GMP synthase [glutamine-hydrolyzing] OS=Mus musculus GN=Gmps PE=1 SV=2
Psat1	Q99K85	5	5	91506	183872.8	178978	225515	107406	191326.5	165585.5	188680.3	224509.8	174274	0.296133	0.528513	157455.6	188875.2	Up	0.262489	0.755265	Phosphoserine aminotransferase OS=Mus musculus GN=Psat1 PE=1 SV=1
Atp12a	Q9Z1W8	4	4	1569799	1671232	2747909	2307274	2643772	2014089	2139492	2158195	1805340	1121896	0.306115	0.514115	2187997	1847802	Down	-0.2438	0.755265	Potassium-transporting ATPase alpha chain 2 OS=Mus musculus GN=Atp12a PE=1 SV=3
Idh2	P54071	4	4	466593.5	935992.5	855955	911149.5	806539	838181	884530.3	811825.8	910696.3	1088613	0.296511	0.527959	795245.9	906769.3	Up	0.189335	0.755265	Isocitrate dehydrogenase [NADP], mitochondrial OS=Mus musculus GN=Idh2 PE=1 SV=3
Mark2	Q05512-4	4	4	76882.75	47597.5	45726.75	49608.5	84068.5	53386.25	59614.25	57362.25	46417.25	35583.5	0.306339	0.513798	60776.8	50472.7	Down	-0.26802	0.755265	Isoform 4 of Serine/threonine-protein kinase MARK2 OS=Mus musculus GN=Mark2
Dixdc1	Q80Y83-11	1	1	250589	222583	158881	166658	201765	196724	185113	178635	172549	164548	0.30642	0.513683	200095.2	179513.8	Down	-0.15659	0.755265	Isoform 11 of Dixin OS=Mus musculus GN=Dixdc1
Stx1a	O35526	4	4	711	1504	1026.33	1586	1531	932.33	1942	1943.67	9398	1584.33	0.296534	0.527926	1271.666	3160.066	Up	1.313235	0.755265	Syntaxin-1A OS=Mus musculus GN=Stx1a PE=1 SV=3
Inpp4a	Q9EPW0	3	3	591948.3	600839.3	648541	604743.7	560101.7	650195.7	659385	591767.7	639963	582854.3	0.297317	0.52678	601234.8	624833.1	Up	0.055542	0.755265	Type I inositol 3,4-bisphosphate 4-phosphatase OS=Mus musculus GN=Inpp4a PE=2 SV=1
Hspa2	P17156	20	20	408250.2	679554.3	605892.7	645161.8	629077.1	658445.5	603712.8	610949.5	652393.7	753094.7	0.298254	0.525414	593587.2	655719.2	Up	0.143618	0.755265	Heat shock-related 70 kDa protein 2 OS=Mus musculus GN=Hspa2 PE=1 SV=2
Wnk2	Q3UH66-3	3	3	151326.3	104309.3	104561	95165	124890.7	118020.3	104522.3	109708.3	94920	90411.33	0.308301	0.511025	116050.5	103516.5	Down	-0.16489	0.756488	Isoform 3 of Serine/threonine-protein kinase WNK2 OS=Mus musculus GN=Wnk2
Hp1bp3	Q3TEA8-3	6	6	108429.5	144643.8	206684.5	187828	344848.3	54065.33	154269.5	187493.7	191661.2	143535.3	0.308404	0.51088	198486.8	146205	Down	-0.44105	0.756488	Isoform 3 of Heterochromatin protein 1-binding protein 3 OS=Mus musculus GN=Hp1bp3
Specc1l	Q2KN98	3	3	357437.3	196266.3	251253.3	205123.3	288334.3	255030	230822.7	237491.3	204029	192411.3	0.308706	0.510455	259682.9	223956.9	Down	-0.21353	0.756488	Cytospin-A OS=Mus musculus GN=Specc1l PE=1 SV=1
Hepacam	Q640R3	4	4	99870.25	499151.5	310398.3	373952	341793.3	378569.3	345541.5	451350	343553	523592.3	0.298508	0.525044	325033.1	408521.2	Up	0.329825	0.755265	Hepatocyte cell adhesion molecule OS=Mus musculus GN=Hepacam PE=1 SV=2
Hax1	O35387	2	2	181076.5	214944.5	242722.5	192930.5	152323.5	293997.5	192917.5	187924	242535.5	206187	0.298661	0.524821	196799.5	224712.3	Up	0.191353	0.755265	HCLS1-associated protein X-1 OS=Mus musculus GN=Hax1 PE=1 SV=1
Uqcrq	Q9CQ69	3	3	1064272	1219883	1413921	1810860	1222306	1338992	1400184	1159780	1118954	825336.3	0.310638	0.507745	1346249	1168649	Down	-0.2041	0.7597	Cytochrome b-c1 complex subunit 8 OS=Mus musculus GN=Uqcrq PE=1 SV=3
Wasf2	Q8BH43	4	4	72441.5	67214	44209.75	60610.25	47669.5	72370	55641.75	56649.25	62798.25	98827.75	0.298909	0.524461	58429	69257.4	Up	0.245284	0.755265	Wiskott-Aldrich syndrome protein family member 2 OS=Mus musculus GN=Wasf2 PE=1 SV=1
Fth1	P09528	2	2	678246	478298.5	404377.5	403856	530553.5	477773	447242.5	450218.5	422445	399181.5	0.311808	0.506113	499066.3	439372.1	Down	-0.18379	0.759952	Ferritin heavy chain OS=Mus musculus GN=Fth1 PE=1 SV=2
Aida	Q8C4Q6	2	2	636544	452441	363304	399257	467602	384611	460919	417985	420217	353177	0.312252	0.505495	463829.6	407381.8	Down	-0.18721	0.759952	Axin interactor, dorsalization-associated protein OS=Mus musculus GN=Aida PE=1 SV=1
Efnb2	P52800	1	1	308915	452435	465843	469744	429059	484196	436881	561019	394099	476492	0.298972	0.524369	425199.2	470537.4	Up	0.146171	0.755265	Ephrin-B2 OS=Mus musculus GN=Efnb2 PE=1 SV=1
Timm8a2	Q4FZG7	1	1	NA	125315	115649	125025	142760	46718	137502	126682	103794	124726	0.313211	0.504163	127187.3	107884.4	Down	-0.23747	0.759952	Putative mitochondrial import inner membrane translocase subunit Tim8 A-B OS=Mus musculus GN=Timm8a2 PE=3 SV=1
Camsap2	Q8C1B1	1	1	160304	200970	209558	204780	197639	189259	191052	246500	196447	232128	0.299812	0.523151	194650.2	211077.2	Up	0.116887	0.755265	Calmodulin-regulated spectrin-associated protein 2 OS=Mus musculus GN=Camsap2 PE=1 SV=3
Sh3gl1	Q62419	6	6	586172.6	312134.2	414286.2	401905.8	428045.8	460552.4	358060.6	413132.8	332902.6	287183.2	0.31473	0.502062	428508.9	370366.3	Down	-0.21037	0.759952	Endophilin-A2 OS=Mus musculus GN=Sh3gl1 PE=1 SV=1
Uqcrb	Q9D855	9	9	637672.6	1049341	928973.9	930056.6	863633.4	906232.1	838547	828959.3	678594.1	725402	0.315328	0.501237	881935.6	795546.9	Down	-0.14873	0.759952	Cytochrome b-c1 complex subunit 7 OS=Mus musculus GN=Uqcrb PE=1 SV=3
Ablim3	Q69ZX8	1	1	5155	9803	6669	8735	7147	9147	10167	8192	7452	7873	0.300069	0.522779	7501.8	8566.2	Up	0.191419	0.755265	Actin-binding LIM protein 3 OS=Mus musculus GN=Ablim3 PE=1 SV=2
Hapln4	Q80WM4	4	4	190227.7	191942.7	180347	181916.7	198265.3	210741	167205	196405.7	145838	145620.3	0.315589	0.500878	188539.9	173162	Down	-0.12275	0.759952	Hyaluronan and proteoglycan link protein 4 OS=Mus musculus GN=Hapln4 PE=2 SV=2
Syt2	P46097	2	2	118974	124939	223621.5	182665	190850	154152	193228.5	143491	118769	88672.5	0.316135	0.500127	168209.9	139662.6	Down	-0.26832	0.759952	Synaptotagmin-2 OS=Mus musculus GN=Syt2 PE=1 SV=1
Pcp4	P63054	2	2	763217	527074	658063	389623	737376	578918	566947	627841	417724	445190	0.316205	0.500031	615070.6	527324	Down	-0.22206	0.759952	Purkinje cell protein 4 OS=Mus musculus GN=Pcp4 PE=2 SV=2
Tardbp	Q921F2	1	1	122675	144526	190320	137573	125568	59578	111222	159724	148444	126621	0.31655	0.499558	144132.4	121117.8	Down	-0.25098	0.759952	TAR DNA-binding protein 43 OS=Mus musculus GN=Tardbp PE=1 SV=1
Rab7	P51150	2	2	145195.5	60200	127471.5	99573	108086.5	96286.5	94114.5	109898.5	92953	55774	0.317596	0.498125	108105.3	89805.3	Down	-0.26756	0.759952	Ras-related protein Rab-7a OS=Mus musculus GN=Rab7a PE=1 SV=2
Zc2hc1a	Q8BJH1	6	6	581373.7	437903.5	368220.5	390904.7	477993.8	464132.3	420323	415610.3	386819.5	334752.2	0.317609	0.498107	451279.2	404327.5	Down	-0.1585	0.759952	Zinc finger C2HC domain-containing protein 1A OS=Mus musculus GN=Zc2hc1a PE=2 SV=1
Atp1a4	Q9WV27	10	10	798489.4	849262.8	1398632	1184627	1347948	1034866	1097130	1100130	924454.9	574430.5	0.317903	0.497705	1115792	946202.3	Down	-0.23785	0.759952	Sodium/potassium-transporting ATPase subunit alpha-4 OS=Mus musculus GN=Atp1a4 PE=1 SV=3
Atpaf1	Q811I0	1	1	272795	79505	112622	53183	149995	127330	90595	73292	75161	79373	0.318085	0.497457	133620	89150.2	Down	-0.58383	0.759952	ATP synthase mitochondrial F1 complex assembly factor 1 OS=Mus musculus GN=Atpaf1 PE=2 SV=1
Vcpip1	Q8CDG3	2	2	206687	114238.5	135298	93792.5	148433.5	146086.5	121060.5	117287.5	109638.5	88023	0.318304	0.497158	139689.9	116419.2	Down	-0.2629	0.759952	Deubiquitinating protein VCIP135 OS=Mus musculus GN=Vcpip1 PE=2 SV=1
Mtss1l	Q6P9S0	4	4	14000.75	21805.75	23143.75	22745	17665.25	22839.25	24693.25	21562	22226.25	19220	0.30103	0.52139	19872.1	22108.15	Up	0.153834	0.755265	MTSS1-like protein OS=Mus musculus GN=Mtss1l PE=1 SV=1
Chordc1	Q9D1P4	1	1	74947	56296	87541	62862	80765	113100	89065	65208	92260	63604	0.301763	0.520334	72482.2	84647.4	Up	0.223839	0.755265	Cysteine and histidine-rich domain-containing protein 1 OS=Mus musculus GN=Chordc1 PE=1 SV=1
Usp8	Q80U87	2	2	248514.5	278358.5	226935.5	262465	262077.5	336976.5	266587.5	287587.5	235438.5	259740.5	0.302788	0.518861	255670.2	277266.1	Up	0.116987	0.755265	Ubiquitin carboxyl-terminal hydrolase 8 OS=Mus musculus GN=Usp8 PE=1 SV=2
Ranbp1	P34022	3	3	NA	1737.5	2250.5	11098.5	14617	NA	3808.5	2669	5414	2371	0.319139	0.49602	7425.875	3565.625	Down	-1.05841	0.759952	Ran-specific GTPase-activating protein OS=Mus musculus GN=Ranbp1 PE=1 SV=2
Mipep	A6H611	1	1	89509	206452	187592	152234	184516	24617	152476	136513	146444	179895	0.319147	0.496009	164060.6	127989	Down	-0.35821	0.759952	Mitochondrial intermediate peptidase OS=Mus musculus GN=Mipep PE=2 SV=1
Ppp2r5e	Q61151	1	1	192153	344272	334258	411826	261331	305183	356188	346720	350445	423004	0.303336	0.518076	308768	356308	Up	0.206602	0.755265	Serine/threonine-protein phosphatase 2A 56 kDa regulatory subunit epsilon isoform OS=Mus musculus GN=Ppp2r5e PE=2 SV=3
Kcnc3	Q63959	1	1	NA	36246	90245	74533	127031	39842	111097	45106	43789	43766	0.3194	0.495665	82013.75	56720	Down	-0.53201	0.759952	Potassium voltage-gated channel subfamily C member 3 OS=Mus musculus GN=Kcnc3 PE=1 SV=2
Mrpl47	Q8K2Y7	1	1	161083	181410	152062	173818	200735	163116	123362	172704	168258	174426	0.31961	0.49538	173821.6	160373.2	Down	-0.11617	0.759952	39S ribosomal protein L47, mitochondrial OS=Mus musculus GN=Mrpl47 PE=2 SV=2
Erc2	Q6PH08-4	4	4	277868.3	294964	268256.3	270715.5	252455.3	301133.3	252040.3	309529	276935.3	292805.3	0.303441	0.517926	272851.9	286488.6	Up	0.07036	0.755265	Isoform 4 of ERC protein 2 OS=Mus musculus GN=Erc2
Rtn4	Q99P72	18	18	30385.08	122031.1	84828.08	114927.8	102891.7	116025	92110.25	104921.9	105864	138797.8	0.303749	0.517485	91012.75	111543.8	Up	0.29347	0.755265	Reticulon-4 OS=Mus musculus GN=Rtn4 PE=1 SV=2
Prosc	Q9Z2Y8	3	3	590971.5	528493.5	470262.5	514933.5	491100	572826.5	470443.5	500912	455575.5	421630	0.319885	0.495006	519152.2	484277.5	Down	-0.10032	0.759952	Proline synthase co-transcribed bacterial homolog protein OS=Mus musculus GN=Prosc PE=1 SV=1
Snap25	P60879-2	15	15	229606.5	167544	181649.7	175068.4	189819	194246.9	173054.6	187440.3	166280.3	152197.8	0.320393	0.494317	188737.5	174644	Down	-0.11196	0.759952	Isoform 2 of Synaptosomal-associated protein 25 OS=Mus musculus GN=Snap25
Eogt	Q8BYW9	1	1	1992416	271219	725760	375653	919352	800740	483456	526144	398840	305038	0.320697	0.493905	856880	502843.6	Down	-0.76898	0.759952	EGF domain-specific O-linked N-acetylglucosamine transferase OS=Mus musculus GN=Eogt PE=1 SV=1
Plcb1	Q9Z1B3	10	10	558655.1	467170.3	535935.3	501130.9	602990.6	624283.3	485826.6	442552.7	473417.4	403344	0.320721	0.493873	533176.4	485884.8	Down	-0.134	0.759952	1-phosphatidylinositol 4,5-bisphosphate phosphodiesterase beta-1 OS=Mus musculus GN=Plcb1 PE=2 SV=2
Rpl15	Q9CZM2	1	1	30425	6882	12787	9314	16526	13984	10393	11519	10257	5697	0.320752	0.493831	15186.8	10370	Down	-0.5504	0.759952	60S ribosomal protein L15 OS=Mus musculus GN=Rpl15 PE=2 SV=4
Vps9d1	Q8C190	1	1	125013	216981	150658	167489	194186	196986	205251	189756	163337	198901	0.304972	0.51574	170865.4	190846.2	Up	0.15955	0.755265	VPS9 domain-containing protein 1 OS=Mus musculus GN=Vps9d1 PE=2 SV=1
Gmppa	Q922H4	1	1	51195	15665	38825	22251	14152	58138	31451	33635	32961	NA	0.305112	0.515541	28417.6	39046.25	Up	0.458399	0.755265	Mannose-1-phosphate guanyltransferase alpha OS=Mus musculus GN=Gmppa PE=2 SV=1
Gpx1	P11352	9	9	47457.22	69933.44	87194.78	65283.33	53823.67	79842.11	67764.56	75915.78	72513	69150.22	0.305198	0.515418	64738.49	73037.13	Up	0.174006	0.755265	Glutathione peroxidase 1 OS=Mus musculus GN=Gpx1 PE=1 SV=2
Shank2	Q80Z38-3	12	12	240921	159183.3	164782.6	144409.6	225670.8	204544.7	144238.2	177630.4	155266.8	128867.4	0.323468	0.490169	186993.5	162109.5	Down	-0.20602	0.762007	Isoform 3 of SH3 and multiple ankyrin repeat domains protein 2 OS=Mus musculus GN=Shank2
Ntm	Q99PJ0	39	39	32359.27	56045.19	44708.19	44257.58	52639.5	49800	49614.62	52729.19	50687.54	51285.77	0.306296	0.513859	46001.95	50823.42	Up	0.143799	0.755265	Neurotrimin OS=Mus musculus GN=Ntm PE=2 SV=2
Mrps11	Q9DCA2	2	2	582565	483358.5	440064.5	504653.5	437749.5	571985	447857	428026.5	352384	411122	0.323696	0.489863	489678.2	442274.9	Down	-0.14689	0.762007	28S ribosomal protein S11, mitochondrial OS=Mus musculus GN=Mrps11 PE=2 SV=2
Hspa1b	P17879	5	5	1156025	2018082	1779765	1890144	1846606	1945859	1752495	1799760	1918298	2234532	0.306467	0.513616	1738124	1930189	Up	0.151211	0.755265	Heat shock 70 kDa protein 1B OS=Mus musculus GN=Hspa1b PE=1 SV=3
Diras2	Q5PR73	1	1	755741	359099	345572	230410	355575	358219	214893	349401	335982	271858	0.324572	0.488689	409279.4	306070.6	Down	-0.41922	0.762007	GTP-binding protein Di-Ras2 OS=Mus musculus GN=Diras2 PE=2 SV=1
Cplx1	P63040	14	14	6015119	3688337	3178017	3431061	3420052	3975376	3306015	3442046	3128763	2845091	0.324633	0.488607	3946517	3339458	Down	-0.24097	0.762007	Complexin-1 OS=Mus musculus GN=Cplx1 PE=1 SV=1
Cplx2	P84086	14	14	4175753	2562573	2217562	2392834	2377464	2766734	2300669	2395567	2181888	1985919	0.325135	0.487936	2745237	2326155	Down	-0.23898	0.762007	Complexin-2 OS=Mus musculus GN=Cplx2 PE=1 SV=1
Sgip1	Q8VD37-4	17	17	219624.5	187355.2	174167.9	178623.5	188150.4	184602.5	183865.7	183591.9	179314.6	170757.5	0.325388	0.487598	189584.3	180426.4	Down	-0.07143	0.762007	Isoform 4 of SH3-containing GRB2-like protein 3-interacting protein 1 OS=Mus musculus GN=Sgip1
Hspa4	Q61316	12	12	265917.4	179163.6	227081.5	186800.6	255519.2	247951.1	198450.1	215855.3	183123.7	134624	0.325527	0.487413	222896.5	196000.8	Down	-0.18551	0.762007	Heat shock 70 kDa protein 4 OS=Mus musculus GN=Hspa4 PE=1 SV=1
Epg5	Q80TA9	2	2	103039.5	180968.5	179738.5	179905.5	216855.5	243790.5	188735	188644	191466.5	171115.5	0.307115	0.512699	172101.5	196750.3	Up	0.193106	0.755778	Ectopic P granules protein 5 homolog OS=Mus musculus GN=Epg5 PE=2 SV=2
Sub1	P11031	4	4	NA	36084.25	33578.5	36451.5	100911.5	20050.5	42531.75	24819.25	38574	35754.75	0.325952	0.486846	51756.44	32346.05	Down	-0.67815	0.762007	Activated RNA polymerase II transcriptional coactivator p15 OS=Mus musculus GN=Sub1 PE=1 SV=3
Ntrk2	P15209-2	4	4	168921.5	89413	114304.3	112370	178095.5	138212.3	109960.8	112376.3	108701.3	92179	0.325976	0.486814	132620.9	112285.9	Down	-0.24013	0.762007	Isoform GP95-TRKB of BDNF/NT-3 growth factors receptor OS=Mus musculus GN=Ntrk2
Hspa1l	P16627	7	7	971519.8	1687282	1488167	1579936	1543979	1626619	1465863	1504373	1603188	1866483	0.307265	0.512487	1454177	1613305	Up	0.149817	0.755778	Heat shock 70 kDa protein 1-like OS=Mus musculus GN=Hspa1l PE=2 SV=4
Tgfb1i1	Q62219-2	1	1	NA	7627	5152	5334	7046	3326	5921	5397	6157	6284	0.326498	0.486119	6289.75	5417	Down	-0.21551	0.762126	Isoform 2 of Transforming growth factor beta-1-induced transcript 1 protein OS=Mus musculus GN=Tgfb1i1
Sirpa	P97797	5	5	70536.4	99054.8	88926.2	81022	129857.4	127517	105572.8	101963.4	107658.8	91449.4	0.308735	0.510414	93879.36	106832.3	Up	0.186468	0.756488	Tyrosine-protein phosphatase non-receptor type substrate 1 OS=Mus musculus GN=Sirpa PE=1 SV=1
Cdc42bpa	Q3UU96	2	2	20654	25796.5	29412.5	29112	18832.5	35628	18556	22080.5	33014	40550.5	0.309242	0.509702	24761.5	29965.8	Up	0.275218	0.757008	Serine/threonine-protein kinase MRCK alpha OS=Mus musculus GN=Cdc42bpa PE=2 SV=2
Ralb	Q9JIW9	1	1	532069	293434	280443	315465	307480	309552	279369	260104	296496	318689	0.327807	0.484382	345778.2	292842	Down	-0.23972	0.762316	Ras-related protein Ral-B OS=Mus musculus GN=Ralb PE=2 SV=1
Paox	Q8C0L6	2	2	19316	41019	46195.5	38251	50397.5	10116.5	35240.5	42465.5	35116	32291	0.328282	0.483753	39035.8	31045.9	Down	-0.3304	0.762316	Peroxisomal N(1)-acetyl-spermine/spermidine oxidase OS=Mus musculus GN=Paox PE=1 SV=3
Aifm1	Q9Z0X1	9	9	48601	32949.11	38525.33	49197.33	39459.11	38019.22	44402.56	36691.78	34155.78	36541	0.328659	0.483254	41746.38	37962.07	Down	-0.13709	0.762316	Apoptosis-inducing factor 1, mitochondrial OS=Mus musculus GN=Aifm1 PE=1 SV=1
Homer1	Q9Z2Y3-5	2	2	22648.5	20084.5	40374	34901.5	38010	23172	45540.5	66628	44655.5	25330.5	0.311613	0.506384	31203.7	41065.3	Up	0.396203	0.759952	Isoform 5 of Homer protein homolog 1 OS=Mus musculus GN=Homer1

Plppr2	Q8VCY8-2	1	1	209660	175636	187195	161265	168871	206855	166578	253761	201127	171653	0.313082	0.504342	180525.4	199994.8	Up	0.147761	0.759952	Isoform 2 of Lipid phosphate phosphatase-related protein type 2 OS=Mus musculus GN=Lppr2
Mrpl23	O35972	3	3	45947	51595.33	50825.33	59669	52097.67	62081.67	56409.67	52879.67	48113.33	57985.33	0.314656	0.502164	52026.87	55493.93	Up	0.093073	0.759952	39S ribosomal protein L23, mitochondrial OS=Mus musculus GN=Mrpl23 PE=2 SV=1
Cd47	Q61735	1	1	17551	58674	55566	72492	49386	54072	69861	57082	54558	73177	0.315378	0.501169	50733.8	61750	Up	0.283492	0.759952	Leukocyte surface antigen CD47 OS=Mus musculus GN=Cd47 PE=1 SV=2
Phpt1	Q9DAK9	1	1	2981843	2199625	2798310	2187286	2686705	3443081	2766399	3111274	2607198	2292917	0.318759	0.496538	2570754	2844174	Up	0.145818	0.759952	14 kDa phosphohistidine phosphatase OS=Mus musculus GN=Phpt1 PE=2 SV=1
Fau	P62862	2	2	480141	277746.5	243993.5	282872	307031.5	279974.5	265946.5	308746	286802	209666.5	0.330365	0.481006	318356.9	270227.1	Down	-0.23647	0.762316	40S ribosomal protein S30 OS=Mus musculus GN=Fau PE=1 SV=1
Cpne6	Q9Z140	8	8	532271.2	358079.8	343180.2	328507.5	447604.2	498231.8	304359.2	359899.5	317102	219508	0.330377	0.48099	401928.6	339820.1	Down	-0.24217	0.762316	Copine-6 OS=Mus musculus GN=Cpne6 PE=2 SV=1
Nap1l4	Q78ZA7	10	10	938654.3	644797.9	567653	530041	587371.3	666347.6	536142.7	551995.1	534299.9	560511.9	0.330483	0.480851	653703.5	569859.4	Down	-0.19803	0.762316	Nucleosome assembly protein 1-like 4 OS=Mus musculus GN=Nap1l4 PE=1 SV=1
Tpm3	P21107	4	4	155499.7	171546.3	143592.3	147852	151899	170248.7	161709	164828	146574.7	160089.3	0.318781	0.496508	154077.9	160689.9	Up	0.06062	0.759952	Tropomyosin alpha-3 chain OS=Mus musculus GN=Tpm3 PE=1 SV=3
Plxnd1	Q3UH93	6	6	13112.5	42702.17	36702.67	41072.83	48741.33	45725.83	43513.83	49182	40624.33	39005.5	0.318931	0.496303	36466.3	43610.3	Up	0.258105	0.759952	Plexin-D1 OS=Mus musculus GN=Plxnd1 PE=1 SV=1
Prkar1b	P12849	2	2	NA	58185	46918	66128	53064.5	26253.5	68586.5	49970	34174	55966	0.331932	0.478951	56073.88	46990	Down	-0.25498	0.762316	cAMP-dependent protein kinase type I-beta regulatory subunit OS=Mus musculus GN=Prkar1b PE=2 SV=2
Ak5	Q920P5	2	2	63699.5	90902.5	82835	101147.5	106848	92438	109611	99443	118224	79516	0.319257	0.49586	89086.5	99846.4	Up	0.164504	0.759952	Adenylate kinase isoenzyme 5 OS=Mus musculus GN=Ak5 PE=2 SV=2
Cpsf6	Q6NVF9	2	2	NA	8218	5147	5896	12833	NA	NA	7209	4926	5766	0.332373	0.478374	8023.5	5967	Down	-0.42723	0.762316	Cleavage and polyadenylation specificity factor subunit 6 OS=Mus musculus GN=Cpsf6 PE=2 SV=1
2-Sep	P42208	5	5	100285.2	58657.4	103963.4	73897	106542	87642.8	82479.2	74047.2	84417.2	57284.8	0.332412	0.478323	88669	77174.24	Down	-0.20031	0.762316	Septin-2 OS=Mus musculus GN=Sept2 PE=1 SV=2
R3hdm2	Q80TM6-3	1	1	202632	89957	92650	151857	266609	168753	194315	104859	65435	38806	0.332528	0.478172	160741	114433.6	Down	-0.49023	0.762316	Isoform 3 of R3H domain-containing protein 2 OS=Mus musculus GN=R3hdm2
Slc25a12	Q8BH59	10	10	774623.9	1128001	1200781	1273311	1083113	1167089	979819.9	966971.7	928301	906117.2	0.332948	0.477624	1091966	989659.8	Down	-0.14192	0.762596	Calcium-binding mitochondrial carrier protein Aralar1 OS=Mus musculus GN=Slc25a12 PE=1 SV=1
G3bp2	P97379-2	3	3	58325.33	101859.7	145410.7	103809.3	101873	99191	129887.7	133717	125662.7	106385.7	0.319672	0.495295	102255.6	118968.8	Up	0.218403	0.759952	Isoform B of Ras GTPase-activating protein-binding protein 2 OS=Mus musculus GN=G3bp2
Calm1	P62204	14	14	34531.6	69234.4	46629	69023.6	48432.6	36310.8	79367.8	76093.4	66906.6	63275.4	0.319876	0.495018	53570.24	64390.8	Up	0.265423	0.759952	Calmodulin OS=Mus musculus GN=Calm1 PE=1 SV=2
Shank2	Q80Z38	13	13	231045.4	157264.2	162370	143044.7	216927.7	195376.3	141700.5	178519.5	153927.3	132100.4	0.335359	0.47449	182130.4	160324.8	Down	-0.18397	0.766066	SH3 and multiple ankyrin repeat domains protein 2 OS=Mus musculus GN=Shank2 PE=1 SV=2
Dpy30	Q99LT0	2	2	21916	61621.5	54829	57440.5	43601.5	57191	54241	56830.5	54650.5	57014	0.320823	0.493735	47881.7	55985.4	Up	0.225576	0.759952	Protein dpy-30 homolog OS=Mus musculus GN=Dpy30 PE=1 SV=1
Slc39a10	Q6P5F6	1	1	78728	123504	119240	126260	78080	147360	110264	138931	98841	107124	0.322656	0.49126	105162.4	120504	Up	0.196462	0.762007	Zinc transporter ZIP10 OS=Mus musculus GN=Slc39a10 PE=1 SV=1
Kcnb1	Q03717	2	2	281216	437660	373362	339355	465602.5	520550	411027.5	464524	397838.5	341025.5	0.323164	0.490577	379439.1	426993.1	Up	0.170344	0.762007	Potassium voltage-gated channel subfamily B member 1 OS=Mus musculus GN=Kcnb1 PE=1 SV=2
Sgip1	Q8VD37	20	20	201234	179201.6	162104.5	168473.8	174887.4	176848.3	172869.7	169675.7	167002.8	161691.4	0.337363	0.471903	177180.3	169617.6	Down	-0.06293	0.767906	SH3-containing GRB2-like protein 3-interacting protein 1 OS=Mus musculus GN=Sgip1 PE=1 SV=1
Cck	P09240	1	1	261810	144847	171588	132436	166457	251717	205358	172466	233557	163984	0.323532	0.490083	175427.6	205416.4	Up	0.227676	0.762007	Cholecystokinin OS=Mus musculus GN=Cck PE=1 SV=1
Pdlim5	Q8CI51-3	1	1	31171	47423	59331	42163	80494	74692	56951	55370	65089	58046	0.32442	0.488892	52116.4	62029.6	Up	0.251219	0.762007	Isoform 3 of PDZ and LIM domain protein 5 OS=Mus musculus GN=Pdlim5
Cox4i1	P19783	4	4	92092.5	40147.5	100758	112538.8	120972.5	79458.25	96199	84761.75	67033.25	57137.75	0.339295	0.469423	93301.85	76918	Down	-0.27858	0.769888	Cytochrome c oxidase subunit 4 isoform 1, mitochondrial OS=Mus musculus GN=Cox4i1 PE=1 SV=2
Sh2d5	Q8JZW5	1	1	NA	87111	69754	194962	219764	150994	164689	257453	158816	218116	0.325543	0.487392	142897.8	190013.6	Up	0.411119	0.762007	SH2 domain-containing protein 5 OS=Mus musculus GN=Sh2d5 PE=2 SV=1
Atad3a	Q925I1	4	4	158429.3	115398.7	161097.3	140328	149123.3	140036	148661.7	138004	131893	114692.3	0.340093	0.468402	144875.3	134657.4	Down	-0.10552	0.770242	ATPase family AAA domain-containing protein 3 OS=Mus musculus GN=Atad3 PE=1 SV=1
Pgrmc1	O55022	9	9	383134.1	663968.4	695698.1	613779.1	844123.9	704752.1	755667.4	829937.8	677236.3	669237.9	0.32615	0.486583	640140.7	727366.3	Up	0.184293	0.762007	Membrane-associated progesterone receptor component 1 OS=Mus musculus GN=Pgrmc1 PE=1 SV=4
Tubgcp2	Q921G8	3	3	26491.33	36030.33	36815.67	35173.33	40358.33	36749.67	42773.33	34412	37502	37585.33	0.327314	0.485035	34973.8	37804.47	Up	0.112282	0.762316	Gamma-tubulin complex component 2 OS=Mus musculus GN=Tubgcp2 PE=2 SV=2
Map1b	P14873	37	37	143678.5	92257.74	77890.71	85119.68	118344.1	101428.1	90446.32	92561.52	86801.19	78831.13	0.340884	0.467393	103458.1	90013.66	Down	-0.20083	0.770242	Microtubule-associated protein 1B OS=Mus musculus GN=Map1b PE=1 SV=2
Hnrnpd	Q60668-3	5	5	221468	504365.3	424849.5	397138	608813.5	325153	496180.8	538076.5	574210	669141.5	0.327518	0.484765	431326.9	520552.4	Up	0.271262	0.762316	Isoform 3 of Heterogeneous nuclear ribonucleoprotein D0 OS=Mus musculus GN=Hnrnpd
Cndp2	Q9D1A2	8	8	390990.9	234107	233792	228550.8	244169.4	236943.5	226139.3	255289.8	227861.9	214837.3	0.341643	0.466427	266322	232214.3	Down	-0.19771	0.770242	Cytosolic non-specific dipeptidase OS=Mus musculus GN=Cndp2 PE=1 SV=1
Ppp1r14b	Q62084	1	1	NA	9680	3376	5327	3119	4180	7772	8728	8170	7369	0.329287	0.482425	5375.5	7243.8	Up	0.430348	0.762316	Protein phosphatase 1 regulatory subunit 14B OS=Mus musculus GN=Ppp1r14b PE=1 SV=2
Anapc16	Q9CPV2	1	1	18025	13899	7566	8599	14669	11700	10380	11094	9091	9808	0.34252	0.465314	12551.6	10414.6	Down	-0.26926	0.770242	Anaphase-promoting complex subunit 16 OS=Mus musculus GN=Anapc16 PE=2 SV=1
Frrs1l	B1AXV0	1	1	508777	383549	423150	428060	471570	444947	414938	401800	468713	307073	0.342591	0.465224	443021.2	407494.2	Down	-0.1206	0.770242	DOMON domain-containing protein FRRS1L OS=Mus musculus GN=Frrs1l PE=1 SV=1
Ric8a	Q3TIR3	1	1	NA	80305	108788	124426	81804	106727	130199	109101	105767	107073	0.329467	0.482188	98830.75	111773.4	Up	0.177545	0.762316	Synembryn-A OS=Mus musculus GN=Ric8a PE=1 SV=2
Zc3h4	Q6ZPZ3	1	1	45619	87219	101913	83750	107080	70861	91591	96610	113969	128332	0.329645	0.481954	85116.2	100272.6	Up	0.236422	0.762316	Zinc finger CCCH domain-containing protein 4 OS=Mus musculus GN=Zc3h4 PE=1 SV=2
Gdi2	Q61598	15	15	251523.9	238308.6	254244.3	239810.7	215300.1	239603.4	248288	259099.7	270259.4	232134.2	0.329752	0.481813	239837.5	249876.9	Up	0.05916	0.762316	Rab GDP dissociation inhibitor beta OS=Mus musculus GN=Gdi2 PE=1 SV=1
Stx6	Q9JKK1	1	1	NA	46301	65747	51707	84949	NA	60058	61595	45528	38658	0.343792	0.463704	62176	51459.75	Down	-0.27291	0.770733	Syntaxin-6 OS=Mus musculus GN=Stx6 PE=1 SV=1
Ddx1	Q91VR5	2	2	149031.5	141884.5	122807	131681	136327.5	151262	138363.5	153605	157155.5	122171.5	0.330294	0.481099	136346.3	144515.1	Up	0.083909	0.762316	ATP-dependent RNA helicase DDX1 OS=Mus musculus GN=Ddx1 PE=1 SV=1
H2afy2	Q8CCK0	1	1	NA	NA	47975	43118	NA	NA	42060	NA	99564	59790	0.331393	0.479657	45546.5	67138	Up	0.559789	0.762316	Core histone macro-H2A.2 OS=Mus musculus GN=H2afy2 PE=1 SV=3
Farp1	F8VPU2	1	1	318133	201903	247936	240378	218308	213629	230036	188152	259205	218647	0.346731	0.460007	245331.6	221933.8	Down	-0.1446	0.772871	FERM, RhoGEF and pleckstrin domain-containing protein 1 OS=Mus musculus GN=Farp1 PE=1 SV=1
Astn2	Q80Z10-2	1	1	34965	72562	72092	97023	85680	92080	88767	78548	76092	85769	0.331409	0.479636	72464.4	84251.2	Up	0.217425	0.762316	Isoform 2 of Astrotactin-2 OS=Mus musculus GN=Astn2
Dnajc19	Q9CQV7-3	2	2	515102	171446	210016	247618	342397.5	302651	243036.5	199244.5	245034.5	155387.5	0.347471	0.459081	297315.9	229070.8	Down	-0.3762	0.772871	Isoform 3 of Mitochondrial import inner membrane translocase subunit TIM14 OS=Mus musculus GN=Dnajc19
Gabarapl2	P60521	1	1	162303	314300	192645	245225	253921	279686	231508	257482	274375	274587	0.331998	0.478865	233678.8	263527.6	Up	0.173427	0.762316	Gamma-aminobutyric acid receptor-associated protein-like 2 OS=Mus musculus GN=Gabarapl2 PE=1 SV=1
Dpp3	Q99KK7	1	1	NA	11993	4748	8075	5693	NA	NA	NA	20766	10735	0.333562	0.476823	7627.25	15750.5	Up	1.046163	0.763322	Dipeptidyl peptidase 3 OS=Mus musculus GN=Dpp3 PE=2 SV=2
Mut	P16332	2	2	40983	55406.5	49915	58279.5	40754.5	58985	52772	51991.5	48542.5	54056.5	0.334522	0.475575	49067.7	53269.5	Up	0.118536	0.764836	Methylmalonyl-CoA mutase, mitochondrial OS=Mus musculus GN=Mut PE=1 SV=2
Aldoa	P05064	100	100	535191.1	288639.8	361771.6	290506.8	442630.8	397010.2	338957.4	340210.7	314035.7	262526.4	0.348336	0.458002	383748	330548.1	Down	-0.2153	0.772871	Fructose-bisphosphate aldolase A OS=Mus musculus GN=Aldoa PE=1 SV=2
Dlg2	Q91XM9-3	12	12	238580.1	207781.5	207830.4	204768.6	230329.2	251267	190527.4	211178.5	185703.9	171960.6	0.348353	0.45798	217858	202127.5	Down	-0.10812	0.772871	Isoform 3 of Disks large homolog 2 OS=Mus musculus GN=Dlg2
Rpl7	P14148	4	4	384235.5	305062.8	236036.8	281550.3	310340.5	402779.3	298509.3	329476.3	358694.3	290243.8	0.336209	0.473391	303445.2	335940.6	Up	0.14677	0.767323	60S ribosomal protein L7 OS=Mus musculus GN=Rpl7 PE=1 SV=2
D630045J1	Q6ZPD9	7	7	40485.57	96490.14	72873.29	69046.43	98396.71	86441.57	85983	91761.86	92948.29	78705.14	0.3366	0.472886	75458.43	87167.97	Up	0.208116	0.767533	UPF0606 protein KIAA1549 OS=Mus musculus GN=Kiaa1549 PE=1 SV=3
Mark2	Q05512	5	5	66076.2	42440.6	45373.8	44555	76262	48208.2	55398.6	53102	43229.8	35694.6	0.3492	0.456926	54941.52	47126.64	Down	-0.22135	0.772871	Serine/threonine-protein kinase MARK2 OS=Mus musculus GN=Mark2 PE=1 SV=3
Limch1	Q3UH68	3	3	NA	18953.67	33513.67	37018	41747.67	46049.33	40649	35366.33	43143	29272	0.337211	0.472098	32808.25	38895.93	Up	0.245561	0.767906	LIM and calponin homology domains-containing protein 1 OS=Mus musculus GN=Limch1 PE=1 SV=2
Mcat	Q8R3F5	1	1	87494	282668	259108	330190	262458	297464	298559	287346	280142	282864	0.338208	0.470816	244383.6	289275	Up	0.243294	0.768492	Malonyl-CoA-acyl carrier protein transacylase, mitochondrial OS=Mus musculus GN=Mcat PE=1 SV=3
Fahd1	Q8R0F8	4	4	78200.25	73430.5	65517.75	70654.75	84150	76543.5	68686	75737	70649.25	56513.25	0.351153	0.454504	74390.65	69625.8	Down	-0.0955	0.772871	Acylpyruvase FAHD1, mitochondrial OS=Mus musculus GN=Fahd1 PE=1 SV=2
2310022B0	Q8C3W1	4	4	92721.5	37513.5	61060	24798.5	77385	46385	26004.5	71979.5	49352	24008	0.352727	0.452561	58695.7	43545.8	Down	-0.43072	0.772871	Uncharacterized protein C1orf198 homolog OS=Mus musculus PE=2 SV=1
Tomm22	Q9CPQ3	2	2	29882.5	65209.5	74609.5	57878.5	78421.5	107344.5	75875	60221.5	76033	51832.5	0.33822	0.470801	61200.3	74261.3	Up	0.279072	0.768492	Mitochondrial import receptor subunit TOM22 homolog OS=Mus musculus GN=Tomm22 PE=2 SV=3
Kcna3	P16390	1	1	518902	679535	786171	991410	670188	400396	751438	619106	777160	589214	0.353553	0.451545	729241.2	627462.8	Down	-0.21687	0.772871	Potassium voltage-gated channel subfamily A member 3 OS=Mus musculus GN=Kcna3 PE=2 SV=3
Stip1	Q60864	13	13	547453.9	409987.1	412852	373651.7	393346.4	411149.8	392602.3	397854.5	396767.3	376097.8	0.353671	0.451401	427458.2	394894.3	Down	-0.11432	0.772871	Stress-induced-phosphoprotein 1 OS=Mus musculus GN=Stip1 PE=1 SV=1
Chat	Q03059	1	1	52056	96128	106278	94136	112467	169221	78343	96151	102336	110755	0.339435	0.469243	92213	111361.2	Up	0.272205	0.769888	Choline O-acetyltransferase OS=Mus musculus GN=Chat PE=2 SV=2
Sprn	Q8BWU1	2	2	11664.5	13483.5	12063	12073.5	NA	9758	13776	15481.5	17512.5	12390.5	0.340682	0.467651	12321.13	13783.7	Up	0.161829	0.770242	Shadow of prion protein OS=Mus musculus GN=Sprn PE=1 SV=1
Cltc	Q68FD5	17	17	174580.5	158980.5	263785.9	324670.3	281423.7	264527.7	309406.5	249871.5	290031.5	266650.5	0.340771	0.467537	240688.2	276097.5	Up	0.198013	0.770242	Clathrin heavy chain 1 OS=Mus musculus GN=Cltc PE=1 SV=3
Herpud2	Q9JJC9	1	1	130809	154436	178548	172168	225430	186091	224987	218645	170052	157124	0.341309	0.466852	172278.2	192779.8	Up	0.162214	0.770242	Homocysteine-responsive endoplasmic reticulum-resident ubiquitin-like domain member 2 protein OS=Mus musculus GN=Herpud2 PE=2 SV=1
Hbb-y	P02104	3	3	6749362	5351038	4111081	3931428	3586320	5526792	4956941	3847894	5976207	7913033	0.341745	0.466298	4745846	5644173	Up	0.250097	0.770242	Hemoglobin subunit epsilon-Y2 OS=Mus musculus GN=Hbb-y PE=1 SV=2
Calu	O35887	3	3	113376.7	60829.67	57555	62959	76050.33	65892	60783.33	75163.67	60738	52360.67	0.354465	0.450427	74154.13	62987.53	Down	-0.23546	0.772871	Calumenin OS=Mus musculus GN=Calu PE=1 SV=1
Myo9a	Q8C170	1	1	463672	242137	298238	253287	308532	232737	284860	262540	335056	228946	0.355781	0.448817	313173.2	268827.8	Down	-0.22028	0.772871	Unconventional myosin-IXa OS=Mus musculus GN=Myo9a PE=2 SV=2
Cbr4	Q91VT4	1	1	NA	210379	214425	195258	228905	228657	188025	219153	165074	194131	0.356123	0.4484	212241.8	199008	Down	-0.09288	0.772871	Carbonyl reductase family member 4 OS=Mus musculus GN=Cbr4 PE=1 SV=2
Gigyf2	Q6Y7W8-2	1	1	248374	229502	158312	190800	249042	311324	242239	237536	240202	181812	0.342597	0.465216	215206	242622.6	Up	0.172996	0.770242	Isoform 2 of PERQ amino acid-rich with GYF domain-containing protein 2 OS=Mus musculus GN=Gigyf2
Rpl18	P35980	3	3	181985.7	93256.67	109041.7	88582	166743.3	135168.3	121412.3	114002.3	87719	68075.67	0.356426	0.448031	127921.9	105275.5	Down	-0.28109	0.772871	60S ribosomal protein L18 OS=Mus musculus GN=Rpl18 PE=2 SV=3
Myh10	Q61879	20	20	23230.89	55860.44	54462.94	57279.61	51930.28	52596.39	55099.89	61839.28	53749	54382.17	0.343203	0.464449	48552.83	55533.35	Up	0.193799	0.770733	Myosin-10 OS=Mus musculus GN=Myh10 PE=1 SV=2
Slmap	Q3URD3-5	2	2	287618.5	299268	261869	251579.5	292399.5	327471	289986	302205.5	266753.5	277007	0.34351	0.464061	278546.9	292684.6	Up	0.071427	0.770733	Isoform 5 of Sarcolemmal membrane-associated protein OS=Mus musculus GN=Slmap
Nit1	Q8VDK1	2	2	154665	143548.5	144459	130155	107254.5	106207.5	154828.5	107736	136111	114458	0.358769	0.445185	136016.4	123868.2	Down	-0.13497	0.772871	Nitrilase homolog 1 OS=Mus musculus GN=Nit1 PE=2 SV=2
Plxna1	P70206	5	5	NA	86058.25	81401	69125	107705.8	78683.75	77701	87773.75	69642.5	71674.75	0.358905	0.44502	86072.5	77095.15	Down	-0.15891	0.772871	Plexin-A1 OS=Mus musculus GN=Plxna1 PE=1 SV=1
Ccdc6	D3YZP9	3	3	87955.33	52478	61262	47842.67	63358	62146	53762.33	61842.33	49582.33	47648.33	0.359197	0.444667	62579.2	54996.26	Down	-0.18635	0.772871	Coiled-coil domain-containing protein 6 OS=Mus musculus GN=Ccdc6 PE=1 SV=1
Armc6	Q8BNU0	2	2	244121.5	328517.5	320733	349785.5	262793	338875.5	317358	329754	334259.5	298225	0.344018	0.463419	301190.1	323694.4	Up	0.103958	0.770733	Armadillo repeat-containing protein 6 OS=Mus musculus GN=Armc6 PE=2 SV=1
Aldh6a1	Q9EQ20	8	8	216990.4	223817.3	243955.4	315716.6	306220.6	232304.3	222208.8	250848	210763.3	273814.9	0.359556	0.444233	261340.1	237987.8	Down	-0.13504	0.772871	Methylmalonate-semialdehyde dehydrogenase [acylating], mitochondrial OS=Mus musculus GN=Aldh6a1 PE=1 SV=1
Slc25a42	Q8R0Y8	1	1	18726	7075	7318	17543	10112	7167	7827	6263	7078	16883	0.359609	0.444169	12154.8	9043.6	Down	-0.42656	0.772871	Mitochondrial coenzyme A transporter SLC25A42 OS=Mus musculus GN=Slc25a42 PE=2 SV=1
Ahcyl1	Q80SW1	9	9	363958.6	177703.6	304778.7	270431.6	284477	210945	297747.9	274865.3	248889.6	192744.7	0.360229	0.443421	280269.9	245038.5	Down	-0.19381	0.772871	Putative adenosylhomocysteinase 2 OS=Mus musculus GN=Ahcyl1 PE=1 SV=1
Stk39	Q9Z1W9	4	4	18614.5	24258	40147	29589.5	37569.5	54193	28927	43228	25909	31270	0.345562	0.461474	30035.7	36705.4	Up	0.289314	0.772871	STE20/SPS1-related proline-alanine-rich protein kinase OS=Mus musculus GN=Stk39 PE=1 SV=1
Slc23a2	Q9EPR4	2	2	NA	51174.5	63627.5	70064.5	53379	78651	51145.5	64384.5	84340.5	57896.5	0.347328	0.45926	59561.38	67283.6	Up	0.175878	0.772871	Solute carrier family 23 member 2 OS=Mus musculus GN=Slc23a2 PE=1 SV=2
Phactr1	Q2M3X8-3	2	2	89248.5	89498.5	69634.5	74777.5	61708	102652	73850.5	68411	79218	111337.5	0.347568	0.45896	76973.4	87093.8	Up	0.17821	0.772871	Isoform 3 of Phosphatase and actin regulator 1 OS=Mus musculus GN=Phactr1
Snx3	O70492	6	6	2331486	691493.6	1248787	764016.6	1316547	1204434	1113386	1075953	844702.4	527915	0.360932	0.442575	1270466	953278.1	Down	-0.41439	0.772871	Sorting nexin-3 OS=Mus musculus GN=Snx3 PE=1 SV=3
Pak3	Q61036	3	3	543775.3	403059	369549.7	369201.3	490345.3	488915.3	355092.3	419435	336338	358293.3	0.361285	0.44215	435186.1	391614.8	Down	-0.1522	0.772871	Serine/threonine-protein kinase PAK 3 OS=Mus musculus GN=Pak3 PE=1 SV=2
Mtdh	Q80WJ7	4	4	33306.75	43160	25172	32254.75	43961.5	46529.5	39046	43117.75	35075	35257	0.347772	0.458705	35571	39805.05	Up	0.16225	0.772871	Protein LYRIC OS=Mus musculus GN=Mtdh PE=1 SV=1
Mapk8ip3	Q9ESN9-3	2	2	161850.5	20786.5	64391.5	39926	79072.5	53005.5	51905	54009	51910	29156	0.362617	0.440552	73205.4	47997.1	Down	-0.609	0.772871	Isoform 1b of C-Jun-amino-terminal kinase-interacting protein 3 OS=Mus musculus GN=Mapk8ip3
4833439L1	Q9DBN4	1	1	465116	301897	359435	240436	296039	376726	259884	302945	247046	256449	0.362806	0.440326	332584.6	288610	Down	-0.2046	0.772871	Putative monooxygenase p33MONOX OS=Mus musculus GN=P33monox PE=1 SV=1
Fkbp1a	P26883	3	3	108700.7	77751.67	56622.33	59394.33	89437.67	60550.67	86007.67	67202.33	66730	57904.33	0.362899	0.440214	78381.33	67679	Down	-0.2118	0.772871	Peptidyl-prolyl cis-trans isomerase FKBP1A OS=Mus musculus GN=Fkbp1a PE=1 SV=2
Rps10	P63325	3	3	27563	59075	69105	77277.33	41604.67	44572.67	82512.67	71560	66370	64772.33	0.347931	0.458507	54925	65957.53	Up	0.264074	0.772871	40S ribosomal protein S10 OS=Mus musculus GN=Rps10 PE=1 SV=1
Cables1	Q9ESJ1	1	1	230651	146791	100305	110327	149196	154836	107410	120571	119528	113066	0.363364	0.439658	147454	123082.2	Down	-0.26064	0.772871	CDK5 and ABL1 enzyme substrate 1 OS=Mus musculus GN=Cables1 PE=1 SV=2
Asphd2	Q80VP9	1	1	NA	132950	84356	110420	105092	129509	128582	120720	119598	101182	0.348379	0.457948	108204.5	119918.2	Up	0.14829	0.772871	Aspartate beta-hydroxylase domain-containing protein 2 OS=Mus musculus GN=Asphd2 PE=2 SV=1
Cpne5	Q8JZW4	3	3	57804.33	71805	68262.33	54552.67	70624.67	57799.67	61527.33	64592.67	52796.67	65712.33	0.363685	0.439275	64609.8	60485.73	Down	-0.09516	0.772871	Copine-5 OS=Mus musculus GN=Cpne5 PE=2 SV=1
Sh2b1	Q91ZM2-3	1	1	55588	51631	36663	40334	59901	51763	53739	54835	62812	46979	0.348428	0.457887	48823.4	54025.6	Up	0.14607	0.772871	Isoform 3 of SH2B adapter protein 1 OS=Mus musculus GN=Sh2b1
Cep170	Q6A065	4	4	133412.3	136240	114973	112252.8	117766.5	126215.8	111395.5	85917.5	112304.5	132746.5	0.364178	0.438686	122928.9	113716	Down	-0.11239	0.772871	Centrosomal protein of 170 kDa OS=Mus musculus GN=Cep170 PE=1 SV=2
Pcca	Q91ZA3	2	2	287920	370180.5	314658.5	356921.5	389166	408163	403829.5	310279.5	335696.5	396569.5	0.350045	0.455876	343769.3	370907.6	Up	0.109619	0.772871	Propionyl-CoA carboxylase alpha chain, mitochondrial OS=Mus musculus GN=Pcca PE=1 SV=2
Fkbp4	P30416	2	2	289837	348607	388075	354660	284323.5	316064.5	317261	390035	396918	377826.5	0.350982	0.454715	333100.5	359621	Up	0.11052	0.772871	Peptidyl-prolyl cis-trans isomerase FKBP4 OS=Mus musculus GN=Fkbp4 PE=1 SV=5
Ndrg2	Q9QYG0	3	3	399737.7	319144.3	264234	295530	254266.7	288365.7	249799	247866.7	287958	316281.3	0.364525	0.438273	306582.5	278054.1	Down	-0.14091	0.772871	Protein NDRG2 OS=Mus musculus GN=Ndrg2 PE=1 SV=1
Mrpl53	Q9D1H8	1	1	296143	360808	419970	348279	479003	356038	369151	299251	279713	409859	0.364659	0.438113	380840.6	342802.4	Down	-0.15181	0.772871	39S ribosomal protein L53, mitochondrial OS=Mus musculus GN=Mrpl53 PE=2 SV=1
Cntn2	Q61330	5	5	109517.4	93099.2	118809.4	116505.6	94079.6	98582.8	113137.6	106699.8	135369.8	119000.4	0.353145	0.452047	106402.2	114558.1	Up	0.106551	0.772871	Contactin-2 OS=Mus musculus GN=Cntn2 PE=1 SV=2
Tpd52l1	O54818	1	1	534814	287131	263277	269578	324917	412275	238689	291163	218201	216050	0.36493	0.43779	335943.4	275275.6	Down	-0.28734	0.772871	Tumor protein D53 OS=Mus musculus GN=Tpd52l1 PE=2 SV=1
Rpl4	Q9D8E6	12	12	473863.1	329288.6	243472.7	295377.8	334817.2	333916.7	288570.7	319085.2	288161.1	242316.6	0.365017	0.437687	335363.9	294410.1	Down	-0.1879	0.772871	60S ribosomal protein L4 OS=Mus musculus GN=Rpl4 PE=1 SV=3
Sgip1	Q8VD37-2	14	14	246745.9	202510.5	189687.1	192462.5	213105.3	194487.9	205384	201516.1	199659.6	189812.8	0.365162	0.437514	208902.3	198172.1	Down	-0.07607	0.772871	Isoform 2 of SH3-containing GRB2-like protein 3-interacting protein 1 OS=Mus musculus GN=Sgip1
Ampd3	O08739	2	2	NA	103523.5	133680	103478	134668.5	156210	110999.5	142014	110562.5	137078.5	0.353805	0.451236	118837.5	131372.9	Up	0.144678	0.772871	AMP deaminase 3 OS=Mus musculus GN=Ampd3 PE=2 SV=2
Stxbp1	O08599	76	76	139347.6	127180.6	124410.2	118397.5	142288.6	118509.7	122153.6	141546.3	128821.9	103136	0.365449	0.437173	130324.9	122833.5	Down	-0.08541	0.772871	Syntaxin-binding protein 1 OS=Mus musculus GN=Stxbp1 PE=1 SV=2
Stxbp1	O08599-2	75	75	139347.6	127180.6	124410.2	118397.5	142288.6	118509.7	122153.6	141546.3	128821.9	103136	0.365449	0.437173	130324.9	122833.5	Down	-0.08541	0.772871	Isoform 2 of Syntaxin-binding protein 1 OS=Mus musculus GN=Stxbp1
Ppp3cb	P48453	7	7	31636.43	76471.86	67425.71	60709.57	77927	71755.14	75078.14	62924	76449.14	72930	0.353811	0.451229	62834.11	71827.28	Up	0.192984	0.772871	Serine/threonine-protein phosphatase 2B catalytic subunit beta isoform OS=Mus musculus GN=Ppp3cb PE=1 SV=2
Dgkz	Q80UP3	2	2	11396.5	60946	61063.5	47549	43205.5	52066	52231	49076	47710	75074.5	0.353985	0.451015	44832.1	55231.5	Up	0.300959	0.772871	Diacylglycerol kinase zeta OS=Mus musculus GN=Dgkz PE=1 SV=2
Myo1d	Q5SYD0	2	2	14941	42168.5	52713	64841	34574	31784	35381	38414	22419	36881	0.365854	0.436692	41847.5	32975.8	Down	-0.34373	0.772871	Unconventional myosin-Id OS=Mus musculus GN=Myo1d PE=1 SV=1
Cyb5a	P56395	2	2	25612	148192	105940.5	87369	78508	55933.5	124410.5	102607	144521.5	146151.5	0.354047	0.450939	89124.3	114724.8	Up	0.364287	0.772871	Cytochrome b5 OS=Mus musculus GN=Cyb5a PE=1 SV=2
Rtn4	Q99P72-3	10	10	32818.17	148137.8	105562.5	148620	135250.8	131963	110388.8	136582.2	135365.8	178784.7	0.354415	0.450488	114077.9	138616.9	Up	0.281084	0.772871	Isoform 2 of Reticulon-4 OS=Mus musculus GN=Rtn4
Abhd6	Q8R2Y0	2	2	56959	236417	279165.5	289174	313859.5	261058.5	310235	307650	315424	230947.5	0.356209	0.448295	235115	285063	Up	0.277914	0.772871	Monoacylglycerol lipase ABHD6 OS=Mus musculus GN=Abhd6 PE=2 SV=1
Reep1	Q8BGH4	3	3	29723.33	37602.33	47403.33	36640	30656.33	41772.67	43295.67	47196.67	38995	31076.67	0.357696	0.446486	36405.06	40467.34	Up	0.152619	0.772871	Receptor expression-enhancing protein 1 OS=Mus musculus GN=Reep1 PE=1 SV=1
Kif2a	P28740-2	2	2	132298	186973.5	351820	244086	254955	203093.5	342067	303083.5	318239	226998	0.358188	0.445889	234026.5	278696.2	Up	0.252021	0.772871	Isoform 2 of Kinesin-like protein KIF2A OS=Mus musculus GN=Kif2a
Cox6c	Q9CPQ1	2	2	445232.5	283935	269476.5	313882.5	328339.5	371590	296795	200049.5	331447.5	228699	0.367312	0.434965	328173.2	285716.2	Down	-0.19987	0.773292	Cytochrome c oxidase subunit 6C OS=Mus musculus GN=Cox6c PE=1 SV=3
Atad1	Q9D5T0	3	3	22510.33	58015.33	72335.67	77702	61712.67	40742.67	83114.67	79950.67	75032.33	73452	0.359443	0.44437	58455.2	70458.47	Up	0.269442	0.772871	ATPase family AAA domain-containing protein 1 OS=Mus musculus GN=Atad1 PE=1 SV=1
Mpp3	O88910	2	2	36429.5	61393	61855	80825.5	39014.5	75348	89258	46537	68042	54514.5	0.360245	0.443402	55903.5	66739.9	Up	0.255611	0.772871	MAGUK p55 subfamily member 3 OS=Mus musculus GN=Mpp3 PE=1 SV=2
Dbi	P31786	3	3	31821.33	39964.33	51594.33	47815.67	65453	51761.33	51940.67	70184	42273.33	55786.67	0.36036	0.443263	47329.73	54389.2	Up	0.200573	0.772871	Acyl-CoA-binding protein OS=Mus musculus GN=Dbi PE=1 SV=2
Pdk3	Q922H2	1	1	NA	28477	25797	32568	NA	59801	NA	NA	35548	NA	0.360648	0.442916	28947.33	47674.5	Up	0.719787	0.772871	[Pyruvate dehydrogenase (acetyl-transferring)] kinase isozyme 3, mitochondrial OS=Mus musculus GN=Pdk3 PE=1 SV=1
Eftud2	O08810	1	1	NA	234611	196481	120674	70480	273098	135744	233988	165072	190280	0.362034	0.441251	155561.5	199636.4	Up	0.35989	0.772871	116 kDa U5 small nuclear ribonucleoprotein component OS=Mus musculus GN=Eftud2 PE=2 SV=1
2900026A(	Q8BRV5	1	1	56628	63367	43943	39981	81104	22790	50464	45298	56200	62570	0.369166	0.432778	57004.6	47464.4	Down	-0.26423	0.77365	Uncharacterized protein KIAA1671 OS=Mus musculus GN=Kiaa1671 PE=2 SV=1
Coq8a	Q60936	2	2	112327	39935	92972.5	89768	90814	108096	68704	66880.5	68895.5	32767.5	0.370047	0.431743	85163.3	69068.7	Down	-0.3022	0.774523	Chaperone activity of bc1 complex-like, mitochondrial OS=Mus musculus GN=Adck3 PE=1 SV=2
Hip1r	Q9JKY5	4	4	NA	105737.5	106545	144810.5	124453.5	109828	108939.5	120222.5	111208.5	102356.5	0.370242	0.431514	120386.6	110511	Down	-0.12349	0.774523	Huntingtin-interacting protein 1-related protein OS=Mus musculus GN=Hip1r PE=1 SV=2
Etfb	Q9DCW4	5	5	2193652	2077067	1790252	2123932	2018328	2230882	2131947	1854566	1523799	1761254	0.37049	0.431224	2040646	1900490	Down	-0.10265	0.774523	Electron transfer flavoprotein subunit beta OS=Mus musculus GN=Etfb PE=1 SV=3
Sqstm1	Q64337	3	3	460000.7	661847.3	561074	455386	558782.7	724443	527966.7	620512	570120.7	514170.3	0.363047	0.440037	539418.1	591442.5	Up	0.132834	0.772871	Sequestosome-1 OS=Mus musculus GN=Sqstm1 PE=1 SV=1
Larp4b	Q6A0A2-3	1	1	65761	93597	90863	66175	81675	62909	30114	101576	78042	62279	0.371567	0.429963	79614.2	66984	Down	-0.24921	0.77551	Isoform 3 of La-related protein 4B OS=Mus musculus GN=Larp4b
Dnajc19	Q9CQV7	3	3	343401.3	130325.7	152610.3	177614	243165.7	219567	174008.3	150862	179959.3	119492	0.372906	0.428401	209423.4	168777.7	Down	-0.3113	0.777251	Mitochondrial import inner membrane translocase subunit TIM14 OS=Mus musculus GN=Dnajc19 PE=2 SV=3
Plec	Q9QXS1-1	36	36	36422.33	96630.33	120435.2	125090.2	121985.9	102519.5	121062.5	120163	124832.8	118776.1	0.363583	0.439396	100112.8	117470.8	Up	0.230676	0.772871	Isoform PLEC-1E,2A of Plectin OS=Mus musculus GN=Plec
Golga3	P55937	2	2	226779.5	188442.5	193413	180209	233402	281709	232988	223506.5	195992.5	185345	0.363781	0.43916	204449.2	223908.2	Up	0.131165	0.772871	Golgin subfamily A member 3 OS=Mus musculus GN=Golga3 PE=1 SV=3
Ap2m1	P84091	9	9	853495	701453.6	706814.4	722213.9	676571.9	649890.6	667112.2	757060.4	751199.8	647802.3	0.374221	0.426872	732109.8	694613.1	Down	-0.07585	0.777251	AP-2 complex subunit mu OS=Mus musculus GN=Ap2m1 PE=1 SV=1
Slc32a1	O35633	3	3	460369.3	260978.7	355968.7	345000.3	505940.3	527030	312294	327634	258347	141358.7	0.37449	0.42656	385651.5	313332.7	Down	-0.2996	0.777251	Vesicular inhibitory amino acid transporter OS=Mus musculus GN=Slc32a1 PE=1 SV=3
Afdn	Q9QZQ1	5	5	158199.8	241652.8	182671.3	193588.3	207231.3	208733.5	213608.3	192089.3	205388	241871.3	0.364402	0.438419	196668.7	212338.1	Up	0.110596	0.772871	Afadin OS=Mus musculus GN=Mllt4 PE=1 SV=3
Rps2	P25444	2	2	531946	75630	172415	80989	284712	271719	132559	160711	107770	21150	0.374838	0.426156	229138.4	138781.8	Down	-0.7234	0.777251	40S ribosomal protein S2 OS=Mus musculus GN=Rps2 PE=2 SV=3
Lypla1	P97823	2	2	69924	59654.5	78129.5	88526	80419	44768	75329.5	73171	75754	71568.5	0.375264	0.425663	75330.6	68118.2	Down	-0.1452	0.777251	Acyl-protein thioesterase 1 OS=Mus musculus GN=Lypla1 PE=1 SV=1
Gnb4	P29387	4	4	119343.3	88874.33	84967.33	73960	80755.67	96986.33	51967	73236.33	95823.67	76298.33	0.375439	0.425461	89580.13	78862.33	Down	-0.18384	0.777251	Guanine nucleotide-binding protein subunit beta-4 OS=Mus musculus GN=Gnb4 PE=2 SV=4
Pdcd6ip	Q9WU78	5	5	25007.25	53108	34776.5	40472	37274.25	34017.25	36670.25	37314	55792.75	62212.75	0.364455	0.438356	38127.6	45201.4	Up	0.245532	0.772871	Programmed cell death 6-interacting protein OS=Mus musculus GN=Pdcd6ip PE=1 SV=3
Rpl35a	O55142	3	3	1009495	262704.7	374838.3	202949.3	391049.7	396574	320970	363900.7	240481.3	184722.7	0.375781	0.425065	448207.4	301329.7	Down	-0.57282	0.777251	60S ribosomal protein L35a OS=Mus musculus GN=Rpl35a PE=1 SV=2
Plec	Q9QXS1-1	35	35	37560.53	99471.56	123661.2	128372.2	125224.3	105457.1	124144.5	123622.7	127857.2	121846	0.3647	0.438064	102858	120585.5	Up	0.229403	0.772871	Isoform PLEC-1H of Plectin OS=Mus musculus GN=Plec
Gart	Q64737-2	2	2	228970.5	116475.5	230679.5	149137.5	180815.5	185262.5	169440	190106.5	128791.5	99212.5	0.376529	0.424202	181215.7	154562.6	Down	-0.22952	0.777251	Isoform Short of Trifunctional purine biosynthetic protein adenosine-3 OS=Mus musculus GN=Gart
Suclg2	Q9Z2I8	1	1	NA	154232	156641	189547	160897	173388	151944	179340	183560	189347	0.36544	0.437184	165329.3	175515.8	Up	0.086259	0.772871	Succinyl-CoA ligase [GDP-forming] subunit beta, mitochondrial OS=Mus musculus GN=Suclg2 PE=1 SV=3
Enah	Q03173-4	7	7	403732.7	266632.3	327689	278555.3	341334.9	363195.6	306185.3	305846.3	250534.1	236101.7	0.377701	0.422852	323588.8	292372.6	Down	-0.14635	0.777251	Isoform 3 of Protein enabled homolog OS=Mus musculus GN=Enah
Dlgap4	B1AZP2	7	7	328903	238913.7	216392.6	223966.1	228474.6	247641.7	242706.3	232887.9	214596.9	187270.3	0.377752	0.422793	247330	225020.6	Down	-0.13638	0.777251	Disks large-associated protein 4 OS=Mus musculus GN=Dlgap4 PE=1 SV=1
Psmd1	Q3TXS7	1	1	117719	87042	79344	54323	107264	107337	56067	80694	82171	48191	0.378261	0.422208	89138.4	74892	Down	-0.25124	0.777251	26S proteasome non-ATPase regulatory subunit 1 OS=Mus musculus GN=Psmd1 PE=1 SV=1
Dynlrb1	P62627	7	7	91591.29	49105	59017.86	56263.57	79040.71	72675.57	61112.71	65971.43	49931.43	38872.86	0.378292	0.422173	67003.69	57712.8	Down	-0.21535	0.777251	Dynein light chain roadblock-type 1 OS=Mus musculus GN=Dynlrb1 PE=1 SV=3
Gucy1b3	O54865	4	4	54292.25	53461.75	76399	69008.25	66717.5	72247.5	77929	71749.5	67775.5	57186.5	0.365494	0.43712	63975.75	69377.6	Up	0.116945	0.772871	Guanylate cyclase soluble subunit beta-1 OS=Mus musculus GN=Gucy1b3 PE=2 SV=1
Rpl23	P62830	13	13	212763.3	186056.1	179614.7	169705.5	192721.7	220528	208467	200146.4	194559.7	170248.8	0.365572	0.437027	188172.3	198790	Up	0.079191	0.772871	60S ribosomal protein L23 OS=Mus musculus GN=Rpl23 PE=1 SV=1
Map2k4	P47809	2	2	52292	57370	75580	42057	95113	57154	56978	66304	56865	34006	0.379283	0.421037	64482.4	54261.4	Down	-0.24898	0.777251	Dual specificity mitogen-activated protein kinase kinase 4 OS=Mus musculus GN=Map2k4 PE=1 SV=2
Robo2	Q7TPD3-2	1	1	134452	119095	151927	142960	176935	173768	133328	123042	122493	101073	0.379321	0.420993	145073.8	130740.8	Down	-0.15008	0.777251	Isoform 2 of Roundabout homolog 2 OS=Mus musculus GN=Robo2
Oxsr1	Q6P9R2	1	1	843235	620343	594374	585106	609936	727657	645727	448904	585110	528749	0.379511	0.420776	650598.8	587229.4	Down	-0.14784	0.777251	Serine/threonine-protein kinase OSR1 OS=Mus musculus GN=Oxsr1 PE=1 SV=1
Glrx3	Q9CQM9	5	5	56621.67	164508.7	147749.7	135018	148468.3	146484	160993.7	141933.7	159368	141779	0.366253	0.436219	130473.3	150111.7	Up	0.202282	0.772871	Glutaredoxin-3 OS=Mus musculus GN=Glrx3 PE=1 SV=1
Tufm	Q8BFR5-2	12	12	397795.5	315640.1	304853.3	351653.2	315683.2	403584.2	334069.3	345636.3	366859.2	338255.4	0.366261	0.436209	337125	357680.9	Up	0.085389	0.772871	Isoform 2 of Elongation factor Tu, mitochondrial OS=Mus musculus GN=Tufm
Map3k11	Q80XI6	1	1	435510	188947	241200	107229	231109	275329	189932	157805	146361	153279	0.381373	0.41865	240799	184541.2	Down	-0.38389	0.778663	Mitogen-activated protein kinase kinase kinase 11 OS=Mus musculus GN=Map3k11 PE=1 SV=1
Taldo1	Q93092	1	1	790229	290355	580488	378093	525670	478315	418652	449927	446676	337695	0.381564	0.418433	512967	426253	Down	-0.26716	0.778663	Transaldolase OS=Mus musculus GN=Taldo1 PE=1 SV=2
Fam213b	Q9DB60	4	4	112384.3	170569.7	118542.7	172095.7	145945.7	141783	168841.3	154306.7	154104.7	167175.7	0.366374	0.436075	143907.6	157242.3	Up	0.127846	0.772871	Prostamide/prostaglandin F synthase OS=Mus musculus GN=Fam213b PE=1 SV=1
Cpne1	Q8C166	1	1	NA	99298	180579	124639	141790	94755	113869	167352	112989	91064	0.381888	0.418064	136576.5	116005.8	Down	-0.23551	0.778663	Copine-1 OS=Mus musculus GN=Cpne1 PE=1 SV=1
Enah	Q03173	5	5	562514.2	368241.6	453402.4	385489.4	473854.6	503208.2	425663.4	425933.6	345710.2	325905.8	0.383738	0.415965	448700.4	405284.2	Down	-0.14682	0.78003	Protein enabled homolog OS=Mus musculus GN=Enah PE=1 SV=2
Cdh20	Q9Z0M3	1	1	91781	78636	65455	75882	67791	98187	69254	59665	40775	62786	0.384658	0.414925	75909	66133.4	Down	-0.19889	0.78003	Cadherin-20 OS=Mus musculus GN=Cdh20 PE=2 SV=1
Enah	Q03173-6	6	6	401801.7	273214.5	342492.7	301407.8	361063.5	377428.3	325984.8	318652.2	259695.5	248387.7	0.385007	0.414531	335996	306029.7	Down	-0.13477	0.78003	Isoform 6 of Protein enabled homolog OS=Mus musculus GN=Enah
Elavl4	Q61701-2	1	1	NA	5683	3123	6448	5200	7816	5087	6104	7777	3845	0.366623	0.43578	5113.5	6125.8	Up	0.260587	0.772871	Isoform 2 of ELAV-like protein 4 OS=Mus musculus GN=Elavl4
Ubl4a	P21126	3	3	118232.3	214928.7	249689.3	229564	243717.3	249917.3	267271.3	233374.7	211628.3	222042.7	0.366684	0.435708	211226.3	236846.9	Up	0.165165	0.772871	Ubiquitin-like protein 4A OS=Mus musculus GN=Ubl4a PE=2 SV=1

Grpel2	O88396	1	1	NA	34430	84154	91940	56069	NA	43867	43541	73894	47544	0.385612	0.413849	66648.25	52211.5	Down	-0.3522	0.78003	GrpE protein homolog 2, mitochondrial OS=Mus musculus GN=Grpel2 PE=2 SV=2
Bckdk	O55028	1	1	NA	42842	26554	24893	77922	42011	26349	36664	25810	20813	0.385804	0.413633	43052.75	30329.4	Down	-0.50539	0.78003	[3-methyl-2-oxobutanoate dehydrogenase [lipoamide]] kinase, mitochondrial OS=Mus musculus GN=Bckdk PE=1 SV=1
Armc1	Q9D7A8	3	3	162613	150489.5	166895.5	188793	215975.5	168898.5	157004	184636	151664	162214.5	0.385987	0.413427	176953.3	164883.4	Down	-0.10192	0.78003	Armadillo repeat-containing protein 1 OS=Mus musculus GN=Armc1 PE=1 SV=1
Chmp5	Q9D7S9	1	1	NA	10226	8288	6326	12131	12872	9413	8457	8307	17718	0.367487	0.434758	9242.75	11353.4	Up	0.29673	0.773292	Charged multivesicular body protein 5 OS=Mus musculus GN=Chmp5 PE=2 SV=1
Ache	P21836	2	2	172892	287073	280985	273390	410405.5	394713.5	296858.5	325917.5	307822.5	303343.5	0.368187	0.433932	284949.1	325731.1	Up	0.192977	0.77365	Acetylcholinesterase OS=Mus musculus GN=Ache PE=1 SV=1
Gas7	Q60780	4	4	473644.3	277475.8	251150.5	288022	215208.8	291495	195149.3	314301.3	254164.5	215571.3	0.386217	0.413169	301100.3	254136.3	Down	-0.24464	0.78003	Growth arrest-specific protein 7 OS=Mus musculus GN=Gas7 PE=1 SV=1
Ppib	P24369	3	3	540084	218826	276624	260139.7	419629.7	351025.7	273848	315352.3	307987.7	143843	0.386311	0.413063	343060.7	278411.3	Down	-0.30125	0.78003	Peptidyl-prolyl cis-trans isomerase B OS=Mus musculus GN=Ppib PE=1 SV=2
Pfkp	Q9WUA3-2	8	8	158234.3	165997.9	162766.7	195823.3	161926.3	135831.3	169965.3	169444.4	174001.3	149587.9	0.386549	0.412795	168949.7	159766	Down	-0.08063	0.78003	Isoform 2 of 6-phosphofructokinase type C OS=Mus musculus GN=Pfkp
Homer1	Q9Z2Y3-3	4	4	155028.3	324706.8	253753.5	365172.8	285660	352407.5	291730.3	334261.8	299401.5	293446.8	0.368525	0.433533	276864.3	314249.6	Up	0.182732	0.77365	Isoform 3 of Homer protein homolog 1 OS=Mus musculus GN=Homer1
Capzb	P47757-4	4	4	289699.7	182531.3	163856	169149.7	163164.7	158820.7	184971.7	170577.3	178533.7	156511	0.388103	0.411053	193680.3	169882.9	Down	-0.18914	0.782004	Isoform 3 of F-actin-capping protein subunit beta OS=Mus musculus GN=Capzb
Hsd17b10	O08756	1	1	94539	100151	158679	246095	107149	196532	155880	159078	209239	139597	0.368619	0.433422	141322.6	172065.2	Up	0.283963	0.77365	3-hydroxyacyl-CoA dehydrogenase type-2 OS=Mus musculus GN=Hsd17b10 PE=1 SV=4
Aspscr1	Q8VBT9-3	2	2	7790	3110	2210	2475	3538	2725	3872	3102	1975	2394	0.389248	0.409774	3824.6	2813.6	Down	-0.44289	0.782481	Isoform 3 of Tether containing UBX domain for GLUT4 OS=Mus musculus GN=Aspscr1
Khsrp	Q3U0V1	4	4	29988.5	62392.75	61966.5	63599.25	100194.8	55504.25	88555	78817.5	87801.75	69087.5	0.36909	0.432868	63628.35	75953.2	Up	0.255441	0.77365	Far upstream element-binding protein 2 OS=Mus musculus GN=Khsrp PE=1 SV=2
Mff	Q6PCP5	9	9	85249.88	150210.1	126690	130844.9	141611.1	179138	136480.3	138645.4	119029.3	132693.1	0.371509	0.430031	126921.2	141197.2	Up	0.153778	0.77551	Mitochondrial fission factor OS=Mus musculus GN=Mff PE=1 SV=1
Rpl32	P62911	6	6	567071.2	418888.2	355663.2	388477.8	451895.2	568158.2	432873	526878.8	448206.7	424422	0.373515	0.427692	436399.1	480107.7	Up	0.13771	0.777251	60S ribosomal protein L32 OS=Mus musculus GN=Rpl32 PE=1 SV=2
Slc12a6	Q924N4	2	2	198637	313682.5	364463	320667	429395.5	361666.5	330502.5	429534	363102	342446	0.374128	0.42698	325369	365450.2	Up	0.167598	0.777251	Solute carrier family 12 member 6 OS=Mus musculus GN=Slc12a6 PE=1 SV=2
Gfm1	Q8K0D5	1	1	NA	142105	162753	196071	147829	187569	175958	162375	150094	211662	0.374537	0.426505	162189.5	177531.6	Up	0.130395	0.777251	Elongation factor G, mitochondrial OS=Mus musculus GN=Gfm1 PE=2 SV=1
Hsph1	Q61699	15	15	222523.7	208863.3	204154.9	204083.7	211848.1	243588.9	217812.8	215411.7	205219.8	206426.3	0.375455	0.425442	210294.7	217691.9	Up	0.049875	0.777251	Heat shock protein 105 kDa OS=Mus musculus GN=Hsph1 PE=1 SV=2
Plppr3	Q7TPB0	1	1	30698	13865	28642	18805	29482	26401	22996	13628	25846	12115	0.393066	0.405535	24298.4	20197.2	Down	-0.26671	0.785793	Lipid phosphate phosphatase-related protein type 3 OS=Mus musculus GN=Lppr3 PE=2 SV=1
Qdpr	Q8BVI4	6	6	1065294	827631.5	788365.5	1037763	732277	641620.2	726296.5	875939.8	876177.7	937133.7	0.393206	0.40538	890266.3	811433.6	Down	-0.13376	0.785793	Dihydropteridine reductase OS=Mus musculus GN=Qdpr PE=1 SV=2
Shank3	O4ACU6-2	7	7	165033.6	140156.7	163167.6	150592.7	171035	172029.1	163229.1	198738.1	148216.1	156557.7	0.376231	0.424545	157997.1	167754.1	Up	0.086449	0.777251	Isoform 2 of SH3 and multiple ankyrin repeat domains protein 3 OS=Mus musculus GN=Shank3
Timm23	Q9WTQ8	1	1	NA	22433	5591	14667	5111	7710	5414	10006	7351	8641	0.393857	0.404661	11950.5	7824.4	Down	-0.61102	0.786181	Mitochondrial import inner membrane translocase subunit Tim23 OS=Mus musculus GN=Timm23 PE=2 SV=1
Srrm2	Q8BTI8-3	1	1	NA	NA	12107	7890	11367	NA	13409	10535	23646	8570	0.377339	0.423268	10454.67	14040	Up	0.425396	0.777251	Isoform 3 of Serine/arginine repetitive matrix protein 2 OS=Mus musculus GN=Srrm2
Gtpbp1	O08582	2	2	NA	16956	17641	12538	15754	21573	14668	12964	20478	18375	0.378704	0.4217	15722.25	17611.6	Up	0.163718	0.777251	GTP-binding protein 1 OS=Mus musculus GN=Gtpbp1 PE=1 SV=2
Tsn	Q62348	1	1	69326	89630	97812	80325	89789	80747	91803	111178	83193	93708	0.379127	0.421215	85376.4	92125.8	Up	0.109768	0.777251	Translin OS=Mus musculus GN=Tsn PE=1 SV=1
Erc1	Q99MI1-4	3	3	370491	385818.3	347391.3	356467.3	329536	392658	328537	402010	360335.7	382276	0.379747	0.420506	357940.8	373163.3	Up	0.060086	0.777251	Isoform 3 of ELKS/Rab6-interacting/CAST family member 1 OS=Mus musculus GN=Erc1
Rpl22	P67984	7	7	1180568	760087.2	819056.2	649895.4	883436.8	953842.6	725477.4	792863.6	698582.8	652165.4	0.396205	0.40208	858608.7	764586.4	Down	-0.16732	0.7878	60S ribosomal protein L22 OS=Mus musculus GN=Rpl22 PE=1 SV=2
Ndufa6	Q9CQZ5	5	5	1467721	550460	516167.8	609380	735277.5	754018.8	600409	569771.3	635930	463723	0.397043	0.401162	775801.3	604770.4	Down	-0.3593	0.788853	NADH dehydrogenase [ubiquinone] 1 alpha subcomplex subunit 6 OS=Mus musculus GN=Ndufa6 PE=1 SV=1
Bcan	Q61361	4	4	40350.25	25757.75	27306.25	45663.25	62946.75	27089.25	38881.75	42222	34819.75	24404.5	0.397707	0.400437	40404.85	33483.45	Down	-0.27108	0.789561	Brevican core protein OS=Mus musculus GN=Bcan PE=1 SV=2
Hk2	O08528	2	2	780800	739801.5	950989	665418	849030.5	663904.5	1019701	517530	779053.5	531389.5	0.398901	0.399135	797207.8	702315.7	Down	-0.18284	0.789808	Hexokinase-2 OS=Mus musculus GN=Hk2 PE=2 SV=1
Ncam2	O35136	3	3	41958.5	102672	93592	87358	119319.5	131014.5	104197.5	90026	96082	93780.5	0.379983	0.420236	88980	103020.1	Up	0.211373	0.777251	Neural cell adhesion molecule 2 OS=Mus musculus GN=Ncam2 PE=2 SV=1
Atp5b	P56480	60	60	583288.7	890058.5	782095	1001985	654909.2	1020994	903995.7	745895.7	817823.6	840702.2	0.381821	0.41814	782467.3	865882.2	Up	0.14614	0.778663	ATP synthase subunit beta, mitochondrial OS=Mus musculus GN=Atp5b PE=1 SV=2
Vhl	P40338	3	3	NA	2392.33	1763	2571.67	4062.33	1917.33	1457.67	2416.67	3000	2140.33	0.399153	0.398861	2697.333	2186.4	Down	-0.30298	0.789808	Von Hippel-Lindau disease tumor suppressor OS=Mus musculus GN=Vhl PE=1 SV=1
Nt5c	Q9JM14	4	4	614740.7	292898.7	280586.3	276502	225716	318611.3	263863.7	238484.3	249897.7	285624.3	0.399552	0.398427	338088.7	271296.3	Down	-0.31753	0.789808	5'(3')-deoxyribonucleotidase, cytosolic type OS=Mus musculus GN=Nt5c PE=1 SV=1
Hmox2	O70252	2	2	133682.5	70792.5	191780.5	132488	239374	135254.5	134402	179144.5	101210	62821	0.400059	0.397876	153623.5	122566.4	Down	-0.32584	0.789808	Heme oxygenase 2 OS=Mus musculus GN=Hmox2 PE=2 SV=1
Syngap1	F6SEU4	28	28	89992.16	110737.4	128412.6	119101	130624.2	118298.3	111379.9	105880	107542.5	99314.68	0.400571	0.39732	115773.5	108483.1	Down	-0.09383	0.789808	Ras/Rap GTPase-activating protein SynGAP OS=Mus musculus GN=Syngap1 PE=1 SV=2
Erc1	Q99MI1	4	4	278711.5	290307.8	261249	268168.5	247859.5	295429.5	247070.3	302254.5	270803	287365.5	0.385281	0.414222	269259.3	280584.6	Up	0.05944	0.78003	ELKS/Rab6-interacting/CAST family member 1 OS=Mus musculus GN=Erc1 PE=1 SV=1
Ampd2	Q9DBT5	1	1	18057	15815	20852	18693	12438	18958	18832	17377	18724	19011	0.385383	0.414107	17171	18580.4	Up	0.113807	0.78003	AMP deaminase 2 OS=Mus musculus GN=Ampd2 PE=1 SV=1
Prdx1	P35700	7	7	526342.9	404404	416025.4	375909.1	397268.7	393004.7	429600.3	390295.3	361419	414942.3	0.403047	0.394644	423990	397852.3	Down	-0.0918	0.789808	Peroxiredoxin-1 OS=Mus musculus GN=Prdx1 PE=1 SV=1
Sorbs1	Q62417-3	2	2	395781	238501	266952	235918.5	438685.5	371686.5	317422	262911.5	212615	161673	0.40312	0.394566	315167.6	265261.6	Down	-0.2487	0.789808	Isoform 3 of Sorbin and SH3 domain-containing protein 1 OS=Mus musculus GN=Sorbs1
Rgs10	Q9CQE5	4	4	30154	73617	45821.5	47808.75	38649.5	42520.25	61124.25	54947.5	60668.5	54502.25	0.386086	0.413316	47210.15	54752.55	Up	0.213829	0.78003	Regulator of G-protein signaling 10 OS=Mus musculus GN=Rgs10 PE=2 SV=1
Shank3	O4ACU6-3	16	16	112462.9	282870.9	166682.9	225880.3	192383.3	261893.9	187451	189530.3	239785	253785.9	0.386147	0.413247	196056	226489.2	Up	0.208176	0.78003	Isoform 3 of SH3 and multiple ankyrin repeat domains protein 3 OS=Mus musculus GN=Shank3
Cadps2	Q8BYR5-3	3	3	34394	129298	139120	145013	125806	138608	144569.5	130355	132686.5	126881.5	0.386819	0.412492	114726.2	134620.1	Up	0.230699	0.78003	Isoform 3 of Calcium-dependent secretion activator 2 OS=Mus musculus GN=Cadps2
1110004F10Rik	Q9R0P4	1	1	252934	106065	99860	65003	138295	125011	98373	99504	117319	67437	0.404648	0.392923	132419.8	101528.8	Down	-0.38323	0.789808	Small acidic protein OS=Mus musculus GN=Smap PE=1 SV=1
Rasgrf1	P27671	2	2	47224.5	92418	112924.5	105403.5	119122.5	131067.5	136038.5	95605	108280.5	81418	0.389222	0.409803	95418.6	110481.9	Up	0.211468	0.782481	Ras-specific guanine nucleotide-releasing factor 1 OS=Mus musculus GN=Rasgrf1 PE=1 SV=2
Glod4	Q9CPV4-3	2	2	NA	NA	4190	12947.5	16398	24994	14796	14918	4876	19477.5	0.389256	0.409765	11178.5	15812.3	Up	0.500321	0.782481	Isoform 3 of Glyoxalase domain-containing protein 4 OS=Mus musculus GN=Glod4
Prr7	Q3V0I2	1	1	NA	130521	238138	66442	155590	216118	110778	279938	87966	299408	0.389693	0.409277	147672.8	198841.6	Up	0.429216	0.782746	Proline-rich protein 7 OS=Mus musculus GN=Prr7 PE=2 SV=1
Anks1b	Q8BIZ1-3	2	2	95927.5	224801	246035.5	235416.5	184153	206034.5	236387	190216.5	235135.5	258019	0.390688	0.40817	197266.7	225158.5	Up	0.190794	0.783679	Isoform 3 of Ankyrin repeat and sterile alpha motif domain-containing protein 1B OS=Mus musculus GN=Anks1b
Endod1	Q8C522	7	7	120296.3	171569.3	166376.2	158937.8	172504.5	199519.3	150182.3	165652.3	175416.2	157684	0.39077	0.408079	157936.8	169690.8	Up	0.103561	0.783679	Endonuclease domain-containing 1 protein OS=Mus musculus GN=Endod1 PE=1 SV=2
Mapk10	Q61831	1	1	624319	689361	647097	686825	719462	655783	768671	661797	595398	386767	0.406874	0.39054	673412.8	613683.2	Down	-0.134	0.789808	Mitogen-activated protein kinase 10 OS=Mus musculus GN=Mapk10 PE=1 SV=2
Sorbs1	Q62417	3	3	287675.3	189080.7	215073.7	185726	365878.7	288217	246068.7	205274.3	174818.7	138192.7	0.406887	0.390526	248686.9	210514.3	Down	-0.24041	0.789808	Sorbin and SH3 domain-containing protein 1 OS=Mus musculus GN=Sorbs1 PE=1 SV=2
Cast	P51125-3	1	1	NA	21047	19334	23414	20307	12464	26959	25141	23943	32903	0.391898	0.406827	21025.5	24282	Up	0.207747	0.785328	Isoform 3 of Calpastatin OS=Mus musculus GN=Cast
Tpi1	P17751	150	150	579493.5	522805.6	445028.3	429915.5	468983.3	548151.7	455319.3	443012.4	417605.9	423429.9	0.407829	0.389522	489245.2	457503.8	Down	-0.09677	0.789808	Triosephosphate isomerase OS=Mus musculus GN=Tpi1 PE=1 SV=4
Nsf	P46460	24	24	778992.5	686125.4	777757.3	641514.5	712496.1	627556.2	750984.1	756070.5	692927.7	576987.3	0.408011	0.389328	719377.2	680905.1	Down	-0.07929	0.789808	Vesicle-fusing ATPase OS=Mus musculus GN=Nsf PE=1 SV=2
Echs1	Q8BH95	7	7	127194.3	116273.3	139914	117948.5	126528.5	128499.8	118550.3	129137	103485.8	120821.8	0.408059	0.389277	125571.7	120098.9	Down	-0.06429	0.789808	Enoyl-CoA hydratase, mitochondrial OS=Mus musculus GN=Echs1 PE=1 SV=1
Fbxl16	A2RT62	2	2	145477	176348	168644.5	139908.5	109957	188581	177311.5	171897	178077.5	109986	0.392708	0.40593	148467	165170.6	Up	0.153815	0.785793	F-box/LRR-repeat protein 16 OS=Mus musculus GN=Fbxl16 PE=2 SV=1
Mrpl51	Q9CPY1	2	2	564227.5	400288.5	413388.5	519500.5	454615	544543	463748.5	409344.5	303790.5	410663.5	0.408364	0.388953	470404	426418	Down	-0.14163	0.789808	39S ribosomal protein L51, mitochondrial OS=Mus musculus GN=Mrpl51 PE=2 SV=1
Cep170b	Q80U49	7	7	121142.7	125320.3	123042.3	83415.5	165001.2	124408.5	118341.7	113088.3	105907.2	93526.17	0.408709	0.388586	123584.4	111054.4	Down	-0.15423	0.789808	Centrosomal protein of 170 kDa protein B OS=Mus musculus GN=Cep170b PE=1 SV=2
Tpm3	P21107-2	2	2	466499	309998	316774	285359	316782	345229	307752	321094	284108	282154	0.40906	0.388213	339082.4	308067.4	Down	-0.13839	0.789808	Isoform 2 of Tropomyosin alpha-3 chain OS=Mus musculus GN=Tpm3
Oxct1	Q9D0K2	19	19	56974.93	51096.57	51078.57	60819.93	56112.14	48503.43	52355.5	40457.57	51983.5	63990.5	0.409117	0.388152	55216.43	51458.1	Down	-0.1017	0.789808	Succinyl-CoA:3-ketoacid coenzyme A transferase 1, mitochondrial OS=Mus musculus GN=Oxct1 PE=1 SV=1
Cntn1	P12960	14	14	122495.7	240927.9	261620.6	238992.3	263010.2	227725.5	272358.7	236008.1	249492.5	271019.7	0.393356	0.405214	225409.3	251320.9	Up	0.156983	0.785793	Contactin-1 OS=Mus musculus GN=Cntn1 PE=1 SV=1
Ntrk2	P15209-4	5	5	246975.2	189468.8	244810.6	240819.6	285218.2	289227.8	271946	270932.6	252870.8	212735	0.394617	0.403824	241458.5	259542.4	Up	0.104195	0.786497	Isoform L10 of BDNF/NT-3 growth factors receptor OS=Mus musculus GN=Ntrk2
Alb	P07724	16	16	274350.1	226698.6	165960.1	163901.3	171816.4	156863.3	207519.5	249543.9	224213.1	320690.9	0.39463	0.40381	200545.3	231766.2	Up	0.208742	0.786497	Serum albumin OS=Mus musculus GN=Alb PE=1 SV=3
Pitpna	P53810	2	2	86878.5	83807.5	79749.5	67858.5	74298	91954	81817.5	79048	77490.5	81393	0.39528	0.403095	78518.4	82340.6	Up	0.068573	0.787182	Phosphatidylinositol transfer protein alpha isoform OS=Mus musculus GN=Pitpna PE=1 SV=2
Bloc1s1	O55102	1	1	21157	18864	22569	13943	24774	35783	20654	21227	18954	21046	0.395737	0.402593	20261.4	23532.8	Up	0.215939	0.787479	Biogenesis of lysosome-related organelles complex 1 subunit 1 OS=Mus musculus GN=Bloc1s1 PE=1 SV=2
Ptgr2	Q8VDQ1	4	4	103899	130466.5	139058.5	118200.5	151341.8	111469	120373.8	133584.5	121687.5	115903.5	0.411875	0.385235	128593.3	120603.7	Down	-0.09254	0.789808	Prostaglandin reductase 2 OS=Mus musculus GN=Ptgr2 PE=1 SV=2
Cbr1	P48758	14	14	787936.9	547591.5	585076.1	545104.4	634883.6	580323.2	641562.3	588580.5	520576.6	550904.5	0.412133	0.384963	620118.5	576389.4	Down	-0.1055	0.789808	Carbonyl reductase [NADPH] 1 OS=Mus musculus GN=Cbr1 PE=1 SV=3
Isca2	Q9DCB8	2	2	9226.5	11834	24772	18837	17775.5	14052	21315.5	27817.5	13671.5	22723	0.398929	0.399104	16489	19915.9	Up	0.272417	0.789808	Iron-sulfur cluster assembly 2 homolog, mitochondrial OS=Mus musculus GN=Isca2 PE=2 SV=2
Sdf2l1	Q9ESP1	3	3	96045.5	80321.5	70740	49257.5	75534	111363.5	74275	84257.5	73576	74964.5	0.399124	0.398892	74379.7	83687.3	Up	0.1701	0.789808	Stromal cell-derived factor 2-like protein 1 OS=Mus musculus GN=Sdf2l1 PE=2 SV=2
Ppp1r21	Q3TDD9	1	1	30372	27383	25248	21633	23784	27612	30335	33805	27465	20836	0.40115	0.396693	25684	28010.6	Up	0.125103	0.789808	Protein phosphatase 1 regulatory subunit 21 OS=Mus musculus GN=Ppp1r21 PE=2 SV=2
Acaa1a	Q921H8	10	10	220691.5	118965.6	124354	104162.8	144499.8	147406	129389.6	129143.3	115747.6	90862	0.412363	0.38472	142536.7	122509.7	Down	-0.21842	0.789808	3-ketoacyl-CoA thiolase A, peroxisomal OS=Mus musculus GN=Acaa1a PE=1 SV=1
Necap1	Q9CR95	10	10	275912.3	242823	220862	258869.9	262276.5	271250.9	223890.3	225748.4	236855.1	247645.3	0.412671	0.384396	252148.7	241078	Down	-0.06478	0.789808	Adaptin ear-binding coat-associated protein 1 OS=Mus musculus GN=Necap1 PE=1 SV=2
Fam120c	Q8C3F2	1	1	108321	111185	145479	118125	43874	80798	174365	125767	130131	115610	0.401168	0.396674	105396.8	125334.2	Up	0.249949	0.789808	Constitutive coactivator of PPAR-gamma-like protein 2 OS=Mus musculus GN=Fam120c PE=2 SV=3
Tfam	P40630	1	1	NA	701287	551936	915108	537179	644380	575622	539875	602444	599902	0.412785	0.384276	676377.5	592444.6	Down	-0.19115	0.789808	Transcription factor A, mitochondrial OS=Mus musculus GN=Tfam PE=1 SV=2
Ndufb4	Q9CQC7	5	5	159986	200408	166487.3	195896.7	162500.3	156455	169250.3	267989	229528.3	167849	0.403721	0.393919	177055.7	198214.3	Up	0.162858	0.789808	NADH dehydrogenase [ubiquinone] 1 beta subcomplex subunit 4 OS=Mus musculus GN=Ndufb4 PE=1 SV=3
Eprs	Q8CGC7	2	2	47404	46429.5	42460.5	36197	41629	49730	52683.5	44711	40343.5	40616	0.403897	0.393729	42824	45616.8	Up	0.091146	0.789808	Bifunctional glutamate/proline--tRNA ligase OS=Mus musculus GN=Eprs PE=1 SV=4
Rps12	P63323	5	5	543669.4	683256.8	465920	511621.2	529310.8	664814.8	562472.6	548051.4	622622.2	404471	0.413113	0.383963	546755.6	585993.7	Up	0.099989	0.789808	40S ribosomal protein S12 OS=Mus musculus GN=Rps12 PE=1 SV=2
Tmem120a	Q8C1E7	1	1	149185	191861	237380	174344	197191	212958	229059	218146	191867	175226	0.40497	0.392577	189992.2	205451.2	Up	0.112856	0.789808	Transmembrane protein 120A OS=Mus musculus GN=Tmem120a PE=2 SV=1
Etfdh	Q921G7	1	1	153195	140885	133405	138907	126519	149287	107489	158325	122949	101125	0.413912	0.383092	138582.2	127835	Down	-0.11646	0.789808	Electron transfer flavoprotein-ubiquinone oxidoreductase, mitochondrial OS=Mus musculus GN=Etfdh PE=1 SV=1
Prdx3	P20108	1	1	65341	213730	101574	161800	157629	194462	154780	133105	160669	183213	0.405199	0.392332	140014.8	165245.8	Up	0.239034	0.789808	Thioredoxin-dependent peroxide reductase, mitochondrial OS=Mus musculus GN=Prdx3 PE=1 SV=1
Psd3	Q2PFD7-4	5	5	1641428	62552.75	281507.5	140020	516745.5	547767.5	236069	260963.8	171329.3	83347.5	0.41404	0.382958	528450.7	259895.4	Down	-1.02384	0.789808	Isoform 4 of PH and SEC7 domain-containing protein 3 OS=Mus musculus GN=Psd3
Aprt	P08030	2	2	4074.5	11309.5	14932.5	15931.5	9108	3718.5	15431.5	18063	16014.5	17014.5	0.405403	0.392113	11071.2	14048.4	Up	0.343594	0.789808	Adenine phosphoribosyltransferase OS=Mus musculus GN=Aprt PE=1 SV=2
Btf3	Q64152	3	3	70796.33	38433.33	40055.33	43536	55045.33	48582.33	48498.33	44113.67	38597	39562.33	0.414589	0.382382	49573.26	43870.73	Down	-0.1763	0.789808	Transcription factor BTF3 OS=Mus musculus GN=Btf3 PE=1 SV=3
Efhd2	Q9D8Y0	6	6	736914.3	788016.3	663422.3	647771.2	515570	546832.2	703711.5	609930.7	698387	579251.3	0.414926	0.382029	670338.8	627622.5	Down	-0.10272	0.789808	EF-hand domain-containing protein D2 OS=Mus musculus GN=Efhd2 PE=1 SV=1
Nkiras1	Q8CEC5	1	1	516862	305121	257522	215803	271314	273900	292780	260376	260421	237046	0.415089	0.381859	313324.4	264904.6	Down	-0.24218	0.789808	NF-kappa-B inhibitor-interacting Ras-like protein 1 OS=Mus musculus GN=Nkiras1 PE=1 SV=1
Adss	P46664	2	2	8361.5	46736.5	30747.5	46011.5	36082	55542.5	50227.5	14911.5	101005	21600	0.406352	0.391098	33587.8	48657.3	Up	0.534719	0.789808	Adenylosuccinate synthetase isozyme 2 OS=Mus musculus GN=Adss PE=1 SV=2
Pccb	Q99MN9	1	1	NA	8608	18200	17066	7104	13064	19840	13180	15923	16059	0.406357	0.391092	12744.5	15613.2	Up	0.292891	0.789808	Propionyl-CoA carboxylase beta chain, mitochondrial OS=Mus musculus GN=Pccb PE=1 SV=2
Cyct	P00015	2	2	3517792	3955598	3977782	4319019	3492463	4273626	3648369	3937905	3686074	5057783	0.406908	0.390504	3852531	4120751	Up	0.097101	0.789808	Cytochrome c, testis-specific OS=Mus musculus GN=Cyct PE=1 SV=3
Sri	Q6P069	1	1	NA	17281	12591	15294	NA	17083	15828	10618	7111	0.417089	0.379771	15055.33	12660	Down	-0.25	0.790842		Sorcin OS=Mus musculus GN=Sri PE=1 SV=1
Phyhip	Q8K0S0	2	2	511058	1125167	544951	883159.5	602644	596865.5	866828	873395	781122	1235212	0.408146	0.389184	733395.8	870684	Up	0.247557	0.789808	Phytanoyl-CoA hydroxylase-interacting protein OS=Mus musculus GN=Phyhip PE=1 SV=1
Ccs	Q9WU84	1	1	27578	NA	19900	3812	8048	8547	12975	8487	12581	5702	0.417483	0.379361	14834.5	9658.4	Down	-0.6191	0.790842	Copper chaperone for superoxide dismutase OS=Mus musculus GN=Ccs PE=2 SV=1
Emc4	Q9CZX9	1	1	875704	679325	892444	912055	1205388	1009858	905841	842138	725661	617727	0.41874	0.378056	912983.2	820245	Down	-0.15453	0.792484	ER membrane protein complex subunit 4 OS=Mus musculus GN=Emc4 PE=2 SV=1
S100b	P50114	1	1	37857	34126	46375	38636	43220	38202	41472	49333	34033	59162	0.409263	0.387998	40042.8	44440.4	Up	0.150329	0.789808	Protein S100-B OS=Mus musculus GN=S100b PE=2 SV=2
Sucla2	Q9Z2I9	7	7	271019	270994.4	289527.4	397768.3	308006.6	319875.9	270250	328242.9	258366	233183.7	0.419277	0.377499	307463.1	281983.7	Down	-0.1248	0.792484	Succinyl-CoA ligase [ADP-forming] subunit beta, mitochondrial OS=Mus musculus GN=Sucla2 PE=1 SV=2
Cct6b	Q61390	3	3	2527753	1432030	1410730	1113159	1571354	1740571	1413202	1208957	1342790	1211994	0.419615	0.377149	1611005	1383503	Down	-0.21964	0.792538	T-complex protein 1 subunit zeta-2 OS=Mus musculus GN=Cct6b PE=2 SV=4
Hspe1	Q64433	21	21	1612712	1463861	1205552	1406256	1269484	1644500	1209150	1138829	1137496	1320288	0.420003	0.376748	1391573	1290053	Down	-0.10929	0.792686	10 kDa heat shock protein, mitochondrial OS=Mus musculus GN=Hspe1 PE=1 SV=2
Cacng3	Q9JJV5	1	1	311244	465855	522191	437502	354411	507444	463852	446223	350374	541029	0.409767	0.387463	418240.6	461784.4	Up	0.142886	0.789808	Voltage-dependent calcium channel gamma-3 subunit OS=Mus musculus GN=Cacng3 PE=2 SV=2
Cd81	P35762	1	1	NA	NA	29260	24336	23726	NA	77236	18326	28101	29886	0.411134	0.386017	25774	38387.25	Up	0.574711	0.789808	CD81 antigen OS=Mus musculus GN=Cd81 PE=1 SV=2
Dcx	O88809	2	2	20739.5	20319.5	20694.5	17679	23177.5	20118	21402.5	22353	18250.5	10509.5	0.421495	0.375208	20522	18526.7	Down	-0.14757	0.793749	Neuronal migration protein doublecortin OS=Mus musculus GN=Dcx PE=1 SV=1
Rac2	Q05144	1	1	64146	67387	87460	61412	70733	33756	79193	119068	106094	81939	0.411146	0.386004	70227.6	84010	Up	0.258523	0.789808	Ras-related C3 botulinum toxin substrate 2 OS=Mus musculus GN=Rac2 PE=2 SV=1
Wasf3	Q8VHI6	2	2	NA	1658	2450	1602	2291	NA	1838	1647	2177	1400	0.42285	0.373814	2000.25	1765.5	Down	-0.1801	0.795133	Wiskott-Aldrich syndrome protein family member 3 OS=Mus musculus GN=Wasf3 PE=2 SV=1
Tomm70a	Q9CZW5	8	8	137555.4	272763	273004.8	297176.8	266269.4	233334.5	324332.4	245891.1	267476.8	326150	0.41151	0.38562	249353.9	279437	Up	0.164328	0.789808	Mitochondrial import receptor subunit TOM70 OS=Mus musculus GN=Tomm70a PE=1 SV=2
Mthfd1	Q922D8	5	5	2740.2	3352.4	5316.5	5427.4	56279	33234	61930.8	55910.8	40449.2	59616.6	0.412208	0.384884	39996.52	50228.28	Up	0.328625	0.789808	C-1-tetrahydrofolate synthase, cytoplasmic OS=Mus musculus GN=Mthfd1 PE=1 SV=4
Cpne2	P59108	2	2	210767.5	222513	267843	235620.5	253617.5	275630	226479.5	266482	227262	266740.5	0.412236	0.384854	240072.3	252518.8	Up	0.072922	0.789808	Copine-2 OS=Mus musculus GN=Cpne2 PE=2 SV=1
Macf1	Q9QXZ0-3	11	11	84189	144961.2	133112.6	131329.6	144305.1	146775.4	156235.1	133078	134454.1	122897	0.412334	0.384751	127579.5	138687.9	Up	0.120446	0.789808	Isoform 3 of Microtubule-actin cross-linking factor 1 OS=Mus musculus GN=Macf1
Dip2b	Q3UH60	2	2	NA	109337	155172.5	114707	146774.5	135819.5	152373.5	111440.5	103023	84787.5	0.425603	0.370995	131497.8	117488.8	Down	-0.16251	0.797383	Disco-interacting protein 2 homolog B OS=Mus musculus GN=Dip2b PE=1 SV=1
C530008M17Rik	Q5PR69	1	1	NA	177831	177665	172663	242101	200472	203373	142050	179671	148059	0.426661	0.369917	192565	174725	Down	-0.14026	0.798011	Uncharacterized protein KIAA1211 OS=Mus musculus GN=Kiaa1211 PE=2 SV=3
Cct8	P42932	4	4	592030	225346.3	292593	184773	308787	328143.3	240229.7	265170.3	241321.7	205525.3	0.427064	0.369507	320705.9	256078.1	Down	-0.32467	0.798011	T-complex protein 1 subunit theta OS=Mus musculus GN=Cct8 PE=1 SV=3
Hsp90ab1	P11499	11	11	419384.8	226468.8	166999.2	189233.8	205401.4	231905.4	163421.8	213149.6	200447.4	193928.6	0.42715	0.36942	241497.6	200570.6	Down	-0.2679	0.798011	Heat shock protein HSP 90-beta OS=Mus musculus GN=Hsp90ab1 PE=1 SV=3
Hspb1	P14602	3	3	NA	3176.33	2875.33	3453.67	4088	1033	7763	4487.33	4368.33	4284.67	0.412734	0.38433	3398.333	4387.266	Up	0.368495	0.789808	Heat shock protein beta-1 OS=Mus musculus GN=Hspb1 PE=1 SV=3
Cdc42bpb	Q7TT50	7	7	54230	66094.33	45513	57878.33	55069.5	72030	54049	55786.33	52400.67	66493.33	0.413012	0.384037	55757.03	60151.87	Up	0.109456	0.789808	Serine/threonine-protein kinase MRCK beta OS=Mus musculus GN=Cdc42bpb PE=1 SV=2
Lypla2	Q9WTL7	1	1	NA	22977	39637	NA	NA	47634	21693	55843	43005		0.413124	0.38392	31307	42043.75	Up	0.425406	0.789808	Acyl-protein thioesterase 2 OS=Mus musculus GN=Lypla2 PE=1 SV=1
Dtx3	Q80V91	3	3	76846.67	60684	73107	69510	97287.67	83295.33	68221.33	79616.67	46262.67	62610.67	0.428209	0.368344	75487.07	68001.33	Down	-0.15067	0.798181	Probable E3 ubiquitin-protein ligase DTX3 OS=Mus musculus GN=Dtx3 PE=2 SV=2
Acot9	Q9R0X4	1	1	60132	215037	159339	191395	123638	171511	167829	167030	178654	189428	0.413224	0.383814	149908.2	174890.4	Up	0.222372	0.789808	Acyl-coenzyme A thioesterase 9, mitochondrial OS=Mus musculus GN=Acot9 PE=1 SV=1
Ndufa13	Q9ERS2	3	3	156356	407960	347292.7	347561.7	254353.7	339290	394196.7	360630	290996.3	338264	0.413245	0.383792	302704.8	344675.4	Up	0.187327	0.789808	NADH dehydrogenase [ubiquinone] 1 alpha subcomplex subunit 13 OS=Mus musculus GN=Ndufa13 PE=1 SV=3
Pitpnc1	Q8K4R4	4	4	135934.3	317058.5	242452	265441.8	242935.8	259225.3	263747.8	257963.5	281979.5	276333.8	0.413915	0.383089	240764.5	267850	Up	0.153803	0.789808	Cytoplasmic phosphatidylinositol transfer protein 1 OS=Mus musculus GN=Pitpnc1 PE=1 SV=1
Llgl1	Q80Y17	1	1	203126	296606	265910	371369	236016	308573	291873	293380	292873	318389	0.414239	0.382749	274605.4	301017.6	Up	0.132488	0.789808	Lethal(2) giant larvae protein homolog 1 OS=Mus musculus GN=Llgl1 PE=1 SV=1
Shank3	Q4ACU6	17	17	144359.6	291152.4	186329.9	236220.8	207527.8	273421.5	199424.9	206378.7	249065.1	262215.8	0.415638	0.381285	213118.1	238101.2	Up	0.159922	0.790267	SH3 and multiple ankyrin repeat domains protein 3 OS=Mus musculus GN=Shank3 PE=1 SV=3
Hip1	Q8VD75	1	1	12840	15235	19350	18590	15094	14480	16950	18516	18819	18645	0.416157	0.380743	16221.8	17482	Up	0.107936	0.790668	Huntingtin-interacting protein 1 OS=Mus musculus GN=Hip1 PE=1 SV=2
Slc4a4	O88343	1	1	24633	33792	51483	47178	62808	49668	41592	38804	35570	19569	0.431396	0.365124	43978.8	37040.6	Down	-0.2477	0.799826	Electrogenic sodium bicarbonate cotransporter 1 OS=Mus musculus GN=Slc4a4 PE=1 SV=2
Rpl3	P27659	6	6	1013225	241908.5	333696.7	234625.2	446720.8	420068.3	301282.3	339555.2	314576.8	254858.3	0.431588	0.364931	454035.2	326068.2	Down	-0.47763	0.799826	60S ribosomal protein L3 OS=Mus musculus GN=Rpl3 PE=1 SV=3
Lurap1	Q9D6I9	1	1	15846	7307	5700	3801	5712	9116	7398	8454	10282	13406	0.41692	0.379947	7673.2	9731.2	Up	0.342789	0.790842	Leucine rich adaptor protein 1 OS=Mus musculus GN=Lurap1 PE=1 SV=1
Mcu	Q3UMR5	1	1	56747	163861	182285	174340	143973	155246	161708	173538	152234	183404	0.417425	0.379422	144241.2	165226	Up	0.195957	0.790842	Calcium uniporter protein, mitochondrial OS=Mus musculus GN=Mcu PE=1 SV=2
Cep41	Q99NF3	1	1	113379	95146	84451	81677	97884	117017	102723	103793	88710	92302	0.419076	0.377707	94507.4	100909	Up	0.094556	0.792484	Centrosomal protein of 41 kDa OS=Mus musculus GN=Cep41 PE=1 SV=1
Me3	Q8BMF3	1	1	57485	83958	90833	80981	103013	101502	94767	80877	87216	87733	0.421205	0.375506	83254	90419	Up	0.119106	0.793749	NADP-dependent malic enzyme, mitochondrial OS=Mus musculus GN=Me3 PE=1 SV=2
Mrpl19	Q9D338	1	1	NA	56419	78259	68281	83158	84435	87525	69040	71998	74875	0.421249	0.375461	71529.25	77574.6	Up	0.117051	0.793749	39S ribosomal protein L19, mitochondrial OS=Mus musculus GN=Mrpl19 PE=2 SV=1
Ube2d2a	P62838	1	1	NA	70426	96738	87133	68474	84822	63043	84064	72506	64293	0.4338	0.36271	80692.75	73745.6	Down	-0.12988	0.800453	Ubiquitin-conjugating enzyme E2 D2 OS=Mus musculus GN=Ube2d2 PE=1 SV=1
Nit2	Q9JHW2	4	4	49191.5	86298.75	74714.5	94974.5	72979.5	77838	86697.75	70287.75	85600	95692	0.422358	0.374319	75631.75	83223.1	Up	0.137992	0.794789	Omega-amidase NIT2 OS=Mus musculus GN=Nit2 PE=1 SV=1
Eif2s1	Q6ZWX6	1	1	NA	21751	42740	36093	42536	26609	NA	19292	34081	40602	0.435756	0.360757	35780	30146	Down	-0.24719	0.802906	Eukaryotic translation initiation factor 2 subunit 1 OS=Mus musculus GN=Eif2s1 PE=1 SV=3
Mapre1	Q61166	3	3	137995.3	60077.67	70890	74404	93342.67	78338.67	88876.33	81096	77668.33	49337.67	0.436423	0.360092	89341.93	75063.4	Down	-0.25123	0.803558	Microtubule-associated protein RP/EB family member 1 OS=Mus musculus GN=Mapre1 PE=1 SV=3
Hist2h3c1	P84228	2	2	NA	1759.5	21246	67814.5	NA	27637.5	2914.5	2139	NA		0.43676	0.359757	30273.33	10897	Down	-1.47412	0.803601	Histone H3.2 OS=Mus musculus GN=Hist1h3b PE=1 SV=2
Rps19	Q9CZX8	7	7	1775423	755808.1	692388.4	581049.6	1080808	1070315	709049.6	849304.7	656106.4	628002.1	0.43737	0.359151	977095.5	782555.6	Down	-0.32031	0.804145	40S ribosomal protein S19 OS=Mus musculus GN=Rps19 PE=1 SV=3
Letm1	Q9Z2I0	7	7	4460	95149.43	86224.43	94193.71	60097.14	78816.14	84343.86	77299.86	87874.14	88263.29	0.424013	0.372621	68024.94	83319.46	Up	0.29259	0.796176	LETM1 and EF-hand domain-containing protein 1, mitochondrial OS=Mus musculus GN=Letm1 PE=2 SV=1
Rab6a	P35279	2	2	75310.5	83044	105773	87398	114040.5	97045.5	99028	97878.5	114772	91979	0.424027	0.372606	93113.2	100140.6	Up	0.104969	0.796176	Ras-related protein Rab-6A OS=Mus musculus GN=Rab6a PE=1 SV=4
Mrpl49	Q9CQ40	2	2	NA	109798.5	127828	113294	73533.5	134609	126043	113226	145700.5	79716	0.424667	0.371951	106113.5	119858.9	Up	0.175729	0.796795	39S ribosomal protein L49, mitochondrial OS=Mus musculus GN=Mrpl49 PE=2 SV=1
Arhgdia	Q99PT1	3	3	20206.67	11886.33	11786	11129	12138.33	13713.33	9892.67	12453.67	12655.67	10786.67	0.441073	0.35549	13429.27	11900.4	Down	-0.17437	0.808634	Rho GDP-dissociation inhibitor 1 OS=Mus musculus GN=Arhgdia PE=1 SV=3
Rap1gap	A2ALS5-3	1	1	822608	365612	547719	334727	471723	572118	462186	512736	333240	225477	0.442313	0.35427	508477.8	421151.4	Down	-0.27185	0.810328	Isoform 3 of Rap1 GTPase-activating protein 1 OS=Mus musculus GN=Rap1gap
Pitpnm3	Q3UHE1	1	1	392636	279941	335973	246167	273526	428857	350629	376567	290097	253132	0.425539	0.371071	305648.6	339856.4	Up	0.153051	0.797383	Membrane-associated phosphatidylinositol transfer protein 3 OS=Mus musculus GN=Pitpnm3 PE=2 SV=2
Cct2	P80314	15	15	387333.8	296515.5	333434.9	342938.6	316289.2	323289.1	341647.2	343410.2	404933.4	349232.5	0.427184	0.369385	335300.4	352502.5	Up	0.072171	0.798011	T-complex protein 1 subunit beta OS=Mus musculus GN=Cct2 PE=1 SV=4
Flot1	O08917	10	10	60163.2	101032.9	82957.8	90726.8	79340.9	96336.2	84824.6	92520	76655.3	96917.7	0.427903	0.368655	82844.32	89450.76	Up	0.110691	0.798181	Flotillin-1 OS=Mus musculus GN=Flot1 PE=1 SV=1
Tsg101	Q61187	1	1	178252	174228	208217	223911	184257	209916	219685	208801	191724	186639	0.427944	0.368613	193773	203353	Up	0.069619	0.798181	Tumor susceptibility gene 101 protein OS=Mus musculus GN=Tsg101 PE=1 SV=2
Api5	O35841	1	1	326593	170735	239744	219540	283219	182563	236156	256798	227009	216525	0.444561	0.352069	247966.2	223810.2	Down	-0.14787	0.810783	Apoptosis inhibitor 5 OS=Mus musculus GN=Api5 PE=1 SV=2
Acp1	Q9D358	2	2	51187	111579.5	85436.5	88181.5	86943.5	97651.5	76943	116141.5	84572.5	97259	0.428791	0.367754	84665.6	94513.5	Up	0.158744	0.798603	Low molecular weight phosphotyrosine protein phosphatase OS=Mus musculus GN=Acp1 PE=1 SV=3
Tufm	Q8BFR5	14	14	351877.6	300884.9	291514.2	337626.3	299620.1	372390.7	304043.8	321763.2	340308	313631.7	0.429539	0.366997	316304.6	330427.5	Up	0.063019	0.798603	Elongation factor Tu, mitochondrial OS=Mus musculus GN=Tufm PE=1 SV=1
Pkp4	Q68FH0	5	5	38243.6	88602	103858	87182.2	104311.8	78504	91207	110525.2	86681.8	114038.6	0.429619	0.366917	84439.52	96191.32	Up	0.187988	0.798603	Plakophilin-4 OS=Mus musculus GN=Pkp4 PE=1 SV=1
Lzic	Q8K3C3	1	1	456905	435520	528660	372515	438457	552473	528217	487499	417233	406481	0.429728	0.366806	446411.4	478380.6	Up	0.099785	0.798603	Protein LZIC OS=Mus musculus GN=Lzic PE=1 SV=1
Pitrm1	Q8K411-3	3	3	71938	101674	100587	106059.3	87499.67	104473	74678.33	110435.7	96521	124979.7	0.430111	0.366419	93551.6	102217.5	Up	0.127808	0.798603	Isoform 3 of Presequence protease, mitochondrial OS=Mus musculus GN=Pitrm1
Snx1	Q9WV80	2	2	142487.5	79804	114171.5	86304	113552	123838	110256	96979	88461	48960.5	0.446894	0.349795	107263.8	93698.9	Down	-0.19506	0.810783	Sorting nexin-1 OS=Mus musculus GN=Snx1 PE=1 SV=1
Tbc1d5	Q80XQ2	1	1	NA	38471	76080	63162	59412	46106	31898	73535	70529	22783	0.446937	0.349754	59281.25	48970.2	Down	-0.27567	0.810783	TBC1 domain family member 5 OS=Mus musculus GN=Tbc1d5 PE=1 SV=2
Dock4	P59764	3	3	NA	8652.33	3947	7468.67	6896.33	1730.33	7473	9367.67	10279.67	14869	0.430305	0.366224	6741.083	8743.934	Up	0.375302	0.798603	Dedicator of cytokinesis protein 4 OS=Mus musculus GN=Dock4 PE=1 SV=1
Gad1	P48318	5	5	16847.8	25983.6	38172	30766.8	40051.2	13449.4	36280.8	31590.6	29089.6	17119.6	0.447108	0.349588	30364.28	25506	Down	-0.25154	0.810783	Glutamate decarboxylase 1 OS=Mus musculus GN=Gad1 PE=2 SV=2
Ngly1	Q9JI78	1	1	70713	157290	110007	117002	121785	136799	109489	136999	122908	133672	0.432044	0.364472	115359.4	127973.4	Up	0.149708	0.800093	Peptide-N(4)-(N-acetyl-beta-glucosaminyl)asparagine amidase OS=Mus musculus GN=Ngly1 PE=1 SV=2
Slc12a9	Q99MR3	1	1	NA	29587	83455	66866	23155	20036	37486	40914	57702	31489	0.448319	0.348413	50765.75	37525.4	Down	-0.43599	0.811286	Solute carrier family 12 member 9 OS=Mus musculus GN=Slc12a9 PE=1 SV=2
Por	P37040	3	3	61841.5	108026.5	113824	112285.5	90393	115650	100747.5	98130	116264.5	100473	0.432497	0.364017	97274.1	106253	Up	0.127376	0.800353	NADPH--cytochrome P450 reductase OS=Mus musculus GN=Por PE=1 SV=2
Rpl13	P47963	2	2	241201.5	104280	103118	86092.5	149443	187248	81909	126634.5	87416.5	56339.5	0.448534	0.348205	136827	107909.5	Down	-0.34253	0.811286	60S ribosomal protein L13 OS=Mus musculus GN=Rpl13 PE=2 SV=3
Mlip	Q5FW52	3	3	162622.7	139760	134348.3	132338.7	163262.7	150508	130243.3	150697.7	139460.7	127948	0.449199	0.347561	146466.5	139771.5	Down	-0.0675	0.811422	Muscular LMNA-interacting protein OS=Mus musculus GN=Mlip PE=1 SV=1
Ndufv2	Q9D6J6-2	2	2	141653.5	300183.5	330441	340500.5	387640.5	269908	311439	317418	233238.5	166381.5	0.449433	0.347335	300083.8	259677	Down	-0.20865	0.811422	Isoform 2 of NADH dehydrogenase [ubiquinone] flavoprotein 2, mitochondrial OS=Mus musculus GN=Ndufv2
Ap3d1	O54774	2	2	95789	419371	490329.5	414558.5	534920	499704	444176.5	481042	446752.5	422427.5	0.432998	0.363514	390993.6	458820.5	Up	0.230785	0.800453	AP-3 complex subunit delta-1 OS=Mus musculus GN=Ap3d1 PE=1 SV=1

Akap5	D3YVF0	6	6	37159.4	46837	29947.2	41533.4	43843.2	47853.6	39507.2	33984.4	41677.6	56203.8	0.433183	0.363329	39864.04	43845.32	Up	0.137335	0.800453	A-kinase anchor protein 5 OS=Mus musculus GN=Akap5 PE=1 SV=2
Cadm1	Q8R5M8-3	6	6	55936.6	148836.8	205234.8	167969.2	179556.2	148118.2	159633.2	175242.6	192969	185608.6	0.433611	0.3629	151506.7	174354.3	Up	0.20264	0.800453	Isoform 3 of Cell adhesion molecule 1 OS=Mus musculus GN=Cadm1
Rbmx	Q9WV02-2	2	2	341887	430028.5	612960.5	443261	1036566	157944.5	605026.5	653258.5	583913.5	219060.5	0.450749	0.346065	572940.5	443840.7	Down	-0.36834	0.811422	Isoform 2 of RNA-binding motif protein, X chromosome OS=Mus musculus GN=Rbmx
Spire1	Q5ZKF3-3	1	1	NA	21686	22290	19777	8212	14774	9840	17937	22060	8640	0.450958	0.345864	17991.25	14650.2	Down	-0.29638	0.811422	Isoform 3 of Protein spire homolog 1 OS=Mus musculus GN=Spire1
Rbmxl1	Q91VM5	3	3	223618.7	298477.3	407192.3	312488.7	668417	116685.7	405827.3	457239.3	382534.7	128199.3	0.451998	0.344863	382038.8	298097.3	Down	-0.35794	0.811422	RNA binding motif protein, X-linked-like-1 OS=Mus musculus GN=Rbmxl1 PE=1 SV=1
Osbpl1a	Q91XL9	1	1	NA	9488	7167	8063	6609	8614	7823	6136	16430	8249	0.434143	0.362367	7831.75	9450.4	Up	0.271041	0.80051	Oxysterol-binding protein-related protein 1 OS=Mus musculus GN=Osbpl1a PE=1 SV=2
Pgrmc2	Q80UU9	2	2	87425	96091	118816.5	118444	142695	105154	130573	154049.5	120915.5	106611	0.439202	0.357336	112694.3	123460.6	Up	0.131636	0.806935	Membrane-associated progesterone receptor component 2 OS=Mus musculus GN=Pgrmc2 PE=1 SV=2
Rtcb	Q99LF4	8	8	139496.3	129340.6	143645.7	116351.7	155595.4	166572.7	164987.4	161204.3	144781.6	103066.9	0.440117	0.356432	136885.9	148122.6	Up	0.113817	0.808037	tRNA-splicing ligase RtcB homolog OS=Mus musculus GN=Rtcb PE=2 SV=1
Bsn	O88737	68	68	162188.5	155499.4	135955.3	147905.8	166098.1	182441.2	146323.3	139592.1	127621.4	124917.4	0.45255	0.344333	153529.4	144179.1	Down	-0.09065	0.811422	Protein bassoon OS=Mus musculus GN=Bsn PE=1 SV=4
Anxa6	P14824	8	8	304736.8	191472.9	278907.5	279096.9	149920.4	233965.6	234182.4	201996	199464.1	208426.3	0.452725	0.344166	240826.9	215606.9	Down	-0.15959	0.811422	Annexin A6 OS=Mus musculus GN=Anxa6 PE=1 SV=3
Rtn3	Q9ES97	6	6	33438	93751.67	72485	71764.67	83291.67	70412	65052.67	90614	85544.33	90624.33	0.440671	0.355886	70946.2	80449.47	Up	0.181357	0.808475	Reticulon-3 OS=Mus musculus GN=Rtn3 PE=1 SV=2
Hbb-b1	P02088	14	14	1749147	1304590	1087734	1040472	946372.6	1364120	1212892	977778.8	1465334	2015217	0.443339	0.353264	1225663	1407069	Up	0.19913	0.810783	Hemoglobin subunit beta-1 OS=Mus musculus GN=Hbb-b1 PE=1 SV=2
Arfgap3	Q9D8S3	2	2	NA	49839	39380	46658	51786	37955.5	51199.5	49265.5	61090.5	53976	0.443356	0.353247	46915.75	50697.4	Up	0.111839	0.810783	ADP-ribosylation factor GTPase-activating protein 3 OS=Mus musculus GN=Arfgap3 PE=1 SV=2
Vsnl1	P62761	2	2	31963	40568	49778	46782	40990	28220	48005	35084	46822	32215	0.455242	0.341758	42016.2	38069.2	Down	-0.14232	0.813657	Visinin-like protein 1 OS=Mus musculus GN=Vsnl1 PE=1 SV=2
0610037L	Q8BHG2-3	1	1	411322	219980	374835	270381	370824	358293	322832	282312	272523	248489	0.455865	0.341164	329468.4	296889.2	Down	-0.15021	0.813768	Isoform 3 of UPF0587 protein C1orf123 homolog OS=Mus musculus
Psma4	Q9R1P0	1	1	10262	8372	5846	7372	5413	6704	4634	7931	7185	6713	0.456152	0.34089	7453	6633.4	Down	-0.16807	0.813768	Proteasome subunit alpha type-4 OS=Mus musculus GN=Psma4 PE=1 SV=1
Hbb-b2	P02089	12	12	2018214	1523061	1276674	1248421	1129534	1583652	1419325	1133072	1735939	2380663	0.443855	0.352759	1439181	1650530	Up	0.197681	0.810783	Hemoglobin subunit beta-2 OS=Mus musculus GN=Hbb-b2 PE=1 SV=2
Gfra2	O08842	1	1	994020	299046	338269	309843	494861	653078	331779	344593	330212	170289	0.456641	0.340425	487207.8	365990.2	Down	-0.41273	0.813815	GDNF family receptor alpha-2 OS=Mus musculus GN=Gfra2 PE=2 SV=2
Pcsk2	P21661	1	1	NA	50077	36868	29455	20286	20655	44031	23034	33850	17643	0.456917	0.340163	34171.5	27842.6	Down	-0.2955	0.813815	Neuroendocrine convertase 2 OS=Mus musculus GN=Pcsk2 PE=1 SV=1
Bsn	O88737-2	66	66	167059.6	161089.2	139950.8	152239.4	170608	188068.1	150800.2	143951.6	131344.3	129258.4	0.457322	0.339778	158189.4	148684.5	Down	-0.0894	0.813844	Isoform 2 of Protein bassoon OS=Mus musculus GN=Bsn
Lztfl1	Q9JHQ5	2	2	NA	30620	20161	8896	8984	NA	27947	35933	7983	22574	0.444066	0.352552	17165.25	23609.25	Up	0.459861	0.810783	Leucine zipper transcription factor-like protein 1 OS=Mus musculus GN=Lztfl1 PE=1 SV=1
Pnkd	Q69ZP3-4	1	1	64415	81825	97709	59001	95393	87114	72198	82056	53149	65123	0.459248	0.337953	79668.6	71928	Down	-0.14746	0.816264	Isoform 4 of Probable hydrolase PNKD OS=Mus musculus GN=Pnkd
Rbmx	Q9WV02	3	3	255074.3	295714.7	430340.7	317089.3	723984	135255.3	430306	464695.7	407252.3	152411.7	0.459986	0.337255	404440.6	317984.2	Down	-0.34697	0.817008	RNA-binding motif protein, X chromosome OS=Mus musculus GN=Rbmx PE=1 SV=1
Phf24	Q80TL4	5	5	548392.8	364194.3	432921.5	359426	415704.5	422737.5	405383	344372.3	427388.5	371546.8	0.460398	0.336867	424127.8	394285.6	Down	-0.10526	0.817174	Protein KIAA1045 OS=Mus musculus GN=Kiaa1045 PE=2 SV=2
Hist1h1d	P43277	14	14	332062.1	390162.4	637428.6	556524.3	1020594	263529.4	545819.9	568096.9	553850.9	476312.3	0.461707	0.335634	587354.2	481521.9	Down	-0.28663	0.818532	Histone H1.3 OS=Mus musculus GN=Hist1h1d PE=1 SV=2
Mat2b	Q99LB6	2	2	482374.5	343599.5	525309	478171	324576	323459.5	469333.5	474335	539469.5	590287.5	0.445116	0.351527	430806	479377	Up	0.154122	0.810783	Methionine adenosyltransferase 2 subunit beta OS=Mus musculus GN=Mat2b PE=2 SV=1
Ndufa9	Q9DC69	4	4	210430.5	283272.8	370813.8	369893.3	300063.5	336093.3	363089.8	317968.5	350105.3	299564.8	0.445379	0.35127	306894.8	333364.3	Up	0.119356	0.810783	NADH dehydrogenase [ubiquinone] 1 alpha subcomplex subunit 9, mitochondrial OS=Mus musculus GN=Ndufa9 PE=1 SV=2
Atp6v1a	P50516-2	15	15	567808.1	392544.3	404895.7	392415.7	445502.3	348213.4	394707.1	529182.9	381496.7	372372.7	0.464055	0.333431	440633.2	405194.6	Down	-0.12096	0.819329	Isoform 2 of V-type proton ATPase catalytic subunit A OS=Mus musculus GN=Atp6v1a
Cct6a	P80317	11	11	1097492	682375.8	674307.8	545278.4	735045.3	810915.8	690383	601978.5	650684.9	581005.5	0.464072	0.333415	746899.9	666993.5	Down	-0.16324	0.819329	T-complex protein 1 subunit zeta OS=Mus musculus GN=Cct6a PE=1 SV=3
Tcerg1	Q8CGF7	2	2	1123.5	27399	20922.5	27246.5	23166.5	2526.5	19214	45440.5	26311.5	44698.5	0.445602	0.351053	19971.6	27638.2	Up	0.468714	0.810783	Transcription elongation regulator 1 OS=Mus musculus GN=Tcerg1 PE=1 SV=2
Phgdh	Q61753	6	6	3733.5	15453.83	16303	18017	15515.83	12976.17	15915.83	19331.83	17274.83	14925.83	0.446288	0.350385	13804.63	16084.9	Up	0.220554	0.810783	D-3-phosphoglycerate dehydrogenase OS=Mus musculus GN=Phgdh PE=1 SV=3
Begain	Q68EF6	3	3	17356	17720	15393	15840	15368	16116	14551	17611	17176	21133	0.446386	0.350289	16335.4	17317.4	Up	0.084221	0.810783	Brain-enriched guanylate kinase-associated protein OS=Mus musculus GN=Begain PE=1 SV=2
Xkr6	E9Q6C8-3	1	1	44913	109031	89210	78579	97839	90415	99651	95328	79941	102258	0.447039	0.349655	83914.4	93518.6	Up	0.156335	0.810783	Isoform 3 of XK-related protein 6 OS=Mus musculus GN=Xkr6
C1qbp	O35658	3	3	NA	158016	77162	230342	NA	75230	120103	90119	180895	106585	0.464911	0.33263	155173.3	114586.4	Down	-0.43744	0.819329	Complement component 1 Q subcomponent-binding protein, mitochondrial OS=Mus musculus GN=C1qbp PE=1 SV=1
Atp6v1a	P50516	22	22	398515.4	289357.6	292646.8	285014.5	319726.3	258444.2	286505.7	374428.5	277764.9	274830	0.465129	0.332427	317052.1	294394.6	Down	-0.10697	0.819329	V-type proton ATPase catalytic subunit A OS=Mus musculus GN=Atp6v1a PE=1 SV=2
Shank3	Q4ACU6-7	13	13	98139.25	316882.3	168335.3	242403.8	196098.5	280746.6	193605.6	183206.7	260001.5	274640	0.447307	0.349394	204371.8	238440.1	Up	0.22243	0.810783	Isoform 6 of SH3 and multiple ankyrin repeat domains protein 3 OS=Mus musculus GN=Shank3
Fam131b	Q3TY60	1	1	36299	16799	20796	19052	30739	29779	17741	25465	17058	15846	0.466827	0.330844	24737	21177.8	Down	-0.22412	0.820165	Protein FAM131B OS=Mus musculus GN=Fam131b PE=1 SV=1
Arpc5l	Q9D898	5	5	794088.5	517972.5	673903.5	708445	581036	623058.8	672858	633331.5	628094.5	499889.8	0.466921	0.330757	655089.1	611446.5	Down	-0.09946	0.820165	Actin-related protein 2/3 complex subunit 5-like protein OS=Mus musculus GN=Arpc5l PE=1 SV=1
Ighg2a	P01864	1	1	462783	443863	355078	334344	438692	359586	366379	397658	391232	411298	0.467614	0.330112	406952	385230.6	Down	-0.07914	0.820165	Ig gamma-2A chain C region secreted form OS=Mus musculus PE=1 SV=1
Mpp6	Q9JLB0	3	3	276035.3	171069	223974.7	194784.3	181129.3	214501.7	238190.7	199279.3	281308	209318	0.448374	0.34836	209398.5	228519.5	Up	0.126066	0.811286	Isoform Alpha of MAGUK p55 subfamily member 6 OS=Mus musculus GN=Mpp6
Pfdn2	O70591	1	1	74372	123494	117897	97562	118206	131004	127212	103329	110045	103505	0.449965	0.346821	106306.2	115019	Up	0.113646	0.811422	Prefoldin subunit 2 OS=Mus musculus GN=Pfdn2 PE=2 SV=2
Ahcyl2	Q68FL4	2	2	271588	314471.5	524421.5	431109	306336.5	282600.5	406774	513575	438669	446847	0.450607	0.346202	369585.3	417693.1	Up	0.176536	0.811422	Putative adenosylhomocysteinase 3 OS=Mus musculus GN=Ahcyl2 PE=1 SV=1
Armcx3	Q8BHS6	1	1	NA	13930	12369	8761	38590	NA	47430	NA	26123	12559	0.45069	0.346122	18412.5	28704	Up	0.640566	0.811422	Armadillo repeat-containing X-linked protein 3 OS=Mus musculus GN=Armcx3 PE=1 SV=1
Ndufs5	Q99LY9	5	5	229726.6	370120.6	260888.2	341739	234899.4	309143.2	288211.2	313793.6	280558.8	380412.4	0.452389	0.344488	287474.8	314423.8	Up	0.129275	0.811422	NADH dehydrogenase [ubiquinone] iron-sulfur protein 5 OS=Mus musculus GN=Ndufs5 PE=1 SV=3
Shank3	Q4ACU6-8	12	12	104956.3	342943.2	181453.8	261736	211391.2	303514.7	208556.6	196918.5	280559	295961.6	0.452502	0.344379	220496.1	257102.1	Up	0.221588	0.811422	Isoform 7 of SH3 and multiple ankyrin repeat domains protein 3 OS=Mus musculus GN=Shank3
Mrps5	Q99N87	2	2	283910	261045.5	263136.5	299807.5	251144	337803	256768.5	216885	216860	236790	0.469825	0.328064	271808.7	253021.3	Down	-0.10333	0.820165	28S ribosomal protein S5, mitochondrial OS=Mus musculus GN=Mrps5 PE=2 SV=1
Opa1	P58281	17	17	51983	101427.4	89188.5	118756.4	85751.79	93591.36	94051.5	94757.36	105163.3	106188.7	0.452548	0.344335	89421.42	98750.44	Up	0.143167	0.811422	Dynamin-like 120 kDa protein, mitochondrial OS=Mus musculus GN=Opa1 PE=1 SV=1
Prdx6	O08709	8	8	138309.3	216331	254774.9	231713.4	237637.5	170048.4	272054.5	228604.5	279336.1	239659.6	0.453109	0.343797	215753.2	237940.6	Up	0.14122	0.811544	Peroxiredoxin-6 OS=Mus musculus GN=Prdx6 PE=1 SV=3
Plekha2	Q9ERS5	2	2	NA	15309	17047.5	15397	18289.5	56301.5	19339	7647.5	21524.5	13137.5	0.454186	0.342766	16510.75	23590	Up	0.51477	0.812732	Pleckstrin homology domain-containing family A member 2 OS=Mus musculus GN=Plekha2 PE=1 SV=1
Lasp1	Q61792	4	4	1407737	1162527	826532.5	1063958	989762.5	974683.5	992772	1040544	981362	1072744	0.470933	0.327041	1090103	1012421	Down	-0.10666	0.820165	LIM and SH3 domain protein 1 OS=Mus musculus GN=Lasp1 PE=1 SV=1
Plekhg5	Q66T02	3	3	167634.3	131192.3	159954.7	122968	164404.7	184360.7	132340.3	127657.3	130101.7	113964	0.471057	0.326927	149230.8	137684.8	Down	-0.11618	0.820165	Pleckstrin homology domain-containing family G member 5 OS=Mus musculus GN=Plekhg5 PE=1 SV=1
Cox6b1	P56391	5	5	39394.5	11060	18187	20381.25	30581.75	11863	17232.5	149002.8	40511.25	10924.25	0.454407	0.342555	23920.9	46086.75	Up	0.94608	0.812732	Cytochrome c oxidase subunit 6B1 OS=Mus musculus GN=Cox6b1 PE=1 SV=2
Ywhaq	P68254	6	6	1298339	1324542	1242790	1254402	1200333	1255519	1452087	1228546	1436713	1186430	0.456256	0.340791	1264081	1311859	Up	0.053524	0.813768	14-3-3 protein theta OS=Mus musculus GN=Ywhaq PE=1 SV=1
Rpl37a	P61514	3	3	440503.5	191837.5	163992	153122	242334.5	236795	197475.5	212706	166253	164443	0.471869	0.326179	238357.9	195534.5	Down	-0.28571	0.820488	60S ribosomal protein L37a OS=Mus musculus GN=Rpl37a PE=2 SV=2
Fam126b	Q8C729	2	2	308093	101860	160352.5	102908	184885.5	155801	155358.5	136107	156846	100656.5	0.472734	0.325383	171619.8	140953.8	Down	-0.28399	0.820823	Protein FAM126B OS=Mus musculus GN=Fam126b PE=2 SV=1
Ctnna1	P26231	1	1	NA	12852	10765	13166	13156	5638	14439	14638	10754	10165	0.472971	0.325165	12484.75	11126.8	Down	-0.16613	0.820823	Catenin alpha-1 OS=Mus musculus GN=Ctnna1 PE=1 SV=1
Aftph	Q80WT5	1	1	456297	476148	479864	499827	545527	701533	512082	445942	601665	416666	0.457569	0.339543	491532.6	535577.6	Up	0.123809	0.813844	Aftiphilin OS=Mus musculus GN=Aftph PE=1 SV=2
Mgea5	Q9EQQ9	1	1	NA	150568	173382	179661	126131	142359	164765	190207	159615	190350	0.461801	0.335545	157435.5	169459.2	Up	0.106177	0.818532	Bifunctional protein NCOAT OS=Mus musculus GN=Mgea5 PE=1 SV=2
Dnajb14	Q149L6	1	1	99266	120081	114848	80745	117499	144662	113097	121117	90631	107445	0.463181	0.334249	106487.8	115390.4	Up	0.115835	0.819329	DnaJ homolog subfamily B member 14 OS=Mus musculus GN=Dnajb14 PE=2 SV=1
Timm22	Q9CQ85	1	1	NA	32210	23739	21806	9253	25039	30912	7685	10423	9386	0.474345	0.323906	21752	16689	Down	-0.38225	0.821451	Mitochondrial import inner membrane translocase subunit Tim22 OS=Mus musculus GN=Timm22 PE=2 SV=1
Speg	Q62407-4	1	1	NA	9727	6567	12828	7233	6590	13448	5884	13235	15778	0.464147	0.333344	9088.75	10987	Up	0.273644	0.819329	Isoform 4 of Striated muscle-specific serine/threonine-protein kinase OS=Mus musculus GN=Speg
Immt	Q8CAQ8-4	4	4	171996.8	314348	171313.8	270724.5	191317	233745.5	228534.8	199873.5	261481	341651.8	0.464265	0.333234	223940	253057.3	Up	0.176352	0.819329	Isoform 4 of Mitochondrial inner membrane protein OS=Mus musculus GN=Immt
Grk2	Q99MK8	4	4	NA	39694	46350.67	59177.33	38271.33	40447.67	50790	35025.67	65761.33	69423.67	0.464646	0.332878	45873.33	52289.67	Up	0.18887	0.819329	Beta-adrenergic receptor kinase 1 OS=Mus musculus GN=Adrbk1 PE=1 SV=2
Clpp	O88696	1	1	NA	54295	96820	90069	46546	67516	95850	169767	31913	95784	0.464702	0.332825	71932.5	92166	Up	0.357591	0.819329	Putative ATP-dependent Clp protease proteolytic subunit, mitochondrial OS=Mus musculus GN=Clpp PE=1 SV=1
Cstb	Q62426	3	3	55894.33	237310	140477.3	157286	157306.7	157601	171893.7	190312	154310	193159	0.466004	0.33161	149654.9	173455.1	Up	0.212923	0.820165	Cystatin-B OS=Mus musculus GN=Cstb PE=1 SV=1
Msi1	Q61474	4	4	9489.5	20314.5	13275.5	13949.25	16175.75	14031	19009.75	20087.25	13171	15488.75	0.467636	0.330092	14640.9	16357.55	Up	0.159952	0.820165	RNA-binding protein Musashi homolog 1 OS=Mus musculus GN=Msi1 PE=1 SV=1
Lzts3	A2AHG0-3	1	1	92809	75034	76873	55512	85068	NA	73587	68807	84109	56107	0.477185	0.321313	77059.2	70652.5	Down	-0.12523	0.821833	Isoform 3 of Leucine zipper putative tumor suppressor 3 OS=Mus musculus GN=Lzts3
Esd	Q9R0P3	5	5	292717.2	123796.4	124612.6	133850.2	159992.6	165527.4	181782	129965.4	286666.4	231679.8	0.468426	0.329359	166993.8	199124.2	Up	0.253874	0.820165	S-formylglutathione hydrolase OS=Mus musculus GN=Esd PE=1 SV=1
Pcyox1	Q9CQF9	2	2	NA	11673.5	19004	15502	9709	8556	18312.5	13030.5	26082.5	17629	0.468561	0.329234	13972.13	16722.1	Up	0.259205	0.820165	Prenylcysteine oxidase OS=Mus musculus GN=Pcyox1 PE=1 SV=1
Mrpl12	Q9DB15	1	1	236781	150945	140615	172677	157963	223322	117623	154972	143941	124442	0.477772	0.320779	171796.2	152860	Down	-0.16849	0.821833	39S ribosomal protein L12, mitochondrial OS=Mus musculus GN=Mrpl12 PE=1 SV=2
Napa	Q9DB05	4	4	53663	112769.3	95668	126465.5	118022	140244	103531.5	120402.8	101620.3	98830	0.468871	0.328947	101317.6	112925.7	Up	0.15649	0.820165	Alpha-soluble NSF attachment protein OS=Mus musculus GN=Napa PE=1 SV=1
Ank2	Q8C8R3-3	13	13	52225.33	61679	75650.89	61847	73808.89	70981.22	77186.33	62071.56	69852.78	64578.78	0.469356	0.328498	65042.22	68934.13	Up	0.083842	0.820165	Isoform 3 of Ankyrin-2 OS=Mus musculus GN=Ank2
Lrrc57	Q9D1G5	2	2	277478	220374	150576	219883	123482	210637	208778	194149	253215	240276	0.469357	0.328497	198358.6	221411	Up	0.158616	0.820165	Leucine-rich repeat-containing protein 57 OS=Mus musculus GN=Lrrc57 PE=2 SV=1
Coq6	Q8R1S0	6	6	95495.33	162964.3	146211.7	149161.7	118947	146171.7	154961.7	129267.7	151748.5	140983.8	0.470089	0.32782	134556	144626.7	Up	0.104127	0.820165	Ubiquinone biosynthesis monooxygenase COQ6 OS=Mus musculus GN=Coq6 PE=1 SV=3
Ckmt1	P30275	80	80	1045324	1006074	919508.4	955647.6	775407.4	1091207	782434.5	745067.6	885305.4	914626.3	0.480811	0.318026	940392.3	883728.1	Down	-0.08966	0.822347	Creatine kinase U-type, mitochondrial OS=Mus musculus GN=Ckmt1 PE=1 SV=1
Rps16	P14131	4	4	504149	197894.3	168282.3	163662.5	259168.5	209636.3	189121	222647.8	208932.5	214954.8	0.480838	0.318001	258631.3	209058.5	Down	-0.30699	0.822347	40S ribosomal protein S16 OS=Mus musculus GN=Rps16 PE=2 SV=4
Stx3	Q64704-4	1	1	NA	132355	94751	91633	82016	99138	105271	98647	108193	142696	0.470844	0.327123	100188.8	110789	Up	0.145094	0.820165	Isoform 3D of Syntaxin-3 OS=Mus musculus GN=Stx3
Dclk1	Q9JLM8	12	12	127648.1	162334.3	236206.1	199110.3	264114	264300.6	240368.1	184170	229103.1	183175.3	0.470924	0.327049	197882.5	220223.4	Up	0.154324	0.820165	Serine/threonine-protein kinase DCLK1 OS=Mus musculus GN=Dclk1 PE=1 SV=1
Ppp1r12a	Q9DBR7	4	4	128695.3	236943	174791.3	187002.3	161099.3	222047.5	207432.5	200152.3	174058.5	164002	0.471214	0.326782	177706.2	193538.6	Up	0.123127	0.820165	Protein phosphatase 1 regulatory subunit 12A OS=Mus musculus GN=Ppp1r12a PE=1 SV=2
Pfdn5	Q9WU28	2	2	2239194	1116616	1191117	1108865	1313652	1504871	1221902	1235213	1178216	952997	0.48183	0.317106	1393889	1218640	Down	-0.19384	0.822347	Prefoldin subunit 5 OS=Mus musculus GN=Pfdn5 PE=1 SV=1
Jam2	Q9JI59	1	1	NA	55109	45777	40706	51174	24287	46622	68236	73451	65756	0.471363	0.326645	48191.5	55670.4	Up	0.208132	0.820165	Junctional adhesion molecule B OS=Mus musculus GN=Jam2 PE=1 SV=1
Traf3	Q60803	1	1	112898	255304	226984	300223	254040	211601	199223	263187	282377	349402	0.473022	0.325119	229889.8	261158	Up	0.18398	0.820823	TNF receptor-associated factor 3 OS=Mus musculus GN=Traf3 PE=1 SV=2
Stx1b	P61264	15	15	50056.08	165615.2	119079.3	138696.5	113094.5	119137.8	144519.5	149219.4	113174.8	139543.5	0.473624	0.324566	117308.3	133119	Up	0.182411	0.820899	Syntaxin-1B OS=Mus musculus GN=Stx1b PE=1 SV=1
Vcpip1	Q8CDG3-2	1	1	NA	52466	43906	46152	43776	54070	52018	43489	57323	40446	0.473706	0.324491	46575	49469.2	Up	0.086975	0.820899	Isoform 2 of Deubiquitinating protein VCIP135 OS=Mus musculus GN=Vcpip1
Hmgb1	P63158	7	7	340623.9	316057.6	459706	413328.7	540846	298164.7	450013.4	348875	453633.6	328830.7	0.483008	0.316046	414112.4	375903.5	Down	-0.13966	0.822557	High mobility group protein B1 OS=Mus musculus GN=Hmgb1 PE=1 SV=2
Pmpca	Q9DC61	2	2	236072	253196	251684	350651.5	265732.5	282572.5	238120	245668.5	230357.5	275434.5	0.4842	0.314975	271467.2	254430.6	Down	-0.09351	0.823731	Mitochondrial-processing peptidase subunit alpha OS=Mus musculus GN=Pmpca PE=1 SV=1
Dclk1	Q9JLM8-2	9	9	145883.6	185524.9	269502.1	226658.3	301844.6	301045.1	274119.1	210480	261397.3	208523.9	0.475003	0.323304	225882.7	251113.1	Up	0.152764	0.821666	Isoform 2 of Serine/threonine-protein kinase DCLK1 OS=Mus musculus GN=Dclk1
Stau2	Q8CJ67-3	1	1	912929	421025	282743	419852	488012	583509	369760	375171	414663	347339	0.484721	0.314508	504912.2	418088.4	Down	-0.27222	0.823834	Isoform 3 of Double-stranded RNA-binding protein Staufen homolog 2 OS=Mus musculus GN=Stau2
Cpt1a	P97742	1	1	NA	432900	416988	363644	296884	350341	349385	265993	367920	410710	0.485618	0.313705	377604	348869.8	Down	-0.11419	0.824811	Carnitine O-palmitoyltransferase 1, liver isoform OS=Mus musculus GN=Cpt1a PE=1 SV=4
Acin1	Q9JIX8-3	1	1	69901	69431	62660	64434	120846	36015	85642	84134	68178	61453	0.486412	0.312996	77454.4	67084.4	Down	-0.20737	0.824942	Isoform 3 of Apoptotic chromatin condensation inducer in the nucleus OS=Mus musculus GN=Acin1
Arvcf	P98203-3	5	5	113812.8	122352.8	129046.5	114836.3	140278.8	134690.8	155712.5	144571.5	115908.8	107888.3	0.475111	0.323205	124065.4	131754.4	Up	0.08675	0.821666	Isoform 3 of Armadillo repeat protein deleted in velo-cardio-facial syndrome homolog OS=Mus musculus GN=Arvcf
Rtn1	Q8K0T0	4	4	NA	74451	101476.7	64614.67	93070.33	43409	52953	111722	74025.67	81266.33	0.487433	0.312085	83403.17	72675.2	Down	-0.19864	0.824942	Reticulon-1 OS=Mus musculus GN=Rtn1 PE=1 SV=1
Acss1	Q99NB1	2	2	NA	NA	4703	8144	NA	NA	41823	7695	NA		0.475948	0.32244	6423.5	24759	Up	1.946522	0.821833	Acetyl-coenzyme A synthetase 2-like, mitochondrial OS=Mus musculus GN=Acss1 PE=1 SV=1
Syt17	Q920M7	1	1	58709	255191	158691	219943	237989	167130	159300	184791	123635	155965	0.488353	0.311266	186104.6	158164.2	Down	-0.23469	0.824942	Synaptotagmin-17 OS=Mus musculus GN=Syt17 PE=2 SV=1
Trap1	Q9CQN1	3	3	NA	6248	2132	1458.5	NA	NA	2893	8011.5	7133	1728.5	0.476034	0.322362	3279.5	4941.5	Up	0.591473	0.821833	Heat shock protein 75 kDa, mitochondrial OS=Mus musculus GN=Trap1 PE=1 SV=1
Cnnm3	Q32NY4	1	1	102011	41627	87536	64348	106997	89869	56004	103377	47584	43448	0.488553	0.311088	80503.8	68056.4	Down	-0.24233	0.824942	Metal transporter CNNM3 OS=Mus musculus GN=Cnnm3 PE=1 SV=2
H2afx	P27661	6	6	316051.2	311446	401762	483533	629825.8	244227	411335.2	428153.8	413979.8	393424.2	0.488568	0.311075	428523.6	378224	Down	-0.18013	0.824942	Histone H2AX OS=Mus musculus GN=H2afx PE=1 SV=2
Eppk1	Q8R0W0	2	2	9569	23304	28207	26249	35976	32369	27015	26981	24867	29474	0.476723	0.321734	24661	28141.2	Up	0.190453	0.821833	Epiplakin OS=Mus musculus GN=Eppk1 PE=1 SV=2
Cmpk1	Q9DBP5	4	4	102460.3	86843	93118.75	66627.25	113406.3	106554.3	85446.75	89313.5	72793	71543	0.488925	0.310758	92491.1	85130.1	Down	-0.11965	0.824961	UMP-CMP kinase OS=Mus musculus GN=Cmpk1 PE=1 SV=1
Hspa4l	P48722	8	8	157820.4	106829.4	142054.9	128684	121206.4	134920.5	123544.1	139998.5	102307	115790.6	0.490234	0.309597	131319	123312.1	Down	-0.09076	0.826625	Heat shock 70 kDa protein 4L OS=Mus musculus GN=Hspa4l PE=1 SV=2
Cdk16	Q04735	1	1	234000	221946	231478	216099	220893	208058	224042	267009	204400	285911	0.476834	0.321633	224883.2	237884	Up	0.081082	0.821833	Cyclin-dependent kinase 16 OS=Mus musculus GN=Cdk16 PE=1 SV=1
Flna	Q8BTM8	1	1	58325	40623	37014	37194	51095	44532	41242	45073	32151	43518	0.491456	0.308515	44850.2	41303.2	Down	-0.11886	0.827136	Filamin-A OS=Mus musculus GN=Flna PE=1 SV=5
9-Sep	Q80UG5	4	4	327991.3	128488.8	146031.5	135756.8	164083	140719.3	162431	152979	149171.8	155346	0.491505	0.308472	180470.3	152129.4	Down	-0.24646	0.827136	Septin-9 OS=Mus musculus GN=Sept9 PE=1 SV=1
Gstm2	P15626	2	2	121890	191999	176991	206681.5	112200.5	173322	161042.5	153641	189339	215179	0.477283	0.321224	161952.4	178504.7	Up	0.140392	0.821833	Glutathione S-transferase Mu 2 OS=Mus musculus GN=Gstm2 PE=1 SV=2
Akr1a1	Q9JII6	4	4	276771	189415.5	208905	204014.8	285984	249051	247908.5	209822.5	188291.8	180924.8	0.492998	0.307155	233018.1	215199.7	Down	-0.11477	0.82802	Alcohol dehydrogenase [NADP(+)] OS=Mus musculus GN=Akr1a1 PE=1 SV=3
Larp1	Q6ZQ58	2	2	36143.5	36165.5	44911.5	32376	45261	53567	39952.5	42068.5	44347	31428	0.477636	0.320903	38971.5	42272.6	Up	0.117303	0.821833	La-related protein 1 OS=Mus musculus GN=Larp1 PE=1 SV=2
Glrx5	Q80Y14	2	2	224277.5	433472.5	225282	377070.5	179873.5	344750	318350.5	352242.5	303658.5	315178.5	0.479992	0.318766	287995.2	326836	Up	0.182522	0.822347	Glutaredoxin-related protein 5, mitochondrial OS=Mus musculus GN=Glrx5 PE=1 SV=2
C330018D	Q9CWB7	1	1	48703	95840	86684	87561	71871	104177	80287	71482	77880	94978	0.48	0.318759	78131.8	85760.8	Up	0.134409	0.822347	Glutaredoxin-like protein C5orf63 homolog OS=Mus musculus PE=1 SV=1
Gfer	P56213	2	2	207455.5	243776.5	273109.5	262619	282172	308550.5	237212	263778	260222.5	264527	0.480052	0.318712	253826.5	266858	Up	0.07223	0.822347	FAD-linked sulfhydryl oxidase ALR OS=Mus musculus GN=Gfer PE=2 SV=2
Bcas3	Q8CCN5	1	1	NA	6069	4808	10831	6210	NA	5343	NA	6407	6070	0.494792	0.305577	6979.5	5940	Down	-0.23266	0.82802	Breast carcinoma-amplified sequence 3 homolog OS=Mus musculus GN=Bcas3 PE=1 SV=2
Tanc1	Q0VGY8	1	1	218246	163102	81657	136894	120477	161917	130229	116094	95672	124635	0.494823	0.30555	144075.2	125709.4	Down	-0.19673	0.82802	Protein TANC1 OS=Mus musculus GN=Tanc1 PE=2 SV=2
Arap1	Q4LDD4-3	1	1	88143	36987	28039	83394	27110	42284	72761	60070	62792	83103	0.480454	0.318348	52734.6	64202	Up	0.283868	0.822347	Isoform 3 of Arf-GAP with Rho-GAP domain, ANK repeat and PH domain-containing protein 1 OS=Mus musculus GN=Arap1
Pfn2	Q9JJV2	5	5	1184262	972257	717098.8	824079.4	902039	977477.2	834766.6	771430	824754.6	878241.2	0.495261	0.305166	919947.2	857333.9	Down	-0.10169	0.82802	Profilin-2 OS=Mus musculus GN=Pfn2 PE=1 SV=3
Aldh1l2	Q8K009	2	2	540530.5	388882	498620	518711	446431.5	507391.5	481228.5	449041	431811	406400.5	0.49584	0.304658	478635	455174.5	Down	-0.07251	0.828448	Mitochondrial 10-formyltetrahydrofolate dehydrogenase OS=Mus musculus GN=Aldh1l2 PE=1 SV=2
Snrpn	P63163	1	1	NA	NA	8281	4359	NA	14023	NA	NA	6455	NA	0.481215	0.317661	6320	10239	Up	0.696078	0.822347	Small nuclear ribonucleoprotein-associated protein N OS=Mus musculus GN=Snrpn PE=2 SV=1
Sirt2	Q8VDQ8	2	2	28785.5	99800.5	96811.5	115056.5	72148.5	56285	78913.5	81175.5	52765	83514	0.496634	0.303964	82520.5	70530.6	Down	-0.2265	0.828694	NAD-dependent protein deacetylase sirtuin-2 OS=Mus musculus GN=Sirt2 PE=1 SV=2
Pfn2	Q9JJV2-3	4	4	1472151	1212814	892514.5	1024886	1123066	1213895	1040454	962097.8	1027976	1095330	0.497943	0.30282	1145086	1067950	Down	-0.10061	0.829871	Isoform 3 of Profilin-2 OS=Mus musculus GN=Pfn2
Vps13c	Q8BX70-3	3	3	NA	73604	109834	113013.3	128499	98118.33	116311	108517.7	78220	79582	0.497988	0.302781	106237.6	96149.8	Down	-0.14394	0.829871	Isoform 3 of Vacuolar protein sorting-associated protein 13C OS=Mus musculus GN=Vps13c
Gsta4	P24472	2	2	NA	103495	97184.5	119448	77640	146884.5	131208	99588	75736.5	100731.5	0.481531	0.317376	99441.88	110829.7	Up	0.156419	0.822347	Glutathione S-transferase A4 OS=Mus musculus GN=Gsta4 PE=1 SV=3
Dctn6	Q9WUB4	1	1	296457	176475	154581	171378	166827	195477	160034	176951	154022	179952	0.49923	0.301699	193143.6	173287.2	Down	-0.15651	0.830019	Dynactin subunit 6 OS=Mus musculus GN=Dctn6 PE=1 SV=1
Ndufv2	Q9D6J6	6	6	76198.75	167747	181360	188947.8	205302.5	149641	170095.8	173448	130087.3	102033.8	0.499403	0.301549	163911.2	145061.2	Down	-0.17625	0.830019	NADH dehydrogenase [ubiquinone] flavoprotein 2, mitochondrial OS=Mus musculus GN=Ndufv2 PE=1 SV=2
Slc25a22	Q9D6M3	14	14	51674.14	51805.36	75892.71	71913.5	88434	62597.86	70201.57	71273.93	60214.86	44853.36	0.499532	0.301437	67943.94	61828.32	Down	-0.13608	0.830019	Mitochondrial glutamate carrier 1 OS=Mus musculus GN=Slc25a22 PE=1 SV=1
Mrpl16	Q99N93	1	1	270441	315795	314533	384335	210718	312632	409220	306248	283608	317026	0.481802	0.317131	299164.4	325746.8	Up	0.122812	0.822347	39S ribosomal protein L16, mitochondrial OS=Mus musculus GN=Mrpl16 PE=2 SV=1
Ptgds	O09114	1	1	2223178	629242	829280	825679	1094156	977204	917667	908914	988407	746759	0.500817	0.300321	1120307	907790.2	Down	-0.30346	0.830338	Prostaglandin-H2 D-isomerase OS=Mus musculus GN=Ptgds PE=1 SV=1
Tnfrsf21	Q9EPU5	1	1	NA	89888	72102	104535	137468	131894	81377	111923	95973	154292	0.481876	0.317065	100998.3	115091.8	Up	0.188455	0.822347	Tumor necrosis factor receptor superfamily member 21 OS=Mus musculus GN=Tnfrsf21 PE=1 SV=2
Adh5	P28474	1	1	1016691	579303	654629	725683	717485	778069	617256	546630	810628	622484	0.501408	0.299809	738758.2	675013.4	Down	-0.13019	0.830338	Alcohol dehydrogenase class-3 OS=Mus musculus GN=Adh5 PE=1 SV=3
2-Mar	Q922Q1	2	2	NA	12363.5	16304.5	17168	21573.5	20185	14591.5	18570.5	12125.5	8856	0.501422	0.299797	16852.38	14865.7	Down	-0.18096	0.830338	MOSC domain-containing protein 2, mitochondrial OS=Mus musculus GN=Marc2 PE=1 SV=1
Ehd1	Q9WVK4	3	3	815005.7	1030812	927551.7	1015205	767947	888438.7	1091239	930301	973759.3	909111.3	0.481975	0.316975	911304.3	958569.9	Up	0.072951	0.822347	EH domain-containing protein 1 OS=Mus musculus GN=Ehd1 PE=1 SV=1
Ssbp1	Q9CYR0	4	4	36439.5	42627.25	43885.75	56444.5	75753.5	46659	62729	38499	41355.5	36064.25	0.502546	0.298824	51030.1	45061.35	Down	-0.17946	0.83079	Single-stranded DNA-binding protein, mitochondrial OS=Mus musculus GN=Ssbp1 PE=1 SV=1
Dpysl3	Q62188	26	26	813809.4	563545.2	537269.5	599795.4	576430.2	586076.5	559079.1	533859.3	605990.6	616560.5	0.502565	0.298808	618169.9	580313.2	Down	-0.09117	0.83079	Dihydropyrimidinase-related protein 3 OS=Mus musculus GN=Dpysl3 PE=1 SV=1
Ndufb9	Q9CQJ8	9	9	295036	227989.5	267014.2	236946.7	232529.8	297128	232005	237067.5	216771.5	204746.3	0.503013	0.298421	251903.2	237543.7	Down	-0.08468	0.83079	NADH dehydrogenase [ubiquinone] 1 beta subcomplex subunit 9 OS=Mus musculus GN=Ndufb9 PE=1 SV=3
Snap25	P60879	21	21	170152.9	133510.1	137972.6	138348.6	154477.1	157478.5	144409.1	150145	132505.8	114141.5	0.503222	0.29824	146892.3	139736	Down	-0.07205	0.83079	Synaptosomal-associated protein 25 OS=Mus musculus GN=Snap25 PE=1 SV=1
Rab5c	P35278	2	2	645850.5	526465	843282	847510.5	836947.5	850578.5	883571	817398	858649	598754	0.482242	0.316735	740011.1	801790.1	Up	0.115678	0.822347	Ras-related protein Rab-5C OS=Mus musculus GN=Rab5c PE=1 SV=2
Rpl7a	P12970	10	10	626420.6	541094.1	394949.3	402696.8	420131.9	560288.3	455999.1	548273.3	513560.7	493929.3	0.482586	0.316425	477058.5	514410.2	Up	0.108753	0.822385	60S ribosomal protein L7a OS=Mus musculus GN=Rpl7a PE=2 SV=2
Tmx2	Q9D710	1	1	NA	53557	61344	65275	32336	35811	58448	29730	62552	45005	0.506191	0.295686	53128	46309.2	Down	-0.19817	0.833319	Thioredoxin-related transmembrane protein 2 OS=Mus musculus GN=Tmx2 PE=2 SV=1
BC037034	Q3UTZ3	1	1	83276	89129	78486	80278	115289	122171	93004	91852	114359	68061	0.48434	0.31485	89291.6	97889.4	Up	0.132628	0.823731	Uncharacterized protein C7orf43 homolog OS=Mus musculus PE=2 SV=1
Gmfb	Q9CQI3	2	2	292902.5	325286	325370.5	322418.5	208861	306030.5	333629.5	342732.5	279817.5	305456.5	0.486769	0.312677	294967.7	313533.3	Up	0.088062	0.824942	Glia maturation factor beta OS=Mus musculus GN=Gmfb PE=1 SV=3
Rab10	P61027	2	2	16703.5	27630.5	31403	35139	22431	33489.5	37211.5	24166	25245.5	28160.5	0.487444	0.312075	26661.4	29654.6	Up	0.153503	0.824942	Ras-related protein Rab-10 OS=Mus musculus GN=Rab10 PE=1 SV=1
Eif5	P59325	2	2	283859	884122	507856	803603	553492.5	736389.5	712688.5	647922.5	607159.5	747857	0.488434	0.311194	606586.5	690403.4	Up	0.186726	0.824942	Eukaryotic translation initiation factor 5 OS=Mus musculus GN=Eif5 PE=1 SV=1
Ndufs4	Q9CXZ1	3	3	175904	73018.67	128284	150951.7	102534.7	134889	129140	100416	162850.7	188781	0.488592	0.311054	126138.6	143215.3	Up	0.183176	0.824942	NADH dehydrogenase [ubiquinone] iron-sulfur protein 4, mitochondrial OS=Mus musculus GN=Ndufs4 PE=1 SV=3
G3bp1	P97855	1	1	79911	73619	74543	69607	98760	81018	95523	67029	71742	48759	0.508281	0.293896	79288	72814.2	Down	-0.12288	0.834545	Ras GTPase-activating protein-binding protein 1 OS=Mus musculus GN=G3bp1 PE=1 SV=1
Rps13	P62301	4	4	1233009	801182.5	685969.8	648917.5	969816	1144570	900187.8	992098.5	865167.5	874684.8	0.491058	0.308867	867778.9	955341.7	Up	0.138689	0.827136	40S ribosomal protein S13 OS=Mus musculus GN=Rps13 PE=1 SV=2
Lemd3	Q9WU40	1	1	84166	125855	176218	238381	377324	53929	232809	167122	188489	153333	0.509717	0.292671	200388.8	159136.4	Down	-0.33254	0.835832	Inner nuclear membrane protein Man1 OS=Mus musculus GN=Lemd3 PE=1 SV=2
Sh3gl2	Q62420	11	11	626471.3	510702.3	433935.6	438982.9	404635.5	469002.5	457466.1	457357.2	486658.6	392689.9	0.510532	0.291977	482945.5	452634.9	Down	-0.09351	0.836632	Endophilin-A1 OS=Mus musculus GN=Sh3gl2 PE=1 SV=2
Srm	Q64674	3	3	562503	271382.7	333348.3	347346	374135.7	443340.7	298202.7	329715.3	299929.7	322817	0.512253	0.290515	377743.1	338801.1	Down	-0.15697	0.838226	Spermidine synthase OS=Mus musculus GN=Srm PE=2 SV=1
Pfkl	P12382	3	3	47393.67	31404.67	41961	48760.33	58070	37452.67	40149	47524	43745	42216.33	0.512451	0.290348	45517.93	42217.4	Down	-0.1086	0.838226	6-phosphofructokinase, liver type OS=Mus musculus GN=Pfkl PE=1 SV=4
Nlgn2	Q69ZK9	6	6	39884	48436.6	45576.4	44564.4	54100.6	44588	42163.4	50482.6	45037	40282	0.512485	0.290319	46512.4	44510.6	Down	-0.06347	0.838226	Neuroligin-2 OS=Mus musculus GN=Nlgn2 PE=1 SV=2
Stx12	Q9ER00	5	5	12475.5	57502	96313.5	90773.25	95495	103533.8	52765.25	108251.8	71290.5	86294.5	0.492198	0.30786	70511.85	84427.15	Up	0.259841	0.827757	Syntaxin-12 OS=Mus musculus GN=Stx12 PE=1 SV=1
Rpl27	P61358	2	2	533419	303437	239913.5	251148.5	233556	315174	297581	208453	300864	227032	0.514044	0.289	312294.8	269820.8	Down	-0.21091	0.839704	60S ribosomal protein L27 OS=Mus musculus GN=Rpl27 PE=1 SV=2
Uggt1	Q6P5E4	1	1	188213	284988	250596	180431	209192	187562	240818	237049	195036	169961	0.514887	0.288288	222684	206085.2	Down	-0.11176	0.839903	UDP-glucose:glycoprotein glucosyltransferase 1 OS=Mus musculus GN=Uggt1 PE=1 SV=4
Prkcg	P63318	8	8	282797.7	391011.3	379710.2	340387.7	353795.7	256392.5	343532.2	412857.5	325313.7	299979	0.514966	0.288221	349540.5	327615	Down	-0.09346	0.839903	Protein kinase C gamma type OS=Mus musculus GN=Prkcg PE=1 SV=1
Cbr3	Q8K354	8	8	490812.1	494061.6	478743.5	539938.3	452100.1	521528.3	513844	466408	405411.8	461713.6	0.515149	0.288067	491131.1	473781.1	Down	-0.05189	0.839903	Carbonyl reductase [NADPH] 3 OS=Mus musculus GN=Cbr3 PE=2 SV=1
Dab2ip	O3UHC7-4	4	4	233665.3	178567.3	157026	153245.7	196621.3	206689	185271	168904.3	143018.7	151565.3	0.515502	0.28777	183825.1	171089.7	Down	-0.10358	0.839943	Isoform 4 of Disabled homolog 2-interacting protein OS=Mus musculus GN=Dab2ip
Ubqln1	Q8R317	2	2	22513.5	20626.5	30053	27651.5	24788	14343.5	21181.5	22802	29549	27052	0.515886	0.287446	25126.5	22985.6	Down	-0.12848	0.840036	Ubiquilin-1 OS=Mus musculus GN=Ubqln1 PE=1 SV=1
Cpe	Q00493	4	4	1256432	250621.3	530317.3	390158	835594	624892	471175.5	581423.3	477800	464378.8	0.517072	0.286449	652624.6	523933.9	Down	-0.31687	0.840874	Carboxypeptidase E OS=Mus musculus GN=Cpe PE=1 SV=2
Pkp4	Q68FH0-3	1	1	NA	105620	87907	74293	108650	NA	30924	132616	41584	98848	0.517738	0.28589	94117.5	75993	Down	-0.3086	0.840874	Isoform 3 of Plakophilin-4 OS=Mus musculus GN=Pkp4
4-Sep	P28661-5	2	2	462446	323229.5	228353.5	267288	351240.5	288303.5	304928	258959	321828.5	312893.5	0.518059	0.285621	326511.5	297382.5	Down	-0.13481	0.840874	Isoform 5 of Septin-4 OS=Mus musculus GN=Sept4
Pcbp3	P57722	8	8	242158.8	218555.5	199522.2	174779	237780	205715	224265.7	211229.5	209659	169147	0.518071	0.285611	214559.1	204003.2	Down	-0.07278	0.840874	Poly(rC)-binding protein 3 OS=Mus musculus GN=Pcbp3 PE=2 SV=3

Gpr18	Q8K1Z6	1	1	156992	173801	139998	135067	186754	179632	107745	130926	144176	173773	0.518096	0.28559	158522.4	147250.4	Down	-0.10642	0.840874 N-arachidonyl glycine receptor OS=Mus musculus GN=Gpr18 PE=1 SV=1
Arl3	Q9WUL7	1	1	623516	619202	1138577	1108219	1096263	945227	850717	771399	969917	567253	0.518407	0.285329	917155.4	820902.6	Down	-0.15996	0.840874 ADP-ribosylation factor-like protein 3 OS=Mus musculus GN=Arl3 PE=1 SV=1
Txndc17	Q9CQM5	2	2	667110	675269.5	455923	522338.5	489832.5	568143	477416	439482	499625.5	632095.5	0.518815	0.284987	562094.7	523352.4	Down	-0.10303	0.840874 Thioredoxin domain-containing protein 17 OS=Mus musculus GN=Txndc17 PE=1 SV=1
Caskin1	Q6P9K8-3	24	24	129798.1	134577.8	138650.3	130976.5	188168.9	170281.5	141666.3	156775.5	141663.4	156692.1	0.493347	0.306848	144434.3	153415.8	Up	0.087033	0.82802 Isoform 3 of Caskin-1 OS=Mus musculus GN=Caskin1
Atp4a	Q64436	1	1	NA	2572527	2836549	3009926	2205188	2383427	2322235	2555887	2488379	2842721	0.519377	0.284517	2656048	2518530	Down	-0.0767	0.840874 Potassium-transporting ATPase alpha chain 1 OS=Mus musculus GN=Atp4a PE=1 SV=3
Uqcrfs1	Q9CR68	10	10	186917.6	129574.7	126396	176440	125718.1	149783.6	145833.3	121567.9	141517.7	136829.7	0.519682	0.284262	149009.3	139106.4	Down	-0.09921	0.840874 Cytochrome b-c1 complex subunit Rieske, mitochondrial OS=Mus musculus GN=Uqcrfs1 PE=1 SV=1
Hadhb	Q99JY0	7	7	127978.4	156910.4	157165.3	203575	170317.9	188437.6	164269.9	161686.7	166212	184611	0.493466	0.306743	163189.4	173043.4	Up	0.084587	0.82802 Trifunctional enzyme subunit beta, mitochondrial OS=Mus musculus GN=Hadhb PE=1 SV=1
Map4k4	P97820	4	4	22307	33519.5	36019.75	35887.25	41518.75	43443.25	44799.5	30654.5	37109.25	29314.5	0.494233	0.306068	33850.45	37064.2	Up	0.130851	0.82802 Mitogen-activated protein kinase kinase kinase kinase 4 OS=Mus musculus GN=Map4k4 PE=1 SV=1
Aak1	Q3UHJ0	12	12	85152.43	45803.71	62642.43	47569	66229.14	70415.57	53038	65669.71	50687.14	36220.14	0.521863	0.282443	61479.54	55206.11	Down	-0.15527	0.842119 AP2-associated protein kinase 1 OS=Mus musculus GN=Aak1 PE=1 SV=2
Gnb2	P62880	7	7	1777939	1832535	1915737	1788814	1918786	2135837	1697136	1874827	1834059	1984055	0.494461	0.305868	1846762	1905183	Up	0.044931	0.82802 Guanine nucleotide-binding protein G(I)/G(S)/G(T) subunit beta-2 OS=Mus musculus GN=Gnb2 PE=1 SV=3
Atp1b3	P97370	3	3	68330	144049.3	191104	195917	165331.7	195165	165711.7	182382.7	153476	157873.3	0.495182	0.305235	152946.4	170921.7	Up	0.16031	0.82802 Sodium/potassium-transporting ATPase subunit beta-3 OS=Mus musculus GN=Atp1b3 PE=1 SV=1
Tbc1d10a	P58802	1	1	142826	182757	161482	130526	175632	163110	164009	187036	148735	172088	0.496539	0.304047	158644.6	166995.6	Up	0.074012	0.828694 TBC1 domain family member 10A OS=Mus musculus GN=Tbc1d10a PE=2 SV=1
Mgll	O35678	3	3	27480	35755	33283	38530.5	21802	31869.5	39713	44701.5	35062	22569.5	0.498384	0.302436	31370.1	34783.1	Up	0.148996	0.829992 Monoglyceride lipase OS=Mus musculus GN=Mgll PE=1 SV=1
Erp29	P57759	5	5	1949010	1340389	1407299	984718	984900	1329450	1214730	1349676	1095503	1033735	0.523922	0.280733	1333263	1204618	Down	-0.14639	0.843476 Endoplasmic reticulum resident protein 29 OS=Mus musculus GN=Erp29 PE=1 SV=2
Fkbp2	P45878	5	5	61281.6	105727.8	90184.2	90631.2	95241.6	125862.8	103402.8	99974.8	80827.8	74468.8	0.499695	0.301295	88613.28	96907.4	Up	0.129084	0.830019 Peptidyl-prolyl cis-trans isomerase FKBP2 OS=Mus musculus GN=Fkbp2 PE=1 SV=1
Prkaa1	Q5EG47	1	1	NA	NA	11208	NA	9827	37977	10458	NA	NA	NA	0.501118	0.30006	10517.5	24217.5	Up	1.203258	0.830338 5'-AMP-activated protein kinase catalytic subunit alpha-1 OS=Mus musculus GN=Prkaa1 PE=1 SV=2
Atox1	O08997	2	2	439651	185267	234877	202037	249556	314449	234698	281712	176034	109583	0.526256	0.278803	262277.6	223295.2	Down	-0.23214	0.844503 Copper transport protein ATOX1 OS=Mus musculus GN=Atox1 PE=1 SV=1
Etl4	A2AQ25-5	4	4	247923.5	154482.5	153620	163519.8	190079.8	172324.5	158537.8	177317.5	179933.5	158692.5	0.526904	0.278269	181925.1	169361.2	Down	-0.10324	0.844503 Isoform 5 of Sickle tail protein OS=Mus musculus GN=Skt
Hprt	P00493	2	2	136884.5	156433	150424	151227	113435	113153.5	143608.5	141507.5	170548.5	197882.5	0.501507	0.299723	141680.7	153340.1	Up	0.114092	0.830338 Hypoxanthine-guanine phosphoribosyltransferase OS=Mus musculus GN=Hprt PE=1 SV=3
Cadm3	Q99N28	9	9	259048.3	503239.7	540539	487418	541923.8	581730.3	440783.5	464706.5	512883.3	541569.7	0.503401	0.298086	466433.8	508334.7	Up	0.124106	0.83079 Cell adhesion molecule 3 OS=Mus musculus GN=Cadm3 PE=1 SV=1
Snap91	Q61548-3	15	15	135843.4	242935.8	245018.1	249407.2	308846.1	298514	247240.1	238467.9	257077.6	247824.2	0.505274	0.296473	236410.1	257824.7	Up	0.125099	0.833319 Isoform 3 of Clathrin coat assembly protein AP180 OS=Mus musculus GN=Snap91
Nono	Q99K48-2	2	2	193242	251004	369826	271910	525432	257097	382488	427858	390726	383463	0.506206	0.295673	322282.8	368326.4	Up	0.192658	0.833319 Isoform 2 of Non-POU domain-containing octamer-binding protein OS=Mus musculus GN=Nono
Lrrfip2	Q91WK0	1	1	NA	53919	54128	45970	63748	28779	56808	58640	61323	42954	0.529113	0.276452	54441.25	49700.8	Down	-0.13143	0.844503 Leucine-rich repeat flightless-interacting protein 2 OS=Mus musculus GN=Lrrfip2 PE=1 SV=1
Myl6	Q60605	4	4	681054	774704.8	640621.8	717757.8	745618.3	706831.3	728113	797833.8	712779.5	715381.5	0.506603	0.295333	711951.3	732187.8	Up	0.040435	0.833319 Myosin light polypeptide 6 OS=Mus musculus GN=Myl6 PE=1 SV=3
Myo18a	Q9JMH9-4	7	7	297857.5	124754.5	177910.3	144008.8	298958.3	242913.8	171915.3	220178.8	140979.5	121396.5	0.529797	0.275891	208697.9	179476.8	Down	-0.21762	0.844503 Isoform 4 of Unconventional myosin-XVIIIa OS=Mus musculus GN=Myo18a
Rimbp2	Q80U40	2	2	93515	101501	72553	80673.5	104286.5	101520.5	91973	82459	87660	120235	0.506674	0.295271	90505.8	96769.5	Up	0.096542	0.833319 RIMS-binding protein 2 OS=Mus musculus GN=Rimbp2 PE=1 SV=3
Mrpl24	Q9CQ06	1	1	12326	14354	14978	17000	14298	16553	12861	13890	12756	13551	0.530071	0.275666	14591.2	13922.2	Down	-0.06771	0.844503 39S ribosomal protein L24, mitochondrial OS=Mus musculus GN=Mrpl24 PE=2 SV=1
Crabp1	P62965	1	1	373917	160650	196430	139489	246619	268476	184007	201812	144653	161810	0.530194	0.275565	223421	192151.6	Down	-0.21752	0.844503 Cellular retinoic acid-binding protein 1 OS=Mus musculus GN=Crabp1 PE=1 SV=2
Prrc2a	Q7TSC1	7	7	517238	185209	237775.9	199053.1	337033	346514.7	254817.6	236787.4	221251.6	192947.6	0.530199	0.27556	295261.8	250463.8	Down	-0.23739	0.844503 Protein PRRC2A OS=Mus musculus GN=Prrc2a PE=1 SV=1
Hnrnpa3	Q8BG05	9	9	38690.67	81454.5	117035.2	104908.2	130825.3	39502.67	130893.2	149950.3	130298.3	109628.5	0.506884	0.295091	94582.77	112054.6	Up	0.244553	0.833319 Heterogeneous nuclear ribonucleoprotein A3 OS=Mus musculus GN=Hnrnpa3 PE=1 SV=1
Eef2	P58252	20	20	247980.8	244973.8	249890.2	228096.7	258482.5	275526.9	239852.6	270718.3	249375	229553.5	0.507304	0.294732	245884.8	253005.2	Up	0.041185	0.833475 Elongation factor 2 OS=Mus musculus GN=Eef2 PE=1 SV=2
Tpbg	Q9Z0L0	1	1	256516	195805	118820	146239	61720	154910	95946	102778	151877	153542	0.530491	0.275322	155820	131810.6	Down	-0.24141	0.844503 Trophoblast glycoprotein OS=Mus musculus GN=Tpbg PE=1 SV=3
Elfn1	Q8C8T7	1	1	NA	27504	25798	41515	15365	29028	33676	18787	34842	45618	0.508951	0.293324	27545.5	32390.2	Up	0.233741	0.835109 Protein ELFN1 OS=Mus musculus GN=Elfn1 PE=2 SV=1
Skp1a	Q9WTX5	4	4	1093747	987754	772691	901089	687740	821712	873543	910744	761608	816273	0.531524	0.274477	888604.2	836776	Down	-0.0867	0.844564 S-phase kinase-associated protein 1 OS=Mus musculus GN=Skp1 PE=1 SV=3
Erbin	Q80TH2-2	2	2	7758	34575.5	38664.5	28175	43103.5	67088.5	51714	22155.5	17804	31906	0.514042	0.289001	30455.3	38133.6	Up	0.324369	0.839704 Isoform 2 of Protein LAP2 OS=Mus musculus GN=Erbb2ip
Hnrnpk	P61979-3	23	23	253511.5	177083.2	260905.2	204397.9	344181.8	198304.6	257881.6	245123.1	243780	189401.8	0.531848	0.274212	248015.9	226898.2	Down	-0.12839	0.844564 Isoform 3 of Heterogeneous nuclear ribonucleoprotein K OS=Mus musculus GN=Hnrnpk
Garnl3	Q3V0G7	2	2	NA	19996	15437.5	44650	35377.5	NA	23095	28407.5	27540.5	16483	0.532837	0.273406	28865.25	23881.5	Down	-0.27344	0.84561 GTPase-activating Rap/Ran-GAP domain-like protein 3 OS=Mus musculus GN=Garnl3 PE=1 SV=1
Pafah1b3	Q61205	1	1	98267	69350	90624	58797	62933	95433	86508	76313	74628	77066	0.519295	0.284586	75994.2	81989.6	Up	0.109552	0.840874 Platelet-activating factor acetylhydrolase IB subunit gamma OS=Mus musculus GN=Pafah1b3 PE=1 SV=1
Rpl21	O09167	5	5	394825.8	209232.8	187452.2	167958.6	279916.8	295386	210848.8	226778.8	200785.6	149388.6	0.534441	0.2721	247877.2	216637.6	Down	-0.19434	0.846591 60S ribosomal protein L21 OS=Mus musculus GN=Rpl21 PE=2 SV=3
Lin7c	O88952	7	7	1080134	514400	562376.5	491757	684053	758567.3	606303	628840	487480.3	451014.5	0.534958	0.27168	666544.1	586441	Down	-0.18471	0.846591 Protein lin-7 homolog C OS=Mus musculus GN=Lin7c PE=1 SV=2
Atp5f1	Q9CQQ7	2	2	61625	78165.5	106759.5	104683.5	68634.5	79840	97911.5	89008	91491.5	95402.5	0.520893	0.283251	83973.6	90730.7	Up	0.111655	0.842119 ATP synthase F(0) complex subunit B1, mitochondrial OS=Mus musculus GN=Atp5f1 PE=1 SV=1
Lyst	P97412	1	1	67605	248630	212641	200383	222751	246192	188077	253680	190865	192466	0.521673	0.282602	190402	214256	Up	0.170287	0.842119 Lysosomal-trafficking regulator OS=Mus musculus GN=Lyst PE=1 SV=1
Ttc9c	Q810A3	1	1	70660	103575	84163	81424	134979	112074	99152	98151	104448	101295	0.52204	0.282296	94960.2	103024	Up	0.117586	0.842119 Tetratricopeptide repeat protein 9C OS=Mus musculus GN=Ttc9c PE=2 SV=1
Eef1d	P57776	5	5	262241.6	215845.2	144959.4	182373.6	196650.4	216800.2	197708.8	206383.4	212641.6	239530.8	0.522094	0.282251	200414	214613	Up	0.098322	0.842119 Elongation factor 1-delta OS=Mus musculus GN=Eef1d PE=1 SV=3
Dcun1d1	Q9QZ73	1	1	NA	1560	2435	718		1209	NA	1297	1245	1069	0.537661	0.269491	1571	1205	Down	-0.38265	0.848489 DCN1-like protein 1 OS=Mus musculus GN=Dcun1d1 PE=2 SV=1
Plin3	Q9DBG5	2	2	NA	21715.5	25904	30336	15109.5	26042.5	18908.5	26926.5	28745.5	28464	0.522894	0.281586	23266.25	25817.4	Up	0.150105	0.842879 Perilipin-3 OS=Mus musculus GN=Plin3 PE=1 SV=1
Itpka	Q8R071	6	6	181720.3	426681.3	340392.3	465894.7	480165.3	386255	455244	457027	443192.7	354134	0.523742	0.280883	378970.8	419170.5	Up	0.145451	0.843476 Inositol-trisphosphate 3-kinase A OS=Mus musculus GN=Itpka PE=2 SV=1
Rp2	Q9EPK2-3	1	1	NA	NA	36584	34129	93005	NA	104031	44177	67575	66883	0.524539	0.280222	54572.67	70666.5	Up	0.372848	0.843938 Isoform 3 of Protein XRP2 OS=Mus musculus GN=Rp2
Fech	P22315	1	1	880452	749452	675264	883189	503115	758153	752249	656999	567195	698644	0.538874	0.268513	738294.4	686648	Down	-0.10463	0.848595 Ferrochelatase, mitochondrial OS=Mus musculus GN=Fech PE=1 SV=2
Syn3	Q8JZP2	3	3	68968.67	46078.33	51145.67	39301.33	44564.33	50703.67	48847	46432	42196	44255	0.539032	0.268385	50011.67	46486.73	Down	-0.10545	0.848595 Synapsin-3 OS=Mus musculus GN=Syn3 PE=1 SV=2
Celf4	Q7TSY6-3	1	1	1241015	1251248	1026361	1196350	1103180	1419229	940408	1013617	965132	1158915	0.539353	0.268127	1163631	1099460	Down	-0.08184	0.848595 Isoform 3 of CUGBP Elav-like family member 4 OS=Mus musculus GN=Celf4
Nudc	O35685	1	1	NA	121037	85789	77244	57061	82189	69303	105813	104459	119225	0.52507	0.279783	85282.75	96197.8	Up	0.17375	0.844263 Nuclear migration protein nudC OS=Mus musculus GN=Nudc PE=1 SV=1
Rpl27a	P14115	3	3	276460.7	257339.3	212787.3	192049.7	280140.7	264269.7	231360.3	250148.3	190680.3	211922	0.541191	0.266649	243755.5	229676.1	Down	-0.08583	0.849697 60S ribosomal protein L27a OS=Mus musculus GN=Rpl27a PE=2 SV=5
D17H6S53	Q9Z1R4	1	1	249304	225163	314602	346932	321527	417069	298816	315611	279127	264356	0.527311	0.277933	291505.6	314995.8	Up	0.111809	0.844503 Uncharacterized protein C6orf47 homolog OS=Mus musculus GN=D17h6s53e PE=2 SV=1
Apc	Q61315-3	2	2	21967	52322.5	39197.5	40541.5	43178.5	52665	44282.5	45432	39793.5	34329	0.528074	0.277305	39441.4	43300.4	Up	0.13467	0.844503 Isoform 3 of Adenomatous polyposis coli protein OS=Mus musculus GN=Apc
Dmxl2	Q8BPN8	5	5	20806.75	38608.75	39373	45513.5	43023	33688.25	51439.75	46563.75	42289	32156.25	0.528634	0.276845	37465	41227.4	Up	0.13806	0.844503 DmX-like protein 2 OS=Mus musculus GN=Dmxl2 PE=1 SV=3
Sarnp	Q9D1J3	1	1	NA	64781	118343	54625	148928	NA	78930	149088	110950	119480	0.528999	0.276545	96669.25	114612	Up	0.245629	0.844503 SAP domain-containing ribonucleoprotein OS=Mus musculus GN=Sarnp PE=1 SV=3
Akap12	Q9WTQ5	2	2	114075	103626.5	160835.5	114282	127988	114887	138015	98153.5	125872.5	104784.5	0.543294	0.264965	124161.4	116342.5	Down	-0.09384	0.851138 A-kinase anchor protein 12 OS=Mus musculus GN=Akap12 PE=1 SV=1
Gigyf2	Q6Y7W8	2	2	168101.5	139188.5	104589	115495	143084	174063	146326	143248	144887.5	111078.5	0.529737	0.27594	134091.6	143920.6	Up	0.102054	0.844503 PERQ amino acid-rich with GYF domain-containing protein 2 OS=Mus musculus GN=Gigyf2 PE=1 SV=2
Ppp2r1a	Q76MZ3	7	7	217702.4	194905.4	218294.7	212390.6	154858	195068.6	228168.6	210879.4	215977.3	193421.4	0.529901	0.275805	199630.2	208703.1	Up	0.064122	0.844503 Serine/threonine-protein phosphatase 2A 65 kDa regulatory subunit A alpha isoform OS=Mus musculus GN=Ppp2r1a PE=1 SV=3
Ppp1r3a	Q99MR9	1	1	840926	506383	365398	329944	317572	551884	240589	319252	448431	440786	0.544613	0.263912	472044.6	400188.4	Down	-0.23824	0.851643 Protein phosphatase 1 regulatory subunit 3A OS=Mus musculus GN=Ppp1r3a PE=1 SV=2
Psma1	Q9R1P4	10	10	452786.2	219355.5	236476	211708.1	248171.3	270215.6	241863	272418.4	231760.9	198605.2	0.545642	0.263092	273699.4	242972.6	Down	-0.1718	0.85175 Proteasome subunit alpha type-1 OS=Mus musculus GN=Psma1 PE=1 SV=1
Nlgn3	Q8BYM5	4	4	137586	71608.33	81448.67	58854.33	95884	100798.7	78419	73734	63330	81285	0.545981	0.262822	89076.27	79513.33	Down	-0.16384	0.85175 Neuroligin-3 OS=Mus musculus GN=Nlgn3 PE=1 SV=2
Etl4	A2AQ25-4	5	5	262215.8	176245.8	162013.2	174912.4	187906	198099	170983.6	181093.2	182745	169964.4	0.546225	0.262628	192658.6	180577	Down	-0.09343	0.85175 Isoform 4 of Sickle tail protein OS=Mus musculus GN=Skt
Trim2	Q9ESN6	3	3	NA	12340.33	18641.33	23066.67	7389	61507.67	10894	9477.33	14716.67	15374	0.530221	0.275543	15359.33	22393.93	Up	0.543992	0.844503 Tripartite motif-containing protein 2 OS=Mus musculus GN=Trim2 PE=1 SV=1
Prkar1a	Q9DBC7	7	7	24202.2	37907	38452.8	43637.2	42678.2	56460.2	35212	46794.4	40418.6	27795.6	0.530378	0.275414	37375.48	41336.16	Up	0.145312	0.844503 cAMP-dependent protein kinase type I-alpha regulatory subunit OS=Mus musculus GN=Prkar1a PE=1 SV=3
Ank2	Q8C8R3	48	48	290185.2	362419	325300.2	328330.5	362057.2	380211.8	312228.5	346908.9	341703.1	343824.7	0.531055	0.27486	333658.4	344975.4	Up	0.048122	0.844564 Ankyrin-2 OS=Mus musculus GN=Ank2 PE=1 SV=2
Mrpl50	Q8VDT9	1	1	250348	310473	322927	373823	293508	354384	336421	381299	258715	314597	0.531562	0.274446	310215.8	329083.2	Up	0.08518	0.844564 39S ribosomal protein L50, mitochondrial OS=Mus musculus GN=Mrpl50 PE=2 SV=2
Psma2	P49722	3	3	140816.3	96875.67	96453.67	100254.3	95598	108811.7	116567	102393.7	123137	110419.3	0.534197	0.272299	105999.6	112265.7	Up	0.082859	0.846591 Proteasome subunit alpha type-2 OS=Mus musculus GN=Psma2 PE=1 SV=3
Grin2b	Q01097	6	6	713795	752064.3	783053.7	833327.7	976560.3	793967.5	772402.3	803780.7	729315.2	806077	0.550762	0.259036	811760.2	781108.5	Down	-0.05553	0.854725 Glutamate receptor ionotropic, NMDA 2B OS=Mus musculus GN=Grin2b PE=1 SV=3
Shank3	Q4ACU6-1	8	8	164456.4	524850.7	262131	388964.9	302557.4	452256.1	292042.1	272416.6	419511	442456.3	0.535004	0.271643	328592.1	375736.4	Up	0.193423	0.846591 Isoform 10 of SH3 and multiple ankyrin repeat domains protein 3 OS=Mus musculus GN=Shank3
Camk2g	Q923T9-3	14	14	222366.5	324624.4	248779.3	316602.6	271067.8	275114.5	251003.4	271653.5	260262.8	260774.1	0.550964	0.258877	276688.1	263761.7	Down	-0.06903	0.854725 Isoform 3 of Calcium/calmodulin-dependent protein kinase type II subunit gamma OS=Mus musculus GN=Camk2g
Nfu1	Q9QZ23	3	3	107356.3	286935.3	272449.7	338406.7	295521.7	309254	326995.7	276896.3	264794.7	262107	0.535106	0.27156	260133.9	288009.5	Up	0.146862	0.846591 NFU1 iron-sulfur cluster scaffold homolog, mitochondrial OS=Mus musculus GN=Nfu1 PE=1 SV=2
Pycr2	Q922Q4	1	1	1237699	791622	892472	753467	757919	630816	938128	860541	878978	793716	0.551673	0.258318	886635.8	820435.8	Down	-0.11195	0.854725 Pyrroline-5-carboxylate reductase 2 OS=Mus musculus GN=Pycr2 PE=2 SV=1
Hnrnpa0	Q9CX86	3	3	105156	75101	98452.5	66319	111767	53420.5	116945	91040	80556	72182	0.551856	0.258174	91359.1	82828.7	Down	-0.14142	0.854725 Heterogeneous nuclear ribonucleoprotein A0 OS=Mus musculus GN=Hnrnpa0 PE=1 SV=1
Fah	P35505	4	4	98582	130832.3	195605.8	166610	168769	137590.3	153200	163015.5	117947.8	129625	0.552376	0.257765	152079.8	140275.7	Down	-0.11656	0.854725 Fumarylacetoacetase OS=Mus musculus GN=Fah PE=1 SV=2
Eif3b	Q8JZQ9	3	3	NA	7066	NA	3593	NA	NA	NA	NA	13002	4838	0.536176	0.270693	5329.5	8920	Up	0.743044	0.847761 Eukaryotic translation initiation factor 3 subunit B OS=Mus musculus GN=Eif3b PE=1 SV=1
Tom1l2	Q5SRX1-5	1	1	NA	NA	21797	21507	34207	26730	28261	20541	44165	28436	0.537012	0.270016	25837	29626.6	Up	0.197455	0.848489 Isoform 5 of TOM1-like protein 2 OS=Mus musculus GN=Tom1l2
Epn1	Q80VP1	15	15	241018.7	197769.5	198924.6	178953.8	200746	230160.9	187145.6	202947.7	176926.8	175781.5	0.55325	0.257079	203482.5	194592.5	Down	-0.06445	0.854725 Epsin-1 OS=Mus musculus GN=Epn1 PE=1 SV=3
Pura	P42669	17	17	424327.2	314869	268052.4	241039.2	354220.7	328427.5	305678.6	300083.5	307054	250972.2	0.553494	0.256887	320501.7	298443.2	Down	-0.10288	0.854725 Transcriptional activator protein Pur-alpha OS=Mus musculus GN=Pura PE=1 SV=1
Idh1	O88844	8	8	265913.9	363383.4	429091	352954	362641.6	357591.8	376790.8	384834	378653.9	364749.8	0.537875	0.269319	354796.8	372524	Up	0.070341	0.848489 Isocitrate dehydrogenase [NADP] cytoplasmic OS=Mus musculus GN=Idh1 PE=1 SV=2
Sh3glb1	Q9JK48	4	4	24619.33	78250.67	60220	106583.3	113374	82753.33	79104.67	81286.33	91555.33	104185	0.537961	0.269249	76609.47	87776.93	Up	0.196319	0.848489 Endophilin-B1 OS=Mus musculus GN=Sh3glb1 PE=1 SV=1
Arhgap12	Q8C0D4	1	1	77264	122919	131330	124899	112160	115878	115216	121882	131083	117812	0.5386	0.268734	113714.4	120374.2	Up	0.082111	0.848595 Rho GTPase-activating protein 12 OS=Mus musculus GN=Arhgap12 PE=1 SV=2
Ank2	Q8C8R3-5	47	47	285704.9	363483.7	325612	327495	361419.8	377262.1	311998	346803.8	338981.9	345365.7	0.539954	0.267643	332743.1	344082.3	Up	0.048345	0.84902 Isoform 5 of Ankyrin-2 OS=Mus musculus GN=Ank2
Actn3	O88990	4	4	220622	216813	315562.3	329940.7	387174	330078.7	347376	321975.7	279099.7	306188.3	0.541266	0.266589	294022.4	316943.7	Up	0.1083	0.849697 Alpha-actinin-3 OS=Mus musculus GN=Actn3 PE=2 SV=1
Arsa	P50428	2	2	297716	200203	208939.5	180190.5	215808	182486.5	225594.5	225904	200860.5	199300.5	0.555485	0.255328	220571.4	206829.2	Down	-0.09281	0.854725 Arylsulfatase A OS=Mus musculus GN=Arsa PE=2 SV=2
Ears2	Q9CXJ1	2	2	28118.5	58806	46736	48508	38439.5	29095	50797	47708	67789.5	50841.5	0.541379	0.266499	44121.6	49246.2	Up	0.158527	0.849697 Probable glutamate--tRNA ligase, mitochondrial OS=Mus musculus GN=Ears2 PE=1 SV=1
Sike1	Q9CPR7	1	1	486429	267967	296736	190907	333362	431708	312310	251384	160003	212396	0.555995	0.254929	315080.2	273560.2	Down	-0.20386	0.854725 Suppressor of IKBKE 1 OS=Mus musculus GN=Sike1 PE=2 SV=1
Clip3	B9EHT4	3	3	7973	45593.67	34380	33991.33	44692.33	28157.67	46744	34250.67	41058.33	40784	0.541788	0.266171	33326.07	38198.93	Up	0.196881	0.849817 CAP-Gly domain-containing linker protein 3 OS=Mus musculus GN=Clip3 PE=1 SV=1
Zfp365	Q8BG89	2	2	12422.5	28300	36473.5	58851.5	95726	39259.5	38412	115701	32163.5	74140.5	0.543173	0.265062	46354.7	59935.3	Up	0.37069	0.851138 Protein ZNF365 OS=Mus musculus GN=Znf365 PE=2 SV=1
Csde1	Q91W50	5	5	605590.2	602929	535193.2	576531	688814.8	683004.8	664683.2	554511.6	587316.2	629359.2	0.543828	0.264538	601811.6	623775	Up	0.051714	0.851142 Cold shock domain-containing protein E1 OS=Mus musculus GN=Csde1 PE=2 SV=1
Cnpy3	Q9DAU1-2	2	2	NA	90592	117546.5	114066.5	74407	49901	117439.5	139060	117588	132224	0.54396	0.264433	99153	111242.5	Up	0.16598	0.851142 Isoform 2 of Protein canopy homolog 3 OS=Mus musculus GN=Cnpy3
Eci2	Q9WUR2	3	3	128772.3	153672.7	238821.7	187486.7	231769.3	188252.3	207029.7	197136.7	118905.7	145635.3	0.557255	0.253946	188104.5	171391.9	Down	-0.13424	0.854725 Enoyl-CoA delta isomerase 2, mitochondrial OS=Mus musculus GN=Eci2 PE=1 SV=2
Hsdl2	Q2TPA8	2	2	129024.5	142797.5	157479.5	124813.5	175744.5	210682	141270	162693	111081.5	164058.5	0.546228	0.262626	145971.9	157957	Up	0.113841	0.85175 Hydroxysteroid dehydrogenase-like protein 2 OS=Mus musculus GN=Hsdl2 PE=1 SV=1
Fabp7	P51880	3	3	127906	86749.67	141942	113695.7	146602	142138	128769.3	124309.3	88842	85151.33	0.560089	0.251743	123379.1	113842	Down	-0.11606	0.856285 Fatty acid-binding protein, brain OS=Mus musculus GN=Fabp7 PE=1 SV=2
4-Sep	P28661-3	5	5	184978.4	133989.6	94238	110261.6	142858.2	121083.4	125364.2	106638.6	133482.4	129038.4	0.560137	0.251706	133265.2	123121.4	Down	-0.11422	0.856285 Isoform 3 of Septin-4 OS=Mus musculus GN=Sept4
Rpl11	Q9CXW4	3	3	NA	68261.5	66311	57472.5	85263	25901	68010	194278	87219	65744	0.546343	0.262535	69327	88230.4	Up	0.347858	0.85175 60S ribosomal protein L11 OS=Mus musculus GN=Rpl11 PE=1 SV=4
Ptgr2	Q8VDQ1-2	2	2	124719	133557	145625	148940.5	134786	148289.5	145449	136510	119542.5	112246	0.560258	0.251612	137525.5	132407.4	Down	-0.05472	0.856285 Isoform 2 of Prostaglandin reductase 2 OS=Mus musculus GN=Ptgr2
Acyp1	P56376	5	5	93661.5	108872.5	132631.8	116503.5	139982.8	144429.5	122375.5	123536.8	123661.5	109346	0.546892	0.262098	118330.4	124669.9	Up	0.075292	0.852088 Acylphosphatase-1 OS=Mus musculus GN=Acyp1 PE=2 SV=2
Hagh	Q99KB8	2	2	NA	21112.5	10132.5	16440	16221	28917	11114	19067	14694.5	17917	0.5473	0.261775	15976.5	18341.9	Up	0.199192	0.852206 Hydroxyacylglutathione hydrolase, mitochondrial OS=Mus musculus GN=Hagh PE=1 SV=2
Cttn	Q60598	3	3	62575.67	67356.33	91154	62261.33	105776.7	91146	75108	71018	67313.33	51541	0.561746	0.25046	77824.8	71225.27	Down	-0.12784	0.856285 Src substrate cortactin OS=Mus musculus GN=Cttn PE=1 SV=2
Ehd3	Q9QXY6	7	7	429799.2	536751	522012.8	558935.8	396263.4	435544.4	607941.4	488596.8	538587	507152.8	0.548653	0.260702	488752.4	515564.5	Up	0.077049	0.853793 EH domain-containing protein 3 OS=Mus musculus GN=Ehd3 PE=1 SV=2
Ank2	Q8C8R3-2	50	50	290424.1	353120.6	321948.7	321018.9	360428.4	376165.7	309282.5	340157.5	336254.7	336524.8	0.550813	0.258996	329388.2	339677	Up	0.044375	0.854725 Isoform 2 of Ankyrin-2 OS=Mus musculus GN=Ank2
Fam89b	Q9QUI1	1	1	NA	66535	125193	105733	160492	221531	199799	57491	112460	97206	0.551325	0.258592	114488.3	137697.4	Up	0.266302	0.854725 Protein FAM89B OS=Mus musculus GN=Fam89b PE=1 SV=1
Rpl10	Q6ZWV3	9	9	1033157	484607.2	492515.2	398946.8	532381.9	609511.3	523826.1	563041.4	483881	391351.1	0.562876	0.249587	588321.6	514322.2	Down	-0.19393	0.85719 60S ribosomal protein L10 OS=Mus musculus GN=Rpl10 PE=1 SV=3
Tiam1	Q60610	2	2	26260.5	28116	61444	42629	59412	37454	40461.5	42957.5	38531.5	34759	0.563445	0.249148	43572.3	38832.7	Down	-0.16614	0.857547 T-lymphoma invasion and metastasis-inducing protein 1 OS=Mus musculus GN=Tiam1 PE=1 SV=1
Pgm2	Q9D0F9	1	1	NA	42248	43303	29366	23392	14234	34733	36179	31930	36244	0.564053	0.24868	34577.25	30664	Down	-0.17328	0.857963 Phosphoglucomutase-1 OS=Mus musculus GN=Pgm1 PE=1 SV=4
Fdps	Q920E5	2	2	NA	19266	12661	13611	5655	8764	7152	5995	14040	17305	0.565127	0.247854	12798.25	10651.2	Down	-0.26493	0.858606 Farnesyl pyrophosphate synthase OS=Mus musculus GN=Fdps PE=1 SV=1
Ap3b2	Q9JME5	4	4	170352.8	338948	289479.5	295263.8	254349.3	252625	273387	310149.3	284903.5	325447.3	0.552457	0.257702	269678.7	289302.4	Up	0.101337	0.854725 AP-3 complex subunit beta-2 OS=Mus musculus GN=Ap3b2 PE=1 SV=2
Srp14	P16254	2	2	622973.5	476545	355673	357143	407584.5	423405.5	355346	423506.5	363813.5	486263	0.567367	0.246136	443983.8	410466.9	Down	-0.11324	0.86147 Signal recognition particle 14 kDa protein OS=Mus musculus GN=Srp14 PE=1 SV=1
Ctnnb1	Q02248	6	6	52795.67	89188	64210	85746	70743.33	54621	97381.33	55248	86640	109465.7	0.552653	0.257547	72536.6	80671.2	Up	0.153345	0.854725 Catenin beta-1 OS=Mus musculus GN=Ctnnb1 PE=1 SV=1
Ndufaf4	Q9D1H6	2	2	107153	149634	65864	121831.5	67096	115209	82421	66022.5	87168	105508	0.568243	0.245466	102315.7	91265.7	Down	-0.16488	0.861517 NADH dehydrogenase [ubiquinone] 1 alpha subcomplex assembly factor 4 OS=Mus musculus GN=Ndufaf4 PE=2 SV=1
Gm996	A2AJA9	4	4	235390.8	215414	153900.8	177799.3	211472.3	209212	202626.3	179837.3	176651.8	176842.8	0.568406	0.245341	198795.4	189034	Down	-0.07264	0.861517 Uncharacterized protein C9orf172 homolog OS=Mus musculus GN=Gm996 PE=1 SV=1
Ssh3	Q8K330	1	1	336607	68901	92060	71828	103807	175942	82744	103472	93887	50940	0.57008	0.244064	134640.6	101397	Down	-0.4091	0.86346 Protein phosphatase Slingshot homolog 3 OS=Mus musculus GN=Ssh3 PE=1 SV=1
Kif5c	P28738	10	10	420511.7	316427.6	378079.9	337023.7	355142.4	381008.1	362183.9	377178.8	390559	356524.2	0.553569	0.256828	361437	373490.8	Up	0.047328	0.854725 Kinesin heavy chain isoform 5C OS=Mus musculus GN=Kif5c PE=1 SV=3
Msi1	Q61474-2	1	1	NA	43863	25834	33449	36720	18198	46163	40363	27038	23917	0.570748	0.243556	34966.5	31135.8	Down	-0.1674	0.863535 Isoform 2 of RNA-binding protein Musashi homolog 1 OS=Mus musculus GN=Msi1
Kxd1	Q80XH1	1	1	157861	92964	91767	77830	111109	123112	82439	102835	89983	85999	0.571496	0.242987	106306.2	96873.6	Down	-0.13405	0.863773 KxDL motif-containing protein 1 OS=Mus musculus GN=Kxd1 PE=1 SV=1
Psmd4	O35226-4	1	1	742671	483151	622737	454938	647476	712743	559065	628974	429618	379537	0.57158	0.242923	590194.6	541987.4	Down	-0.12293	0.863773 Isoform Rpn10D of 26S proteasome non-ATPase regulatory subunit 4 OS=Mus musculus GN=Psmd4
Pi4kb	Q8BKC8-3	1	1	376769	272026	386374	304326	398344	321639	393031	313843	306895	314688	0.57316	0.241724	347567.8	330019.2	Down	-0.07474	0.865297 Isoform 3 of Phosphatidylinositol 4-kinase beta OS=Mus musculus GN=Pi4kb
Hsd17b4	P51660	4	4	71273.75	108584	96462.75	91164.5	102186	83825.25	108666.8	105800.3	100084.8	95276.75	0.554285	0.256267	93934.2	98730.75	Up	0.071849	0.854725 Peroxisomal multifunctional enzyme type 2 OS=Mus musculus GN=Hsd17b4 PE=1 SV=3
Ylpm1	Q9R0I7	2	2	NA	2959	3388	4913	NA	7723	2920	4165	3001	4260	0.554672	0.255964	3753.333	4413.8	Up	0.233849	0.854725 YLP motif-containing protein 1 OS=Mus musculus GN=Ylpm1 PE=2 SV=2
Camsap3	Q80VC9	7	7	164323.3	173947.8	169913.5	158018.8	157563.8	214789.3	195175.5	164288.3	145885	148061.8	0.554686	0.255953	164753.4	173640	Up	0.075791	0.854725 Calmodulin-regulated spectrin-associated protein 3 OS=Mus musculus GN=Camsap3 PE=1 SV=1
Dync1li1	Q8R1Q8	4	4	85394.5	96813.75	79080.5	78653.5	91018.25	75399.25	93729.25	91510	73549.25	80991.5	0.575285	0.240117	86192.1	83035.85	Down	-0.05382	0.86598 Cytoplasmic dynein 1 light intermediate chain 1 OS=Mus musculus GN=Dync1li1 PE=1 SV=1
Add3	Q9QYB5	3	3	42447	55186.67	44983	39892	46055.67	57724.67	44279.33	37384.33	40448	34593.33	0.575385	0.240041	45712.87	42885.93	Down	-0.0921	0.86598 Gamma-adducin OS=Mus musculus GN=Add3 PE=1 SV=2
Arfgap1	Q9EPJ9	11	11	91659	78385.57	75170	73170.57	84595.57	87084.29	89970.14	65952.57	74163.86	68437.43	0.575426	0.240011	80596.14	77121.66	Down	-0.06357	0.86598 ADP-ribosylation factor GTPase-activating protein 1 OS=Mus musculus GN=Arfgap1 PE=1 SV=2
Actl6b	Q99MR0	1	1	71296	122331	140848	127806	186174	78122	144458	142299	187742	177767	0.555104	0.255626	129691	146077.6	Up	0.171657	0.854725 Actin-like protein 6B OS=Mus musculus GN=Actl6b PE=1 SV=1
Naca	Q60817	1	1	370968	494719	413994	323179	624632	621958	423029	467145	473620	438883	0.555654	0.255196	445498.4	484927	Up	0.122347	0.854725 Nascent polypeptide-associated complex subunit alpha OS=Mus musculus GN=Naca PE=1 SV=1
Commd6	Q3V4B5	1	1	478989	174171	188588	162559	295296	344593	197676	229906	168070	157876	0.576762	0.239003	259920.6	219624.2	Down	-0.24303	0.86598 COMM domain-containing protein 6 OS=Mus musculus GN=Commd6 PE=2 SV=1
Aldoc	P05063	37	37	291326.6	301542.2	291011.1	331317.9	271817.5	373737.9	305941.6	295856.5	278656.1	292700.2	0.556446	0.254577	297403	309378.5	Up	0.056953	0.854725 Fructose-bisphosphate aldolase C OS=Mus musculus GN=Aldoc PE=1 SV=4
Rpl12	P35979	2	2	1364001	1285433	1101581	1215430	1130096	1430565	1236031	1114969	1193631	1349205	0.556453	0.254572	1219308	1264880	Up	0.052938	0.854725 60S ribosomal protein L12 OS=Mus musculus GN=Rpl12 PE=1 SV=2
Mecr	Q9DCS3	3	3	NA	89260	51801.5	83058.5	59698	35940	68937.5	62098.5	79094.5	74174	0.577719	0.238283	70954.5	64048.9	Down	-0.14772	0.86598 Trans-2-enoyl-CoA reductase, mitochondrial OS=Mus musculus GN=Mecr PE=1 SV=2
Ccdc22	Q9JIG7	4	4	25178.5	42900.25	29696.25	34075	36536.5	28796.5	37095	31107.25	33507	27757.25	0.577862	0.238176	33677.3	31652.6	Down	-0.08945	0.86598 Coiled-coil domain-containing protein 22 OS=Mus musculus GN=Ccdc22 PE=1 SV=1
Nefh	P19246	6	6	98717.5	190683.8	209536.5	194987	162835.7	169543.8	196989.3	211650.7	170253.5	175271.5	0.556661	0.254409	171352.1	184741.8	Up	0.108546	0.854725 Neurofilament heavy polypeptide OS=Mus musculus GN=Nefh PE=1 SV=3
Nckap1	P28660	2	2	84133	40001.5	75817	51710	88323	79603	69270	57773	56931.5	43740	0.579386	0.237032	67996.9	61463.5	Down	-0.14574	0.867387 Nck-associated protein 1 OS=Mus musculus GN=Nckap1 PE=1 SV=2
Tjp2	Q9Z0U1	3	3	511683	381248.3	404072.7	375128	447062	437127	399983	414905.3	421188	363183	0.57976	0.236752	423838.8	407277.3	Down	-0.0575	0.86744 Tight junction protein ZO-2 OS=Mus musculus GN=Tjp2 PE=1 SV=2
Pcsk1n	Q9QXV0	6	6	578531.8	392563.8	388821.5	357288	424945.5	444576.8	335696.5	436595	400730.8	397253.5	0.580442	0.236241	428430.1	402970.5	Down	-0.08839	0.867629 ProSAAS OS=Mus musculus GN=Pcsk1n PE=1 SV=2
Eif2s3y	Q9Z0N2	1	1	NA	81170	79132	100399	88213	52740	135617	66659	116652	117636	0.557128	0.254045	87228.5	97840.8	Up	0.165637	0.854725 Eukaryotic translation initiation factor 2 subunit 3, Y-linked OS=Mus musculus GN=Eif2s3y PE=2 SV=2
Nhp2	Q9CRB2	2	2	102286.5	124525.5	152119.5	107477	114105.5	73664.5	164216.5	155856	152971	113041.5	0.559719	0.25203	120102.8	131949.9	Up	0.13572	0.856285 H/ACA ribonucleoprotein complex subunit 2 OS=Mus musculus GN=Nhp2 PE=2 SV=1
Nek7	Q9ES74	1	1	218866	237088	155986	246121	222977	156368	192848	227962	287396	321454	0.560152	0.251694	216207.6	237205.6	Up	0.133721	0.856285 Serine/threonine-protein kinase Nek7 OS=Mus musculus GN=Nek7 PE=2 SV=1
Aldh1a1	P24549	4	4	62730.75	139216.8	134159.3	94517.75	122410.8	114922.8	123309.3	127071.5	129528.8	104845.3	0.561156	0.250916	110607.1	119935.5	Up	0.116815	0.856285 Retinal dehydrogenase 1 OS=Mus musculus GN=Aldh1a1 PE=1 SV=5
Grin2a	P35436	2	2	238221.5	291508	254051.5	248884	374136.5	320000	282265.5	255469.5	251567	209198.5	0.584452	0.233251	281360.3	263700.1	Down	-0.09352	0.869765 Glutamate receptor ionotropic, NMDA 2A OS=Mus musculus GN=Grin2a PE=1 SV=2
Impad1	Q80V26	1	1	306997	267606	286464	243099	295223	368449	324396	255397	264557	262815	0.561674	0.250516	279877.8	295122.8	Up	0.076518	0.856285 Inositol monophosphatase 3 OS=Mus musculus GN=Impad1 PE=1 SV=1
Rpl8	P62918	8	8	451846.3	253692.4	220077	225767.3	254485.5	356902.4	238282.4	249799	210522	203684.1	0.585892	0.232182	281173.7	251838	Down	-0.15897	0.869765 60S ribosomal protein L8 OS=Mus musculus GN=Rpl8 PE=2 SV=2
Gart	Q64737	7	7	113776	133540	148075.1	141178.7	123132.4	143441.6	133028.3	136282.4	118079	101362.9	0.586946	0.231402	131940.5	126438.8	Down	-0.06145	0.869765 Trifunctional purine biosynthetic protein adenosine-3 OS=Mus musculus GN=Gart PE=2 SV=3
Ywhab	Q9CQV8	9	9	1295236	1021381	1105899	979730.8	1060462	1057660	1207099	1002222	1115563	851254.7	0.58701	0.231355	1092542	1046760	Down	-0.06176	0.869765 14-3-3 protein beta/alpha OS=Mus musculus GN=Ywhab PE=1 SV=3
Tdrkh	Q80VL1	2	2	12930	38984	34866	31665	41428.5	60905	35609	27959	27821.5	31794.5	0.561755	0.250453	31974.7	36817.8	Up	0.203473	0.856285 Tudor and KH domain-containing protein OS=Mus musculus GN=Tdrkh PE=1 SV=1
Adgrb2	Q8CGM1-3	1	1	65288	125792	133412	133755	177582	138756	108873	150589	132595	167642	0.561924	0.250322	127165.8	139691	Up	0.135528	0.856285 Isoform 3 of Brain-specific angiogenesis inhibitor 2 OS=Mus musculus GN=Bai2
Snrpa1	P57784	1	1	152901	106863	129163	89056	151030	62172	126831	137415	131444	118956	0.587217	0.231201	125802.6	115363.6	Down	-0.12497	0.869765 U2 small nuclear ribonucleoprotein A' OS=Mus musculus GN=Snrpa1 PE=1 SV=2
Ica1l	Q3TY65	1	1	NA	117476	127410	146564	176884	214512	124265	132836	210136	108416	0.561948	0.250304	142083.5	158033	Up	0.153487	0.856285 Islet cell autoantigen 1-like protein OS=Mus musculus GN=Ica1l PE=2 SV=1
Dbnl	Q62418-3	17	17	196843	149503.2	138167.2	139895	125890.8	139738.5	152999.2	141212.7	138410.9	141239	0.587876	0.230714	150059.8	142720	Down	-0.07235	0.869765 Isoform 3 of Drebrin-like protein OS=Mus musculus GN=Dbnl
Snca	O55042	44	44	342712.5	367517.3	283674.7	307382.5	292189.7	342588.8	331825.4	281052.6	265691.4	311444.1	0.58789	0.230704	318695.3	306520.5	Down	-0.05619	0.869765 Alpha-synuclein OS=Mus musculus GN=Snca PE=1 SV=2
Snca	O55042-2	43	43	342712.5	367517.3	283674.7	307382.5	292189.7	342588.8	331825.4	281052.6	265691.4	311444.1	0.58789	0.230704	318695.3	306520.5	Down	-0.05619	0.869765 Isoform 2 of Alpha-synuclein OS=Mus musculus GN=Snca
Reps1	O54916-4	1	1	NA	14191	11859	21059	14604	55189	10436	16588	10784	11657	0.565146	0.247839	15428.25	20930.8	Up	0.440053	0.858606 Isoform 4 of RalBP1-associated Eps domain-containing protein 1 OS=Mus musculus GN=Reps1
Gpi1	P06745	11	11	198365.1	155020.3	216153	190606.2	179622.7	166869.2	197602.3	202559.5	191979.6	135282.3	0.588554	0.230214	187955.3	178858.6	Down	-0.07156	0.869765 Glucose-6-phosphate isomerase OS=Mus musculus GN=Gpi PE=1 SV=4
Sod1	P08228	2	2	4531.5	98208	260380	179090.5	242689	133446	193984.5	255230.5	154313	205868	0.568062	0.245604	156979.8	188568.4	Up	0.264509	0.861517 Superoxide dismutase [Cu-Zn] OS=Mus musculus GN=Sod1 PE=1 SV=2
Hist1h2ak	Q8CGP7	4	4	200418.3	273181	472542.3	510435.3	681998.3	221432.7	427170.3	479211.7	391411.3	342819.7	0.588821	0.230017	427715.1	372409.1	Down	-0.19976	0.869765 Histone H2A type 1-K OS=Mus musculus GN=Hist1h2ak PE=1 SV=3
Mical3	Q8CJ19	3	3	207249.3	252670	229986.3	221955	277460.3	337802.3	221181.7	263351.3	215247.3	229130	0.570362	0.243849	237864.2	253342.5	Up	0.090951	0.86346 Protein-methionine sulfoxide oxidase MICAL3 OS=Mus musculus GN=Mical3 PE=2 SV=2
Plxna4	Q80UG2	1	1	593890	1054631	853047	1052923	941014	1009880	1016388	798601	895851	1065405	0.57335	0.24158	899101	957225	Up	0.090375	0.865297 Plexin-A4 OS=Mus musculus GN=Plxna4 PE=1 SV=3

Rab1a	P62821	5	5	24264.2	90078	105052	144891.6	109849.2	113128.2	116561.8	92680.4	110841.6	102437.4	0.573761	0.241269	94827	107129.9	Up	0.175991	0.865297	Ras-related protein Rab-1A OS=Mus musculus GN=Rab1A PE=1 SV=3
Sod3	O09164	1	1	148018	143871	175291	137926	210386	196666	145645	241677	114596	193197	0.573939	0.241134	163098.4	178356.2	Up	0.129019	0.865297	Extracellular superoxide dismutase [Cu-Zn] OS=Mus musculus GN=Sod3 PE=1 SV=1
Trim32	Q8CH72	2	2	72027	46906.5	65684.5	52491	49216.5	48446.5	58001	60258.5	72188	65874.5	0.576438	0.239247	57265.1	60953.7	Up	0.090058	0.86598	E3 ubiquitin-protein ligase TRIM32 OS=Mus musculus GN=Trim32 PE=1 SV=2
Phldb1	Q6PDH0	2	2	91080	89717.5	175404	123678	163633.5	210424	128961	150444	104440.5	123498	0.576654	0.239085	128702.6	143553.5	Up	0.157547	0.86598	Pleckstrin homology-like domain family B member 1 OS=Mus musculus GN=Phldb1 PE=1 SV=1
Sfn	O70456	4	4	1947508	1907498	1756830	1792599	1686872	1767897	2087191	1744478	2065378	1707979	0.577154	0.238708	1818261	1874584	Up	0.044011	0.86598	14-3-3 protein sigma OS=Mus musculus GN=Sfn PE=1 SV=2
Sh3bgrl2	Q8BG73	5	5	74737	157375.3	151565	165786.5	118574.3	143965.3	139404.5	139031	150589.5	146074	0.577383	0.238536	133607.6	143812.9	Up	0.106191	0.86598	SH3 domain-binding glutamic acid-rich-like protein 2 OS=Mus musculus GN=Sh3bgrl2 PE=1 SV=1
Pvalb	P32848	4	4	555418.3	777160.3	453952.7	624571.7	326356.3	358127	508479	569792.7	529723.3	531579	0.5925	0.227312	547491.9	499540.2	Down	-0.13224	0.871178	Parvalbumin alpha OS=Mus musculus GN=Pvalb PE=1 SV=3
Spag9	Q58A65-3	1	1	NA	32857	52889	44970	16040	NA	52681	59824	40484	21176	0.578108	0.237991	36689	43541.25	Up	0.247035	0.86598	Isoform 3 of C-Jun-amino-terminal kinase-interacting protein 4 OS=Mus musculus GN=Spag9
Tdrd3	Q91W18-3	1	1	29837	31292	20284	22973	12610	22133	11532	24419	16494	29572	0.593187	0.226808	23399.2	20830	Down	-0.1678	0.871194	Isoform 3 of Tudor domain-containing protein 3 OS=Mus musculus GN=Tdrd3
Wdr13	Q91V09	3	3	39168.5	61515.5	71584.5	62516	128279.5	53466	56392.5	73823.5	75787	58985.5	0.593486	0.22659	72612.8	63690.9	Down	-0.18914	0.871194	WD repeat-containing protein 13 OS=Mus musculus GN=Wdr13 PE=1 SV=1
Cacna2d1	O08532-3	6	6	410336	407206	399643.5	419400.5	370281.8	352815.3	478965.3	378231.3	374096.3	351600	0.594253	0.226029	401373.6	387141.6	Down	-0.05208	0.871821	Isoform 2C of Voltage-dependent calcium channel subunit alpha-2/delta-1 OS=Mus musculus GN=Cacna2d1
Ndufa4	Q62425	3	3	591250.7	388620.7	384326.7	421723	310795	465109.3	383480	448661	344544.7	298595.3	0.594975	0.225501	419343.2	388078.1	Down	-0.11178	0.872003	NADH dehydrogenase [ubiquinone] 1 alpha subcomplex subunit 4 OS=Mus musculus GN=Ndufa4 PE=1 SV=2
Psmd5	Q8BJY1	1	1	NA	20803	30144	12682	11586	NA	30701	14993	37423	10263	0.580827	0.235953	18803.75	23345	Up	0.312093	0.867629	26S proteasome non-ATPase regulatory subunit 5 OS=Mus musculus GN=Psmd5 PE=1 SV=4
Dapk3	O54784	1	1	68906	38932	52896	41840	54988	65840	53116	72193	39348	49824	0.580902	0.235897	51512.4	56064.2	Up	0.12216	0.867629	Death-associated protein kinase 3 OS=Mus musculus GN=Dapk3 PE=1 SV=1
Mrpl46	Q9EQI8	1	1	145787	155265	218824	164172	209288	213508	135215	154160	198794	129530	0.596377	0.224479	178667.2	166241.4	Down	-0.104	0.872938	39S ribosomal protein L46, mitochondrial OS=Mus musculus GN=Mrpl46 PE=1 SV=1
Psd	Q5DTT2-2	2	2	99476.5	185451.5	146127	124607.5	207924.5	140736	191415	133501.5	198370.5	167628	0.582952	0.234367	152717.4	166330.2	Up	0.123186	0.869765	Isoform 2 of PH and SEC7 domain-containing protein 1 OS=Mus musculus GN=Psd
Eef1d	P57776-2	4	4	327802	258333.5	181199.3	223399.3	246188	271000.3	239770	244496.5	258614	299413.5	0.584283	0.233377	247384.4	262658.9	Up	0.086436	0.869765	Isoform 2 of Elongation factor 1-delta OS=Mus musculus GN=Eef1d
Hist2h2ac	Q64523	3	3	293485.5	408346.5	703692	762586	1012554	329462	636973	714732.5	584511.5	512764	0.597929	0.22335	636132.8	555688.6	Down	-0.19505	0.873215	Histone H2A type 2-C OS=Mus musculus GN=Hist2h2ac PE=1 SV=3
H2afj	Q8R1M2	3	3	293485.5	408346.5	703692	762586	1012554	329462	636973	714732.5	584511.5	512764	0.597929	0.22335	636132.8	555688.6	Down	-0.19505	0.873215	Histone H2A.J OS=Mus musculus GN=H2afj PE=1 SV=1
Sdf2	Q9DCT5	3	3	375072	329666	305982	274685	422575	528202	324855	408375	343233	255449	0.584893	0.232924	341596	372022.8	Up	0.1231	0.869765	Stromal cell-derived factor 2 OS=Mus musculus GN=Sdf2 PE=2 SV=1
Ipo7	Q9EPL8	1	1	32301	49209	115936	88550	92285	111412	85621	99694	89440	45666	0.587151	0.23125	75656.2	86366.6	Up	0.191015	0.869765	Importin-7 OS=Mus musculus GN=Ipo7 PE=1 SV=2
Arpc4	P59999	1	1	3756	1286	2848	2042	2349	866	2498	3077	2037	2303	0.600835	0.221245	2456.2	2156.2	Down	-0.18794	0.875961	Actin-related protein 2/3 complex subunit 4 OS=Mus musculus GN=Arpc4 PE=1 SV=3
Ubqln2	Q9QZM0	1	1	17976	4908	14844	11811	6198	10755	7919	7593	14311	7404	0.602217	0.220247	11147.4	9596.4	Down	-0.21614	0.876574	Ubiquilin-2 OS=Mus musculus GN=Ubqln2 PE=1 SV=2
Dpysl4	O35098	7	7	20169.86	333031.4	217776.3	253844.3	316345.1	186589.9	271480.7	252662.6	314905	289324.9	0.587212	0.231205	228233.4	262992.6	Up	0.204512	0.869765	Dihydropyrimidinase-related protein 4 OS=Mus musculus GN=Dpysl4 PE=1 SV=1
Setd7	Q8VHL1	2	2	NA	114682	83343.5	78932	84354	100621.5	99367	96783.5	88966	91315	0.587218	0.231201	90327.88	95410.6	Up	0.078978	0.869765	Histone-lysine N-methyltransferase SETD7 OS=Mus musculus GN=Setd7 PE=2 SV=2
Gamt	O35969	1	1	283939	168957	255035	198577	238496	120524	205688	289517	242848	192201	0.60308	0.219625	229000.8	210155.6	Down	-0.12389	0.876574	Guanidinoacetate N-methyltransferase OS=Mus musculus GN=Gamt PE=1 SV=1
Atp6v1h	Q8BVE3	5	5	577840.3	818264.3	1201512	1007337	1063772	889561.5	1136087	1146296	1023357	830878.8	0.588207	0.23047	933745.1	1005236	Up	0.106433	0.869765	V-type proton ATPase subunit H OS=Mus musculus GN=Atp6v1h PE=1 SV=1
Apoa1	Q00623	3	3	270788	165651.7	124928.3	136848	152672	113750.7	145436.7	165075.3	163230.3	184543.3	0.603532	0.2193	170177.6	154407.3	Down	-0.1403	0.876574	Apolipoprotein A-I OS=Mus musculus GN=Apoa1 PE=1 SV=2
Blvrb	Q923D2	3	3	431887	248233	290876.7	232845	328669.7	342064.7	264639.3	293602.3	274421.7	250751.7	0.603862	0.219062	306502.3	285095.9	Down	-0.10445	0.876574	Flavin reductase (NADPH) OS=Mus musculus GN=Blvrb PE=2 SV=3
Myo5a	Q99104	15	15	207434.1	297275.7	286361.9	284650.6	293736.9	314969.5	273626.2	303472.9	271700.5	261510.3	0.588767	0.230057	273891.8	285055.9	Up	0.057639	0.869765	Unconventional myosin-Va OS=Mus musculus GN=Myo5a PE=1 SV=2
Ppp1r3f	Q9JIG4	1	1	254227	283317	250871	235158	231738	566288	146273	237666	256290	259257	0.588827	0.230012	251062.2	293154.8	Up	0.223618	0.869765	Protein phosphatase 1 regulatory subunit 3F OS=Mus musculus GN=Ppp1r3f PE=1 SV=3
Ankib1	Q6ZPS6	2	2	22046.5	42505	38920.5	36692	56501.5	37854.5	36312	35961	33312	37655	0.605733	0.217719	39333.1	36218.9	Down	-0.119	0.878107	Ankyrin repeat and IBR domain-containing protein 1 OS=Mus musculus GN=Ankib1 PE=1 SV=2
Pak1	O88643	4	4	1249417	890465.3	810272.3	816271.7	1065477	1114306	851257	1050093	756656.3	756872.3	0.60613	0.217434	966380.6	905836.8	Down	-0.09334	0.878187	Serine/threonine-protein kinase PAK 1 OS=Mus musculus GN=Pak1 PE=1 SV=1
Rida	P52760	14	14	220905.6	161651.4	200543	208280.9	248980.3	238459.4	215255.8	296960.1	217841.4	149975.6	0.589368	0.229613	208072.3	223698.5	Up	0.104471	0.869765	Ribonuclease UK114 OS=Mus musculus GN=Hrsp12 PE=1 SV=3
Tmem132a	Q922P8	2	2	48962	119067	168919	170978.5	189753	142495	200572.5	133012.5	157285	144720	0.589459	0.229546	139535.9	155617	Up	0.157363	0.869765	Transmembrane protein 132A OS=Mus musculus GN=Tmem132a PE=2 SV=2
Edc4	Q3UJB9	7	7	54800.2	93862.4	82531.4	61913	78595.2	85837.4	78297.4	80268.8	70314.8	78539.4	0.59067	0.228655	74340.44	78651.56	Up	0.081328	0.869957	Enhancer of mRNA-decapping protein 4 OS=Mus musculus GN=Edc4 PE=1 SV=2
Celf1	P28659-3	3	3	145212.3	88765.67	111409.3	110826.7	177915.3	117425	108425.3	149345	97540	113244	0.609028	0.215363	126825.9	117195.9	Down	-0.11393	0.880394	Isoform 3 of CUGBP Elav-like family member 1 OS=Mus musculus GN=Celf1
Grm1	P97772	1	1	123724	185466	224535	221581	238127	222039	258417	235355	191215	161454	0.590708	0.228627	198686.6	213696	Up	0.105065	0.869957	Metabotropic glutamate receptor 1 OS=Mus musculus GN=Grm1 PE=1 SV=2
Cotl1	Q9CQI6	3	3	53444	24880.33	30250.33	22533.67	29158.67	34045.33	28911.67	28546	28937.33	24128.67	0.61009	0.214606	32053.4	28913.8	Down	-0.14872	0.880741	Coactosin-like protein OS=Mus musculus GN=Cotl1 PE=1 SV=3
Ddx39	Q8VDW0	1	1	107012	276776	287243	287385	220883	177688	266506	360476	265630	236367	0.590743	0.228601	235859.8	261333.4	Up	0.147962	0.869957	ATP-dependent RNA helicase DDX39A OS=Mus musculus GN=Ddx39a PE=2 SV=1
Tm9sf3	Q9ET30	1	1	247762	261643	165439	228966	230168	267069	198464	210523	222255	176682	0.611526	0.213585	226795.6	214998.6	Down	-0.07707	0.880741	Transmembrane 9 superfamily member 3 OS=Mus musculus GN=Tm9sf3 PE=1 SV=1
Aldh1b1	Q9CZS1	2	2	585471	423503	386835	633806	450247	562477	356941	492079	466956	446042	0.611681	0.213475	495972.4	464899	Down	-0.09334	0.880741	Aldehyde dehydrogenase X, mitochondrial OS=Mus musculus GN=Aldh1b1 PE=1 SV=1
Prickle2	Q80Y24	2	2	41246.5	83243.5	55462	50447.5	41393	57589.5	42466.5	63279.5	60298	74255	0.590946	0.228452	54358.5	59577.7	Up	0.132267	0.869957	Prickle-like protein 2 OS=Mus musculus GN=Prickle2 PE=1 SV=3
Ppp2cb	P62715	1	1	NA	44976	11795	36390	48028	16385	15761	NA	38012	46792	0.61213	0.213156	35297.25	29237.5	Down	-0.27174	0.880741	Serine/threonine-protein phosphatase 2A catalytic subunit beta isoform OS=Mus musculus GN=Ppp2cb PE=1 SV=1
Ppp2ca	P63330	2	2	NA	44976	11795	36390	48028	16385	15761	NA	38012	46792	0.61213	0.213156	35297.25	29237.5	Down	-0.27174	0.880741	Serine/threonine-protein phosphatase 2A catalytic subunit alpha isoform OS=Mus musculus GN=Ppp2ca PE=1 SV=1
Ncan	P55066	11	11	93557.33	189031.6	205933.1	214143.4	158993.6	149892.6	296689	211850.2	126393.1	180052	0.591653	0.227933	172331.8	192975.4	Up	0.163228	0.870498	Neurocan core protein OS=Mus musculus GN=Ncan PE=2 SV=1
Rpl23a	P62751	4	4	152612.5	141831	106159.5	116492	81888.25	100271	134223.5	110210.8	98689	117707.8	0.613247	0.212365	119796.7	112220.4	Down	-0.09425	0.880741	60S ribosomal protein L23a OS=Mus musculus GN=Rpl23a PE=1 SV=1
Rhoa	Q9QUI0	5	5	269538.4	264106.6	233747	293241.8	218234.6	228851.6	259157.4	255849.4	283084.6	303114.8	0.592795	0.227095	255773.7	266011.6	Up	0.056621	0.871178	Transforming protein RhoA OS=Mus musculus GN=Rhoa PE=1 SV=1
Nptn	P97300-4	1	1	NA	785715	495375	571496	600203	677601	681393	603729	618591	670965	0.595144	0.225378	613197.3	650455.8	Up	0.0851	0.872003	Isoform 4 of Neuroplastin OS=Mus musculus GN=Nptn
Limch1	Q3UH68-3	2	2	NA	7346.5	10319.5	16990.5	13902	24210.5	16063	7502	10881.5	11941	0.595398	0.225193	12139.63	14119.6	Up	0.217975	0.872003	Isoform 3 of LIM and calponin homology domains-containing protein 1 OS=Mus musculus GN=Limch1
Etl4	A2AQ25	6	6	246471.2	199685.2	159035.7	180096.8	185293.7	204037.3	171809	178901	186447.8	187562.7	0.614139	0.211733	194116.5	185751.6	Down	-0.06355	0.880741	Sickle tail protein OS=Mus musculus GN=Skt PE=1 SV=1
Serpine2	Q07235	1	1	108077	184161	121686	151576	116178	204837	133362	115480	118897	170027	0.597071	0.223974	136335.6	148520.6	Up	0.123501	0.873096	Glia-derived nexin OS=Mus musculus GN=Serpine2 PE=2 SV=2
Stambp	Q9CQ26	1	1	78390	29050	47382	44735	65031	NA	61147	35119	63393	25110	0.61636	0.210166	52917.6	46192.25	Down	-0.1961	0.882096	STAM-binding protein OS=Mus musculus GN=Stambp PE=2 SV=1
Rab5b	P61021	4	4	76064.75	111058	143054	163025.8	159242.8	130635	145850.3	166015.8	140784.5	119907.3	0.597166	0.223905	130489.1	140638.6	Up	0.108063	0.873096	Ras-related protein Rab-5B OS=Mus musculus GN=Rab5b PE=1 SV=1
Mrps34	Q9JIK9	2	2	216833	99994	219553	155759.5	214009	197465.5	181639	194287.5	172762.5	74953.5	0.617293	0.209509	181229.7	164221.6	Down	-0.14218	0.882096	28S ribosomal protein S34, mitochondrial OS=Mus musculus GN=Mrps34 PE=2 SV=1
Gpc4	P51655	1	1	NA	153455	189593	166696	176363	169518	184019	211325	183297	145692	0.599201	0.222427	171526.8	178770.2	Up	0.059673	0.874574	Glypican-4 OS=Mus musculus GN=Gpc4 PE=2 SV=2
Pcbp1	P60335	15	15	143229.9	126577.4	135968	119693.7	147662.4	118781.5	139758.6	148145.1	134730	108686.2	0.617558	0.209322	134626.3	130020.3	Down	-0.05022	0.882096	Poly(rC)-binding protein 1 OS=Mus musculus GN=Pcbp1 PE=1 SV=1
Micu1	Q8VCX5-3	2	2	NA	3408	2170	NA	NA	NA	2508	4676	NA	NA	0.600291	0.221638	2789	3592	Up	0.365039	0.875666	Isoform 3 of Calcium uptake protein 1, mitochondrial OS=Mus musculus GN=Micu1
Ccsap	Q8QZT2	4	4	NA	7016.5	1454	3507.5	5073.5	NA	5889.5	1785	4213.5	1929.5	0.618141	0.208912	4262.875	3454.375	Down	-0.3034	0.882096	Centriole, cilia and spindle-associated protein OS=Mus musculus GN=Ccsap PE=2 SV=1
Osbpl3	Q9DBS9	1	1	15194	15298	19903	11695	16251	18082	13923	14260	19496	17199	0.60279	0.219834	15668.2	16592	Up	0.082648	0.876574	Oxysterol-binding protein-related protein 3 OS=Mus musculus GN=Osbpl3 PE=1 SV=2
Safb	D3YXK2	2	2	52967.5	36207.5	96769.5	46296.5	89041.5	86617	75558.5	65186.5	103704.5	35234.5	0.602966	0.219707	64256.5	73260.2	Up	0.189187	0.876574	Scaffold attachment factor B1 OS=Mus musculus GN=Safb PE=1 SV=2
Dnajc6	Q80TZ3-3	10	10	51861.67	94321.22	133131.3	114879.3	115365	108558.3	111481.7	112984.4	108822.1	107065.6	0.603189	0.219547	101911.7	109782.4	Up	0.107327	0.876574	Isoform 3 of Putative tyrosine-protein phosphatase auxilin OS=Mus musculus GN=Dnajc6
Vim	P20152	4	4	133683.7	159195.7	208463	248126.7	285471.3	147208.3	268642	211780	268298.3	235737.3	0.604234	0.218795	206988.1	226333.2	Up	0.128901	0.876574	Vimentin OS=Mus musculus GN=Vim PE=1 SV=3
Hdhd2	Q3UGR5	3	3	644743	499007.7	508739.7	507520.7	528711.7	520616.3	511865.7	542779	615557.3	589312.3	0.604334	0.218723	537744.5	556026.1	Up	0.048232	0.876574	Haloacid dehalogenase-like hydrolase domain-containing protein 2 OS=Mus musculus GN=Hdhd2 PE=1 SV=2
Coro1a	O89053	6	6	129211.3	111528.7	96419.33	86199.67	131784	109694	134680.3	169448.7	85896.67	101651.3	0.606721	0.217011	111028.6	120274.2	Up	0.115396	0.878546	Coronin-1A OS=Mus musculus GN=Coro1a PE=1 SV=5
Calb2	Q08331	5	5	61771.25	71191	61721	66304.25	87609.25	27852	77307.75	63750	75222.25	76910.25	0.621161	0.206796	69719.35	64208.45	Down	-0.1188	0.88272	Calretinin OS=Mus musculus GN=Calb2 PE=1 SV=3
Pgam1	Q9DBJ1	9	9	98366.5	191391	173856.3	141625.3	156977.8	142471	125746.6	164296.5	149535.3	135253	0.621756	0.20638	152443.4	143460.5	Down	-0.08762	0.88272	Phosphoglycerate mutase 1 OS=Mus musculus GN=Pgam1 PE=1 SV=3
Cox6a1	P43024	3	3	126777	81249.5	45492.5	106689	82497.5	88785.5	104480	106109	89551.5	92814.5	0.607209	0.216662	88541.1	96348.1	Up	0.121909	0.878756	Cytochrome c oxidase subunit 6A1, mitochondrial OS=Mus musculus GN=Cox6a1 PE=1 SV=2
Dhrs1	Q99L04	1	1	NA	21203	55039	79713	20810	NA	70342	19143	65059	62496	0.607574	0.216401	44191.25	54260	Up	0.296128	0.878788	Dehydrogenase/reductase SDR family member 1 OS=Mus musculus GN=Dhrs1 PE=2 SV=1
Atp1b2	P14231	4	4	66289	155641.3	174105.7	193472	187104.7	169223	171876.3	180466	169627.7	150451.3	0.609964	0.214696	155322.5	168328.9	Up	0.116015	0.880741	Sodium/potassium-transporting ATPase subunit beta-2 OS=Mus musculus GN=Atp1b2 PE=1 SV=2
Ndrg3	Q9QYF9	1	1	NA	53745	50997	30061	17568	21005	45794	15587	46043	34316	0.622685	0.205732	38092.75	32549	Down	-0.2269	0.882941	Protein NDRG3 OS=Mus musculus GN=Ndrg3 PE=1 SV=1
Eif3j2	Q66JS6	2	2	73209	57412	54288	42065	42430	54038	56753	56789	56703	61127	0.61092	0.214016	53880.8	57082	Up	0.083265	0.880741	Eukaryotic translation initiation factor 3 subunit J-B OS=Mus musculus GN=Eif3j2 PE=1 SV=1
Nefm	P08553	22	22	247828.5	311356.1	312471.7	342257.8	251737.9	275397.8	305449.4	354641.1	285809	305303.4	0.612	0.213249	293130.4	305320.1	Up	0.05878	0.880741	Neurofilament medium polypeptide OS=Mus musculus GN=Nefm PE=1 SV=4
Lgi3	Q8K406	1	1	NA	6180	7339	4544	NA	NA	8103	7790	5044	5572	0.612805	0.212678	6021	6627.25	Up	0.138407	0.880741	Leucine-rich repeat LGI family member 3 OS=Mus musculus GN=Lgi3 PE=1 SV=1
Pnpla8	Q8K1N1	3	3	148200.7	162892.7	190698.7	207011.3	191372.7	194158.7	181123	165225.7	152803.7	174205.3	0.624828	0.20424	180035.2	173503.3	Down	-0.05332	0.88306	Calcium-independent phospholipase A2-gamma OS=Mus musculus GN=Pnpla8 PE=1 SV=1
Cpq	Q9WVJ3	1	1	158444	155216	179519	163435	162448	225316	152294	188873	148257	147301	0.613368	0.212279	163812.4	172408.2	Up	0.073784	0.880741	Carboxypeptidase Q OS=Mus musculus GN=Cpq PE=2 SV=1
Slc12a5	Q91V14	6	6	77297	139487.5	152308.2	150554	172098.3	139216.3	138741.7	165713	149627.3	144045	0.613553	0.212148	138349	147468.7	Up	0.092096	0.880741	Solute carrier family 12 member 5 OS=Mus musculus GN=Slc12a5 PE=1 SV=2
Slc25a46	Q9CQS4	2	2	128814.5	318040	284270.5	268178.5	281888.5	256498.5	260534	332969	232057	298379	0.61401	0.211825	256238.4	276087.5	Up	0.107639	0.880741	Solute carrier family 25 member 46 OS=Mus musculus GN=Slc25a46 PE=1 SV=1
Atp6v1d	P57746	8	8	85430.71	52278.86	76028.71	71444.43	77432	73154.14	91386.29	64615.29	88840	65439.14	0.614423	0.211533	72522.94	76686.97	Up	0.080544	0.880741	V-type proton ATPase subunit D OS=Mus musculus GN=Atp6v1d PE=1 SV=1
Ttyh3	Q6P5F7	2	2	NA	14526.5	25764.5	25584	29953	16732.5	32936.5	27187.5	21639	33392.5	0.616723	0.20991	23957	26377.6	Up	0.138866	0.882096	Protein tweety homolog 3 OS=Mus musculus GN=Ttyh3 PE=1 SV=1
Pycrl	Q9DCC4	1	1	129434	70152	81640	83670	83818	137090	80782	79762	96487	92612	0.617418	0.209421	89742.8	97346.6	Up	0.117334	0.882096	Pyrroline-5-carboxylate reductase 3 OS=Mus musculus GN=Pycrl PE=2 SV=2
Fubp1	Q91WJ8	2	2	54199	47059	51420.5	47726	64001.5	43932.5	48541	57751	54776.5	49519	0.62754	0.202359	52881.2	50904	Down	-0.05498	0.88306	Far upstream element-binding protein 1 OS=Mus musculus GN=Fubp1 PE=1 SV=1
Ywhaz	P63101	19	19	780529.4	622852.5	637336.2	575162.3	619402.4	622974.4	689074.7	592970.3	692015.9	517988.4	0.627694	0.202252	647056.5	623004.7	Down	-0.05465	0.88306	14-3-3 protein zeta/delta OS=Mus musculus GN=Ywhaz PE=1 SV=1
Adcy9	P51830	1	1	30808	35746	26544	33699	37031	17073	40039	27641	37685	29910	0.627992	0.202046	32765.6	30469.6	Down	-0.10481	0.88306	Adenylate cyclase type 9 OS=Mus musculus GN=Adcy9 PE=1 SV=1
Pum1	Q80U78-4	2	2	NA	26180.5	19348	22403	27438	29965	23682.5	13042	24726.5	19195	0.628311	0.201825	23842.38	22122.2	Down	-0.10803	0.88306	Isoform 4 of Pumilio homolog 1 OS=Mus musculus GN=Pum1
Rabgef1	Q9JM13	1	1	172884	244952	256701	242734	265314	342108	231475	218771	253159	212275	0.617656	0.209253	236517	251557.6	Up	0.088945	0.882096	Rab5 GDP/GTP exchange factor OS=Mus musculus GN=Rabgef1 PE=1 SV=1
Prpsap2	Q8R574	3	3	88287	141713	107145.3	150434.3	179754.3	183171.7	102704.7	137871	119829	183556.7	0.618268	0.208823	133466.8	145426.6	Up	0.12381	0.882096	Phosphoribosyl pyrophosphate synthase-associated protein 2 OS=Mus musculus GN=Prpsap2 PE=1 SV=1
Prkce	P16054	3	3	270173.7	469404.3	464908.7	587314.3	559669	250000	518166.3	450925.7	427770.3	518194	0.629068	0.201302	470294	433011.3	Down	-0.11916	0.88306	Protein kinase C epsilon type OS=Mus musculus GN=Prkce PE=1 SV=1
Ubap1	Q8BH48	1	1	95293	105563	92600	59221	82238	109150	83996	100446	76644	90067	0.618471	0.208681	86983	92060.6	Up	0.08185	0.882096	Ubiquitin-associated protein 1 OS=Mus musculus GN=Ubap1 PE=1 SV=1
Rbm38	Q62176	1	1	798444	352335	448033	303044	573870	540678	450712	506341	391207	341191	0.629443	0.201044	495145.2	446025.8	Down	-0.15072	0.88306	RNA-binding protein 38 OS=Mus musculus GN=Rbm38 PE=1 SV=2
Mtfmt	Q9D799	1	1	128068	192088	217396	228559	128155	123410	186604	148258	161128	208942	0.629681	0.200879	178853.2	165668.4	Down	-0.11048	0.88306	Methionyl-tRNA formyltransferase, mitochondrial OS=Mus musculus GN=Mtfmt PE=2 SV=3
Nova1	Q9JKN6	4	4	70891.5	49444.5	69540.75	53873	87268.75	86205.5	69757.25	64008.25	39613.75	44079.5	0.629822	0.200782	66203.7	60732.85	Down	-0.12443	0.88306	RNA-binding protein Nova-1 OS=Mus musculus GN=Nova1 PE=1 SV=2
Rnh1	Q91VI7	1	1	NA	10933	7143	11137	5941	NA	6620	21121	7081	NA	0.61881	0.208443	8788.5	11607.33	Up	0.401348	0.882096	Ribonuclease inhibitor OS=Mus musculus GN=Rnh1 PE=1 SV=1
Hist2h2ab	Q64522	3	3	307272.5	458679	762644	813498	1052368	334170	691323	760145.5	643188	583059	0.630513	0.200306	678892.2	602377.1	Down	-0.17252	0.883063	Histone H2A type 2-B OS=Mus musculus GN=Hist2h2ab PE=1 SV=3
Psd	Q5DTT2	2	2	NA	25806.5	29156	5276.5	35915.5	17408	10370	24065	22363.5	27291	0.631032	0.199949	24038.63	20299.5	Down	-0.24391	0.883266	PH and SEC7 domain-containing protein 1 OS=Mus musculus GN=Psd PE=1 SV=2
Hspa12a	Q8K0U4	7	7	47172	80349.67	113376.7	105324	72650.17	70349	99889.33	81819.83	95679.5	106473.5	0.619764	0.207774	83774.5	90842.23	Up	0.116852	0.882617	Heat shock 70 kDa protein 12A OS=Mus musculus GN=Hspa12a PE=1 SV=1
Raly	Q64012	1	1	72818	90374	115459	84380	129775	34678	144747	93998	80419	87688	0.632165	0.19917	98561.2	88306	Down	-0.15851	0.883529	RNA-binding protein Raly OS=Mus musculus GN=Raly PE=1 SV=3
Scg2	Q03517	8	8	242254.8	147770.5	181247.3	176880.8	186105.8	239623	181054.8	196989	191994.5	174192	0.619864	0.207704	186851.8	196770.7	Up	0.074621	0.882617	Secretogranin-2 OS=Mus musculus GN=Scg2 PE=1 SV=1
Rps26	P62855	1	1	NA	102263	53183	NA	86294	97041	58344	72029	61156	72015	0.634085	0.197853	80580	72117	Down	-0.16008	0.885265	40S ribosomal protein S26 OS=Mus musculus GN=Rps26 PE=2 SV=3
2700060E0	Q9CQE8	4	4	361434.8	360316	244748.5	212770	250465.8	295926	274474	275919	234221	265875.3	0.634158	0.197803	285947	269283.1	Down	-0.08662	0.885265	UPF0568 protein C14orf166 homolog OS=Mus musculus PE=2 SV=1
Camk2a	P11798-2	10	10	102163	108428	107464.4	96238	91576	107137.6	116306	117747.1	115832.3	72798.86	0.62031	0.207391	101173.9	105964.4	Up	0.066742	0.88272	Isoform Alpha KAP of Calcium/calmodulin-dependent protein kinase type II subunit alpha OS=Mus musculus GN=Camk2a
Tom1	O88746	3	3	8221	9491	9296	8029	8493.5	8207	8688.5	11617	8208.5	8654	0.621862	0.206306	8706.1	9075	Up	0.059871	0.88272	Target of Myb protein 1 OS=Mus musculus GN=Tom1 PE=1 SV=1
Prmt5	Q8CIG8	1	1	184427	210825	200064	197581	125559	108291	282040	70201	206957	149728	0.635513	0.196876	183691.2	163443.4	Down	-0.16849	0.885709	Protein arginine N-methyltransferase 5 OS=Mus musculus GN=Prmt5 PE=1 SV=3
Rpl10l	P86048	8	8	525604.1	232041.6	245823.3	200367.5	258276.3	299593.8	238549.8	304568.5	237075.1	227138.3	0.636392	0.196275	292422.5	261385.1	Down	-0.16188	0.886452	60S ribosomal protein L10-like OS=Mus musculus GN=Rpl10l PE=2 SV=1
Ddn	Q80TS7	2	2	NA	52944	62623	46312.5	46933	42338.5	61511.5	64272	64714	44319.5	0.621887	0.206289	52203.13	55431.1	Up	0.086559	0.88272	Dendrin OS=Mus musculus GN=Ddn PE=1 SV=3
Sptb	P15508	10	10	52171.5	111886.8	101559.6	108956.3	94846	83173.8	112357.6	106394.5	108856.4	90426.1	0.622002	0.206208	93884.04	100241.7	Up	0.094531	0.88272	Spectrin beta chain, erythrocytic OS=Mus musculus GN=Sptb PE=1 SV=4
Agpat4	Q8K4X7	1	1	205373	314407	342209	265880	401675	366163	309965	322766	340538	284321	0.622847	0.205619	305908.8	324750.6	Up	0.086231	0.882941	1-acyl-sn-glycerol-3-phosphate acyltransferase delta OS=Mus musculus GN=Agpat4 PE=2 SV=1
Luc7l	Q9CYI4	1	1	150413	167971	114824	139229	118477	180260	171759	135083	125586	118953	0.623633	0.205071	138182.8	146328.2	Up	0.08263	0.88306	Putative RNA-binding protein Luc7-like 1 OS=Mus musculus GN=Luc7l PE=2 SV=2
Rpl14	Q9CR57	4	4	1079710	973200.5	785393.3	834204.8	963628.8	1163400	655305.3	1216856	872237.3	1024917	0.624194	0.20468	927227.5	986542.9	Up	0.089458	0.88306	60S ribosomal protein L14 OS=Mus musculus GN=Rpl14 PE=1 SV=3
Hapln1	Q9QUP5	3	3	196228	200597.7	194020.3	155895	155874	212638.3	149592	221400.7	191169.7	170252.3	0.625505	0.203769	180523	189010.6	Up	0.066284	0.88306	Hyaluronan and proteoglycan link protein 1 OS=Mus musculus GN=Hapln1 PE=2 SV=1
Nectin1	Q9JKF6	2	2	663204	397398	310945	380858	402823.5	435361.5	357608.5	347656	434802	422376	0.641047	0.19311	431045.7	399560.8	Down	-0.10943	0.888879	Poliovirus receptor-related protein 1 OS=Mus musculus GN=Pvrl1 PE=1 SV=3
Igdcc4	Q9EQS9-3	1	1	NA	6512	8375	8935	5054	3808	5057	12975	8561	10635	0.625689	0.203641	7219	8207.2	Up	0.185091	0.88306	Isoform 3 of Immunoglobulin superfamily DCC subclass member 4 OS=Mus musculus GN=Igdcc4
Timm10	P62073	2	2	NA	NA	5546	3910.5	5106.5	NA	NA	NA	10718	3668	0.626534	0.203055	4854.333	7193	Up	0.56732	0.88306	Mitochondrial import inner membrane translocase subunit Tim10 OS=Mus musculus GN=Timm10 PE=1 SV=1
Ppme1	Q8BVQ5	3	3	443182	355706	467325.3	396142.3	343679	388354.3	463439.3	458792.3	437696.7	342085.3	0.627093	0.202668	401206.9	418073.6	Up	0.05941	0.88306	Protein phosphatase methylesterase 1 OS=Mus musculus GN=Ppme1 PE=1 SV=5
Mpp6	Q9JLB0	2	2	414053	231109	314597.5	276385	243516.5	294408	332403.5	277338	378520	293000	0.62723	0.202573	295932.2	315133.9	Up	0.090698	0.88306	MAGUK p55 subfamily member 6 OS=Mus musculus GN=Mpp6 PE=1 SV=1
Sel1l	Q9Z2G6	2	2	40253.5	70240	43939	51033.5	98142	49382	86137	81390	77962	43714	0.627282	0.202537	60721.6	67717	Up	0.157308	0.88306	Protein sel-1 homolog 1 OS=Mus musculus GN=Sel1l PE=2 SV=2
Isoc2a	P85094	3	3	NA	46187	126224	160766	99947	68729	129860	107179	95607	74132	0.644775	0.190592	108281	95101.4	Down	-0.18724	0.890698	Isochorismatase domain-containing protein 2A, mitochondrial OS=Mus musculus GN=Isoc2a PE=1 SV=1
Atp2a2	O55143	12	12	74308.56	98619.44	110211.9	118101.4	123542	103779.6	119817.9	116464.2	111109.3	98160.78	0.628607	0.201621	104956.7	109866.4	Up	0.065966	0.88306	Sarcoplasmic/endoplasmic reticulum calcium ATPase 2 OS=Mus musculus GN=Atp2a2 PE=1 SV=2
Mrpl52	Q9D0Y8	2	2	99863.5	93505.5	110931	128101.5	118618.5	143257	118844	95394	93849.5	64031.5	0.64537	0.190191	110204	103075.2	Down	-0.09648	0.890698	39S ribosomal protein L52, mitochondrial OS=Mus musculus GN=Mrpl52 PE=2 SV=1
Rap2a	Q80ZJ1	1	1	819524	521967	600977	450315	586614	656866	527062	548326	569231	514864	0.645391	0.190177	595879.4	563269.8	Down	-0.08119	0.890698	Ras-related protein Rap-2a OS=Mus musculus GN=Rap2a PE=1 SV=2
Cyb5r3	Q9DCN2	1	1	178362	281453	373116	348556	384823	382139	379577	252253	351473	315225	0.628959	0.201378	313262	336133.4	Up	0.101664	0.88306	NADH-cytochrome b5 reductase 3 OS=Mus musculus GN=Cyb5r3 PE=1 SV=3
Cdk5r1	P61809	1	1	247509	299629	208078	220037	212385	193106	232665	288395	373956	196080	0.629385	0.201084	237527.6	256840.4	Up	0.112777	0.88306	Cyclin-dependent kinase 5 activator 1 OS=Mus musculus GN=Cdk5r1 PE=1 SV=1
Rap2c	Q8BU31	2	2	411440.5	262839.5	302434	226812	295042.5	330053	265437	276601.5	285966.5	259363.5	0.646596	0.189367	299713.7	283484.3	Down	-0.08032	0.890698	Ras-related protein Rap-2c OS=Mus musculus GN=Rap2c PE=1 SV=1
Ppp5c	Q60676	3	3	39511	211703	183430	183931.5	192759.5	133430	121325.5	168142	180364	127122.5	0.646715	0.189287	162267	146076.8	Down	-0.15164	0.890698	Serine/threonine-protein phosphatase 5 OS=Mus musculus GN=Ppp5c PE=1 SV=3
Lrrc7	Q80TE7	13	13	137960.1	208066.6	179832.6	205123.1	270106.6	235690.3	179665.9	187224.7	180547.3	157753.8	0.646738	0.189272	200217.8	188176.4	Down	-0.08948	0.890698	Leucine-rich repeat-containing protein 7 OS=Mus musculus GN=Lrrc7 PE=1 SV=2
Synrg	Q5SV85	2	2	44069.5	41155.5	36933	40645	63780.5	56479.5	38894.5	42705	36255.5	37950	0.647191	0.188968	45316.7	42456.9	Down	-0.09404	0.890843	Synergin gamma OS=Mus musculus GN=Synrg PE=1 SV=1
Atp5l	Q9CPQ8	6	6	422679.5	479781.2	514771.3	619387.5	485835.3	498954.8	446875.5	457176.7	535472.7	497202.5	0.648133	0.188336	504491	487136.4	Down	-0.0505	0.891542	ATP synthase subunit g, mitochondrial OS=Mus musculus GN=Atp5l PE=1 SV=1
Epb41l3	Q9WV92-3	16	16	45072.77	90141.92	81051.31	109557.1	103072	106237.9	84404.46	96717.23	82340.54	90196.08	0.63033	0.200432	85779.02	91979.23	Up	0.100683	0.883063	Isoform 3 of Band 4.1-like protein 3 OS=Mus musculus GN=Epb41l3
Atg5	Q99J83	1	1	157494	218350	291124	258275	325953	252713	242776	287846	266070	279753	0.631347	0.199732	250239.2	265831.6	Up	0.087205	0.883266	Autophagy protein 5 OS=Mus musculus GN=Atg5 PE=1 SV=1
Lyrm7	Q9DA03	1	1	NA	22649	20821	37790	9827	23012	22802	53149	27553	10152	0.632225	0.199128	22771.75	27333.6	Up	0.26343	0.883529	Complex III assembly factor LYRM7 OS=Mus musculus GN=Lyrm7 PE=2 SV=2
Nono	Q99K48	3	3	96621	136138	204032	157993	304232.5	138533	200622	220220.5	230858.5	206929.5	0.647764	0.188594	179803.3	199432.7	Up	0.149482	0.885629	Non-POU domain-containing octamer-binding protein OS=Mus musculus GN=Nono PE=1 SV=3
Reep2	Q8VCD6	4	4	16091.5	23739.75	30610.25	29847.5	33722.5	16015	34984	48813	25938.75	24210.5	0.635333	0.196999	26802.3	29992.25	Up	0.162233	0.885709	Receptor expression-enhancing protein 2 OS=Mus musculus GN=Reep2 PE=2 SV=2
Nefl	P08551	26	26	134872.8	204081	207357.3	173333.1	179960	157739.9	211155.6	211148.2	167039.7	194562	0.637325	0.195639	179920.8	188329.1	Up	0.065893	0.886969	Neurofilament light polypeptide OS=Mus musculus GN=Nefl PE=1 SV=5
Arhgap33	Q80YF9	1	1	184582	256766	297847	236255	344731	303838	316887	307263	206094	269465	0.637455	0.19555	264036.2	280709.4	Up	0.088342	0.886969	Rho GTPase-activating protein 33 OS=Mus musculus GN=Arhgap33 PE=1 SV=1
Gprin1	Q3UNH4	9	9	228536.7	222279.4	156339.1	201178	244088.4	260328.3	192123.9	160383.6	202861.2	184261.6	0.653735	0.184598	210484.3	199991.7	Down	-0.07377	0.895498	G protein-regulated inducer of neurite outgrowth 1 OS=Mus musculus GN=Gprin1 PE=1 SV=2
Srsf1	Q6PDM2-3	2	2	10984	7066	7308	5442	5916	8477	7982	6167	6419	4946	0.654508	0.184085	7343.2	6798.2	Down	-0.11126	0.895498	Isoform 3 of Serine/arginine-rich splicing factor 1 OS=Mus musculus GN=Srsf1
Ppp3ca	P63328	10	10	155219.9	224506.4	255355	238713.5	265925.6	281629.4	240368.8	251106.3	209015.4	215085	0.639654	0.194055	227944.1	239441	Up	0.07099	0.888879	Serine/threonine-protein phosphatase 2B catalytic subunit alpha isoform OS=Mus musculus GN=Ppp3ca PE=1 SV=1
Nap1l1	P28656	7	7	1310098	689341.4	641096.3	617905	928043.6	885022.1	773753.7	795878.1	773175.6	633836.3	0.654765	0.183915	837458.9	772333.2	Down	-0.1168	0.895498	Nucleosome assembly protein 1-like 1 OS=Mus musculus GN=Nap1l1 PE=1 SV=2
Htra2	Q9JIY5	1	1	699683	395464	508718	608676	564387	578652	446059	699325	533828	333988	0.655529	0.183408	555385.6	518370.4	Down	-0.09951	0.896064	Serine protease HTRA2, mitochondrial OS=Mus musculus GN=Htra2 PE=1 SV=2
Ubxn6	Q99PL6	8	8	105917.4	139747.3	143313.1	150109.1	163763.4	118032.9	145606.6	133852.7	142273.3	137964.1	0.656245	0.182934	140570.1	135545.9	Down	-0.05251	0.896565	UBX domain-containing protein 6 OS=Mus musculus GN=Ubxn6 PE=1 SV=1
Ccdc92	Q8VDN4	1	1	353841	342155	376149	338383	351925	343752	382174	427764	341411	317974	0.640456	0.193511	352490.6	362615	Up	0.040854	0.888879	Coiled-coil domain-containing protein 92 OS=Mus musculus GN=Ccdc92 PE=2 SV=1
Hnrnpm	Q9D0E1	8	8	44626.12	91606.25	123332.9	119004.5	224505.1	100895.8	140559.8	157779.5	147916.3	133211.1	0.64046	0.193508	120615	136072.5	Up	0.173966	0.888879	Heterogeneous nuclear ribonucleoprotein M OS=Mus musculus GN=Hnrnpm PE=1 SV=3
Arhgap23	Q69ZH9	3	3	108521.5	89459	89571.5	83173.5	93234.5	100820	89108	93464.5	82390	85910.5	0.657749	0.18194	92792	90338.6	Down	-0.03866	0.896731	Rho GTPase-activating protein 23 OS=Mus musculus GN=Arhgap23 PE=2 SV=2
Snph	Q80U23	3	3	NA	38189	36784	24201	50736.5	17857.5	45350	13759.5	16373.5	21676	0.658114	0.181929	33058	28817.4	Down	-0.19806	0.896731	Syntaphilin OS=Mus musculus GN=Snph PE=2 SV=3
Psmc6	P62334	4	4	323226.3	334505.7	324765	269499.7	257691.7	315466.3	353461.3	291113.7	322275.7	277459.7	0.640695	0.193349	301937.7	311955.3	Up	0.047089	0.888879	26S protease regulatory subunit 10B OS=Mus musculus GN=Psmc6 PE=1 SV=1
Slc27a1	Q60714	1	1	74286	86369	123209	100987	167783	110672	110485	114405	94744	81055	0.658877	0.181196	110526.8	102272.2	Down	-0.11198	0.896849	Long-chain fatty acid transport protein 1 OS=Mus musculus GN=Slc27a1 PE=1 SV=1
Atrn	Q9WU60	1	1	NA	25731	33031	32612	45733	30395	37872	38636	36670	39065	0.641222	0.192992	34276.75	36527.6	Up	0.091757	0.888879	Attractin OS=Mus musculus GN=Atrn PE=2 SV=3
Nptn	P97300-3	5	5	602716	975085.3	694050.3	769333.8	843991	782520.8	796670.5	785267.8	867109.3	817146	0.641255	0.192969	777035.3	809742.9	Up	0.059484	0.888879	Isoform 3 of Neuroplastin OS=Mus musculus GN=Nptn
Epb41l3	Q9WV92-5	15	15	48828.83	97653.75	87805.58	118686.8	111661.3	115091	91438.17	103479.3	89202.25	97712.42	0.642851	0.19189	92927.26	99384.63	Up	0.096921	0.89061	Isoform 5 of Band 4.1-like protein 3 OS=Mus musculus GN=Epb41l3
Snx2	Q9CWK8	3	3	159154	141306.7	151621	127056.3	149527.3	114854.7	168397.7	152240	163832	155834	0.644475	0.190794	145733.1	151031.7	Up	0.051523	0.890698	Sorting nexin-2 OS=Mus musculus GN=Snx2 PE=1 SV=2
Mpi	Q924M7	1	1	127166	205585	224884	215873	181367	227696	188653	220971	182351	183806	0.644584	0.19072	190975	200695.4	Up	0.071624	0.890698	Mannose-6-phosphate isomerase OS=Mus musculus GN=Mpi PE=2 SV=1
Wipf2	Q6PEV3	2	2	NA	37242.5	47037	41652	68371	61847	59643	29258.5	36710.5	33088	0.661258	0.179629	48575.63	44109.4	Down	-0.13915	0.897011	WAS/WASL-interacting protein family member 2 OS=Mus musculus GN=Wipf2 PE=2 SV=1
Fnbp1	Q80TY0-3	1	1	926957	599515	558770	627768	646956	1126934	596709	632542	702199	591538	0.645344	0.190209	671993.2	729984.4	Up	0.119419	0.890698	Isoform 3 of Formin-binding protein 1 OS=Mus musculus GN=Fnbp1
Ptges3	Q9R0Q7	1	1	269532	235446	145505	238847	156015	171819	210137	195430	197174	211187	0.662381	0.178892	209069	197149.4	Down	-0.08469	0.897011	Prostaglandin E synthase 3 OS=Mus musculus GN=Ptges3 PE=1 SV=1
Hcls1	P49710	1	1	1177220	577615	543233	393342	619340	750194	571205	705741	480074	466351	0.662962	0.178511	662150	594833	Down	-0.15467	0.897011	Hematopoietic lineage cell-specific protein OS=Mus musculus GN=Hcls1 PE=1 SV=2
Syt11	Q9R0N3	1	1	455485	439269	464229	362438	434684	451948	465855	488468	411918	217023	0.663121	0.178407	431221	407042.4	Down	-0.08325	0.897011	Synaptotagmin-11 OS=Mus musculus GN=Syt11 PE=2 SV=2
Gm4737	P50247	2	2	81170	96887	77633.5	69059	58452.5	73046.5	69327.5	80037.5	70971.5	74350.5	0.663199	0.178356	76640.4	73546.7	Down	-0.05944	0.897011	Adenosylhomocysteinase OS=Mus musculus GN=Ahcy PE=1 SV=3
Slc25a18	Q9DB41	2	2	125651.5	296525	316260.5	341649.5	435784	263317	330855	272910	284641.5	242244	0.66323	0.178336	303174.1	278793.5	Down	-0.12095	0.897011	Mitochondrial glutamate carrier 2 OS=Mus musculus GN=Slc25a18 PE=2 SV=4

Pkp4	Q68FH0-2	3	3	63739.33	119231.3	152579.3	119347.3	148642	99223.67	122350.7	156512	120606.7	150516.7	0.646438	0.189473	120707.9	129841.9	Up	0.105237	0.890698	Isoform 2 of Plakophilin-4 OS=Mus musculus GN=Pkp4
Ptpn11	P35235	3	3	78406.33	165431.3	239421.3	170404	228689.3	143910	220748.3	246438.3	195352.7	158378.7	0.646557	0.189393	176470.5	192965.6	Up	0.128917	0.890698	Tyrosine-protein phosphatase non-receptor type 11 OS=Mus musculus GN=Ptpn11 PE=1 SV=2
Snapin	Q9Z266	1	1	NA	21511	38505	34367	48457	65029	43879	39653	13968	39532	0.648394	0.188161	35710	40412.2	Up	0.178463	0.891542	SNARE-associated protein Snapin OS=Mus musculus GN=Snapin PE=1 SV=1
Acot7	Q91V12-3	4	4	536127.3	147498	261516	220709	237384.7	274781	263167	246914.7	317570	132809.7	0.664423	0.177555	280647	247048.5	Down	-0.18396	0.897011	Isoform C of Cytosolic acyl coenzyme A thioester hydrolase OS=Mus musculus GN=Acot7
Dock7	Q8R1A4	2	2	75632.5	368618.5	376561	366632.5	377299.5	366034.5	363341.5	313973.5	373015	297536.5	0.649283	0.187566	312948.8	342780.2	Up	0.131357	0.892285	Dedicator of cytokinesis protein 7 OS=Mus musculus GN=Dock7 PE=1 SV=3
Ppp3r1	Q63810	10	10	557321.8	541173.9	424353.6	561605.1	357712	478339.3	434051	406720.4	538416.8	478107.8	0.665697	0.176723	488433.3	467127.1	Down	-0.06435	0.897011	Calcineurin subunit B type 1 OS=Mus musculus GN=Ppp3r1 PE=1 SV=3
Pmm1	O35621	2	2	27419	26187	39416	18906.5	15432	18263	25186.5	22538.5	26423.5	24896.5	0.665895	0.176594	25472.1	23461.6	Down	-0.11862	0.897011	Phosphomannomutase 1 OS=Mus musculus GN=Pmm1 PE=1 SV=1
Ncam1	P13595-3	4	4	70801.67	80912	105883.3	92558.67	109177	92954.33	111422.7	93991.67	103829.3	78642.33	0.651646	0.185988	91866.53	96168.07	Up	0.066019	0.894751	Isoform 3 of Neural cell adhesion molecule 1 OS=Mus musculus GN=Ncam1
Rap2b	P61226	2	2	409762	265579	303357	228428	295172	331008	268112.5	277945.5	288480.5	262189.5	0.666458	0.176227	300459.6	285547.2	Down	-0.07344	0.897011	Ras-related protein Rap-2b OS=Mus musculus GN=Rap2b PE=1 SV=1
Nfx1	B1AY10	1	1	NA	20018	19899	12242	16355	10262	17756	19133	14346	18564	0.666565	0.176157	17128.5	16012.2	Down	-0.09723	0.897011	Transcriptional repressor NF-X1 OS=Mus musculus GN=Nfx1 PE=2 SV=1
Nptx1	Q62443	3	3	259558.7	201165	255770.3	195066.7	226356.7	249786	277815.3	253359.3	211533.7	193055.3	0.651776	0.185902	227583.5	237109.9	Up	0.05916	0.894751	Neuronal pentraxin-1 OS=Mus musculus GN=Nptx1 PE=2 SV=1
Cltb	Q6IRU5-3	2	2	11111	58552	48367.5	45915	48499	25700.5	33998.5	71183	44598	64899	0.652481	0.185432	42488.9	48075.8	Up	0.178225	0.89524	Isoform 3 of Clathrin light chain B OS=Mus musculus GN=Cltb
Rps6	P62754	6	6	286412.7	220045.7	209034.5	170381.5	222193.3	287129.7	202759.8	207687.7	185445.7	162531.8	0.668822	0.174689	221613.5	209110.9	Down	-0.08378	0.898407	40S ribosomal protein S6 OS=Mus musculus GN=Rps6 PE=1 SV=1
Psmd4	O35226	2	2	371335.5	293957	345003.5	269473	355555	390857	315109.5	361678.5	260750	226117.5	0.669135	0.174486	327064.8	310902.5	Down	-0.07311	0.898407	26S proteasome non-ATPase regulatory subunit 4 OS=Mus musculus GN=Psmd4 PE=1 SV=1
Hnrnpul2	Q00PI9	1	1	NA	323738	351131	331601	549163	269506	334320	457277	413372	327241	0.66974	0.174094	388908.3	360343.2	Down	-0.11006	0.898407	Heterogeneous nuclear ribonucleoprotein U-like protein 2 OS=Mus musculus GN=Hnrnpul2 PE=1 SV=2
Adgrg1	Q8K209	1	1	NA	105293	65179	106555	114249	68731	107686	133884	94741	121019	0.653381	0.184833	97819	105212.2	Up	0.105115	0.895498	G-protein coupled receptor 56 OS=Mus musculus GN=Gpr56 PE=1 SV=1
Pde4a	O89084-3	2	2	248910.5	199804.5	188577.5	286314	220664.5	121498.5	186015.5	304752	232693.5	221645.5	0.669862	0.174015	228854.2	213321	Down	-0.1014	0.898407	Isoform 3 of cAMP-specific 3',5'-cyclic phosphodiesterase 4A OS=Mus musculus GN=Pde4a
Fxr2	Q9WVR4	1	1	106127	46763	35563	36933	38828	36583	38447	52915	47708	56773	0.670621	0.173523	52842.8	46485.2	Down	-0.18494	0.898589	Fragile X mental retardation syndrome-related protein 2 OS=Mus musculus GN=Fxr2 PE=1 SV=1
Dnajb12	Q9QYI4	1	1	NA	105212	110344	91338	111590	125692	102529	101680	101091	107491	0.653706	0.184618	104621	107696.6	Up	0.0418	0.895498	DnaJ homolog subfamily B member 12 OS=Mus musculus GN=Dnajb12 PE=2 SV=2
Ccdc124	Q9D8X2	2	2	1201	17869	6384.5	12775.5	10212.5	2136	9039	1799	16827.5	9893.5	0.671049	0.173246	9688.5	7939	Down	-0.28732	0.898589	Coiled-coil domain-containing protein 124 OS=Mus musculus GN=Ccdc124 PE=2 SV=1
Cdkl5	Q3UTQ8	1	1	48519	54210	58679	55091	53969	60262	57131	79702	47181	42191	0.654629	0.184005	54093.6	57293.4	Up	0.082911	0.895498	Cyclin-dependent kinase-like 5 OS=Mus musculus GN=Cdkl5 PE=2 SV=1
Dlgap1	Q9D415	7	7	437029.2	339134.2	320774.5	358483.8	393966.3	411908.8	361999.2	329980.5	391419	287523	0.672057	0.172594	369877.6	356566.1	Down	-0.05288	0.898999	Disks large-associated protein 1 OS=Mus musculus GN=Dlgap1 PE=1 SV=3
Scoc	Q78YZ6-3	3	3	38800.67	48316	30702	48478	52399.33	79126.33	47573	61501	25809.33	29839	0.656735	0.18261	43739.2	48769.73	Up	0.157059	0.896617	Isoform 3 of Short coiled-coil protein OS=Mus musculus GN=Scoc
Eps8	Q08509	3	3	6987	9224	7428.67	8449.67	11942.67	8038	14087	8512	1853.33	6771	0.672999	0.171986	8806.402	7852.266	Down	-0.16544	0.899065	Epidermal growth factor receptor kinase substrate 8 OS=Mus musculus GN=Eps8 PE=1 SV=2
Snap29	Q9ERB0	2	2	93384	66146	46748	45853	57317	87471	57804	65881	49019	17793	0.673158	0.171883	61889.6	55593.6	Down	-0.15478	0.899065	Synaptosomal-associated protein 29 OS=Mus musculus GN=Snap29 PE=2 SV=1
Myo18a	Q9JMH9-5	5	5	73015.5	93190.5	72141	85887.5	66698.5	38484	93481	110304.5	82346	97514.5	0.656982	0.182447	78186.6	84426	Up	0.110766	0.896617	Isoform 5 of Unconventional myosin-XVIIIa OS=Mus musculus GN=Myo18a
Fkbp3	Q62446	2	2	NA	NA	NA	19038	27374	NA	24614	NA	26743	NA	0.658751	0.181279	23206	25678.5	Up	0.146063	0.896849	Peptidyl-prolyl cis-trans isomerase FKBP3 OS=Mus musculus GN=Fkbp3 PE=1 SV=2
Wdr7	Q920I9	6	6	83663.67	127505.2	165393	176331.2	142841.5	125069.8	158845.3	141967.3	174133.5	138352.3	0.658903	0.181179	139146.9	147673.7	Up	0.085804	0.896849	WD repeat-containing protein 7 OS=Mus musculus GN=Wdr7 PE=1 SV=3
Dlgap1	Q9D415-3	6	6	516603.4	402147.2	379792.6	426120	466576.8	489090.2	429139.4	390228.6	464504	340821.4	0.677499	0.169091	438248	422756.7	Down	-0.05192	0.902819	Isoform 3 of Disks large-associated protein 1 OS=Mus musculus GN=Dlgap1
Ythdc2	B2RR83	1	1	160720	82493	148003	77895	158966	142501	131208	121689	94259	92199	0.677731	0.168943	125615.4	116371.2	Down	-0.11028	0.902819	Probable ATP-dependent RNA helicase YTHDC2 OS=Mus musculus GN=Ythdc2 PE=1 SV=1
Cep170b	Q80U49-2	2	2	235454	297215	267152	164177	377378.5	255616.5	297801.5	253627.5	236552.5	215061.5	0.678217	0.168631	268275.3	251731.9	Down	-0.09183	0.902998	Isoform 2 of Centrosomal protein of 170 kDa protein B OS=Mus musculus GN=Cep170b
Ywhag	P61982	11	11	734959.6	612940.8	681553.2	588343.9	525307.2	574947.7	723660.5	565488.8	648798	520315.6	0.678607	0.168382	628620.9	606642.1	Down	-0.05134	0.903048	14-3-3 protein gamma OS=Mus musculus GN=Ywhag PE=1 SV=2
Ildr2	B5TVM2	3	3	NA	41721.67	51965	50406	61126.67	59723	53405	69867	46516	41632.67	0.659822	0.180573	51304.84	54228.73	Up	0.079963	0.897011	Immunoglobulin-like domain-containing receptor 2 OS=Mus musculus GN=Ildr2 PE=2 SV=2
Cth	Q8VCN5	1	1	NA	4830	8694	4145	5235	NA	2560	3463	9820	3648	0.680224	0.167348	5726	4872.75	Down	-0.23279	0.903329	Cystathionine gamma-lyase OS=Mus musculus GN=Cth PE=1 SV=1
Actr2	P61161	4	4	242030.3	254892	387530.8	364738.8	511011	468593.8	370151.3	360384	366505.5	321544.5	0.659955	0.180486	352040.6	377435.8	Up	0.10049	0.897011	Actin-related protein 2 OS=Mus musculus GN=Actr2 PE=1 SV=1
Gdi1	P50396	17	17	300039	253437.3	261619.5	237675.3	206576.9	232947.9	254268.5	254227.4	253296.5	229081.7	0.680699	0.167045	251869.6	244764.4	Down	-0.04128	0.903329	Rab GDP dissociation inhibitor alpha OS=Mus musculus GN=Gdi1 PE=1 SV=3
Psd3	Q2PFD7-3	8	8	1082584	279870.7	344569.7	237774.6	541097	581970.6	363835.6	464167.3	405351.9	316812.7	0.681539	0.166509	497179.2	426427.6	Down	-0.22147	0.903329	Isoform 3 of PH and SEC7 domain-containing protein 3 OS=Mus musculus GN=Psd3
Ddah1	Q9CWS0	9	9	162379.8	145724.5	131271	120785	130431.7	134934	131105.8	154565.8	119501.5	130796.5	0.681645	0.166442	138118.4	134180.7	Down	-0.04173	0.903329	N(G),N(G)-dimethylarginine dimethylaminohydrolase 1 OS=Mus musculus GN=Ddah1 PE=1 SV=3
Mink1	Q9JM52-2	4	4	141646	114814.8	127904.3	126617.5	142985.8	151608.3	145197.5	99413.75	126383.5	106542.8	0.681864	0.166302	130793.7	125829.2	Down	-0.05583	0.903329	Isoform 1 of Misshapen-like kinase 1 OS=Mus musculus GN=Mink1
Krt222	Q8CCX5	1	1	74865	41909	57616	40846	35669	56480	48650	45585	37173	46103	0.681874	0.166296	50181	46798.2	Down	-0.10069	0.903329	Keratin-like protein KRT222 OS=Mus musculus GN=Krt222 PE=2 SV=1
Ccdc85a	Q5SP85-3	1	1	128300	83773	82024	70474	103465	93689	94259	100583	79391	75977	0.682623	0.165819	93607.2	88779.8	Down	-0.07639	0.903329	Isoform 3 of Coiled-coil domain-containing protein 85A OS=Mus musculus GN=Ccdc85a
Smarce1	O54941	1	1	23988	22739	29139	20931	48679	13629	29778	30441	31840	26838	0.682691	0.165776	29095.2	26505.2	Down	-0.13451	0.903329	SWI/SNF-related matrix-associated actin-dependent regulator of chromatin subfamily E member 1 OS=Mus musculus GN=Smarce1 PE=1 SV=1
Hmgb3	O54879	1	1	NA	82574	117759	77502	197581	127158	161623	140916	150122	86638	0.660072	0.180409	118854	133291.4	Up	0.165393	0.897011	High mobility group protein B3 OS=Mus musculus GN=Hmgb3 PE=2 SV=3
Rpsa	P14206	6	6	595618.2	262192.5	461082.5	274563.5	471386.3	485236.5	426658.3	421319.8	328033.3	240030.2	0.6832	0.165452	412968.6	380255.6	Down	-0.11906	0.90353	40S ribosomal protein SA OS=Mus musculus GN=Rpsa PE=1 SV=4
Ubxn1	Q922Y1	1	1	276711	197823	163103	148475	226429	239933	183074	197719	173631	161271	0.683691	0.16514	202508.2	191125.6	Down	-0.08346	0.903713	UBX domain-containing protein 1 OS=Mus musculus GN=Ubxn1 PE=1 SV=1
Txn1	P10639	3	3	162815.7	186839.7	168919	171877.7	150011.7	181235	154359	167556	162471.3	158833	0.684531	0.164607	168092.7	164890.9	Down	-0.02775	0.903939	Thioredoxin OS=Mus musculus GN=Txn PE=1 SV=3
Ccdc115	Q8VE99	2	2	23244	25203	27362	24533.5	38596.5	29452	25150	25947.5	33027	32795	0.660982	0.17981	27787.8	29274.3	Up	0.075183	0.897011	Coiled-coil domain-containing protein 115 OS=Mus musculus GN=Ccdc115 PE=2 SV=1
Eif4a2	P10630	2	2	304442	229073	177706.5	241029.5	298626	362162	259025	263721	237864	207601.5	0.662309	0.178939	250175.4	266074.7	Up	0.088891	0.897011	Eukaryotic initiation factor 4A-II OS=Mus musculus GN=Eif4a2 PE=2 SV=2
Nmt1	O70310	2	2	57815.5	47142.5	55902.5	52155	65515.5	63391.5	67462.5	71760.5	48800	41886	0.663654	0.178058	55706.2	58660.1	Up	0.074542	0.897011	Glycylpeptide N-tetradecanoyltransferase 1 OS=Mus musculus GN=Nmt1 PE=1 SV=1
Rplp1	P47955	4	4	1.37E+07	8380344	6709853	5128789	8892563	9950335	7327575	7968574	6515262	7728794	0.68599	0.163682	8561871	7898108	Down	-0.11642	0.904033	60S acidic ribosomal protein P1 OS=Mus musculus GN=Rplp1 PE=2 SV=1
Srr	Q9QZX7	4	4	38900	43222.25	46908.5	56305.75	63278.75	27253.75	51262.5	44132	52469.75	58812.25	0.68624	0.163524	49723.05	46786.05	Down	-0.08784	0.904033	Serine racemase OS=Mus musculus GN=Srr PE=1 SV=1
Pdk1	Q8BFP9	1	1	160614	346729	373992	378904	319904	354809	325077	337809	320179	337237	0.664116	0.177756	316028.6	335022.2	Up	0.084202	0.897011	[Pyruvate dehydrogenase (acetyl-transferring)] kinase isozyme 1, mitochondrial OS=Mus musculus GN=Pdk1 PE=1 SV=2
Oat	P29758	2	2	281043	224960	212461	230378	168050	287080	188095	175136	148328	252243	0.686914	0.163098	223378.4	210176.4	Down	-0.08789	0.904052	Ornithine aminotransferase, mitochondrial OS=Mus musculus GN=Oat PE=1 SV=1
Eif3a	P23116	2	2	36721	28415.5	28045	26959.5	37130.5	36259.5	28153.5	35980	25172	24600.5	0.687516	0.162717	31454.3	30033.1	Down	-0.0667	0.904052	Eukaryotic translation initiation factor 3 subunit A OS=Mus musculus GN=Eif3a PE=1 SV=5
Caskin1	Q6P9K8	33	33	116848.8	112320.8	115139.5	109457.1	159368.2	143118.8	117496.7	130344.5	117178.1	128809.1	0.664268	0.177657	122626.9	127389.4	Up	0.05497	0.897011	Caskin-1 OS=Mus musculus GN=Caskin1 PE=1 SV=2
Mrps22	Q9CXW2	1	1	49865	89761	72979	92756	59736	75748	75172	80327	71327	82282	0.665318	0.176971	73019.4	76971.2	Up	0.076039	0.897011	28S ribosomal protein S22, mitochondrial OS=Mus musculus GN=Mrps22 PE=2 SV=1
Sf1	Q64213-3	1	1	42417	16379	40739	31853	84230	62763	38744	21057	29629	35687	0.688676	0.161985	43123.6	37576	Down	-0.19867	0.904052	Isoform 3 of Splicing factor 1 OS=Mus musculus GN=Sf1
Has3	O08650	1	1	113025	198391	249904	237721	182555	284860	267713	199100	119157	196420	0.665977	0.176541	196319.2	213450	Up	0.120697	0.897011	Hyaluronan synthase 3 OS=Mus musculus GN=Has3 PE=1 SV=2
Gripap1	Q8VD04	4	4	109483	224577.3	189249.8	166737.8	155133.8	193958	160165.8	162754	165426.3	211717.8	0.666721	0.176056	169036.3	178804.4	Up	0.081049	0.897011	GRIP1-associated protein 1 OS=Mus musculus GN=Gripap1 PE=1 SV=1
Plch2	A2AP18-3	1	1	533247	466106	681581	788411	530844	916595	494485	835220	563002	441939	0.667414	0.175605	600037.8	650248.2	Up	0.115937	0.897472	Isoform 3 of 1-phosphatidylinositol 4,5-bisphosphate phosphodiesterase eta-2 OS=Mus musculus GN=Plch2
Thsd7a	Q69ZU6	1	1	43261	11298	27863	30338	14684	57363	15041	11244	11307	10037	0.690316	0.160952	25488.8	20998.4	Down	-0.27958	0.904052	Thrombospondin type-1 domain-containing protein 7A OS=Mus musculus GN=Thsd7a PE=2 SV=2
Pck2	Q8BH04	3	3	61704	66660	81017.33	96597.33	62531.67	67316.33	71203	78155.67	74775	95432.67	0.669788	0.174063	73702.07	77376.53	Up	0.070191	0.898407	Phosphoenolpyruvate carboxykinase [GTP], mitochondrial OS=Mus musculus GN=Pck2 PE=1 SV=1
Zc3h15	Q3TIV5-2	1	1	70721	47439	56756	64657	45789	54998	95129	44164	68892	45814	0.670759	0.173433	57072.4	61799.4	Up	0.1148	0.898589	Isoform 2 of Zinc finger CCCH domain-containing protein 15 OS=Mus musculus GN=Zc3h15
Cdv3	Q4VAA2	1	1	163000	73674	48629	57602	114584	75398	180180	86082	22936	174603	0.671755	0.172789	91497.8	107839.8	Up	0.237081	0.898999	Protein CDV3 OS=Mus musculus GN=Cdv3 PE=1 SV=2
Rtn3	Q9ES97-5	4	4	NA	46526	34032.5	43364	52382	59852	36715.5	59958.5	47460.5	31751	0.672595	0.172246	44076.13	47147.5	Up	0.097184	0.899065	Isoform 5 of Reticulon-3 OS=Mus musculus GN=Rtn3
Immt	Q8CAQ8-5	16	16	315994.1	362026.4	324934.7	361608.4	341658.9	378476	322018.1	353852.2	299239.8	317810.7	0.693262	0.159103	341244.5	334279.4	Down	-0.02975	0.905515	Isoform 5 of Mitochondrial inner membrane protein OS=Mus musculus GN=Immt
Apool	Q78IK4	2	2	21435	58687	57218	60638	52866	27669	61528	57972	46626	37319	0.693418	0.159005	50168.8	46222.8	Down	-0.11819	0.905515	Apolipoprotein O-like OS=Mus musculus GN=Apool PE=2 SV=1
Nae1	Q8VBW6	1	1	NA	74213	150412	34897	167707	189291	58385	156947	115442	99387	0.675542	0.170348	106807.3	123890.4	Up	0.214055	0.901779	NEDD8-activating enzyme E1 regulatory subunit OS=Mus musculus GN=Nae1 PE=1 SV=1
Samm50	Q8BGH2	1	1	NA	19226	31764	44506	NA	NA	28832	NA	22462	65047	0.676404	0.169794	31832	38780.33	Up	0.284847	0.902458	Sorting and assembly machinery component 50 homolog OS=Mus musculus GN=Samm50 PE=1 SV=1
Synj1	Q8CHC4	14	14	97582.5	141855.8	192042.8	194318.8	205833.6	181292.3	170369.4	176289.5	199460.7	152272.4	0.677357	0.169182	166326.7	175936.9	Up	0.081038	0.902819	Synaptojanin-1 OS=Mus musculus GN=Synj1 PE=1 SV=3
Adk	P55264	4	4	19546.25	78707.5	78336.75	81345.25	79364.25	48644.5	89384.5	78050.25	70894.75	80260.5	0.679094	0.16807	67460	73446.9	Up	0.122669	0.903227	Adenosine kinase OS=Mus musculus GN=Adk PE=1 SV=2
Cacna1a	P97445	2	2	199991	259983.5	232022	237012	257632	211621	259126	313204.5	234088	215105.5	0.680549	0.167141	237328.1	246629	Up	0.05546	0.903329	Voltage-dependent P/Q-type calcium channel subunit alpha-1A OS=Mus musculus GN=Cacna1a PE=1 SV=2
Ppm1a	P49443	2	2	27761	40592	37755	31187	36068	34970	32224.5	30234	38671	31648	0.695221	0.157877	34672.6	33549.5	Down	-0.0475	0.905749	Protein phosphatase 1A OS=Mus musculus GN=Ppm1a PE=1 SV=1
Gnaz	O70443	2	2	420031	239953	371996	337345	372733	306743	429649	357962	368483	355941	0.682695	0.165773	348411.6	363755.6	Up	0.062177	0.903329	Guanine nucleotide-binding protein G(z) subunit alpha OS=Mus musculus GN=Gnaz PE=2 SV=4
Rapgef4	Q9EQZ6-3	1	1	NA	50062	40899	61750	32706	53487	41026	39834	50672	62913	0.684752	0.164467	46354.25	49586.4	Up	0.097243	0.903939	Isoform 3 of Rap guanine nucleotide exchange factor 4 OS=Mus musculus GN=Rapgef4
Thy1	P01831	7	7	1049426	1586921	1514076	1459486	1709146	1459169	1566015	1485076	1597748	1460692	0.68492	0.16436	1463811	1513740	Up	0.048388	0.903939	Thy-1 membrane glycoprotein OS=Mus musculus GN=Thy1 PE=1 SV=1
Eno2	P17183	68	68	239802.2	256993.8	220369.6	225958	210466.3	224031.8	245720.5	242675.5	231003.8	181587.6	0.697104	0.156702	230718	225003.8	Down	-0.03618	0.906482	Gamma-enolase OS=Mus musculus GN=Eno2 PE=1 SV=2
Hist1h2bm	P10854	13	13	316243.1	575190.8	875517.6	788171.5	1568851	147128.8	781098.4	983515.9	903099.1	791546.3	0.698518	0.155822	824794.7	721277.7	Down	-0.19348	0.906978	Histone H2B type 1-M OS=Mus musculus GN=Hist1h2bm PE=1 SV=2
Nptn	P97300	7	7	565086.6	835184.4	622410.2	671998	753867	693213.2	718766	692827.2	738426.2	710659.2	0.685322	0.164105	689709.2	710778.4	Up	0.043411	0.904004	Neuroplastin OS=Mus musculus GN=Nptn PE=1 SV=3
Dld	O08749	25	25	310431.8	336602.5	352257.4	449262.7	316112.5	357431.3	354282.7	338205	337597.6	323682.3	0.699247	0.155369	352933.4	342239.8	Down	-0.04439	0.906978	Dihydrolipoyl dehydrogenase, mitochondrial OS=Mus musculus GN=Dld PE=1 SV=2
Nos1	Q9Z0J4-4	2	2	1535.5	131672.5	139117	106418	112733	122213.5	106965	109068	106088	101636	0.686402	0.163421	98295.2	109194.1	Up	0.151702	0.904033	Isoform NNOS gamma of Nitric oxide synthase, brain OS=Mus musculus GN=Nos1
Sdha	Q8K2B3	21	21	113503.2	178284.5	160234.9	215344	128227.6	158730	158655.5	153142.9	171776.4	194894.6	0.687965	0.162434	159118.8	167439.9	Up	0.073539	0.904052	Succinate dehydrogenase [ubiquinone] flavoprotein subunit, mitochondrial OS=Mus musculus GN=Sdha PE=1 SV=1
Nwd2	Q6P5U7	1	1	7819	4354	5548	5989	4406	4143	7810	2210	8080	8916	0.688558	0.162059	5623.2	6231.8	Up	0.148258	0.904052	Leucine-rich repeat and WD repeat-containing protein KIAA1239 OS=Mus musculus GN=Kiaa1239 PE=1 SV=2
Epb41l2	O70318	5	5	133472.3	239327	183137.7	211769.3	222577.3	180562.7	194783	230625	187341.3	243616.7	0.689276	0.161607	198056.7	207385.7	Up	0.066403	0.904052	Band 4.1-like protein 2 OS=Mus musculus GN=Epb41l2 PE=1 SV=2
9-Sep	O80UG5-2	1	1	778355	322316	420579	393778	381390	357515	482966	358281	428000	493689	0.700767	0.154426	459283.6	424090.2	Down	-0.11501	0.906978	Isoform 2 of Septin-9 OS=Mus musculus GN=Sept9
Eif1ax	Q8BMJ3	2	2	NA	1058	6874	4224	2623.5	NA	4833.5	6003	3021.5	3338	0.6895	0.161466	3694.875	4299	Up	0.218476	0.904052	Eukaryotic translation initiation factor 1A, X-chromosomal OS=Mus musculus GN=Eif1ax PE=2 SV=3
Gatc	Q8CBY0	2	2	32485	37098	35974	36303	28445	57037	31236	34419	28722	30519	0.690146	0.161059	34061	36386.6	Up	0.095286	0.904052	Glutamyl-tRNA(Gln) amidotransferase subunit C, mitochondrial OS=Mus musculus GN=Gatc PE=2 SV=1
Plp1	P60202	1	1	NA	24490	93534	105793	55158	103954	80343	69468	43061	97231	0.690418	0.160888	69743.75	78811.4	Up	0.17634	0.904052	Myelin proteolipid protein OS=Mus musculus GN=Plp1 PE=1 SV=2
Mrpl41	Q9CQN7	1	1	NA	20453	25878	38874	NA	27203	36196	18487	49153	26875	0.690516	0.160826	28401.67	31582.8	Up	0.153163	0.904052	39S ribosomal protein L41, mitochondrial OS=Mus musculus GN=Mrpl41 PE=2 SV=1
Dazap1	Q9JII5	1	1	NA	16384	14669	26038	51695	17510	25285	12963	36297	24738	0.703194	0.152925	27196.5	23358.6	Down	-0.21947	0.906978	DAZ-associated protein 1 OS=Mus musculus GN=Dazap1 PE=2 SV=2
Atp1a1	Q8VDN2	43	43	249533	424431.3	557634.6	546433.3	595018.5	458595.2	479061.8	509809.4	445173.2	341444.7	0.703216	0.152911	474610.1	446816.8	Down	-0.08706	0.906978	Sodium/potassium-transporting ATPase subunit alpha-1 OS=Mus musculus GN=Atp1a1 PE=1 SV=1
Sncg	Q9Z0F7	2	2	247713.5	562668	413941.5	554575.5	352253.5	376654	485080.5	420133	423263	569702.5	0.690649	0.160743	426230.4	454966.6	Up	0.094127	0.904052	Gamma-synuclein OS=Mus musculus GN=Sncg PE=1 SV=1
Ywhah	P68510	11	11	809897.8	671617.8	737567.3	648968.8	570329.6	621184.1	798415.2	634595.7	704240.3	567986.1	0.703771	0.152569	687676.2	665284.3	Down	-0.04776	0.906978	14-3-3 protein eta OS=Mus musculus GN=Ywhah PE=1 SV=2
Cap1	P40124	6	6	127854	115353.7	75829.67	98534.67	88776	92837.5	108549.7	82703.67	99305.5	154969.8	0.693135	0.159182	101269.6	107673.2	Up	0.088458	0.905515	Adenylyl cyclase-associated protein 1 OS=Mus musculus GN=Cap1 PE=1 SV=4
Tln2	Q71LX4	15	15	17547.64	53599.09	66902.55	61235.55	61719.64	47130.82	59018.27	64473.55	48185.82	62185.18	0.694033	0.15862	52200.89	56198.73	Up	0.106463	0.905515	Talin-2 OS=Mus musculus GN=Tln2 PE=1 SV=3
Hist1h2bk	Q8CGP1	12	12	338091.2	626576.9	951771.3	858057.6	1702798	159398.4	849931.3	1071213	983701.3	863254.8	0.705388	0.151572	895459.1	785499.8	Down	-0.18902	0.908458	Histone H2B type 1-K OS=Mus musculus GN=Hist1h2bk PE=1 SV=3
Trappc1	Q5NCF2	1	1	151151	107608	145132	132121	128468	64582	156402	153747	93631	155032	0.705845	0.151291	132896	124678.8	Down	-0.09208	0.908458	Trafficking protein particle complex subunit 1 OS=Mus musculus GN=Trappc1 PE=1 SV=1
Eef1d	P57776-3	8	8	271534.4	202853.1	143340	168625.4	182031.3	202186.3	192834.6	204535.3	201300	213867.6	0.694123	0.158564	193676.9	202944.7	Up	0.067435	0.905515	Isoform 3 of Elongation factor 1-delta OS=Mus musculus GN=Eef1d
Atp1a2	Q6PIE5	52	52	212401.3	484991.1	632625.7	573892	596426.9	535399.1	539854.1	604661.8	523994.4	462088.4	0.694239	0.158491	500067.4	533199.5	Up	0.092553	0.905515	Sodium/potassium-transporting ATPase subunit alpha-2 OS=Mus musculus GN=Atp1a2 PE=1 SV=1
Sdcbp	O08992	2	2	11590	36467	20439.5	16965	23894.5	18154	22362	14276	25675.5	19786	0.706775	0.150719	21871.2	20050.7	Down	-0.12538	0.908458	Syntenin-1 OS=Mus musculus GN=Sdcbp PE=1 SV=1
Nubp2	Q9R061	1	1	NA	NA	14012	22112	16236	NA	10833	13885	13872	24967	0.707675	0.150166	17453.33	15889.25	Down	-0.13545	0.908583	Cytosolic Fe-S cluster assembly factor NUBP2 OS=Mus musculus GN=Nubp2 PE=1 SV=1
Rgs17	Q9QZB0	1	1	NA	278807	185353	168807	166807	216657	233712	254197	184457	174314	0.694552	0.158295	199943.5	212667.4	Up	0.089007	0.905515	Regulator of G-protein signaling 17 OS=Mus musculus GN=Rgs17 PE=1 SV=1
Sirpa	P97797-3	3	3	107681.3	119957.7	99362	90133	151258	135121	125092.7	107674.3	133471.3	94054.67	0.694593	0.15827	113678.4	119082.8	Up	0.067007	0.905515	Isoform 3 of Tyrosine-protein phosphatase non-receptor type substrate 1 OS=Mus musculus GN=Sirpa
1810026J2	Q8BGX2	2	2	14835	154575	155669	173419.5	214530	114781.5	181014	162061.5	172603	156463.5	0.695787	0.157524	142605.7	157384.7	Up	0.142264	0.905749	Uncharacterized protein C19orf52 homolog OS=Mus musculus PE=2 SV=1
Ganab	Q8BHN3	6	6	578987.3	305139	369258.8	385753.8	469100	459703.3	404925.8	431771	391934.3	316189.8	0.709415	0.1491	421647.8	400904.9	Down	-0.07278	0.908706	Neutral alpha-glucosidase AB OS=Mus musculus GN=Ganab PE=1 SV=1
Cnpy3	Q9DAU1	3	3	360447.7	223901.7	236859	216547.7	245716.7	300403.3	279491	309079	247795	211747.7	0.695833	0.157495	256694.5	269703.2	Up	0.07132	0.905749	Protein canopy homolog 3 OS=Mus musculus GN=Cnpy3 PE=1 SV=1
Ppp6r2	Q8R3Q2	1	1	NA	6211	2264	4514	NA	7513	3765	4700	3852	4644	0.696266	0.157225	4329.667	4894.8	Up	0.176994	0.905852	Serine/threonine-protein phosphatase 6 regulatory subunit 2 OS=Mus musculus GN=Ppp6r2 PE=1 SV=1
Ap2a1	P17426	13	13	445610.2	224809	339555.5	286769.8	294161	355928.3	351603.6	357634.3	296175.3	309454.7	0.698751	0.155678	318181.1	334159.2	Up	0.070688	0.906978	AP-2 complex subunit alpha-1 OS=Mus musculus GN=Ap2a1 PE=1 SV=1
Hgs	Q99LI8	4	4	135024.3	101034.7	99570.67	84233	114187.7	145091.3	125068.3	96856.67	101978.3	91402.33	0.699704	0.155086	106810.1	112079.4	Up	0.069473	0.906978	Hepatocyte growth factor-regulated tyrosine kinase substrate OS=Mus musculus GN=Hgs PE=1 SV=2
Eef1a2	P62631	24	24	307399.3	332616.1	356832.4	297783.5	331838.3	381726.8	316271.2	264357.5	325038.6	296550	0.711125	0.148054	325293.9	316788.8	Down	-0.03822	0.908706	Elongation factor 1-alpha 2 OS=Mus musculus GN=Eef1a2 PE=1 SV=1
Stx7	O70439	4	4	67554	132338	143050	124343.3	155167.3	161496.8	120858	154680.8	112077.3	110718.8	0.699894	0.154968	124490.5	131966.3	Up	0.084134	0.906978	Syntaxin-7 OS=Mus musculus GN=Stx7 PE=1 SV=3
App	P12023-3	3	3	340517.3	309301.7	241193.3	240848.7	239579	312683.7	247056.7	341196.7	275587.3	250705	0.700734	0.154447	274288	285445.9	Up	0.057526	0.906978	Isoform APP751 of Amyloid beta A4 protein OS=Mus musculus GN=App
Rps8	P62242	3	3	8792.67	9590	31714.33	28154	35252.33	11903.33	28770	11679	38466.67	6932.67	0.712057	0.147485	22700.67	19550.33	Down	-0.21554	0.908706	40S ribosomal protein S8 OS=Mus musculus GN=Rps8 PE=1 SV=2
Ankrd63	A2ARS0	1	1	225209	223629	181188	188398	275839	293797	197598	250297	192262	211061	0.700749	0.154438	218852.6	229003	Up	0.065407	0.906978	Ankyrin repeat domain-containing protein 63 OS=Mus musculus GN=Ankrd63 PE=4 SV=1
Smarcc1	P97496	1	1	45799	65552	102739	89706	168152	44182	106007	92926	152725	131645	0.70101	0.154276	94389.6	105497	Up	0.160502	0.906978	SWI/SNF complex subunit SMARCC1 OS=Mus musculus GN=Smarcc1 PE=1 SV=2
Ano10	Q8BH79-3	1	1	69975	86789	83630	124031	159690	61930	94936	126705	95136	108119	0.713831	0.146405	104823	97365.2	Down	-0.10648	0.90982	Isoform 3 of Anoctamin-10 OS=Mus musculus GN=Ano10
Cntnap2	Q9CPW0	2	2	9332.5	57785.5	47391.5	64434.5	48725.5	43622.5	50583	37477	51351.5	66195.5	0.701376	0.154049	45533.9	49845.9	Up	0.130534	0.906978	Contactin-associated protein-like 2 OS=Mus musculus GN=Cntnap2 PE=1 SV=2
Ppp2r1b	Q7TNP2	5	5	300000.4	255157.8	284467.6	273031.4	197563.4	252560.4	300433.4	272354.8	279163	246182.8	0.701972	0.15368	262044.1	270138.9	Up	0.043892	0.906978	Serine/threonine-protein phosphatase 2A 65 kDa regulatory subunit A beta isoform OS=Mus musculus GN=Ppp2r1b PE=1 SV=2
Bphl	Q8R164	2	2	311421.5	369990.5	344012	420054.5	296119.5	346985.5	355503.5	359247	361167.5	364236.5	0.702482	0.153365	348319.6	357428	Up	0.037241	0.906978	Valacyclovir hydrolase OS=Mus musculus GN=Bphl PE=1 SV=1
Pex11b	Q9Z210	1	1	217284	188162	198667	173720	179756	235914	199481	196715	176102	175889	0.703565	0.152696	191517.8	196820.2	Up	0.0394	0.906978	Peroxisomal membrane protein 11B OS=Mus musculus GN=Pex11b PE=1 SV=1
Tst	P52196	4	4	373417.8	298940.8	303344.8	296110.5	271301.5	306376	309278.5	304343.3	233148	343227.8	0.716576	0.144738	308623.1	299274.7	Down	-0.04438	0.910355	Thiosulfate sulfurtransferase OS=Mus musculus GN=Tst PE=1 SV=3
Gsr	P47791	1	1	185276	224500	171559	200592	76245	91907	282767	91293	202330	290368	0.703779	0.152564	171634.4	191733	Up	0.15976	0.906978	Glutathione reductase, mitochondrial OS=Mus musculus GN=Gsr PE=1 SV=3
Idh3a	Q9D6R2-2	9	9	262086.1	429074	370127.9	508227.4	313332.8	402342.4	373216.2	370409.9	396971.9	430057.9	0.703855	0.152517	376569.6	394599.7	Up	0.067473	0.906978	Isoform 2 of Isocitrate dehydrogenase [NAD] subunit alpha, mitochondrial OS=Mus musculus GN=Idh3a
Celf2	Q9Z0H4-2	6	6	758288	561079.3	522397.3	552260.8	775021	649036.3	578937.3	660394.8	608109	561776.3	0.717932	0.143917	633809.3	611650.8	Down	-0.05134	0.910355	Isoform 2 of CUGBP Elav-like family member 2 OS=Mus musculus GN=Celf2
Pla2g7	Q60963	1	1	78496	79147	90594	47422	104653	146546	92299	40523	55686	19143	0.718663	0.143475	80062.4	70839.4	Down	-0.17657	0.910355	Platelet-activating factor acetylhydrolase OS=Mus musculus GN=Pla2g7 PE=2 SV=2
Hspa8	P63017	74	74	978791.8	713378.5	689054.4	670145	787226.2	814683.9	709297.5	747099.8	708310.5	745488.7	0.718719	0.143441	767719.2	744976.1	Down	-0.04338	0.910355	Heat shock cognate 71 kDa protein OS=Mus musculus GN=Hspa8 PE=1 SV=1
Zfp706	Q9D115	1	1	325932	144276	175154	146796	216389	219583	212178	168514	155019	185080	0.719249	0.143121	201709.4	188074.8	Down	-0.10097	0.910355	Zinc finger protein 706 OS=Mus musculus GN=Znf706 PE=2 SV=1
Lmnb2	P21619-2	4	4	8420	52777.75	91160.5	61889.25	113464.5	30437.75	89654.75	80313.5	91264.5	77475	0.706392	0.150954	65542.4	73829.1	Up	0.171761	0.908458	Isoform B3 of Lamin-B2 OS=Mus musculus GN=Lmnb2
Csf1	P07141	1	1	95138	61523	74679	44751	75809	121251	70598	52204	40938	33527	0.719872	0.142745	70380	63703.6	Down	-0.14379	0.910355	Macrophage colony-stimulating factor 1 OS=Mus musculus GN=Csf1 PE=1 SV=2
Phb	P67778	14	14	58221	142960.1	95563.21	128294.3	98826	109606.8	96855.14	92490.64	92060.43	104097.8	0.720068	0.142626	104772.9	99022.16	Down	-0.08144	0.910355	Prohibitin OS=Mus musculus GN=Phb PE=1 SV=1
Hnrnpa2b1	O88569	12	12	46086.91	36138.82	87586.55	56479.55	119118.2	11180.27	94936	94862.36	91108.73	97379.82	0.706709	0.150759	69082	77893.44	Up	0.173192	0.908458	Heterogeneous nuclear ribonucleoproteins A2/B1 OS=Mus musculus GN=Hnrnpa2b1 PE=1 SV=2
Cntn3	Q07409	2	2	NA	39726.5	72989.5	55550	65645	60760.5	53938.5	75520	56222.5	62151.5	0.707708	0.150146	58477.75	61718.6	Up	0.077818	0.908583	Contactin-3 OS=Mus musculus GN=Cntn3 PE=1 SV=2
Fam81a	Q3UXZ6	1	1	157282	228577	148214	161684	222982	209648	169398	186915	174744	139276	0.720357	0.142452	183747.8	175996.2	Down	-0.06218	0.910355	Protein FAM81A OS=Mus musculus GN=Fam81a PE=2 SV=2
Slc12a2	P55012	4	4	2712.5	42064.25	54753.75	68916.5	55953.25	30879.5	69559	53662	49327	46395.25	0.707936	0.150006	44880.05	49964.55	Up	0.154831	0.908583	Solute carrier family 12 member 2 OS=Mus musculus GN=Slc12a2 PE=1 SV=2
Myl9	Q9CQ19	1	1	NA	NA	63706	202729	NA	NA	NA	67274	174846	56775	0.721059	0.142029	133217.5	99631.67	Down	-0.41911	0.910355	Myosin regulatory light polypeptide 9 OS=Mus musculus GN=Myl9 PE=1 SV=3
Txndc5	Q91W90	5	5	236993.5	213951.5	221907	162950.5	329097.5	216348	217491.5	259340	186772	229534.5	0.721206	0.141941	232980	221897.2	Down	-0.07031	0.910355	Thioredoxin domain-containing protein 5 OS=Mus musculus GN=Txndc5 PE=1 SV=2
Snx18	Q91ZR2	2	2	85514	58709.5	99028	124742	134106.5	101140	102501	107698	70424.5	92102.5	0.721685	0.141652	100420	94773.2	Down	-0.0835	0.910355	Sorting nexin-18 OS=Mus musculus GN=Snx18 PE=1 SV=1
Rps24	P62849-3	3	3	187606.3	166964.7	217107	182368.7	291849	207558	214102	214092.3	191083	174771	0.721748	0.141614	209179.1	200321.3	Down	-0.06242	0.910355	Isoform 3 of 40S ribosomal protein S24 OS=Mus musculus GN=Rps24
Arhgap44	Q5SSM3-3	4	4	75505	76367.25	70345.25	67687.5	71112	77901.25	69049.5	75571.75	65030	79739	0.709274	0.149186	72203.4	73458.3	Up	0.024859	0.908706	Isoform 3 of Rho GTPase-activating protein 44 OS=Mus musculus GN=Arhgap44
Tollip	Q9QZ06	3	3	399209.7	316775.7	268365.7	287852	310000	311363	326817.7	319151.3	316441	355151.3	0.70968	0.148937	316440.6	325784.9	Up	0.041985	0.908706	Toll-interacting protein OS=Mus musculus GN=Tollip PE=1 SV=1
Gnb1	P62874	16	16	817874.6	797305.8	863651.9	762035.5	853180.1	946902.9	752187.9	846573	796810.1	824326.9	0.709816	0.148854	818809.6	833360.2	Up	0.025412	0.908706	Guanine nucleotide-binding protein G(I)/G(S)/G(T) subunit beta-1 OS=Mus musculus GN=Gnb1 PE=1 SV=3
Plcxd3	Q8BLJ3	1	1	NA	22669	56291	21919	34812	44039	58014	53340	24672	12422	0.710046	0.148714	33922.75	38497.4	Up	0.182508	0.908706	PI-PLC X domain-containing protein 3 OS=Mus musculus GN=Plcxd3 PE=2 SV=1
Wbp11	Q923D5	2	2	340659.5	186689.5	275693	170958	318974.5	250424	268251.5	267451	245251.5	193458.5	0.726462	0.138787	258594.9	244967.3	Down	-0.0781	0.914051	WW domain-binding protein 11 OS=Mus musculus GN=Wbp11 PE=1 SV=2
Fuom	Q8R2K1-4	1	1	30086	52367	54953	26858	15796	27100	55139	45016	42079	28555	0.710946	0.148163	36012	39577.8	Up	0.136214	0.908706	Isoform 4 of Fucose mutarotase OS=Mus musculus GN=Fuom
Cct3	P80318	4	4	137584.3	166679.7	144436.7	139287.3	143945.3	170676.3	122893.3	158378.3	132078	128501.7	0.728366	0.13765	146386.7	142505.5	Down	-0.03877	0.91543	T-complex protein 1 subunit gamma OS=Mus musculus GN=Cct3 PE=1 SV=1
Ptprs	B0V2N1-4	2	2	NA	73366	76081	75556.5	46766	18212.5	74428	86271.5	75562	60074	0.72863	0.137493	67942.38	62909.6	Down	-0.11103	0.91543	Isoform 4 of Receptor-type tyrosine-protein phosphatase S OS=Mus musculus GN=Ptprs
Psmc2	P46471	3	3	34144.5	56549.5	54525	50184.5	80311.5	52156	45084.5	54998.5	58927	50339	0.730301	0.136498	55143	52301	Down	-0.07634	0.916258	26S protease regulatory subunit 7 OS=Mus musculus GN=Psmc2 PE=1 SV=5
Eps15l1	Q60902-3	2	2	193630	265018	208910	247547.5	290667.5	290944	187869	268693.5	252589.5	253125	0.711517	0.147815	241154.6	250644.2	Up	0.055683	0.908706	Isoform 3 of Epidermal growth factor receptor substrate 15-like 1 OS=Mus musculus GN=Eps15l1
Timm8a1	Q9WVA2	2	2	63010	135071.5	127981.5	151309	153506.5	107883.5	144156	112462	100023	133010.5	0.730361	0.136462	126175.7	119507	Down	-0.07834	0.916258	Mitochondrial import inner membrane translocase subunit Tim8 A OS=Mus musculus GN=Timm8a1 PE=1 SV=1
Scrib	Q80U72-3	1	1	198912	150955	79710	120121	133737	84161	127860	135810	134299	159989	0.731197	0.135966	136687	128423.8	Down	-0.08996	0.9168	Isoform 3 of Protein scribble homolog OS=Mus musculus GN=Scrib
Myh11	O08638	3	3	NA	5429	5278.33	8838.67	23902	5814	9898.67	9296.67	15422.33	5068	0.731508	0.135781	10862	9099.934	Down	-0.25536	0.9168	Myosin-11 OS=Mus musculus GN=Myh11 PE=1 SV=1
Prdx2	Q61171	5	5	301840.5	196442.3	158705.5	152121.8	163684.3	218192.8	158772.3	173565.5	182277	186541	0.732498	0.135194	194558.9	183869.7	Down	-0.08152	0.917592	Peroxiredoxin-2 OS=Mus musculus GN=Prdx2 PE=1 SV=3
Eps15l1	Q60902-4	3	3	193630	265018	208910	247547.5	290667.5	290944	187869	268693.5	252589.5	253125	0.711517	0.147815	241154.6	250644.2	Up	0.055683	0.908706	Isoform 4 of Epidermal growth factor receptor substrate 15-like 1 OS=Mus musculus GN=Eps15l1
Smarcc2	Q6PDG5	3	3	24863.5	34540.5	53014	46110.5	85982	23661.5	54475.5	48045	77964	67012	0.712218	0.147387	48902.1	54231.6	Up	0.149237	0.908706	SWI/SNF complex subunit SMARCC2 OS=Mus musculus GN=Smarcc2 PE=1 SV=2
Srsf3	P84104	1	1	338377	544877	675619	508896	621823	565048	648668	625650	455641	524473	0.712287	0.147345	537918.4	563896	Up	0.068042	0.908706	Serine/arginine-rich splicing factor 3 OS=Mus musculus GN=Srsf3 PE=1 SV=1
Cadps	Q80TJ1-4	8	8	15366	59471.29	64759.57	61298.29	68547.29	63215.14	51067.43	65822	53944.43	55135	0.714608	0.145932	53888.49	57836.8	Up	0.102011	0.90982	Isoform 4 of Calcium-dependent secretion activator 1 OS=Mus musculus GN=Cadps
Rbp1	Q00915	1	1	682547	364520	394793	408221	451156	544822	450855	465644	409450	309281	0.735905	0.133178	460247.4	436010.4	Down	-0.07805	0.918866	Retinol-binding protein 1 OS=Mus musculus GN=Rbp1 PE=2 SV=2
Fam21	Q6PGL7	1	1	138057	172927	138460	203452	152382	230741	181667	134587	138048	161075	0.714792	0.14582	161055.6	169223.6	Up	0.071372	0.90982	WASH complex subunit FAM21 OS=Mus musculus GN=Fam21 PE=1 SV=1

Ppm1h	Q3UYC0	3	3	259262.3	498269.3	559086.3	542259.7	635992	675015.7	532067	556463	476717.7	403215.7	0.714932	0.145735	498973.9	528695.8	Up	0.083473	0.90982 Protein phosphatase 1H OS=Mus musculus GN=Ppm1h PE=1 SV=1
Ints3	Q7TPD0	2	2	11606	7997.5	10166.5	7047	12508	8089	11083.5	12993	9548.5	10114.5	0.714934	0.145734	9865	10365.7	Up	0.071427	0.90982 Integrator complex subunit 3 OS=Mus musculus GN=Ints3 PE=2 SV=2
Ptprn2	P80560	3	3	125981	116668.3	121558	127897.7	139236.3	166985.3	139536.7	106822	122733	116757.7	0.716819	0.14459	126268.3	130566.9	Up	0.048297	0.910355 Receptor-type tyrosine-protein phosphatase N2 OS=Mus musculus GN=Ptprn2 PE=1 SV=2
Mug1	P28665	1	1	NA	105156	53654	71744	61459	43431	71375	105403	80218	93850	0.717694	0.144061	73003.25	78855.4	Up	0.111249	0.910355 Murinoglobulin-1 OS=Mus musculus GN=Mug1 PE=1 SV=3
Hnrnpa2b:	O88569-3	11	11	50695.6	39433.5	95892.3	61984.9	130352.4	11445.6	104005.3	102831.5	99872.3	106376.5	0.719284	0.1431	75671.74	84906.24	Up	0.166116	0.910355 Isoform 3 of Heterogeneous nuclear ribonucleoproteins A2/B1 OS=Mus musculus GN=Hnrnpa2b1
Mink1	Q9JM52	5	5	122173	104024.4	111334.6	109998.4	122931	132208	126109.4	90381.2	109825.4	96332.2	0.739083	0.131307	114092.3	110971.2	Down	-0.04002	0.918866 Misshapen-like kinase 1 OS=Mus musculus GN=Mink1 PE=1 SV=3
Snx15	Q91WE1	4	4	43745.75	30413.25	61172	45773	64399.25	56749.5	36975.75	40038.25	48326	50960.25	0.739542	0.131037	49100.65	46609.95	Down	-0.0751	0.918866 Sorting nexin-15 OS=Mus musculus GN=Snx15 PE=2 SV=1
Rpl26	P61255	9	9	790328.6	356861	376497	323086.7	425063.1	481973.3	439040.9	452179.6	367325.2	375198.9	0.739896	0.130829	454367.3	423143.6	Down	-0.10271	0.918866 60S ribosomal protein L26 OS=Mus musculus GN=Rpl26 PE=1 SV=1
Capn2	O08529	1	1	74959	42929	59741	53602	21054	63819	43043	60784	60108	43458	0.720124	0.142593	50457	54242.4	Up	0.104367	0.910355 Calpain-2 catalytic subunit OS=Mus musculus GN=Capn2 PE=2 SV=4
Cops8	Q8VBV7	1	1	571011	303811	404911	454564	570381	619354	439789	572286	417744	383090	0.720311	0.14248	460935.6	486452.6	Up	0.077734	0.910355 COP9 signalosome complex subunit 8 OS=Mus musculus GN=Cops8 PE=1 SV=1
Cntn5	P68500	1	1	NA	NA	19115	32416	22255	42013	20857	15325	21948	11296	0.74026	0.130616	24595.33	22287.8	Down	-0.14213	0.918866 Contactin-5 OS=Mus musculus GN=Cntn5 PE=1 SV=2
Tpt1	P63028	4	4	295373.5	249754.3	198888	234512.5	156947.5	262139.3	258221	211184	234576	217526.3	0.720477	0.14238	227095.2	236729.3	Up	0.059941	0.910355 Translationally-controlled tumor protein OS=Mus musculus GN=Tpt1 PE=1 SV=1
Tbc1d10b	Q8BHL3	4	4	44239.5	75394.75	64563.25	67343	103233.3	72229.25	58934.5	69112	74154.75	62791.5	0.740327	0.130576	70954.75	67444.4	Down	-0.0732	0.918866 TBC1 domain family member 10B OS=Mus musculus GN=Tbc1d10b PE=1 SV=2
Uap1l1	Q3TW96	1	1	8794	14300	19156	6678	10546	14098	11628	12380	11769	13883	0.722612	0.141095	11894.8	12751.6	Up	0.100347	0.910996 UDP-N-acetylhexosamine pyrophosphorylase-like protein 1 OS=Mus musculus GN=Uap1l1 PE=2 SV=1
Ablim1	Q8K4G5	2	2	137041.5	114813.5	117242.5	109550.5	117991	144699	124455.5	122783.5	105832.5	113658.5	0.722999	0.140862	119327.8	122285.8	Up	0.035327	0.911036 Actin-binding LIM protein 1 OS=Mus musculus GN=Ablim1 PE=1 SV=1
Soga1	E1U8D0	2	2	15583.5	5751.5	8593.5	6236.5	4439.5	6531	8115	6516	17132	7464	0.725045	0.139635	8120.9	9151.6	Up	0.172384	0.913165 Protein SOGA1 OS=Mus musculus GN=Soga1 PE=1 SV=3
Vps35	Q9EQH3	1	1	115660	194343	273486	221271	255097	202782	248875	237009	234511	191619	0.726444	0.138798	211971.4	222959.2	Up	0.07291	0.914051 Vacuolar protein sorting-associated protein 35 OS=Mus musculus GN=Vps35 PE=1 SV=1
Actr3b	Q641P0	1	1	NA	3696	2919	8486	3185	3184	2389	2665	7718	4101	0.743837	0.128522	4571.5	4011.4	Down	-0.18856	0.919027 Actin-related protein 3B OS=Mus musculus GN=Actr3b PE=2 SV=1
Dnaja1	P63037	2	2	589801	583161.5	468162.5	444272.5	346836.5	505991	451554	525759.5	556191.5	482671	0.728094	0.137813	486446.8	504433.4	Up	0.052382	0.91543 DnaJ homolog subfamily A member 1 OS=Mus musculus GN=Dnaja1 PE=1 SV=1
Rps6kc1	Q8BLK9-3	1	1	NA	7881	7457	11932	9853	NA	5121	9193	11996	14012	0.730344	0.136473	9280.75	10080.5	Up	0.119254	0.916258 Isoform 3 of Ribosomal protein S6 kinase delta-1 OS=Mus musculus GN=Rps6kc1
Pgd	Q9DCD0	1	1	270666	265600	376070	262772	229652	240417	344742	283154	295715	177270	0.744318	0.128241	280892	268259.6	Down	-0.06669	0.919027 6-phosphogluconate dehydrogenase, decarboxylating OS=Mus musculus GN=Pgd PE=1 SV=3
Rpl6	P47911	6	6	1314293	1060178	844850.2	811824.7	883355.2	1054797	1013894	1045407	842744	770937.7	0.744458	0.12816	982900.2	945555.8	Down	-0.05588	0.919027 60S ribosomal protein L6 OS=Mus musculus GN=Rpl6 PE=1 SV=3
Arcn1	Q5XJY5	1	1	165117	288887	191795	218533	221557	89880	260353	245996	263350	301492	0.732927	0.134939	217177.8	232214.2	Up	0.09658	0.917681 Coatomer subunit delta OS=Mus musculus GN=Arcn1 PE=1 SV=2
H2-Ke6	P50171	2	2	142950	115615.5	125254.5	119976	135462.5	165856	126638.5	141338.5	115449	109958.5	0.73364	0.134517	127851.7	131868.1	Up	0.044624	0.918133 Estradiol 17-beta-dehydrogenase 8 OS=Mus musculus GN=Hsd17b8 PE=1 SV=2
Pfas	Q5SUR0	1	1	85993	316307	335335	372395	305798	255466	307376	285553	374374	290185	0.734699	0.133891	283165.6	302590.8	Up	0.095722	0.918866 Phosphoribosylformylglycinamidine synthase OS=Mus musculus GN=Pfas PE=2 SV=1
Tmx4	Q8C0L0	1	1	39150	51825	81466	73353	94379	82473	96376	58684	69823	54896	0.735716	0.13329	68034.6	72450.4	Up	0.090725	0.918866 Thioredoxin-related transmembrane protein 4 OS=Mus musculus GN=Tmx4 PE=2 SV=2
Ndufs8	Q8K3J1	11	11	78317.11	121111.6	120673.8	113493.6	106436.6	157602.4	127073.2	98801.22	87357.44	95805.67	0.736244	0.132978	108006.5	113328	Up	0.069386	0.918866 NADH dehydrogenase [ubiquinone] iron-sulfur protein 8, mitochondrial OS=Mus musculus GN=Ndufs8 PE=1 SV=1
Rrbp1	Q99PL5-1:	1	1	59855	60801	73877	78832	116549	79491	60566	95446	78634	54719	0.748364	0.125887	77982.8	73771.2	Down	-0.0801	0.921905 Isoform RRp1.8 of Ribosome-binding protein 1 OS=Mus musculus GN=Rrbp1
Alg2	Q9DBE8	3	3	53447	65870	68662	76060	110499.5	113629	81477.5	71353	78068	53761.5	0.737579	0.132191	74907.7	79657.8	Up	0.088702	0.918866 Alpha-1,3/1,6-mannosyltransferase ALG2 OS=Mus musculus GN=Alg2 PE=2 SV=2
Psmf1	Q8BHL8	1	1	71480	112323	124494	75640	236289	100767	143635	87386	99404	135300	0.749209	0.125397	124045.2	113298.4	Down	-0.13074	0.921905 Proteasome inhibitor PI31 subunit OS=Mus musculus GN=Psmf1 PE=2 SV=1
Fus	P56959	4	4	70770.33	57882.33	80031.33	65770.67	92504.33	53812.33	79149.33	83690	91628.33	73825	0.737589	0.132186	73391.8	76421	Up	0.05835	0.918866 RNA-binding protein FUS OS=Mus musculus GN=Fus PE=1 SV=1
Rims3	Q80U57	1	1	NA	17367	36894	7804	17100	16119	14830	13770	26244	16854	0.750408	0.124703	19791.25	17563.4	Down	-0.17229	0.922887 Regulating synaptic membrane exocytosis protein 3 OS=Mus musculus GN=Rims3 PE=2 SV=2
Pin1	Q9QUR7	5	5	50461	74262	45434.25	70765.25	46922.75	61757.5	49762.5	51745.25	68199.5	69268.5	0.737718	0.13211	57569.05	60146.65	Up	0.063191	0.918866 Peptidyl-prolyl cis-trans isomerase NIMA-interacting 1 OS=Mus musculus GN=Pin1 PE=1 SV=1
G6pdx	Q00612	3	3	140136.3	190455	199074.3	187191	145223.3	138581.7	182670	180460	174220	162027.3	0.753141	0.123124	172416	167591.8	Down	-0.04094	0.92536 Glucose-6-phosphate 1-dehydrogenase X OS=Mus musculus GN=G6pdx PE=1 SV=3
Cnrip1	Q5M8N0	6	6	532910.3	359547.3	307239.2	321394.2	308925	374517.3	317258.7	371323.3	361553.3	331757.8	0.754186	0.122522	366003.2	351282.1	Down	-0.05923	0.9262 CB1 cannabinoid receptor-interacting protein 1 OS=Mus musculus GN=Cnrip1 PE=1 SV=1
Sphkap	Q6NSW3-:	1	1	NA	31401	22933	44982	65003	21190	17824	33041	61444	51553	0.75481	0.122162	41079.75	37010.4	Down	-0.1505	0.92637 Isoform 3 of A-kinase anchor protein SPHKAP OS=Mus musculus GN=Sphkap
Pgk1	P09411	34	34	570160.5	454202	376715.5	434638.6	426439.6	419836.8	434619.2	418348.8	521906.2	405188.8	0.755047	0.122026	452431.2	439980	Down	-0.04026	0.92637 Phosphoglycerate kinase 1 OS=Mus musculus GN=Pgk1 PE=1 SV=4
Cycs	P62897	12	12	1181880	1070299	1239528	1266938	1122487	1274242	1114640	1112012	1005614	1272234	0.755538	0.121744	1176226	1155748	Down	-0.02534	0.926528 Cytochrome c, somatic OS=Mus musculus GN=Cycs PE=1 SV=2
Acat1	Q8QZT1	23	23	1359098	1536784	1330323	1514836	1418083	1728619	1430260	1410985	1275525	1461714	0.737965	0.131964	1431825	1461420	Up	0.029516	0.918866 Acetyl-CoA acetyltransferase, mitochondrial OS=Mus musculus GN=Acat1 PE=1 SV=1
Ablim1	Q8K4G5-4	3	3	95106.67	78047	80913	75052.67	80986.67	99548.67	85192.33	84613	73190.33	77372.33	0.738664	0.131553	82021.2	83983.33	Up	0.034106	0.918866 Isoform 4 of Actin-binding LIM protein 1 OS=Mus musculus GN=Ablim1
Xkr4	Q5GH67	1	1	NA	110563	135598	88158	133391	55720	109097	158755	108033	120975	0.75732	0.120721	116927.5	110516	Down	-0.08136	0.927051 XK-related protein 4 OS=Mus musculus GN=Xkr4 PE=1 SV=1
Frmpd4	A2AFR3-3	1	1	NA	333229	495436	336264	343573	398460	330426	358984	401374	478116	0.73994	0.130803	377125.5	393472	Up	0.061216	0.918866 Isoform 3 of FERM and PDZ domain-containing protein 4 OS=Mus musculus GN=Frmpd4
Coro1c	Q9WUM4	7	7	79335.71	83770.29	94842.43	71210.43	119737.4	100971.7	91667.29	118469.4	79735.86	77424.57	0.740132	0.130691	89779.26	93653.77	Up	0.060955	0.918866 Coronin-1C OS=Mus musculus GN=Coro1c PE=1 SV=2
Trio	Q0KL02-4	3	3	18701.67	21123.67	14971.67	24439	20735.67	19017.33	25597.67	14327.33	24242.33	19809	0.740304	0.13059	19994.33	20784.07	Up	0.069322	0.918866 Isoform 4 of Triple functional domain protein OS=Mus musculus GN=Trio
Acadl	P51174	3	3	147202.7	307512	303046	469277.3	335562.3	337538.7	343444.3	320260.3	365603	288338	0.741879	0.129667	312520.1	331036.9	Up	0.083043	0.919027 Long-chain specific acyl-CoA dehydrogenase, mitochondrial OS=Mus musculus GN=Acadl PE=1 SV=2
Ensa	P60840-3	4	4	478256.3	374027	284208.5	369736.5	373987.5	392009.3	362084.5	342667.8	345099	385828.8	0.759235	0.119624	376043.2	365537.9	Down	-0.04088	0.927512 Isoform 3 of Alpha-endosulfine OS=Mus musculus GN=Ensa
Etl4	A2AQ25-7	1	1	319385	263299	195586	220483	179211	301197	220767	196196	193991	215052	0.759826	0.119286	235592.8	225440.6	Down	-0.06355	0.927614 Isoform 7 of Sickle tail protein OS=Mus musculus GN=Skt
Armc10	Q9D0L7	2	2	NA	6088.5	23503.5	17751.5	12043	7206.5	9113	22502.5	18639.5	9072.5	0.760043	0.119162	14846.63	13306.8	Down	-0.15797	0.927614 Armadillo repeat-containing protein 10 OS=Mus musculus GN=Armc10 PE=1 SV=1
Ndufa12	Q7TMF3	11	11	128008.3	104311.1	120962.4	129467.4	115972	144467.9	119076.6	103725.7	106906.6	110853.1	0.760825	0.118715	119744.2	117006	Down	-0.03337	0.928127 NADH dehydrogenase [ubiquinone] 1 alpha subcomplex subunit 12 OS=Mus musculus GN=Ndufa12 PE=1 SV=2
Unc5a	Q8K1S4	1	1	56004	78587	77647	60119	109497	102381	57783	68278	65290	68928	0.761308	0.11844	76370.8	72532	Down	-0.0744	0.928274 Netrin receptor UNC5A OS=Mus musculus GN=Unc5a PE=2 SV=1
Fmr1	P35922-1:	1	1	NA	9625	19969	20319	22039	14852	27224	17239	20093	16659	0.7426	0.129245	17988	19213.4	Up	0.095078	0.919027 Isoform ISO11 of Fragile X mental retardation protein 1 homolog OS=Mus musculus GN=Fmr1
Ckap5	A2AGT5-3	4	4	122900	301161	324146.5	341158.5	369665.8	341929.8	349028.5	316144.3	302304.5	232944.8	0.742618	0.129235	291806.4	308470.4	Up	0.080121	0.919027 Isoform 3 of Cytoskeleton-associated protein 5 OS=Mus musculus GN=Ckap5
Ccdc53	Q9CR27	1	1	NA	51875	99054	75257	106274	82550	153372	44573	83172	88500	0.743733	0.128583	83115	90433.4	Up	0.121747	0.919027 WASH complex subunit CCDC53 OS=Mus musculus GN=Ccdc53 PE=2 SV=1
Gpd2	Q64521	9	9	124161.5	170671	195609.1	230796.3	113150.8	137112.8	212104.4	131010.6	166122.9	146755.3	0.76346	0.117214	166877.7	158621.2	Down	-0.07321	0.929129 Glycerol-3-phosphate dehydrogenase, mitochondrial OS=Mus musculus GN=Gpd2 PE=1 SV=2
Actn1	Q7TPR4	18	18	197668.8	206512.6	247662.9	256696.6	253194.7	239991.1	260798	230300.1	238656.9	216617.8	0.743954	0.128454	232347.1	237272.8	Up	0.030265	0.919027 Alpha-actinin-1 OS=Mus musculus GN=Actn1 PE=1 SV=1
Chgb	P16014	1	1	26158	27905	39730	29495	36644	49755	24434	23648	31869	21058	0.764545	0.116597	31986.4	30152.8	Down	-0.08517	0.929129 Secretogranin-1 OS=Mus musculus GN=Chgb PE=1 SV=2
Cmas	Q99KK2	1	1	109561	145652	174969	221606	331522	57230	210490	241208	180212	216214	0.764547	0.116596	196662	181070.8	Down	-0.11916	0.929129 N-acylneuraminate cytidylyltransferase OS=Mus musculus GN=Cmas PE=1 SV=2
Ehd2	Q8BH64	1	1	8778	8718	11729	9147	9408	5609	11036	8170	9585	11502	0.765567	0.116017	9556	9180.4	Down	-0.05785	0.929928 EH domain-containing protein 2 OS=Mus musculus GN=Ehd2 PE=1 SV=1
Cpd	O89001	2	2	360920	223619	285088.5	154854.5	231236.5	279439	250697.5	236422	224437	207869	0.767755	0.114777	251143.7	239772.9	Down	-0.06684	0.932144 Carboxypeptidase D OS=Mus musculus GN=Cpd PE=1 SV=2
Ndufv1	Q91YT0	13	13	118924.9	170526.5	151799.9	214496.5	145394.2	183478.5	169889.2	162051.5	163134.7	151489.9	0.744252	0.12828	160228.4	166008.8	Up	0.05113	0.919027 NADH dehydrogenase [ubiquinone] flavoprotein 1, mitochondrial OS=Mus musculus GN=Ndufv1 PE=1 SV=1
Atp5d	Q9D3D9	5	5	NA	116559.8	74281.2	98244.4	24299.8	85286	89572.2	82525	79186	90998	0.744576	0.128091	78346.3	85513.44	Up	0.126286	0.919027 ATP synthase subunit delta, mitochondrial OS=Mus musculus GN=Atp5d PE=1 SV=1
Vps8	Q0P5W1	1	1	106339	128019	116707	101455	126356	144430	115540	134764	105491	96311	0.744753	0.127988	115775.2	119307.2	Up	0.043355	0.919027 Vacuolar protein sorting-associated protein 8 homolog OS=Mus musculus GN=Vps8 PE=2 SV=1
Pfn1	P62962	19	19	742182.4	442913.8	380924.9	400487.3	414547.1	436933.7	455871.4	439425.5	490297	454644.3	0.774401	0.111034	476211.1	455434.4	Down	-0.06436	0.938115 Profilin-1 OS=Mus musculus GN=Pfn1 PE=1 SV=2
Apoa1bp	Q8K4Z3	1	1	61527	55485	59075	64987	71255	80968	62317	60113	61878	55729	0.744866	0.127922	62465.8	64201	Up	0.039529	0.919027 NAD(P)H-hydrate epimerase OS=Mus musculus GN=Apoa1bp PE=1 SV=1
Rpl13a	P19253	3	3	1050608	763369	627896	672367	655640.7	747815.3	744432.7	716134.3	870760.3	833094.7	0.745118	0.127775	753976.1	782447.5	Up	0.053475	0.919027 60S ribosomal protein L13a OS=Mus musculus GN=Rpl13a PE=1 SV=4
Daam2	Q80U19	1	1	4972	9417	8956	9014	9800	9211	9303	11041	6188	8385	0.747321	0.126493	8431.8	8825.6	Up	0.065854	0.921301 Disheveled-associated activator of morphogenesis 2 OS=Mus musculus GN=Daam2 PE=2 SV=4
Vps52	Q8C754	1	1	NA	11091	NA	5269	NA		NA	9181	4788	NA	0.776231	0.110009	8180	6984.5	Down	-0.22794	0.938559 Vacuolar protein sorting-associated protein 52 homolog OS=Mus musculus GN=Vps52 PE=2 SV=1
Cic	Q924A2	1	1	NA	17411	25397	20338	36809	NA	33353	NA	22052	25091	0.748724	0.125678	24988.75	26832	Up	0.102676	0.921905 Protein capicua homolog OS=Mus musculus GN=Cic PE=1 SV=2
Ppm1j	Q149T7	1	1	248333	357286	342224	293532	442413	430448	285317	318400	331987	252575	0.776701	0.109746	336757.6	323745.4	Down	-0.05685	0.938559 Protein phosphatase 1J OS=Mus musculus GN=Ppm1j PE=1 SV=1
Hnrnpc	Q9Z204-3	3	3	115388.5	164687	176642.5	182136	66772.5	185461.5	208701	178185	114748.5	177776	0.777176	0.109481	159713.5	150773.7	Down	-0.0831	0.938691 Isoform 3 of Heterogeneous nuclear ribonucleoproteins C1/C2 OS=Mus musculus GN=Hnrnpc
Fam98b	Q80VD1	1	1	384164	229018	193691	201376	268143	259643	248748	262006	239129	213137	0.777783	0.109142	255278.4	244532.6	Down	-0.06204	0.938982 Protein FAM98B OS=Mus musculus GN=Fam98b PE=2 SV=1
Glul	P15105	19	19	149484.4	191532.5	174318.6	194740.8	195758.6	190279.6	169982.6	190328.3	177066.8	194884.8	0.74925	0.125373	181167	184508.4	Up	0.026367	0.921905 Glutamine synthetase OS=Mus musculus GN=Glul PE=1 SV=6
Slc25a4	P48962	9	9	196434.8	506428.1	533720.1	684664.9	539626.9	425692.9	502787.8	503807	471983.3	434212.4	0.778797	0.108576	492175	467696.7	Down	-0.0736	0.939213 ADP/ATP translocase 1 OS=Mus musculus GN=Slc25a4 PE=1 SV=4
Rpl36a	P83882	3	3	302408.3	216254.3	182569.3	180758.3	185974.7	267230.7	194628.7	190647.3	200973	173475.7	0.779359	0.108262	213593	205391.1	Down	-0.05649	0.939213 60S ribosomal protein L36a OS=Mus musculus GN=Rpl36a PE=1 SV=2
Ptpa	P58389	1	1	85973	125033	114538	123994	112884	101574	113264	119501	131512	110685	0.75308	0.123159	112484.4	115307.2	Up	0.035758	0.92536 Serine/threonine-protein phosphatase 2A activator OS=Mus musculus GN=Ppp2r4 PE=1 SV=1
Rps7	P62082	3	3	308283.7	208864.7	190496.7	216411.7	146061	239174.3	181709.7	234043	199533.7	175844.3	0.779807	0.108013	214623.5	206061	Down	-0.05874	0.939213 40S ribosomal protein S7 OS=Mus musculus GN=Rps7 PE=2 SV=1
Eif4b	Q8BGD9	7	7	234522	244000	197865	180520.3	180912.3	245252.7	189182.3	212890.3	192703.7	225093.3	0.756277	0.121319	207563.8	213024.7	Up	0.037465	0.926822 Eukaryotic translation initiation factor 4B OS=Mus musculus GN=Eif4b PE=1 SV=1
Pzp	Q61838	1	1	261227	293621	306261	276417	349122	289872	270805	309040	302457	290676	0.782178	0.106694	297329.6	292570	Down	-0.02328	0.941184 Alpha-2-macroglobulin OS=Mus musculus GN=A2m PE=1 SV=3
Pip5k1c	O70161-2	8	8	135821.6	123746.9	149240	137055	177993.3	180015.9	128158.6	171593	135178.7	131892.7	0.756501	0.12119	144771.3	149367.8	Up	0.045093	0.926822 Isoform 2 of Phosphatidylinositol 4-phosphate 5-kinase type-1 gamma OS=Mus musculus GN=Pip5k1c
Igbp1	Q61249	3	3	96696.33	64337.67	74343.33	82185	58115.67	65934.33	87950.33	66608.33	83300	59520.67	0.78439	0.105468	75135.6	72662.73	Down	-0.04828	0.942961 Immunoglobulin-binding protein 1 OS=Mus musculus GN=Igbp1 PE=1 SV=1
2410015M	Q8R404	2	2	333339.5	425111	351503	566277	296316.5	369322	400676.5	350321	416988.5	526462	0.757714	0.120495	394509.4	412754	Up	0.065223	0.927051 Protein QIL1 OS=Mus musculus GN=Qil1 PE=2 SV=1
Gls	D3Z7P3	9	9	118693	97461.83	130287.8	195713	141313.2	123029.3	154949.2	104284.5	141152.5	133693.7	0.785593	0.104802	136693.8	131421.8	Down	-0.05674	0.943522 Glutaminase kidney isoform, mitochondrial OS=Mus musculus GN=Gls PE=1 SV=1
Anks1b	Q8BIZ1-5	2	2	228995	189998	186619.5	165698.5	219272.5	204160.5	221442	199594	172258	216045	0.758258	0.120183	198116.7	202699.9	Up	0.032995	0.927051 Isoform 5 of Ankyrin repeat and sterile alpha motif domain-containing protein 1B OS=Mus musculus GN=Anks1b
Ppp1r1b	Q60829	3	3	901288	834896	779793	487517	959482	1114280	816631	890994	631911	696401	0.75827	0.120176	792595.2	830043.4	Up	0.066603	0.927051 Protein phosphatase 1 regulatory subunit 1B OS=Mus musculus GN=Ppp1r1b PE=2 SV=2
Pdap1	Q3UHX2	1	1	NA	40329	24903	20695	32739	22343	40307	30823	27538	35962	0.758496	0.120047	29666.5	31394.6	Up	0.081682	0.927051 28 kDa heat- and acid-stable phosphoprotein OS=Mus musculus GN=Pdap1 PE=1 SV=1
Nudt3	Q9JI46	1	1	838353	732805	714259	657758	689941	869635	720176	776000	728860	618422	0.762672	0.117662	726623.2	742618.6	Up	0.031414	0.929129 Diphosphoinositol polyphosphate phosphohydrolase 1 OS=Mus musculus GN=Nudt3 PE=1 SV=1
Numb	Q9QZS3-3	2	2	178500.5	144930	144259	173298.5	210728	224114.5	159769	150851	154113.5	135481	0.788124	0.103405	170343.2	164865.8	Down	-0.04715	0.944349 Isoform 3 of Protein numb homolog OS=Mus musculus GN=Numb
Snph	Q80U23-2	4	4	NA	831	25808.33	25768.67	16652	33824.33	13142	30645.67	9697.33	11332.67	0.762828	0.117573	17265	19728.4	Up	0.192424	0.929129 Isoform 2 of Syntaphilin OS=Mus musculus GN=Snph
Nedd4l	Q8CFI0-3	2	2	22273	39279	31976	30994.5	34827.5	32356.5	28841	36162	40041.5	27604.5	0.763329	0.117288	31870	33001.1	Up	0.050315	0.929129 Isoform 3 of E3 ubiquitin-protein ligase NEDD4-like OS=Mus musculus GN=Nedd4l
Omg	Q63912	1	1	NA	226642	130899	171945	210637	181078	209539	165489	176212	159988	0.789949	0.102401	185030.8	178461.2	Down	-0.05215	0.94521 Oligodendrocyte-myelin glycoprotein OS=Mus musculus GN=Omg PE=1 SV=1
Igsf8	Q8R366	17	17	75307.08	160484.2	157590.4	141734.3	139557.8	119110.2	133185.6	140540.2	156969.9	151440	0.76442	0.116668	134934.7	140249.2	Up	0.05573	0.929129 Immunoglobulin superfamily member 8 OS=Mus musculus GN=Igsf8 PE=1 SV=2
B3gat3	P58158	1	1	82195	43154	184623	99881	147148	63713	124900	126668	111314	92428	0.791566	0.101513	111400.2	103804.6	Down	-0.10188	0.945857 Galactosylgalactosylxylosylprotein 3-beta-glucuronosyltransferase 3 OS=Mus musculus GN=B3gat3 PE=2 SV=1
Tial1	P70318	1	1	NA	13632	12822	28234	21466	20796	NA	17215	15730	18097	0.791597	0.101496	19038.5	17959.5	Down	-0.08417	0.945857 Nucleolysin TIAR OS=Mus musculus GN=Tial1 PE=1 SV=1
Ubb	P0CG49	3	3	NA	293882	101218.3	196289.7	124420.3	93791	106385.3	121558	302199	381757	0.770904	0.113	178952.6	201138.1	Up	0.168609	0.935524 Polyubiquitin-B OS=Mus musculus GN=Ubb PE=2 SV=1
Erlin1	Q91X78	2	2	NA	118396.5	78611	92981	78203	95566	62331.5	82687	117339	124463.5	0.772756	0.111958	92047.88	96477.4	Up	0.067807	0.937133 Erlin-1 OS=Mus musculus GN=Erlin1 PE=1 SV=1
Mvb12b	Q6KAU4	1	1	NA	10121	21619	15678	18553	9072	19065	8845	19149	21364	0.792995	0.10073	16492.75	15499	Down	-0.08966	0.946204 Multivesicular body subunit 12B OS=Mus musculus GN=Mvb12b PE=2 SV=2
Pde4a	O89084	3	3	183523.3	173815	161078.7	214117.7	176662	124413	150753.7	239395.3	183895.7	181673	0.793616	0.10039	181839.3	176026.1	Down	-0.04687	0.946415 cAMP-specific 3',5'-cyclic phosphodiesterase 4A OS=Mus musculus GN=Pde4a PE=2 SV=2
Rps29	P62274	1	1	419092	344870	333231	201350	361142	147217	375388	360500	331397	369602	0.79435	0.099988	331937	316820.8	Down	-0.06724	0.946415 40S ribosomal protein S29 OS=Mus musculus GN=Rps29 PE=2 SV=2
Mrps31	Q61733	1	1	NA	29407	9354	33405	9679	18257	12073	9470	23977	28519	0.795221	0.099512	20461.25	18459.2	Down	-0.14855	0.946415 28S ribosomal protein S31, mitochondrial OS=Mus musculus GN=Mrps31 PE=2 SV=1
Txndc15	Q6P6J9	1	1	NA	NA	54025	32346	NA	93565	31057	43104	47573	25776	0.772961	0.111842	43185.5	48215	Up	0.158935	0.937133 Thioredoxin domain-containing protein 15 OS=Mus musculus GN=Txndc15 PE=1 SV=1
Pde10a	Q8CA95-3	1	1	NA	48493	127050	81806	92005	118604	86824	90899	57213	112420	0.774837	0.11079	87338.5	93192	Up	0.093588	0.938115 Isoform 3 of cAMP and cAMP-inhibited cGMP 3',5'-cyclic phosphodiesterase 10A OS=Mus musculus GN=Pde10a
Hist1h2ba	P70696	6	6	384118.7	1018513	1445717	1372188	2686039	178369	1342453	1754899	1593193	1409068	0.795731	0.099234	1381315	1255596	Down	-0.13767	0.946415 Histone H2B type 1-A OS=Mus musculus GN=Hist1h2ba PE=1 SV=3
Tjp1	P39447	6	6	468469.8	399460.8	351375.6	389752	369394.8	442735.2	361432.6	402464.4	366789.6	371316.2	0.795757	0.09922	395690.6	388947.6	Down	-0.0248	0.946415 Tight junction protein ZO-1 OS=Mus musculus GN=Tjp1 PE=1 SV=2
Ndufa8	Q9DCJ5	5	5	91582.5	206388.3	223425.8	306084.8	177292	239154	222389	221461.5	200000.5	176790.8	0.774869	0.110772	200954.7	211959.2	Up	0.076916	0.938115 NADH dehydrogenase [ubiquinone] 1 alpha subcomplex subunit 8 OS=Mus musculus GN=Ndufa8 PE=1 SV=3
Dvl1	P51141	1	1	NA	14009	6261	10988	29187	20191	14518	31012	10055	9262	0.775688	0.110313	15111.25	17007.6	Up	0.170557	0.938559 Segment polarity protein dishevelled homolog DVL-1 OS=Mus musculus GN=Dvl1 PE=1 SV=2
Specc1	Q5SXY1-3	1	1	NA	21481	34509	15822	19100	17465	10057	23553	12182	41153	0.797549	0.098243	22728	20882	Down	-0.12221	0.946632 Isoform 4 of Cytospin-B OS=Mus musculus GN=Specc1
Slitrk6	Q8C110	1	1	1130825	816570	661688	665178	771699	1007172	871829	952168	741527	634604	0.776563	0.109823	809192	841460	Up	0.056413	0.938559 SLIT and NTRK-like protein 6 OS=Mus musculus GN=Slitrk6 PE=1 SV=1
Idh3g	P70404	8	8	82300.5	76942.67	105738.3	118768	110774.2	61318.33	112264.8	83719.17	130187.3	130217	0.778663	0.10865	98904.73	103541.3	Up	0.066095	0.939213 Isocitrate dehydrogenase [NAD] subunit gamma 1, mitochondrial OS=Mus musculus GN=Idh3g PE=1 SV=1
Tpd52	Q62393-3	6	6	462205	432509.3	397197.7	377447.2	451369.5	480638.3	446106.3	461393.2	382733.2	386862.3	0.779775	0.108031	424145.7	431546.7	Up	0.024957	0.939213 Isoform 3 of Tumor protein D52 OS=Mus musculus GN=Tpd52
Ndufs6	P52503	3	3	286219	709360	565849	631793	613978	633056	607181	575137	465101	415306	0.798962	0.097474	561439.8	539156.2	Down	-0.05843	0.947151 NADH dehydrogenase [ubiquinone] iron-sulfur protein 6, mitochondrial OS=Mus musculus GN=Ndufs6 PE=1 SV=2
Wdr47	Q8CGF6	3	3	279944.3	496159.7	458002	517645	540223	536497.3	461474.7	432770.3	390629.3	400121.3	0.800573	0.096599	458394.8	444298.6	Down	-0.04506	0.948622 WD repeat-containing protein 47 OS=Mus musculus GN=Wdr47 PE=1 SV=2
Exoc1	Q8R3S6	2	2	104699.5	163810.5	195909.5	160676.5	159306.5	145711.5	186701	130410	183510	164349.5	0.781215	0.107229	156880.5	162136.4	Up	0.047542	0.940466 Exocyst complex component 1 OS=Mus musculus GN=Exoc1 PE=1 SV=4
Clip1	Q922J3	3	3	629725.3	511629	327417	437723	458527.3	512679.3	483505.3	492281.3	382576.7	421878.3	0.802211	0.095711	473004.3	458584.2	Down	-0.04467	0.949454 CAP-Gly domain-containing linker protein 1 OS=Mus musculus GN=Clip1 PE=1 SV=1
Epn2	Q8CHU3	2	2	21023	73421.5	51896	57283	69558.5	46584.5	70986	55165.5	56189.5	58746	0.784328	0.105502	54636.4	57534.3	Up	0.07456	0.942961 Epsin-2 OS=Mus musculus GN=Epn2 PE=1 SV=1
Trf	Q921I1	11	11	463634.1	332770.8	284188.6	241581.8	250828.9	315430.1	263424.7	378657.4	322400.2	357562.8	0.784848	0.105214	314600.8	327495	Up	0.057951	0.943069 Serotransferrin OS=Mus musculus GN=Tf PE=1 SV=1
Hars2	Q99KK9	1	1	135622	233941	312100	363732	183840	297663	329874	219725	229315	219665	0.786487	0.104308	245847	259248.4	Up	0.076574	0.944153 Probable histidine--tRNA ligase, mitochondrial OS=Mus musculus GN=Hars2 PE=2 SV=1
Cd99l2	Q8BIF0	1	1	NA	85023	56951	48044	74927	77330	66120	57733	60788	83018	0.787264	0.10388	66236.25	68997.8	Up	0.058929	0.944349 CD99 antigen-like protein 2 OS=Mus musculus GN=Cd99l2 PE=1 SV=1
Pde1b	Q01065	2	2	115969.5	106627	69112.5	69983	42553	87341	56286.5	78703.5	80358	126111	0.78755	0.103722	80849	85760	Up	0.085075	0.944349 Calcium/calmodulin-dependent 3',5'-cyclic nucleotide phosphodiesterase 1B OS=Mus musculus GN=Pde1b PE=1 SV=2
Set	Q9EQU5	1	1	NA	NA	NA	3838	3888	NA	4242	4528	2791	3466	0.80437	0.094544	3863	3756.75	Down	-0.04024	0.949454 Protein SET OS=Mus musculus GN=Set PE=1 SV=1
Ndufb11	O09111	1	1	240371	380733	371359	321757	352840	369678	365685	310279	337025	323705	0.788015	0.103466	333412	341274.4	Up	0.033626	0.944349 NADH dehydrogenase [ubiquinone] 1 beta subcomplex subunit 11, mitochondrial OS=Mus musculus GN=Ndufb11 PE=1 SV=2
Cask	O70589-2	2	2	326487	156897	284512	211627	204973	264087	277228	231627	267066	192111	0.789199	0.102813	236899.2	246423.8	Up	0.056868	0.945195 Isoform 2 of Peripheral plasma membrane protein CASK OS=Mus musculus GN=Cask
Ccdc93	Q7TQK5	1	1	NA	17114	18245	15219	15107	8261	18009	15616	21256	22721	0.789692	0.102542	16421.25	17172.6	Up	0.064545	0.94521 Coiled-coil domain-containing protein 93 OS=Mus musculus GN=Ccdc93 PE=2 SV=1
Phf24	Q80TL4-2	3	3	498309	438452	473932.5	358503.5	428823	405010.5	480666	406512.5	491103.5	455728.5	0.791282	0.101669	439604	447804.2	Up	0.026664	0.945857 Isoform 2 of Protein KIAA1045 OS=Mus musculus GN=Kiaa1045
Bpnt1	Q9Z0S1	3	3	112735.3	104131.7	154062	126902.3	140985.7	138697.7	129188.7	130419.3	132443	121286	0.792721	0.10088	127763.4	130406.9	Up	0.029546	0.946204 3'(2'),5'-bisphosphate nucleotidase 1 OS=Mus musculus GN=Bpnt1 PE=1 SV=2
Ociad2	Q9D8W7	3	3	329864	264391	284319	330822.3	278088.3	369435.7	308989.3	269384.3	287818	215428	0.807759	0.092718	297496.9	290211.1	Down	-0.03577	0.950291 OCIA domain-containing protein 2 OS=Mus musculus GN=Ociad2 PE=1 SV=1
Psma5	Q9Z2U1	1	1	292933	975991	712966	868606	850483	689195	845574	773575	893729	674506	0.792872	0.100797	740195.8	775315.8	Up	0.066877	0.946204 Proteasome subunit alpha type-5 OS=Mus musculus GN=Psma5 PE=1 SV=1
Rac1	P63001	3	3	176281.7	331506.3	534295.7	454418	366257.3	330443.3	466052.7	422783.3	431844	303983.7	0.795223	0.099511	372551.8	391021.4	Up	0.069807	0.946415 Ras-related C3 botulinum toxin substrate 1 OS=Mus musculus GN=Rac1 PE=1 SV=1
Mri1	Q9CQT1	2	2	NA	1018	2051	710	3103	1568	1099	2436	1907	804	0.808542	0.092297	1720.5	1562.8	Down	-0.13869	0.950291 Methylthioribose-1-phosphate isomerase OS=Mus musculus GN=Mri1 PE=2 SV=1
Btbd17	Q9DB72	3	3	82410.33	191710.7	140624.3	148413	168054.7	127663	138286.3	147105	156367	190402.7	0.795225	0.09951	146242.6	151964.8	Up	0.055374	0.946415 BTB/POZ domain-containing protein 17 OS=Mus musculus GN=Btbd17 PE=2 SV=1
Ndufaf2	Q59J78	2	2	NA	560	398.5	1625.5	2557	NA	1847.5	2096.5	377	1503.5	0.796234	0.098959	1285.25	1456.125	Up	0.180085	0.946544 Mimitin, mitochondrial OS=Mus musculus GN=Ndufaf2 PE=2 SV=1
Kras	P32883	10	10	307609.6	310994.6	227003.4	257971	221835.2	232162	277170	231140.4	262477.6	294147.2	0.810776	0.091099	265082.8	259419.4	Down	-0.03116	0.950291 GTPase KRas OS=Mus musculus GN=Kras PE=1 SV=1
Ndufs7	Q9DC70	3	3	158616	501933	413270.5	448852.5	360249.5	398256	406092.5	442477	299725.5	422903	0.797406	0.098321	376584.3	393890.8	Up	0.064823	0.946632 NADH dehydrogenase [ubiquinone] iron-sulfur protein 7, mitochondrial OS=Mus musculus GN=Ndufs7 PE=1 SV=1
Ttc9b	Q9D6E4	3	3	181126.3	104449.3	97194.33	95110.33	118368.7	135369	105580.3	114022	114881.3	105066.7	0.811041	0.090957	119249.8	114983.9	Down	-0.05256	0.950291 Tetratricopeptide repeat protein 9B OS=Mus musculus GN=Ttc9b PE=2 SV=1
Lrrc59	Q922Q8	7	7	147133.1	171002.4	184876.1	152792.3	193039.7	183920.4	166933.7	201738.4	151972.4	160897.9	0.797583	0.098224	169768.7	173092.6	Up	0.027973	0.946632 Leucine-rich repeat-containing protein 59 OS=Mus musculus GN=Lrrc59 PE=1 SV=1
Srr	Q9QZX7-3	3	3	27205.33	57629.67	52469	50392	57425	36338.33	51843.33	44970.67	57014.33	64847.33	0.797785	0.098114	49024.2	51002.8	Up	0.057082	0.946632 Isoform 3 of Serine racemase OS=Mus musculus GN=Srr
Irgq	Q8VIM9	3	3	234485.3	246394	228783	223077.7	220065	231577.7	259408.7	243553.7	243744.3	191263	0.798156	0.097912	230561	233909.5	Up	0.020802	0.946633 Immunity-related GTPase family Q protein OS=Mus musculus GN=Irgq PE=2 SV=1
Nmral1	Q8K2T1	1	1	65810	92394	87415	148597	75562	64155	97853	92211	121445	117175	0.801553	0.096068	93955.6	98567.8	Up	0.069137	0.949344 NmrA-like family domain-containing protein 1 OS=Mus musculus GN=Nmral1 PE=1 SV=1
Dhrs11	Q3U0B3	1	1	NA	26425	53359	18733	37816	21770	30179	25491	48470	57840	0.802943	0.095315	34083.25	36750	Up	0.108681	0.949454 Dehydrogenase/reductase SDR family member 11 OS=Mus musculus GN=Dhrs11 PE=2 SV=1
Gyg	Q9R062	1	1	445027	532161	462011	407362	365076	514637	370414	434144	379390	465412	0.812372	0.090245	442327.4	432799.4	Down	-0.03142	0.950301 Glycogenin-1 OS=Mus musculus GN=Gyg1 PE=2 SV=3
Lrrc8a	Q80WG5	2	2	120987	183367	226535	233766	263330	252341	266901	173376	209576.5	166789.5	0.804035	0.094725	205597	213796.8	Up	0.056421	0.949454 Leucine-rich repeat-containing protein 8A OS=Mus musculus GN=Lrrc8a PE=2 SV=1
Kif21a	Q9QXL2-3	13	13	109065.2	80608.83	103819.7	97617.5	104533.2	123053.4	98005.5	111488.4	103016.7	72688.08	0.804064	0.094709	99128.87	101650.4	Up	0.036239	0.949454 Isoform 3 of Kinesin-like protein KIF21A OS=Mus musculus GN=Kif21a
Nucb2	P81117	1	1	71305	46511	45148	45312	51636	80774	53656	54114	54122	29520	0.804189	0.094642	51982.4	54437.2	Up	0.06657	0.949454 Nucleobindin-2 OS=Mus musculus GN=Nucb2 PE=1 SV=2
Rcan1	Q9JHG6	1	1	583231	284543	296879	215450	323710	360096	327478	357038	289572	289251	0.815773	0.088431	340762.6	324687	Down	-0.06972	0.95254 Calcipressin-1 OS=Mus musculus GN=Rcan1 PE=1 SV=1
Ranbp2	Q9ERU9	1	1	44750	134250	159428	139847	249221	76490	181949	176950	183173	158379	0.80435	0.094555	145499.2	155388.2	Up	0.094866	0.949454 E3 SUMO-protein ligase RanBP2 OS=Mus musculus GN=Ranbp2 PE=1 SV=2
Pddc1	Q8BFQ8	2	2	55545.5	33271.5	44436.5	35552.5	50672.5	48073	37869	45446	41798	40693	0.817885	0.087308	43895.7	42775.8	Down	-0.03728	0.95278 Parkinson disease 7 domain-containing protein 1 OS=Mus musculus GN=Pddc1 PE=1 SV=1
Hist2h2aa	Q6GSS7	4	4	435854.3	773757	872341	946934.7	1628366	258674.3	947855	1128052	923808.7	1100628	0.818542	0.086959	931450.6	871803.5	Down	-0.09548	0.95278 Histone H2A type 2-A OS=Mus musculus GN=Hist2h2aa1 PE=1 SV=3
Hist3h2a	Q8BFU2	4	4	435854.3	773757	872341	946934.7	1628366	258674.3	947855	1128052	923808.7	1100628	0.818542	0.086959	931450.6	871803.5	Down	-0.09548	0.95278 Histone H2A type 3 OS=Mus musculus GN=Hist3h2a PE=1 SV=3
Cenpv	Q9CXS4	1	1	140909	193540	314812	263049	614192	170537	299945	305533	333492	311377	0.819155	0.086634	305300.4	284176.8	Down	-0.10344	0.95278 Centromere protein V OS=Mus musculus GN=Cenpv PE=2 SV=2
Vti1b	O88384	5	5	NA	55382	30581.25	65700.5	29014	29120.75	16316.25	47352	83432.25	69377.75	0.804894	0.094261	45169.44	49119.8	Up	0.120958	0.949454 Vesicle transport through interaction with t-SNAREs homolog 1B OS=Mus musculus GN=Vti1b PE=1 SV=1
Ak2	Q9WTP6	3	3	254082	168185	165151	234484.7	222726.7	260862.7	195482	183115.3	232013	202313.3	0.805074	0.094164	208925.9	214757.3	Up	0.039716	0.949454 Adenylate kinase 2, mitochondrial OS=Mus musculus GN=Ak2 PE=1 SV=5
Aldh3b1	Q80VQ0	1	1	NA	90201	175336	190921	127521	114159	184864	140187	89187	167401	0.820246	0.086056	145994.8	139159.6	Down	-0.06918	0.95278 Aldehyde dehydrogenase family 3 member B1 OS=Mus musculus GN=Aldh3b1 PE=2 SV=1
Cherp	Q8CGZ0	1	1	92038	148971	109716	70935	185492	62329	138628	139467	111974	125393	0.820623	0.085856	121430.4	115558.2	Down	-0.07151	0.95278 Calcium homeostasis endoplasmic reticulum protein OS=Mus musculus GN=Cherp PE=1 SV=1
Nup214	Q80U93	1	1	NA	49724	32674	39757	56859	32786	40718	46499	47251	66377	0.805351	0.094015	44753.5	46726.2	Up	0.062231	0.949454 Nuclear pore complex protein Nup214 OS=Mus musculus GN=Nup214 PE=1 SV=2
Vasp	P70460	1	1	240775	152013	420272	263896	396714	389521	295362	341476	265971	253316	0.806397	0.093451	294734	309129.2	Up	0.068796	0.950251 Vasodilator-stimulated phosphoprotein OS=Mus musculus GN=Vasp PE=1 SV=4
Trim28	Q62318	1	1	167243	228522	249100	238182	339229	219693	298253	226409	273790	244035	0.807286	0.092973	244455.2	252436	Up	0.046348	0.950291 Transcription intermediary factor 1-beta OS=Mus musculus GN=Trim28 PE=1 SV=3
Aldh1a7	O35945	2	2	13326	23164	20965	26627.5	49089	6467.5	30488	27974	33173.5	26106.5	0.821354	0.08547	26634.3	24841.9	Down	-0.10051	0.95278 Aldehyde dehydrogenase, cytosolic 1 OS=Mus musculus GN=Aldh1a7 PE=2 SV=1
Hist3h2ba	Q9D2U9	12	12	233491.7	646461.7	930352.6	895249.9	1698596	127919.3	870156	1118803	1033300	906211.4	0.821449	0.085419	880830.3	811277.9	Down	-0.11867	0.95278 Histone H2B type 3-A OS=Mus musculus GN=Hist3h2ba PE=1 SV=3
Cyth2	P63034-3	1	1	NA	NA	11625	23175	7633	27116	6008	11401	9143	9942	0.821804	0.085232	14144.33	12722	Down	-0.1529	0.95278 Isoform 3 of Cytohesin-2 OS=Mus musculus GN=Cyth2

Slc8a1	P70414	5	5	18092	30217	31310.2	30718.4	30900.6	35334.8	30003.2	35598.4	24928.8	20291.8	0.808021	0.092577	28247.64	29231.4	Up	0.049389	0.950291	Sodium/calcium exchanger 1 OS=Mus musculus GN=Slc8a1 PE=1 SV=1
Sntb2	Q61235	1	1	301120	229294	235588	292366	189780	262236	220424	229457	207936	297417	0.822298	0.084971	249629.6	243494	Down	-0.0359	0.95278	Beta-2-syntrophin OS=Mus musculus GN=Sntb2 PE=1 SV=2
Hist1h1c	P15864	11	11	158931.4	331591.8	478288.8	518665.6	801765	172103.6	433682	503033.8	542830.8	492352.6	0.824182	0.083977	457848.5	428800.5	Down	-0.09456	0.954532	Histone H1.2 OS=Mus musculus GN=Hist1h1c PE=1 SV=2
Gna14	P30677	3	3	48311.33	56991	105017	85796	97076.33	96575.67	96207.33	101337.3	73687.67	44676	0.808122	0.092523	78638.33	82496.8	Up	0.069105	0.950291	Guanine nucleotide-binding protein subunit alpha-14 OS=Mus musculus GN=Gna14 PE=1 SV=2
Otud4	B2RRE7	1	1	NA	82988	65101	51087	81061	111607	53132	76890	65276	59362	0.810033	0.091497	70059.25	73253.4	Up	0.06432	0.950291	OTU domain-containing protein 4 OS=Mus musculus GN=Otud4 PE=1 SV=1
Mecp2	Q9Z2D6	3	3	NA	70745.5	101759.5	120030	144935	31444.5	101749	169312	119393.5	93829.5	0.825599	0.083231	109367.5	103145.7	Down	-0.0845	0.954878	Methyl-CpG-binding protein 2 OS=Mus musculus GN=Mecp2 PE=1 SV=1
Ddt	O35215	6	6	229663	323586.6	289260.6	326589.4	208507.8	302333.4	289565.4	230667.4	274928.6	315376.2	0.810517	0.091238	275521.5	282574.2	Up	0.036465	0.950291	D-dopachrome decarboxylase OS=Mus musculus GN=Ddt PE=1 SV=3
Tanc2	A2A690	1	1	221726	354156	227490	244196	244803	319102	247166	236014	242463	283680	0.810801	0.091086	258474.2	265685	Up	0.039696	0.950291	Protein TANC2 OS=Mus musculus GN=Tanc2 PE=1 SV=1
Rpl9	P51410	2	2	237999.5	269093.5	171596	186927.5	251121	238986	214288	260012	179369.5	253668	0.811111	0.09092	223347.5	229264.7	Up	0.037724	0.950291	60S ribosomal protein L9 OS=Mus musculus GN=Rpl9 PE=1 SV=2
Pcp4l1	Q6W8Q3	1	1	174230	170488	101052	165892	210700	238461	137923	180986	154322	142322	0.811144	0.090902	164472.4	170802.8	Up	0.054486	0.950291	Purkinje cell protein 4-like protein 1 OS=Mus musculus GN=Pcp4l1 PE=1 SV=1
Rps4x	P62702	8	8	893581.4	525647.8	663997.6	502128.6	604566.6	739113.1	653946.4	671222.8	544156.8	487856.8	0.829056	0.081416	637984.4	619259.2	Down	-0.04298	0.95565	40S ribosomal protein S4, X isoform OS=Mus musculus GN=Rps4x PE=2 SV=2
Cox5a	P12787	17	17	276360	321382.5	273224.9	288947.7	275832.3	312489.6	247922.2	339397.5	292789	216029.6	0.829316	0.08128	287149.5	281725.6	Down	-0.02751	0.95565	Cytochrome c oxidase subunit 5A, mitochondrial OS=Mus musculus GN=Cox5a PE=1 SV=2
Hist2h2be	Q64524	11	11	251000.3	717186	1029644	992563.8	1876726	140781.1	964180.3	1241020	1146281	1006596	0.829371	0.081251	973424.1	899771.6	Down	-0.11351	0.95565	Histone H2B type 2-E OS=Mus musculus GN=Hist2h2be PE=1 SV=3
Slc27a4	Q91VE0	1	1	88632	43709	71385	81949	92348	31215	90360	80145	102233	93010	0.811284	0.090827	75604.6	79392.6	Up	0.070531	0.950291	Long-chain fatty acid transport protein 4 OS=Mus musculus GN=Slc27a4 PE=1 SV=1
Tnr	Q8BYI9	8	8	344595.4	367784.4	433498.8	339728.6	338061.6	366140.4	380333.4	335171.4	357778.2	362298.8	0.829816	0.081018	364733.8	360344.4	Down	-0.01747	0.95565	Tenascin-R OS=Mus musculus GN=Tnr PE=1 SV=2
Lin7a	Q8JZS0	5	5	1192341	853370.8	877046.8	886907.4	941147.2	1160555	968914.2	892869.6	829850.8	799213.2	0.829995	0.080925	950162.6	930280.6	Down	-0.03051	0.95565	Protein lin-7 homolog A OS=Mus musculus GN=Lin7a PE=1 SV=2
Cpne4	Q8BLR2	3	3	162997.7	164350.3	174691.3	153753	168139.7	184686.3	151458.7	166886	176438.3	153510.7	0.811765	0.09057	164786.4	166596	Up	0.015757	0.950291	Copine-4 OS=Mus musculus GN=Cpne4 PE=1 SV=1
Rps3a1	P97351	4	4	1026415	642506.7	681037.7	746461.3	844851.7	1148505	856505	766788.3	687314.3	623401.7	0.811992	0.090448	788254.5	816502.9	Up	0.050797	0.950291	40S ribosomal protein S3a OS=Mus musculus GN=Rps3a PE=1 SV=3
Hace1	O3U0D9-3	2	2	27704.5	21166	50470	34066	49290	30092.5	15911.5	41530.5	62063	44880.5	0.813685	0.089544	36539.3	38895.6	Up	0.090158	0.951403	Isoform 3 of E3 ubiquitin-protein ligase HACE1 OS=Mus musculus GN=Hace1
Qk	Q9QYS9-5	3	3	314562	272422	366512	359456.5	320851.5	388218	309917.5	376062.5	248453	276085	0.834043	0.078812	326760.8	319747.2	Down	-0.0313	0.957038	Isoform 5 of Protein quaking OS=Mus musculus GN=Qki
Sgip1	Q8VD37-3	19	19	215137.2	213161.7	203496.1	203056.7	211001.5	206571.7	214676.1	213916.4	207682.7	206742.8	0.814813	0.088942	209170.6	209918	Up	0.005145	0.952177	Isoform 3 of SH3-containing GRB2-like protein 3-interacting protein 1 OS=Mus musculus GN=Sgip1
Paics	Q9DCL9	2	2	27062	39655	48933	37386	28954.5	21582.5	40568.5	46689.5	31952.5	34973.5	0.834893	0.078369	36398.1	35153.3	Down	-0.0502	0.957038	Multifunctional protein ADE2 OS=Mus musculus GN=Paics PE=1 SV=4
Gars	Q9CZD3	1	1	66350.5	52443.5	55386	90715	32987.5	71136	60034.5	45687	70981.5	62949	0.81509	0.088794	59576.5	62157.6	Up	0.061187	0.952177	Glycine--tRNA ligase OS=Mus musculus GN=Gars PE=1 SV=1
Gad2	P48320	7	7	82475.17	154654.2	193168.3	187408.5	168793.2	183064.5	192002.5	141389	161311	136156	0.817328	0.087604	157299.9	162784.6	Up	0.049447	0.95278	Glutamate decarboxylase 2 OS=Mus musculus GN=Gad2 PE=2 SV=1
Stoml2	Q99JB2	7	7	34039	35129.83	27251.17	38566.67	36987.17	40715.33	29709.83	32963.5	31446.5	34231	0.835416	0.078097	34394.77	33813.23	Down	-0.0246	0.957038	Stomatin-like protein 2, mitochondrial OS=Mus musculus GN=Stoml2 PE=1 SV=1
Aifm3	Q3TY86-3	1	1	34075	30272	55287	39176	47879	44791	42431	53253	33254	39618	0.819269	0.086573	41337.8	42669.4	Up	0.04574	0.95278	Isoform 3 of Apoptosis-inducing factor 3 OS=Mus musculus GN=Aifm3
Anks1	P59672	1	1	130964	132506	84515	117602	147460	177277	88981	105112	88736	131621	0.835652	0.077975	122609.4	118345.4	Down	-0.05107	0.957038	Ankyrin repeat and SAM domain-containing protein 1A OS=Mus musculus GN=Anks1a PE=1 SV=3
Me1	P06801	1	1	163041	135402	135268	181569	97817	130654	137681	126463	141830	159743	0.835681	0.077959	142619.4	139274.2	Down	-0.03424	0.957038	NADP-dependent malic enzyme OS=Mus musculus GN=Me1 PE=1 SV=2
Acot13	Q9CQR4	11	11	437780.9	468233.9	458162.8	499173.4	527553.3	662402.1	456336.8	430931.1	479779.6	418072	0.820086	0.086141	478180.8	489504.3	Up	0.033765	0.95278	Acyl-coenzyme A thioesterase 13 OS=Mus musculus GN=Acot13 PE=1 SV=1
Ntpcr	Q9CQA9	2	2	NA	32555	30066	33714.5	12407.5	31036	33163	34170	21794.5	22533.5	0.820737	0.085796	27185.75	28539.4	Up	0.070104	0.95278	Cancer-related nucleoside-triphosphatase homolog OS=Mus musculus GN=Ntpcr PE=2 SV=1
Sgip1	Q8VD37-8	22	22	199258.9	201952.5	188239.8	190257	195637.2	196297.6	200158.2	196843.2	192346.9	193185.6	0.8209	0.08571	195069	195766.3	Up	0.005147	0.95278	Isoform 7 of SH3-containing GRB2-like protein 3-interacting protein 1 OS=Mus musculus GN=Sgip1
Plcl2	Q8K394	1	1	73466	58574	114481	83067	141194	201146	48093	141567	94194	27154	0.821048	0.085631	94156.4	102430.8	Up	0.121519	0.95278	Inactive phospholipase C-like protein 2 OS=Mus musculus GN=Plcl2 PE=1 SV=2
Pum1	Q80U78-3	4	4	10081.67	19654.33	21661	25667	28289.33	27587.67	23246	17594.33	24455.67	16849.33	0.822024	0.085116	21070.67	21946.6	Up	0.058762	0.95278	Isoform 3 of Pumilio homolog 1 OS=Mus musculus GN=Pum1
Tbc1d24	Q3UUG6	1	1	NA	136665	137506	149384	164624	171635	89711	178459	156185	120272	0.840999	0.075205	147044.8	143252.4	Down	-0.0377	0.960553	TBC1 domain family member 24 OS=Mus musculus GN=Tbc1d24 PE=2 SV=2
Psmd9	Q9CR00	3	3	65121	44938	49671.5	56425.5	59998	86034	38653	46047	54504.5	41104	0.841493	0.074949	55230.8	53268.5	Down	-0.05219	0.960631	26S proteasome non-ATPase regulatory subunit 9 OS=Mus musculus GN=Psmd9 PE=1 SV=1
Rgs20	Q9QZB1	1	1	217679	258629	196754	377085	340542	271648	343034	218744	353747	253380	0.825131	0.083477	278137.8	288110.2	Up	0.050821	0.954878	Regulator of G-protein signaling 20 OS=Mus musculus GN=Rgs20 PE=1 SV=1
Fam185a	Q7TPD2	1	1	128525	115104	176935	99192	145696	226248	125863	97312	110930	74541	0.842395	0.074484	133090.4	126978.8	Down	-0.06782	0.960631	Protein FAM185A OS=Mus musculus GN=Fam185a PE=2 SV=1
Gsk3b	Q9WV60	3	3	NA	92416.5	97437	72570	82714	67080	74716.5	58646	101641.5	116627.5	0.842567	0.074396	86284.38	83742.3	Down	-0.04314	0.960631	Glycogen synthase kinase-3 beta OS=Mus musculus GN=Gsk3b PE=1 SV=2
Lin7b	O88951	1	1	467743	350740	419219	330068	510761	347773	416378	428191	433467	413909	0.843285	0.074026	415706.2	407943.6	Down	-0.02719	0.960842	Protein lin-7 homolog B OS=Mus musculus GN=Lin7b PE=1 SV=2
Eif5a2	Q8BGY2	3	3	1705160	2002498	1459087	1781009	1445740	1543373	1761055	1479627	1605263	1874001	0.843501	0.073914	1678699	1652664	Down	-0.02255	0.960842	Eukaryotic translation initiation factor 5A-2 OS=Mus musculus GN=Eif5a2 PE=2 SV=3
Crocc	Q8CJ40-3	2	2	94237	133772	140942.5	152978	165581	187007.5	125020	106106	123285	168313	0.825335	0.08337	137502.1	141946.3	Up	0.045892	0.954878	Isoform 3 of Rootletin OS=Mus musculus GN=Crocc
Kctd16	Q5DTY9	2	2	214467.5	165988.5	155492.5	144644.5	235872.5	238179.5	181879	147884.5	186621.5	187837.5	0.826545	0.082733	183293.1	188480.4	Up	0.040262	0.955541	BTB/POZ domain-containing protein KCTD16 OS=Mus musculus GN=Kctd16 PE=1 SV=2
Sccpdh	Q8R127	2	2	132339	518510	659758	688196	457292	456722	610225	534744	610325	369247	0.827638	0.08216	491219	516252.6	Up	0.071711	0.95565	Saccharopine dehydrogenase-like oxidoreductase OS=Mus musculus GN=Sccpdh PE=2 SV=1
Hecw2	Q6I6G8	1	1	NA	78497	53834	70638	42775	54654	53154	57147	60189	94700	0.827865	0.08204	61436	63968.8	Up	0.058284	0.95565	E3 ubiquitin-protein ligase HECW2 OS=Mus musculus GN=Hecw2 PE=2 SV=1
Arl15	Q8BGR6	1	1	NA	101458	116044	99558	101274	111684	119615	112096	86597	100702	0.828697	0.081604	104583.5	106138.8	Up	0.021297	0.95565	ADP-ribosylation factor-like protein 15 OS=Mus musculus GN=Arl15 PE=2 SV=1
Nudcd3	Q8R1N4-2	2	2	19277	34717.5	21428.5	19711	21879.5	22857	24037.5	21082.5	21648.5	24377.5	0.846997	0.072118	23402.7	22800.6	Down	-0.0376	0.962006	Isoform 4 of NudC domain-containing protein 3 OS=Mus musculus GN=Nudcd3
Atxn2l	Q7TQH0-3	1	1	NA	38663	67527	50753	77194	78581	61161	79346	44607	14062	0.847287	0.071969	58534.25	55551.4	Down	-0.07546	0.962006	Isoform 3 of Ataxin-2-like protein OS=Mus musculus GN=Atxn2l
Cadps2	Q8BYR5-7	1	1	NA	49043	90074	68906	109523	119684	82567	75112	54913	46385	0.847526	0.071847	79386.5	75732.2	Down	-0.06799	0.962006	Isoform 7 of Calcium-dependent secretion activator 2 OS=Mus musculus GN=Cadps2
Epha4	Q03137	2	2	NA	160161	121920.5	133508.5	118808.5	72451	142315.5	144545.5	166119.5	164477	0.829737	0.08106	133599.6	137981.7	Up	0.046561	0.95565	Ephrin type-A receptor 4 OS=Mus musculus GN=Epha4 PE=1 SV=2
Gpr158	Q8C419	10	10	36294.83	62959	54772	61269	48418.83	56515.33	45900	81497.33	40127.17	49265	0.831638	0.080066	52742.73	54660.97	Up	0.051539	0.957038	Probable G-protein coupled receptor 158 OS=Mus musculus GN=Gpr158 PE=1 SV=2
Spr	Q64105	5	5	79766	83626.33	66879.67	76873.33	71412.67	80632.67	65701.67	90204.33	66836.33	69708	0.849896	0.070634	75711.6	74616.6	Down	-0.02102	0.962828	Sepiapterin reductase OS=Mus musculus GN=Spr PE=1 SV=1
Gns	Q8BFR4	3	3	75857	47519	55352.67	47539.67	67021.67	57321.67	62831.33	65183.33	58255.33	56221	0.832717	0.079503	58658	59962.53	Up	0.031733	0.957038	N-acetylglucosamine-6-sulfatase OS=Mus musculus GN=Gns PE=2 SV=1
Kif1b	Q60575-2	1	1	NA	37780	23030	22510	9207	16558	20887	28330	23748	33416	0.833301	0.079198	23131.75	24587.8	Up	0.088068	0.957038	Isoform 2 of Kinesin-like protein KIF1B OS=Mus musculus GN=Kif1b
Ppp3ca	P63328-2	10	10	171449.4	203143.1	231597.5	220554.6	249202.8	257345.3	219552.3	232652.1	193766.6	192184.2	0.834056	0.078805	215189.5	219100.1	Up	0.025983	0.957038	Isoform 2 of Serine/threonine-protein phosphatase 2B catalytic subunit alpha isoform OS=Mus musculus GN=Ppp3ca
Dnm1l	Q8K1M6	13	13	281517.4	291695.8	269230.6	268743.3	262223	283325.6	234739.6	304490.5	274629.8	290278.9	0.835072	0.078276	274682	277492.9	Up	0.014688	0.957038	Dynamin-1-like protein OS=Mus musculus GN=Dnm1l PE=1 SV=2
Dnm1l	Q8K1M6-3	11	11	281517.4	291695.8	269230.6	268743.3	262223	283325.6	234739.6	304490.5	274629.8	290278.9	0.835072	0.078276	274682	277492.9	Up	0.014688	0.957038	Isoform 3 of Dynamin-1-like protein OS=Mus musculus GN=Dnm1l
Eno1	P17182	122	122	157691.7	179173.4	162104.6	180588.7	144670.3	165038	179272.7	171928.7	174053.6	143790.4	0.835438	0.078086	164845.7	166816.7	Up	0.017147	0.957038	Alpha-enolase OS=Mus musculus GN=Eno1 PE=1 SV=3
Ube2l3	P68037	7	7	333971.4	291392.1	362311.4	376434.9	385894.4	409478.9	385604.4	357964	296975.1	269885	0.853642	0.068724	350009.9	343981.5	Down	-0.02503	0.964676	Ubiquitin-conjugating enzyme E2 L3 OS=Mus musculus GN=Ube2l3 PE=2 SV=1
Actg1	P63260	23	23	2677403	2384220	2536523	2192032	2316490	2474322	2278009	2517038	2481997	2259455	0.855075	0.067996	2421334	2402164	Down	-0.01147	0.965435	Actin, cytoplasmic 2 OS=Mus musculus GN=Actg1 PE=1 SV=1
Mrps21	P58059	1	1	10234	10724	8862	8977	9444	10779	9459	10114	7797	9513	0.855348	0.067857	9648.2	9532.4	Down	-0.01742	0.965435	28S ribosomal protein S21, mitochondrial OS=Mus musculus GN=Mrps21 PE=2 SV=1
Rhob	P62746	4	4	362555.8	427888	365442.3	465257.5	395978.8	352783	440216.8	348941.8	415862	433227.8	0.855444	0.067808	403424.5	398206.3	Down	-0.01878	0.965435	Rho-related GTP-binding protein RhoB OS=Mus musculus GN=Rhob PE=1 SV=1
Kras	P32883-2	12	12	222007.7	224522.9	164419.6	186470.7	160534.6	168054	200569.7	171818.7	189991.1	212511.4	0.857296	0.066869	191591.1	188589	Down	-0.02278	0.966814	Isoform 2B of GTPase KRas OS=Mus musculus GN=Kras
Rprd1b	Q9CSU0-3	1	1	40667	32229	33473	18868	44721	NA	40940	9172	40291	38839	0.857812	0.066608	33991.6	32310.5	Down	-0.07318	0.966814	Isoform 3 of Regulation of nuclear pre-mRNA domain-containing protein 1B OS=Mus musculus GN=Rprd1b
Atp2a1	Q8R429	2	2	NA	154875	120489	158259	156346	112814	188615	162630	154924	135130	0.837883	0.076817	147492.3	150822.6	Up	0.032213	0.959131	Sarcoplasmic/endoplasmic reticulum calcium ATPase 1 OS=Mus musculus GN=Atp2a1 PE=2 SV=1
Ank2	Q8C8R3-8	10	10	191443	347508.1	288788.7	241517.1	297097.6	306271.4	265211.7	298227.1	271015.1	255593.9	0.840148	0.075644	273270.9	279263.8	Up	0.031297	0.960553	Isoform 8 of Ankyrin-2 OS=Mus musculus GN=Ank2
Timm13	P62075	1	1	NA	15262	25046	23160	10635	NA	23055	10332	24725	12294	0.859107	0.065953	18525.75	17601.5	Down	-0.07383	0.967154	Mitochondrial import inner membrane translocase subunit Tim13 OS=Mus musculus GN=Timm13 PE=1 SV=1
Adssl1	P28650	2	2	NA	23571	13327.5	12939.5	9578.5	9219	13203	19522.5	16231.5	19927	0.840585	0.075418	14854.13	15620.6	Up	0.072586	0.960553	Adenylosuccinate synthetase isozyme 1 OS=Mus musculus GN=Adssl1 PE=1 SV=2
Nipsnap1	O55125	7	7	286314.8	330840	333831.7	413063	371312.5	355404.5	368898.3	302063.3	342980.5	343854.8	0.859841	0.065582	347072.4	342640.3	Down	-0.01854	0.967415	Protein NipSnap homolog 1 OS=Mus musculus GN=Nipsnap1 PE=1 SV=1
G6pd2	P97324	1	1	NA	156656	114357	156740	81128	32209	133747	128434	143329	171123	0.86032	0.06534	127220.3	121768.4	Down	-0.06319	0.967478	Glucose-6-phosphate 1-dehydrogenase 2 OS=Mus musculus GN=G6pd2 PE=1 SV=3
Capn5	O08688	2	2	448934.5	227230.5	315508	275665	378643	399356.5	298304	329827	313708	263778	0.860669	0.065164	329196.2	320994.7	Down	-0.0364	0.967478	Calpain-5 OS=Mus musculus GN=Capn5 PE=2 SV=1
Txnl1	Q8CDN6	1	1	NA	74610	59359	50059	23593	30909	57485	60068	77723	47195	0.840918	0.075246	51905.25	54476	Up	0.075027	0.960553	Thioredoxin-like protein 1 OS=Mus musculus GN=Txnl1 PE=2 SV=3
Slmo1	Q8VE85	1	1	68616	100956	98753	101470	47645	67525	89043	120768	65826	89560	0.840948	0.075231	83488	86544.4	Up	0.051872	0.960553	Protein slowmo homolog 1 OS=Mus musculus GN=Slmo1 PE=2 SV=1
Arhgef2	Q60875-3	1	1	NA	50836	38457	48669	17436	19289	44266	47891	39074	53743	0.842289	0.074539	38849.5	40852.6	Up	0.072532	0.960631	Isoform 3 of Rho guanine nucleotide exchange factor 2 OS=Mus musculus GN=Arhgef2
Grsf1	Q8C5Q4	3	3	67651.33	71539.33	81621.67	85086.67	65550.33	79667	83574.67	67119.67	75537	60459	0.862958	0.06401	74289.87	73271.47	Down	-0.01991	0.967478	G-rich sequence factor 1 OS=Mus musculus GN=Grsf1 PE=1 SV=2
Lmnb2	P21619	5	5	70789.2	100628.4	174824.4	134801.6	251321	54516.2	160390.6	156466.2	174424.8	152530.6	0.863352	0.063812	146472.9	139665.7	Down	-0.06866	0.967478	Lamin-B2 OS=Mus musculus GN=Lmnb2 PE=1 SV=2
H2afv	Q3THW5	5	5	326860.4	393192	639345.2	673779	891561.2	301603.8	598895.8	637573.8	647206.2	630975.8	0.863419	0.063778	584947.6	563251.1	Down	-0.05453	0.967478	Histone H2A.V OS=Mus musculus GN=H2afv PE=1 SV=3
Isca1	Q9D924	1	1	NA	183630	123595	176125	112807	168967	134495	142884	128135	152962	0.863703	0.063636	149039.3	145488.6	Down	-0.03479	0.967478	Iron-sulfur cluster assembly 1 homolog, mitochondrial OS=Mus musculus GN=Isca1 PE=2 SV=1
Acat2	Q8CAY6	10	10	44952.88	40229.38	46876.25	45305.5	46627.88	53304.75	46493	43394.62	38832.38	44673.62	0.844811	0.07324	44798.38	45339.67	Up	0.017328	0.961655	Acetyl-CoA acetyltransferase, cytosolic OS=Mus musculus GN=Acat2 PE=1 SV=2
Cops4	O88544	2	2	616018	609288	465378	508604	378827	595829	508456	494074	547174	482926	0.845416	0.07293	515623	525691.8	Up	0.027901	0.961655	COP9 signalosome complex subunit 4 OS=Mus musculus GN=Cops4 PE=1 SV=1
Necab2	Q91ZP9	3	3	180940	188846	192853	166250	227206	221751	190898.3	172603	177257.3	207312.3	0.845682	0.072793	191219	193964.4	Up	0.020566	0.961655	N-terminal EF-hand calcium-binding protein 2 OS=Mus musculus GN=Necab2 PE=1 SV=1
Slc25a5	P51881	6	6	137729.5	223633.8	252639	282572	219887.5	210271.3	256835.3	233273.7	212692	179089.7	0.865106	0.062931	223292.4	218432.4	Down	-0.03175	0.967816	ADP/ATP translocase 2 OS=Mus musculus GN=Slc25a5 PE=1 SV=3
Ik	Q9Z1M8	1	1	59859	97515	110461	88219	128306	27077	112232	78137	113671	134027	0.866449	0.062257	96872	93028.8	Down	-0.0584	0.968896	Protein Red OS=Mus musculus GN=Ik PE=1 SV=2
Aldh7a1	Q9DBF1	4	4	332487.3	414210.5	480002.3	578631.3	522528.8	488253.8	496418.8	503027.5	446382.8	439757.5	0.845716	0.072775	465572	474768.1	Up	0.028219	0.961655	Alpha-aminoadipic semialdehyde dehydrogenase OS=Mus musculus GN=Aldh7a1 PE=1 SV=4
Pacs1	Q8K212	8	8	103115.4	186007.6	192537.8	166500.5	185842.4	196481.8	154330.3	169019.9	173863.3	158637.1	0.846602	0.072321	166800.7	170466.5	Up	0.031362	0.962006	Phosphofurin acidic cluster sorting protein 1 OS=Mus musculus GN=Pacs1 PE=2 SV=2
Coq9	Q8K1Z0	1	1	156809	359877	422492	543799	291266	417453	376622	245646	326293	483877	0.848542	0.071327	354848.6	369978.2	Up	0.060237	0.962733	Ubiquinone biosynthesis protein COQ9, mitochondrial OS=Mus musculus GN=Coq9 PE=1 SV=1
Chchd2	Q9D1L0	2	2	6567	6755	16856	14351	16544	13054	15282.5	10476.5	13789.5	10934.5	0.84981	0.070678	12214.6	12707.4	Up	0.057062	0.962828	Coiled-coil-helix-coiled-coil-helix domain-containing protein 2, mitochondrial OS=Mus musculus GN=Chchd2 PE=2 SV=1
Negr1	Q80Z24	13	13	235806.4	389908.8	329587.3	309321.7	401303	364099.8	310842.9	346035	330712.4	345375.5	0.850266	0.070445	333185.4	339413.1	Up	0.026717	0.962828	Neuronal growth regulator 1 OS=Mus musculus GN=Negr1 PE=1 SV=1
Nisch	Q80TM9-4	2	2	93029	80201	73252.5	62292	75406.5	54141	101243	81701	75271.5	63790.5	0.870302	0.06033	76836.2	75229.4	Down	-0.03049	0.970329	Isoform 4 of Nischarin OS=Mus musculus GN=Nisch
Dnaja2	Q9QYJ0	3	3	187579.3	161027.7	150852.7	139453.7	118035.7	178952.7	150065.3	141190.3	139844	135135.7	0.870846	0.060059	151389.8	149037.6	Down	-0.02259	0.970329	DnaJ homolog subfamily A member 2 OS=Mus musculus GN=Dnaja2 PE=1 SV=1
Ethe1	Q9DCM0	3	3	112198.3	138090.7	148978.7	164375	150842.7	111473.7	162413.3	136941.3	165709.3	150792.7	0.850522	0.070314	142897.1	145466.1	Up	0.025706	0.962828	Persulfide dioxygenase ETHE1, mitochondrial OS=Mus musculus GN=Ethe1 PE=1 SV=2
Lamtor1	Q9CQ22	5	5	141029.4	83498	79919.6	67641	97212.8	91666.6	81178.4	89809	113016.6	107407	0.850865	0.070139	93860.16	96615.52	Up	0.041742	0.962828	Ragulator complex protein LAMTOR1 OS=Mus musculus GN=Lamtor1 PE=1 SV=1
Baiap2	Q8BKX1-2	28	28	394432.8	481509.8	378007.5	376292.7	449666.9	450900.4	426430.1	414152.7	396034.1	415023.1	0.85088	0.070132	415981.9	420508.1	Up	0.015613	0.962828	Isoform 2 of Brain-specific angiogenesis inhibitor 1-associated protein 2 OS=Mus musculus GN=Baiap2
Gnaq	P21279	4	4	51244	50274	107497.3	86000.5	90035.75	94819.5	91142	90963.75	75681	46319	0.852736	0.069185	77010.3	79785.05	Up	0.051067	0.964503	Guanine nucleotide-binding protein G(q) subunit alpha OS=Mus musculus GN=Gnaq PE=1 SV=4
Rps17	P63276	11	11	1564650	1275701	979923.1	940687.7	1095098	1463088	1026190	1146814	1141338	1208545	0.8535	0.068796	1171212	1197195	Up	0.031656	0.964676	40S ribosomal protein S17 OS=Mus musculus GN=Rps17 PE=1 SV=2
Pip5k1c	O70161	7	7	145660	129173.7	157822.7	145298.2	191046.2	188610.5	136705.8	183505.2	139844.2	134994.8	0.858166	0.066429	153800.1	156732.1	Up	0.027244	0.966814	Phosphatidylinositol 4-phosphate 5-kinase type-1 gamma OS=Mus musculus GN=Pip5k1c PE=1 SV=2
Ptprr	Q62132-3	1	1	NA	3700	7919	1877	9652	NA	10767	3774	4065	2833	0.873173	0.0589	5787	5359.75	Down	-0.11065	0.970487	Isoform Gamma of Receptor-type tyrosine-protein phosphatase R OS=Mus musculus GN=Ptprr
Mfn2	Q80U63	2	2	82626	93375.5	110636	76833.5	108106.5	112789	82829.5	96771	80248.5	91655	0.87368	0.058648	94315.5	92858.6	Down	-0.02246	0.970629	Mitofusin-2 OS=Mus musculus GN=Mfn2 PE=1 SV=3
Pdha1	P35486	18	18	146429	207742	164316.3	228958.2	193971.3	185518.9	198203.4	180061.2	201258.4	162830.5	0.874149	0.058415	188283.4	185574.5	Down	-0.02091	0.970702	Pyruvate dehydrogenase E1 component subunit alpha, somatic form, mitochondrial OS=Mus musculus GN=Pdha1 PE=1 SV=1
Wash1	Q8VDD8	3	3	33800.5	91275.5	121087	146209.5	138581	113664	112750	91451	116682	78418.5	0.875065	0.05796	106190.7	102593.1	Down	-0.04972	0.970702	WAS protein family homolog 1 OS=Mus musculus GN=Wash1 PE=2 SV=1
Fip1l1	Q9D824-4	1	1	NA	62455	155749	51658	263232	139125	134805	81285	126011	142312	0.875092	0.057946	133273.5	124707.6	Down	-0.09584	0.970702	Isoform 4 of Pre-mRNA 3'-end-processing factor FIP1 OS=Mus musculus GN=Fip1l1
Idh3a	Q9D6R2	12	12	232020.3	344088.7	314365.1	418392.6	269253	337500.3	310310.3	305572.8	321564	334125.3	0.858174	0.066425	315623.9	321814.5	Up	0.028023	0.966814	Isocitrate dehydrogenase [NAD] subunit alpha, mitochondrial OS=Mus musculus GN=Idh3a PE=1 SV=1
Ndufs1	Q91VD9	10	10	96480.14	102522.4	93508	100279.1	75440.71	77497.14	92539	87167.71	97563.86	107842.6	0.876223	0.057385	93646.08	92522.06	Down	-0.01742	0.970839	NADH-ubiquinone oxidoreductase 75 kDa subunit, mitochondrial OS=Mus musculus GN=Ndufs1 PE=1 SV=2
Cdh2	P15116	8	8	87940.71	131898.1	127542.7	133382.4	163712.7	109395.1	141752.6	134765.9	135285.4	112108	0.876375	0.05731	128895.3	126661.4	Down	-0.02522	0.970839	Cadherin-2 OS=Mus musculus GN=Cdh2 PE=1 SV=2
Mtap	Q9CQ65	2	2	56981.5	30241	44848.5	25187	49098.5	33985	30040	33281.5	66003	35946.5	0.876843	0.057078	41271.3	39851.2	Down	-0.05052	0.970839	S-methyl-5'-thioadenosine phosphorylase OS=Mus musculus GN=Mtap PE=1 SV=1
Cog6	Q8R3I3	1	1	117389	52411	165769	111953	189316	228679	66776	161549	85328	NA	0.859232	0.06589	127367.6	135583	Up	0.090178	0.967154	Conserved oligomeric Golgi complex subunit 6 OS=Mus musculus GN=Cog6 PE=2 SV=2
Shank1	D3YZU1	35	35	72838.41	77025.09	78076.06	72627.53	87541.88	77835.38	79680.62	84835.44	73276.24	69489.21	0.87826	0.056377	77621.79	77023.38	Down	-0.01117	0.970839	SH3 and multiple ankyrin repeat domains protein 1 OS=Mus musculus GN=Shank1 PE=1 SV=1
Ptprs	B0V2N1-6	3	3	NA	48910.67	58707.67	57784	40654.33	29651	55951.33	65613.33	66293.67	47352.67	0.861044	0.064975	51514.17	52972.4	Up	0.040272	0.967478	Isoform 6 of Receptor-type tyrosine-protein phosphatase S OS=Mus musculus GN=Ptprs
Usp9x	P70398	1	1	NA	703	2125	1627	642	1752	1149	830	2345	724	0.86183	0.064578	1274.25	1360	Up	0.093958	0.967478	Probable ubiquitin carboxyl-terminal hydrolase FAF-X OS=Mus musculus GN=Usp9x PE=1 SV=2
Thop1	Q8C1A5	1	1	508043	670402	589811	727002	596818	675702	571810	606081	731807	459876	0.879338	0.055844	618415.2	609055.2	Down	-0.022	0.970839	Thimet oligopeptidase OS=Mus musculus GN=Thop1 PE=1 SV=1
Ufm1	P61961	2	2	59647	104710	119875.5	117019	146181	105870	121492.5	142745	112167.5	80736.5	0.86292	0.064029	109486.5	112602.3	Up	0.040483	0.967478	Ubiquitin-fold modifier 1 OS=Mus musculus GN=Ufm1 PE=1 SV=1
Pdlim5	Q8CI51	2	2	65020.5	73690.5	82608	56344	108481.5	115613.5	81201.5	73546.5	56180.5	71418.5	0.863756	0.063609	77228.9	79592.1	Up	0.043484	0.967478	PDZ and LIM domain protein 5 OS=Mus musculus GN=Pdlim5 PE=1 SV=4
Rem2	Q8VEL9	1	1	190591	145552	153885	119184	123095	201555	137512	130476	122963	124540	0.879927	0.055553	146461.4	143409.2	Down	-0.03038	0.970839	GTP-binding protein REM 2 OS=Mus musculus GN=Rem2 PE=1 SV=2
Dlgap1	Q9D415-4	5	5	463572.4	395272.2	361506.2	412425.4	439935	468875	411738	375392.4	455313.4	337843.8	0.880055	0.05549	414542.2	409832.5	Down	-0.01648	0.970839	Isoform 4 of Disks large-associated protein 1 OS=Mus musculus GN=Dlgap1
Flot2	Q60634-3	7	7	179296.1	327611	289127	316497	328698.7	327988.4	288025.3	271269.6	257870.6	271920.3	0.880467	0.055287	288246	283414.8	Down	-0.02439	0.970839	Isoform 3 of Flotillin-2 OS=Mus musculus GN=Flot2
Acad8	Q9D7B6	1	1	251413	194651	288859	276128	264231	244271	310790	155289	225765	312904	0.880687	0.055178	255056.4	249803.8	Down	-0.03002	0.970839	Isobutyryl-CoA dehydrogenase, mitochondrial OS=Mus musculus GN=Acad8 PE=1 SV=2
Hibadh	Q99L13	10	10	117555.3	161486.4	129847.9	151159.4	131073.9	142588.9	142318.9	134377.3	141952	137239.2	0.864049	0.063462	138224.6	139695.3	Up	0.015269	0.967478	3-hydroxyisobutyrate dehydrogenase, mitochondrial OS=Mus musculus GN=Hibadh PE=1 SV=1
Gapvd1	Q6PAR5-4	1	1	74902	21862	43819	40944	15430	NA	31986	62363	61287	13164	0.864817	0.063076	39391.4	42200	Up	0.099362	0.967816	Isoform 4 of GTPase-activating protein and VPS9 domain-containing protein 1 OS=Mus musculus GN=Gapvd1
Ywhae	P62259	9	9	1214686	1078563	1016026	1014362	999205.4	1039429	1185082	1026903	1156303	964848.8	0.867185	0.061888	1064568	1074513	Up	0.013414	0.969296	14-3-3 protein epsilon OS=Mus musculus GN=Ywhae PE=1 SV=1
Matr3	Q8K310	15	15	65026.08	92753.08	116726.9	105231.8	171030.4	86598	102940.6	130077.8	130599.5	117278.9	0.869011	0.060975	110153.6	113499	Up	0.043162	0.970329	Matrin-3 OS=Mus musculus GN=Matr3 PE=1 SV=1
Acsl6	Q91WC3	2	2	142179	220663.5	203304	229102.5	175334	258234	183173	218224	152102	179505	0.869086	0.060937	194116.6	198247.6	Up	0.03038	0.970329	Long-chain-fatty-acid--CoA ligase 6 OS=Mus musculus GN=Acsl6 PE=2 SV=1
Pdhb	Q9D051	13	13	210908.4	237987.4	194923.6	302532.2	208655.6	237324.7	222649	193967.3	211484.1	272018.1	0.884336	0.053383	231001.4	227488.6	Down	-0.02211	0.971812	Pyruvate dehydrogenase E1 component subunit beta, mitochondrial OS=Mus musculus GN=Pdhb PE=1 SV=1
Crk	Q64010	3	3	NA	76495	69148.5	70731	74714.5	97161	72665.5	69728.5	66105	63645.5	0.869295	0.060833	72772.25	73861.1	Up	0.021426	0.970329	Adapter molecule crk OS=Mus musculus GN=Crk PE=1 SV=1
Pdxk	Q8K183	1	1	249630	112910	151381	110188	148535	183282	125381	163760	142193	137236	0.884603	0.053252	154528.8	150370.4	Down	-0.03936	0.971812	Pyridoxal kinase OS=Mus musculus GN=Pdxk PE=1 SV=1
Dlgap2	Q8BJ42	4	4	566779	475316.3	429849.5	464011.5	474038.5	552183.8	465736.5	448007.8	523293	393953	0.88556	0.052782	481999	476634.8	Down	-0.01615	0.972447	Disks large-associated protein 2 OS=Mus musculus GN=Dlgap2 PE=1 SV=2
Ralyl	Q8BTF8	2	2	24914.5	21461	40158	40979.5	76313.5	53079	54522	39908	33536.5	32021.5	0.870159	0.060401	40765.3	42613.4	Up	0.063966	0.970329	RNA-binding Raly-like protein OS=Mus musculus GN=Ralyl PE=2 SV=1
Cadm4	Q8R464	3	3	64509	161781	157019	186598	130945	185822	115713	132870	135845	150814	0.871405	0.05978	140170.4	144212.8	Up	0.041017	0.970329	Cell adhesion molecule 4 OS=Mus musculus GN=Cadm4 PE=1 SV=1
Baiap2	Q8BKX1	27	27	406090.3	499566	391723.8	388367.5	462753.9	465007.1	441509.2	428403.1	407689	426213.7	0.87145	0.059758	429700.3	433764.4	Up	0.013581	0.970329	Brain-specific angiogenesis inhibitor 1-associated protein 2 OS=Mus musculus GN=Baiap2 PE=1 SV=2
Xrn1	P97789-2	2	2	174479	237911	243226	194233	337733	286127	267844	254764	195033	156605	0.887455	0.051854	237516.4	232074.6	Down	-0.03344	0.972747	Isoform 2 of 5'-3' exoribonuclease 1 OS=Mus musculus GN=Xrn1
Dmtn	Q9WV69-3	7	7	83032	90812	113651.2	79117.6	94638.8	124889	88763.8	88628.8	81745.2	69133	0.88785	0.05166	92250.32	90631.96	Down	-0.02553	0.972747	Isoform 3 of Dematin OS=Mus musculus GN=Dmtn
Pclo	Q9QYX7	42	42	70540.79	102666.4	80139.87	90378.95	98340.97	92251.63	84576.53	82384.26	75163	102262.3	0.888452	0.051366	88413.39	87327.54	Down	-0.01783	0.972747	Protein piccolo OS=Mus musculus GN=Pclo PE=1 SV=4
Nsfl1c	Q9CZ44-3	4	4	49258.75	85549.25	71296.75	63451	63570.25	53089	90316.75	51833.5	72072	73590.25	0.871517	0.059724	66625.2	68180.3	Up	0.033287	0.970329	Isoform 3 of NSFL1 cofactor p47 OS=Mus musculus GN=Nsfl1c
Aco2	Q99KI0	34	34	386150.6	430574	301331.8	426746.6	298923	369171.6	343098.8	338573.1	347335.2	421734.8	0.889544	0.050833	368745.2	363982.7	Down	-0.01875	0.972747	Aconitate hydratase, mitochondrial OS=Mus musculus GN=Aco2 PE=1 SV=1
Numbl	O08919	1	1	174976	39814	43420	39484	147133	210656	52186	67959	46315	36175	0.889601	0.050805	88965.4	82658.2	Down	-0.10609	0.972747	Numb-like protein OS=Mus musculus GN=Numbl PE=1 SV=3
Map1a	Q9QYR6	34	34	235534.8	168751.1	183972.8	169108	219763	193313.1	194358.7	224209.1	189504.7	163735	0.889629	0.050791	195425.9	193024.1	Down	-0.01784	0.972747	Microtubule-associated protein 1A OS=Mus musculus GN=Map1a PE=1 SV=2
Syncrip	Q7TMK9	11	11	562309.3	450082.2	434342.3	426198.7	483278.4	480879.9	545595.6	491423.4	453903.6	411916.9	0.872345	0.059312	471242.2	476743.9	Up	0.016746	0.970475	Heterogeneous nuclear ribonucleoprotein Q OS=Mus musculus GN=Syncrip PE=1 SV=2
Hmgb2	P30681	4	4	121245.3	201966.5	231063	222892.3	270826.8	170857.8	287112.5	202828.5	236755.5	177588.3	0.872426	0.059271	209598.8	215028.5	Up	0.036898	0.970475	High mobility group protein B2 OS=Mus musculus GN=Hmgb2 PE=1 SV=3
Rgcc	Q9DBX1	1	1	NA	8667	12356	11327	15127	NA	14254	7332	11688	13058	0.891825	0.04972	11869.25	11583	Down	-0.03522	0.973648	Regulator of cell cycle RGCC OS=Mus musculus GN=Rgcc PE=1 SV=1
Qk	Q9QYS9-6	5	5	157281	168289.8	236504.3	207244.8	216923.5	244480.8	180303.8	235837.3	146926.3	161384.3	0.892361	0.049459	197248.7	193786.5	Down	-0.02555	0.973648	Isoform 6 of Protein quaking OS=Mus musculus GN=Qki
Samd14	Q8K070	1	1	65504	NA	20919	16627	NA	NA	20206	NA	25758	49201	0.892557	0.049364	34350	31721.67	Down	-0.11484	0.973648	Sterile alpha motif domain-containing protein 14 OS=Mus musculus GN=Samd14 PE=1 SV=1
Osbp	Q3B7Z2	1	1	101016	158481	137047	167489	197382	153852	148334	189807	138555	146209	0.872783	0.059094	152283	155351.4	Up	0.02878	0.970475	Oxysterol-binding protein 1 OS=Mus musculus GN=Osbp PE=1 SV=3
Map1a	Q9QYR6-2	35	35	228397.4	163905.1	178566.9	164147.9	213273.5	187746.6	188664.8	217626.2	183998.9	159016.2	0.893253	0.049026	189658.1	187410.5	Down	-0.0172	0.973802	Isoform 2 of Microtubule-associated protein 1A OS=Mus musculus GN=Map1a
Dennd1a	Q8K382	4	4	99213	189200.5	150432.3	145964.3	193539	196457	175023	139812.8	126897.3	157336	0.87526	0.057863	155669.8	159105.2	Up	0.031492	0.970702	DENN domain-containing protein 1A OS=Mus musculus GN=Dennd1a PE=1 SV=2
Lmna	P48678-3	7	7	97765.33	178379.5	228513.3	267836.3	361084.2	99641.67	253929.2	263330.3	287278	274134.2	0.876993	0.057004	226715.7	235662.7	Up	0.055839	0.970839	Isoform C2 of Prelamin-A/C OS=Mus musculus GN=Lmna
8-Sep	Q8CHH9	9	9	89569.38	181220.8	107605.8	133380	134393.3	124415.6	111048.9	131776.3	119632.9	147702.4	0.894526	0.048407	129233.8	126915.2	Down	-0.02612	0.973946	Septin-8 OS=Mus musculus GN=Sept8 PE=1 SV=4
Dlgap3	Q6PFD5	3	3	745034.7	631942.7	570827.7	621229	635771.3	738511.3	614562.3	594723	695831.7	529359	0.895489	0.04794	640961.1	634597.5	Down	-0.01439	0.974072	Disks large-associated protein 3 OS=Mus musculus GN=Dlgap3 PE=1 SV=1
Rtn2	O70622	1	1	57473	107774	135088	129054	141286	131862	117207	117210	84689	107997	0.895686	0.047844	114135	111793	Down	-0.02991	0.974072	Reticulon-2 OS=Mus musculus GN=Rtn2 PE=1 SV=1
Rps6ka2	Q9WUT3	1	1	NA	23592	63516	42027	57708	41682	47090	57900	41275	38946	0.896219	0.047586	46710.75	45378.6	Down	-0.04174	0.974072	Ribosomal protein S6 kinase alpha-2 OS=Mus musculus GN=Rps6ka2 PE=1 SV=1
Sars2	Q9JJL8	1	1	289919	373964	321373	476408	301851	457521	335146	396276	310628	299656	0.878326	0.056344	352703	359845.4	Up	0.028923	0.970839	Serine--tRNA ligase, mitochondrial OS=Mus musculus GN=Sars2 PE=1 SV=2
Eif4h	Q9WUK2	11	11	12434.62	38702.88	50094.5	41022.62	39844.25	30223.5	47006.5	40295	42408.12	27981.5	0.878343	0.056336	36419.77	37582.92	Up	0.045355	0.970839	Eukaryotic translation initiation factor 4H OS=Mus musculus GN=Eif4h PE=1 SV=3
Rapgef2	Q8CHG7	9	9	72581.33	57695.17	61873	63581.83	69478.67	84993.83	54533.5	79905.5	65123	46806.83	0.879775	0.055628	65042	66272.53	Up	0.027039	0.970839	Rap guanine nucleotide exchange factor 2 OS=Mus musculus GN=Rapgef2 PE=1 SV=2
Rpl38	Q9JJI8	5	5	820512.2	801471	537261.8	620771.6	627424.2	854822	693634.6	676598.4	600396.4	637071.4	0.879834	0.055599	681488.2	692504.6	Up	0.023135	0.970839	60S ribosomal protein L38 OS=Mus musculus GN=Rpl38 PE=2 SV=3
Afg3l2	Q8JZQ2	4	4	41596.25	47216.75	78354.5	83689	90862.75	104747.5	61064	76197.5	65645.75	44918.25	0.88147	0.054792	68343.85	70514.6	Up	0.04511	0.970921	AFG3-like protein 2 OS=Mus musculus GN=Afg3l2 PE=1 SV=1
Ppp1r13b	Q62415	3	3	20277.67	29336.33	23008.67	22663.33	18351.67	26354.33	23854	18413.33	22800.33	23967	0.881519	0.054768	22727.53	23077.8	Up	0.022064	0.970921	Apoptosis-stimulating of p53 protein 1 OS=Mus musculus GN=Ppp1r13b PE=2 SV=2
Tsfm	Q9CZR8	7	7	16253.17	36240.33	46440.5	49138	45941.33	47356.33	42774.17	36902	29348.67	33088.67	0.898884	0.046296	38802.67	37893.97	Down	-0.03419	0.974072	Elongation factor Ts, mitochondrial OS=Mus musculus GN=Tsfm PE=1 SV=1
Cask	O70589-4	3	3	244380.5	134435	218370	153096.5	164383.5	205263.5	193015	185677.5	198663	149749.5	0.882607	0.054233	182933.1	186473.7	Up	0.027656	0.971517	Isoform 4 of Peripheral plasma membrane protein CASK OS=Mus musculus GN=Cask
Rrbp1	Q99PL5	2	2	29927.5	38945	54127.5	51048	77686.5	46873.5	44789.5	65902	48861.5	39212	0.899325	0.046083	50346.9	49127.7	Down	-0.03537	0.974072	Ribosome-binding protein 1 OS=Mus musculus GN=Rrbp1 PE=1 SV=2
Kif7	B7ZNG0	1	1	224194	137307	140268	104198	123612	150503	132217	135253	175453	121222	0.899582	0.045959	145915.8	142929.6	Down	-0.02983	0.974072	Kinesin-like protein KIF7 OS=Mus musculus GN=Kif7 PE=1 SV=1
Mrpl11	Q9CQF0	1	1	NA	58360	81338	72911	34898	95759	48844	68436	50159	56438	0.882818	0.054129	61876.75	63927.2	Up	0.047032	0.971517	39S ribosomal protein L11, mitochondrial OS=Mus musculus GN=Mrpl11 PE=2 SV=1

Sgta	Q8BJU0	1	1	445630	298394	446610	331773	389514	409704	402044	420263	348162	357065	0.883939	0.053578	382384.2	387447.6	Up	0.018978	0.971812 Small glutamine-rich tetratricopeptide repeat-containing protein alpha OS=Mus musculus GN=Sgta PE=1 SV=2
Rph3a	P47708	12	12	204184	216655.4	190002.3	203712.6	148693.2	208224.2	193656.4	181369.6	205886.2	183984.4	0.884411	0.053346	192649.5	194024.2	Up	0.014712	0.971812 Rabphilin-3A OS=Mus musculus GN=Rph3a PE=1 SV=2
Nrgn	P60761	4	4	7977.25	6957	6295.5	6231	6951.75	4678	7621.75	8650.75	6295.25	6702.5	0.90399	0.043836	6882.5	6789.65	Down	-0.0196	0.976438 Neurogranin OS=Mus musculus GN=Nrgn PE=1 SV=1
Pnpt1	Q8K1R3	1	1	247501	116116	120597	130287	110058	159467	143057	128879	145368	130973	0.904444	0.043618	144911.8	141548.8	Down	-0.03388	0.976438 Polyribonucleotide nucleotidyltransferase 1, mitochondrial OS=Mus musculus GN=Pnpt1 PE=1 SV=1
Fip1l1	Q9D824-3	2	2	NA	41503.5	90188	31377.5	150773.5	81416	82779	46469.5	77215	86180	0.904456	0.043613	78460.63	74811.9	Down	-0.0687	0.976438 Isoform 3 of Pre-mRNA 3'-end-processing factor FIP1 OS=Mus musculus GN=Fip1l1
Agfg2	Q80WC7	5	5	122229.5	106900	82958.5	96415	124866.5	131688	102832.5	115687.5	100327	80924	0.88713	0.052013	104673.9	106291.8	Up	0.022129	0.972747 Arf-GAP domain and FG repeat-containing protein 2 OS=Mus musculus GN=Agfg2 PE=1 SV=1
Hk1	P17710-3	19	19	194131.6	253907.1	306950.5	266214.7	251573.1	228648.6	307766.3	236237.8	277192.4	207143.8	0.904815	0.04344	254555.4	251397.7	Down	-0.01801	0.976438 Isoform HK1 of Hexokinase-1 OS=Mus musculus GN=Hk1
Icam5	Q60625	9	9	1313317	838602.1	761252.3	702050.9	771925.6	1038072	887610.4	853811.6	806857.9	724932.3	0.905759	0.042987	877429.6	862256.8	Down	-0.02517	0.976546 Intercellular adhesion molecule 5 OS=Mus musculus GN=Icam5 PE=1 SV=2
Agfg2	Q80WC7-2	2	2	122229.5	106900	82958.5	96415	124866.5	131688	102832.5	115687.5	100327	80924	0.88713	0.052013	104673.9	106291.8	Up	0.022129	0.972747 Isoform 2 of Arf-GAP domain and FG repeat-containing protein 2 OS=Mus musculus GN=Agfg2
Fam171a2	A2A699	2	2	281589.5	330405.5	331956.5	317129	394074.5	368918	358200	331515	318760.5	294342.5	0.887352	0.051904	331031	334347.2	Up	0.014381	0.972747 Protein FAM171A2 OS=Mus musculus GN=Fam171a2 PE=1 SV=1
Lhpp	Q9D7I5	1	1	27141	27731	30692	28487	19288	31750	21893	33853	16440	27121	0.906438	0.042662	26667.8	26211.4	Down	-0.0249	0.976546 Phospholysine phosphohistidine inorganic pyrophosphate phosphatase OS=Mus musculus GN=Lhpp PE=2 SV=1
Palm2	Q8BR92	1	1	194282	372445	365925	319536	333544	375817	271078	284162	324384	307760	0.907041	0.042373	317146.4	312640.2	Down	-0.02065	0.97678 Paralemmin-2 OS=Mus musculus GN=Palm2 PE=1 SV=1
Scg5	P12961	1	1	132465	71806	84730	71285	103506	139397	97665	91740	81313	65851	0.888733	0.051229	92758.4	95193.2	Up	0.037381	0.972747 Neuroendocrine protein 7B2 OS=Mus musculus GN=Scg5 PE=1 SV=1
Fam120a	Q6A0A9	4	4	50660	90053.75	155569.3	131043	91586.75	60207.5	115929.5	129128.5	107672	121463.5	0.891282	0.049985	103782.6	106880.2	Up	0.042431	0.973648 Constitutive coactivator of PPAR-gamma-like protein 1 OS=Mus musculus GN=FAM120A PE=1 SV=2
Strn	O55106	4	4	392296.3	347910	275075	351416.3	293773.7	326027.7	321394.3	326762	333973	339326.3	0.90905	0.041412	332094.3	329496.7	Down	-0.01133	0.977079 Striatin OS=Mus musculus GN=Strn PE=1 SV=2
Hkdc1	Q91W97	2	2	406865.5	753098	838875.5	602613.5	704145.5	533573.5	970763	472325	733124	526056.5	0.909213	0.041334	661119.6	647168.4	Down	-0.03077	0.977079 Putative hexokinase HKDC1 OS=Mus musculus GN=Hkdc1 PE=2 SV=1
Tpr	F6ZDS4	1	1	75216	50625	78605	46411	99498	50480	75050	90792	72242	54597	0.909221	0.041331	70071	68632.2	Down	-0.02993	0.977079 Nucleoprotein TPR OS=Mus musculus GN=Tpr PE=1 SV=1
Chl1	P70232	3	3	349290	263022.7	281243.3	245546	238538.3	287736.7	319292.3	289749.7	259794.7	204935	0.910139	0.040892	275528.1	272301.7	Down	-0.01699	0.97754 Neural cell adhesion molecule L1-like protein OS=Mus musculus GN=Chl1 PE=1 SV=2
Ppa2	Q91VM9	1	1	NA	12549	NA	18172	NA	NA	NA	[illegible]	[illegible]	10781	0.891403	0.049926	15360.5	16366.5	Up	0.091521	0.973648 Inorganic pyrophosphatase 2, mitochondrial OS=Mus musculus GN=Ppa2 PE=1 SV=1
Hint1	P70349	6	6	336058.6	350293.6	445597.2	448507.8	375979	367342.8	422860.2	418305.6	412259	317787.4	0.911181	0.040395	391287.2	387711	Down	-0.01325	0.977948 Histidine triad nucleotide-binding protein 1 OS=Mus musculus GN=Hint1 PE=1 SV=3
Dlgap1	Q9D415-6	2	2	1110337	952129.5	860864.5	990135	1053163	1131372	989167	900232.5	1100261	802596.5	0.911556	0.040217	993325.7	984725.7	Down	-0.01254	0.977948 Isoform 6 of Disks large-associated protein 1 OS=Mus musculus GN=Dlgap1
Clip2	Q9Z0H8	9	9	18210	55309.38	36402.25	38687.38	38353	34412.38	40553.75	40345.5	36082.75	39867.12	0.892732	0.049279	37392.4	38252.3	Up	0.032801	0.973648 CAP-Gly domain-containing linker protein 2 OS=Mus musculus GN=Clip2 PE=1 SV=2
Fbl	P35550	1	1	625168	517936	654722	625934	813158	410363	660208	732096	695908	696391	0.914043	0.039033	647383.6	638993.2	Down	-0.01882	0.978096 rRNA 2'-O-methyltransferase fibrillarin OS=Mus musculus GN=Fbl PE=2 SV=2
Dpysl2	O08553	97	97	1270874	1109599	855224.7	1006838	959802.5	1030114	964080.5	887549.8	1046056	1224322	0.914163	0.038976	1040468	1030424	Down	-0.01399	0.978096 Dihydropyrimidinase-related protein 2 OS=Mus musculus GN=Dpysl2 PE=1 SV=2
Pip5k1c	O70161-3	6	6	95976.8	86590.4	91824	83898	99303.6	114284.6	86999.2	103520.4	79216	78891	0.893674	0.048821	91518.56	92582.24	Up	0.016671	0.973846 Isoform 3 of Phosphatidylinositol 4-phosphate 5-kinase type-1 gamma OS=Mus musculus GN=Pip5k1c
Psmc3	O88685	7	7	60417.86	71347	94685.71	78340.86	92947.14	92384	80749.29	95137.71	71868.71	63648.43	0.894343	0.048496	79547.71	80757.63	Up	0.021778	0.973946 26S protease regulatory subunit 6A OS=Mus musculus GN=Psmc3 PE=1 SV=2
A830010M	Q80TK0	4	4	207800.3	118566.5	100857.5	105505.3	130861.8	150913.5	117712.8	145865	113706.3	123730.5	0.915352	0.038412	132718.3	130385.6	Down	-0.02558	0.978096 Uncharacterized protein KIAA1107 OS=Mus musculus GN=Kiaa1107 PE=2 SV=5
Prkcsh	O08795	2	2	NA	25806	17390	17410	26272	16034	7738	24062	27041	37548	0.896532	0.047434	21719.5	22484.6	Up	0.049946	0.974072 Glucosidase 2 subunit beta OS=Mus musculus GN=Prkcsh PE=1 SV=1
Rab3a	P63011	2	2	NA	26311	60492	70808	NA	NA	35130	150545	16897	26278	0.897551	0.046941	52537	57212.5	Up	0.122997	0.974072 Ras-related protein Rab-3A OS=Mus musculus GN=Rab3a PE=1 SV=1
Dennd4b	Q3U1Y4	1	1	NA	798	724	1701	840	652	1943	684	551	1488	0.897863	0.04679	1015.75	1063.6	Up	0.06641	0.974072 DENN domain-containing protein 4B OS=Mus musculus GN=Dennd4b PE=2 SV=2
Stam	P70297	3	3	NA	66061	153095	130808	230640	184801	133156	168071	138610	125703	0.89789	0.046777	145151	150068.2	Up	0.048064	0.974072 Signal transducing adapter molecule 1 OS=Mus musculus GN=Stam PE=2 SV=3
Napb	P28663	5	5	67543.6	69236.4	62802.4	68502.2	69079.4	44444.2	61547.6	67849.2	86386.8	82111.6	0.898208	0.046623	67432.8	68467.88	Up	0.021977	0.974072 Beta-soluble NSF attachment protein OS=Mus musculus GN=Napb PE=1 SV=2
Hnrnpl	Q8R081	7	7	183416.7	138093.2	211610.5	161493.2	281626	131718	194852.7	232454.2	221773.7	178918	0.916667	0.037788	195247.9	191943.3	Down	-0.02463	0.978096 Heterogeneous nuclear ribonucleoprotein L OS=Mus musculus GN=Hnrnpl PE=1 SV=2
Baiap2	Q8BKX1-4	23	23	397202.8	493702.6	391229.5	382702.3	467380.4	461238.6	435799.1	429113.2	396145.3	426516.6	0.898502	0.046481	426443.5	429762.5	Up	0.011185	0.974072 Isoform 4 of Brain-specific angiogenesis inhibitor 1-associated protein 2 OS=Mus musculus GN=Baiap2
Ckb	Q04447	43	43	212437.7	161630.1	164699.6	165852.1	147776.6	166762.7	180177.3	186616.7	170839.5	156069.4	0.899168	0.046159	170479.2	172093.1	Up	0.013594	0.974072 Creatine kinase B-type OS=Mus musculus GN=Ckb PE=1 SV=1
Lrrc47	Q505F5	2	2	217524	223775	189214	196405	249565	286026	253904	191434	162704	198553	0.901076	0.045239	215296.6	218524.2	Up	0.021468	0.975278 Leucine-rich repeat-containing protein 47 OS=Mus musculus GN=Lrrc47 PE=2 SV=1
Sncb	Q91ZZ3	22	22	284950.2	377058.9	283851.9	315867.1	295480.1	338268.3	335759.6	293248.1	290593.4	337620	0.902642	0.044484	311441.6	314297.9	Up	0.013171	0.976438 Beta-synuclein OS=Mus musculus GN=Sncb PE=1 SV=1
Smc3	Q9CW03	1	1	291958	305332	340620	284227	456083	331929	369260	334028	325419	299552	0.918554	0.036895	335644	332037.6	Down	-0.01559	0.978493 Structural maintenance of chromosomes protein 3 OS=Mus musculus GN=Smc3 PE=1 SV=2
Cops2	P61202	1	1	153292	84062	77792	84369	79269	133818	90252	83529	104155	78118	0.902925	0.044348	95756.8	97974.4	Up	0.03303	0.976438 COP9 signalosome complex subunit 2 OS=Mus musculus GN=Cops2 PE=1 SV=1
Actn4	P57780	15	15	96000.92	90303.62	124838.2	130804.3	146956.6	114444.9	137852	122379.9	114056.6	107629.9	0.904619	0.043534	117780.7	119272.7	Up	0.01816	0.976438 Alpha-actinin-4 OS=Mus musculus GN=Actn4 PE=1 SV=1
Gstp2	P46425	3	3	77201	30225.5	43873.5	30695.5	43271.5	59213	41827.5	58310	30135	30244	0.920248	0.036095	45053.4	43945.9	Down	-0.03591	0.978741 Glutathione S-transferase P 2 OS=Mus musculus GN=Gstp2 PE=2 SV=2
Myh14	Q6URW6-3	5	5	47419.67	134210.3	129040	171516	130150	136064.7	119424.3	110651.3	105348.3	129688	0.920314	0.036064	122467.2	120235.3	Down	-0.02653	0.978741 Isoform 3 of Myosin-14 OS=Mus musculus GN=Myh14
Hba-a1	P01942	16	16	2783502	1849290	1081005	1252936	1496806	1223641	1494625	1523429	1795683	2247811	0.921483	0.035513	1692708	1657038	Down	-0.03073	0.979577 Hemoglobin subunit alpha OS=Mus musculus GN=Hba PE=1 SV=2
Ap3m1	Q9JKC8	1	1	295772	344811	329121	375671	313129	262261	384216	257633	439348	295576	0.92325	0.034681	331700.8	327806.8	Down	-0.01704	0.980814 AP-3 complex subunit mu-1 OS=Mus musculus GN=Ap3m1 PE=1 SV=1
Camta2	Q80Y50-3	1	1	219356	142080	131647	125616	183414	155334	159185	182677	137453	157756	0.923412	0.034604	160422.6	158481	Down	-0.01757	0.980814 Isoform 3 of Calmodulin-binding transcription activator 2 OS=Mus musculus GN=Camta2
Qk	Q9QYS9-3	7	7	123395.7	135361.5	179348.7	169273.8	171810.7	180689.2	145279.8	185545.7	121003.5	138447.7	0.923969	0.034343	155838.1	154193.2	Down	-0.01531	0.980999 Isoform 3 of Protein quaking OS=Mus musculus GN=Qki
Mccc2	Q3ULD5	4	4	NA	18011	39751.5	17594.75	20657.75	15964	24613.25	NA	23526.75	29536.5	0.925266	0.033733	24003.75	23410.13	Down	-0.03613	0.981292 Methylcrotonoyl-CoA carboxylase beta chain, mitochondrial OS=Mus musculus GN=Mccc2 PE=1 SV=1
Maob	Q8BW75	4	4	69422	68411	75401.5	73520.5	63539.5	76572	76252.25	70040.75	68906.25	63942.25	0.906238	0.042758	70058.9	70416.7	Up	0.007349	0.976546 Amine oxidase [flavin-containing] B OS=Mus musculus GN=Maob PE=1 SV=4
Pls3	Q99K51	1	1	NA	8536	7038	6551	8236	NA	5470	4716	7500	13742	0.906319	0.042719	7590.25	7857	Up	0.049831	0.976546 Plastin-3 OS=Mus musculus GN=Pls3 PE=1 SV=3
Rabif	Q91X96	1	1	684778	587705	822593	442707	536567	795709	732173	742284	367355	381559	0.925776	0.033494	614870	603816	Down	-0.02617	0.981292 Guanine nucleotide exchange factor MSS4 OS=Mus musculus GN=Rabif PE=2 SV=1
Ldhb	P16125	9	9	116274.8	211074.9	253386.4	209412.9	176712.5	153507.5	165606.9	253462.6	169492.5	210880.8	0.926854	0.032989	193372.3	190590.1	Down	-0.02091	0.98197 L-lactate dehydrogenase B chain OS=Mus musculus GN=Ldhb PE=1 SV=2
Strn3	Q9ERG2	2	2	548766	536972	424324.5	561196	444462.5	591176.5	508740.5	447334.5	473984	476632	0.927182	0.032835	503144.2	499573.5	Down	-0.01027	0.98197 Striatin-3 OS=Mus musculus GN=Strn3 PE=1 SV=1
Ndufs3	Q9DCT2	3	3	594072.3	575068.7	611174.3	705547	474708.3	438582.7	601400	671951.3	654708.7	568016.7	0.927991	0.032456	592114.1	586931.9	Down	-0.01268	0.982086 NADH dehydrogenase [ubiquinone] iron-sulfur protein 3, mitochondrial OS=Mus musculus GN=Ndufs3 PE=1 SV=2
Dbt	P53395	1	1	NA	169656	81883	163305	70700	105793	136867	111091	98940	172273	0.907651	0.042081	121386	124992.8	Up	0.042243	0.97703 Lipoamide acyltransferase component of branched-chain alpha-keto acid dehydrogenase complex, mitochondrial OS=Mus musculus GN=Dbt PE=1 SV=2
Tuba8	Q9JJZ2	55	55	301933.5	330617.8	404132.7	318635.3	384006.1	390564.1	326615.1	307447.7	367780.6	361666.6	0.908034	0.041898	347865.1	350814.8	Up	0.012182	0.977032 Tubulin alpha-8 chain OS=Mus musculus GN=Tuba8 PE=1 SV=1
Prkacb	P68181-3	1	1	25750	18642	22101	20308	23882	29500	20351	22799	19671	17228	0.928824	0.032067	22136.6	21909.8	Down	-0.01486	0.982086 Isoform 3 of cAMP-dependent protein kinase catalytic subunit beta OS=Mus musculus GN=Prkacb
Hap1	O35668	2	2	46255.5	61200.5	56176	49628	70085	89038	42694.5	45615.5	54268.5	57177.5	0.910413	0.040762	56669	57758.8	Up	0.027481	0.97754 Huntingtin-associated protein 1 OS=Mus musculus GN=Hap1 PE=1 SV=1
Zcchc8	Q9CYA6	1	1	NA	9426	21429	14690	23295	45681	5907	10193	18600	10135	0.913701	0.039196	17210	18103.2	Up	0.072998	0.978096 Zinc finger CCHC domain-containing protein 8 OS=Mus musculus GN=Zcchc8 PE=2 SV=3
Ube2n	P61089	2	2	560813	330667	291741	419963	480374	419540	438671	412074	464372	379495	0.914669	0.038736	416711.6	422470.4	Up	0.019801	0.978096 Ubiquitin-conjugating enzyme E2 N OS=Mus musculus GN=Ube2n PE=1 SV=1
Stim2	P83093	1	1	200858	107023	104131	88245	139602	159271	129871	143091	120623	99472	0.915225	0.038472	127971.8	130465.6	Up	0.027844	0.978096 Stromal interaction molecule 2 OS=Mus musculus GN=Stim2 PE=1 SV=2
Cnpy4	Q8BQ47	1	1	449204	349689	261927	254710	309797	358197	318108	339717	351973	278617	0.915598	0.038295	325065.4	329322.4	Up	0.018771	0.978096 Protein canopy homolog 4 OS=Mus musculus GN=Cnpy4 PE=1 SV=1
Map2k1	P31938	3	3	406112	387746	420279.3	398912	319921.3	367533.3	411273.7	363000.3	394380.7	388149.3	0.932733	0.030243	386594.1	384867.5	Down	-0.00646	0.983417 Dual specificity mitogen-activated protein kinase kinase 1 OS=Mus musculus GN=Map2k1 PE=1 SV=2
Gng7	Q61016	2	2	177273	63232	200451	160988	NA	110026	180072	76588	210463	197270	0.915648	0.038271	150486	154883.8	Up	0.041557	0.978096 Guanine nucleotide-binding protein G(I)/G(S)/G(O) subunit gamma-7 OS=Mus musculus GN=Gng7 PE=2 SV=2
Rbm39	Q8VH51-3	1	1	378523	190522	313363	287480	244721	308829	304818	292220	286661	206030	0.933241	0.030006	282921.8	279711.6	Down	-0.01646	0.983511 Isoform 3 of RNA-binding protein 39 OS=Mus musculus GN=Rbm39
Ide	Q9JHR7	1	1	138832	584113	563808	613239	411760	436241	494207	502297	405417	434379	0.934444	0.029447	462350.4	454508.2	Down	-0.02468	0.983847 Insulin-degrading enzyme OS=Mus musculus GN=Ide PE=1 SV=1
Coro2b	Q8BH44	2	2	422961.5	339471.5	245873	334956.5	236899.5	281553.5	301880	303924	364718.5	348933.5	0.915796	0.038201	316032.4	320201.9	Up	0.018909	0.978096 Coronin-2B OS=Mus musculus GN=Coro2b PE=2 SV=2
Mpp1	P70290	1	1	NA	42240	38000	32573	18931	27894	40991	35110	30425	27815	0.934888	0.02924	32936	32447	Down	-0.02158	0.983847 55 kDa erythrocyte membrane protein OS=Mus musculus GN=Mpp1 PE=1 SV=1
Gk	Q64516-2	3	3	231286.7	256974.7	266542.7	295402.3	307194.3	387784.3	282983	259533.3	205937	239741	0.916158	0.03803	271480.1	275195.7	Up	0.019611	0.978096 Isoform 2 of Glycerol kinase OS=Mus musculus GN=Gk
Dnm1l	Q8K1M6-4	11	11	344076.8	351592.2	326177.6	323254.8	317819.7	341143	282594.1	368079.9	331228.8	348483.1	0.916364	0.037932	332584.2	334305.8	Up	0.007449	0.978096 Isoform 4 of Dynamin-1-like protein OS=Mus musculus GN=Dnm1l
Cend1	Q9JKC6	4	4	23855.75	23314.75	20191.75	31456	31633.75	35107.5	24227.25	23569.25	25034.5	21141.75	0.936599	0.028449	26090.4	25816.05	Down	-0.01525	0.984512 Cell cycle exit and neuronal differentiation protein 1 OS=Mus musculus GN=Cend1 PE=1 SV=1
Rpl30	P62889	6	6	684678.3	321423.7	293810.5	261714.2	414939.7	456360.5	330370.8	365588	436262.3	354912.5	0.937838	0.027872	395313.3	388698.8	Down	-0.02434	0.984512 60S ribosomal protein L30 OS=Mus musculus GN=Rpl30 PE=2 SV=2
Fignl1	Q8BPY9	1	1	NA	79039	84850	86829	77191	33038	107652	72734	90548	114005	0.917501	0.037393	81977.25	83595.4	Up	0.0282	0.978096 Fidgetin-like protein 1 OS=Mus musculus GN=Fignl1 PE=2 SV=1
Fgb	Q8K0E8	2	2	46753.5	104066.5	126095	122588	127818.5	52235.5	99463.5	149029.5	111066	106829	0.938189	0.02771	105464.3	103724.7	Down	-0.024	0.984512 Fibrinogen beta chain OS=Mus musculus GN=Fgb PE=2 SV=1
Apoa4	P06728	1	1	76203	150768	107013	94209	109084	139621	81409	103907	88019	117857	0.938317	0.02765	107455.4	106162.6	Down	-0.01746	0.984512 Apolipoprotein A-IV OS=Mus musculus GN=Apoa4 PE=1 SV=3
Kbtbd11	Q8BNW9	5	5	126508.2	138618.4	138587.6	153155.4	160845.2	146421.8	159591.6	150030.8	124863.2	133454.6	0.939927	0.026906	143543	142872.4	Down	-0.00676	0.984512 Kelch repeat and BTB domain-containing protein 11 OS=Mus musculus GN=Kbtbd11 PE=1 SV=3
Map7	O88735	1	1	NA	13645	4355	18573	9516	NA	10558	13216	NA	NA	0.917561	0.037365	11522.25	11887	Up	0.044962	0.978096 Ensconsin OS=Mus musculus GN=Map7 PE=1 SV=1
Nudt21	Q9CQF3	4	4	56971.25	67958.75	101538	91435.25	133874.5	66673	99234	116417.3	85761.25	77476.5	0.940384	0.026695	90355.55	89112.4	Down	-0.01999	0.984512 Cleavage and polyadenylation specificity factor subunit 5 OS=Mus musculus GN=Nudt21 PE=1 SV=1
Tdrd6	P61407	1	1	23527	19789	17450	15445	19178	20345	18442	20970	18701	17728	0.917793	0.037255	19077.8	19237.2	Up	0.012004	0.978096 Tudor domain-containing protein 6 OS=Mus musculus GN=Tdrd6 PE=1 SV=1
Arhgef9	Q3UTH8-4	1	1	NA	27848	16893	56044	81350	57289	36904	25125	63676	53496	0.9178	0.037252	45533.75	47298	Up	0.054843	0.978096 Isoform 4 of Rho guanine nucleotide exchange factor 9 OS=Mus musculus GN=Arhgef9
Rgs7bp	Q8BQP9	1	1	74972	151987	176543	277177	210894	202724	142916	172518	176844	183156	0.941573	0.026146	178314.6	175631.6	Down	-0.02187	0.984512 Regulator of G-protein signaling 7-binding protein OS=Mus musculus GN=Rgs7bp PE=1 SV=1
Acbd6	Q9D061-3	1	1	630058	324258	406120	267241	379412	453473	372644	337455	402442	416039	0.941692	0.026091	401417.8	396410.6	Down	-0.01811	0.984512 Isoform 3 of Acyl-CoA-binding domain-containing protein 6 OS=Mus musculus GN=Acbd6
Brk1	Q91VR8	5	5	151495.4	86529.4	110214.4	121684.4	137709.6	125710.2	139584.6	110134.2	118384.8	109033.2	0.941699	0.026088	121526.6	120569.4	Down	-0.01141	0.984512 Protein BRICK1 OS=Mus musculus GN=Brk1 PE=2 SV=1
Adam22	Q9R1V6-1	6	6	94138.2	198012.2	225122	219678.6	245291	205784	214840.4	196594.4	194405.2	185231	0.919093	0.036641	196448.4	199371	Up	0.021305	0.97866 Isoform 9 of Disintegrin and metalloproteinase domain-containing protein 22 OS=Mus musculus GN=Adam22
Sema7a	Q9QUR8	1	1	NA	16362	15357	17519	17717	26367	17251	3521	18043	17086	0.942258	0.02583	16738.75	16453.6	Down	-0.02479	0.984512 Semaphorin-7A OS=Mus musculus GN=Sema7a PE=1 SV=1
Uqcrc1	Q9CZ13	13	13	30212.6	74355.8	98306.6	98590.6	95210.4	74361.2	85283.5	86413.4	88000	69815	0.920011	0.036207	79335.2	80774.62	Up	0.025941	0.978741 Cytochrome b-c1 complex subunit 1, mitochondrial OS=Mus musculus GN=Uqcrc1 PE=1 SV=2
Dhrs4	Q99LB2	2	2	22318	73880.5	88319.5	86771.5	104323	57251	74393	97133.5	74420.5	79965.5	0.925508	0.03362	75122.5	76632.7	Up	0.028715	0.981292 Dehydrogenase/reductase SDR family member 4 OS=Mus musculus GN=Dhrs4 PE=1 SV=3
Hist1h4h	P62806	9	9	713401.6	828064.3	1383343	1205757	2201043	329484.4	1407764	1286077	1885317	1602360	0.925656	0.03355	1266322	1302200	Up	0.040308	0.981292 Histone H4 OS=Mus musculus GN=Hist1h4a PE=1 SV=2
Tmem263	Q9DAM7	9	9	144887.1	151951.3	117204.6	115084.6	117882.4	154239.8	132380.8	119109.8	122072.7	115586.3	0.946775	0.023753	129402	128677.9	Down	-0.0081	0.987791 UPF0444 transmembrane protein C12orf23 homolog OS=Mus musculus PE=2 SV=1
Vps4a	Q8VEJ9	2	2	NA	48913.5	57790	52561.5	63573.5	40503.5	67987.5	49683	53312.5	65000	0.947042	0.023631	55709.63	55911.9	Up	0.005274	0.987791 Vacuolar protein sorting-associated protein 4A OS=Mus musculus GN=Vps4a PE=1 SV=1
Mast3	Q3U214	4	4	NA	16404	22401	14783	23307.67	9426.33	18386.67	21839.67	21092.67	24232	0.947323	0.023502	19223.92	18995.47	Down	-0.01725	0.987791 Microtubule-associated serine/threonine-protein kinase 3 OS=Mus musculus GN=Mast3 PE=1 SV=3
Npm1	Q61937	7	7	895164	425504.3	493920.5	364664.5	383369.3	577379.8	475906.8	676762	390937.5	492729.5	0.928797	0.032079	512524	522743.1	Up	0.028483	0.982086 Nucleophosmin OS=Mus musculus GN=Npm1 PE=1 SV=1
Camkk1	Q8VBY2	2	2	133364.5	93165	79391	75536	146792	112147.5	114899.5	106176	80036.5	109609.5	0.94821	0.023095	105649.7	104573.8	Down	-0.01477	0.987872 Calcium/calmodulin-dependent protein kinase kinase 1 OS=Mus musculus GN=Camkk1 PE=1 SV=1
Rcn2	Q8BP92	9	9	NA	7188	5954.33	7978	6501.17	NA	5093.5	15170.67	4494.83	3911.83	0.928806	0.032075	6905.375	7167.708	Up	0.053792	0.982086 Reticulocalbin-2 OS=Mus musculus GN=Rcn2 PE=2 SV=1
Myef2	Q8C854	4	4	141807.3	118873.5	150486.3	147961.3	209711	57470.5	160302.3	138344.8	235267	166845.8	0.949755	0.022388	153767.8	151646.1	Down	-0.02005	0.988385 Myelin expression factor 2 OS=Mus musculus GN=Myef2 PE=1 SV=1
Trim9	Q8C7M3-4	9	9	50392.22	80885.22	80596.22	64319.56	95694.22	72805.78	67375.11	88063.11	72863.56	74733.67	0.929288	0.03185	74377.49	75168.25	Up	0.015257	0.982171 Isoform 4 of E3 ubiquitin-protein ligase TRIM9 OS=Mus musculus GN=Trim9
Farsa	Q8C0C7	3	3	533631	264669	357583	337958	281604	323200	384676	323403	388083	379228	0.930145	0.031449	355089	359718	Up	0.018686	0.982672 Phenylalanine--tRNA ligase alpha subunit OS=Mus musculus GN=Farsa PE=2 SV=1
Nudt5	Q9JKX6	1	1	346804	518540	470248	367230	457783	511085	378978	395688	431824	430498	0.951313	0.021677	432121	429614.6	Down	-0.00839	0.988461 ADP-sugar pyrophosphatase OS=Mus musculus GN=Nudt5 PE=1 SV=1
Ighm	P01872	2	2	25446.5	53888	81878	54639	80558.5	10547.5	56097.5	97076.5	69780.5	57444.5	0.951693	0.021503	59282	58189.3	Down	-0.02684	0.988461 Ig mu chain C region OS=Mus musculus GN=Ighm PE=1 SV=2
Arhgap21	Q6DFV3	1	1	74466	73398	56952	67560	62219	87050	67434	63913	50089	63966	0.951743	0.02148	66919	66490.4	Down	-0.00927	0.988461 Rho GTPase-activating protein 21 OS=Mus musculus GN=Arhgap21 PE=1 SV=1
Slc25a25	A2ASZ8-3	1	1	94355	174845	216961	269005	168258	171518	202766	203803	180623	178298	0.930958	0.03107	184684.8	187401.6	Up	0.021068	0.982895 Isoform 3 of Calcium-binding mitochondrial carrier protein SCaMC-2 OS=Mus musculus GN=Slc25a25
Itgav	P43406	1	1	580164	700807	744307	532323	754438	654455	576336	814713	734434	510563	0.95277	0.021012	662407.8	658100.2	Down	-0.00941	0.989044 Integrin alpha-V OS=Mus musculus GN=Itgav PE=1 SV=2
Slitrk3	Q8108 9	1	1	277355	813873	539674	657356	688233	699169	612229	511816	509911	688349	0.931124	0.030992	595298.2	604294.8	Up	0.02164	0.982895 SLIT and NTRK-like protein 3 OS=Mus musculus GN=Slitrk3 PE=2 SV=2
Csnk2a1	Q60737	2	2	NA	28365	27988.5	23269.5	36186	31204.5	26689.5	34812	26465	27000.5	0.931834	0.030661	28952.25	29234.3	Up	0.013987	0.983241 Casein kinase II subunit alpha OS=Mus musculus GN=Csnk2a1 PE=1 SV=2
Rps11	P62281	9	9	595626.1	310853.9	223166.8	249035.3	312708.1	399675	323023	325760.9	341680.8	280822.2	0.955474	0.019781	338278	334192.4	Down	-0.01753	0.990298 40S ribosomal protein S11 OS=Mus musculus GN=Rps11 PE=1 SV=3
Pde4d	Q01063-2	1	1	327588	358671	211311	468696	328263	159456	329632	459466	377334	350187	0.955524	0.019758	338905.8	335215	Down	-0.0158	0.990298 Isoform 9 of cAMP-specific 3',5'-cyclic phosphodiesterase 4D OS=Mus musculus GN=Pde4d
Myef2	Q8C854-3	3	3	189076.3	160186	200600.7	198423	280623.7	78741	213332	186403.7	313834.3	224401.3	0.956353	0.019382	205781.9	203342.5	Down	-0.0172	0.990757 Isoform 2 of Myelin expression factor 2 OS=Mus musculus GN=Myef2
Mrrf	Q9D6S7	1	1	NA	24397	19964	20887	13005	12681	22114	20902	21238	22197	0.932769	0.030226	19563.25	19826.4	Up	0.019277	0.983417 Ribosome-recycling factor, mitochondrial OS=Mus musculus GN=Mrrf PE=1 SV=1
Gstm1	P10649	6	6	661028.2	618884.5	601608.2	707136.5	545771.5	658862.7	680774.8	609771.2	630660.8	568632.7	0.934445	0.029446	626885.8	629740.4	Up	0.006555	0.983847 Glutathione S-transferase Mu 1 OS=Mus musculus GN=Gstm1 PE=1 SV=2
Lamb2	Q61292	1	1	2516	6601	5015	9869	13170	7261	6704	5837	12755	3977	0.958614	0.018356	7434.2	7306.8	Down	-0.02494	0.991596 Laminin subunit beta-2 OS=Mus musculus GN=Lamb2 PE=2 SV=2
Rexo2	Q9D8S4	1	1	NA	42235	16618	32261	8994	NA	15927	15828	34194	32214	0.959049	0.018159	25027	24540.75	Down	-0.02831	0.991596 Oligoribonuclease, mitochondrial OS=Mus musculus GN=Rexo2 PE=1 SV=2
Myef2	Q8C854-2	3	3	161208.7	126739.7	165779.3	165828.7	228874.7	63057	177642.7	147943	264702.3	185598	0.960224	0.017627	169686.2	167788.6	Down	-0.01622	0.991596 Isoform 4 of Myelin expression factor 2 OS=Mus musculus GN=Myef2
Aldh2	P47738	5	5	180507.3	334824.3	267666	399878	294651.3	207857	337649.3	332739.7	210327.3	376036	0.96045	0.017525	295505.4	292921.9	Down	-0.01267	0.991596 Aldehyde dehydrogenase, mitochondrial OS=Mus musculus GN=Aldh2 PE=1 SV=1
Chmp2a	Q9DB34	1	1	NA	41677	107896	97792	119950	75250	103967	130414	104341	39189	0.960519	0.017494	91828.75	90632.2	Down	-0.01892	0.991596 Charged multivesicular body protein 2a OS=Mus musculus GN=Chmp2a PE=1 SV=1
Cox5b	P19536	6	6	1217965	1120888	1042965	1323304	1143667	1369933	1122672	1080237	1070733	1186865	0.960944	0.017302	1169758	1166088	Down	-0.00453	0.991596 Cytochrome c oxidase subunit 5B, mitochondrial OS=Mus musculus GN=Cox5b PE=1 SV=1
Nos1	Q9Z0J4-5	3	3	30328.33	138640	137869	107388	118816.7	97451	114271	117115	107673	105234.3	0.935457	0.028976	106608.4	108348.9	Up	0.023363	0.983847 Isoform NNOS Mu of Nitric oxide synthase, brain OS=Mus musculus GN=Nos1
Hist1h1t	Q07133	4	4	37507.25	112456	207524.5	224081.8	305327.8	24712	183160	165764.3	214128	283265	0.961031	0.017263	177379.5	174205.9	Down	-0.02605	0.991596 Histone H1t OS=Mus musculus GN=Hist1h1t PE=1 SV=4
Wipf3	P0C7L0	5	5	77063.4	77265.8	102259.8	79360	108169.6	119936	101668.6	103390	70675.8	44667.4	0.961618	0.016997	88823.72	88067.56	Down	-0.01233	0.991801 WAS/WASL-interacting protein family member 3 OS=Mus musculus GN=Wipf3 PE=1 SV=1
Ndufa10	Q99LC3	9	9	169429.6	182193.9	142355.9	189085.8	130633.4	181041.3	153854.3	156830	142745.5	175933.4	0.962173	0.016747	162739.7	162080.9	Down	-0.00585	0.991898 NADH dehydrogenase [ubiquinone] 1 alpha subcomplex subunit 10, mitochondrial OS=Mus musculus GN=Ndufa10 PE=1 SV=1
Sparcl1	P70663	4	4	1011278	922605	774280.8	827983.5	836926.8	905697.8	782069.3	941148.5	871569	860815.8	0.963338	0.016221	874614.8	872260.1	Down	-0.00389	0.991898 SPARC-like protein 1 OS=Mus musculus GN=Sparcl1 PE=2 SV=3
Trpv2	Q9WTR1	2	2	1541.5	5023	5221.5	5815	6752	4790.5	6124.5	4252	4986	4600	0.93548	0.028965	4870.6	4950.6	Up	0.023504	0.983847 Transient receptor potential cation channel subfamily V member 2 OS=Mus musculus GN=Trpv2 PE=1 SV=2
Ptcd3	Q14C51	1	1	36666	57538	67823	63967	72367	30761	60638	77177	69262	58162	0.9637	0.016058	59672.2	59200	Down	-0.01146	0.991898 Pentatricopeptide repeat domain-containing protein 3, mitochondrial OS=Mus musculus GN=Ptcd3 PE=2 SV=2
Coro1b	Q9WUM3	2	2	177775	190702	156512	170930	190382	230499	212375	152118	155273	131256	0.964031	0.015909	177260.2	176304.2	Down	-0.0078	0.991898 Coronin-1B OS=Mus musculus GN=Coro1b PE=1 SV=1
Cep170	Q6A065-2	1	1	115133	255810	222777	188789	218275	226964	210144	155885	206489	212145	0.937878	0.027854	200156.8	202325.4	Up	0.015547	0.984512 Isoform 2 of Centrosomal protein of 170 kDa OS=Mus musculus GN=Cep170
Homer3	Q99JP6	1	1	NA	12291	21332	16675	15491	20246	14204	22282	13403	13141	0.940356	0.026708	16447.25	16655.2	Up	0.018126	0.984512 Homer protein homolog 3 OS=Mus musculus GN=Homer3 PE=1 SV=2
Flnb	Q80X90	1	1	NA	35974	32759	59299	96571	58508	39477	43852	73325	61914	0.965409	0.015289	56150.75	55415.2	Down	-0.01902	0.992171 Filamin-B OS=Mus musculus GN=Flnb PE=1 SV=3
Pgm3	Q9CYR6	1	1	NA	168976	157695	195975	182057	142947	221316	180882	169342	172182	0.941329	0.026259	176175.8	177333.8	Up	0.009452	0.984512 Phosphoacetylglucosamine mutase OS=Mus musculus GN=Pgm3 PE=1 SV=1
Rmdn3	Q3UJU9	1	1	35034	37749	31757	37176	34998	38201	34304	36438	32014	35437	0.966542	0.014779	35342.8	35278.8	Down	-0.00261	0.992171 Regulator of microtubule dynamics protein 3 OS=Mus musculus GN=Rmdn3 PE=1 SV=2
Fh1	P97807	18	18	365221.1	382561.9	341373.9	428213.8	329861.3	391574.9	340495.9	345217.9	355752.7	409551.2	0.96749	0.014354	369446.4	368518.5	Down	-0.00363	0.992171 Fumarate hydratase, mitochondrial OS=Mus musculus GN=Fh PE=1 SV=3
Tppp	Q7TQD2	30	30	798825.9	353344.1	341138.7	328112.6	548994.1	529150.7	421925.3	482018.4	427707.4	489633.8	0.967589	0.014309	474083.1	470087.1	Down	-0.01221	0.992171 Tubulin polymerization-promoting protein OS=Mus musculus GN=Tppp PE=1 SV=1
Hspa9	P38647	34	34	403714.3	496516.2	457510.4	528756.9	465767.1	515615.6	466294.5	449136.2	447287.1	483259.1	0.941351	0.026248	470453	472318.5	Up	0.00571	0.984512 Stress-70 protein, mitochondrial OS=Mus musculus GN=Hspa9 PE=1 SV=3
Tagln3	Q9R1Q8	10	10	262562	317613.5	285020.4	273574.5	345687.1	378194.6	313823.3	303986.9	257537	241677.1	0.941891	0.025999	296891.5	299043.8	Up	0.010421	0.984512 Transgelin-3 OS=Mus musculus GN=Tagln3 PE=1 SV=1
Fbll1	Q80WS3	1	1	266358	219567	335198	277891	422946	167221	310804	335801	354512	372545	0.942259	0.02583	304392	308176.6	Up	0.017827	0.984512 rRNA/tRNA 2'-O-methyltransferase fibrillarin-like protein 1 OS=Mus musculus GN=Fbll1 PE=2 SV=1
Gstm3	P19639	1	1	84516	86585	99115	102071	84299	57613	103148	117694	102552	73160	0.968533	0.013886	91317.2	90833.4	Down	-0.00766	0.992171 Glutathione S-transferase Mu 3 OS=Mus musculus GN=Gstm3 PE=1 SV=2
Trim9	Q8C7M3	10	10	45353	73575.5	73138.2	57887.6	86752.1	66507.9	60637.6	79256.8	65931.1	67260.3	0.943481	0.025267	67341.28	67918.74	Up	0.012319	0.985388 E3 ubiquitin-protein ligase TRIM9 OS=Mus musculus GN=Trim9 PE=1 SV=2
Cuta	Q9CQ89	1	1	278510	282880	316851	269584	303799	393553	288802	265761	246161	267081	0.946587	0.023839	290324.8	292271.6	Up	0.009642	0.987791 Protein CutA OS=Mus musculus GN=Cuta PE=2 SV=3
Nudt7	Q99P30-5	1	1	109419	56454	94226	43278	107034	104043	68688	132159	66719	45699	0.948012	0.023186	82082.2	83461.6	Up	0.024043	0.987872 Isoform 5 of Peroxisomal coenzyme A diphosphatase NUDT7 OS=Mus musculus GN=Nudt7
Micu3	Q9CTY5	2	2	158917	156436.5	215043	222960	225885	175662.5	213369	231241.5	164260.5	201339.5	0.948557	0.022937	195848.3	197174.6	Up	0.009737	0.987872 Calcium uptake protein 3, mitochondrial OS=Mus musculus GN=Micu3 PE=2 SV=2
Tubb1	A2AQ07	5	5	2293263	2846291	2249205	2415873	2398371	1949534	2402572	2412028	2959563	2542385	0.949821	0.022358	2440600	2453216	Up	0.007438	0.988385 Tubulin beta-1 chain OS=Mus musculus GN=Tubb1 PE=1 SV=1
Wdr48	Q8BH57	1	1	NA	52432	115018	133002	92181	61863	129212	90992	137759	78299	0.950513	0.022042	98158.25	99625	Up	0.021398	0.988461 WD repeat-containing protein 48 OS=Mus musculus GN=Wdr48 PE=1 SV=1
Actg2	P63268	16	16	3611555	3269166	3486627	2997194	3185050	3420920	3124601	3465813	3400839	3113757	0.972626	0.012054	3309918	3305186	Down	-0.00206	0.993868 Actin, gamma-enteric smooth muscle OS=Mus musculus GN=Actg2 PE=1 SV=1
Crym	O54983	6	6	86032	137865	123993.8	120819	115188.7	111467.5	98924.67	89923.33	130048.8	151124.8	0.973312	0.011748	116779.7	116297.8	Down	-0.00597	0.993868 Ketimine reductase mu-crystallin OS=Mus musculus GN=Crym PE=1 SV=1
Them4	Q3UUI3	1	1	120762	132862	139209	150385	108772	139871	143881	123407	112914	134785	0.951823	0.021444	130398	130971.6	Up	0.006332	0.988461 Acyl-coenzyme A thioesterase THEM4 OS=Mus musculus GN=Them4 PE=1 SV=1
Vcp	Q01853	25	25	244645.8	192352.8	215291.4	196360.6	203580.5	214873.4	215201.3	218927.8	208462.7	197861.4	0.953407	0.020722	210446.2	211065.3	Up	0.004238	0.989267 Transitional endoplasmic reticulum ATPase OS=Mus musculus GN=Vcp PE=1 SV=4
H2afy	Q9QZQ8	4	4	110858.8	182224	284429	335299	417483.8	130015.3	296417.3	296291.3	310119.5	287029.8	0.974966	0.011011	266058.9	263974.6	Down	-0.01135	0.993868 Core histone macro-H2A.1 OS=Mus musculus GN=H2afy PE=1 SV=3
Spon1	Q8VCC9	2	2	50414	22460	44670	14471	43681	56196	47941	20820.5	27416.5	21712.5	0.975335	0.010846	35139.2	34817.3	Down	-0.01328	0.993868 Spondin-1 OS=Mus musculus GN=Spon1 PE=2 SV=1
Ptrh2	Q8R2Y8	1	1	NA	223861	203878	248368	74193	115406	154680	264355	180525	215510	0.975753	0.01066	187575	186095.2	Down	-0.01143	0.993868 Peptidyl-tRNA hydrolase 2, mitochondrial OS=Mus musculus GN=Ptrh2 PE=2 SV=1
Fam162a	Q9D6U8	1	1	110581	225874	241179	250153	249153	201197	302223	241840	186236	156407	0.953757	0.020562	215388	217580.6	Up	0.014612	0.989267 Protein FAM162A OS=Mus musculus GN=Fam162a PE=2 SV=1
Kdm3b	Q6ZPY7	1	1	48601	36000	22813	26220	26428	20729	39618	39011	28058	31736	0.976099	0.010506	32012.4	31830.4	Down	-0.00823	0.993868 Lysine-specific demethylase 3B OS=Mus musculus GN=Kdm3b PE=2 SV=2
Rps28	P62858	3	3	NA	30665	16782	5581.5	19140	NA	11387.5	30070	11114	18752.5	0.976254	0.010437	18042.13	17831	Down	-0.01698	0.993868 40S ribosomal protein S28 OS=Mus musculus GN=Rps28 PE=2 SV=1
Rpl31	P62900	1	1	507305	413386	293020	293265	365763	519772	297850	405914	336890	328124	0.956773	0.019191	374547.8	377710	Up	0.012129	0.990792 60S ribosomal protein L31 OS=Mus musculus GN=Rpl31 PE=1 SV=1
Gls2	Q571F8	1	1	5155	7930	5303	9565	8731	12842	6682	4359	6297	6966	0.957915	0.018673	7336.8	7429.2	Up	0.018056	0.991573 Glutaminase liver isoform, mitochondrial OS=Mus musculus GN=Gls2 PE=1 SV=2
Rpl17	Q9CPR4	3	3	590702.7	410786.7	349414	335995.7	360176.7	452660	431565	417696.3	365487	392410.7	0.960957	0.017296	409415.1	411963.8	Up	0.008953	0.991596 60S ribosomal protein L17 OS=Mus musculus GN=Rpl17 PE=2 SV=3
Yars	Q91WQ3	2	2	516719	244429.5	305924.5	248581.5	249696	327703.5	329948.5	321085.5	273722	305354	0.978651	0.009372	313070.1	311562.7	Down	-0.00696	0.994353 Tyrosine--tRNA ligase, cytoplasmic OS=Mus musculus GN=Yars PE=2 SV=3
Rpl18a	P62717	2	2	253359	168302	129006.5	195696.5	227645	109923	169375.5	202953	271556.5	215503.5	0.978953	0.009238	194801.8	193862.3	Down	-0.00697	0.994353 60S ribosomal protein L18a OS=Mus musculus GN=Rpl18a PE=1 SV=1
Pklr	P53657	3	3	NA	131489	94095	141827.3	196270.3	194828.7	127306.7	151945.7	125661	110991	0.96364	0.016085	140920.4	142146.6	Up	0.012499	0.991898 Pyruvate kinase PKLR OS=Mus musculus GN=Pklr PE=2 SV=1
Emd	O08579	4	4	127020	102302	92048	95699	95104	123331	100383	106143	95348	88971	0.964208	0.015829	102434.6	102835.2	Up	0.005631	0.991898 Emerin OS=Mus musculus GN=Emd PE=1 SV=1
Decr1	Q9CQ62	2	2	483806	539752.5	595053.5	694551	646972.5	670504	525622.5	487912.5	636225.5	633284	0.980219	0.008677	592027.1	590709.7	Down	-0.00321	0.994971 2,4-dienoyl-CoA reductase, mitochondrial OS=Mus musculus GN=Decr1 PE=1 SV=1
Pcx	Q05920	6	6	265711.8	222233.8	270116.3	278397	222637.8	273800	257798.2	261113.8	209898.2	260266	0.964421	0.015733	251819.4	252575.2	Up	0.004324	0.991898 Pyruvate carboxylase, mitochondrial OS=Mus musculus GN=Pc PE=1 SV=1

Elavl1	P70372	2	2	291579	261290	436018	306763	626062	121192	399434	530946	529762	328600	0.982061	0.007862	384342.4	381986.8	Down	-0.00887	0.995973	ELAV-like protein 1 OS=Mus musculus GN=Elavl1 PE=1 SV=2
Ppp6c	Q9CQR6	1	1	37999	41205	34244	18172	13919	25273	19988	35344	30450	33773	0.982372	0.007724	29107.8	28965.6	Down	-0.00707	0.995973	Serine/threonine-protein phosphatase 6 catalytic subunit OS=Mus musculus GN=Ppp6c PE=2 SV=1
Strn4	P58404	7	7	187877	177522.4	140303.4	188036.4	147860	168842.8	178336.6	156914.4	172038.4	164273.6	0.983178	0.007368	168319.8	168081.2	Down	-0.00205	0.996144	Striatin-4 OS=Mus musculus GN=Strn4 PE=1 SV=2
Nav1	Q8CH77-3	1	1	151685	413006	236639	224302	294527	322118	255751	264304	203927	268983	0.983715	0.007131	264031.8	263016.6	Down	-0.00556	0.996144	Isoform 3 of Neuron navigator 1 OS=Mus musculus GN=Nav1
Ubap2	Q91VX2	1	1	NA	NA	6316	4218	4440	NA	4697	5452	4924	NA	0.966072	0.014991	4991.333	5024.333	Up	0.009507	0.992171	Ubiquitin-associated protein 2 OS=Mus musculus GN=Ubap2 PE=1 SV=1
Naxd	Q9CZ42-3	3	3	NA	396353.5	305189.5	336035	267049.5	354349	277186.5	295597.5	332672	377822.5	0.968366	0.01396	326156.9	327525.5	Up	0.006041	0.992171	Isoform 3 of ATP-dependent (S)-NAD(P)H-hydrate dehydratase OS=Mus musculus GN=Carkd
Rbbp9	O88851	1	1	NA	84137	78245	72512	80275	42531	118461	105551	70291	60146	0.968434	0.01393	78792.25	79396	Up	0.011013	0.992171	Putative hydrolase RBBP9 OS=Mus musculus GN=Rbbp9 PE=1 SV=2
Eef1a1	P10126	21	21	399708.3	352522.1	451099.3	361372.6	395139.2	484710.2	384375.6	333849	405538.3	357752.2	0.968478	0.01391	391968.3	393245.1	Up	0.004692	0.992171	Elongation factor 1-alpha 1 OS=Mus musculus GN=Eef1a1 PE=1 SV=3
Mtmr12	Q80TA6	1	1	NA	6586	5240	3851	7874	NA	6178	5361	8326	3585	0.985262	0.006448	5887.75	5862.5	Down	-0.0062	0.996144	Myotubularin-related protein 12 OS=Mus musculus GN=Mtmr12 PE=2 SV=2
Lgalsl	Q8VED9	2	2	538391	369475.5	317319	330527	388293.5	346898.5	425186	405747.5	398269	376492	0.968804	0.013764	388801.2	390518.6	Up	0.006359	0.992171	Galectin-related protein OS=Mus musculus GN=Lgalsl PE=2 SV=1
Itsn2	Q9Z0R6	1	1	33239	26229	27347	29172	41210	35107	32579	34638	31066	23512	0.986735	0.005799	31439.4	31380.4	Down	-0.00271	0.996659	Intersectin-2 OS=Mus musculus GN=Itsn2 PE=1 SV=2
Acta1	P68134	19	19	3355976	3040056	3238781	2790236	2948741	3172708	2908533	3220562	3159591	2902019	0.986938	0.00571	3074758	3072683	Down	-0.00097	0.996659	Actin, alpha skeletal muscle OS=Mus musculus GN=Acta1 PE=1 SV=1
Mif	P34884	6	6	311788	249430	295497.6	242085.2	260170	315608.8	293167.4	267118.6	219230.6	262225.6	0.988156	0.005174	271794.2	271470.2	Down	-0.00172	0.997496	Macrophage migration inhibitory factor OS=Mus musculus GN=Mif PE=1 SV=2
Gna11	P21278	2	2	54348.5	54042.5	100621.5	81111	100572	105240	90331.5	100537	61302.5	36636	0.968944	0.013701	78139.1	78809.4	Up	0.012323	0.992171	Guanine nucleotide-binding protein subunit alpha-11 OS=Mus musculus GN=Gna11 PE=1 SV=1
Arhgef17	Q80U35	1	1	NA	73927	53182	66999	72478	41298	58963	83445	61016	90489	0.969959	0.013247	66646.5	67042.2	Up	0.00854	0.992814	Rho guanine nucleotide exchange factor 17 OS=Mus musculus GN=Arhgef17 PE=2 SV=2
Tstd3	Q9D0B5	2	2	282279	267648	320619	286880	290749.5	310823	291491.5	312328	302556	229802.5	0.989783	0.00446	289635.1	289400.2	Down	-0.00117	0.99777	Thiosulfate sulfurtransferase/rhodanese-like domain-containing protein 3 OS=Mus musculus GN=Tstd3 PE=1 SV=1
Cttnbp2	B9EJA2-3	1	1	NA	73842	56244	61445	37739	52587	58301	58415	45162	73843	0.970451	0.013026	57317.5	57661.6	Up	0.008635	0.99292	Isoform 3 of Cortactin-binding protein 2 OS=Mus musculus GN=Cttnbp2
Cisd1	Q91WS0	3	3	166622.7	213541.3	187287.7	221505	176990	261040.7	165759.3	187848.7	163279	186896.7	0.991658	0.003638	193189.3	192964.9	Down	-0.00168	0.997872	CDGSH iron-sulfur domain-containing protein 1 OS=Mus musculus GN=Cisd1 PE=1 SV=1
Kcnab2	P62482	4	4	258154.3	319255	267020.7	349114.7	246077.3	259649.7	298466.3	274306.7	309998.7	301248	0.971721	0.012458	287924.4	288733.9	Up	0.00405	0.993666	Voltage-gated potassium channel subunit beta-2 OS=Mus musculus GN=Kcnab2 PE=2 SV=1
Rgs9	O54828	2	2	145526.5	89462	85079	75195	83304	120007.5	84059	86318	97996	92790	0.971955	0.012354	95713.3	96234.1	Up	0.007829	0.993666	Regulator of G-protein signaling 9 OS=Mus musculus GN=Rgs9 PE=1 SV=3
Canx	P35564	11	11	92309.67	169896.1	175022.8	165498.3	197566.4	179670.3	162477.4	174975.3	144316.6	137874.7	0.992382	0.003321	160058.7	159862.9	Down	-0.00177	0.997872	Calnexin OS=Mus musculus GN=Canx PE=1 SV=1
Usp14	Q9JMA1	1	1	NA	243435	189178	267513	184352	246604	180864	210368	204828	261705	0.992541	0.003252	221119.5	220873.8	Down	-0.0016	0.997872	Ubiquitin carboxyl-terminal hydrolase 14 OS=Mus musculus GN=Usp14 PE=1 SV=3
Itsn1	Q9Z0R4	6	6	142435	151998.4	144022	142665.6	151936.6	137890.6	154444.4	171572.4	143172	127356.8	0.973509	0.01166	146611.5	146887.2	Up	0.002711	0.993868	Intersectin-1 OS=Mus musculus GN=Itsn1 PE=1 SV=2
Spryd7	Q3TFQ1	1	1	NA	21666	14829	12525	10767	NA	16759	11243	16680	15012	0.993527	0.00282	14946.75	14923.5	Down	-0.00225	0.997872	SPRY domain-containing protein 7 OS=Mus musculus GN=Spryd7 PE=2 SV=2
Tpm1	P58771	3	3	NA	48974	8622	36844.5	17254	NA	5569	15484.5	64827	NA	0.974779	0.011094	27923.63	28626.83	Up	0.035882	0.993868	Tropomyosin alpha-1 chain OS=Mus musculus GN=Tpm1 PE=1 SV=1
Cct7	P80313	9	9	78820.62	98729.5	85114.38	99950.38	90924	80208.25	99170.88	87395.88	97668.25	89920.25	0.976004	0.010548	90707.78	90872.7	Up	0.002621	0.993868	T-complex protein 1 subunit eta OS=Mus musculus GN=Cct7 PE=1 SV=1
Ncln	Q8VCM8	1	1	73450	59960	61527	50481	61016	42100	59380	69604	71497	63608	0.994094	0.002573	61286.8	61237.8	Down	-0.00115	0.997872	Nicalin OS=Mus musculus GN=Ncln PE=2 SV=2
Pdia3	P27773	17	17	748308.4	617615.8	464276.1	456107.6	580347.2	691478.1	543978.1	621348.6	467042.6	540387.6	0.994368	0.002453	573331	572847	Down	-0.00122	0.997872	Protein disulfide-isomerase A3 OS=Mus musculus GN=Pdia3 PE=1 SV=2
Fut8	Q9WTS2	1	1	NA	89072	84488	60867	108741	86177	84460	95311	88433	74221	0.994864	0.002236	85792	85720.4	Down	-0.0012	0.997939	Alpha-(1,6)-fucosyltransferase OS=Mus musculus GN=Fut8 PE=2 SV=2
Ppp1r12c	Q3UMT1	2	2	29552	18287	21498	16146	23806	26206	21667	22989	20072	18760	0.976657	0.010258	21857.8	21938.8	Up	0.005336	0.993868	Protein phosphatase 1 regulatory subunit 12C OS=Mus musculus GN=Ppp1r12c PE=1 SV=1
Stt3b	Q3TDQ1	1	1	NA	22319	24970	16420	18972	7326	27712	22896	19319	26725	0.976805	0.010192	20670.25	20795.6	Up	0.008722	0.993868	Dolichyl-diphosphooligosaccharide--protein glycosyltransferase subunit STT3B OS=Mus musculus GN=Stt3b PE=1 SV=2
Crmp1	P97427	23	23	1217565	906480.5	734731.1	902891.8	816117.8	949636.5	843991.4	848842.5	934749.2	998889.4	0.997078	0.001271	915557.2	915221.8	Down	-0.00053	0.998668	Dihydropyrimidinase-related protein 1 OS=Mus musculus GN=Crmp1 PE=1 SV=1
Cdc42ep4	Q9JM96	1	1	NA	35125	31712	112344	51673	42612	NA	32948	108017	50200	0.977835	0.009734	57713.5	58444.25	Up	0.018152	0.994353	Cdc42 effector protein 4 OS=Mus musculus GN=Cdc42ep4 PE=1 SV=1
Mapre2	Q8R001	3	3	37466.67	47426	37593	40038.33	46024	54222	35429	34913.67	43660.33	40881.33	0.979065	0.009188	41709.6	41821.27	Up	0.003857	0.994353	Microtubule-associated protein RP/EB family member 2 OS=Mus musculus GN=Mapre2 PE=1 SV=1
Cacna2d2	Q6PHS9-3	2	2	NA	71999	73424.5	53563.5	33451	40867.5	58067	60377	67434	63662.5	0.997984	0.000876	58109.5	58081.6	Down	-0.00069	0.998764	Isoform 3 of Voltage-dependent calcium channel subunit alpha-2/delta-2 OS=Mus musculus GN=Cacna2d2
Scamp5	Q9JKD3	1	1	NA	182726	158421	250034	202368	205211	157921	189753	222547	216265	0.998391	0.000699	198387.3	198339.4	Down	-0.00035	0.998781	Secretory carrier-associated membrane protein 5 OS=Mus musculus GN=Scamp5 PE=2 SV=1
Huwe1	Q7TMY8-3	3	3	21296.33	65449.33	85488.67	74193.67	89370	40397	77598.67	79701.33	63054	74960.33	0.999064	0.000407	67159.6	67142.27	Down	-0.00037	0.999064	Isoform 3 of E3 ubiquitin-protein ligase HUWE1 OS=Mus musculus GN=Huwe1
Dnm2	P39054	10	10	450470.3	601574.3	700424	631649.1	538765.5	502372.4	719172.5	540654.7	658254.7	510584.3	0.979223	0.009118	584576.6	586207.7	Up	0.00402	0.994353	Dynamin-2 OS=Mus musculus GN=Dnm2 PE=1 SV=2
Exoc6b	A6H5Z3	1	1	NA	17888	20106	7840	15687	NA	NA	NA	7986	NA		#NUM!	15380.25	7986	Down	-0.94553		Exocyst complex component 6B OS=Mus musculus GN=Exoc6b PE=2 SV=1
Nhsl2	B1AXH1	1	1	NA	13229	7859	NA	6623	NA	NA	NA	7103	NA		#NUM!	9237	7103	Down	-0.379		NHS-like protein 2 OS=Mus musculus GN=Nhsl2 PE=2 SV=1
Zfand4	D3Z3C6	1	1	NA	NA	NA	13068	NA	NA	NA	NA	NA	NA		#NUM!	13068	#DIV/0!	#DIV/0!	#DIV/0!		AN1-type zinc finger protein 4 OS=Mus musculus GN=Zfand4 PE=4 SV=1
Hpca	P84075	1	1	127146	101040	95268	76286	105091	120513	101875	109334	85992	88411	0.980856	0.008395	100966.2	101225	Up	0.003693	0.995224	Neuron-specific calcium-binding protein hippocalcin OS=Mus musculus GN=Hpca PE=1 SV=2
Gbas	O55126	1	1	NA	NA	NA	NA	NA	NA	10061	NA	NA	NA		#NUM!	#DIV/0!	10061	#DIV/0!	#DIV/0!		Protein NipSnap homolog 2 OS=Mus musculus GN=Gbas PE=2 SV=1
Wnt1	P04426	1	1	NA	NA	64264	NA	NA	NA	NA	NA	NA	NA		#NUM!	64264	#DIV/0!	#DIV/0!	#DIV/0!		Proto-oncogene Wnt-1 OS=Mus musculus GN=Wnt1 PE=1 SV=1
Ccny	Q8BGU5	1	1	99352	188629	224813	231582	270279	167531	287281	201525	206602	155324	0.984951	0.006585	202931	203652.6	Up	0.005121	0.996144	Cyclin-Y OS=Mus musculus GN=Ccny PE=1 SV=1
Trhr	P21761	1	1	NA	NA	NA	NA	NA	NA	NA	10935	50010	NA		#NUM!	#DIV/0!	30472.5	#DIV/0!	#DIV/0!		Thyrotropin-releasing hormone receptor OS=Mus musculus GN=Trhr PE=2 SV=1
Agk	Q9ESW4	2	2	65151.5	164675	158942	208356.5	180880.5	126494	143430.5	139253	202741.5	168791.5	0.985028	0.006551	155601.1	156142.1	Up	0.005007	0.996144	Acylglycerol kinase, mitochondrial OS=Mus musculus GN=Agk PE=1 SV=1
Psmd7	P26516	2	2	NA	NA	NA	NA	NA	NA	NA	57915	NA	27024		#NUM!	#DIV/0!	42469.5	#DIV/0!	#DIV/0!		26S proteasome non-ATPase regulatory subunit 7 OS=Mus musculus GN=Psmd7 PE=1 SV=2
Mff	Q6PCP5-3	7	7	56519.83	117953.7	92893	99650.67	113468.8	129727.8	92967.5	94428.33	75558.17	89165.17	0.985098	0.006521	96097.2	96369.4	Up	0.004081	0.996144	Isoform 3 of Mitochondrial fission factor OS=Mus musculus GN=Mff
Slc6a1	P31648	2	2	NA	NA	NA	NA	NA	NA	NA	14714	32485.5	NA		#NUM!	#DIV/0!	23599.75	#DIV/0!	#DIV/0!		Sodium- and chloride-dependent GABA transporter 1 OS=Mus musculus GN=Slc6a1 PE=1 SV=2
Mrps6	P58064	1	1	NA	NA	NA	10537	NA	NA	NA	NA	NA	NA		#NUM!	10537	#DIV/0!	#DIV/0!	#DIV/0!		28S ribosomal protein S6, mitochondrial OS=Mus musculus GN=Mrps6 PE=2 SV=3
Vps33b	P59016	1	1	NA	NA	NA	NA	NA	NA	NA	16175	NA	2504		#NUM!	#DIV/0!	9339.5	#DIV/0!	#DIV/0!		Vacuolar protein sorting-associated protein 33B OS=Mus musculus GN=Vps33b PE=1 SV=1
Inpp5j	P59644	1	1	NA	NA	11588	12384	17282	NA	NA	NA	NA	NA		#NUM!	13751.33	#DIV/0!	#DIV/0!	#DIV/0!		Phosphatidylinositol 4,5-bisphosphate 5-phosphatase A OS=Mus musculus GN=Inpp5j PE=2 SV=2
Pdia4	P08003	4	4	190110.5	141133.3	205135.3	148134	186123.8	212995.5	172119.3	200847.3	168751.8	117918.3	0.9851	0.00652	174127.4	174526.4	Up	0.003302	0.996144	Protein disulfide-isomerase A4 OS=Mus musculus GN=Pdia4 PE=1 SV=3
Tceb2	P62869	1	1	NA	NA	6156	NA	NA	NA	NA	5298	6764	6357		#NUM!	6156	6139.667	Down	-0.00383		Transcription elongation factor B polypeptide 2 OS=Mus musculus GN=Tceb2 PE=1 SV=1
Htra1	Q9R118	4	4	112225.5	125463	102980	104474.5	85656.5	96697	95752	120791	88616	129828.5	0.98673	0.005802	106159.9	106336.9	Up	0.002403	0.996659	Serine protease HTRA1 OS=Mus musculus GN=Htra1 PE=1 SV=2
Dctn4	Q8CBY8	3	3	225440	153910.5	149198	174579.5	136697.5	182873	168814.5	202597.5	144202	142774	0.988563	0.004996	167965.1	168252.2	Up	0.002464	0.997515	Dynactin subunit 4 OS=Mus musculus GN=Dctn4 PE=1 SV=1
Sms	P97355	2	2	NA	NA	NA	NA	NA	NA	NA	16705	26187	NA		#NUM!	#DIV/0!	21446	#DIV/0!	#DIV/0!		Spermine synthase OS=Mus musculus GN=Sms PE=1 SV=1
C1qa	P98086	1	1	NA	NA	NA	13176	NA	NA	NA	NA	NA	NA		#NUM!	13176	#DIV/0!	#DIV/0!	#DIV/0!		Complement C1q subcomponent subunit A OS=Mus musculus GN=C1qa PE=1 SV=2
Svip	Q3UZP4	1	1	NA	NA	26038	12341	NA	NA	NA	NA	NA	NA		#NUM!	19189.5	#DIV/0!	#DIV/0!	#DIV/0!		Small VCP/p97-interacting protein OS=Mus musculus GN=Svip PE=2 SV=1
Ermn	Q5EBJ4	1	1	NA	NA	NA	NA	NA	NA	NA	18818	36154	NA		#NUM!	#DIV/0!	27486	#DIV/0!	#DIV/0!		Ermin OS=Mus musculus GN=Ermn PE=1 SV=1
Arhgdib	Q61599	1	1	NA	NA	NA	NA	NA	NA	NA	8345	13352	NA		#NUM!	#DIV/0!	10848.5	#DIV/0!	#DIV/0!		Rho GDP-dissociation inhibitor 2 OS=Mus musculus GN=Arhgdib PE=1 SV=3
Ndrg1	Q62433	2	2	NA	NA	NA	NA	NA	NA	NA	NA	13432	14974		#NUM!	#DIV/0!	14203	#DIV/0!	#DIV/0!		Protein NDRG1 OS=Mus musculus GN=Ndrg1 PE=1 SV=1
Ttbk1	Q6PCN3	2	2	242007	171295	191495.5	169868	256574	181811	225324.5	264133	176745.5	184886	0.989608	0.004537	206247.9	206580	Up	0.002321	0.99777	Tau-tubulin kinase 1 OS=Mus musculus GN=Ttbk1 PE=2 SV=3
Rab3ip	Q68EF0	1	1	NA	7757	NA	NA	NA	NA	NA	NA	5705	NA		#NUM!	7757	5705	Down	-0.44327		Rab-3A-interacting protein OS=Mus musculus GN=Rab3ip PE=1 SV=1
Sv2c	Q69ZS6	1	1	NA	NA	8182	6931	7704	NA	NA	NA	NA	NA		#NUM!	7605.667	#DIV/0!	#DIV/0!	#DIV/0!		Synaptic vesicle glycoprotein 2C OS=Mus musculus GN=Sv2c PE=1 SV=2
Hnrnpa1	P49312	4	4	44930	31715	44399	21486.5	100020.5	39524.5	41555	43882	72418	46148	0.989984	0.004372	48510.2	48705.5	Up	0.005797	0.99777	Heterogeneous nuclear ribonucleoprotein A1 OS=Mus musculus GN=Hnrnpa1 PE=1 SV=2
Lrfn4	Q80XU8	2	2	NA	NA	NA	NA	NA	NA	NA	15194	NA	NA		#NUM!	#DIV/0!	15194	#DIV/0!	#DIV/0!		Leucine-rich repeat and fibronectin type-III domain-containing protein 4 OS=Mus musculus GN=Lrfn4 PE=1 SV=1
Slitrk5	Q810B7	1	1	NA	NA	NA	NA	NA	NA	28720	NA	NA	NA		#NUM!	#DIV/0!	28720	#DIV/0!	#DIV/0!		SLIT and NTRK-like protein 5 OS=Mus musculus GN=Slitrk5 PE=2 SV=1
Sv2b	Q8BG39	2	2	NA	NA	NA	62993	NA	NA	NA	NA	56595.5	65839		#NUM!	62993	61217.25	Down	-0.04125		Synaptic vesicle glycoprotein 2B OS=Mus musculus GN=Sv2b PE=1 SV=1
Lonp1	Q8CGK3	5	5	301910.2	328007.8	234467.6	291322.4	218667.6	287263.2	266557.6	224105.4	272958.8	324899.6	0.991754	0.003596	274875.1	275156.9	Up	0.001478	0.997872	Lon protease homolog, mitochondrial OS=Mus musculus GN=Lonp1 PE=1 SV=2
Exog	Q8C163	1	1	208649	194311	159129	187258	131434	227721	226884	68299	205442	154130	0.9922	0.003401	176156.2	176495.2	Up	0.002774	0.997872	Nuclease EXOG, mitochondrial OS=Mus musculus GN=Exog PE=2 SV=1
Naa30	Q8CES0	1	1	NA	NA	NA	NA	NA	41915	108140	15502	NA	NA		#NUM!	#DIV/0!	55185.67	#DIV/0!	#DIV/0!		N-alpha-acetyltransferase 30 OS=Mus musculus GN=Naa30 PE=1 SV=2
Ank2	Q8C8R3-6	7	7	205252.1	389444.7	284221.1	242990.9	313853.4	312339.1	282422.4	310636.6	275220.9	256739.7	0.992717	0.003175	287152.5	287471.7	Up	0.001603	0.997872	Isoform 6 of Ankyrin-2 OS=Mus musculus GN=Ank2
Atp1a3	Q6PIC6	68	68	183719.5	328737.4	443004.5	408733.8	388027.7	332642.2	376737	394252.2	344903.1	305743.1	0.993541	0.002814	350444.6	350855.5	Up	0.001691	0.997872	Sodium/potassium-transporting ATPase subunit alpha-3 OS=Mus musculus GN=Atp1a3 PE=1 SV=1
Cdc42ep1	Q91W92	1	1	NA	NA	NA	NA	NA	NA	NA	22613	NA	NA		#NUM!	#DIV/0!	22613	#DIV/0!	#DIV/0!		Cdc42 effector protein 1 OS=Mus musculus GN=Cdc42ep1 PE=1 SV=1
Gcsh	Q91WK5	1	1	NA	NA	NA	NA	NA	NA	NA	10085	33118	NA		#NUM!	#DIV/0!	21601.5	#DIV/0!	#DIV/0!		Glycine cleavage system H protein, mitochondrial OS=Mus musculus GN=Gcsh PE=1 SV=2
Dlg2	Q91XM9-5	2	2	NA	NA	NA	NA	NA	NA	22306	44549	19925	NA		#NUM!	#DIV/0!	28926.67	#DIV/0!	#DIV/0!		Isoform 5 of Disks large homolog 2 OS=Mus musculus GN=Dlg2
Atp5j2	P56135	3	3	NA	113045.3	207901.7	135062.7	173176.7	174927.7	152485.3	163945.3	156477.7	139555.3	0.993795	0.002703	157296.6	157478.3	Up	0.001665	0.997872	ATP synthase subunit f, mitochondrial OS=Mus musculus GN=Atp5j2 PE=1 SV=3
Nenf	Q9CQ45	1	1	NA	NA	NA	NA	21920	NA	NA	NA	NA	NA		#NUM!	21920	#DIV/0!	#DIV/0!	#DIV/0!		Neudesin OS=Mus musculus GN=Nenf PE=1 SV=1
Nck1	Q99M51	2	2	105597	123568.5	154390.5	122495	161339	159033.5	135023.5	138111	128868.5	106770.5	0.995214	0.002084	133478	133561.4	Up	0.000901	0.997939	Cytoplasmic protein NCK1 OS=Mus musculus GN=Nck1 PE=1 SV=1
Fam171b	Q14CH0	4	4	191729.3	228263.3	168024.8	183497.5	262045.3	322107.5	178059.5	185848.5	189801.8	158540	0.996389	0.001571	206712	206871.5	Up	0.001112	0.998668	Protein FAM171B OS=Mus musculus GN=Fam171b PE=1 SV=2
Tmem55a	Q9CZX7	1	1	NA	NA	NA	NA	NA	9358	9928	NA	NA	5956		#NUM!	#DIV/0!	8414	#DIV/0!	#DIV/0!		Type 2 phosphatidylinositol 4,5-bisphosphate 4-phosphatase OS=Mus musculus GN=Tmem55a PE=1 SV=1
Zfp292	Q9Z2U2	1	1	NA	353520	284926	386402	244225	474947	337667	197893	327690	249172	0.99723	0.001205	317268.3	317473.8	Up	0.000934	0.998668	Zinc finger protein 292 OS=Mus musculus GN=Zfp292 PE=1 SV=2
Chmp4b	Q9D8B3	1	1	NA	NA	NA	NA	NA	NA	NA	100122	39837	NA		#NUM!	#DIV/0!	69979.5	#DIV/0!	#DIV/0!		Charged multivesicular body protein 4b OS=Mus musculus GN=Chmp4b PE=1 SV=2
Tsc22d4	Q9EQN3	1	1	NA	NA	NA	NA	NA	NA	NA	NA	11360	NA		#NUM!	#DIV/0!	11360	#DIV/0!	#DIV/0!		TSC22 domain family protein 4 OS=Mus musculus GN=Tsc22d4 PE=1 SV=2
Abcb10	Q9JI39	2	2	NA	NA	NA	NA	NA	NA	NA	NA	12473	NA		#NUM!	#DIV/0!	12473	#DIV/0!	#DIV/0!		ATP-binding cassette sub-family B member 10, mitochondrial OS=Mus musculus GN=Abcb10 PE=2 SV=1
Acot2	Q9QYR9	1	1	NA	NA	NA	NA	NA	NA	NA	NA	11257	NA		#NUM!	#DIV/0!	11257	#DIV/0!	#DIV/0!		Acyl-coenzyme A thioesterase 2, mitochondrial OS=Mus musculus GN=Acot2 PE=1 SV=2
Wdr54	Q9R0D8	1	1	NA	NA	6909	NA	NA	NA	6637	NA	6603	7238		#NUM!	6909	6826	Down	-0.01744		WD repeat-containing protein 54 OS=Mus musculus GN=Wdr54 PE=2 SV=1
Hnrnpab	Q99020	2	2	216729.5	159420.5	170164	165358	201149	212553.5	162378	197536.5	160692.5	179903.5	0.997499	0.001088	182564.2	182612.8	Up	0.000384	0.998668	Heterogeneous nuclear ribonucleoprotein A/B OS=Mus musculus GN=Hnrnpab PE=1 SV=1
Atp6v1c1	Q9Z1G3	1	1	NA	NA	NA	NA	NA	NA	NA	15259	3454	NA		#NUM!	#DIV/0!	9356.5	#DIV/0!	#DIV/0!		V-type proton ATPase subunit C 1 OS=Mus musculus GN=Atp6v1c1 PE=1 SV=4
